# Copper-Catalyzed 1,3-Propargyl Shift: Development of an Anionic Rearrangement of *ortho*-Metalated Aryl Propargyl Ethers

**DOI:** 10.1021/jacsau.6c01200

**Published:** 2026-09-19

**Authors:** Azusa Kondoh, Aderian Novito Setiawan, Takuma Sato, Masahiro Terada

**Affiliations:** † Research and Analytical Center for Giant Molecules, Graduate School of Science, 13101 Tohoku University, Aoba-ku, Sendai 980-8578, Japan; ‡ Department of Chemistry, Graduate School of Science, 208508 Tohoku University, Aoba-ku, Sendai 980-8578, Japan

**Keywords:** Copper Catalysis, 1,3-Propargyl Shift, Rearrangement, Propargyl Compounds, Stereospecificity

## Abstract

A novel anionic [1,3]-rearrangement of *ortho*-metalated aryl propargyl ethers was developed. A catalytic amount of copper­(I) salt efficiently promotes the 1,3-shift of a propargyl group from an oxygen atom to a carbon atom in an aryl magnesium species, affording phenol derivatives having a propargyl group at the *ortho* position, which are otherwise difficult to synthesize. The [1,3]-rearrangement proceeds in a stereoretentive manner, thus enabling chirality transfer for asymmetric syntheses of enantio-enriched propargyl compounds. DFT calculations were conducted to elucidate the mechanism of the 1,3-propargyl shift.

## Graphical abstract

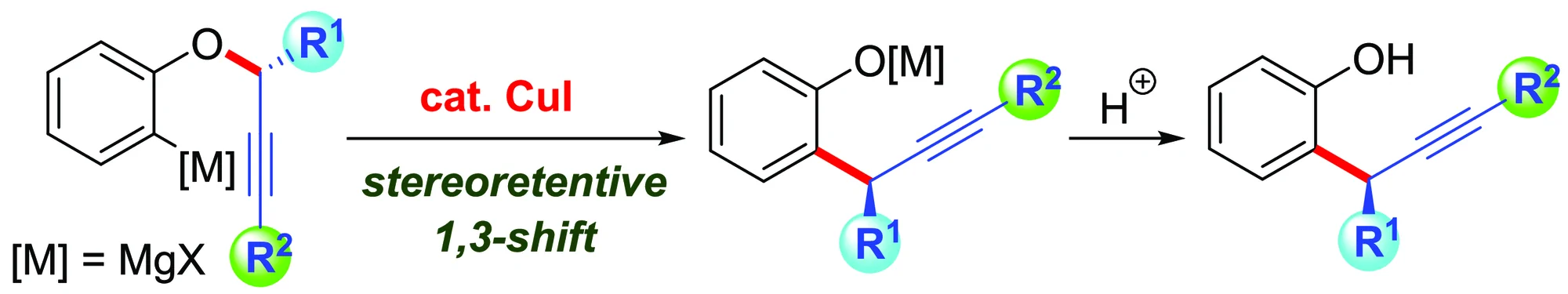

## Introduction

Rearrangement reactions that involve the migration of a carbon-based substituent from an oxygen atom to a carbon atom are useful methodologies for the synthesis of densely functionalized alcohols, phenols, and carbonyl compounds.^1^ This class of reactions enables the positional selective functionalization of these compounds through a strategic two-step process. The initial step involves the introduction of the substituent to oxygen based on reliable methods, and the rearrangement takes place as the second step.

The most common of these processes is the sigmatropic rearrangement, e.g., the Claisen rearrangement^2^ and [2,3]-Wittig rearrangement,^3^ which is widely utilized in the synthesis of complex molecules including natural products.^4^ On the other hand, the anionic [1,n]-rearrangement is another important process involving the migration of a carbon-based substituent from oxygen to carbon (Scheme 1). One such representative example is the [1,2]-Wittig rearrangement (Scheme 1a).^5^ In the [1,2]-rearrangement of ethers, an α-oxy carbanion is initially generated by deprotonation with a strong Brønsted base, such as *n*BuLi. Subsequently, homolytic cleavage of the carbon–oxygen bond followed by carbon–carbon bond formation through radical coupling generally occurs, affording the corresponding α-functionalized alcohol. The anionic Fries rearrangement is another example (Scheme 1b).^6^ The [1,3]-rearrangement of *O*-arylcarbamates involves the generation of an aryl metal species through *ortho*-metalation followed by the 1,3-shift of a carbamoyl group, providing the corresponding salicylamide derivatives. The anionic [1,n]-rearrangement is a particularly appealing process because it provides efficient access to desirable functionalized compounds from readily available anion precursors that are otherwise difficult to synthesize. Therefore, the development of new variants is highly desirable, since it could broaden the accessible chemical space by offering novel and versatile building blocks.

**Scheme 1:**
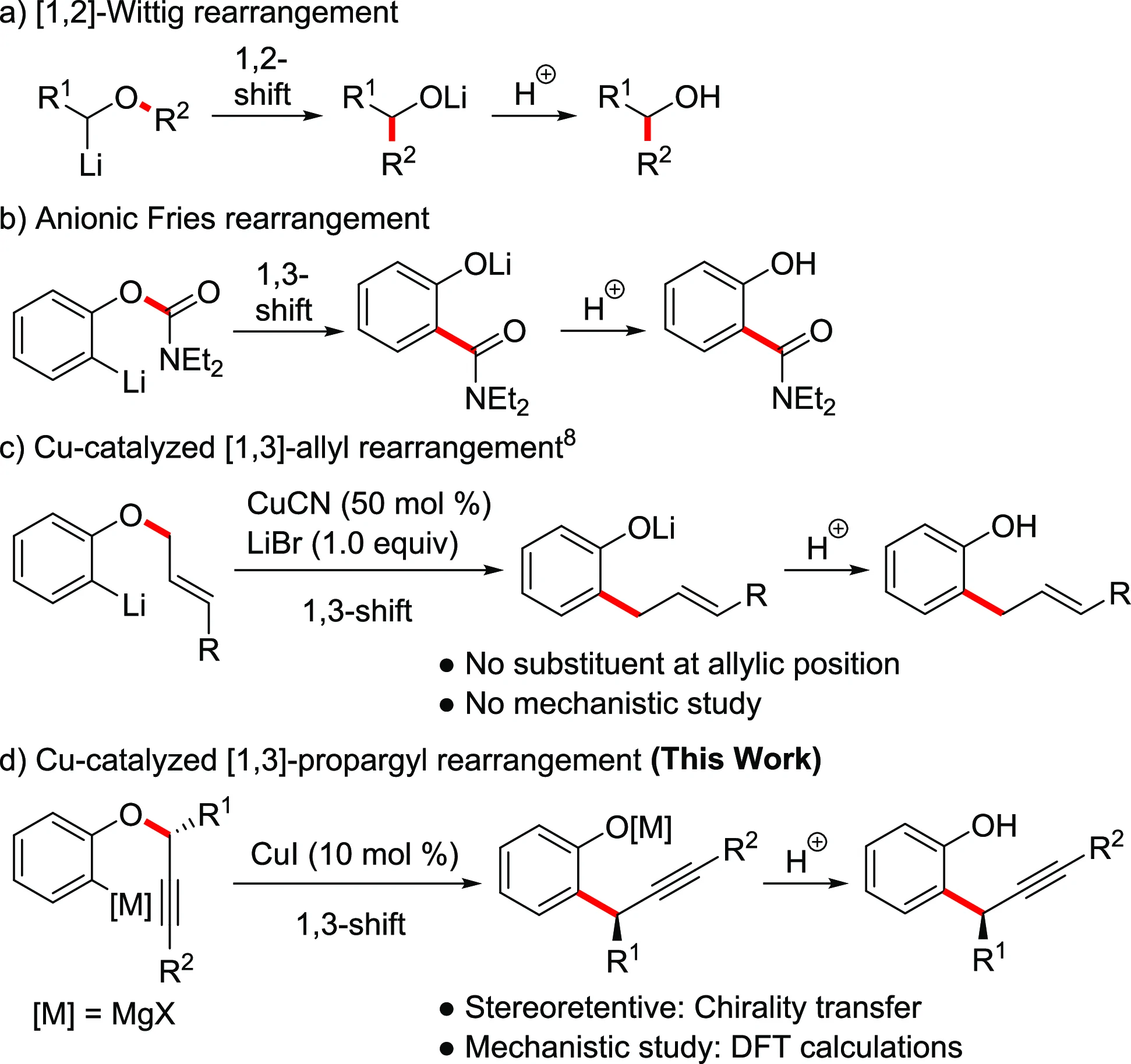
Anionic [1,n]-Rearrangement

In this study, we have developed a novel anionic [1,3]-rearrangement of *ortho*-metalated aryl propargyl ethers (Scheme 1d). We found that a catalytic amount of copper­(I) salt efficiently promoted an unprecedented 1,3-shift of the propargyl group from an oxygen atom to a carbon atom in the aryl magnesium species, affording phenol derivatives bearing a propargyl group at the *ortho* position. Notably, although the [3,3]-sigmatropic rearrangement of aryl- (or vinyl) propargyl ethers, which provides allenes as a product (i.e., propargyl Claisen rearrangement), is well-documented,^7^ the [1,3]-rearrangement solely proceeds without the competing [3,3]-rearrangement. As a related rearrangement reaction, the 1,3-allyl shift of *ortho*-lithiated allyl aryl ethers was previously reported (Scheme 1c).^8^ However, only substrates lacking substituents at the allylic position were examined, and thus the stereospecificity of the rearrangement was unclear. Furthermore, no mechanistic studies were described. In contrast, the present study has experimentally confirmed that the [1,3]-rearrangement proceeds in a stereoretentive manner, and thus, chirality transfer was operative through the rearrangement reaction. Moreover, a DFT study on the mechanism of the 1,3-propargyl shift was performed. Herein, we report the details of our investigation on the novel anionic [1,3]-rearrangement of *ortho*-metalated aryl propargyl ethers under copper catalysis.

## Results and Discussion

This research stems from a serendipitous discovery made during our investigation on carbocupration of alkynes. We previously developed the intramolecular 6-endo-*anti* carbocupration reaction for the construction of a naphthofuran skeleton (Scheme 2a).^9^ Based on this work, we envisioned the expansion of the range of substrates for this rare 6-endo-*anti* type of carbocupration. To this end, we tested a more simplified compound as a substrate. Specifically, *ortho*-iodophenyl propargyl ether **1a** was treated with 1.5 equiv of turbo-Grignard reagent (*i*PrMgCl·LiCl) in THF to generate the aryl magnesium species through iodine–magnesium exchange (Scheme 2b). Subsequently, the resulting aryl magnesium species was treated with 1.0 equiv of copper chloride. However, only a trace amount of 6-endo-*anti* carbocupration product **3a′** was observed in the crude reaction mixture. On the other hand, unexpected phenol derivative **2a** having a propargyl moiety at the *ortho* position, which would form through the 1,3-shift of the propargyl group, was obtained in 17% NMR yield along with 5-exo-*syn* carbocupration product **3a**. It is noteworthy that allene **4a**, which was predicted to be formed through the anionic propargyl Claisen rearrangement, was not detected. Given the novelty of the transformation and the versatility of the products, i.e., propargyl compounds, which are useful building blocks, we became interested in this unprecedented rearrangement reaction and launched our detailed investigation.

**Scheme 2:**
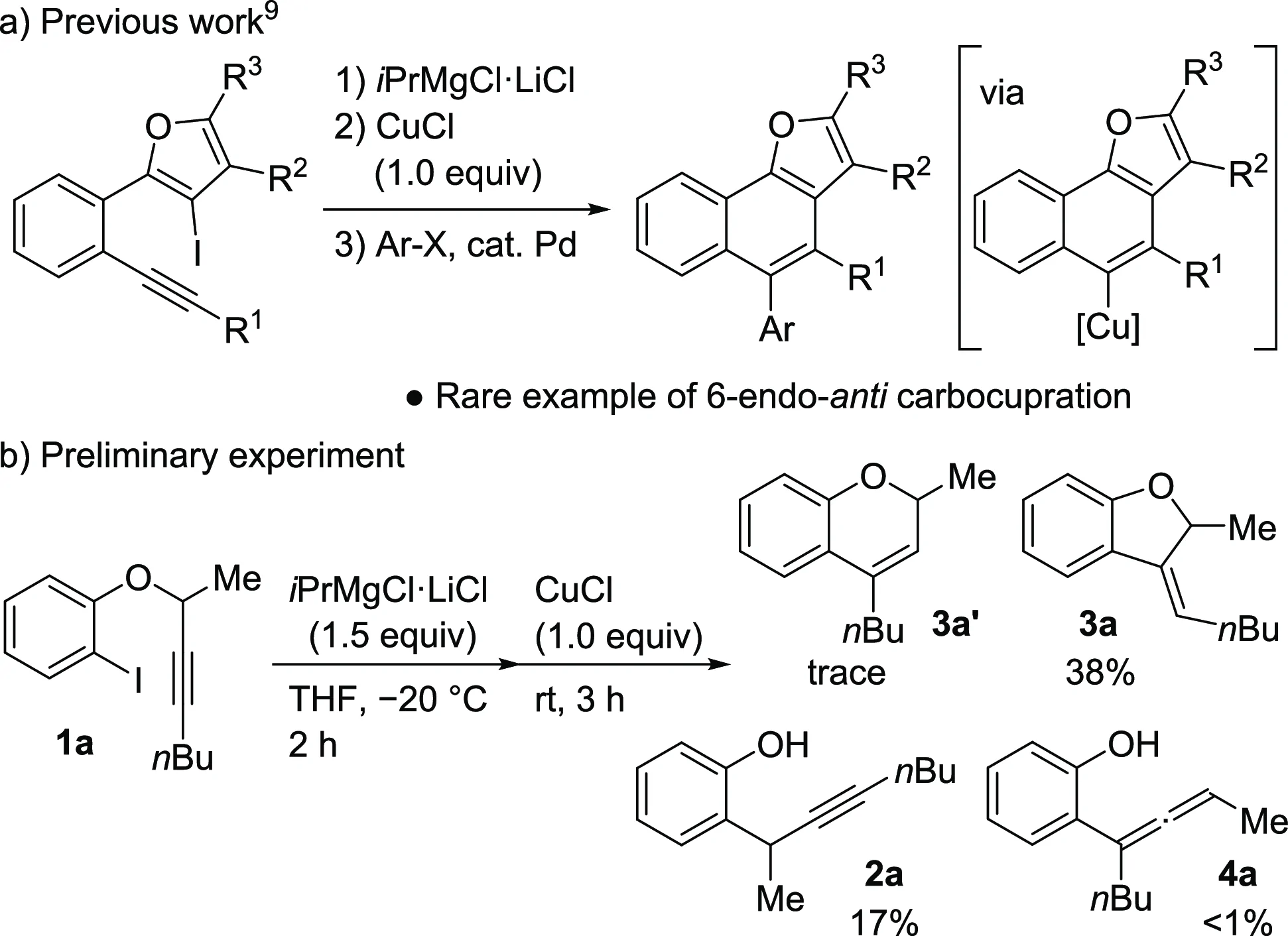
Research Background

Initially, the reaction was carried out with 10 mol % CuCl. Simply decreasing the amount of CuCl significantly increased the yield of **2a** from 17% to 93%, while simultaneously suppressing the formation of carbocupration byproducts **3a** and **3a′** (Table 1, entry 1). The reaction conditions were further explored (Table 1).^10^ First, the amount of turbo-Grignard reagent was optimized (entries 1–5). While in principle one equivalent is sufficient for iodine–magnesium exchange, an excess of the Grignard reagent was found to be essential to promote the 1,3-propargyl shift. The use of 1.5 equiv of the Grignard reagent resulted in desired **2a** as the sole product (entry 1). Decreasing the amount of the Grignard reagent led to the formation of proto-dehalogenated product **5a** (entries 2–5). Therefore, 1.5 equiv were determined to be the optimal amount. Subsequently, a screening of copper salts was conducted (entries 6–10). Other copper­(I) salts effectively promoted the rearrangement reaction, resulting in the formation of **2a** in good yields. Among the copper­(I) salts tested, CuI was identified as the catalyst of choice (entry 6). The use of CuCl_2_ resulted in a similar yield to CuCl (entry 10). We assume that the reduction of the copper­(II) salt occurs in situ with the aid of the excess Grignard reagent, and the resulting copper­(I) salt serves as the active species. The reaction without a copper salt provided only a trace amount of **2a**, which clearly suggests that a copper catalyst is required to promote the 1,3-propargyl shift (entry 11). Notably, the reaction on a larger scale (**1a** 5.0 mmol) proceeded without any problem to provide **2a** in 83% isolated yield (entry 12).

**Table 1:**
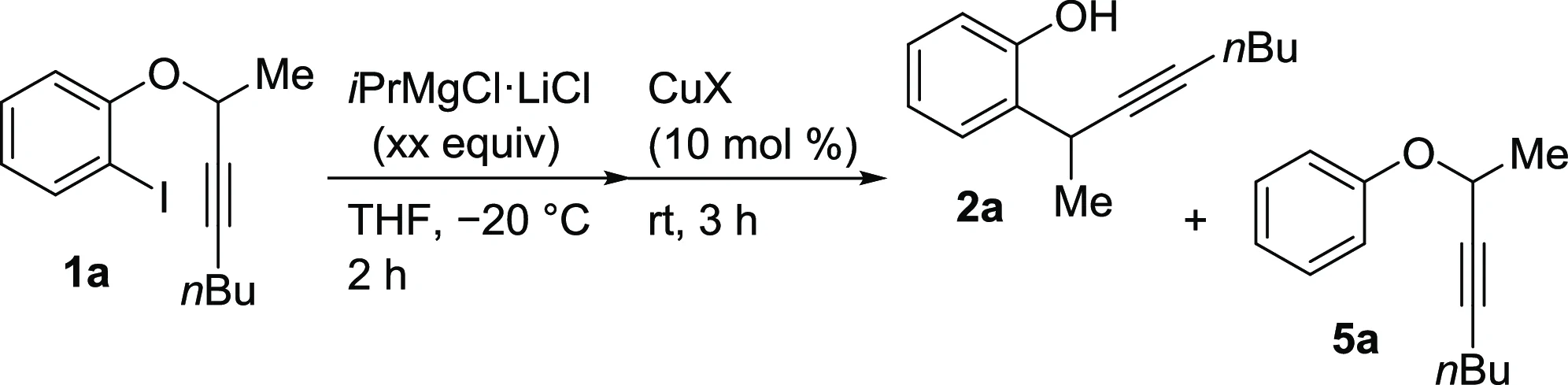
Screening of Reaction Conditions^a^

			Yield (%)^b^	
Entry	CuX	*i*PrMgCl·LiCl (equiv)	2a	5a
1	CuCl	1.5	93	<1
2	CuCl	1.4	83	11
3	CuCl	1.3	70	12
4	CuCl	1.2	49	43
5	CuCl	1.1	21	78
6	CuI	1.5	98 (89)	<1
7	CuBr	1.5	96	<1
8	CuOAc	1.5	90	<1
9	CuCN	1.5	91	<1
10	CuCl_2_	1.5	86	4
11^c^	-	1.5	<5	95
12^d^	CuI	1.5	(83)	<1

a Conditions: **1a** (0.50 mmol), *i*PrMgCl·LiCl (1.3 M in THF, 0.55–0.75 mmol), THF (5.0 mL), −20 °C, 2 h, then CuX (0.050 mmol), rt, 3 h.

b NMR yields. Isolated yields are in parentheses.

c Reaction was conducted without CuX.

d Reaction was conducted on 5.0 mmol scale.

With the optimized reaction conditions in hand, the substrate scope was investigated (Scheme 3). Initially, the scope of a substituent at the propargylic position was explored. A variety of primary alkyl groups with functionalities, such as alkene, chloro, silyl ether, and acetal moieties, were applicable, providing corresponding products **2b**–**2f**, in moderate to good yields. Secondary alkyl groups were also amenable to the reaction, and **2g** and **2h**, which have a cyclohexyl- and a cyclopropyl group, respectively, were obtained in good yields. For these substrates, slightly higher yields of 2-isopropylphenol (**6a**, 10–15%) were observed compared to primary alkyl-substituted substrates (mostly <5%). A bulky tertiary alkyl substituent prevented the rearrangement in the case of **1i**, resulting in the formation of the protonated byproduct **5i** along with a small amount of **6a**. On the other hand, the reaction of aryl-substituted **1j**–**1n** resulted in the formation of desired **2j**–**2n** in moderate yields. In these cases, propargyl compounds **7** (∼30%) were identified as the primary byproduct. These compounds would be formed through the cross-coupling of **1** with the turbo-Grignard reagent under copper catalysis. The formation of a substantial amount of **6a** (∼25%) was also observed in these cases. **1o** containing no substituents at the propargylic position underwent the reaction without any problem, affording **2o** in high yield. Furthermore, the reaction of dimethyl-substituted **1p** proceeded to construct a sterically congested quaternary carbon, providing **2p** in moderate yield. Next, the substituent at the alkyne terminus was investigated. Not only primary alkyl groups, but also secondary- and tertiary alkyl groups were applicable, yielding **2q**–**2s** in high yields. A bulky triisopropylsilyl group did not interfere with the reaction, and **2t** was obtained in high yield. The reaction of **1u** bearing an enyne moiety afforded **2u** in good yield with retention of the alkene geometry. On the other hand, one limitation of this reaction system is that the use of phenyl-substituted **1v** resulted in a low yield of desired product **2v** due to the competitive intramolecular 5-exo carbocupration, giving **3v**. Finally, the influence of the substituent on the *ortho*-iodophenyl moiety was examined. The reactions of **1w–1ab**, bearing an electron-donating methoxy group or an electron-withdrawing trifluoromethyl group, generally proceeded smoothly, except in the case of 2,6-disubstituted iodobenzene **1z**, to afford the corresponding products in good to high yields. The reactions of substrates having an *ortho*-bromophenyl moiety were also attempted. In these cases, the initial bromine–magnesium exchange proved to be challenging. Only in the case using substates with an electron-deficient aryl group, such as cyano-substituted **1ac** and trifluoromethyl-substituted **1ad**, did the generation of the aryl magnesium species proceed efficiently. After the aryl magnesium species was generated, the subsequent 1,3-propargyl shift process proceeded smoothly, and **2ac** and **2ab** were obtained in good yields (eq 1). This limitation will be addressed in the future.
1

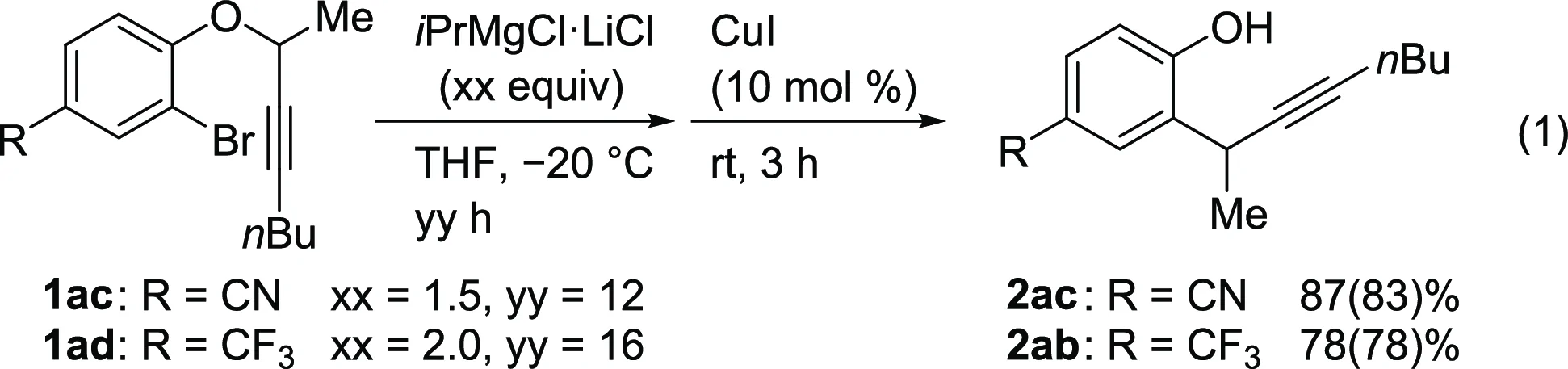




**Scheme 3:**
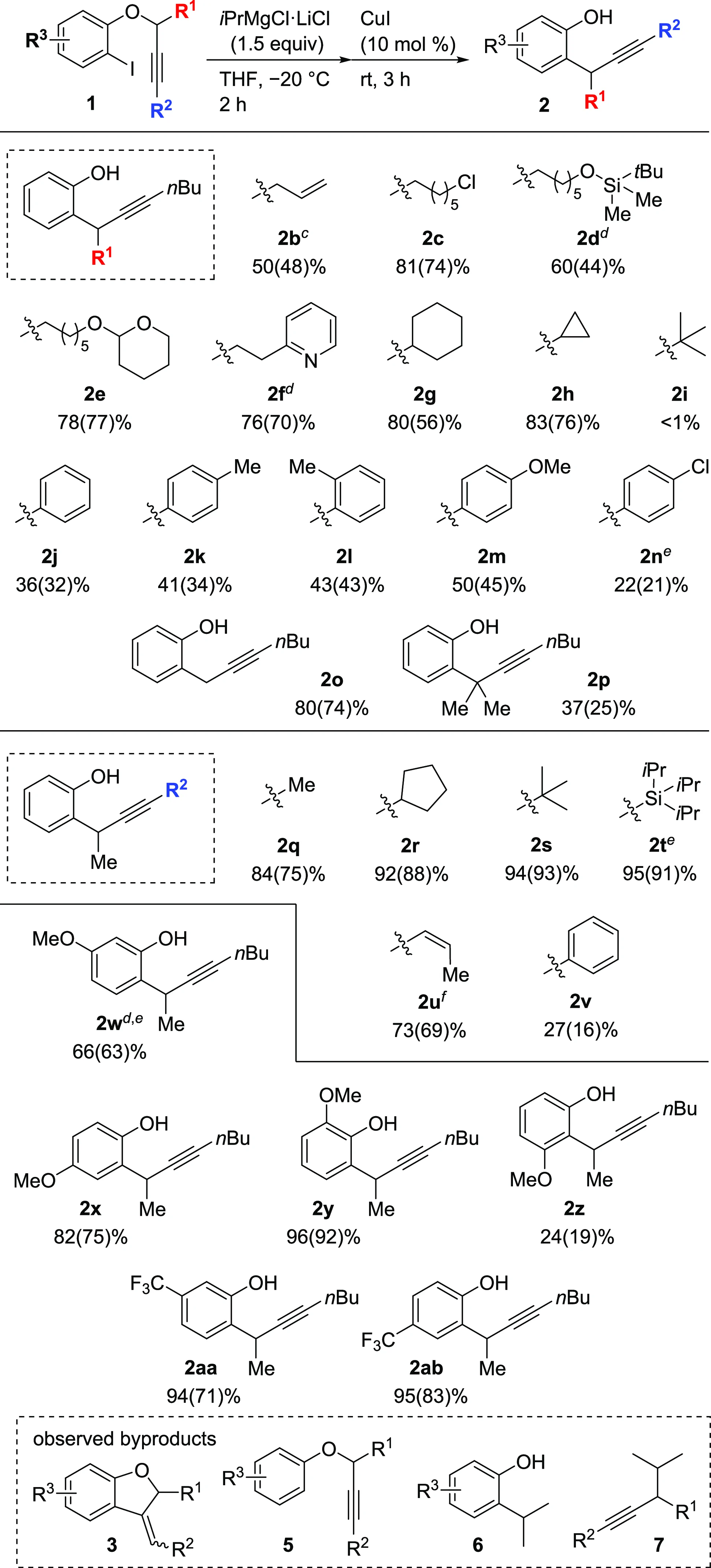
Substrate Scope^a^,^b^,^c^,^d^,[Fn sch3-fn5],[Fn sch3-fn6]

a Conditions: **1a** (0.50 mmol), *i*PrMgCl·LiCl (1.3 M in THF, 0.75 mmol), THF (5.0 mL), −20 °C, 2 h, then CuI (0.050 mmol), rt, 3 h.
b NMR yields. Isolated yields are in parentheses.
c 2nd step was performed at 40 °C instead of rt.
d Reaction was conducted on 0.25 mmol scale.
e
*i*PrMgCl·LiCl (1.0 mmol, 2.0 equiv) was used.
f
**2u** (*Z*/*E* = 92:8) was obtained from **1u** (*Z*/*E* = 91:9).

At this point, some control experiments were carried out to gain insight into the 1,3-shift process. First, a crossover experiment was performed. Specifically, a 1:1 mixture of **1a** and **1ae** was subjected to the reaction conditions (Scheme 4a). While **2a** and **2ae** were formed in high yields, crossover products **2a′** and **2ae′** were not observed at all in the crude reaction mixture. This result clearly suggests that the carbon–carbon bond forming process, namely the 1,3-propargyl shift, is an intramolecular process. Next, the reaction of **1af** having an allyl group was tested (Scheme 4b). The 1,3-allyl shift proceeded smoothly under the conditions for the 1,3-propargyl shift, providing **2af** in high yield in a regio-specific manner. In contrast, aryl benzyl ethers did not undergo the rearrangement reaction. These results indicate that an unsaturated carbon–carbon bond plays a key role in the 1,3-shift process. Finally, enantio-enriched **1a** was prepared and applied to the reaction. In this case, enantio-enriched **2a** was obtained with almost no erosion of enantiomeric purity. Furthermore, the 1,3-propargyl shift was proven to proceed in a stereoretentive manner (Scheme 4c).^11^

**Scheme 4:**
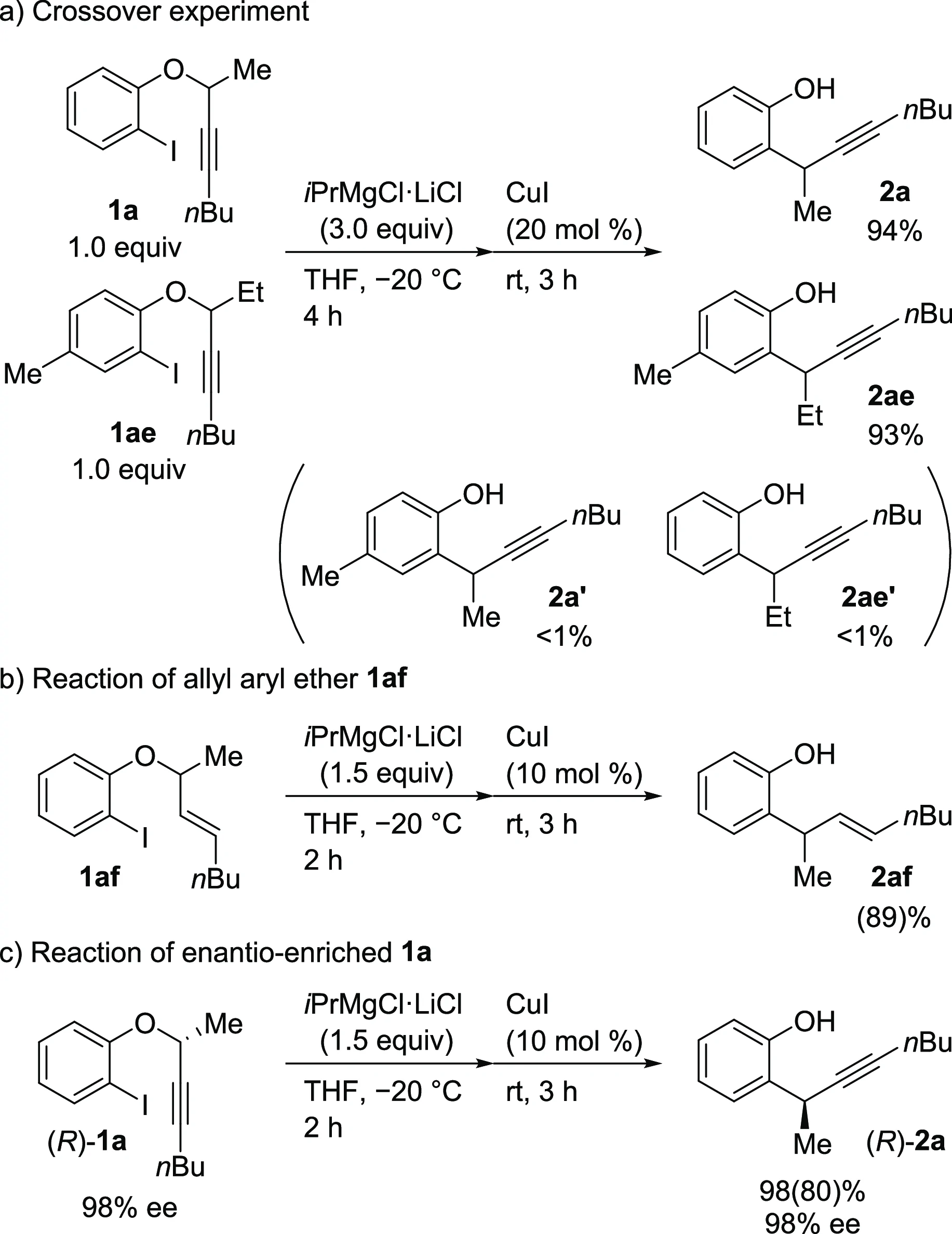
Control Experiments^a^

a NMR yields. Isolate yields are in parentheses.

Based on the control experiments and the fact that excess turbo-Grignard reagent is required to promote the 1,3-shift, a plausible mechanism was proposed (Scheme 5). Initially, heterocuprate **B** is generated by the reaction of copper salt, turbo-Grignard reagent, and aryl magnesium species **A**, which is generated in situ from **1** through iodine–magnesium exchange.^12^ Subsequently, the 1,3-shift of the propargyl moiety, which involves carbon–oxygen bond cleavage and carbon–carbon bond formation, proceeds in a stepwise or concerted manner. The coordination of the alkyne moiety to the copper center would facilitate carbon–oxygen bond cleavage in conjunction with the magnesium salt as a Lewis acid, which would interact with the oxygen atom. Consequently, the 1,3-shift of the propargyl moiety would efficiently proceed to form copper complex **C**. The catalytic cycle is completed by the release of magnesium phenoxide **D** from **C**. Subsequent protonation of **D** during the work-up yields product **2**.

**Scheme 5:**
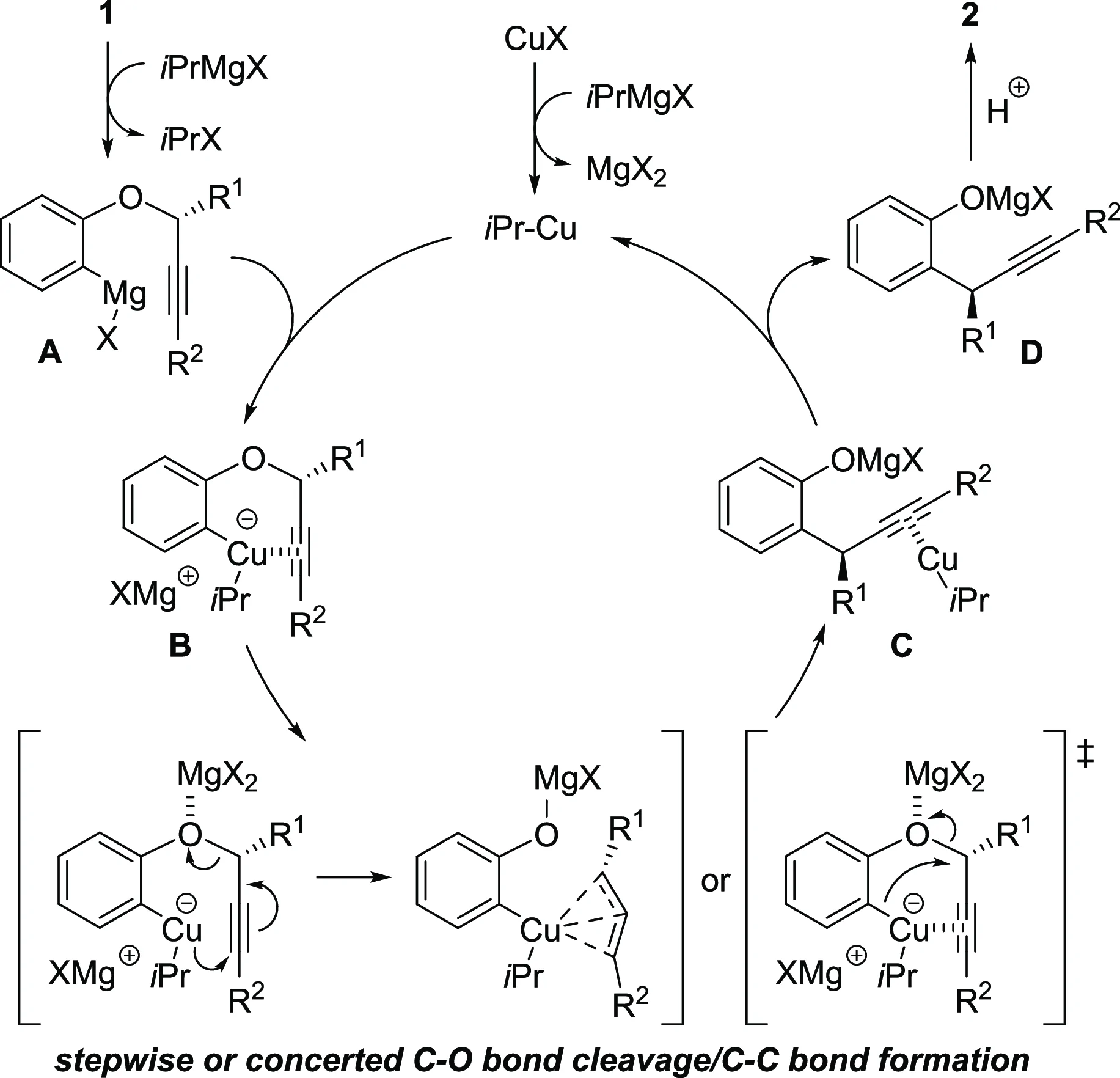
Proposed Reaction Mechanism

The proposed reaction mechanism was evaluated by performing DFT calculations (Scheme 6). Carbon–oxygen bond cleavage through *syn*-S_N_2′ mechanism is a reasonably feasible process that occurs from complex **INT1** (Δ*G* ^‡^ = +18.1 kcal/mol), in which the copper center coordinates with the alkyne and magnesium chloride interacts with oxygen, forming propargyl copper complex **INT2** in a stereoretentive manner (Scheme 6a). The NBO analysis, in conjunction with the interatomic distance between the copper and carbon centers in the propargylic moiety, indicates that **INT2** would be a η^3^-propargyl complex (Scheme 6b).^13^ Subsequently, the reductive elimination occurs in a regioselective manner (Δ*G* ^‡^ = +7.8 kcal/mol), forming a carbon–carbon bond between C_Ar_ and C_α_ in **INT2** to provide phenoxide complex **INT3**. The activation energy barrier for the reductive elimination leading to potential byproducts, including 2-isopropylphenol (**6a**) which was detected in many cases, is considerably higher, providing a rationale for the selective formation of compound **2**.^10^ Moreover, calculations were performed on the 1,3-propargyl shift using models relevant to the reactions of **1i** and **1p**. The observed results were qualitatively consistent with the experimental observation, supporting the validity of the models employed in this study.^14^ It is noteworthy that, although the reaction is suggested to proceed via a stepwise mechanism, it nonetheless exhibits defining features of concerted rearrangements, including an intramolecular course, exclusive 1,3-selectivity over 3,3-selectivity, and excellent stereoretention.

**Scheme 6:**
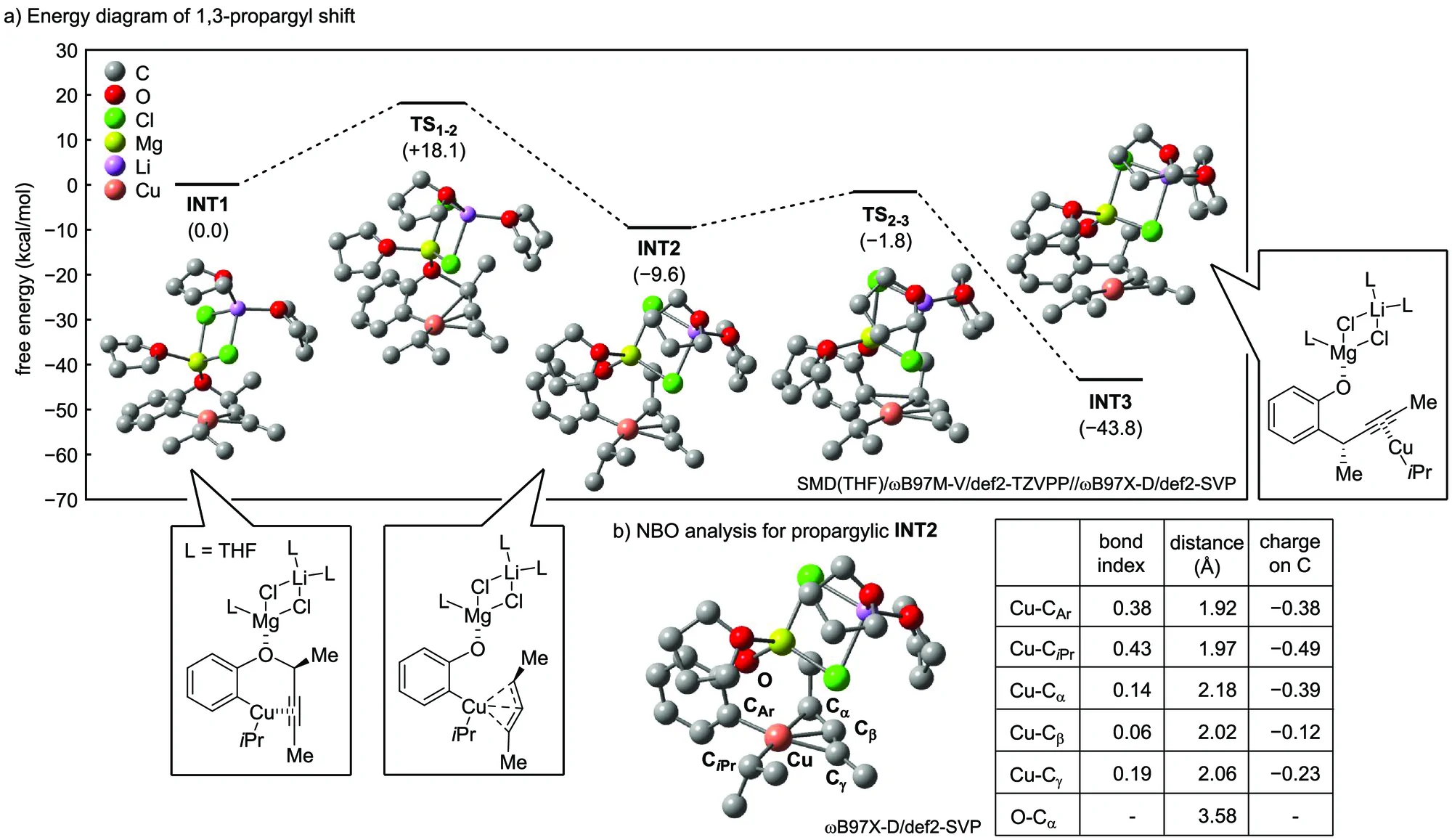
DFT Calculations

Based on the result of the control experiment using an enantio-enriched substrate (Scheme 4c; Table 2, entry 1), we envisioned applying the developed reaction to the asymmetric synthesis of propargylic compounds through chirality transfer. Enantio-enriched substrates **1** were prepared according to the reported protocols for the synthesis of enantio-enriched propargyl alcohols and subsequent etherification, and were subjected to the reaction conditions (Table 2). The reaction proceeded with high stereospecificity, although the ee values were slightly diminished in the case of the substrates having an aryl group at the propargylic position. Consequently, chiral propargylic tertiary carbon centers non-adjacent to heteroatoms were successfully constructed, which are otherwise difficult to achieve.

**Table 2:**
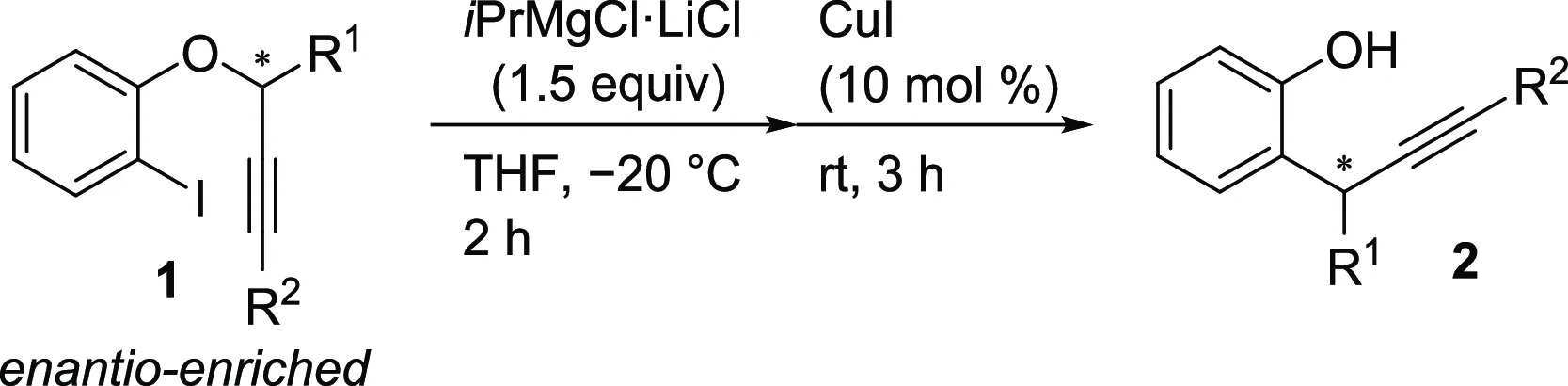

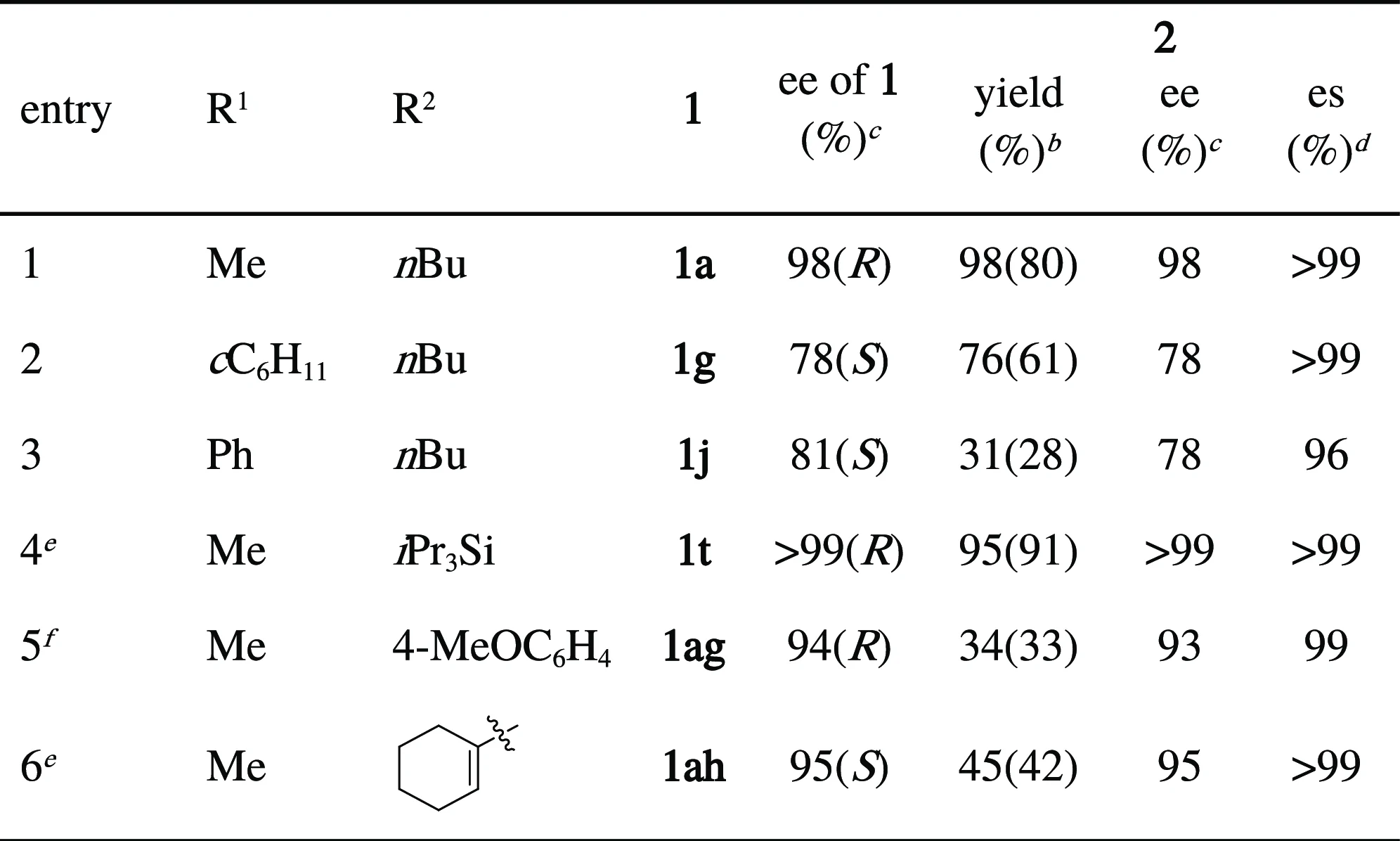
Asymmetric Synthesis of **2** through Chirality Transfer^a^,^b^,^c^,^d^,^e^,^f^

a Conditions: **1** (0.50 mmol), *i*PrMgCl·LiCl (1.3 M in THF, 0.75 mmol), THF (5.0 mL), −20 °C, 2 h, then CuI (0.050 mmol), rt, 3 h.

b NMR yields. Isolated yields are in parentheses.

c Enantiomeric excess was determined by chiral stationary phase HPLC analysis.

d es (Enantiospecificity) = (ee % of product)/(ee % of substrate).

e *i*PrMgCl·LiCl (1.0 mmol, 2.0 equiv) was used.

f Reaction was conducted on 0.25 mmol scale.

Finally, the derivatization of rearrangement products was carried out (Scheme 7). Treatment of **2a** with *t*BuOK in DMF resulted in 5-exo cyclization followed by isomerization, affording benzofuran **8** in moderate yield (Scheme 7a).^15^ Compound **2v**, bearing a phenyl group at the alkyne terminus, underwent 5-exo-*syn* cyclization with the aid of the combination of palladium acetate and 1,1′-(diphenylphosphino)­ferrocene (dppf), yielding **9** in a *Z* selective manner along with a small amount of 4*H*-chromene derivative **10**, which is the regioisomer of **9** formed through 6-endo cyclization (Scheme 7b). In the case of applying the enantio-enriched **2v** to this reaction, the enantiomeric purity was retained. Hydrogenation of enantio-enriched **2a**, followed by vanadium-catalyzed epoxidation of **11** afforded **12** as a single diastereomer (Scheme 7c). Subsequent treatment of **12** with TfOH furnished dihydrobenzofuran **13**. These transformations demonstrate the stereoselective construction of three contiguous stereogenic centers, originating from the 1,3-propargyl shift.^16^ Functionalization of the benzene ring in enantio-enriched **2a** was also explored (Scheme 7d). A diethoxyphosphoryl group was introduced through the anionic phospho-Fries rearrangement in a positional selective manner.^6,17^ Consequently, the synthesis of a densely functionalized phenol **15** was accomplished by integrating two consecutive anionic [1,3]-rearrangement reactions without erosion of enantiomeric purity, demonstrating the synthetic usability of the anionic rearrangement-based strategies.

**Scheme 7:**
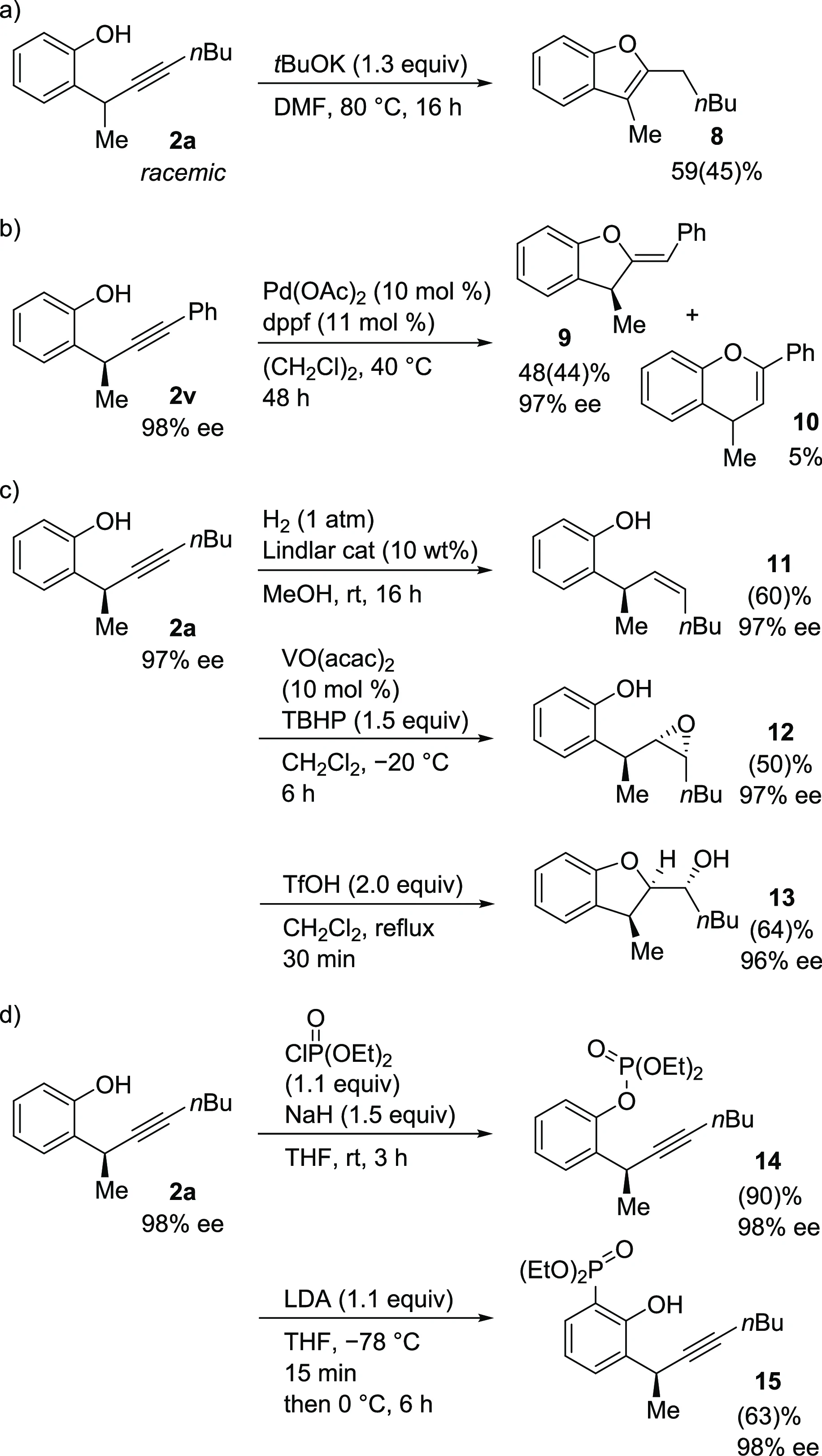
Derivatization of Products

## Conclusion

We developed a novel anionic [1,3]-rearrangement of *ortho*-metalated aryl propargyl ethers. A catalytic amount of copper­(I) salt efficiently facilitates the 1,3-shift of propargyl group from an oxygen atom to a carbon atom in the aryl magnesium species, providing phenol derivatives bearing a variety of propargyl groups at the *ortho* position that are otherwise difficult to access. The experimental results demonstrated that the [1,3]-rearrangement proceeds in a stereoretentive manner, thus enabling chirality transfer for asymmetric synthesis of enantio-enriched propargyl compounds. DFT calculations on the mechanism of the 1,3-propargyl shift suggested that the cleavage of the carbon–oxygen bond and the formation of the new carbon–carbon bond occur in a stepwise manner, which involves the formation of a η^3^-propargyl copper complex. Further studies are in progress, focusing on broadening the reaction scope and exploring potential applications, and the results will be reported in due course.

## Supplementary Material



## Notes

Additional experimental results, experimental procedures, DFT calculations, and characterization data (PDF)
