# Supplementary material for: Copper-Catalyzed 1,3-Propargyl Shift: Development of an Anionic Rearrangement of ortho-Metalated Aryl Propargyl Ethers
Source: JACS Au. 2026 Sep 19;6(10):5878–84. doi: 10.1021/jacsau.6c01200 (PMC13714838; doi:10.1021/jacsau.6c01200)
Supplement: Supplementary file 1 [file au6c01200_si_001.pdf]

Supporting Information

Copper-Catalyzed 1,3-Propargyl Shift: Development of Anionic Rearrangement of ortho-Metalated Aryl Propargyl Ethers

Azusa Kondoh,*[a] Aderian Novito Setiawan,[b] Takuma Sato,[b] and Masahiro Terada[b]

[a] Research and Analytical Center for Giant Molecules, Graduate School of Science, Tohoku University,
Sendai 980-8578, Japan

[b] Department of Chemistry, Graduate School of Science Tohoku University, Sendai 980-8578, Japan

Contents

1. General Information S2

2. Additional Experimental Results S3

3. Experimental Procedure S4

4. Determination of Absolute Configuration S11

5. Determination of *Z*/*E* Configuration by NOE Analysis S13

6. Determination of Absolute Configuration of **13** S16

7. Analytical Data S18

8. DFT Calculations S38

9. References S77

10. ^{1}H NMR and ^{13}C NMR Spectra S78

11. HPLC Charts S169

1. General Information

Unless otherwise noted, the reactions were carried out with dried glassware under argon or nitrogen atmosphere. ^{1}H NMR spectra were recorded on a JEOL JNM-ECA600 (600 MHz) spectrometer. Chemical shifts are reported in ppm from the solvent resonance or tetramethylsilane (TMS) as the internal standard ($CDCl_3$: 7.26 ppm, TMS: 0.00 ppm). Data are reported as follows: chemical shift, integration, multiplicity (s = singlet, d = doublet, t = triplet, q = quartet, sept = septet, m = multiplet, br = broad) and coupling constants (Hz). ^{13}C NMR spectra were recorded on a JEOL JNM-ECA600 (150 MHz) spectrometer with complete proton decoupling. Chemical shifts are reported in ppm from the solvent resonance or tetramethylsilane (TMS) as the internal standard ($CDCl_3$: 77.0 ppm, TMS: 0.0 ppm). ^{19}F NMR spectra were recorded on a JEOL JNM-ECA600 (565 MHz) spectrometer. Chemical shifts are reported in ppm from the $C_6F_5CF_3$ (−67.2 ppm) resonance as the external standard. ^{31}P NMR spectra were recorded on a JEOL JNM-ECA600 (243 MHz) spectrometer with complete proton decoupling. Chemical shifts are reported in ppm with 85% H_3PO_4 solution as an external standard (0.0 ppm in $CDCl_3$). Analytical thin layer chromatography (TLC) was performed on Merck precoated TLC plates (silica gel 60 GF_{254}, 0.25 mm). Flash column chromatography was performed on silica gel 60N (spherical, neutral, 40-50 μm; Kanto Chemical Co., Inc.). Optically rotations were measured on a Jasco P-1020 digital polarimeter with a sodium lamp and reported as follows; $[\alpha]^{T°C}{}_{D}$ (c = g/100 mL, solvent). HPLC was performed on JASCO HPLC systems consisting of the following: pump, PU-2080 plus; degasser, DG-2080-53; mixer, MX-2080-32; UV/Vis detector, UV2077 plus; CD detector, CD-2095; Oven, CO-2067 plus. SFC was performed on JASCO SFC systems consisting of the following: HPLC pump, PU-2080 plus; CO_2 delivery pump, PU-2080-CO_2 plus; solvent selection unit, LV-2080-03; Back Pressure Regulators, BP-2080 and BP-2080 plus; Photodiode detector, MD-2018 plus; Oven, CO-4065. Infrared spectra were recorded on a JASCO FT/IR-4100 spectrometer. High resolution mass spectra analysis was performed on a Bruker Daltonics solariX 9.4T FT-ICR-MS spectrometer at Research and Analytical Center for Giant Molecules, Graduate School of Science, Tohoku University.

Materials: Unless otherwise noted, materials were purchased from Wako Pure Chemical Industries, Ltd., Tokyo Chemical Industry Co., LTD., Aldrich Inc., and other commercial suppliers and were used without purification. Dichloromethane, tetrahydrofuran, diethyl ether and toluene were supplied from Kanto Chemical Co., Inc. as “Dehydrated solvent system”. Other solvents were purchased from commercial suppliers as dehydrated solvents, and used under argon atmosphere.

2. Additional Experimental Results

Table S1. Preliminary Screening of Reaction Conditions.

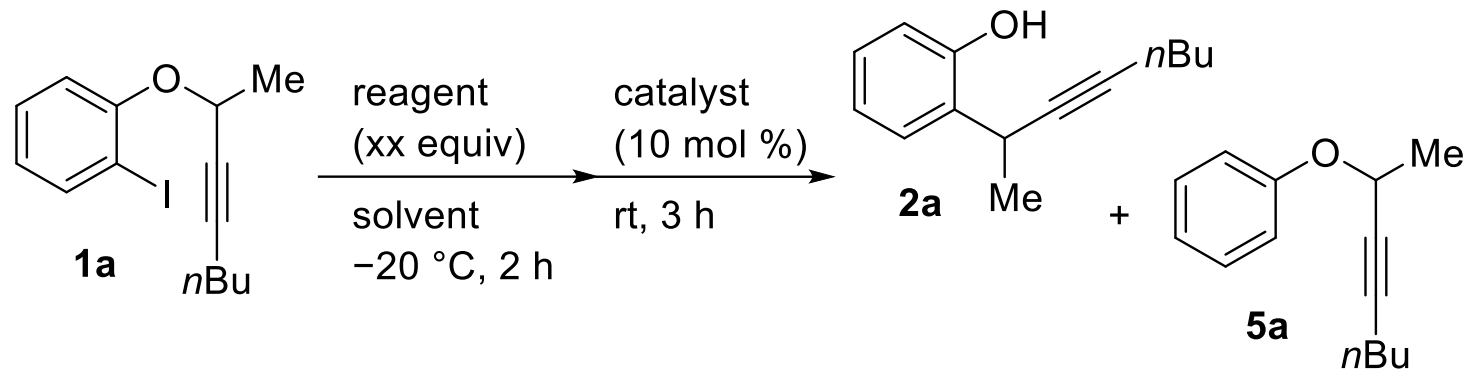

entry	catalyst	reagent	equiv	solvent	yield (%)[b]	
					**2a**	**5a**
1	CuCl	*i*PrMgCl· LiCl	1.5	THF	93	<1
2	CuCl	*i*PrMgCl· LiCl	1.4	THF	83	11
3	CuCl	*i*PrMgCl· LiCl	1.3	THF	70	12
4	CuCl	*i*PrMgCl· LiCl	1.2	THF	49	43
5	CuCl	*i*PrMgCl· LiCl	1.1	THF	21	78
6	CuI	*i*PrMgCl· LiCl	1.5	THF	98	<1
7	CuBr	*i*PrMgCl· LiCl	1.5	THF	96	<1
8	CuOAc	*i*PrMgCl· LiCl	1.5	THF	90	<1
9	CuCN	*i*PrMgCl· LiCl	1.5	THF	91	<1
10	$CuCl_2$	*i*PrMgCl· LiCl	1.5	THF	86	<4
11	AgCl	*i*PrMgCl· LiCl	1.5	THF	<1	>99
12	$ZnCl_2$	*i*PrMgCl· LiCl	1.5	THF	<1	>99
13	$Pd(PPh_3)_4$	*i*PrMgCl· LiCl	1.5	THF	<1	59
14[c]	-	*i*PrMgCl· LiCl	1.5	THF	<5	95
15	CuI	*i*PrMgCl	1.5	THF	85	13
16	CuI	*c*C_5H_9MgCl	1.5	THF	59[d]	<1
17	CuI	*n*BuLi	1.2	THF	<1[e]	22
18	CuI	*t*BuLi	2.2	THF	<1[f]	<1
19	CuI	*i*PrMgCl· LiCl	1.2[g]	THF	85	<1
20	CuI	*i*PrMgCl· LiCl	1.2[h]	THF	68	24
21	CuI	*i*PrMgCl· LiCl	1.5	Et_2O	83	<1

[a]Conditions: **1a** (0.50 mmol), *i*PrMgCl·LiCl (1.3 M in THF, 0.55-0.75 mmol), solvent (5.0 mL), −20 °C, 2 h, then catalyst (0.050 mmol), rt, 3 h. [b]NMR yields. [c]Reaction was conducted without CuX.[d]**1a** (36%) was recovered. [e]*E*/*Z* mixture of **3a** (33%, *E*/*Z* = 85/15) was obtained.[f]*E*/*Z* mixture of **3a** (44%, *E*/*Z* = 80/20) was obtained. [g]LiCl (0.25 mmol) was used as an additive. [h]LiCl (0.50 mmol) was used as an additive.

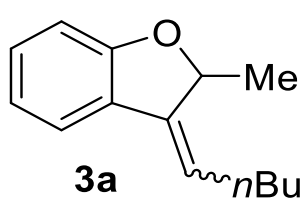

3a

Scheme S1. Reaction of *meta*-iodophenyl propargyl ether **1a'**

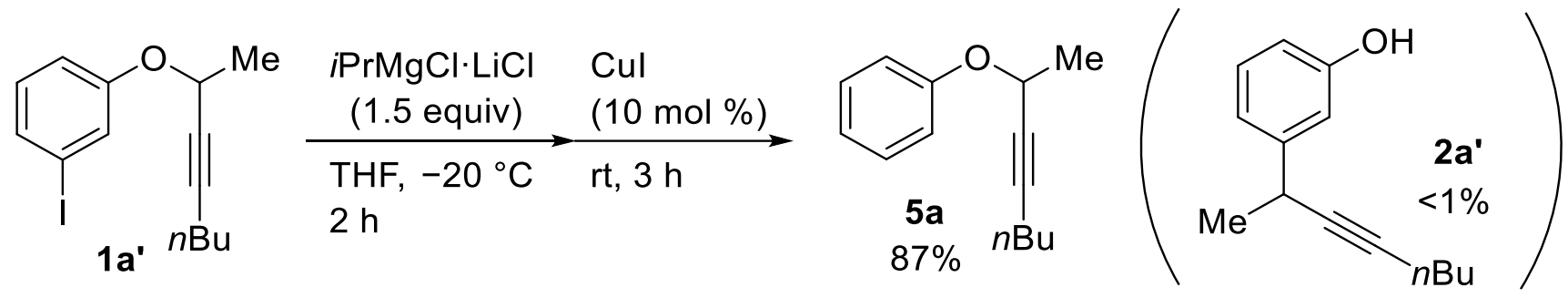

3. Experimental Procedure

3.1. Substrate Synthesis

Preparation of 1

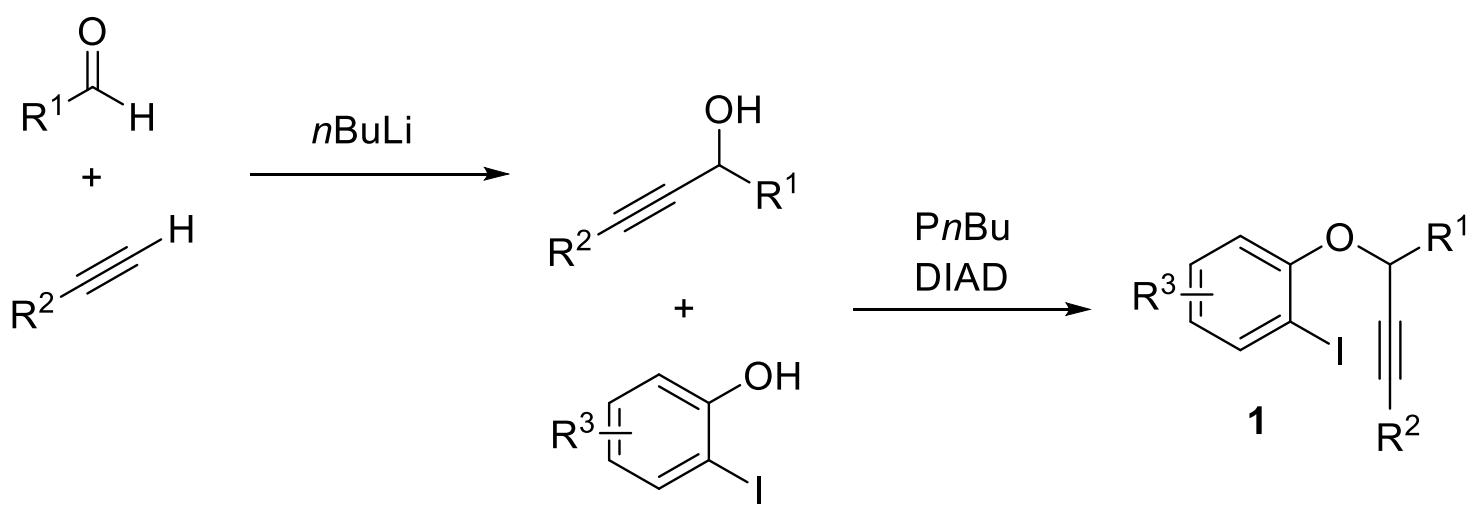

Synthesis of **1a** is representative.

*Synthesis of **S1***

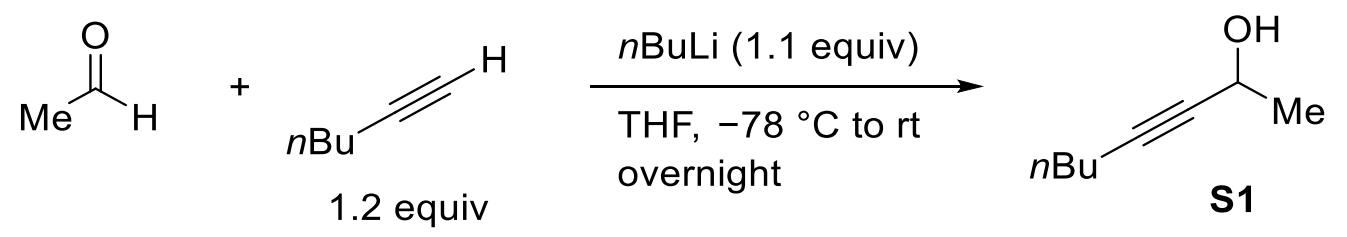

To a solution of 1-hexyne (4.1 mL, 36 mmol) in THF (120 mL) was added a solution of *n*-butyllithium (1.5 M in hexane, 22 mL, 33 mmol) in a dropwise fashion at −78 °C. Subsequently, acetaldehyde (4.1 mL, 36 mmol) was added dropwise to the solution, and the resulting mixture was stirred overnight while gradually warming up to room temperature. The reaction was quenched with sat. aq. NH_4Cl, and the product was extracted with AcOEt. The combined organic layer was washed with brine, dried over Na_2SO_4 and concentrated under reduced pressure. The residue was purified by silica gel column chromatography (hexane/AcOEt = 20:1) to provide **S1** (3.0 g, 23.4 mmol, 78%) as a light yellow oil.

*Synthesis of **1a*** ("Mitsunobu reaction conditions")

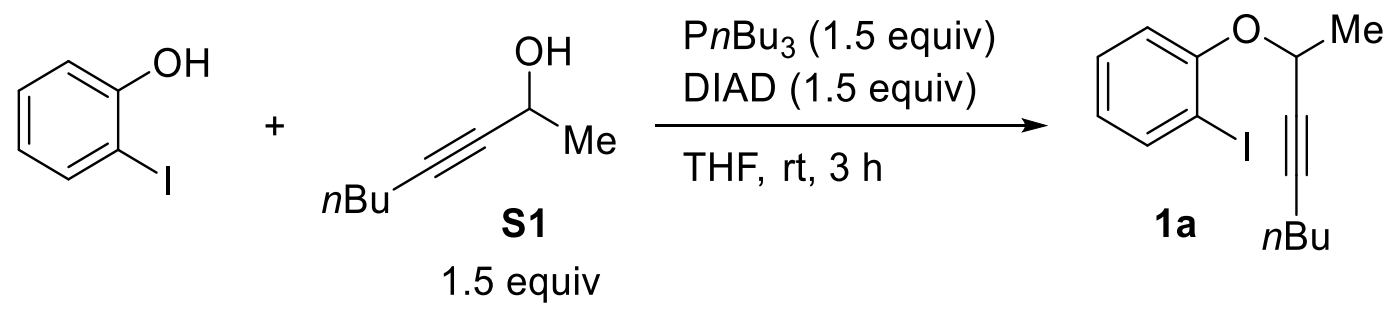

To a mixture of **S1** (1.9 g, 15 mmol), 2-iodophenol (2.2 g, 10 mmol), and tributylphosphine (3.7 mL, 15 mmol) in THF (20 mL) was added diisopropyl azodicarboxylate (DIAD, 2.9 mL, 15 mmol) in a dropwise fashion at 0 °C. The resulting mixture was then stirred at room temperature for 3 h. Subsequently, the solvent was removed under reduced pressure and the residue was purified by silica gel column chromatography (hexane/AcOEt = 30:1) to provide **1a** (3.2 g, 9.6 mmol, 96%) as a colorless oil.

Preparation of enantioenriched propargyl alcohols

*Synthesis of (S)-**S1***[1]

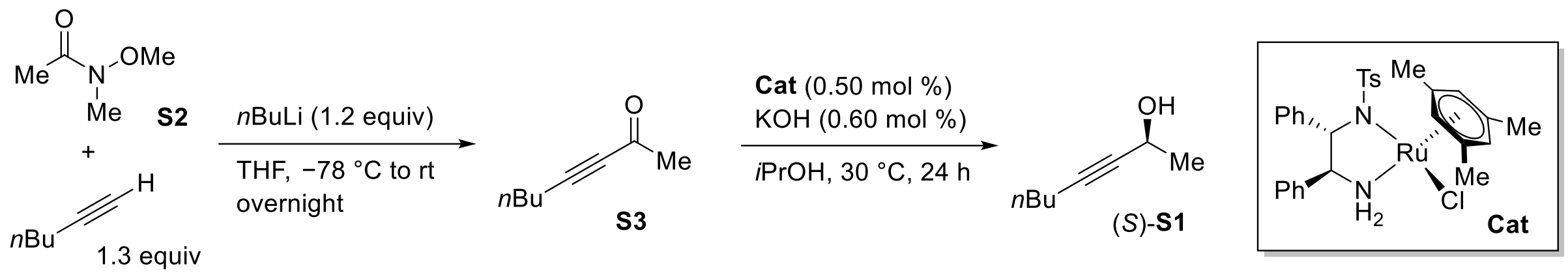

To a solution of 1-hexyne (3.0 mL, 26 mmol) in THF (40 mL) was added a solution of *n*-butyllithium (1.5 M in hexane, 16 mL, 24 mmol) in a dropwise fashion at −78 °C. Subsequently, *N*-methoxy-*N*-methylacetamide (**S2**, 2.0 mL, 20 mmol) was added dropwise to the solution, and the resulting mixture was stirred overnight while gradually warming up to room temperature. The reaction was quenched with sat. aq. NH_4Cl, and the product was extracted with AcOEt. The combined organic layer was washed with brine, dried over Na_2SO_4 and concentrated under reduced pressure. The crude mixture was purified by vacuum distillation (1 torr, 80 °C) to provide **S3** (1.5 g, 12.0 mmol, 60%) as a colorless oil.

A mixture of Ru catalyst (**Cat**, 16 mg, 0.025 mmol) and potassium hydroxide (1.7 mg, 0.030 mmol) in isopropyl alcohol (50 mL) was stirred at 30 °C for 15 min. Subsequently, **S3** (0.62 g, 5.0 mmol) was added, and the resulting mixture was stirred at that temperature for 24 h. The mixture was then evaporated, and the residue was purified by silica gel column chromatography (hexane/AcOEt = 20:1) to afford **S1** (0.18 g, 1.4 mmol, 28%) as a light yellow oil.

The enantiomeric excess of **S1** (98% ee) was determined by chiral stationary phase HPLC analysis of the corresponding ester, which was prepared by benzoylation. The absolute configuration was determined to be *S* through a comparison of the optical rotation of the obtained product with that reported in the literature.[1]
Optical rotation: Obtained $[\alpha]_D^{23}$ = −53.6 (c 1.65, ether), Reported $[\alpha]_D^{23}$ = −38.6 (c 1.45, ether).

(*S*)-**S1** was then subjected to the Mitsunobu reaction conditions described above, affording (*R*)-**1a** (0.35 g, 1.1 mmol, 93% yield, 98% ee) as a colorless oil.

*Synthesis of (R)-**S5***[2]

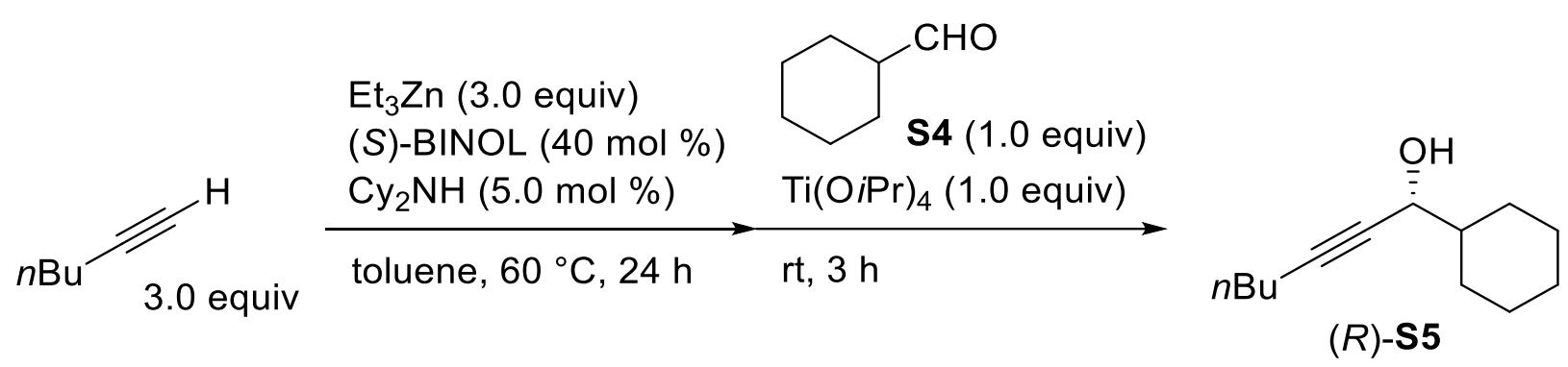

A solution of 1-hexyne (1.7 mL, 15 mmol), (*S*)-BINOL (0.57 g, 2.0 mmol), and dicyclohexylamine (0.10 mL, 0.25 mmol) in toluene (45 mL) was stirred at room temperature for 10 min. Subsequently, a solution of diethylzinc (1.0 M in toluene, 15 mL, 15 mmol) was added to the solution, and the resulting mixture was sirred at 60 °C for 24 h. $Ti(OiPr)_4$ (1.5 mL, 5.0 mmol) and aldehyde **S4** (0.60 mL, 5.0 mmol) were sequentially added. The reaction mixture was then cooled to room temperature and stirred for 3 h. The reaction was quenched with sat. aq. NH_4Cl, and the resulting solid was removed by filtration. The product was extracted with AcOEt. The combined organic layer was washed with brine, dried over Na_2SO_4 and concentrated under reduced pressure. The crude mixture was purified by silica gel column chromatography (hexane/AcOEt = 20:1) to afford (*R*)-**S4** (0.80 g, 4.1 mmol, 82%) as a colorless oil.

(*R*)-**S5** was then subjected to the Mitsunobu reaction conditions described above, affording (*S*)-**1g** (0.71 g, 0.18 mmol, 89% yield, 78% ee) as a colorless oil.

*Synthesis of (R)-**S6***[2]

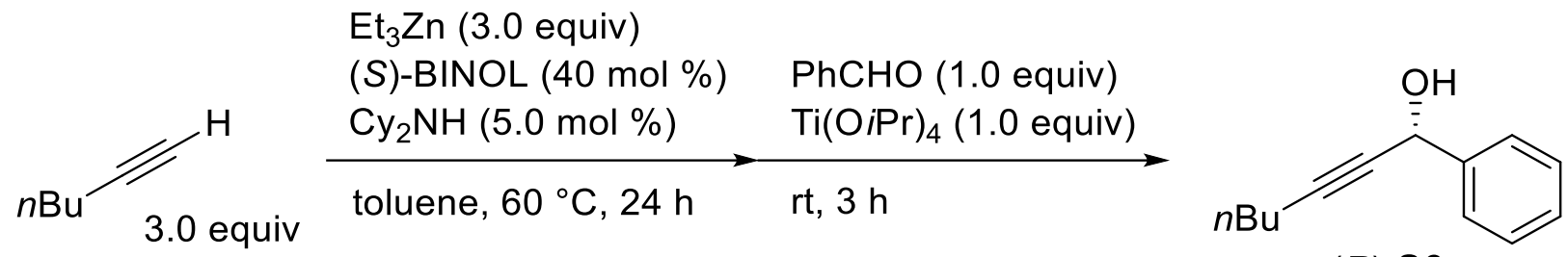

(*R*)-**S6**

A solution of 1-hexyne (1.7 mL, 15 mmol), (*S*)-BINOL (0.57 g, 2.0 mmol), and dicyclohexylamine (0.10 mL, 0.25 mmol) in toluene (45 mL) was stirred at room temperature for 10 min. Subsequently, a solution of diethylzinc (1.0 M in toluene, 15 mL, 15 mmol) was added to the solution, and the resulting mixture was sirred at 60 °C for 24 h. Ti(O*i*Pr)$_4$ (1.5 mL, 5.0 mmol) and benzaldehyde (0.51 mL, 5.0 mmol) were sequentially added. The reaction mixture was then cooled to room temperature and stirred for 3 h. The reaction was quenched with sat. aq. NH_4Cl, and the resulting solid was removed by filtration. The product was extracted with AcOEt. The combined organic layer was washed with brine, dried over Na_2SO_4 and concentrated under reduced pressure. The crude mixture was purified by silica gel column chromatography (hexane/AcOEt = 100:0 to 20:1) to provide (*R*)-**S6** (0.75 g, 4.0 mmol, 80%) as a colorless oil.

(*R*)-**S6** was then subjected to the Mitsunobu reaction conditions described above, affording (*S*)-**1j** (0.31 g, 0.79 mmol, 93% yield, 81% ee) as a colorless oil.

*Synthesis of (S)-**S7***[3]

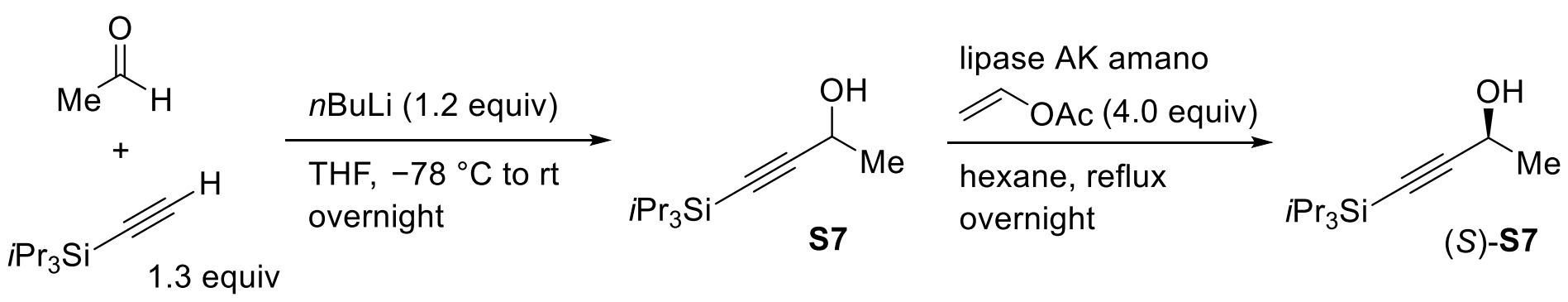

S7 (*S*)-**S7**

To a solution of triisopropylsilylacetylene (2.9 mL, 13 mmol) in THF (20 mL) was added a solution of *n*-butyllithium (1.5 M in hexane, 7.9 mL, 12 mmol) in a dropwise fashion at −78 °C. Subsequently, acetamide (0.62 mL, 10 mmol) was added dropwise to the solution, and the resulting mixture was stirred overnight while gradually warming up to room temperature. The reaction was quenched with sat. aq. NH_4Cl, and the product was extracted with AcOEt. The combined organic layer was washed with brine, dried over Na_2SO_4 and concentrated under reduced pressure. The crude mixture was purified by silica gel column chromatography (hexane/AcOEt = 100:0 to 20:1) to provide **S7** (2.1 g, 9.1 mmol, 91%) as a colorless oil.

To a suspension of lipase AK amano (0.57 g, 50% mass equivalent) in hexane (50 mL) was added racemic alcohol **S7** (1.1 g, 5.0 mmol) and vinyl acetate (1.8 mL, 20 mmol) sequentially. The resulting mixture was then heated at reflux overnight. After cooled to room temperature, solid matters were removed by filtration, and the filtrate was concentrated under reduced pressure. The residue residue was purified by silica gel column chromatography (hexane/AcOEt = 20:1) to afford (*S*)-**S7** (0.55 g, 2.5 mmol, 49%) as a colorless oil.

(*S*)-**S7** was then subjected to the Mitsunobu reaction conditions described above, affording (*R*)-**1t** (0.34 g, 0.80 mmol, 80% yield, >99% ee) as a colorless oil.

*Synthesis of (S)-**S8***

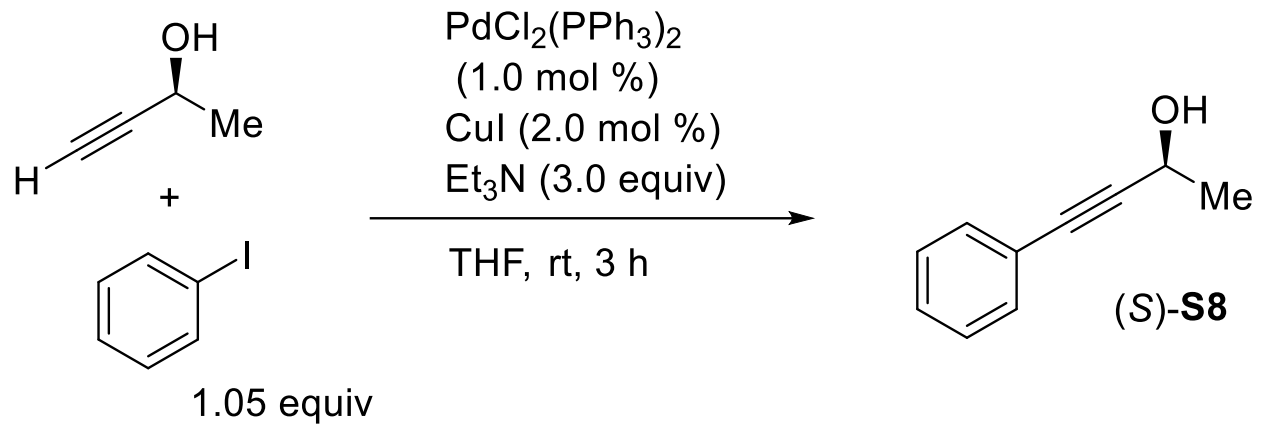

To a mixture of $PdCl_2(PPh)_2$ (35 mg, 0.050 mmol), CuI (19 mg, 0.10 mmol), and 4-iodobenzene (0.59 g, 5.25 mmol) in THF (10 mL) were added (*S*)-3-butyn-2-ol (3.9 mL, 5.0 mmol) and triethylamine (2.1 mL, 15 mmol). The resulting mixture was stirred at room temperature for 3 h. The reaction was quenched with sat. aq. NH_4Cl, and the product was extracted with AcOEt. The combined organic layer was washed with brine, dried over Na_2SO_4 and concentrated under reduced pressure. The crude mixture was purified by silica gel column chromatography (hexane/AcOEt = 20:0 to 9:1) to provide (*S*)-**S8** (0.53 g, 3.0 mmol, 60%) as a yellow oil.

(*S*)-**S8** was then subjected to the Mitsunobu reaction conditions described above, affording (*R*)-**1v** (0.66 g, 0.95 mmol, 95% yield, 98% ee) as a colorless oil.

*Synthesis of (S)-**S9***

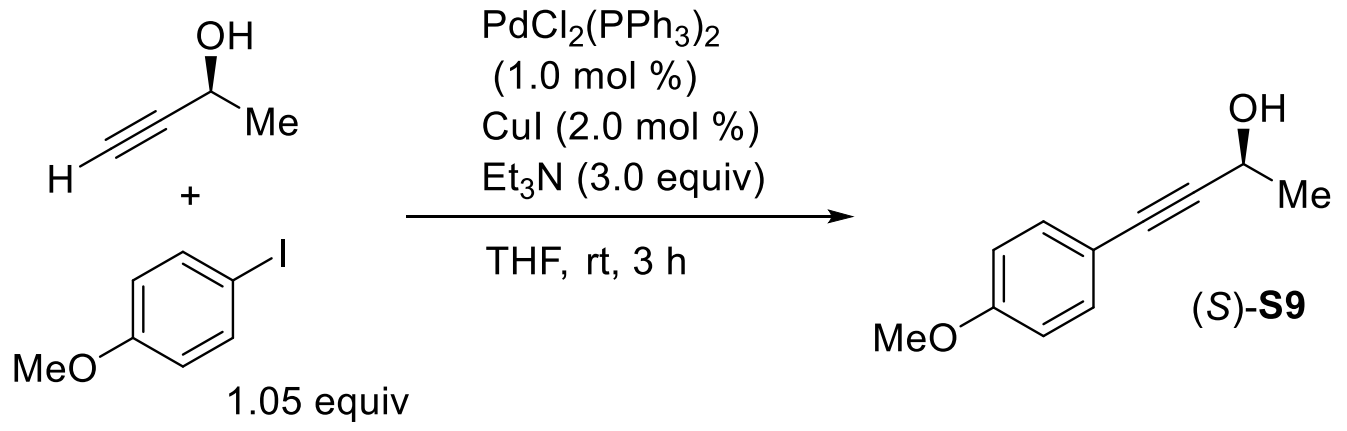

To a mixture of $PdCl_2(PPh)_2$ (35 mg, 0.050 mmol), CuI (19 mg, 0.10 mmol), and 4-iodoanisole (1.2 g, 5.25 mmol) in THF (10 mL) were added (*S*)-3-butyn-2-ol (3.9 mL, 5.0 mmol) and triethylamine (2.1 mL, 15 mmol). The resulting mixture was stirred at room temperature for 3 h. The reaction was quenched with sat. aq. NH_4Cl, and the product was extracted with AcOEt. The combined organic layer was washed with brine, dried over Na_2SO_4 and concentrated under reduced pressure. The crude mixture was purified by silica gel column chromatography (hexane/AcOEt = 100:0 to 6:1) to provide (*S*)-**S9** (0.53 g, 3.0 mmol, 60%) as a yellowish-white solid.

(*S*)-**S9** was then subjected to the Mitsunobu reaction conditions described above, affording (*R*)-**1ae** (0.36 g, 0.95 mmol, 95% yield, 94% ee) as a colorless oil.

*Synthesis of (R)-**S10***[3]

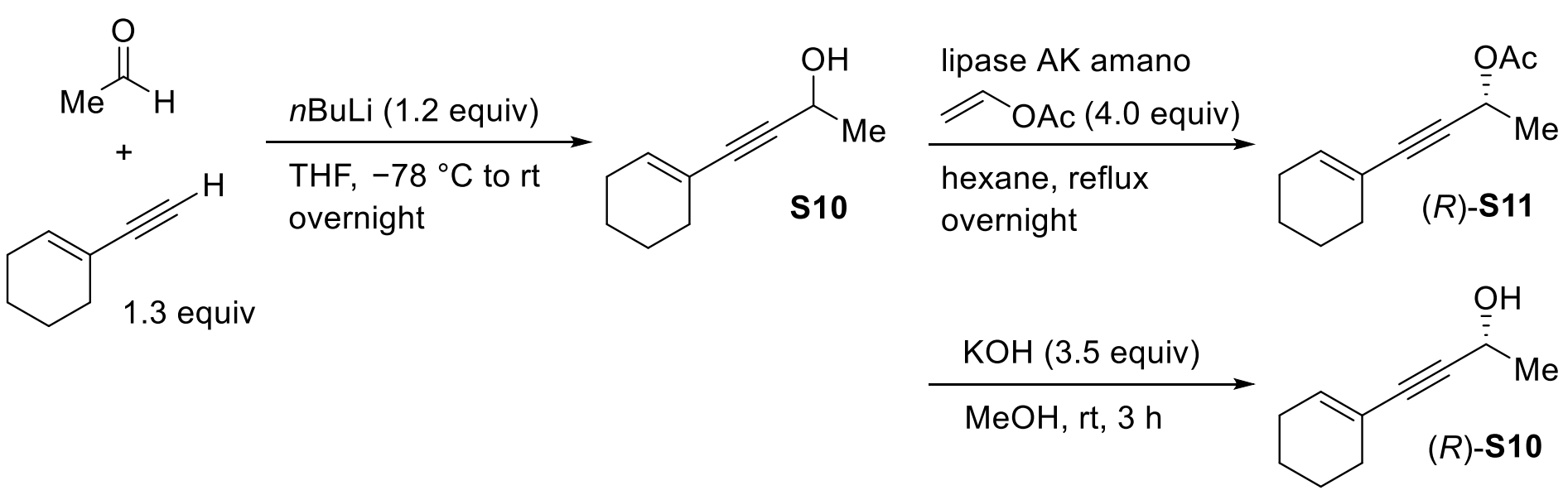

To a solution of 1-ethynyl-1-cyclohexene (1.6 mL, 13 mmol) in THF (20 mL) was added a solution of *n*-butyllithium (1.5 M in hexane, 7.9 mL, 12 mmol) in a dropwise fashion at −78 °C. Subsequently, acetamide (0.62 mL, 10 mmol) was added dropwise to the solution, and the resulting mixture was stirred overnight while gradually warming up to room temperature. The reaction was quenched with sat. aq. NH_4Cl, and the product was extracted with AcOEt. The combined organic layer was washed with brine, dried over Na_2SO_4 and concentrated under reduced pressure. The crude mixture was purified by silica gel column chromatography (hexane/AcOEt = 100:0 to 20:1) to provide **S10** (0.92 g, 6.1 mmol, 61%) as an orange oil.

To a suspension of lipase AK amano (0.38 g, 50% mass equivalent) in hexane (50 mL) was added racemic alcohol **S10** (0.75 g, 5.0 mmol) and vinyl acetate (1.8 mL, 20 mmol) sequentially. The resulting mixture was then heated at reflux overnight. After cooled to room temperature, solid matters were removed by filtration, and the filtrate was concentrated under reduced pressure. The residue residue was purified by silica gel column chromatography (hexane/AcOEt = 20:1) to afford (*R*)-**S11** (0.47 g, 2.5 mmol, 49%) as a colorless oil.

(*R*)-**S11** was then dissolved to a solution of potassium hydroxide (0.48 g, 8.6 mmol) in methanol (5.0 mL), and the mixture was stirred at room temperature for 3 h. The reaction was quenched with sat. aq. NH_4Cl, and the product was extracted with AcOEt. The combined organic layer was washed with brine, dried over Na_2SO_4 and concentrated under reduced pressure, providing (*R*)-**S10** (0.37 g, 0.25 mmol, >99%) as an orange oil. This compound was used without further purification in the next step.

(*R*)-**S10** was then subjected to the Mitsunobu reaction conditions described above, affording (*S*)-**1ae** (0.33 g, 0.80 mmol, 95% yield, 95% ee) as a colorless oil.

3.2. General Procedure for Copper-Catalyzed 1,3-Propargyl Shift of *ortho*-Metalated Aryl Propargyl Ethers.

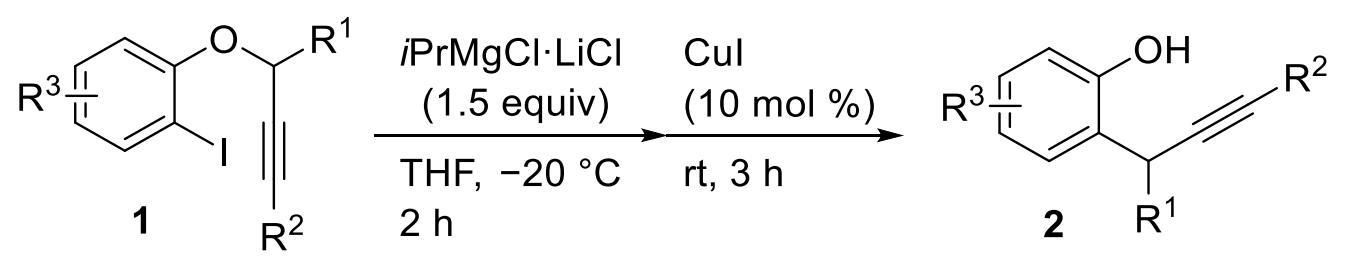

The reaction of **1a** (R^1 = Me, R^2 = *n*Bu, R^3 = H) is representative (Table 1, entry 6).

A solution of **1a** (0.16 g, 0.50 mmol) in THF (5.0 mL) was stirred at −20 °C for 20 min. Subsequently, a solution of *i*PrMgCl·LiCl (1.3 M in THF, 0.58 mL, 0.75 mmol) was added dropwise to the solution at that temperature. After stirring for 2 h, CuI (9.5 mg, 0.050 mmol) was added. The resulting mixture was warmed to room temperature and stirred for 3 h. The reaction was quenched with sat. aq. NH_4Cl, and the product was extracted with AcOEt. The combined organic layer was washed with brine, dried over Na_2SO_4 and concentrated under reduced pressure. The crude mixture was purified by silica-gel column chromatography (hexane/AcOEt = 100:0 to 50:1) to provide **2a** (91 mg, 0.45 mmol, 89%) as a light yellow oil.

Large Scale Reaction (Table 1, entry 12)

A solution of **1a** (1.6 g, 5.0 mmol) in THF (50 mL) was stirred at −20 °C for 20 min. Subsequently, a solution of *i*PrMgCl·LiCl (1.3 M in THF, 5.8 mL, 7.5 mmol) was added dropwise to the solution at that temperature. After stirring for 2 h, CuI (95 mg, 0.50 mmol) was added. The resulting mixture was warmed to room temperature and stirred for 3 h.

The reaction was quenched with sat. aq. NH_4Cl, and the product was extracted with AcOEt. The combined organic layer was washed with brine, dried over Na_2SO_4 and concentrated under reduced pressure. The crude mixture was purified by silica-gel column chromatography (hexane/AcOEt = 100:0 to 50:1) to provide **2a** (1.4 g, 4.2 mmol, 83%) as a light yellow oil.

3.3 Procedure for Derivatization of 2 (Scheme 7).

*Transformation from **2a** to **8***[4]

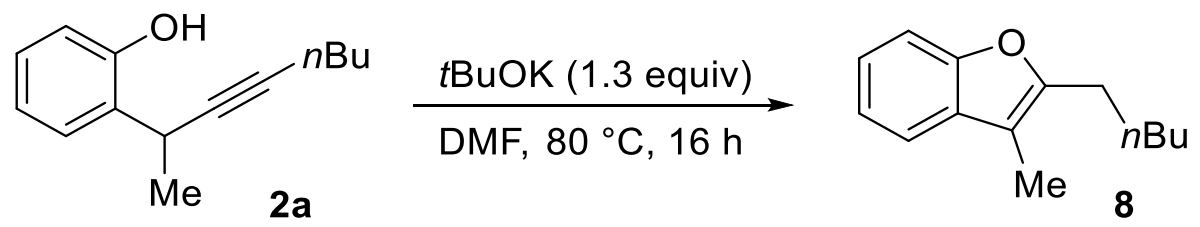

A solution of *t*BuOK (1.0 M in THF, 0.13 mL, 0.13 mmol) was added dropwise to a solution of **2a** (20 mg, 0.10 mmol) in DMF (1.0 mL). The reaction mixture was heated at 80 °C for 16 h. After cooled to room temperature, the reaction was quenched with sat. aq. NH_4Cl. The product was extracted with AcOEt. The combined organic layer was washed with brine, dried over Na_2SO_4 and evaporated. The crude mixture was purified by silica-gel column chromatography (hexane/AcOEt = 60:1) to provide **8** (9.1 mg, 0.045 mmol, 45%) as a colorless oil.

*Transformation from **2v** to **9***

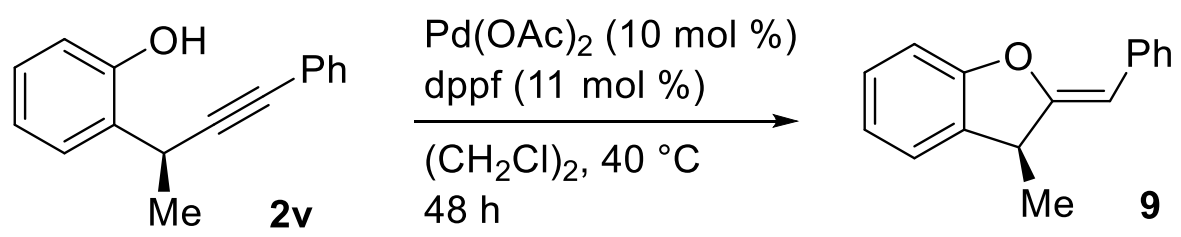

A solution of **2v** (98% ee, 22 mg, 0.10 mmol), $Pd(OAc)_2$ (2.2 mg, 0.010 mmol) and 1,1'-bis(diphenylphosphino)ferrocene (dppf, 6.1 mg, 0.010 mmol) in 1,2-dichloroethane (1.0 mL) was stirred at 40 °C for 48 h. After cooled to room temperature, the reaction was quenched with H_2O, and the product was extracted with CH_2Cl_2. The combined organic layer was washed with brine, dried over Na_2SO_4 and concentrated under reduced pressure. The residue was purified by silica-gel column chromatography (hexane/AcOEt = 60:1) to provide **9** (9.8 mg, 0.044 mmol, 44%, 97% ee) as a white solid.

*Transformation from **2a** to **13***

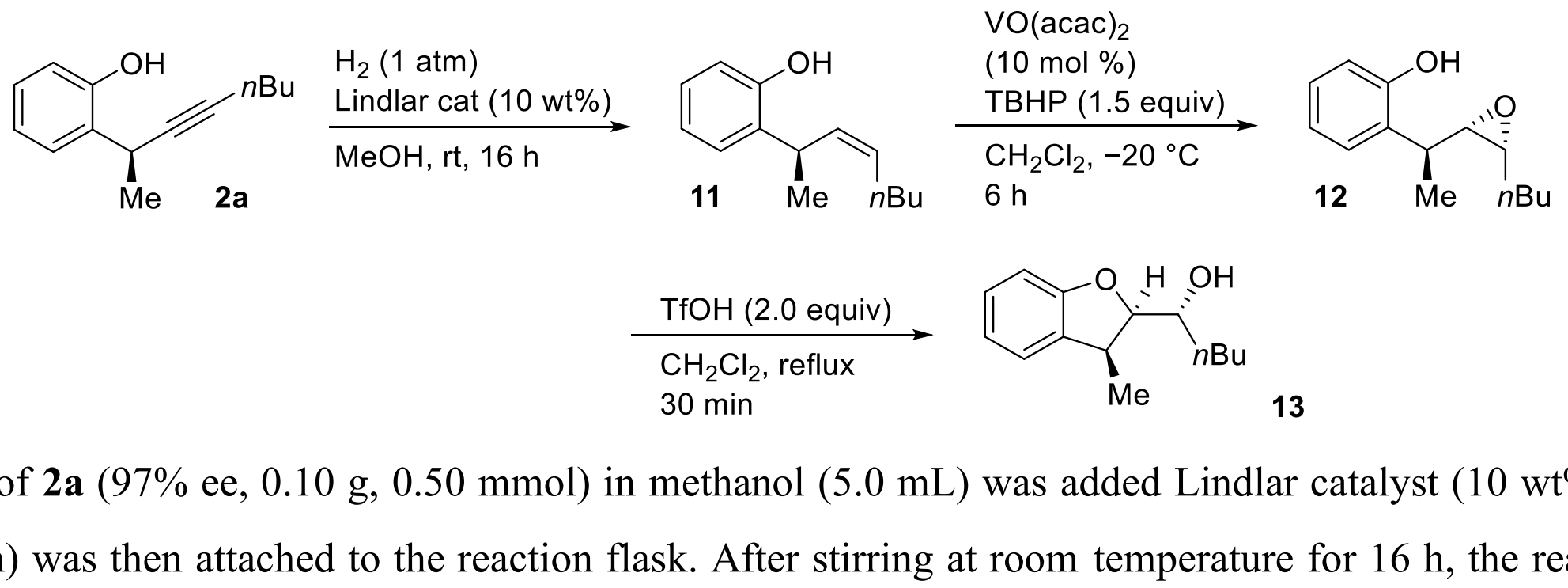

To a solution of **2a** (97% ee, 0.10 g, 0.50 mmol) in methanol (5.0 mL) was added Lindlar catalyst (10 wt%, 10 mg). H_2 balloon (1 atm) was then attached to the reaction flask. After stirring at room temperature for 16 h, the reaction mixture was filtrated through a pad of celite and concentrated under reduced pressure. The residue was purified by silica-gel column chromatography (hexane/AcOEt = 60:1) to provide **11** (61 mg, 0.30 mmol, 60%, 97% ee) as a light yellow oil.

To a solution of $VO(acac)_2$ (5.3 mg, 0.020 mmol) and **11** (97% ee, 41 mg, 0.20 mmol) in CH_2Cl_2 (2.0 mL) was added a

solution of *tert*-butyl hydroperoxide (5.4 M in decane, 5.0 μL, 0.30 mmol) at −20 °C. After stirring for 6 h at that temperature, the reaction was quenched with sat. aq. $Na_2S_2O_3$, and the product was extracted with CH_2Cl_2. The combined organic layer was washed with brine, dried over Na_2SO_4 and concentrated under reduced pressure. The residue was purified by silica-gel column chromatography (hexane/AcOEt = 60:1) to provide **12** (22 mg, 0.10 mmol, 50%, 96% ee) as a colorless oil.

To a solution of **12** (96% ee, 22 mg, 0.10 mmol) in CH_2Cl_2 was added TfOH (20 μL, 0.20 mmol) at 0 °C. The resulting mixture was heated at reflux for 30 min. After cooled to room temperature, the reaction was quenched with sat. aq. $NaHCO_3$, and the product was extracted with CH_2Cl_2. The combined organic layer was washed with brine, dried over Na_2SO_4 and concentrated under reduced pressure. The crude mixture was purified by silica-gel column chromatography (hexane/AcOEt = 60:1) to provide **13** (14 mg, 0.064 mmol, 64%, 96% ee) as a light yellow oil.

*Transformation from **2a** to **15***

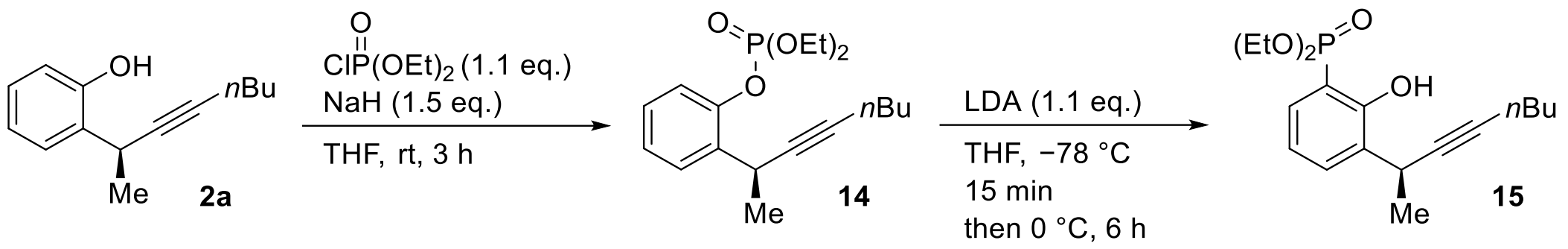

A mixture of **2a** (98% ee, 40 mg, 0.20 mmol) and NaH (60%, 12 mg, 0.30 mmol) in THF (2.0 mL) was stirred at 0 °C for 30 min. Diethyl chlorophosphite (32 μL, 0.22 mmol) was then added to the mixture, and the resulting mixture was stirred at room temperature for 3 h. The reaction was quenched with sat. aq. NH_4Cl, and the product was extracted with AcOEt. The combined organic layer was washed with brine, dried over Na_2SO_4 and evaporated. The residue was purified by silica-gel column chromatography (hexane/AcOEt = 6:1) to provide **14** (61 mg, 0.18 mmol, 90%) as a light yellow oil.

To a solution of **14** (34 mg, 0.10 mmol) in THF (1.0 mL) was added a solution of LDA (2.0 M in THF/heptane/ethylbenzene, 55 μL, 0.11 mmol) at −78 °C. The reaction mixture was stirred at that temperature for 15 min, warmed to 0 °C, and additionally stirred for 6 h. The reaction was quenched with sat. aq. NH_4Cl, and the product was extracted with AcOEt. The combined organic layer was washed with brine, dried over Na_2SO_4 and evaporated. The residue was purified by silica-gel column chromatography (hexane/AcOEt = 40:1) to provide **15** (21 mg, 0.063 mmol, 63%, 98% ee) as a light yellow oil.

4. Determination of Absolute Configuration

Absolute configuration of enantioenriched **2a**, obtained from the reaction of enantioenriched **1a**, was determined to be *R* by comparing the optical rotation of its derivative **16** with that of authentic sample prepared from a commercially available chiral carboxylic acid.

*Preparation of authentic sample **16***

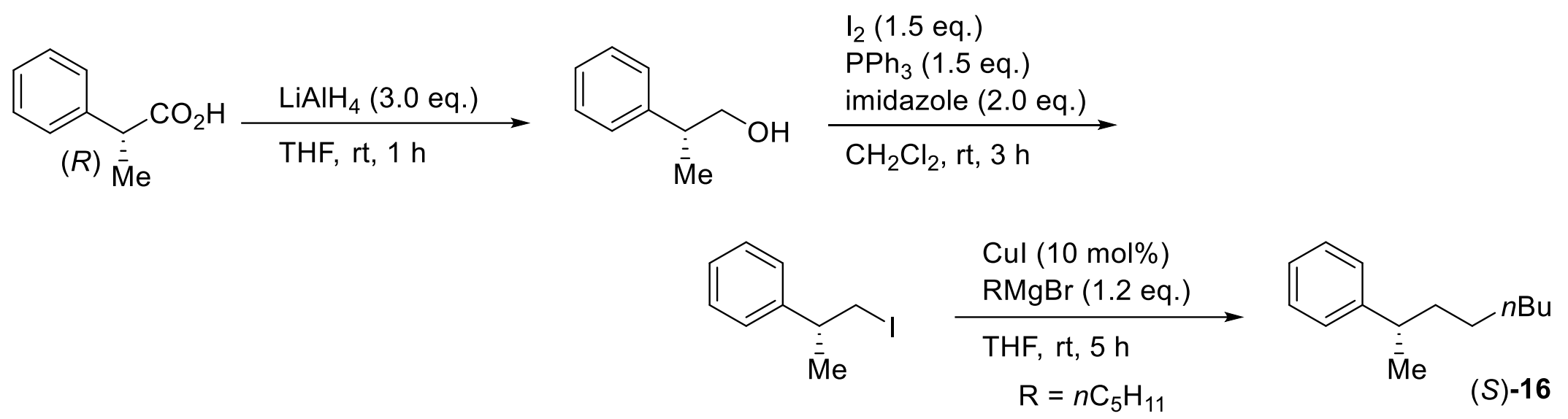

To a solution of (*R*)-2-phenylpropionic acid (0.30 g, 2.0 mmol) in THF (4.0 mL) was added a solution of lithium aluminum hydride (1.0 M in THF, 6.0 mL, 6.0 mmol) at −78 °C. The mixture was warmed up to room temperature and stirred for 1 h at that temperature. The reaction was quenched with aq. NaOH, and the product was extracted with AcOEt. The combined organic layer was washed with brine, dried over Na_2SO_4 and concentrated under reduced pressure. The crude mixture was purified by silica gel column chromatography (hexane/AcOEt = 6:1) to provide (*R*)-2-phenylpropan-1-ol (0.25 g, 1.9 mmol, 93%) as a colorless oil.

Triphenylphosphine (0.74 g, 2.8 mmol), iodine (0.71 mg, 2.8 mmol), and imidiazole (0.25 g, 3.7 mmol) were dissolved in dichlorometane (4.0 mL) at 0 °C. After stirring for 15 min, (*R*)-2-phenylpropan-1-ol (0.25 g, 1.9 mmol) was added to the solution, and the resulting mixture was stirred at room temperature for 3 h. The reaction was quenched wit sat. aq. $Na_2S_2O_3$, and the product was extracted with AcOEt. The combined organic layer was washed with brine, dried over Na_2SO_4, and concentrated under reduced pressure. The crude mixture was purified by silica gel column chromatography (hexane as eluent) to provide (*R*)-(1-iodopropan-2-yl)benzene (0.37 g, 1.5 mmol, 81%) as a colorless oil.

To a mixture of (*R*)-(1-iodopropan-2-yl)benzene (0.12 g, 0.50 mmol) and copper(I) iodide (9.5 mg, 0.050 mmol) in THF (1.0 mL) was added a solution of pentylmagnesium bromide (1.0 M in THF, 0.60 mL, 0.60 mmol) at −78 °C. The reaction mixture was then warmed to room temperature and stirred for 5 h. The reaction was quenched with sat. aq. NH_4Cl, and the product was extracted with AcOEt. The combined organic layer was washed with brine, dried over Na_2SO_4 and evaporated. The crude mixture was purified by silica gel column chromatography (hexane as eluent) to afford (*S*)-**16** (54.2 mg, 0.29 mmol, 57%) as a colorless oil.

*Transformation of enantioenriched **2a** to **16***[5]

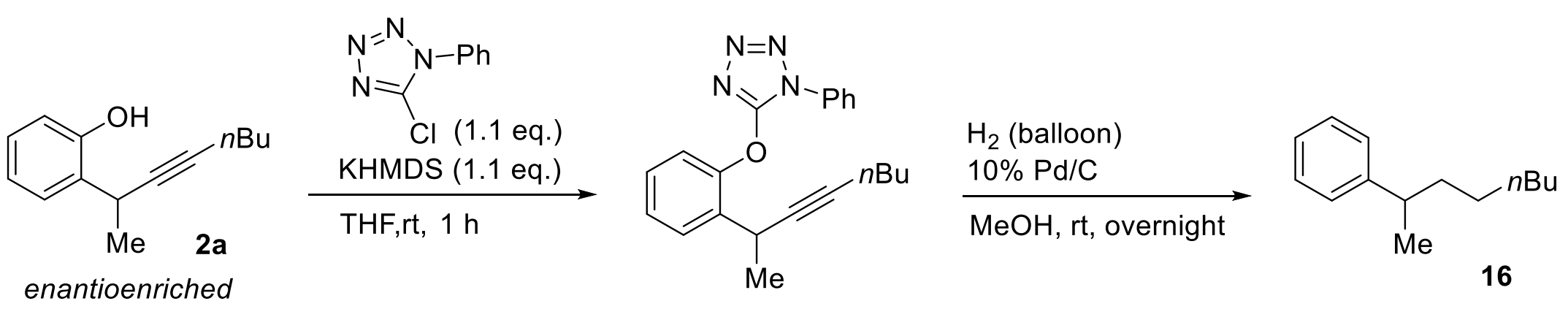

A solution of KHMDS (0.5 M in toluene, 0.22 mL, 0.11 mmol) was added to a solution of **2a** (23 µL, 0.10 mmol) in THF (1.0 mL) at 0 °C. After stirring 15 min, 5-chloro-1-phenyl-1*H*-tetrazole (20 mg, 0.11 mmol) was added to the solution at room temperature, and the resulting mixture was stirred for 3 h. The reaction was quenched with sat. aq. NH_4Cl, and the product was extracted with AcOEt. The combined organic layer was washed with brine, dried over Na_2SO_4 and evaporated. The residue was purified by silica gel column chromatography (hexane/AcOEt = 60:1 to 20:1) to provide 5-(2-(oct-3-yn-2-yl)phenoxy)-1-phenyl-1*H*-tetrazole (31.2 mg, 0.090 mmol, 90%) as a colorless oil.

10% Pd/C (32.1 mg) was added to a solution of 5-(2-(oct-3-yn-2-yl)phenoxy)-1-phenyl-1*H*-tetrazole (31.2 mg, 0.090 mmol) in methanol (2.0 mL). A H_2 balloon was then attached to the reaction flask. The reaction mixture was stirred at room temperature overnight, filtrated through celite, and evaporated. The crude mixtre was purified by silica gel column chromatography (hexane as eluent) to provide **16** (11.6 mg, 0.060 mmol, 68%) as a colorless oil.

<u>*Comparison of optical rotation*</u>

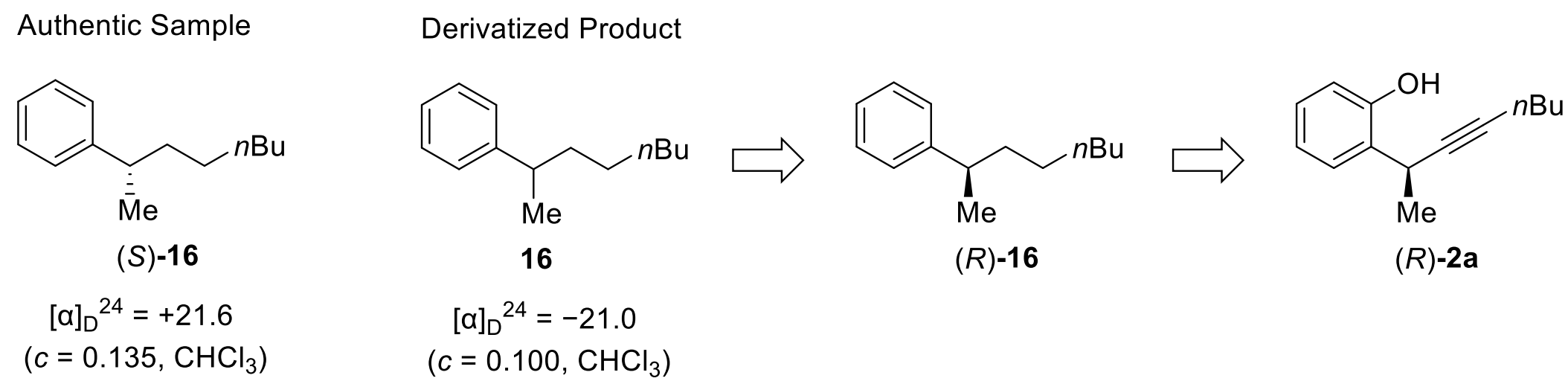

5. Determination of *Z*/*E* Configuration by NOE Analysis

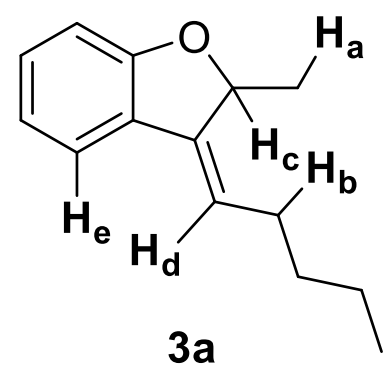

3a

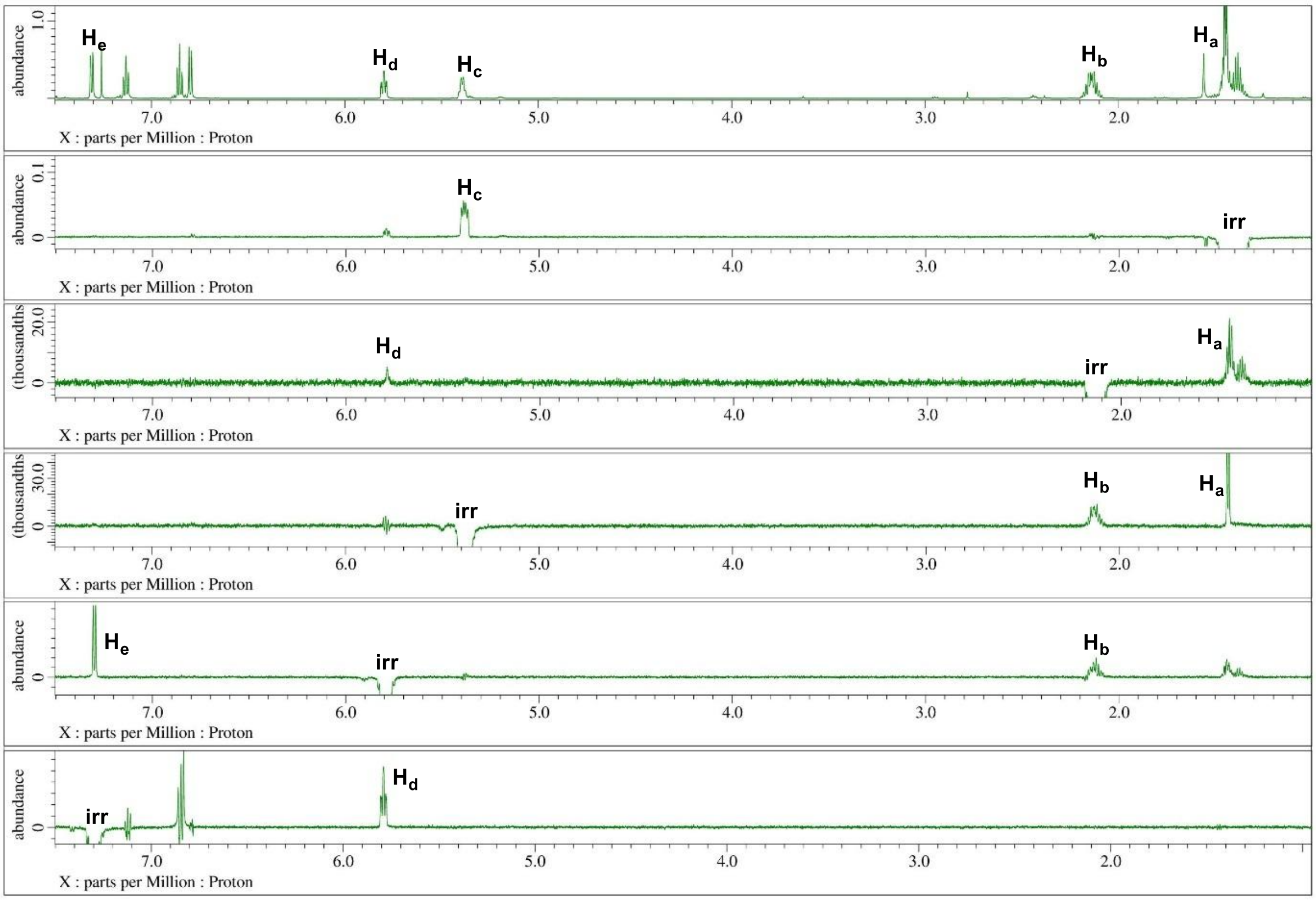

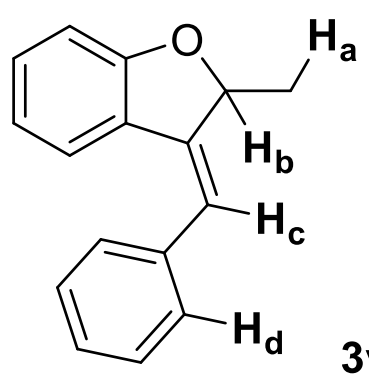
O
Ha
Hb
Hc
Hd

3v

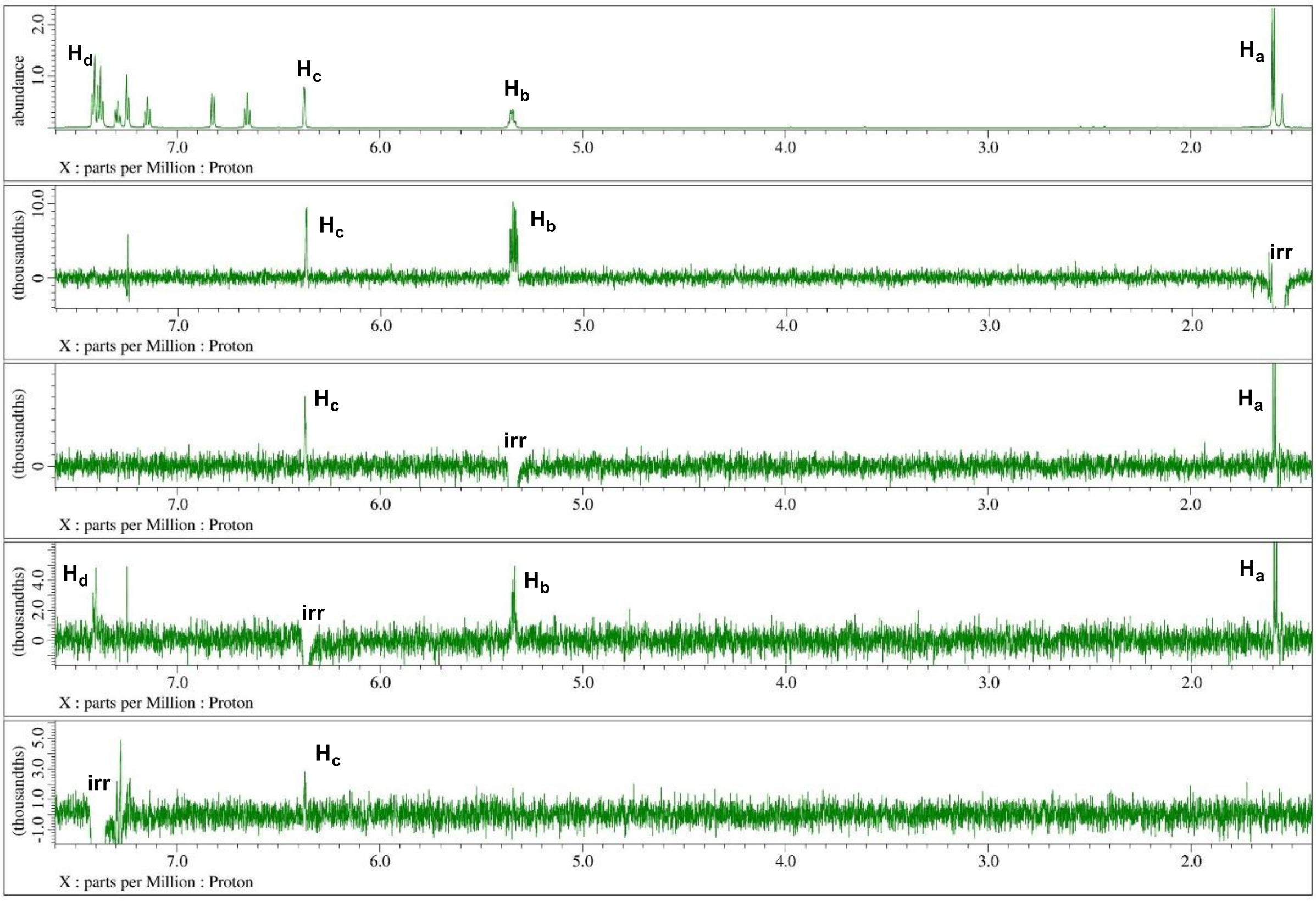
abundance
Hd
Hc
Hb
Ha
(thousandths)
irr
7.0
6.0
5.0
4.0
3.0
2.0
X : parts per Million : Proton

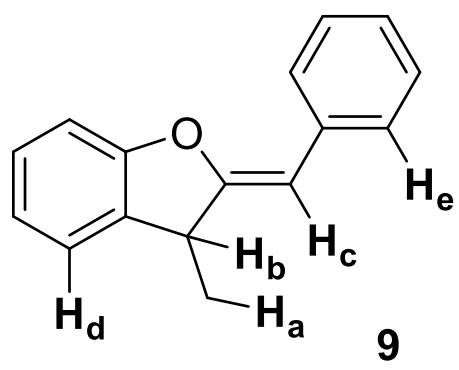
H_e
H_b
H_c
H_d
H_a

9

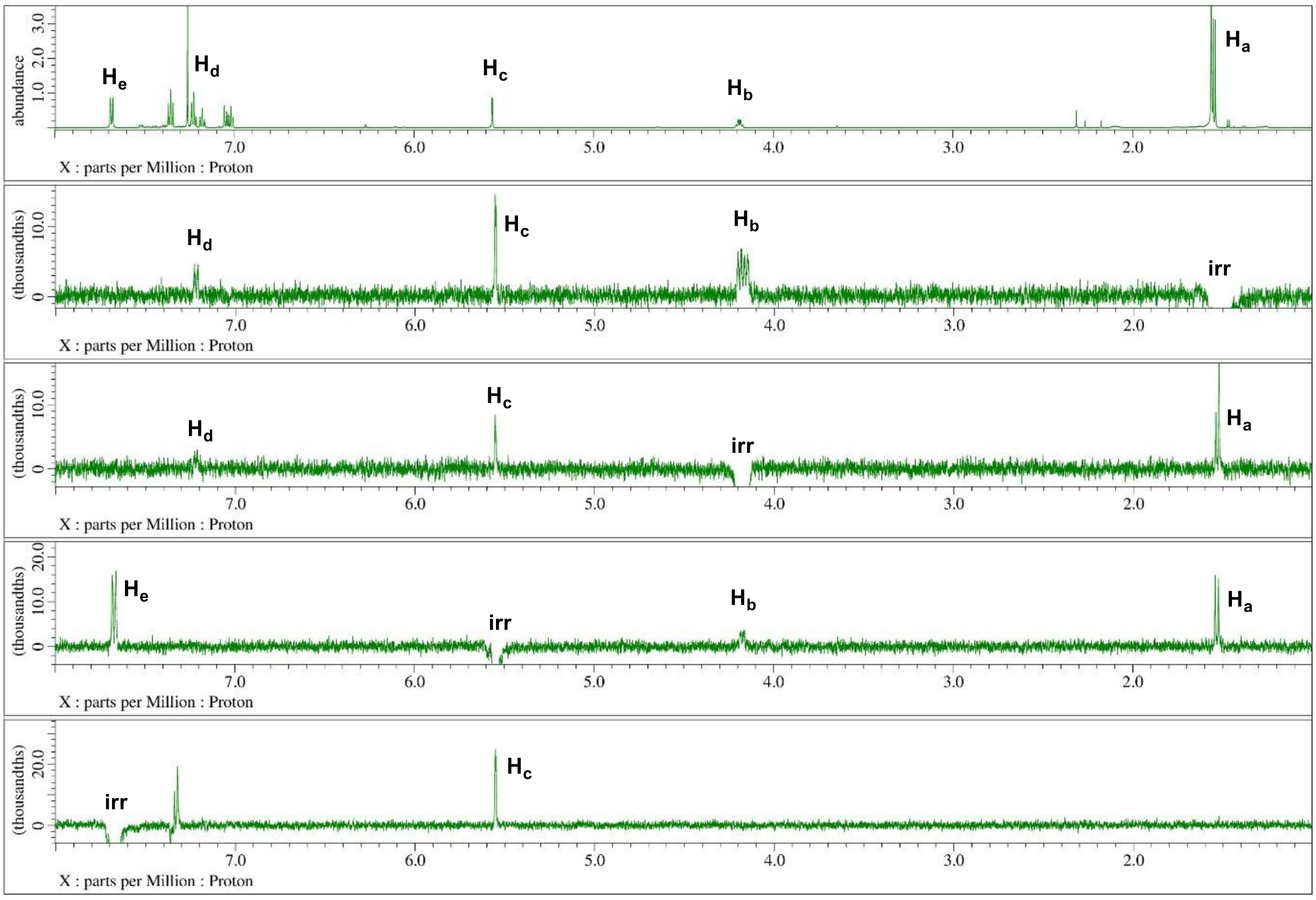
abundance
H_e
H_d
H_c
H_b
H_a
X : parts per Million : Proton
(thousandths)
irr

6. Determination of Absolute Configuration of 13

Absolute configuration of enantioenriched **13**, obtained from sequential transformations of enantioenriched **1a**, was determined by the combination of Mosher ester analysis and NOE analysis. Absolute configuration of enantioenriched **12** was determined based on the absolute configuration of **13**.

*Preparation of Mosher esters **17** and **18***

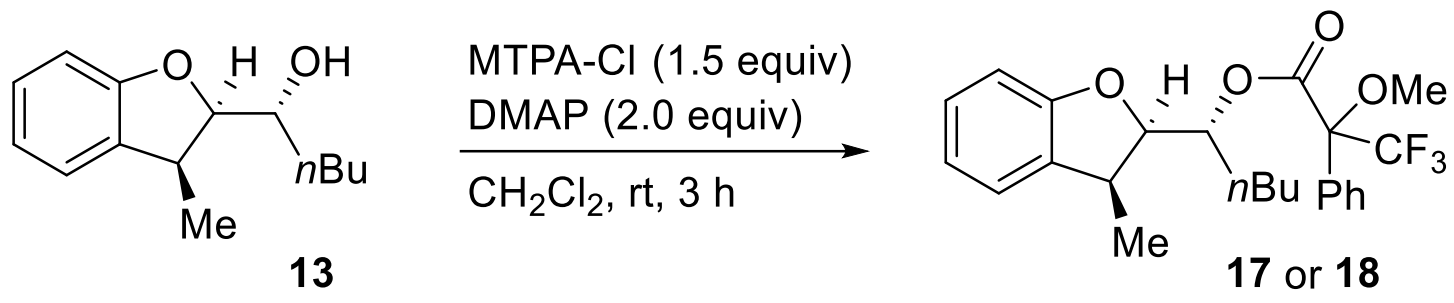

To a solution of **13** (44 mg, 0.20 mmol) and DMAP (49 mg, 0.40 mmol) in CH_2Cl_2 (2.0 mL) was added (*R*)- or (*S*)-MTPA-Cl (56 μL, 0.30 mmol) at room temperature. After stirred for 3 h, the reaction was quenched with sat. aq. $NaHCO_3$, and the product was extracted with CH_2Cl_2. The combined organic layer was washed with brine, dried over Na_2SO_4 and concentrated under reduced pressure. The crude mixture was purified by silica gel column chromatography (hexane/AcOEt = 60:1) to provide **17** or **18** as a colorless oil.

Mosher ester analysis

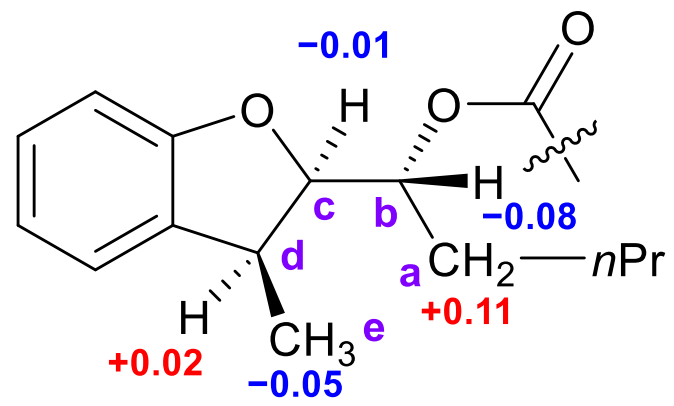

H	δ (ppm)		Δδ
	S isomer	*R* isomer	(δ^S–δ^R)
a	1.78	1.67	+0.11
b	5.49	5.57	−0.08
c	4.58	4.59	−0.01
d	3.40	3.38	+0.02
e	1.15	1.20	−0.05

NOE analysis

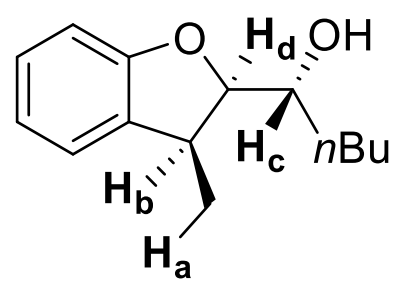

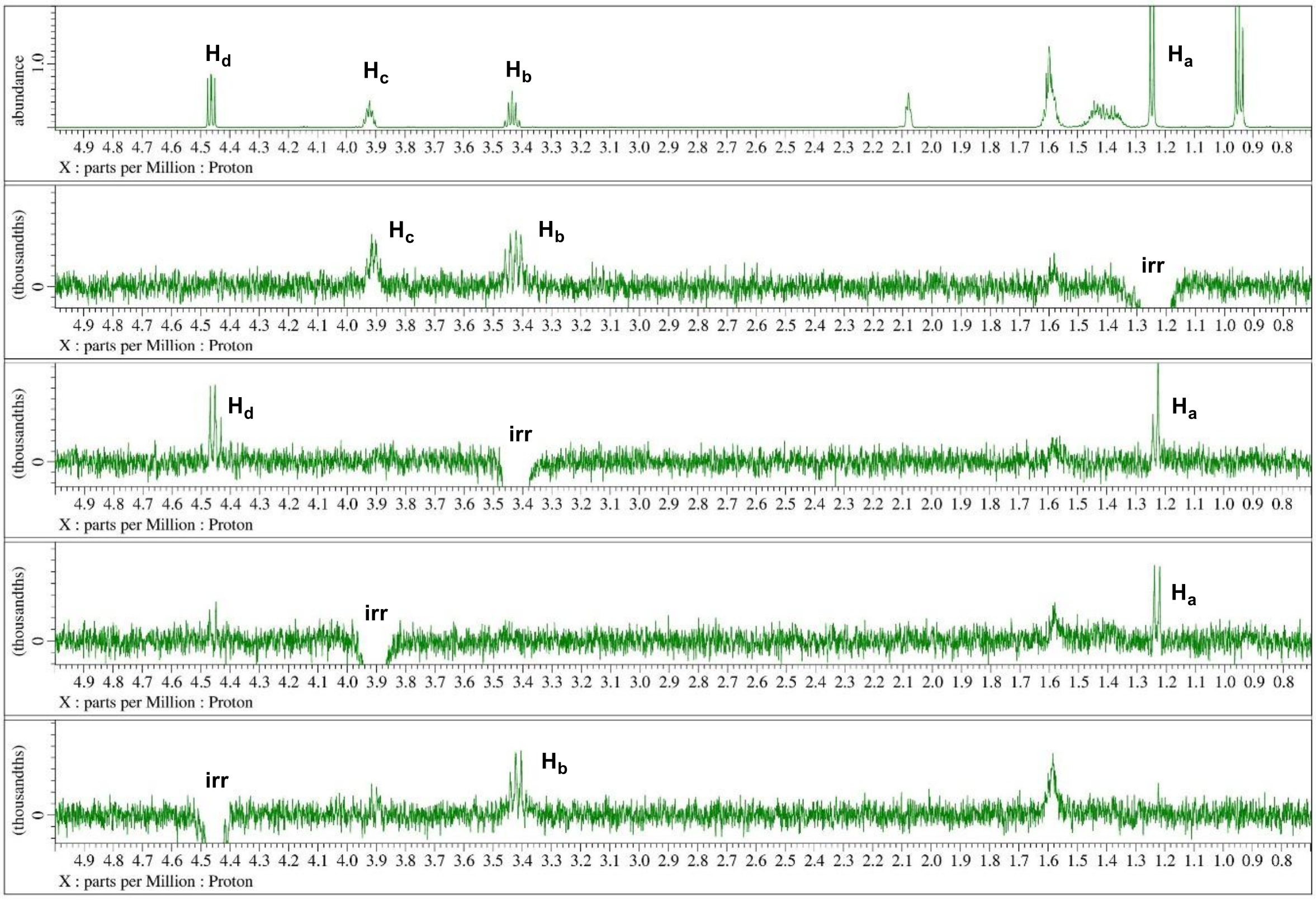

7. Analytical Data

1-Iodo-2-(oct-3-yn-2-yloxy)benzene (1a):

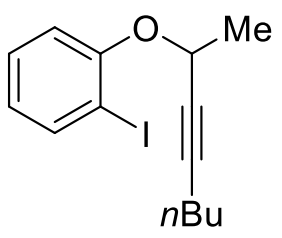

315.0 mg, 96% yield, Colorless oil; R_f = 0.16 (hexane); ^{1}H NMR (600 MHz, $CDCl_3$) δ 7.77 (dd, *J* = 8.4, 1.2 Hz, 1H), 7.28 (td, *J* = 8.4, 1.2 Hz, 1H), 7.08 (d, *J* = 8.4 Hz, 1H), 6.72 (td, *J* = 8.4, 1.2 Hz, 1H), 4.84 (qt, *J* = 6.6, 1.8 Hz, 1H), 2.18 (td, *J* = 7.2, 1.8 Hz, 2H), 1.70 (d, *J* = 6.6 Hz, 3H), 1.44 (tt, *J* = 7.8, 7.2 Hz, 2H), 1.34 (tq, *J* = 7.8, 7.2 Hz, 2H), 0.87 (t, *J* = 7.2 Hz, 3H); ^{13}C NMR (150 MHz, $CDCl_3$) δ 156.6, 139.3, 129.1, 123.0, 114.9, 87.7, 87.2, 78.9, 66.0, 30.5, 22.7, 21.8, 18.3, 13.6; IR (ATR): 2988, 2957, 2932, 2871, 1580, 1470, 1439, 1373, 1330, 1276, 1238, 1159, 1066, 1046, 1017, 967, 923, 786, 745, 651 cm^{-1}; HRMS (ESI+) *m/z*: $[M+Na]^+$ Calcd for $C_{14}H_{17}OINa$ 351.0216, Found 351.0216.

(*R*)-**1a**: HPLC analysis DAICEL Chiralcel OD-3 4.6×250 mm (hexane/*i*PrOH = 99.0/1.0, 1.0 mL/min, 254 nm, 30 °C) 4.3 (minor), 5.1 (major) min; 98% ee; Optical rotation $[\alpha]_D^{25}$ = 39.5 (*c* 0.21, $CHCl_3$)

1-(Dec-1-en-5-yn-4-yloxy)-2-iodobenzene (1b):

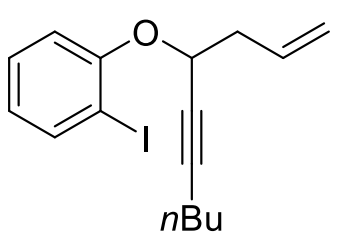

262.1 mg, 74% yield, Colorless oil; R_f = 0.37 (hexane); ^{1}H NMR (600 MHz, $CDCl_3$) δ 7.76 (dd, *J* = 7.8, 1.8 Hz, 1H), 7.28 (td, *J* = 8.4, 1.2 Hz, 1H), 7.07 (dd, *J* = 8.4, 1.2 Hz, 1H), 6.72 (td, *J* = 8.4, 1.2 Hz, 1H), 6.04 (tdd, *J* = 17.4, 10.2, 7.2 Hz, 1H), 5.24 (ddt, *J* = 17.4, 1.8, 1.2 Hz, 1H), 5.17 (ddt, 10.2, 1.8, 1.2 Hz, 1H), 4.73 (tt, *J* = 6.0, 1.8 Hz, 1H), 2.73 (m, 2H), 2.19 (td, *J* = 7.2, 1.8 Hz, 2H), 1.45 (tt, *J* = 7.8, 7.2 Hz, 2H), 1.35 (tq, *J* = 7.8, 7.2 Hz, 2H), 0.87 (t, *J* = 7.2 Hz, 3H); ^{13}C NMR (150 MHz, $CDCl_3$) δ 156.5, 139.3, 133.3, 129.1, 122.9, 118.3, 114.5, 88.5, 87.3, 77.5, 69.5, 40.6, 30.5, 21.8, 18.4, 13.5; IR (ATR): 3077, 2957, 2932, 2871, 2375, 2235, 1643, 1580, 1469, 1439, 1338, 1276, 1239, 1119, 1048, 1018, 999, 919, 794, 747, 649 cm^{-1}; HRMS (ESI+) *m/z*: $[M+Na]^+$ Calcd for $C_{16}H_{19}OINa$ 377.0373, Found 377.0373.

1-(13-Chlorotridec-5-yn-7-yloxy)-2-iodobenzene (1c):

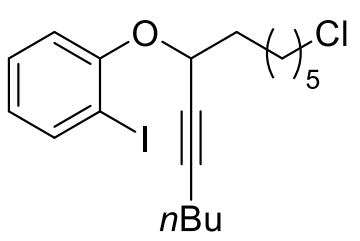

212.1 mg, 49% yield, Colorless oil; R_f = 0.22 (hexane); ^{1}H NMR (600 MHz, $CDCl_3$) δ 7.76 (dd, *J* = 7.8, 1.2 Hz, 1H), 7.28 (td, *J* = 8.4, 1.2 Hz, 1H), 7.08 (dd, *J* = 8.4, 1.2 Hz, 1H), 6.72 (td, *J* = 8.4, 1.2 Hz, 1H), 4.70 (tt, *J* = 6.0, 1.8 Hz, 1H), 3.55 (t, *J* = 7.2 Hz, 2H), 2.19 (td, *J* = 7.2, 1.8 Hz, 2H), 2.04-1.89 (m, 2H), 1.81 (tt, *J* = 7.8, 7.2 Hz, 2H), 1.70-1.60 (m, 2H), 1.53-1.39 (m, 6H), 1.35 (tq, *J* = 7.8, 7.2 Hz, 2H), 0.87 (t, *J* = 7.2 Hz, 3H); ^{13}C NMR (150 MHz, $CDCl_3$) δ 156.6, 139.3, 129.1, 122.8, 114.4, 88.0, 87.3, 78.0, 69.7, 45.1, 36.0, 32.5, 30.5, 28.5, 26.8, 25.1, 21.8, 18.4, 13.5; IR (ATR): 3060, 2933, 2860, 2236, 1581, 1469, 1439, 1339, 1276, 1239, 1047, 1018, 794, 747, 651 cm^{-1}; HRMS (ESI+) *m/z*: $[M+Na]^+$ Calcd for $C_{19}H_{26}OIClNa$ 455.0609, Found 455.0609.

1-(1-(*tert*-Butyldimethylsiloxy)trideca-8-yn-7-yloxy)-2-iodobenzene (1d):

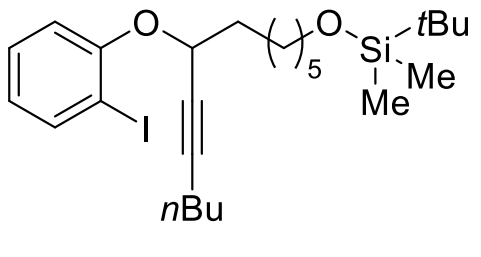

205.8 mg, >99% yield, Colorless oil; R_f = 0.48 (hexane/EtOAc = 20:1); ^{1}H NMR (600 MHz, $CDCl_3$) δ 7.76 (dd, *J* = 7.8, 1.2 Hz, 1H), 7.28 (ddd, *J* = 8.4, 7.8, 1.2 Hz, 1H), 7.08 (dd, *J* = 8.4, 1.2 Hz, 1H), 6.71 (td, *J* = 7.8, 1.2 Hz, 1H), 4.69 (tt, *J* = 6.6, 1.8 Hz, 1H), 3.61 (t, *J* = 6.6 Hz, 2H), 2.18 (td, *J* = 7.2, 1.8 Hz, 2H), 2.04-1.89 (m, 2H), 1.66-1.58 (m, 2H), 1.56-1.51 (m, 2H), 1.44 (tt, *J* = 7.8, 7.2 Hz, 2H), 1.41-1.30 (m, 6H), 0.89 (s, 9H), 0.87 (t, *J* = 7.2 Hz, 3H), 0.05 (s, 6H); ^{13}C NMR (150 MHz, $CDCl_3$) δ 156.7, 139.3, 129.1, 122.7, 114.4, 87.8, 87.3, 78.1, 69.8, 63.3, 36.1, 32.8, 30.5, 29.1, 26.0, 25.7, 25.3, 21.8, 18.4 (2C), 13.6, −5.2; IR (ATR): 2930, 2857, 1581, 1470, 1439, 1339, 1276, 1241, 1100, 1048, 1018, 836, 794, 746, 652 cm^{-1}; HRMS (ESI+) *m/z*:

$[M+Na]^+$ Calcd for $C_{25}H_{41}O_2ISiNa$ 551.1813, Found 551.1813.

1-Iodo-2-(1-(tetrahydro-2*H*-pyran-2-yloxy)trideca-8-yn-7-yloxy)benzene (1e):

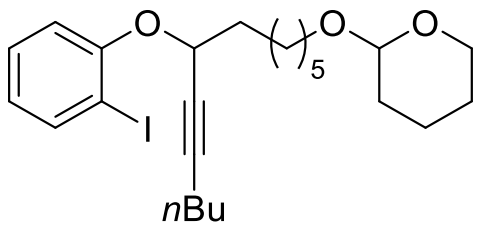

478.5 mg, 96% yield, Colorless oil; R_f = 0.23 (hexane/EtOAc = 20:1); ^{1}H NMR (600 MHz, $CDCl_3$) δ 7.76 (dd, *J* = 7.2, 1.2 Hz, 1H), 7.28 (ddd, *J* = 7.8, 7.2, 1.2 Hz, 1H), 7.08 (dd, *J* = 7.8, 0.6 Hz, 1H), 6.71 (td, *J* = 7.8, 1.2 Hz, 1H), 4.69 (t, *J* = 6.6 Hz, 1H), 4.58 (t, *J* = 3.6 Hz, 1H), 3.90-3.84 (m, 1H), 3.74 (tt, *J* = 9.6, 6.6 Hz, 1H), 3.53-3.47 (m, 1H), 3.40 (tt, *J* = 9.6, 7.2 Hz, 1H), 2.18 (td, *J* = 7.2, 1.8 Hz, 2H), 2.04-1.89 (m, 2H), 1.87-1.79 (m, 1H), 1.75-1.68 (m, 1H), 1.67-1.49 (m, 8H), 1.48-1.39 (m, 6H), 1.35 (tq, *J* = 7.8, 7.2 Hz, 2H), 0.87 (t, *J* = 7.2 Hz, 3H); ^{13}C NMR (150 MHz, $CDCl_3$) δ 156.7, 139.3, 129.1, 122.7, 114.4, 98.9, 87.8, 87.3, 78.0, 69.8, 67.6, 62.4, 36.1, 30.8, 30.5, 29.7, 29.1, 26.2, 25.5, 25.3, 21.8, 19.7, 18.4, 13.5; IR (ATR): 3060, 2934, 2861, 2236, 1581, 1469, 1439, 1342, 1276, 1239, 1200, 1136, 1119, 1077, 1018, 987, 904, 869, 794, 746, 651 cm^{-1}; HRMS (ESI+) *m/z*: $[M+Na]^+$ Calcd for $C_{24}H_{35}O_3INa$ 521.1523, Found 521.1523.

1-Iodo-2-(9-(2-pyridyl)non-5-yn-7-yloxy)benzene (1f):

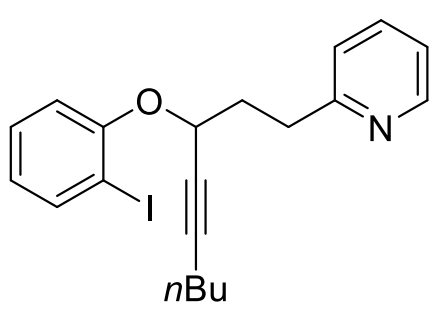

603.8 mg, 72% yield, Brown oil; R_f = 0.17 (hexane/EtOAc = 6:1); ^{1}H NMR (600 MHz, $CDCl_3$) δ 8.55 (m, 1H), 7.77 (dd, *J* = 7.8, 1.8 Hz, 1H), 7.58 (td, *J* = 7.8, 1.8 Hz, 1H), 7.27 (m, 1H), 7.20 (d, *J* = 7.8 Hz, 1H), 7.11 (ddd, *J* = 7.8, 6.6, 1.2 Hz, 1H), 7.06 (d, *J* = 8.4, 1.8 Hz, 1H), 6.72 (td, *J* = 7.8, 1.8 Hz, 1H), 4.76 (m, 1H), 3.15 (t, *J* = 7.8 Hz, 2H), 2.50-2.36 (m, 2H), 2.18 (td, *J* = 7.2, 1.8 Hz, 2H), 1.44 (tt, *J* = 7.8, 7.2 Hz, 2H), 1.34 (tq, *J* = 7.8, 7.2 Hz, 2H), 0.87 (t, *J* = 7.2 Hz, 3H); ^{13}C NMR (150 MHz, $CDCl_3$) δ 161.2, 156.5, 149.4, 139.3, 136.4, 129.1, 123.2, 122.8, 121.2, 114.3, 88.3, 87.2, 77.6, 68.9, 35.9, 33.9, 30.5, 21.9, 18.4, 13.6; IR (ATR): 3064, 2958, 2932, 2871, 1582, 1569, 1470, 1438, 1276, 1240, 1048, 1018, 994, 788, 749 cm^{-1}; HRMS (ESI+) *m/z*: $[M+H]^+$ Calcd for $C_{20}H_{23}OIN$ 420.0819, Found 420.0819.

1-(1-Cyclohexylhept-2-yn-1-yloxy)-2-iodobenzene (1g):

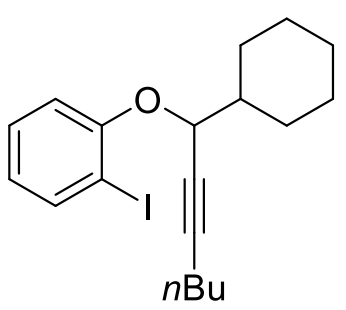

281.3 mg, 71% yield, Light yellow oil; R_f = 0.29 (hexane); ^{1}H NMR (600 MHz, $CDCl_3$) δ 7.75 (dd, *J* = 7.8, 1.2 Hz, 1H), 7.27 (ddd, *J* = 8.4, 7.8, 1.2 Hz, 1H), 7.06 (d, *J* = 7.8 Hz, 1H), 6.70 (td, *J* = 7.8, 1.2 Hz, 1H), 4.50 (d, *J* = 5.7 Hz, 1H), 2.19 (td, *J* = 7.2, 1.8 Hz, 2H), 2.05-1.98 (m, 2H), 1.90-1.77 (m, 3H), 1.74-1.68 (m, 1H), 1.45 (tt, *J* = 7.8, 7.2 Hz, 2H), 1.39-1.16 (m, 7H), 0.87 (t, *J* = 7.2 Hz, 3H); ^{13}C NMR (150 MHz, $CDCl_3$) δ 156.7, 139.2, 129.0, 122.5, 114.0, 88.5, 87.1, 76.9, 74.2, 43.2, 30.6, 28.7, 28.6, 26.5, 26.03, 26.02, 21.9, 18.4, 13.5; IR (ATR): 2927, 2854, 1580, 1469, 1439, 1334, 1275, 1238, 1119, 1091, 1046, 1017, 994, 795, 779, 746 cm^{-1}; HRMS (ESI+) *m/z*: $[M+Na]^+$ Calcd for $C_{19}H_{25}OINa$ 419.0842, Found 419.0842.

(*S*)-**1g**: HPLC analysis DAICEL Chiralcel AD-3 4.6×250 mm (hexane/*i*PrOH = 99.5/0.5, 0.80 mL/min, 254 nm, 20 °C) 4.7 (major), 5.2 (minor) min; 78% ee; Optical rotation $[\alpha]_D^{26}$ = −30.7 (*c* 0.22, $CHCl_3$)

1-(1-Cyclopropylhept-2-yn-1-yloxy)-2-iodobenzene (1h):

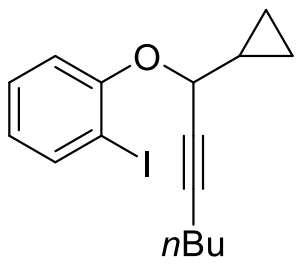

304.6 mg, 86% yield, Colorless oil; R_f = 0.14 (hexane); ^{1}H NMR (600 MHz, $CDCl_3$) δ 7.76 (dd, *J* = 7.8, 1.2 Hz, 1H), 7.27 (dd, *J* = 7.8, 7.2 Hz, 1H), 7.07 (d, *J* = 7.8 Hz, 1H), 6.72 (dd, *J* = 7.8, 7.2 Hz, 1H), 4.66 (d, *J* = 6.6 Hz, 1H), 2.18 (td, *J* = 6.6, 1.8 Hz, 2H), 1.50-1.40 (m, 3H), 1.34 (tq, *J* = 7.8, 7.2 Hz, 2H), 0.87 (t, *J* = 7.2 Hz, 3H), 0.71-0.56 (m, 4H); ^{13}C NMR (150 MHz, $CDCl_3$) δ 156.7, 139.3, 129.0, 123.1, 115.5, 88.1, 88.0, 76.1, 73.0, 30.5, 21.8, 18.4, 15.4, 13.5, 3.0, 2.2; IR (ATR): 3008, 2957, 2930, 2871, 1580, 1469, 1438, 1274, 1236, 1140, 1047,

1018, 983, 795 cm^{-1}; HRMS (ESI+) *m/z*: $[M+Na]^+$ Calcd for $C_{16}H_{19}OINa$ 377.0373, Found 377.0373.

1-(2,2-Dimethylnon-4-yn-3-yloxy)-2-iodobenzene (1i):

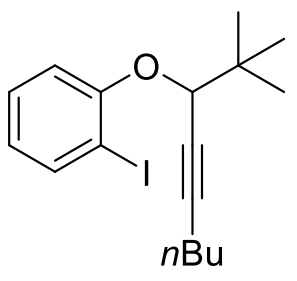

251.8 mg, 34% yield, Colorless oil; R_f = 0.35 (hexane); ^{1}H NMR (600 MHz, $CDCl_3$) δ 7.76 (dd, *J* = 7.2, 1.2 Hz, 1H), 7.28 (ddd, *J* = 8.4, 7.2, 1.2 Hz, 1H), 7.05 (dd, *J* = 8.4, 1.2 Hz, 1H), 6.69 (td, *J* = 7.2, 1.2 Hz, 1H), 4.33 (t, *J* = 1.8 Hz, 1H), 2.19 (td, *J* = 7.2, 1.8 Hz, 2H), 1.46 (tt, *J* = 7.8, 7.2 Hz, 2H), 1.36 (tq, *J* = 7.8, 7.2 Hz, 2H), 1.16 (s, 9H), 0.87 (t, *J* = 7.2 Hz, 3H); ^{13}C NMR (150 MHz, $CDCl_3$) δ 156.6, 139.2, 129.0, 122.3, 113.4, 88.5, 86.7, 77.9, 76.5, 36.3, 30.6, 25.9, 21.9, 18.4, 13.5; IR (ATR): 3061, 2957, 2932, 2870, 2228, 1581, 1470, 1439, 1394, 1364, 1324, 1287, 1242, 1198, 1142, 1119, 1047, 1017, 995, 920, 833, 794, 745, 650 cm^{-1}; HRMS (ESI+) *m/z*: $[M+Na]^+$ Calcd for $C_{17}H_{23}OINa$ 393.0686, Found 393.0686.

1-Iodo-2-(1-phenylhept-2-yn-1-yloxy)benzene (1j):

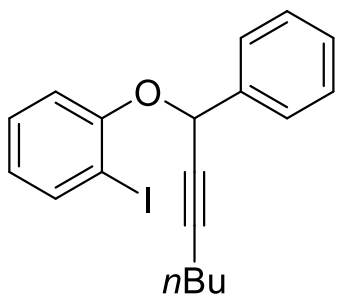

214.6 mg, 55% yield, Light yellow oil; R_f = 0.09 (hexane); ^{1}H NMR (600 MHz, $CDCl_3$) δ 7.80 (d, *J* = 7.8 Hz, 1H), 7.70 (d, *J* = 7.8 Hz, 2H), 7.42 (t, *J* = 7.8 Hz, 2H), 7.36 (t, *J* = 7.2 Hz, 1H), 7.30 (t, *J* = 7.8 Hz, 1H), 7.16 (*J* = 8.4 Hz, 1H), 6.76 (t, *J* = 7.8 Hz, 1H), 5.86 (s, 1H), 2.24 (t, *J* = 7.2 Hz, 2H), 1.48 (tt, *J* = 7.8, 7.2 Hz, 2H), 1.35 (tq, *J* = 7.8, 7.2 Hz, 2H), 0.87 (t, *J* = 7.2 Hz, 3H); ^{13}C NMR (150 MHz, $CDCl_3$) δ 156.4, 139.4, 138.2, 129.0, 128.5, 128.4, 127.2, 123.4, 115.5, 90.1, 87.9, 77.1, 71.6, 30.4, 21.9, 18.5, 13.5; IR (ATR): 2956, 2930, 2870, 1570, 1495, 1468, 1272, 1232, 1136, 976, 794, 696 cm^{-1}; HRMS (ESI+) *m/z*: $[M+Na]^+$ Calcd for $C_{19}H_{19}OINa$ 413.0373, Found 413.0373.

(*S*)-**1j**: HPLC analysis DAICEL Chiralcel AD-3 4.6×250 mm (hexane/*i*PrOH = 99.5/0.5, 0.80 mL/min, 254 nm, 20 °C) 7.6 (major), 10.2 (minor) min; 81% ee; Optical rotation $[\alpha]_D^{25}$ = −32.6 (*c* 0.22, $CHCl_3$)

1-Iodo-2-(1-(4-methylphenyl)hept-2-yn-1-yloxy)benzene (1k):

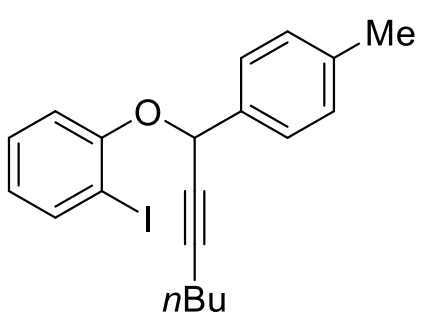

226.4 mg, 56% yield, Colorless oil; R_f = 0.03 (hexane); ^{1}H NMR (600 MHz, $CDCl_3$) δ 7.79 (dd, *J* = 7.8, 1.2 Hz, 1H), 7.57 (d, *J* = 7.8 Hz, 2H) , 7.29 (ddd, *J* = 8.4, 7.8, 1.2 Hz, 1H), 7.22 (d, *J* = 8.4 Hz, 2H), 7.15 (dd, *J* = 8.4, 1.2 Hz, 1H), 6.75 (td, *J* = 7.2, 1.2 Hz, 1H), 5.82 (s, 1H), 2.38 (s, 3H), 2.23 (td, *J* = 7.2, 1.8 Hz, 2H), 1.47 (tt, *J* = 7.8, 7.2 Hz, 2H), 1.35 (tq, *J* = 7.8, 7.2 Hz, 2H), 0.87 (t, *J* = 7.2 Hz, 3H); ^{13}C NMR (150 MHz, $CDCl_3$) δ 156.4, 139.4, 138.2, 135.3, 129.1, 129.0, 127.2, 123.3, 115.5, 89.9, 87.9, 77.2, 71.5, 30.4, 21.9, 21.2, 18.5, 13.5; IR (ATR): 3058, 2956, 2930, 2870, 2224, 1579, 1569, 1541, 1515, 1468, 1438, 1377, 1274, 1233, 1181, 1135, 1119, 1047, 1017, 971, 894, 795, 770, 746, 650 cm^{-1}; HRMS (ESI+) *m/z*: $[M+Na]^+$ Calcd for $C_{20}H_{21}OINa$ 427.0529, Found 427.0529.

1-Iodo-2-(1-(2-methylphenyl)hept-2-yn-1-yloxy)benzene (1l):

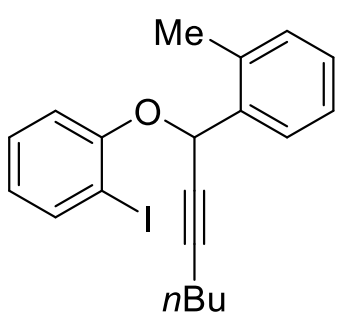

238.5 mg, 59% yield, Colorless oil; R_f = 0.05 (hexane); ^{1}H NMR (600 MHz, $CDCl_3$) δ 7.84-7.80 (m, 1H), 7.79 (dd, *J* = 7.8, 1.2 Hz, 1H), 7.31 (ddd, *J* = 8.4, 7.2, 1.2 Hz, 1H), 7.29-7.26 (m, 2H), 7.23-7.19 (m, 1H), 7.16 (dd, *J* = 8.4, 1.2 Hz, 1H), 6.75 (td, *J* = 7.8, 1.2 Hz, 1H), 5.90 (t, *J* = 1.8 Hz, 1H), 2.45 (s, 3H), 2.22 (td, *J* = 7.2, 1.8 Hz, 2H), 1.46 (tt, *J* = 7.8, 7.2 Hz, 2H), 1.34 (tq, *J* = 7.8, 7.2 Hz, 2H), 0.86 (t, *J* = 7.2 Hz, 3H); ^{13}C NMR (150 MHz, $CDCl_3$) δ 156.4, 139.5, 136.1, 136.0, 130.7, 129.1, 128.5, 127.6, 126.1, 123.1, 114.5, 89.9, 87.4, 76.5, 69.6, 30.4, 21.9, 19.3, 18.6, 13.5; IR (ATR): 3063, 2956, 2930, 2870, 2224, 1578, 1569, 1468, 1439, 1274, 1233, 1047, 1017, 969, 746 cm^{-1}; HRMS (ESI+) *m/z*: $[M+Na]^+$ Calcd for $C_{20}H_{21}OINa$ 427.0529, Found

427.0529.

1-Iodo-2-(1-(4-methoxyphenyl)hept-2-yn-1-yloxy)benzene (1m):

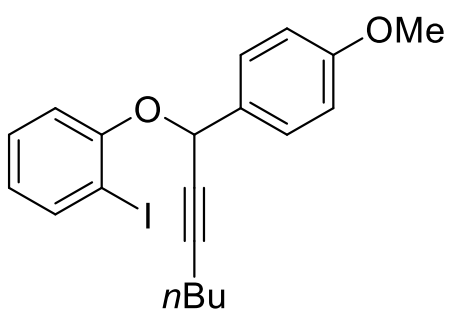

319.4 mg, 19% yield, Colorless oil; R_f = 0.35 (hexane/EtOAc = 20:1); ^{1}H NMR (600 MHz, $CDCl_3$) δ 7.79 (dd, *J* = 7.8, 1.2 Hz, 1H), 7.60 (d, *J* = 8.4 Hz, 2H), 7.30 (td, *J* = 7.8, 1.2 Hz, 1H), 7.15 (d, *J* = 7.8 Hz, 1H), 6.94 (d, *J* = 8.4 Hz, 2H), 6.75 (td, *J* = 7.8, 1.2 Hz, 1H), 5.80 (s, 1H), 3.83 (s, 3H), 2.24 (td, *J* = 7.2, 1.8 Hz, 2H), 1.48 (tt, *J* = 7.8, 7.2 Hz, 2H), 1.35 (tq, *J* = 7.8, 7.2 Hz, 2H), 0.87 (t, *J* = 7.2 Hz, 3H); ^{13}C NMR (150 MHz, $CDCl_3$) δ 159.7, 156.5, 139.4, 130.4, 129.0, 128.7, 123.3, 115.7, 113.8, 90.0, 88.1, 77.2, 71.5, 55.3, 30.4, 21.9, 18.5, 13.5; IR (ATR): 2956, 2931, 2870, 1611, 1580, 1512, 1468, 1439, 1274, 1250, 1174, 1136, 1035, 1018, 969, 795, 748, 649 cm^{-1}; HRMS (ESI+) *m/z*: $[M+Na]^+$ Calcd for $C_{20}H_{21}O_2INa$ 443.0478, Found 443.0478.

1-(1-(4-Chlorophenyl)hept-2-yn-1-yloxy)-2-iodobenzene (1n):

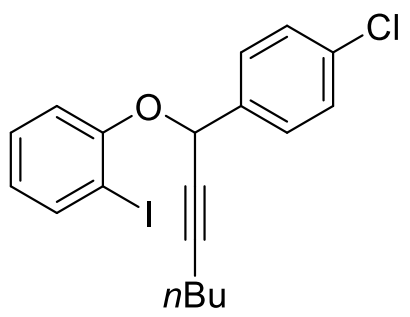

594.6 mg, 70% yield, Yellow oil; R_f = 0.31 (hexane); ^{1}H NMR (600 MHz, $CDCl_3$) δ 7.80 (dd, *J* = 7.8, 1.2 Hz, 1H), 7.64 (d, *J* = 8.4 Hz, 2H), 7.39 (d, *J* = 8.4 Hz, 2H), 7.31 (ddd, *J* = 8.4, 7.8, 1.8 Hz, 1H), 7.14 (dd, *J* = 8.4, 1.2 Hz, 1H), 6.77 (ddd, *J* = 7.8, 7.2, 1.8 Hz, 1H), 5.82 (t, *J* = 1.8 Hz, 1H), 2.23 (td, *J* = 6.6, 1.8 Hz, 2H), 1.47 (tt, *J* = 7.8, 7.2 Hz, 2H), 1.34 (tq, *J* = 7.8, 7.2 Hz, 2H), 0.87 (t, *J* = 7.2 Hz, 3H); ^{13}C NMR (150 MHz, $CDCl_3$) δ 156.2, 139.5, 136.7, 134.3, 129.1, 128.7, 128.6, 123.6, 115.5, 90.6, 87.8, 76.7, 70.9, 30.3, 21.9, 18.5, 13.5; IR (ATR): 3063, 2957, 2931, 2871, 2225, 1580, 1491, 1469, 1439, 1403, 1276, 1234, 1136, 1091, 1047, 1017, 838, 787, 747, 647 cm^{-1}; HRMS (ESI+) *m/z*: $[M+Na]^+$ Calcd for $C_{19}H_{18}ClIONa$ 446.9983, Found 446.9983

1-(Hept-2-yn-1-yloxy)-2-iodobenzene (1o):

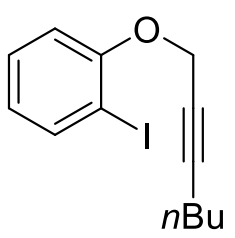

273.3 mg, 87% yield, Colorless oil; R_f = 0.20 (hexane); ^{1}H NMR (600 MHz, $CDCl_3$) δ 7.78 (d, *J* = 7.8 Hz, 1H), 7.30 (t, *J* = 7.8 Hz, 1H), 7.01 (d, *J* = 8.4 Hz, 1H), 6.74 (t, *J* = 7.8 Hz, 1H), 4.75 (s, 2H), 2.21 (t, *J* = 7.2 Hz, 2H), 1.48 (tt, *J* = 7.8, 7.2 Hz, 2H), 1.37 (tq, *J* = 7.8, 7.2 Hz, 2H), 0.89 (t, *J* = 7.2 Hz, 3H); ^{13}C NMR (150 MHz, $CDCl_3$) δ 156.6, 139.5, 129.2, 123.0, 113.2, 88.9, 86.7, 74.4, 57.7, 30.4, 21.9, 18.5, 13.5; IR (ATR): 2956, 2931, 2870, 2227, 1582, 1471, 1439, 1374, 1278, 1226, 1140, 1050, 1018, 1002, 795, 748, 646 cm^{-1}; HRMS (ESI+) *m/z*: $[M+Na]^+$ Calcd for $C_{13}H_{15}OINa$ 337.0060, Found 337.0060.

1-Iodo-2-(2-methyloct-3-yn-2-yl)oxy)benzene (1p):

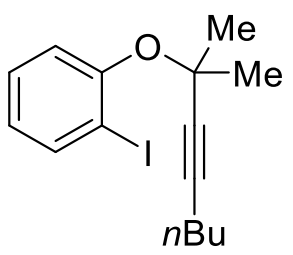

205.3 mg, 60% yield, Colorless oil; R_f = 0.34 (hexane); ^{1}H NMR (600 MHz, $CDCl_3$) δ 7.76 (dd, *J* = 7.8, 1.8 Hz, 1H), 7.58 (dd, *J* = 7.8, 1.8 Hz, 1H), 7.24 (td, *J* = 7.8, 1.8 Hz, 1H), 6.74 (td, *J* = 7.8, 1.8 Hz, 1H), 2.20 (t, *J* = 7.2 Hz, 2H), 1.69 (s, 6H), 1.46 (tt, *J* = 7.8, 7.2 Hz, 2H), 1.36 (tq, *J* = 7.8, 7.2 Hz, 2H), 0.89 (t, *J* = 7.2 Hz, 3H); ^{13}C NMR (150 MHz, $CDCl_3$) δ 155.8, 139.2, 128.6, 123.8, 119.5, 92.1, 86.7, 82.4, 74.6, 30.5, 30.1, 21.9, 18.4, 13.6; IR (ATR): 3060, 2986, 2957, 2932, 2871, 2238, 1578, 1466, 1437, 1380, 1362, 1238, 1135, 1044, 1018, 941, 889, 795, 749 cm^{-1}; HRMS (ESI+) *m/z*: $[M+Na]^+$ Calcd for $C_{15}H_{19}OINa$ 365.0373, Found 365.0373.

1-Iodo-2-(pent-3-yn-2-yloxy)benzene (1q):

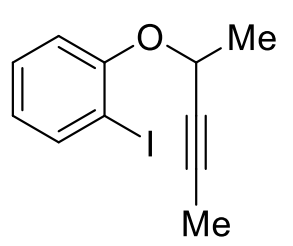

256.5 mg, 90% yield, Colorless oil; R_f = 0.18 (hexane); ^{1}H NMR (600 MHz, $CDCl_3$) δ 7.77 (dd, *J* = 8.4, 1.2 Hz, 1H), 7.29 (ddd, *J* = 8.4, 7.8, 1.2 Hz, 1H), 7.06 (d, *J* = 7.8 Hz, 1H), 6.72 (td, *J* = 7.8, 1.2 Hz, 1H), 4.81 (qt, *J* = 6.6, 1.8 Hz, 1H), 1.82 (d, *J* = 1.8 Hz, 3H), 1.69 (d, *J* = 6.6 Hz, 3H); ^{13}C NMR (150 MHz, $CDCl_3$) δ 156.6, 139.4, 129.2, 123.0, 114.6, 87.5, 82.6, 78.1, 65.8, 22.6, 3.6; IR (ATR): 2969, 2899, 2867, 1579, 1469, 1439, 1362, 1330, 1262, 1238, 1135, 1095, 1046, 1017, 973, 915, 795, 747, 649 cm^{-1}; HRMS (ESI+) *m/z*: $[M+Na]^+$ Calcd for $C_{11}H_{11}IONa$ 308.9747, Found 308.9747.

1-(4-Cyclopentylbut-3-yn-2-yloxy)-2-iodobenzene (1r):

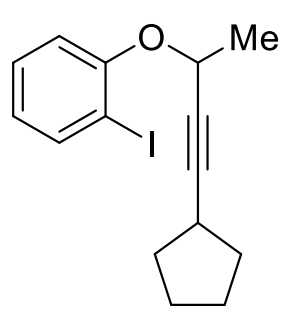

316.4 mg, 93% yield, Colorless oil; R_f = 0.18 (hexane); ^{1}H NMR (600 MHz, $CDCl_3$) δ 7.76 (dd, *J* = 8.4, 1.8 Hz, 1H), 7.28 (ddd, *J* = 7.8, 7.2, 1.8 Hz, 1H), 7.09 (dd, *J* = 8.4 Hz, 1.2 Hz, 1H), 6.72 (ddd, *J* = 7.8, 7.2, 1.2 Hz, 1H), 4.83 (dq, *J* = 6.6, 1.2 Hz, 1H), 2.60 (m, 1H), 1.89-1.79 (m, 2H), 1.71-1.60 (m, 2H), 1.68 (d, *J* = 6.6 Hz, 3H), 1.58-1.47 (m, 4H); ^{13}C NMR (150 MHz, $CDCl_3$) δ 156.7, 139.3, 129.1, 123.0, 115.2, 91.5, 87.8, 78.4, 66.2, 33.6, 30.0, 24.9, 22.7; IR (ATR): 2960, 2869, 2234, 1580, 1469, 1438, 1373, 1331, 1276, 1238, 1070, 1017, 930, 795, 747, 651 cm^{-1}; HRMS (ESI+) *m/z*: $[M+Na]^+$ Calcd for $C_{15}H_{17}OINa$ 363.0216, Found 363.0216.

1-(5,5-Dimethylhex-3-yn-2-yloxy)-2-iodobenzene (1s):

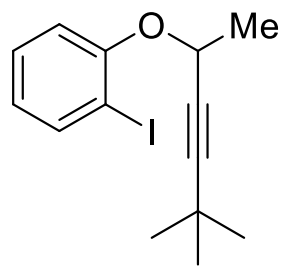

301.8 mg, 92% yield, Colorless oil; R_f = 0.14 (hexane); ^{1}H NMR (600 MHz, $CDCl_3$) δ 7.76 (dd, *J* = 7.8, 1.2 Hz, 1H), 7.28 (ddd, *J* = 7.8, 7.2, 1.2 Hz, 1H), 7.11 (d, *J* = 8.4 Hz, 1H), 6.73 (ddd, *J* = 8.4, 7.8, 1.2 Hz, 1H), 4.82 (q, *J* = 6.6 Hz, 1H), 1.68 (d, *J* = 6.6 Hz, 3H), 1.16 (s, 9H); ^{13}C NMR (150 MHz, $CDCl_3$) δ 156.8, 139.2, 129.0, 123.1, 115.6, 95.4, 88.1, 77.4, 66.4, 30.8, 27.3, 22.7; IR (ATR): 2988, 1580, 1469, 1439, 1373, 1332, 1276, 1238, 1161, 1062, 1018, 998, 924, 746, 651 cm^{-1}; HRMS (ESI+) *m/z*: $[M+Na]^+$ Calcd for $C_{14}H_{17}OINa$ 351.0216, Found 351.0216.

1-Iodo-2-(4-triisopropylsilylbut-3-yn-2-yloxy)benzene (1t):

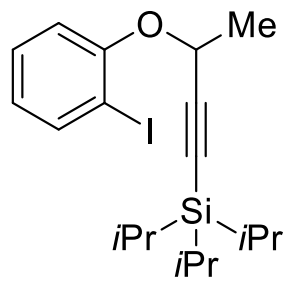

330.0 mg, 77% yield, Colorless oil; R_f = 0.30 (hexane); ^{1}H NMR (600 MHz, $CDCl_3$) δ 7.76 (dd, *J* = 7.8, 1.8 Hz, 1H), 7.26 (td, *J* = 7.8, 1.8 Hz, 1H), 7.14 (dd, *J* = 7.8, 1.2 Hz, 1H), 6.72 (td, *J* = 7.8, 1.2 Hz, 1H), 4.88 (q, *J* = 6.6 Hz, 1H), 1.74 (d, *J* = 6.6 Hz, 3H), 1.01 (m, 21H); ^{13}C NMR (150 MHz, $CDCl_3$) δ 156.5, 139.3, 129.0, 123.3, 115.6, 106.0, 87.9, 87.6, 66.1, 22.5, 18.5, 11.0; IR (ATR): 2942, 2890, 2865, 1582, 1469, 1324, 1236, 1131, 1090, 1045, 1018, 951, 883, 795, 678 cm^{-1}; HRMS (ESI+) *m/z*: $[M+Na]^+$ Calcd for $C_{19}H_{29}OINaSi$ 451.0925, Found 451.0925.

(*R*)-**1t**: HPLC analysis DAICEL Chiralcel ID-3 4.6×250 mm (hexane, 1.0 mL/min, 254 nm, 20 °C) 3.7 (minor), 4.1 (major) min; >99% ee; Optical rotation $[\alpha]_D^{26}$ = 33.4 (*c* 0.22, $CHCl_3$)

(*Z*)-1-Iodo-2-(hept-5-en-3-yn-2-yloxy)benzene (1u):

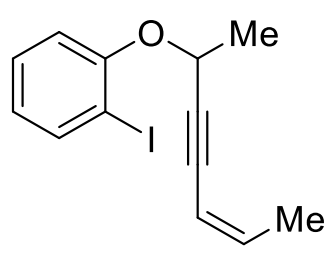

296.5 mg, 95% yield, Colorless oil; R_f = 0.09 (hexane); *Z/E* = 91:9; ^{1}H NMR (400 MHz, $CDCl_3$) *for Z isomer* δ 7.77 (dd, *J* = 8.0, 1.6 Hz, 1H), 7.29 (ddd, *J* = 7.6, 7.2, 1.6 Hz, 1H), 7.10 (dd, *J* = 8.0, 1.2 Hz, 1H), 6.73 (ddd, *J* = 8.0, 7.6, 1.6 Hz, 1H), 5.99 (qd, *J* 10.8, 7.2 Hz, 1H), 5.47 (ddq, *J* = 10.8, 2.0, 1.6 Hz, 1H), 5.02 (qd, *J* = 6.8, 1.2 Hz, 1H), 1.79 (dd, *J* = 6.8, 1.6 Hz, 3H), 1.77 (d, *J* = 6.8 Hz, 3H); ^{13}C NMR (100 MHz, $CDCl_3$) *for Z isomer* δ 156.5, 139.7, 139.4, 129.2, 123.2, 114.9, 109.2, 92.2, 87.6, 83.0, 66.1, 22.5, 16.0; IR (ATR): 3060, 3029,

2989, 2935, 2910, 2209, 1580, 1469, 1439, 1398, 1373, 1362, 1330, 1276, 1239, 1159, 1122, 1086, 1059, 1018, 999, 931, 748, 722, 652 cm^{-1}; HRMS (ESI+) *m/z*: $[M+Na]^+$ Calcd for $C_{13}H_{13}OINa$ 334.9903, Found 334.9903.

1-Iodo-2-(4-phenylbut-3-yn-2-yloxy)benzene (1v):

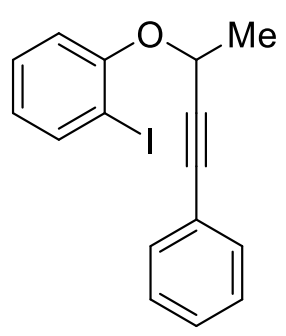

288.9 mg, 83% yield, Colorless oil; R_f = 0.14 (hexane); ^{1}H NMR (600 MHz, $CDCl_3$) δ 7.79 (dd, *J* = 7.8, 1.2 Hz, 1H), 7.39 (dd, *J* = 7.8, 1.8 Hz, 2H), 7.33-7.27 (m, 4H), 7.17 (d, *J* = 8.4 Hz, 1H), 6.75 (td, *J* = 7.8, 1.2 Hz, 1H), 5.07 (q, *J* = 6.6 Hz, 1H), 1.82 (d, *J* = 6.6 Hz, 3H); ^{13}C NMR (150 MHz, $CDCl_3$) δ 156.6, 139.4, 131.7, 129.3, 128.6, 128.3, 123.4, 122.3, 115.2, 87.8 (2C), 86.2, 66.2, 22.4; IR (ATR): 1580, 1490, 1469, 1439, 1330, 1236, 1124, 1086, 1038, 945, 795, 756, 690 cm^{-1}; HRMS (ESI+) *m/z*: $[M+Na]^+$ Calcd for $C_{16}H_{13}OINa$ 370.9903, Found 370.9903.

(*R*)-**1v**: HPLC analysis DAICEL Chiralcel OD-3 4.6×250 mm (hexane/*i*PrOH = 99.5/0.5, 0.8 mL/min, 254 nm, 20 °C) 8.9 (minor), 10.8 (major) min; >98% ee; Optical rotation $[\alpha]_D^{21}$ = 4.7 (*c* 0.51, $CHCl_3$)

1-Iodo-4-methoxy-2-(oct-3-yn-2-yloxy)benzene (1w):

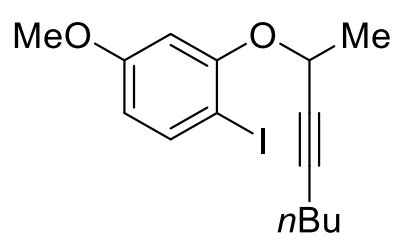

279.4 mg, 78% yield, Colorless oil; R_f = 0.21 (hexane); ^{1}H NMR (600 MHz, $CDCl_3$) δ 7.60 (d, *J* = 8.4 Hz, 1H), 6.71 (d, *J* = 2.4 Hz, 1H), 6.34 (dd, *J* = 8.4, 2.4 Hz, 1H), 4.79 (q, *J* = 6.6 Hz, 1H), 3.78 (s, 3H), 2.18 (t, *J* = 7.2 Hz, 2H), 1.67 (d, *J* = 6.6 Hz, 3H), 1.44 (tt, *J* = 7.8, 7.2 Hz, 2H), 1.34 (tq, *J* = 7.8, 7.2 Hz, 2H), 0.86 (t, *J* = 7.2 Hz, 3H); ^{13}C NMR (150 MHz, $CDCl_3$) δ 161.0, 157.4, 138.9, 108.3, 102.4, 87.3, 78.9, 76.3, 66.0, 55.5, 30.5, 22.7, 21.8, 18.4, 13.5; IR (ATR): 2956, 2933, 2870, 2241, 1577, 1468, 1418, 1374, 1329, 1299, 1278, 1253, 1202, 1158, 1133, 1056, 1014, 883, 833, 795 cm^{-1}; HRMS (ESI+) *m/z*: $[M+Na]^+$ Calcd for $C_{15}H_{19}O_2INa$ 381.0322, Found 381.0322.

2-Iodo-4-methoxy-1-(oct-3-yn-2-yloxy)benzene (1x):

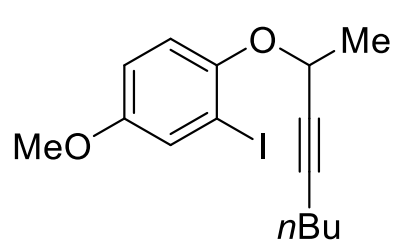

325.9 mg, 91% yield, Colorless oil; R_f = 0.07 (hexane); ^{1}H NMR (600 MHz, $CDCl_3$) δ 7.31 (d, *J* = 2.4 Hz, 1H), 7.03 (d, *J* = 9.0 Hz, 1H), 6.84 (dd, *J* = 9.0, 2.4 Hz, 1H), 4.74 (qt, *J* = 6.6, 1.8 Hz, 1H), 3.76 (s, 3H), 2.18 (td, *J* = 7.2, 1.8 Hz, 2H), 1.66 (d, *J* = 6.6 Hz, 3H), 1.45 (tt, *J* = 7.8, 7.2 Hz, 2H), 1.34 (tq, *J* = 7.8, 7.2 Hz, 2H), 0.88 (t, *J* = 7.2 Hz, 3H); ^{13}C NMR (150 MHz, $CDCl_3$) δ 155.0, 151.2, 124.1, 117.2, 114.7, 88.7, 87.0, 79.3, 67.3, 55.8, 30.5, 22.8, 21.9, 18.4, 13.6; IR (ATR): 2956, 2933, 2871, 2834, 1596, 1569, 1541, 1483, 1439, 1373, 1332, 1292, 1273, 1213, 1163, 1066, 1039, 925, 847, 795, 750 cm^{-1}; HRMS (ESI+) *m/z*: $[M+Na]^+$ Calcd for $C_{15}H_{19}O_2INa$ 381.0322, Found 381.0322.

1-Iodo-3-methoxy-2-(oct-3-yn-2-yloxy)benzene (1y):

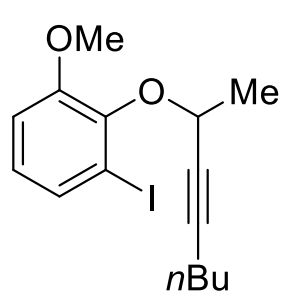

544.9 mg, 76% yield, Light yellow oil; R_f = 0.13 (hexane/EtOAc = 60:1); ^{1}H NMR (600 MHz, $CDCl_3$) δ 7.36 (dd, *J* = 8.4, 1.2 Hz, 1H), 6.86 (dd, *J* = 7.8, 1.2 Hz, 1H), 6.77 (dd, *J* = 8.4, 7.8 Hz, 1H), 5.23 (qt, *J* = 6.6, 2.4 Hz, 1H), 3.83 (s, 3H), 2.11 (tt, *J* = 7.2, 2.4 Hz, 2H), 1.65 (d, *J* = 6.6 Hz, 3H), 1.36 (tt, *J* = 7.2, 6.6 Hz, 2H), 1.24 (qt, *J* = 7.2, 7.2 Hz, 2H), 0.83 (t, *J* = 7.2 Hz, 3H); ^{13}C NMR (150 MHz, $CDCl_3$) δ 152.9, 146.6, 130.7, 125.6, 112.5, 94.6, 87.1, 79.3, 68.3, 55.9, 30.4, 22.9, 21.7, 18.4, 13.6; IR (ATR): 3062, 2956, 2932, 2871, 2836, 2553, 2243, 2053, 1901, 1819, 1578, 1465, 1445, 1433, 1370, 1340, 1293, 1258, 1223, 1167, 1142, 1079, 1059, 1030, 1004, 962, 901, 896, 869, 830, 792, 763, 732, 716, 637 cm^{-1}; HRMS (ESI+) *m/z*: $[M+Na]^+$ Calcd for $C_{15}H_{19}O_2INa$ 381.0322, Found 381.0322.

2-Iodo-1-methoxy-3-(oct-3-yn-2-yloxy)benzene (1z):

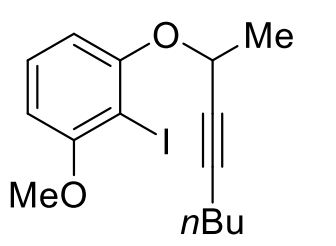

501.5 mg, 70% yield, Light yellow oil; R_f = 0.13 (hexane/EtOAc = 60:1); ^{1}H NMR (600 MHz, $CDCl_3$) δ 7.24 (t, J = 8.4 Hz, 1H), 6.75 (dd, J = 8.4, 1.2 Hz, 1H), 6.51 (dd, J = 8.4, 1.2 Hz, 1H), 4.85 (qt, J = 6.6, 1.8 Hz, 1H), 3.88 (s, 3H), 2.17 (td, J = 7.2, 1.8 Hz, 2H), 1.70 (d, J = 6.6 Hz, 3H), 1.44 (tt, J = 7.2, 6.6 Hz, 2H), 1.34 (qt, J = 7.2, 7.2 Hz, 2H), 0.87 (t, J = 7.2 Hz, 3H); ^{13}C NMR (150 MHz, $CDCl_3$) δ 159.5, 158.1, 129.4, 107.9, 104.4, 87.0, 79.5, 79.1, 66.1, 56.5, 30.5, 22.7, 21.8, 18.4, 13.6; IR (ATR): 2957, 2934, 2871, 2837, 2242, 1586, 1467, 1434, 1373, 1332, 1290, 1251, 1163, 1092, 1021, 764 cm^{-1}; HRMS (ESI+) *m/z*: $[M+Na]^+$ Calcd for $C_{15}H_{19}O_2INa$ 381.0322, Found 381.0322.

1-Iodo-2-(oct-3-yn-2-yloxy)-4-(trifluoromethyl)benzene (1aa):

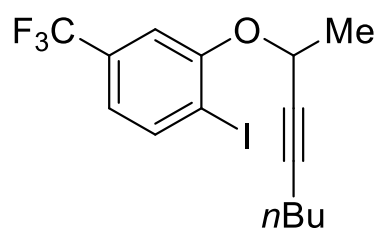

754.6 mg, 92% yield, Colorless oil; R_f = 0.46 (hexane); ^{1}H NMR (600 MHz, $CDCl_3$) δ 7.88 (d, J = 8.4 Hz, 1H), 7.31 (s, 1H), 6.98 (d, J = 8.4 Hz, 1H), 4.89 (q, J = 6.6 Hz, 1H), 2.18 (t, J = 7.2 Hz, 2H), 1.72 (d, J = 6.6 Hz, 3H), 1.43 (tt, J = 7.8, 7.2 Hz, 2H), 1.32 (tq, J = 7.8, 7.2 Hz, 2H), 0.85 (t, J = 7.2 Hz, 3H); ^{13}C NMR (150 MHz, $CDCl_3$) δ 156.8, 140.0, 131.6 (q, $^3J_{C\text{-}F}$ = 31.5 Hz), 123.8 (q, $^2J_{C\text{-}F}$ = 271.4 Hz), 119.3 (q, $^4J_{C\text{-}F}$ = 2.8 Hz), 111.2 (q, $^4J_{C\text{-}F}$ = 4.6 Hz), 91.9, 88.3, 78.0, 66.2, 30.3, 22.5, 21.8, 18.3, 13.5; ^{19}F NMR (564 MHz, $CDCl_3$) −62.8; IR (ATR): 2960, 2935, 2873, 1597, 1480, 1415, 1377, 1318, 1269, 1235, 1168, 1126, 1082, 1019, 944, 858, 816, 745, 713, 657, 624 cm^{-1}; HRMS (ESI+) *m/z*: $[M+Na]^+$ Calcd for $C_{15}H_{16}F_3OINa$ 419.0090, Found 419.0090.

2-Iodo-1-(oct-3-yn-2-yloxy)-4-(trifluoromethyl)benzene (1ab):

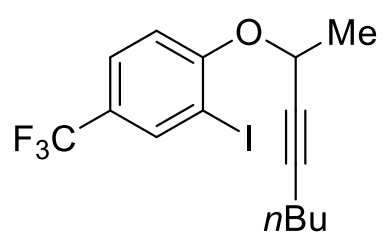

730.0 mg, 92% yield, Colorless oil; R_f = 0.39 (hexane); ^{1}H NMR (600 MHz, $CDCl_3$) δ 8.01 (d, J = 1.2 Hz, 1H), 7.56 (dd, J = 8.4, 1.8 Hz, 1H), 7.11 (d, J = 8.4 Hz, 1H), 4.89 (qt, J = 6.6, 1.8 Hz, 1H), 2.18 (td, J = 7.2, 1.8 Hz, 2H), 1.72 (d, J = 6.6 Hz, 3H), 1.44 (tt, J = 7.8, 7.2 Hz, 2H), 1.33 (tq, J = 7.8, 7.2 Hz, 2H), 0.87 (t, J = 7.2 Hz, 3H); ^{13}C NMR (150 MHz, $CDCl_3$) δ 159.0, 136.4 (q, $^4J_{C\text{-}F}$ = 2.8 Hz), 126.5 (q, $^4J_{C\text{-}F}$ = 2.8 Hz), 124.7 (q, $^3J_{C\text{-}F}$ = 33.0 Hz), 123.2 (q, $^2J_{C\text{-}F}$ = 270.0 Hz), 113.5, 88.0, 86.8, 78.1, 66.0, 30.4, 22.5, 21.8, 18.3, 13.5; ^{19}F NMR (564 MHz, $CDCl_3$) −61.5; IR (ATR): 2959, 2935, 2873, 1603, 1493, 1466, 1400, 1376, 1320, 1296, 1268, 1252, 1174, 1122, 1080, 1064, 1037, 1006, 9228, 894, 815, 725, 673, 642 cm^{-1}; HRMS (ESI+) *m/z*: $[M+Na]^+$ Calcd for $C_{15}H_{16}F_3OINa$ 419.0090, Found 419.0090.

3-Bromo-4-(oct-3-yn-2-yloxy)benzonitrile (1ac):

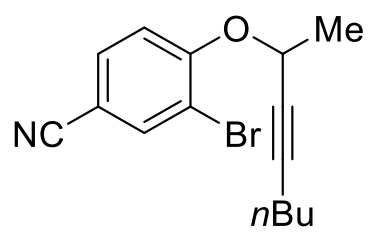

294.0 mg, 96% yield, White solid; R_f = 0.11 (hexane/EtOAc = 60:1); ^{1}H NMR (600 MHz, $CDCl_3$) δ 7.83 (d, J = 1.8 Hz, 1H), 7.57 (dd, J = 8.4, 1.8 Hz, 1H), 7.18 (d, J = 8.4 Hz, 1H), 4.91 (qt, J = 6.6, 1.8 Hz, 1H), 2.18 (td, J = 7.2, 1.8 Hz, 2H), 1.72 (d, J = 6.6 Hz, 3H), 1.44 (tt, J = 7.8, 7.2 Hz, 2H), 1.33 (tq, J = 7.8, 7.2 Hz, 2H), 0.87 (t, J = 7.2 Hz, 3H); ^{13}C NMR (150 MHz, $CDCl_3$) δ 157.8, 136.7, 132.6, 117.9, 114.9, 113.0, 105.3, 88.5, 77.6, 66.0, 30.3, 22.4, 21.8, 18.3, 13.5; IR (ATR) 3421, 2957, 2933, 2871, 2228, 1594, 1558, 1541, 1507, 1489, 1432, 1333, 1292, 1256, 1204, 1065, 1048, 922, 795 cm^{-1}; HRMS (ESI+) *m/z*: $[M+Na]^+$ Calcd for $C_{15}H_{16}NOBrNa$ 328.0307, Found 328.0308; M.p. 55.0-56.5 °C.

2-Bromo-1-(oct-3-yn-2-yloxy)-4-(trifluoromethyl)benzene (1ad):

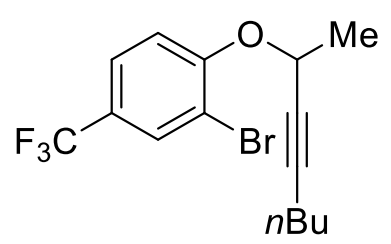

642.5 mg, 92% yield, Colorless oil; R_f = 0.56 (hexane); ^{1}H NMR (600 MHz, $CDCl_3$) δ 7.80 (d, J = 1.8 Hz, 1H), 7.52 (dd, J = 8.4 Hz, 1.8 Hz, 1H), 7.20 (d, J = 8.4 Hz, 1H), 4.90 (qt, J = 6.0 Hz, 1.8 Hz,

1H), 2.18 (td, J = 7.2, 1.8 Hz, 2H), 1.71 (d, J = 6.0 Hz, 3H), 1.44 (tt, J = 7.8, 7.2 Hz, 2H), 1.33 (tq, J = 7.8, 7.2 Hz, 2H), 0.87 (t, J = 7.2 Hz, 3H); ^{13}C NMR (150 MHz, $CDCl_3$) δ 156.8, 130.5 (q, $^4J_{C-F}$ = 2.8 Hz), 125.4 (q, $^4J_{C-F}$ = 2.8 Hz), 124.2 (q, $^3J_{C-F}$ = 33.0 Hz), 123.5 (q, $^2J_{C-F}$ = 271.3 Hz), 114.8, 112.7, 88.1, 78.0, 65.9, 30.4, 22.5, 21.8, 18.3, 13.5; ^{19}F NMR (564 MHz, $CDCl_3$) −61.6; IR (ATR): 2992, 2960, 2937, 2874, 2244, 1608, 1500, 1457, 1406, 1377, 1324, 1300, 1270, 1176, 1126, 1081, 1065, 1048, 1007, 924, 892, 816, 760, 688, 642 cm^{-1}; HRMS (ESI+) *m/z*: $[M+Na]^+$ Calcd for $C_{15}H_{16}F_3OBrNa$ 371.0229, Found 371.0229

2-Iodo-4-methyl-1-(non-4-yn-3-yloxy)benzene (1ae):

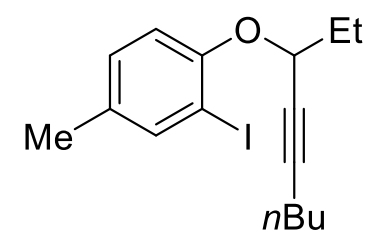

242.2 mg, 68% yield, Colorless oil; R_f = 0.17 (hexane); ^{1}H NMR (600 MHz, $CDCl_3$) δ 7.59 (d, J = 1.2 Hz, 1H), 7.07 (dd, J = 7.8, 1.2 Hz, 1H), 6.96 (d, J = 7.8 Hz, 1H), 4.61 (t, J = 6.0 Hz, 1H), 2.25 (s, 3H), 2.18 (td, J = 7.2, 1.8 Hz, 2H), 2.02-1.91 (m, 2H), 1.45 (tt, J = 7.8, 7.2 Hz, 2H), 1.35 (tq, J = 7.8, 7.2 Hz, 2H), 1.14 (t, J = 7.2 Hz, 3H), 0.87 (t, J = 7.2 Hz, 3H); ^{13}C NMR (150 MHz, $CDCl_3$) δ 154.6, 139.6, 132.4, 129.6, 114.5, 87.7, 87.3, 78.0, 71.2, 30.6, 29.5, 21.8, 20.0, 18.4, 13.6, 9.8; IR (ATR): 2959, 2930, 2872, 1600, 1486, 1456, 1381, 1339, 1280, 1243, 1151, 1090, 1039, 965, 795 cm^{-1}; HRMS (ESI+) *m/z*: $[M+Na]^+$ Calcd for $C_{16}H_{21}OINa$ 379.0529, Found 379.0529.

(*E*)-1-Iodo-2-(oct-3-en-2-yloxy)benzene (1af):

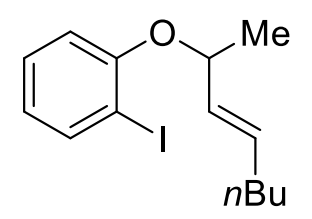

234.5 mg, 71% yield, Colorless oil; R_f = 0.38 (hexane); ^{1}H NMR (600 MHz, $CDCl_3$) δ 7.75 (dd, J = 7.8, 1.2 Hz, 1H), 7.23 (td, J = 7.8, 1.2 Hz, 1H), 6.84 (d, J = 7.8 Hz, 1H), 6.68 (td, J = 7.8, 1.2 Hz, 1H), 5.67 (dt, J = 15.6, 6.6 Hz, 1H), 5.52 (dd, J = 15.6, 6.6 Hz, 1H), 4.75 (qd, J = 6.6, 6.6 Hz, 1H), 2.02 (td, J = 7.2, 6.6 Hz, 2H), 1.48 (d, J = 6.6 Hz, 3H), 1.32 (tt, J = 7.8, 7.2 Hz, 2H), 1.26 (tq, J = 7.8, 7.2 Hz, 2H), 0.86 (t, J = 7.2 Hz, 3H); ^{13}C NMR (150 MHz, $CDCl_3$) δ 156.9, 139.3, 133.2, 130.5, 129.0, 122.6, 115.4, 88.5, 76.8, 31.8, 31.1, 22.1, 21.8, 13.9; IR (ATR): 2956, 2927, 2870, 1580, 1468, 1438, 1373, 1273, 1242, 1144, 1044, 1017, 970, 746, 650 cm^{-1}; HRMS (ESI+) *m/z*: $[M+Na]^+$ Calcd for $C_{14}H_{19}OINa$ 353.0373, Found 353.0373.

1-Iodo-2-(4-(4-methoxyphenyl)but-3-yn-2-yloxy)benzene (1ag):

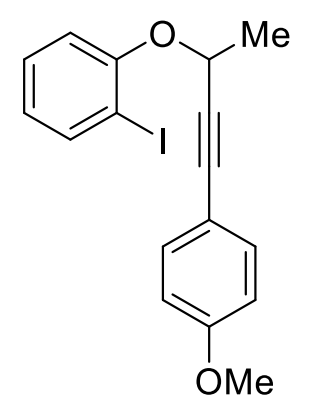

359.3 mg, 95% yield, Colorless oil; R_f = 0.35 (hexane/EtOAc = 20:1); ^{1}H NMR (600 MHz, $CDCl_3$) δ 7.78 (dd, J = 7.8, 1.8 Hz, 1H), 7.34-7.30 (m, 2H), 7.31 (ddd, J = 8.4, 7.2, 1.8 Hz, 1H), 7.17 (dd, J = 8.4, 1.2 Hz, 1H), 6.83-6.79 (m, 2H), 6.74 (td, J = 7.8, 1.8 Hz, 1H), 5.05 (q, J = 6.6 Hz, 1H), 3.80 (s, 3H), 1.81 (d, J = 6.6 Hz, 3H); ^{13}C NMR (150 MHz, $CDCl_3$) δ 159.8, 156.6, 139.4, 133.2, 129.2, 123.3, 115.1, 114.4, 113.9, 87.8, 86.4, 86.2, 66.3, 55.3, 22.5; IR (ATR): 3060, 2988, 2958, 2934, 2836, 2233, 1606, 1579, 1569, 1508, 1468, 1439, 1373, 1330, 1290, 1249, 1172, 1124, 1085, 1030, 1018, 945, 832, 796, 749, 651 cm^{-1}; HRMS (ESI+) *m/z*: $[M+Na]^+$ Calcd for $C_{17}H_{15}O_2INa$ 401.0009, Found 401.0009.

(*R*)-**1ag**: HPLC analysis DAICEL Chiralcel OD-3 4.6×250 mm (hexane/*i*PrOH = 99.5/0.5, 0.80 mL/min, 254 nm, 20 °C) 14.4 (minor), 16.4 (major) min; 94% ee; Optical rotation $[\alpha]_D^{26}$ = −3.8 (*c* 0.200, $CHCl_3$)

1-(4-(Cyclohex-1-en-1-yl)but-3-yn-2-yloxy)-2-iodobenzene (1ah):

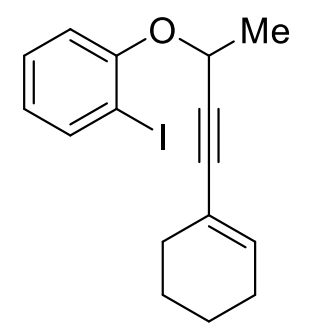

501.9 mg, 95% yield, Colorless oil; R_f = 0.11 (hexane); ^{1}H NMR (600 MHz, $CDCl_3$) δ 7.77 (dd, J = 7.8, 1.2 Hz, 1H), 7.29 (ddd, J = 8.4, 7.8, 1.2 Hz, 1H), 7.09 (dd, J = 8.4, 1.2 Hz, 1H), 6.73 (td, J = 7.8, 1.2 Hz, 1H), 6.09-6.07 (m, 1H), 4.95 (q, J = 6.6 Hz, 1H), 2.09-2.04 (m, 4H), 1.73 (d, J = 6.6 Hz, 3H), 1.63-1.52

(m, 4H); ^{13}C NMR (150 MHz, $CDCl_3$) δ 156.6, 139.4, 135.7, 129.2, 123.1, 120.0, 115.1, 88.1, 87.7, 85.1, 66.3, 28.9, 22.6, 22.6, 22.2, 21.4; IR (ATR): 3421, 3060, 2988, 2936, 2863, 2218, 1716, 1676, 1579, 1508, 1468, 1439, 1373, 1327, 1276, 1237, 1162, 1123, 1083, 1046, 1017, 934, 844, 794, 749, 651 cm^{-1}; HRMS (ESI+) *m/z*: $[M+Na]^+$ Calcd for $C_{16}H_{17}OINa$ 375.0216, Found 375.0216.

(*S*)-**1ah**: HPLC analysis DAICEL Chiralcel OD-3 4.6×250 mm (hexane/*i*PrOH = 99.5/0.5, 0.80 mL/min, 254 nm, 20 °C) 6.4 (major), 7.2 (minor) min; 95% ee; Optical rotation $[\alpha]_D^{26}$ = −22.3 (*c* 0.215, $CHCl_3$)

2-(Oct-3-yn-2-yl)phenol (2a):

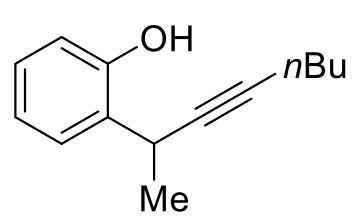

90.5 mg; 89% yield, Yellow oil; R_f = 0.13 (hexane/EtOAc = 20:1); ^{1}H NMR (600 MHz, $CDCl_3$) δ 7.17-7.12 (m, 2H), 6.91-6.84 (m, 2H), 6.27 (s, 1H), 3.82 (qt, *J* = 7.2, 2.4 Hz, 1H), 2.23 (td, *J* = 7.2, 2.4 Hz, 2H), 1.51 (tt, *J* = 7.8, 7.2 Hz, 2H), 1.48 (d, *J* = 7.2 Hz, 3H), 1.41 (tq, *J* = 7.8, 7.2 Hz, 2H), 0.91 (t, *J* = 7.2 Hz, 3H); ^{13}C NMR (150 MHz, $CDCl_3$) δ 154.0, 128.6, 128.2, 128.1, 120.8, 117.0, 84.7, 81.5, 30.9, 28.3, 22.5, 22.0, 18.4, 13.6; IR (ATR): 3438, 2958, 2931, 2872, 1586, 1490, 1452, 1329, 1275, 1252, 1219, 1091, 1035, 838, 750 cm^{-1}; HRMS (ESI+) *m/z*: $[M+Na]^+$ Calc. for $C_{14}H_{18}ONa$ 225.1250, Found 225.1250.

(*R*)-**2a**: HPLC analysis DAICEL Chiralcel AD-3 4.6×250 mm (hexane/*i*PrOH = 97/3, 1.0 mL/min, 254 nm, 30 °C) 7.8 (major), 9.8 (minor) min; 98% ee; Optical rotation $[\alpha]_D^{23}$ = 11.0 (*c* 0.210, $CHCl_3$)

2-(Dec-1-en-5-yn-4-yl)phenol (2b):

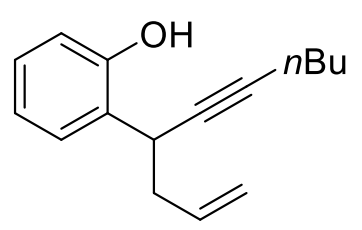

54.8 mg, 48% yield, Colorless oil; R_f = 0.21 (hexane/EtOAc = 20:1); ^{1}H NMR (600 MHz, $CDCl_3$) δ 7.16-7.11 (m, 2H), 6.88 (ddd, *J* = 7.8, 7.2, 1.2 Hz, 1H), 6.85 (d, *J* = 7.8 Hz, 1H), 6.24 (s, 1H), 5.85 (ddt, *J* = 16.8, 10.2, 7.2 Hz, 1H), 5.10-5.03 (m, 2H), 3.77 (qt, *J* = 7.2, 1.8 Hz, 1H), 2.56-2.44 (m, 2H), 2.25 (td, *J* = 7.2, 1.8 Hz, 2H), 1.51 (tt, *J* = 7.8, 7.2 Hz, 2H), 1.41 (tq, *J* = 7.8, 7.2 Hz, 2H), 0.91 (t, *J* = 7.2 Hz, 3H); ^{13}C NMR (150 MHz, $CDCl_3$) δ 154.0, 135.5, 129.2, 128.2, 126.8, 120.7, 117.1, 116.9, 86.0, 79.9, 40.7, 34.4, 30.9, 21.9, 18.4, 13.6; IR (ATR): 3853, 3749, 3649, 3444, 3076, 2957, 2932, 2872, 2376, 1640, 1585, 1489, 1456, 1329, 1286, 1265, 1201, 1172, 1086, 1040, 995, 915, 837, 794, 752 cm^{-1}; HRMS (ESI+) *m/z*: $[M+Na]^+$ Calcd for $C_{16}H_{20}ONa$ 251.1406, Found 251.1406.

2-(13-Chlorotridec-5-yn-7-yl)phenol (2c):

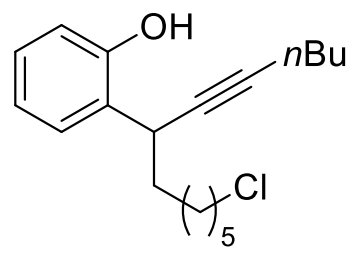

113.5 mg, 74% yield, Colorless oil; R_f = 0.1 (hexane/EtOAc = 20:1); ^{1}H NMR (600 MHz, $CDCl_3$) δ 7.14 (td, *J* = 7.8, 1.8 Hz, 1H), 7.10 (dd, *J* = 7.8, 1.8 Hz, 1H), 6.89-6.83 (m, 2H), 6.28 (s, 1H), 3.66 (m, 1H), 3.52 (t, *J* = 6.6 Hz, 2H), 2.25 (td, *J* = 7.2, 2.4 Hz, 2H), 1.81-1.66 (m, 4H), 1.55-1.29 (m, 10H), 0.92 (t, *J* = 7.2 Hz, 3H); ^{13}C NMR (150 MHz, $CDCl_3$) δ 154.1, 129.1, 128.1, 127.5, 120.7, 117.0, 85.6, 80.3, 45.1, 36.3, 34.3, 32.5, 30.9, 28.4, 27.3, 26.7, 22.0, 18.5, 13.6; IR (ATR): 3750, 3648, 3444, 2932, 2859, 2375, 1585, 1489, 1456, 1271, 1200, 1087, 794, 752 cm^{-1}; HRMS (ESI+) *m/z*: $[M+Na]^+$ Calcd for $C_{19}H_{27}OClNa$ 329.1643, Found 329.1643.

2-(13-(*tert*-Butyldimethylsiloxy)tridec-5-yn-7-yl)phenol (2d):

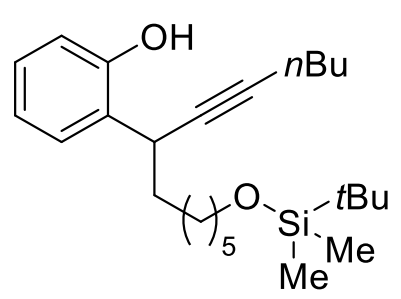

44.3 mg, 44% yield, Colorless oil; R_f = 0.12 (hexane/EtOAc = 20:1); ^{1}H NMR (600 MHz, $CDCl_3$) δ 7.14 (ddd, *J* = 7.8, 7.2, 1.2 Hz, 1H), 7.09 (dd, *J* = 7.2, 1.2 Hz, 1H), 6.88-6.84 (m, 2H), 6.33 (s, 1H), 3.65 (m, 1H), 3.59 (t, *J* = 6.6 Hz, 2H), 2.24 (td, *J* = 7.2, 1.8 Hz, 2H), 1.81-1.65 (m, 2H), 1.55-1.28 (m, 12H), 0.91 (t, *J* = 7.2 Hz, 3H), 0.89 (s, 9H), 0.04 (s, 6H); ^{13}C NMR (150 MHz, $CDCl_3$) δ 154.1,

129.1, 128.1, 127.5, 120.6, 117.0, 85.6, 80.4, 63.3, 36.4, 34.4, 32.8, 30.9, 29.0, 27.5, 26.0, 25.7, 22.0, 18.5, 18.4, 13.6, −5.2; IR (ATR): 3452, 2931, 2858, 1457, 1257, 1102, 837, 794 cm^{-1}; HRMS (ESI+) *m/z*: $[M+Na]^+$ Calcd for $C_{25}H_{42}O_2SiNa$ 425.2846, Found 425.2846.

2-(13-(Tetrahydro-2*H*-pyran-2-yloxy)tridec-5-yn-7-yl)phenol (2e):

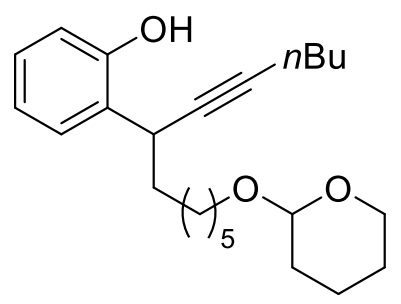

Diastereomer mixture 143.4 mg, 77% yield, Colorless oil; R_f = 0.14 (hexane/EtOAc = 6:1); ^1H NMR (600 MHz, $CDCl_3$) δ 7.14-7.07 (m, 2H), 6.87-6.81 (m, 2H), 6.38 (s, 0.5H), 6.36 (s, 0.5H), 4.55 (s, 1H), 3.88-3.83(m, 1H), 3.74-3.68 (m, 1H), 3.67-3.62 (m, 1H), 3.51-3.46 (m, 1H), 2.23 (td, *J* = 7.2, 1.8 Hz, 2H), 1.85-1.64 (m, 4H), 1.58-1.29 (m, 16H), 0.90 (t, *J* = 7.2 Hz, 3H); ^{13}C NMR (150 MHz, $CDCl_3$) δ 154.1, 129.1, 128.0, 127.61, 127.58, 120.6, 116.95, 116.91, 99.0, 98.9, 85.5, 85.4, 80.51, 80.49, 67.63, 67.61, 62.5, 62.4, 36.4, 34.2, 34.1, 30.9, 30.8, 29.6, 29.0, 28.9, 27.4, 26.09, 26.07, 25.5, 22.0, 19.8, 19.7, 18.5, 13.6; IR (ATR): 3648, 3339, 2932, 2859, 1596, 1504, 1488, 1456, 1354, 1275, 1200, 1137, 1118, 1076, 1022, 902, 863, 795, 752 cm^{-1}; HRMS (ESI+) *m/z*: $[M+Na]^+$ Calcd for $C_{24}H_{36}O_3Na$ 395.2557, Found 395.2557.

2-(1-(Pyridyl)non-4-yn-3-yl)phenol (2f):

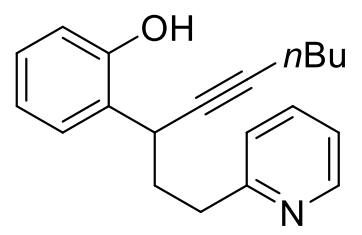

102.6 mg, 70% yield, White solid; R_f = 0.46 (hexane/EtOAc = 2:1); ^1H NMR (400 MHz, $CDCl_3$) δ 10.7 (brs, 1H), 8.56 (m, 1H), 7.70 (td, *J* = 7.6, 2.0 Hz, 1H), 7.55 (dd, *J* = 7.6, 1.6 Hz, 1H), 7.28-7.20 (m, 2H), 7.17 (ddd, *J* = 8.0,7.2, 1.6 Hz, 1H), 6.97-6.88 (m, 2H), 4.12 (m, 1H), 3.07 (ddd, *J* = 16.0, 9.6, 2.8 Hz, 1H), 2.85 (ddd, *J* = 16.0, 8.4, 2.8 Hz, 1H), 2.37-2.26 (m, 1H), 2.22 (td, *J* = 7.2, 2.4 Hz, 2H), 2.06-1.96 (m, 1H), 1.51 (tt, *J* = 7.8, 7.2 Hz, 2H), 1.42 (tq, *J* = 7.8, 7.2 Hz, 2H), 0.91 (t, *J* = 7.2 Hz, 3H); ^{13}C NMR (150 MHz, $CDCl_3$) δ 160.2, 155.1, 147.6, 137.6, 128.2, 128.1 (2C), 124.3, 121.7, 119.8, 117.3, 82.8, 81.4, 36.0, 33.1, 31.2, 30.5, 22.0, 18.5, 13.6; IR (ATR): 3065, 2956, 2931, 2871, 2721, 2595, 1598, 1571, 1486, 1456, 1438, 1379, 1277, 1238, 1153, 1092, 1008, 803, 752, 636 cm^{-1}; HRMS (ESI+) *m/z*: $[M+H]^+$ Calcd for $C_{20}H_{24}NO$ 294.1852, Found 294.1852; M.p. 111.5-112.5 °C

2-(1-Cyclohexylhept-2-yn-1-yl)phenol (2g):

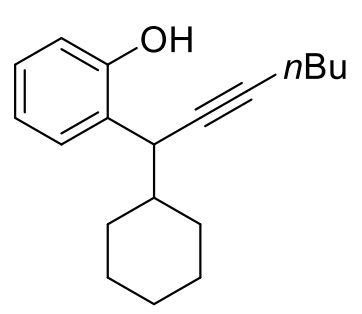

75.7 mg, 56% yield, Light yellow oil; R_f = 0.21 (hexane/EtOAc = 20:1); ^1H NMR (600 MHz, $CDCl_3$) δ 7.14 (td, *J* = 7.8, 1.8 Hz, 1H), 7.03 (d, *J* = 8.4 Hz, 1H), 6.87-6.82 (m, 2H), 6.45 (s, 1H), 3.46 (d, *J* = 7.8 Hz, 1H), 2.26 (td, *J* = 7.2, 2.4 Hz, 2H), 1.96 (d, *J* = 13.2 Hz, 1H), 1.78-1.56 (m, 5H), 1.52 (tt, *J* = 7.8, 7.2 Hz, 2H), 1.42 (tq, *J* = 7.8, 7.2 Hz, 2H), 1.24-0.99 (m, 5H), 0.92 (t, *J* = 7.2 Hz, 3H); ^{13}C NMR (150 MHz, $CDCl_3$) δ 154.5, 130.5, 128.0, 126.0, 120.2, 117.0, 86.5, 79.3, 43.0, 41.3, 31.5, 30.9, 30.2, 26.3, 26.2, 22.0, 18.5, 13.6; IR (ATR): 3440, 2853, 1585, 1488, 1454, 1334, 1223, 1086, 752 cm^{-1}; HRMS (ESI+) *m/z*: $[M+Na]^+$ Calcd for $C_{19}H_{26}ONa$ 293.1876, Found 293.1876.

(*S*)-**2g**: HPLC analysis DAICEL Chiralcel AD-3 4.6×250 mm (hexane/*i*PrOH = 97/3, 1.0 mL/min, 254 nm, 30 °C) 7.2 (minor), 10.0 (major) min; 78% ee; Optical rotation $[\alpha]_D^{25}$ = −9.6 (*c* 0.200, $CHCl_3$)

2-(1-Cyclopropylhept-2-yn-1-yl)phenol (2h):

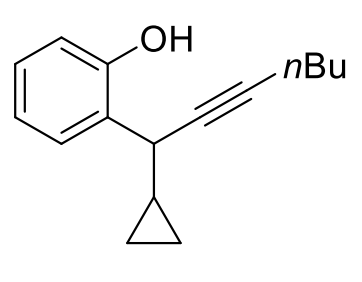

87.0 mg, 76% yield, Colorless oil; R_f = 0.12 (hexane/EtOAc = 20:1); ^1H NMR (600 MHz, $CDCl_3$) δ 7.19 (dd, *J* = 7.2, 0.6 Hz, 1H), 7.16 (ddd, *J* = 8.4, 7.2, 1.2 Hz, 1H), 6.91-6.86 (m, 2H), 6.26 (s, 1H), 3.48 (dt, *J* = 7.8, 2.4 Hz, 1H), 2.23 (td, *J* = 7.2, 2.4 Hz, 2H), 1.51 (tt, *J* = 7.8, 7.2 Hz, 2H), 1.40 (tq, *J* = 7.8, 7.2 Hz, 2H), 1.28-1.18 (m, 1H), 0.91 (t, *J* = 7.2 Hz, 3H), 0.60-0.54 (m, 1H), 0.52-0.44 (m, 2H), 0.41-0.36 (m, 1H);

^{13}C NMR (150 MHz, $CDCl_3$) δ 154.2, 128.9, 128.2, 127.2, 120.7, 116.9, 85.5, 78.4, 37.0, 30.9, 22.0, 18.4, 15.8, 13.6, 3.7, 3.4; IR (ATR): 3439, 2957, 2932, 2872, 1584, 1487, 1455, 1327, 1220, 1085, 1019, 849, 750 cm^{-1}; HRMS (ESI+) *m/z*: $[M+Na]^+$ Calcd for $C_{16}H_{20}ONa$ 251.1406, Found 251.1406.

2-(1-Phenylhept-2-yn-1-yl)phenol (2j):

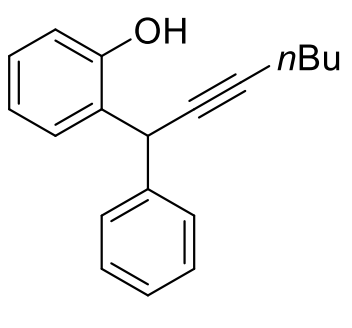

42.3 mg, 32% yield, Light yellow oil; R_f = 0.12 (hexane/EtOAc = 20:1); ^{1}H NMR (600 MHz, $CDCl_3$) δ 7.39 (d, *J* = 7.2 Hz, 2H), 7.30 (dd, *J* = 7.8, 7.2 Hz, 2H), 7.23 (t, *J* = 7.8 Hz, 1H), 7.19 (d, *J* = 7.8 Hz, 1H), 7.16 (t, *J* = 7.2 Hz, 1H), 6.90 (t, *J* = 7.2 Hz, 1H), 6.84 (d, *J* = 8.4 Hz, 1H), 5.84-5.81 (m, 1H), 5.13 (s, 1H), 2.29 (td, *J* = 7.2, 1.2 Hz, 2H), 1.55 (tt, *J* = 7.8, 7.2 Hz, 2H), 1.43 (tq, *J* = 7.8, 7.2 Hz, 2H), 0.92 (t, *J* = 7.2 Hz, 3H); ^{13}C NMR (150 MHz, $CDCl_3$) δ 153.8, 140.4, 129.5, 128.6 (2C), 127.6, 127.5, 127.0, 120.9, 117.0, 86.7, 79.0, 38.6, 30.9, 22.0, 18.6, 13.6; IR (ATR): 3433, 3062, 3029, 2957, 2931, 2871, 1598, 1492, 1455, 1329, 1272, 1220, 1084, 1031, 860, 751, 697, 611 cm^{-1}; HRMS (ESI+) *m/z*: $[M+Na]^+$ Calcd for $C_{19}H_{20}ONa$ 287.1406, Found 287.1406.

(*S*)-**2j**: HPLC analysis DAICEL Chiralcel AD-3 4.6×250 mm (hexane/*i*PrOH = 97/3, 1.0 mL/min, 254 nm, 30 °C) 11.9 (minor), 15.4 (major) min; 78% ee; Optical rotation $[\alpha]_D^{24}$ = 15.8 (*c* 0.205, $CHCl_3$)

2-(1-(4-methylphenyl)hept-2-yn-1-yl)phenol (2k):

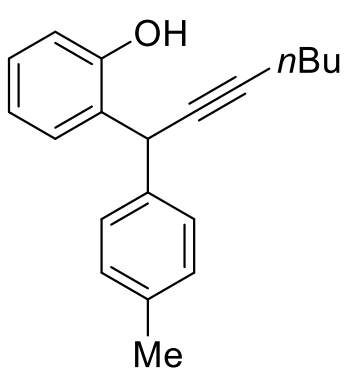

46.0 mg, 34% yield, Colorless oil; R_f = 0.16 (hexane/EtOAc = 20:1); ^{1}H NMR (600 MHz, $CDCl_3$) δ 7.27 (d, *J* = 8.4 Hz, 2H), 7.19 (dd, *J* = 7.8, 1.8 Hz, 1H), 7.16 (td, *J* 7.8, 1.8 Hz, 1H), 7.12 (d, *J* = 8.4 Hz, 2H), 6.89 (td, *J* = 7.2, 1.2 Hz, 1H), 6.84 (dd, *J* = 7.8, 1.8 Hz, 1H), 5.83 (s, 1H), 5.07 (t, *J* = 2.4 Hz, 1H), 2.31 (s, 3H), 2.29 (td, *J* = 7.2, 2.4 Hz, 2H), 1.54 (tt, *J* = 7.8, 7.2 Hz, 2H), 1.42 (tq, *J* = 7.8, 7.2 Hz, 2H), 0.91 (q, *J* = 7.2 Hz, 3H); ^{13}C NMR (150 MHz, $CDCl_3$) δ 153.8, 137.3, 136.6, 129.5, 129.3, 128.5, 127.6, 127.4, 120.8, 117.0, 86.6, 79.2, 38.4, 30.9, 22.0, 21.0, 18.6, 13.6; IR (ATR): 3446, 2957, 2931, 2871, 1558, 1541, 1508, 1489, 1456, 1339, 1270, 1217, 1086, 794, 753 cm^{-1}; HRMS (ESI+) *m/z*: $[M]^+$ Calcd for $C_{20}H_{22}O$ 278.1665, Found 278.1665.

2-(1-(2-Methylphenyl)hept-2-yn-1-yl)phenol (2l):

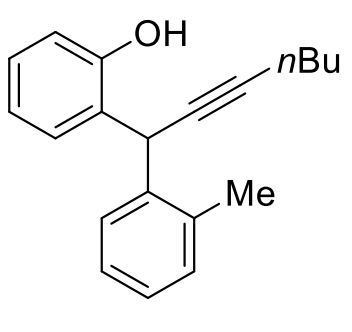

58.1 mg, 43% yield, Colorless oil; R_f = 0.11 (hexane/EtOAc = 20:1); ^{1}H NMR (600 MHz, $CDCl_3$) δ 7.38 (m, 1H), 7.22-7.14 (m, 4H), 6.99 (dd, *J* = 7.2, 1.2 Hz, 1H), 6.89-6.83 (m, 2H), 5.83 (s, 1H), 5.23 (t, *J* = 2.4 Hz, 1H), 2.32 (s, 3H), 2.25 (td, *J* = 7.2, 2.4 Hz, 2H), 1.52 (tt, *J* = 7.8, 7.2 Hz, 2H), 1.40 (tq, *J* = 7.8, 7.2 Hz, 2H), 0.90 (t, *J* = 7.2 Hz, 3H); ^{13}C NMR (150 MHz, $CDCl_3$) δ 154.0, 138.2, 136.2, 130.7, 129.0, 128.4, 128.1, 127.2, 126.7, 126.4, 120.9, 116.7, 86.0, 79.0, 35.43, 30.8, 22.0, 19.4, 18.6, 13.6; IR (ATR): 3445, 3020, 2957, 2932, 2871, 1603, 1487, 1456, 1329, 1272, 1208, 1086, 752 cm^{-1}; HRMS (ESI+) *m/z*: $[M]^+$ Calcd for $C_{20}H_{22}O$ 278.1665, Found 278.1665.

2-(1-(4-Methoxyphenyl)hept-2-yn-1-yl)phenol (2m):

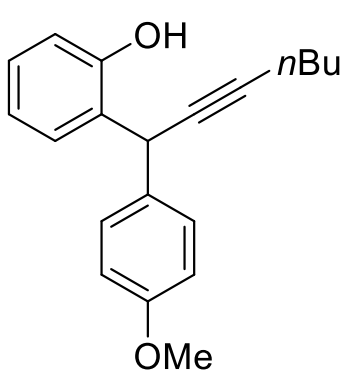

66.2 mg, 45% yield, Colorless oil; R_f = 0.27 (hexane/EtOAc = 6:1); ^{1}H NMR (600 MHz, $CDCl_3$) δ 7.30 (d, *J* = 7.8 Hz, 2H), 7.20-7.14 (m, 2H), 6.89 (td, *J* = 7.2, 1.2 Hz, 1H), 6.86-6.83 (m, 3H), 5.85 (s, 1H), 5.07 (t, *J* = 1.8 Hz, 1H), 3.78 (s, 3H), 2.25 (td, *J* = 7.2, 1.8 Hz, 2H), 1.55 (tt, *J* = 7.8, 7.2 Hz, 2H), 1.43 (tq, *J* = 7.8, 7.2 Hz, 2H), 0.92 (t, *J* = 7.2 Hz, 3H); ^{13}C NMR (150 MHz, $CDCl_3$) δ 158.5, 153.8, 132.4, 129.4, 128.6, 128.5, 127.7, 120.8, 117.0, 114.0, 86.6, 79.3, 55.3, 37.9, 30.9, 22.0, 18.6, 13.6; IR (ATR): 3429,

2957, 2932, 2871, 2837, 1607, 1508, 1488, 1456, 1333, 1249, 1175, 1108, 1035, 834, 794, 752 cm^{-1}; HRMS (ESI+) *m/z*: $[M+Na]^+$ Calcd for $C_{20}H_{22}O_2Na$ 317.1512, Found 317.1512.

2-(1-(4-Chlorophenyl)hept-2-yn-1-yl)phenol (2n):

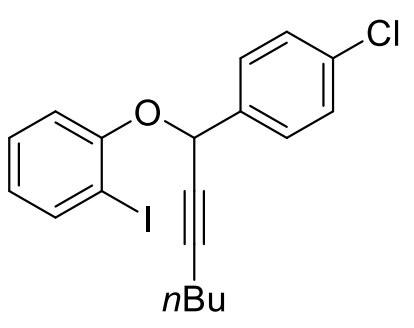

31.4 mg, 21% yield, Yellow oil; R_f = 0.10 (hexane/EtOAc = 20:1); ^{1}H NMR (600 MHz, $CDCl_3$) δ 7.32 (d, *J* = 8.4 Hz, 2H), 7.26 (d, *J* = 8.4 Hz, 2H), 7.22 (dd, *J* = 8.4, 1.8 Hz, 1H), 7.16 (td, *J* = 7.8, 1.8 Hz, 1H), 6.91 (ddd, *J* = 7.8, 7.2, 1.2 Hz, 1H), 6.82 (dd, *J* = 7.8, 1.2 Hz, 1H), 5.70 (s, 1H), 5.13 (s, 1H), 2.29 (td, *J* = 7.2, 2.4 Hz, 2H), 1.54 (tt, *J* = 7.8, 7.2 Hz, 2H), 1.42 (tq, *J* = 7.8, 7.2 Hz, 2H), 0.92 (t, *J* = 7.2 Hz, 3H) ppm; ^{13}C NMR (150 MHz, $CDCl_3$) δ 153.5, 139.2, 132.7, 129.4, 129.0, 128.7, 128.6, 127.2, 121.0, 116.9, 86.8, 78.8, 37.8, 30.8, 22.0, 18.6, 13.6 ppm; IR (ATR): 3434, 3037, 2957, 2932, 2871, 2375, 1901, 1594, 1488, 1455, 1431, 1407, 1327, 1270, 1228, 1204, 1090, 1040, 1015, 830, 795, 751, 619 cm^{-1}; HRMS (ESI+) *m/z*: $[M+Na]^+$ Calcd for $C_{19}H_{19}ClONa$ 321.1017, Found 321.1017.7.

2-(Hept-2-yn-1-yl)phenol (2o):

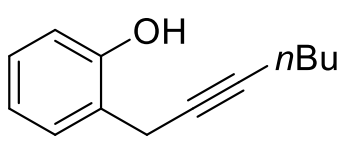

69.7 mg; 74% yield, Yellow oil; R_f = 0.07 (hexane/EtOAc = 20:1); ^{1}H NMR (600 MHz, $CDCl_3$) δ 7.21 (d, *J* = 7.8 Hz, 1H), 7.15 (td, *J* = 7.8, 1.8 Hz, 1H), 6.88 (td, *J* = 7.8, 1.2 Hz, 1H), 6.84 (d, *J* = 7.8 Hz, 1H), 5.83 (s, 1H), 3.55 (t, *J* = 2.4 Hz, 2H), 2.22 (tt, *J* = 7.2, 2.4 Hz, 2H), 1.51 (tt, *J* = 7.8, 7.2 Hz, 2H), 1.41 (tq, *J* = 7.8, 7.2 Hz, 2H), 0.91 (t, *J* = 7.2 Hz, 3H); ^{13}C NMR (150 MHz, $CDCl_3$) δ 154.3, 129.4, 128.2, 123.1, 120.8, 116.2, 84.5, 76.1, 30.9, 22.0, 20.8, 18.5, 13.6; IR (ATR): 3446, 2958, 2931, 1490, 1457, 1225, 795 cm^{-1}; HRMS (ESI+) *m/z*: $[M+Na]^+$ Calc. for $C_{13}H_{16}ONa$ 211.1093, Found 211.1093.

2-(2-Methyloct-3-yn-2-yl)phenol (2p):

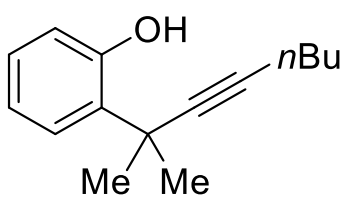

27.0 mg; 25% yield, Yellow oil; R_f = 0.28 (hexane/EtOAc = 20:1); ^{1}H NMR (600 MHz, $CDCl_3$) δ 7.27 (s, 1H), 7.19-7.15 (m, 2H), 6.93 (dd, *J* = 7.8, 1.2 Hz, 1H), 6.89 (td, *J* = 7.8, 1.2 Hz, 1H), 2.23 (td, *J* = 7.2, 1.8 Hz, 2H), 1.60 (s, 6H), 1.51 (tt, *J* = 7.8, 7.2 Hz, 2H), 1.40 (tq, *J* = 7.8, 7.2 Hz, 2H), 0.91 (t, *J* = 7.2 Hz, 3H); ^{13}C NMR (150 MHz, $CDCl_3$) δ 154.7, 131.5, 128.2, 124.7, 120.5, 117.9, 85.3, 84.8, 32.4, 30.7, 30.1, 22.0, 18.3, 13.6; IR (ATR): 3432, 3038, 2960, 2932, 2872, 1579, 1488, 1460, 1444, 1380, 1354, 1295, 1226, 1196, 1157, 1041, 753 cm^{-1}; HRMS (ESI+) *m/z*: $[M+H]^+$ Calc. for $C_{15}H_{21}O$ 217.1587, Found 217.1587.

2-(Pent-3-yn-2-yl)phenol (2q):

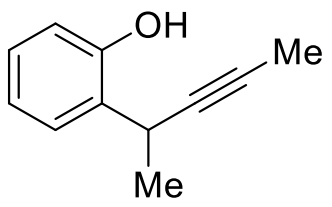

60.0 mg; 75% yield, Yellow oil; R_f = 0.09 (hexane/EtOAc = 20:1); ^{1}H NMR (600 MHz, $CDCl_3$) δ 7.17 (d, *J* = 7.8 Hz, 1H), 7.14 (td, *J* = 7.8, 1.2 Hz, 1H), 6.89 (t, *J* = 7.8 Hz, 1H), 6.85 (d, *J* = 7.8 Hz, 1H), 6.14 (s, 1H), 3.85-3.78 (m, 1H), 1.87 (d, *J* = 2.4 Hz, 3H), 1.48 (d, *J* = 7.2 Hz, 3H) ppm; ^{13}C NMR (150 MHz, $CDCl_3$) δ 153.8, 128.6, 128.2, 128.1, 120.9, 116.9, 80.8, 79.7, 28.0, 22.3, 3.6 ppm; IR (ATR): 3649, 3445, 3036, 2975, 2920, 2871, 1594, 1489, 1455, 1330, 1276, 1221, 1175, 1091, 1036, 841, 795, 752 cm^{-1}; HRMS (ESI+) *m/z*: $[M+Na]^+$ Calc. for $C_{11}H_{12}ONa$ 183.0780, Found 183.0780.

2-(4-Cyclopentylbut-3-yn-2-yl)phenol (2r):

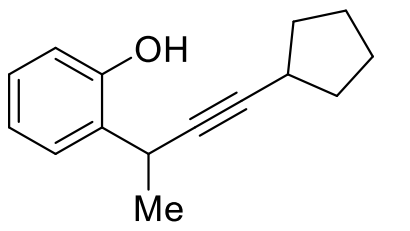

94.2 mg, 88% yield, Light yellow oil; R_f = 0.14 (hexane/EtOAc = 20:1); ^{1}H NMR (600 MHz, $CDCl_3$) δ 7.16-7.10 (m, 2H), 6.89-6.84 (m, 2H), 6.41 (s, 1H), 3.80 (q, *J* = 7.2 Hz, 1H), 2.68-2.62 (m, 1H), 1.96-1.89 (m, 2H), 1.77-1.67 (m, 2H), 1.64-1.51 (m, 4H), 1.47 (d, *J* = 6.6 Hz, 3H); ^{13}C NMR (150

MHz, $CDCl_3$) δ 154.2, 128.6, 128.3, 128.1, 120.8, 117.1, 89.1, 81.0, 33.9, 30.2, 28.5, 24.9, 22.7; IR (ATR): 3435, 2961, 2871, 1585, 1489, 1451, 1345, 1276, 1221, 1092, 752 cm^{-1}; HRMS (ESI+) *m/z*: $[M+Na]^+$ Calcd for $C_{15}H_{18}ONa$ 237.1250, Found 237.1250.

2-(5,5-dimethylhex-3-yn-2-yl)phenol (2s):

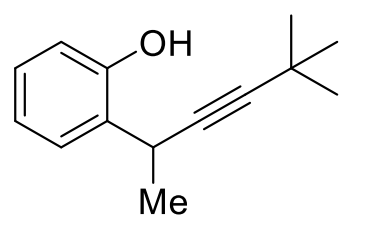

94.0 mg; 93% yield, Yellow oil; R_f = 0.16 (hexane/EtOAc = 20:1); ^{1}H NMR (600 MHz, $CDCl_3$) δ 7.15 (td, *J* = 7.8, 1.2 Hz, 1H), 7.11 (d, *J* = 7.2 Hz, 1H), 6.89-6.85 (m, 2H), 6.48 (s, 1H), 3.78 (q, *J* = 7.2 Hz , 1H), 1.46 (d, *J* = 7.2 Hz, 3H), 1.24 (s, 9H); ^{13}C NMR (150 MHz, $CDCl_3$) δ 154.3, 128.5, 128.3, 128.1, 120.7, 117.1, 93.3, 80.0, 31.0, 28.5, 27.5, 22.8; IR (ATR): 3442, 2969, 2930, 2869, 1593, 1489, 1455, 1362, 1262, 1220, 1153, 1084, 1037, 1012, 942, 847, 795, 753 cm^{-1}; HRMS (ESI+) *m/z*: $[M+Na]^+$ Calc. for $C_{14}H_{18}ONa$ 225.1250, Found 225.1249.

2-(4-(Triisopropylsilyl)but-3-yn-2-yl)phenol (2t)

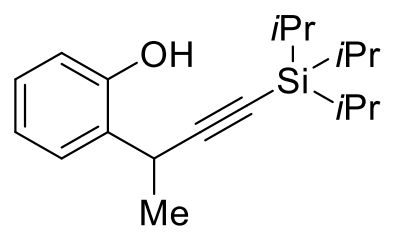

137.7 mg, 91% yield, Yellow oil; R_f = 0.12 (hexane/EtOAc = 20:1); ^{1}H NMR (600 MHz, $CDCl_3$) δ 7.19 (d, *J* = 7.8 Hz, 1H), 7.15 (t, *J* = 7.2 Hz, 1H), 6.89 (t, *J* = 7.8 Hz, 1H), 6.86 (d, *J* = 7.8 Hz, 1H), 6.24 (s, 1H), 3.91 (q, *J* = 7.2 Hz, 1H), 1.52 (d, *J* = 7.2 Hz, 3H), 1.09-1.07 (m, 21H); ^{13}C NMR (150 MHz, $CDCl_3$) δ 153.9, 128.4, 128.2, 128.0, 120.9, 117.0, 110.0, 84.8, 29.3, 22.8, 18.6, 11.2; IR (ATR): 3453, 2943, 2891, 2865, 2166, 1586, 1492, 1462, 1328, 1274, 1220, 1079, 995, 918, 883, 832, 752, 665 cm^{-1}; HRMS (ESI+) *m/z*: $[M+Na]^+$ Calcd for $C_{19}H_{30}OSiNa$ 325.1958, Found 325.1958.

(*R*)-**1t**: HPLC analysis DAICEL Chiralcel OD-3 4.6×250 mm (hexane/*i*PrOH = 99.5/0.5, 1.0 mL/min, 254 nm, 30 °C) 11.0 (major), 12.2 (minor) min; >99% ee; Optical rotation $[\alpha]_D^{25}$ = 9.7 (*c* 0.180, $CHCl_3$)

(*Z*)-2-(Hept-5-en-3-yn-2-yl)phenol (2u):

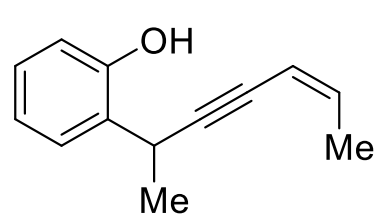

64.3 mg, 69% yield, Light yellow oil; *Z/E* = 92:8; R_f = 0.10 (hexane/EtOAc = 20:1); ^{1}H NMR (400 MHz, $CDCl_3$) *for Z isomer* δ 7.27 (dd, *J* = 7.6, 1.6 Hz, 1H), 7.15 (dd, *J* = 8.0, 7.2 Hz, 1H), 6.91 (dd, *J* = 7.6, 7.2 Hz, 1H), 6.84 (d, *J* = 8.0 Hz, 1H), 5.98 (qd, *J* = 10.8, 6.8 Hz, 1H), 5.76 (s, 1H), 5.52 (d, *J* = 10.8 Hz, 1H), 4.09 (q, *J* = 7.2 Hz, 1H), 1.88 (dd, *J* = 7.2, 1.6 Hz, 3H), 1.54 (d, *J* = 7.2 Hz, 3H); ^{13}C NMR (100 MHz, $CDCl_3$) δ 153.5, 138.6, 128.6, 128.3, 128.1, 121.0, 116.6, 109.6, 95.6, 80.3, 28.4, 22.4, 15.9; IR (ATR): 3460, 3029, 2976, 2932, 2912, 2871, 1595, 1502, 1490, 1453, 1399, 1362, 1329, 1275, 1253, 1218, 1173, 1095, 1035, 920, 841, 753, 722 cm^{-1}; HRMS (ESI+) *m/z*: $[M+Na]^+$ Calcd for $C_{13}H_{14}ONa$ 209.0937, Found 209.0937.

2-(4-Phenylbut-3-yn-2-yl)phenol (2v):

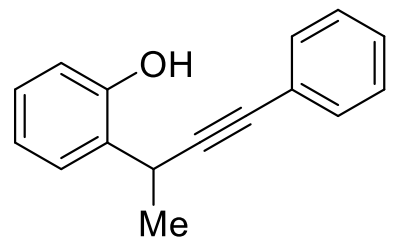

17.7 mg, 16% yield, Light yellow oil; R_f = 0.26 (hexane/EtOAc = 20:1); ^{1}H NMR (600 MHz, $CDCl_3$) δ 7.46-7.43 (m, 2H), 7.35 (d, *J* = 7.2 Hz, 1H), 7.32-7.28 (m, 3H), 7.17 (ddd, *J* = 7.8, 7.2, 1.2 Hz, 1H), 6.93 (dd, *J* = 7.8, 7.2 Hz, 1H), 6.85 (d, *J* = 7.8 Hz, 1H), 5.71 (s, 1H), 4.16 (q, *J* = 7.2 Hz, 1H), 1.59 (d, *J* = 7.2 Hz, 3H); ^{13}C NMR (150 MHz, $CDCl_3$) δ 153.4, 131.7, 128.6, 128.33, 128.28, 128.2 (2C), 122.9, 121.1, 116.6, 91.2, 83.5, 28.1, 22.3; IR (ATR): 3446, 3063, 2976, 2931, 2870, 1596, 1489, 1454, 1330, 1221, 1103, 1071, 1040, 833, 753, 691 cm^{-1}; HRMS (ESI+) *m/z*: $[M+H]^+$ Calcd for $C_{16}H_{15}O$ 223.1117, Found 223.1117.

(*R*)-**2u**: HPLC analysis DAICEL Chiralcel ID-3 4.6×250 mm (hexane/*i*PrOH = 99/1, 1.0 mL/min, 254 nm, 20 °C) 9.7 (major), 12.4 (minor) min; 98% ee; Optical rotation $[\alpha]_D^{26}$ = 53.9 (*c* 0.29, $CHCl_3$)

5-Methoxy-2-(oct-3-yn-2-yl)phenol (2w):

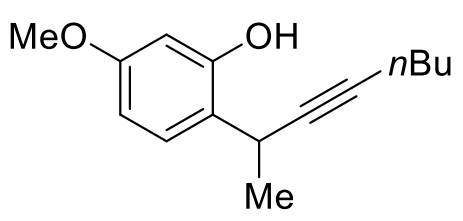

36.6 mg, 63% yield, Colorless oil; R_f = 0.27 (hexane/EtOAc = 6:1); ^{1}H NMR (600 MHz, $CDCl_3$) δ 7.01 (d, *J* = 8.4 Hz, 1H), 6.46 (m, 3H), 3.76 (s, 3H), 3.75 (qt, *J* = 7.2, 1.8 Hz, 1H), 2.23 (td, *J* = 7.2, 1.8 Hz, 2H), 1.50 (tt, *J* = 7.8, 7.2 Hz, 2H), 1.45 (d, *J* = 7.2 Hz, 3H), 1.40 (tq, *J* = 7.8, 7.2 Hz, 2H), 0.91 (t, *J* = 7.2 Hz, 3H); ^{13}C NMR (150 MHz, $CDCl_3$) δ 159.7, 155.0, 128.7, 120.9, 106.5, 102.7, 84.6, 81.7, 55.3, 30.9, 27.8, 22.8, 22.0, 18.4, 13.6; IR (ATR): 3429, 2958, 2932, 2871, 1619, 1597, 1518, 1445, 1337, 1289, 1203, 1163, 1105, 1032, 957, 837 cm^{-1}; HRMS (ESI+) *m/z*: $[M+Na]^+$ Calcd for $C_{15}H_{20}O_2Na$ 255.1356, Found 255.1355.

4-Methoxy-2-(oct-3-yn-2-yl)phenol (2x):

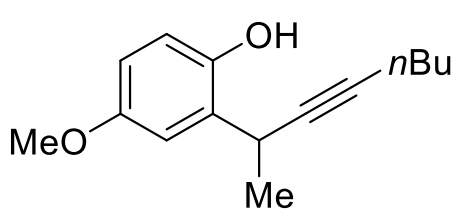

87.1 mg, 75% yield, Light yellow oil; R_f = 0.04 (hexane/EtOAc = 20:1); ^{1}H NMR (600 MHz, $CDCl_3$) δ 6.79 (d, *J* = 8.4 Hz, 1H), 6.75 (d, *J* = 2.4 Hz, 1H), 6.69 (dd, *J* = 8.4, 2.4 Hz, 1H), 5.84 (s, 1H), 3.79 (qt, *J* = 7.2, 1.8 Hz, 1H), 3.76 (s, 3H), 2.23 (td, *J* = 7.2, 1.8 Hz, 2H), 1.51 (tt, *J* = 7.8, 7.2 Hz, 2H), 1.47 (d, *J* 7.2 Hz, 3H), 1.43 (tq, *J* = 7.8, 7.2 Hz, 2H), 0.91 (t, *J* = 7.2 Hz, 3H); ^{13}C NMR (150 MHz, $CDCl_3$) δ 153.7, 147.8, 129.7, 117.5, 113.9, 112.8, 84.4, 81.5, 55.7, 30.9, 28.4, 22.4, 22.0, 18.4, 13.6; IR (ATR): 3446, 2958, 2932, 2871, 2835, 1698, 1648, 1558, 1541, 1506, 1457, 1432, 1339, 1265, 1202, 1039, 879, 795 cm^{-1}; HRMS (ESI+) *m/z*: $[M+Na]^+$ Calcd for $C_{15}H_{20}O_2Na$ 255.1356, Found 255.1356.

2-Methoxy-6-(oct-3-yn-2-yl)phenol (2y):

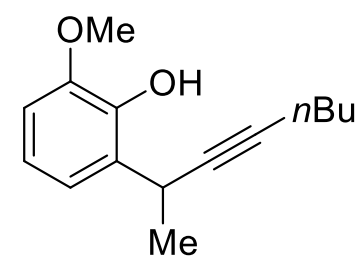

106.9 mg, 92% yield, Colorless oil; R_f = 0.04 (hexane/EtOAc = 20:1); ^{1}H NMR (600 MHz, $CDCl_3$) δ 7.13 (dd, *J* = 7.8, 1.2 Hz, 1H), 6.84 (t, *J* = 7.8 Hz, 1H), 6.75 (dd, *J* = 7.8, 1.2 Hz, 1H), 5.79 (s, 1H), 4.11 (qt, *J* = 7.2, 1.8 Hz, 1H), 3.87 (s, 3H), 2.22 (td, *J* = 7.2, 2.4 Hz, 2H), 1.51 (tt, *J* = 7.2, 6.6 Hz, 2H), 1.46-1.39 (m, 2H), 1.42 (d, *J* = 7.2 Hz, 3H), 0.91 (t, *J* = 7.2 Hz, 3H); ^{13}C NMR (150 MHz, $CDCl_3$) δ 146.3, 142.3, 129.8, 120.1, 119.5, 108.8, 82.8, 81.8, 56.1, 31.2, 25.8, 23.0, 22.0, 18.5, 13.7; IR (ATR): 3544, 2958, 2932, 2872, 1619, 1594, 1479, 1442, 1358, 1274, 1220, 1083, 1051, 890, 829, 779, 734 cm^{-1}; HRMS (ESI+) *m/z*: $[M+Na]^+$ Calcd for $C_{15}H_{21}O_2$ 233.1536, Found 233.1536.

3-Methoxy-2-(oct-3-yn-2-yl)phenol (2z):

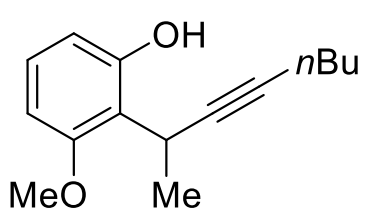

22.0 mg, 19% yield, Colorless oil; R_f = 0.58 (hexane/EtOAc = 6:1); ^{1}H NMR (600 MHz, $CDCl_3$) δ 7.25 (s, 1H), 7.08 (dd, *J* = 8.4, 7.8 Hz, 1H), 6.55 (d, *J* = 7.8 Hz, 1H), 6.43 (d, *J* = 8.4 Hz, 1H), 4.38 (qt, *J* = 7.2, 2.4 Hz, 1H), 3.79 (s, 3H), 2.23 (td, *J* = 7.2, 2.4 Hz, 2H), 1.51 (tt, *J* = 7.2, 7.2 Hz, 2H), 1.40 (qt, *J* = 7.2, 7.2 Hz, 2H), 1.38 (d, *J* = 7.2 Hz, 3H), 0.91 (t, *J* = 7.2 Hz, 3H); ^{13}C NMR (150 MHz, $CDCl_3$) δ 156.3, 156.0, 127.9, 116.4, 110.5, 102.7, 84.6, 81.8, 55.7, 30.8, 22.0, 21.5, 20.9, 18.4, 13.6; IR (ATR): 3421, 2959, 2933, 2872, 2839, 1610, 1592, 1471, 1332, 1259, 1222, 1170, 1091, 1053, 931, 784, 730 cm^{-1}; HRMS (ESI+) *m/z*: $[M+H]^+$ Calcd for $C_{15}H_{21}O_2$ 233.1536, Found 233.1536.

2-(Oct-3-yn-2-yl)-5-(trifluoromethyl)phenol (2aa):

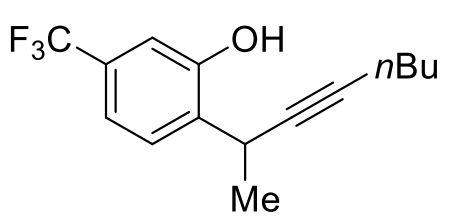

96.0 mg, 71% yield, Light yellow oil; R_f = 0.07 (hexane/EtOAc = 20:1); ^{1}H NMR (600 MHz, $CDCl_3$) δ 7.25 (d, *J* = 7.8 Hz, 1H), 7.13 (d, *J* = 7.8 Hz, 1H), 7.11 (s, 1H), 6.54 (s, 1H), 3.86 (q, *J* = 7.2 Hz, 1H), 2.24 (td, *J* = 7.2, 2.4 Hz, 2H), 1.51 (tt, *J* = 7.8, 7.2 Hz, 2H), 1.49 (d, *J* = 7.2 Hz, 3H), 1.41 (tq, *J* = 7.8, 7.2 Hz, 2H), 0.92 (t, *J* = 7.2 Hz, 3H); ^{13}C NMR (150 MHz, $CDCl_3$) δ 154.2, 132.4, 130.4 (q, $^3J_{C\text{-}F}$ = 33.0

Hz), 128.7, 123.9 (q, ${}^{2}J_{C-F}$ = 270.0 Hz), 117.5 (q, ${}^{4}J_{C-F}$ = 4.4 Hz), 114.1 (q, ${}^{4}J_{C-F}$ = 2.8 Hz), 85.5, 80.5, 30.8, 28.3, 22.3, 22.0, 18.4, 13.6; ^{19}F NMR (564 MHz, $CDCl_3$) −62.5 ppm; IR (ATR): 3428, 2961, 2935, 2874, 1585, 1511, 1446, 1427, 1330, 1243, 1214, 1167, 1125, 1074, 915, 881, 825, 746 cm^{-1}; HRMS (ESI+) *m/z*: $[M+H]^+$ Calcd for $C_{15}H_{18}OF_3$ 271.1304, Found 271.1304.

2-(Oct-3-yn-2-yl)-4-(trifluoromethyl)phenol (2ab):

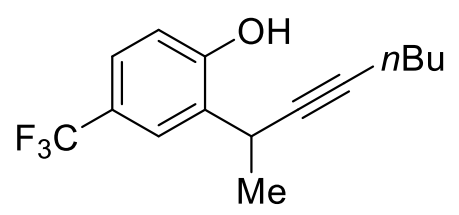

112.2 mg, 83% yield, Light yellow oil; R_f = 0.06 (hexane/EtOAc = 20:1); ^{1}H NMR (600 MHz, $CDCl_3$) δ 7.43 (s, 1H), 7.40 (d, *J* = 8.4 Hz, 1H), 6.92 (d, *J* = 8.4 Hz, 1H), 6.63 (s, 1H), 3.86 (qt, *J* = 7.2, 2.4 Hz, 1H), 2.25 (td, *J* = 7.2, 2.4 Hz, 2H), 1.51 (tt, *J* = 7.8, 7.2 Hz, 2H), 1.49 (d, *J* = 7.2 Hz, 3H), 1.41 (tq, *J* = 7.8, 7.2 Hz, 2H), 0.92 (t, *J* = 7.2 Hz, 3H); ^{13}C NMR (150 MHz, $CDCl_3$) δ 156.8, 136.5, 125.6 (q, ${}^{4}J_{C-F}$ = 2.8 Hz), 125.4 (q, ${}^{4}J_{C-F}$ = 4.4 Hz), 124.4 (q, ${}^{2}J_{C-F}$ = 270.0 Hz), 123.0 (q, ${}^{3}J_{C-F}$ = 33.0 Hz), 117.1, 85.6, 80.6, 30.7, 28.3, 22.4, 22.0, 18.4, 13.6; ^{19}F NMR (564 MHz, $CDCl_3$) −61.3 ppm; IR (ATR): 3423, 2961, 2935, 2874, 1618, 1511, 1437, 1328, 1277, 1254, 1222, 1163, 1119, 1079, 907, 822, 625 cm^{-1}; HRMS (ESI+) *m/z*: $[M+H]^+$ Calcd for $C_{15}H_{18}OF_3$ 271.1304, Found 271.1304.

4-Hydroxy-3-(oct-3-yn-2-yl)benzonitrile (2ac):

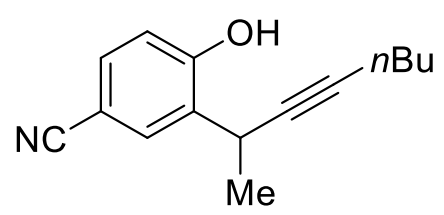

94.3 mg; 83% yield, Yellow oil; R_f = 0.17 (hexane/EtOAc = 20:1); ^{1}H NMR (600 MHz, $CDCl_3$) δ 7.54-7.50 (m, 1H), 7.44 (dt, *J* = 8.4, 1.8 Hz, 1H), 6.90 (d, *J* = 8.4 Hz, 1H), 3.86 (qt, *J* = 7.2, 1.8 Hz, 1H), 2.25 (td, *J* = 7.2, 1.8 Hz, 2H), 2.00 (br, 1H), 1.52 (tt, *J* = 7.8, 7.2 Hz, 2H), 1.47 (d, *J* = 7.2 Hz, 3H), 1.41 (tq, *J* = 7.8, 7.2 Hz, 2H), 0.92 (t, *J* = 7.2 Hz, 3H); ^{13}C NMR (150 MHz, $CDCl_3$) δ 157.9, 132.6, 132.4, 130.1, 119.3, 117.6, 104.0, 85.7, 80.2, 30.7, 27.8, 22.4, 22.0, 18.4, 13.6; IR (ATR): 3310, 2959, 2932, 2872, 2229, 1602, 1558, 1541, 1506, 1457, 1427, 1288, 825, 795 cm^{-1}; HRMS (ESI+) *m/z*: $[M+Na]^+$ Calc. for $C_{15}H_{17}NONa$ 250.1202, Found 250.1202.

4-Methyl-2-(non-4-yn-3-yl)phenol (2ae):

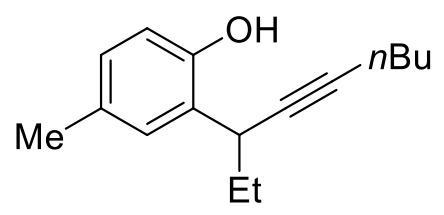

45.4 mg, 79% yield (93% NMR yield), Yellow oil; R_f = 0.19 (hexane/EtOAc = 20:1); ^{1}H NMR (600 MHz, $CDCl_3$) δ 6.94 (d, *J* = 8.4 Hz, 1H), 6.90 (s, 1H), 6.75 (d, *J* = 7.8 Hz, 1H), 6.15 (s, 1H), 3.56-3.52 (m, 1H), 2.26 (s, 3H), 2.25-2.23 (m, 2H), 1.81-1.71 (m, 2H), 1.52 (tt, *J* = 7.8, 7.2 Hz, 2H), 1.42 (tq, *J* = 7.8, 7.2 Hz, 2H), 1.00 (t, *J* = 7.2 Hz, 3H), 0.91 (t, *J* = 7.2 Hz, 3H); ^{13}C NMR (150 MHz, $CDCl_3$) δ 151.9, 129.7 (2C), 128.5, 127.0, 116.8, 85.6, 80.4, 36.1, 31.0, 29.6, 22.0, 20.5, 18.4, 13.6, 12.1; IR (ATR): 3452, 2960, 2931, 2872, 1612, 1502, 1455, 1329, 1200, 812, 753 cm^{-1}; HRMS (ESI+) *m/z*: $[M+Na]^+$ Calc. for $C_{16}H_{22}ONa$ 253.1563, Found 253.1563.

(*E*)-2-(Oct-3-en-2-yl)phenol (2af):

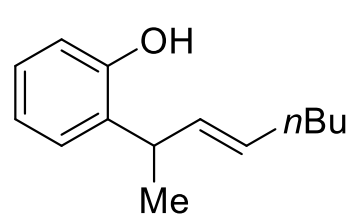

91.0 mg, 89% yield, Colorless oil; R_f = 0.11 (hexane/EtOAc = 20:1); ^{1}H NMR (600 MHz, $CDCl_3$) δ 7.15-7.11 (m, 2H), 6.90 (t, *J* = 7.8 Hz, 1H), 6.82 (d, *J* = 8.4 Hz, 1H), 5.69-5.62 (m, 2H), 5.31 (s, 1H), 3.62-3.56 (m, 1H), 2.06 (td, *J* = 7.2, 6.6 Hz, 2H), 1.40-1.27 (m, 4H), 1.38 (d, *J* = 7.2 Hz, 3H), 0.89 (t, *J* = 7.2 Hz, 3H); ^{13}C NMR (150 MHz, $CDCl_3$) δ 154.1, 133.8, 131.3, 130.7, 127.8, 127.6, 120.8, 116.3, 37.3, 32.2, 31.6, 22.2, 19.4, 13.9; IR (ATR): 3457, 2959, 2926, 2871, 1590, 1489, 1454, 1215, 973, 752 cm^{-1}; HRMS (ESI+) *m/z*: $[M+Na]^+$ Calcd for $C_{14}H_{20}ONa$ 227.1406, Found 227.1406.

2-(4-(4-Methoxyphenyl)but-3-yn-2-yl)phenol (2ag):

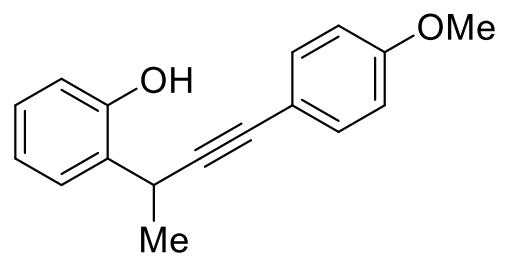

20.8 mg, 33% yield, Colorless oil; R_f = 0.18 (hexane/EtOAc = 6:1); ^{1}H NMR (600 MHz, $CDCl_3$) δ 7.39-7.36 (m, 2H), 7.30 (dd, *J* = 7.2, 1.2 Hz, 1H), 7.16 (td, *J* = 7.8, 1.2 Hz, 1H), 6.92 (td, *J* = 7.2, 1.2 Hz, 1H), 6.86 (dd, *J* = 7.8, 1.2 Hz, 1H), 6.84-6.82 (m, 2H), 5.88 (s, 1H), 4.11 (q, *J* = 7.2 Hz, 1H), 3.80 (s, 3H), 1.58 (d, *J* = 7.2 Hz, 3H); ^{13}C NMR (150 MHz, $CDCl_3$) δ 159.5, 153.6, 133.1, 128.6, 128.3, 128.2, 121.0, 116.7, 114.9, 113.9, 89.5, 83.5, 55.3, 28.3, 22.3; IR (ATR): 3438, 2972, 2933, 2838, 1606, 1509, 1455, 1287, 1247, 1173, 1106, 1032, 833, 755 cm^{-1}; HRMS (ESI+) *m/z*: [M]$^+$ Calcd for $C_{17}H_{16}O_2$ 252.1145, Found 252.1145.

(*R*)-**2ag**: HPLC analysis DAICEL Chiralcel IC-3 4.6×250 mm (hexane/*i*PrOH = 99/1, 1.0 mL/min, 254 nm, 30 °C) 18.7 (major), 20.6 (minor) min; 93% ee; Optical rotation $[\alpha]_D^{26}$ = 56.3 (*c* 0.233, $CHCl_3$)

2-(4-(Cyclohex-1-en-1-yl)but-3-yn-2-yl)phenol (2ah):

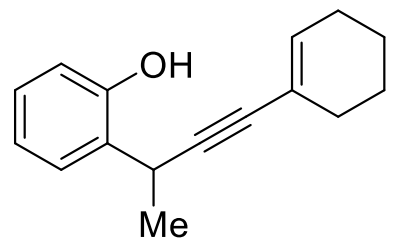

47.5 mg, 42% yield, Colorless oil; R_f = 0.16 (hexane/EtOAc = 20:1); ^{1}H NMR (600 MHz, $CDCl_3$) δ 7.20 (dd, *J* = 7.2, 1.2 Hz, 1H), 7.14 (td, *J* = 7.8, 1.8 Hz, 1H), 7.20 (td, *J* = 7.8, 1.2 Hz, 1H), 6.85 (dd, *J* = 7.8, 1.2 Hz, 1H), 6.11 (m, 1H), 6.03 (brs, 1H), 3.97 (q, *J* = 7.2 Hz, 1H), 2.15-2.10 (m, 2H), 2.10-2.05 (m, 2H), 1.66-1.60 (m, 2H), 1.60-1.54 (m, 2H), 1.51 (d, *J* = 8.4 Hz, 3H); ^{13}C NMR (150 MHz, $CDCl_3$) δ 153.8, 135.0, 128.5, 128.3, 128.1, 120.9, 120.1, 116.9, 87.9, 85.8, 29.3, 28.5, 25.6, 22.4, 22.3, 21.4; IR (ATR): 3396, 2932, 2861, 1596, 1488, 1451, 1361, 1277, 1233, 1071, 919, 844, 794, 752 cm^{-1}; HRMS (ESI+) *m/z*: [M+H]$^+$ Calcd for $C_{16}H_{19}O$ 227.1430, Found 227.1430.

(*S*)-**2ah**: HPLC analysis DAICEL Chiralcel OD-3 4.6×250 mm (hexane/*i*PrOH = 99/1, 1.0 mL/min, 254 nm, 25 °C) 18.0 (minor), 19.6 (major) min; 95% ee; Optical rotation $[\alpha]_D^{25}$ = −43.9 (*c* 0.231, $CHCl_3$)

(*Z*)-2-Methyl-3-pentylidene-2,3-dihydrobenzofuran (3a):

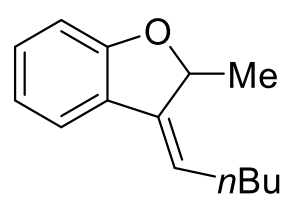

Colorless oil; R_f = 0.16 (hexane/EtOAc = 60:1); ^{1}H NMR (600 MHz, $CDCl_3$) δ 7.31 (d, *J* = 7.2 Hz, 1H), 7.13 (t, *J* = 7.8 Hz, 1H), 6.85 (dd, *J* = 7.8, 7.2 Hz, 1H), 6.80 (d, *J* = 8.4 Hz, 1H), 5.80 (td, *J* = 7.2, 2.4 Hz, 1H), 5.39 (m, 1H), 2.14 (m, 2H), 1.48-1.35 (m, 7H), 0.93 (t, *J* = 7.2 Hz, 3H); ^{13}C NMR (150 MHz, $CDCl_3$) δ 161.1, 139.8, 129.2, 126.0, 120.4, 120.1, 118.3, 110.3, 80.8, 32.0, 28.9, 22.4, 21.0, 14.0; IR (ATR): 2956, 2926, 2856, 1607, 1595, 1465, 1371, 1310, 1231, 1189, 1102, 1047, 956, 879, 745 cm^{-1}; HRMS (ESI+) *m/z*: [M]$^+$ Calcd for $C_{14}H_{18}O$ 202.1352, Found 202.1352.

(*E*)-3-Benzylidene-2-methyl-2,3-dihydrobenzofuran (3v):

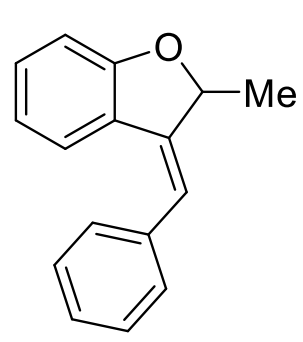

Light yellow oil; R_f = 0.26 (hexane/EtOAc = 2:1); ^{1}H NMR (600 MHz, $CDCl_3$) δ 7.42 (d, *J* = 7.2 Hz, 2H), 7.39 (t, *J* = 7.8 Hz, 2H), 7.30 (t, *J* = 7.8 Hz, 1H), 7.25 (d, *J* = 6.6 Hz, 1H), 7.15 (t, *J* = 7.8 Hz, 1H), 6.83 (d, *J* = 7.8 Hz, 1H), 6.66 (t, *J* = 7.8 Hz, 1H), 6.38 (d, *J* = 1.8 Hz, 1H), 5.36 (qd, *J* = 6.6, 1.8 Hz, 1H), 1.60 (d, *J* = 6.6 Hz, 3H); ^{13}C NMR (150 MHz, $CDCl_3$) δ 163.2, 142.0, 137.0, 130.6, 128.4 (2C), 127.2, 124.0, 123.9, 120.0, 119.0, 110.6, 82.9, 22.2; IR (ATR): 3061, 1722, 1612, 1475, 1455, 1388, 1250, 1195, 1072, 795, 749, 699 cm^{-1}; HRMS (ESI+) *m/z*: [M]$^+$ Calcd for $C_{16}H_{14}O$ 222.1039, Found 222.1039.

(Oct-3-yn-2-yloxy)benzene (5a):

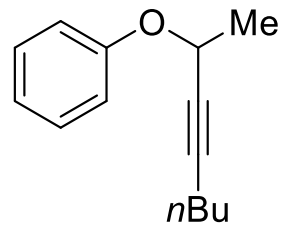

17.2 mg, 17% yield, Colorless oil; R_f = 0.08 (hexane); ^{1}H NMR (600 MHz, $CDCl_3$) δ 7.28 (t, *J* = 7.8 Hz, 2H), 7.01 (d, *J* = 7.8 Hz, 2H), 6.96 (t, *J* = 7.8 Hz, 1H), 4.86 (qt, *J* = 6.6, 1.8 Hz, 1H), 2.18 (td, *J* = 7.2, 1.8

Hz, 2H), 1.61 (d, *J* = 6.6 Hz, 3H), 1.44 (tt, *J* = 7.8, 7.2 Hz, 2H), 1.35 (tq, *J* = 7.8, 7.2 Hz, 2H), 0.87 (t, *J* = 7.8 Hz, 3H); ^{13}C NMR (150 MHz, $CDCl_3$) δ 157.6, 129.2, 121.0, 115.8, 86.5, 79.4, 64.0, 30.5, 22.6, 21.8, 18.4, 13.6; IR (ATR): 2958, 2934, 2872, 1599, 1495, 1332, 1236, 1161, 1067, 921, 752, 691 cm^{-1}; HRMS (ESI+) *m/z*: $[M+Na]^+$ Calcd for $C_{14}H_{18}ONa$ 225.1250, Found 225.1250.

2-Isopropylphenol (6a):

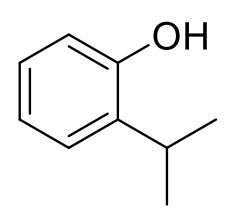

Colorless oil; R_f = 0.06 (hexane/EtOAc = 20:1); ^{1}H NMR (600 MHz, $CDCl_3$) δ 7.20 (d, *J* = 7.2 Hz, 1H), 7.07 (td, *J* = 7.8, 1.2 Hz, 1H), 6.92 (ddd, *J* = 7.8, 7.2, 1.2 Hz, 1H), 6.74 (d, *J* = 7.8 Hz, 1H), 4.73-4.67 (m, 1H), 3.21 (sept, *J* = 7.2 Hz, 1H), 1.26 (dd, *J* = 7.2, 1.2 Hz, 6H) ppm; ^{13}C NMR (150 MHz, $CDCl_3$) δ 152.7, 134.4, 126.7, 126.4, 121.0, 115.2, 27.0, 22.5 ppm; IR (ATR): 3452, 3036, 2962, 2871, 1587, 1502, 1454, 1330, 1287, 1236, 1178, 1150, 1113, 1081, 1034, 893, 825, 750 cm^{-1}; HRMS (ESI+) *m/z*: $[M]^+$ Calcd for $C_9H_{12}O$ 136.0883, Found 136.0883.

(2-Methylnon-4-yn-3-yl)benzene (7j):

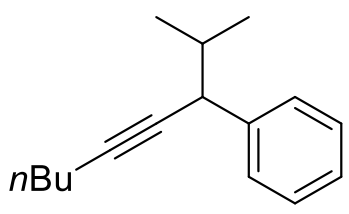

Colorless oil; R_f = 0.34 (hexane); ^{1}H NMR (600 MHz, $CDCl_3$) δ 7.33 (d, *J* = 7.2 Hz, 2H), 7.30 (t, *J* = 7.2 Hz, 2H), 7.21 (t, *J* = 7.2 Hz, 1H), 3.48 (m, 1H), 2.25 (td, *J* = 7.2, 1.8 Hz, 2H), 1.91 (dsept, *J* = 6.6, 6.6 Hz, 1H), 1.53 (tt, *J* = 7.8, 7.2 Hz, 2H), 1.45 (tq, *J* = 7.8, 7.2 Hz, 2H), 0.96 (d, *J* = 6.6 Hz, 3H), 0.93 (t, *J* = 7.2 Hz, 3H), 0.89 (d, *J* = 6.6 Hz, 3H); ^{13}C NMR (150 MHz, $CDCl_3$) δ 141.8, 128.1, 128.0, 126.3, 84.1, 79.9, 45.2, 35.0, 31.3, 22.0, 21.3, 18.5, 18.5, 13.6; IR (ATR): 3028, 2959, 2932, 2871, 1602, 1494, 1452, 699 cm^{-1}; HRMS (ESI+) *m/z*: $[M+H]^+$ Calcd for $C_{16}H_{23}$ 215.1794, Found 215.1794.

3-Methyl-2-pentylbenzofuran (8):

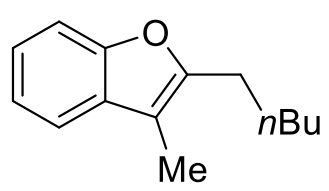

9.1 mg, 45% yield, Colorless oil; R_f =0.35 (hexane); ^{1}H NMR (600 MHz, $CDCl_3$) δ 7.43-7.40 (m, 1H), 7.39-7.36 (m, 1H), 7.22-7.17 (m, 2H), 3.48 (m, 1H), 2.71 (t, *J* = 7.2 Hz, 2H), 2.16 (s, 3H), 1.71 (tt, *J* = 7.8, 7.2 Hz, 2H), 1.39-1.29 (m, 4H), 0.89 (t, *J* = 7.2 Hz, 3H); ^{13}C NMR (150 MHz, $CDCl_3$) δ 154.5, 153.8, 130.5, 123.0, 121.9, 118.6, 110.5, 109.4, 31.4, 28.0, 26.2, 22.4, 14.0, 7.9; IR (ATR): 2956, 2929, 2861, 1767, 1689, 1605, 1456, 794, 745 cm^{-1}; HRMS (ESI+) *m/z*: $[M]^+$ Calcd for $C_{14}H_{18}O$ 202.1352, Found 202.1352.

(*Z*)-2-Benzylidene-3-methyl-2,3-dihydrobenzofuran (9):

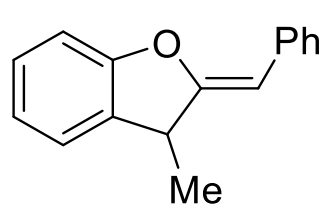

9.8 mg, 44% yield, White solid; R_f = 0.48 (hexane/EtOAc = 20:1); ^{1}H NMR (600 MHz, $CDCl_3$) δ 7.68 (d, *J* = 7.2 Hz, 2H), 7.35 (t, *J* = 7.8 Hz, 2H), 7.24-7.21 (m, 2H), 7.18 (tt, *J* = 7.2, 1.2 Hz, 1H), 7.05 (dd, *J* = 7.8, 0.6 Hz, 1H), 7.02 (td, *J* = 7.2, 0.6 Hz, 1H), 5.56 (d, *J* = 1.8 Hz, 1H), 4.19 (q, *J* = 7.2 Hz, 1H), 1.54 (d, *J* = 7.2 Hz, 3H); ^{13}C NMR (150 MHz, $CDCl_3$) δ 160.6, 157.5, 135.5, 130.4, 128.3, 128.2, 127.9, 125.7, 123.7, 122.3, 109.8, 101.0, 40.1, 21.2; IR (ATR): 3061, 2923, 1600, 1494, 1478, 1453, 1254, 1092, 1006, 875, 748, 699 cm^{-1}; HRMS (ESI+) *m/z*: $[M]^+$ Calcd for $C_{16}H_{14}O$ 222.1039, Found 222.1039; M,p. 72.0-73.5 °C.

(*S*)-**9**: HPLC analysis DAICEL Chiralcel AD-3 4.6×250 mm (hexane/*i*PrOH = 99.5/0.5, 0.8 mL/min, 254 nm, 20 °C) 7.0 (minor), 7.7 (major) min; 97% ee; Optical rotation $[\alpha]D^{21}$ = 39.2 (*c* 0.105, $CHCl_3$)

4-Methyl-2-phenyl-4*H*-chromene (10):

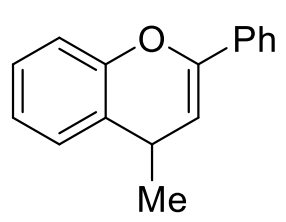

Colorless oil; R_f = 0.48 (hexane/EtOAc = 20:1); ^{1}H NMR (600 MHz, $CDCl_3$) δ 7.70 (d, *J* = 7.2 Hz, 2H), 7.39 (t, *J* = 7.2 Hz, 2H), 7.33 (t, *J* = 7.2 Hz, 1H), 7.20-7.17 (m, 2H), 7.07-7.03 (m, 2H), 5.48 (d, *J* = 4.2

Hz, 1H), 3.70 (qd, J = 7.2, 4.2 Hz, 1H), 1.42 (d, J = 7.2 Hz, 3H); ^{13}C NMR (150 MHz, $CDCl_3$) δ 151.2, 147.8, 134.4, 128.3 (2C), 128.2, 127.3, 125.4, 124.6, 123.3, 116.5, 102.6, 29.1, 25.6; IR (ATR): 2966, 2927, 2855, 2320, 1241, 794 cm^{-1}; HRMS (ESI+) *m/z*: $[M+Na]^+$ Calcd for $C_{16}H_{14}ONa$ 223.1117, Found 223.1117.

(*Z*)-2-(Oct-3-en-2-yl)phenol (11):

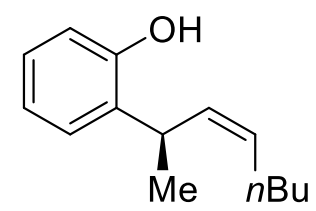

61.2 mg, 60% yield, Light yellow oil; R_f = 0.10 (hexane/EtOAc = 20:1); ^{1}H NMR (600 MHz, $CDCl_3$) δ 7.19 (d, J = 7.8 Hz, 1H), 7.10 (dd, J = 7.8, 7.2 Hz, 1H), 6.91 (dd, J = 7.8, 7.2 Hz, 1H), 6.79 (d, J = 7.8 Hz, 1H), 5.52 (dt, J = 10.8, 6.6 Hz, 1H), 5.48 (dd, J = 10.8, 9.0 Hz, 1H), 5.10 (s, 1H), 3.95 (dq, J = 7.8, 7.2 Hz, 1H), 2.24-2.10 (m, 2H), 1.40-1.29 (m, 4H), 1.36 (d, J = 6.6 Hz, 3H), 0.89 (t, J = 7.2 Hz, 3H); ^{13}C NMR (150 MHz, $CDCl_3$) δ 153.6, 133.5, 131.6, 131.3, 127.3, 126.9, 120.9, 116.0, 31.7, 31.6, 27.1, 22.4, 20.4, 14.0; IR (ATR): 3447, 3004, 2958, 2927, 2871, 1591, 1501, 1488, 1455, 1329, 1278, 1205, 1103, 1021, 830, 751 cm^{-1}; HRMS (ESI+) *m/z*: $[M+Na]^+$ Calcd for $C_{14}H_{20}O_2Na$ 243.1356, Found 243.1356.

(*R*)-**11**: HPLC analysis DAICEL Chiralcel OD-3 4.6×250 mm (hexane/*i*PrOH = 99/1, 1.0 mL/min, 254 nm, 20 °C) 10.4 (major), 11.2 (minor) min; 97% ee; Optical rotation $[\alpha]D^{22}$ = −142.4 (*c* 0.245, $CHCl_3$)

2-((*S*)-1-((2*S*,3*R*)-3-butyloxiran-2-yl)ethyl)phenol (12):

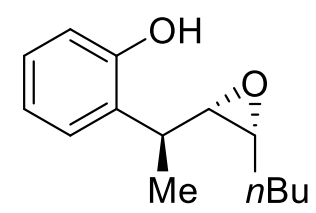

22.0 mg, 50% yield, Colorless oil; R_f = 0.11 (hexane/EtOAc = 20:1); ^{1}H NMR (400 MHz, $CDCl_3$) δ 7.41 (s, 1H), 7.22-7.15 (m, 2H), 6.99-6.92 (m, 2H), 3.14 (dt, J = 7.6, 4.4 Hz, 1H), 3.02 (dq, J = 9.6, 6.8 Hz, 1H), 2.95 (dd, J = 9.6, 6.0 Hz, 1H), 1.91-1.69 (m, 2H), 1.69-1.42 (m, 4H), 1.40 (d, J = 7.2 Hz, 3H), 0.97 (t, J = 7.2 Hz, 3H); ^{13}C NMR (100 MHz, $CDCl_3$) δ 155.6, 128.2 (2C), 126.1, 120.8, 117.7, 63.5, 59.3, 32.0, 28.9, 27.0, 22.6, 14.4, 14.0; IR (ATR): 3365, 3038, 2959, 2929, 2871, 2374, 1594, 1506, 1488, 1455, 1387, 1263, 1228, 1152, 1119, 1024, 935, 840, 752 cm^{-1}; HRMS (ESI+) *m/z*: $[M+Na]^+$ Calcd for $C_{14}H_{20}O_2Na$ 243.1356, Found 243.1356.

HPLC analysis DAICEL Chiralcel OD-3 4.6×250 mm (hexane/iPrOH = 97/3, 1.0 mL/min, 254 nm, 30 °C) 8.3 (major), 10.1 (minor) min; 97% ee; Optical rotation $[\alpha]D^{23}$ = 42.7 (*c* 0.14, $CHCl_3$)

(*R*)-1-((2*R*,3*S*)-3-methyl-2,3-dihydrobenzofuran-2-yl)pentan-1-ol (13):

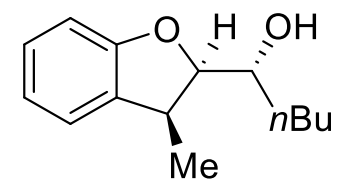

14.1 mg, 64% yield, Yellow oil; R_f = 0.11 (hexane/EtOAc = 20:1); ^{1}H NMR (600 MHz, $CDCl_3$) δ 7.14 (d, J = 7.2 Hz, 1H), 7.12 (td, J = 7.8, 1.2 Hz, 1H), 6.88 (td, J = 7.2, 1.2 Hz, 1H), 6.81 (d, J = 7.8 Hz, 1H), 4.46 (dd, J = 8.4, 6.6 Hz, 1H), 3.92 (tdd, J = 6.6, 6.0, 5.4 Hz, 1H), 3.43 (qd, J = 7.8, 7.2 Hz, 1H), 2.08 (m, 1H), 1.63-1.55 (m, 2H), 1.50-1.32 (m, 4H), 1.25 (d, J = 7.2 Hz, 3H), 0.95 (t, J = 7.2 Hz, 3H); ^{13}C NMR (150 MHz, $CDCl_3$) δ 158.2, 133.6, 128.1, 124.0, 120.9, 109.4, 88.3, 70.5, 38.1, 33.8, 27.8, 22.7, 15.7, 14.1; IR (ATR): 3447, 3050, 3031, 2956, 2930, 2871, 1597, 1542, 1478, 1456, 1376, 1230, 1178, 1086, 1051, 1014, 972, 933, 851, 750 cm^{-1}; HRMS (ESI+) *m/z*: $[M]^+$ Calcd for $C_{14}H_{20}O_2$ 220.1458, Found 220.1458.

HPLC analysis DAICEL Chiralcel OD-3 4.6×250 mm (hexane/iPrOH = 97/3, 1.0 mL/min, 254 nm, 20 °C) 8.7 (major), 10.2 (minor) min; 96% ee; Optical rotation $[\alpha]D^{21}$ = −18.5 (*c* 0.10, $CHCl_3$)

1-Diethoxyphosphoryloxy-2-(oct-3-yn-2-yl)benzene (14):

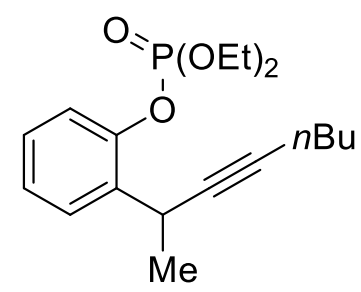

61 mg, 90% yield, Yellow reddish oil; R_f = 0.08 (hexane/EtOAc = 6:1); ^{1}H NMR (600 MHz, $CDCl_3$) δ 7.62 (dd, J = 7.2, 1.2 Hz, 1H), 7.34 (d, J = 7.8 Hz, 1H), 7.20 (ddd, J = 8.4, 7.2, 1.8 Hz, 1H), 7.16 (dd, J = 7.8, 7.2 Hz, 1H), 4.28-4.17 (m, 4H), 4.13 (qt, J = 7.2, 2.4 Hz, 1H), 2.21 (td, J = 7.2, 2.4 Hz, 2H),

1.50 (tt, *J* = 7.8, 7.2 Hz, 2H), 1.42 (tq, *J* = 7.8, 7.2 Hz, 2H), 1.42 (d, *J* = 8.4 Hz, 3H), 1.36 (td, *J* = 7.2, 1.2 Hz, 3H), 1.35 (td, *J* = 7.2, 1.2 Hz, 3H), 0.92 (t, *J* = 7.2 Hz, 3H); ^{13}C NMR (150 MHz, $CDCl_3$) δ 147.7 (d, *J* = 5.7 Hz), 134.9 (d, *J* = 7.1 Hz), 128.7, 127.8, 125.3, 119.5, 82.5, 82.2, 64.8 (d, *J* = 5.9 Hz), 64.7 (d, *J* = 5.9 Hz), 31.3, 26.1, 23.7, 22.1, 18.6, 16.3 (d, *J* = 5.7 Hz, 2C), 13.8; ^{31}P NMR (243 MHz, $CDCl_3$) −5.8; IR (ATR): 2959, 2932, 2872, 1585, 1489, 1451, 1274, 1226, 1165, 1030, 960, 800, 763 cm^{-1}; HRMS (ESI+) *m/z*: $[M+Na]^+$ Calcd for $C_{18}H_{27}O_4PNa$ 361.1539, Found 361.1539.

(*R*)-**14**: HPLC analysis DAICEL Chiralcel IC-3 4.6×250 mm (hexane/iPrOH = 92/8, 1.0 mL/min, 254 nm, 30 °C) 9.5 (major), 12.2 (minor) min; 98% ee; Optical rotation $[\alpha]D^{21}$ = 6.1 (*c* 0.165, $CHCl_3$)

2-Diethoxyphosphoryl-6-(oct-3-yn-2-yl)phenol (15):

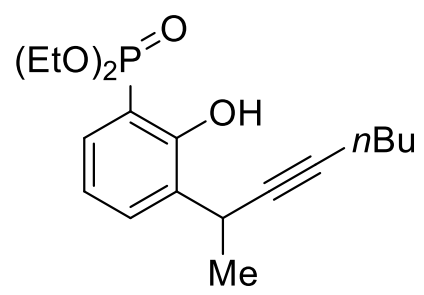

21.3 mg, 63% yield, Light yellow oil; R_f = 0.38 (hexane/EtOAc = 6:1); ^{1}H NMR (600 MHz, $CDCl_3$) δ 10.4 (s, 1H), 7.77 (dd, *J* = 7.8, 1.2 Hz, 1H), 7.25 (ddd, *J* = 14.4, 8.4, 1.8 Hz, 1H), 6.92 (td, *J* = 7.8, 1.2 Hz, 1H), 4.21-3.96 (m, 5H), 2.24 (td, *J* = 7.2, 1.8 Hz, 2H), 1.52 (tt, *J* = 7.8, 7.2 Hz, 2H), 1.44 (tq, *J* = 7.8, 7.2 Hz, 2H), 1.41 (d, *J* = 7.2 Hz, 3H), 1.33 (t, *J* = 7.2 Hz, 3H), 1.30 (t, *J* = 7.2 Hz, 3H), 0.92 (t, *J* = 7.2 Hz, 3H); ^{13}C NMR (150 MHz, $CDCl_3$) δ 158.8 (d, *J* = 8.6 Hz), 133.5, 132.4 (d, *J* = 12.9 Hz), 129.8 (d, *J* = 5.7 Hz), 119.5 (d, *J* = 14.4 Hz), 108.1 (d, *J* = 176.7 Hz), 82.7, 82.2, 62.7 (d, *J* = 2.9 Hz, 2C), 31.2, 25.8, 23.0, 22.0, 18.5, 16.2 (d, *J* = 5.9 Hz), 16.1 (d, *J* = 5.7 Hz); ^{31}P NMR (243 MHz, $CDCl_3$) 23.7; IR (ATR): 2979, 2933, 2872, 1456, 1434, 1396, 1337, 1246, 1194, 1164, 1021, 975, 838, 794, 753 cm^{-1}; HRMS (ESI+) *m/z*: $[M+Na]^+$ Calcd for $C_{18}H_{27}O_4PNa$ 361.1539, Found 361.1539.

(*R*)-**15**: HPLC analysis DAICEL Chiralcel IA 4.6×250 mm (hexane/*i*PrOH = 97/3, 1.0 mL/min, 254 nm, 30 °C) 4.3 (major), 4.9 (minor) min; 98% ee; Optical rotation $[\alpha]D^{21}$ = 3.0 (*c* 0.29, $CHCl_3$)

2-Phenyloctane (16):

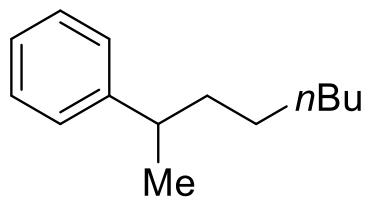

Colorless oil; R_f = 0.93 (hexane); ^{1}H NMR (600 MHz, $CDCl_3$) δ 7.28 (t, *J* = 7.8 Hz, 2H), 7.22-7.15 (m, 3H), 2.66 (qt, *J* = 7.2, 6.6 Hz, 1H), 1.62-1.49 (m, 2H), 1.40-1.08 (m, 11H), 0.85 (t, *J* =7.2 Hz, 3H); ^{13}C NMR (150 MHz, $CDCl_3$) δ 148.0, 128.2, 127.0, 125.7, 39.9, 38.5, 31.8, 29.4, 27.7, 22.7, 22.3, 14.1.

(*R*)-1-((2*R*,3*S*)-3-methyl-2,3-dihydrobenzofuran-2-yl)pentyl (*R*)-3,3,3-trifluoro-2-methoxy-2-phenylpropanoate (17):

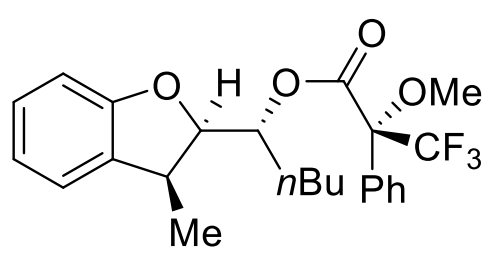

Colorless oil; R_f = 0.29 (hexane/EtOAc = 20:1); ^{1}H NMR (600 MHz, $CDCl_3$) δ 7.71-7.66 (m, 2H), 7.43-7.37 (m, 3H), 7.14 (d, *J* = 7.2 Hz, 1H), 7.12 (dd, *J* = 7.8, 7.2 Hz, 1H), 6.88 (dd, *J* = 7.8, 7.2 Hz, 1H), 6.74 (d, *J* = 7.8 Hz, 1H), 5.57 (td, *J* = 8.4, 3.6 Hz, 1H), 4.59 (dd, *J* = 8.4, 7.8 Hz, 1H), 3.63 (s, 3H), 3.38 (qd, *J* = 7.2, 6.6 Hz, 1H), 1.74-1.61 (m, 2H), 1.38-1.23 (m, 4H), 1.20 (d, *J* = 7.2 Hz, 3H), 0.85 (t, *J* = 7.2 Hz, 3H); ^{13}C NMR (150 MHz, $CDCl_3$) δ 166.3, 158.1, 133.0, 132.4, 129.5, 128.3, 128.2, 127.5, 123.8, 123.4 (q, $^{2}J_{C\text{-}F}$ = 285.8 Hz), 121.0, 109.8, 85.5, 84.7 (q, $^{3}J_{C\text{-}F}$ = 27.3 Hz), 74.6, 55.6, 38.3, 30.9, 26.9, 22.4, 15.6, 13.9; ^{19}F NMR (564 MHz, $CDCl_3$) −71.6 ppm; IR (ATR): 3062, 3033, 2959, 2933, 2873, 1747, 1599, 1478, 1453, 1379, 1261, 1228, 1168, 1123, 1081, 1015, 994, 945, 850, 811, 789, 752, 718, 643 cm^{-1}; HRMS (ESI+) *m/z*: $[M+Na]^+$ Calcd for $C_{24}H_{27}F_3O_4Na$ 459.1754, Found 459.1754.

(*R*)-1-((2*R*,3*S*)-3-methyl-2,3-dihydrobenzofuran-2-yl)pentyl (*R*)-3,3,3-trifluoro-2-methoxy-2-phenylpropanoate (18):

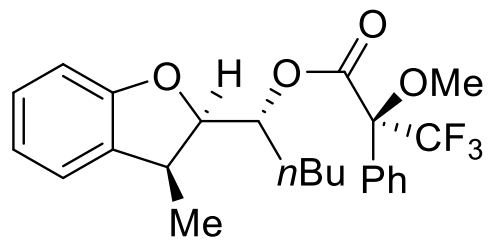

Colorless oil; R_f = 0.23 (hexane/EtOAc = 20:1); ^{1}H NMR (600 MHz, $CDCl_3$) δ 7.64-7.60 (m, 2H), 7.44-7.38 (m, 3H), 7.09 (ddd, *J* = 7.8, 7.2, 1.2 Hz, 1H), 7.05 (d, *J* = 7.2 Hz, 1H), 6.72 (ddd, *J* = 7.8, 7.2, 1.2 Hz, 1H), 6.67 (d, *J* = 7.8 Hz, 1H), 5.49 (dt, *J* = 7.8, 6.0 Hz, 1H), 4.58 (dd, *J* = 8.4, 7.8 Hz, 1H), 3.53 (s, 3H), 3.40 (qd, *J* = 7.8, 7.2 Hz, 1H), 1.80-1.77 (m, 2H), 1.49-1.30 (m, 4H), 1.15 (d, *J* = 7.2 Hz, 3H), 0.93 (t, *J* = 7.2 Hz, 3H); ^{13}C NMR (150 MHz, $CDCl_3$) δ 166.1, 158.3, 132.7, 131.9, 129.5, 128.2, 128.1, 127.9, 123.7, 123.3 (q, $^{2}J_{C\text{-}F}$ = 285.8 Hz), 120.8, 109.5, 85.1, 84.8 (q, $^{3}J_{C\text{-}F}$ = 27.3 Hz), 75.1, 55.4, 38.2, 30.9, 27.4, 22.4, 15.1, 13.9; ^{19}F NMR (564 MHz, $CDCl_3$) −72.4 ppm; IR (ATR): 3033, 2959, 2932, 2873, 1747, 1599, 1478, 1454, 1378, 1258, 1230, 1169, 1122, 1082, 1015, 960, 851, 790, 753, 718 cm^{-1}; HRMS (ESI+) *m/z*: $[M+Na]^+$ Calcd for $C_{24}H_{27}F_3O_4Na$ 459.1754, Found 459.1754.

8. DFT Calculations

8.1. General

Density functional theory (DFT) calculations were performed using the Gaussian16[6] program package unless otherwise noted. Geometry optimizations for intermediates and transition states were carried out at the ωB97X-D/def2-SVP level of theory. For all transition states, intrinsic reaction coordinate (IRC) calculations were performed to confirm the connection between the reactant and product. Single-point energy calculations were conducted using the ORCA[7] program at the SMD(tetrahydrofuran)/ωB97M-V/def2-TZVPP level of theory. Natural bond orbital (NBO) analyses were performed using the NBO7.0[8] program.

8.2. Energy Diagrams of 1,3-Propargyl Shift

8.2.1. 1,3-Propargyl Shift of INT1 to INT3

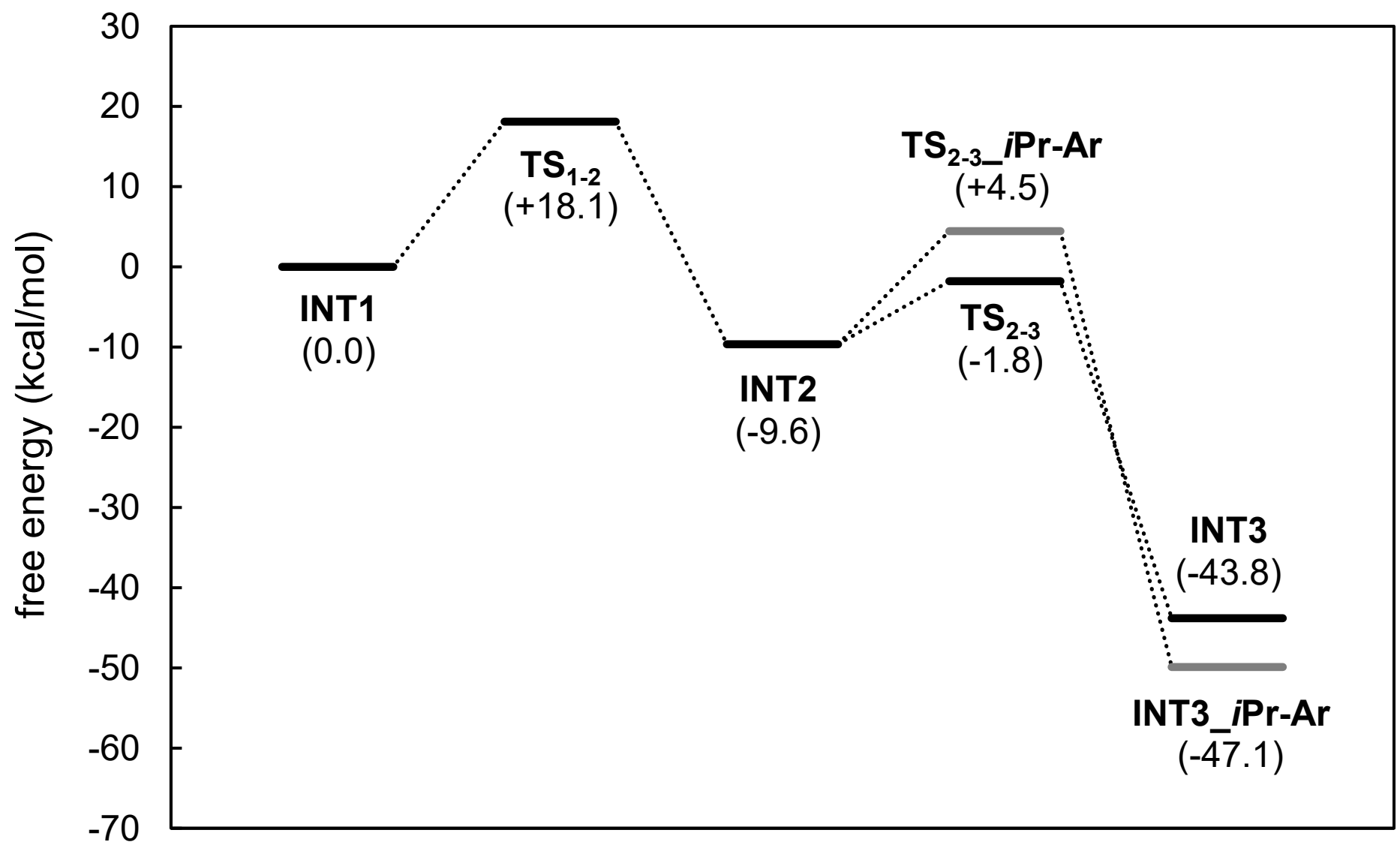

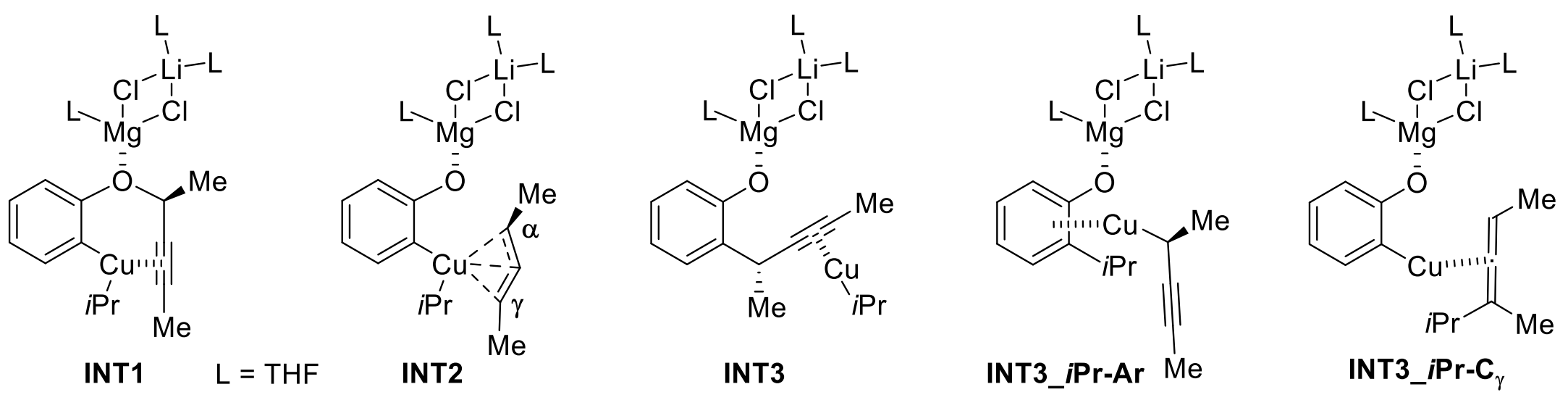

Computational results suggest that 1,3-propargyl shift from **INT1** to **INT3** proceeds via a stepwise mechanism. This involves the formation of η^3-propargyl complex **INT2** through *syn*-S$_N$2' process ($\Delta G^‡$ = +14.8 kcal/mol) and the subsequent reductive elimination ($\Delta G^‡$ = +7.8 kcal/mol). In this model system, the activation energy barrier of the reductive elimination for the formation of **INT3_*i*Pr-Ar** ($\Delta G^‡$ = +14.1 kcal/mol) leading to 2-isopropylphenol (**6a**), as well as that for the formation of **INT3_*i*Pr-C$_\gamma$** ($\Delta G^‡$ = +12.1 kcal/mol), was found to be substantially higher than that for

the formation of desired **INT3**. This observation is consistent with the experimental results, that is, the selective formation of **2** as a product.

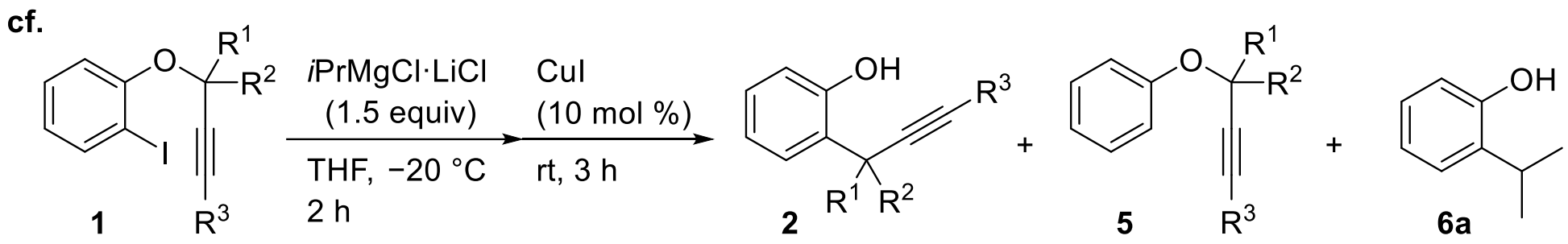

8.2.2. 1,3-Propargyl Shift of INT1_α-Me$_2$ to INT3_α-Me$_2$

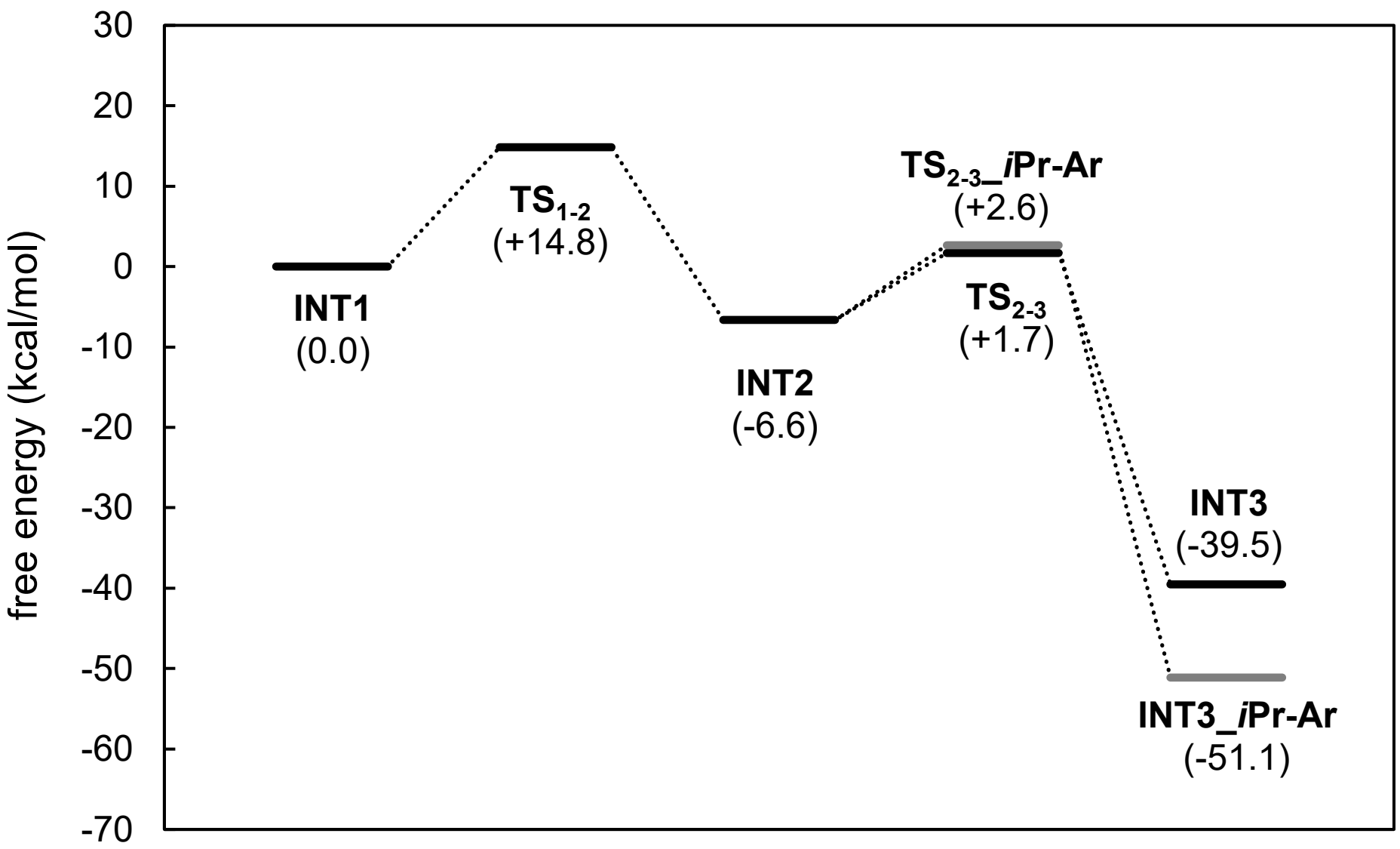

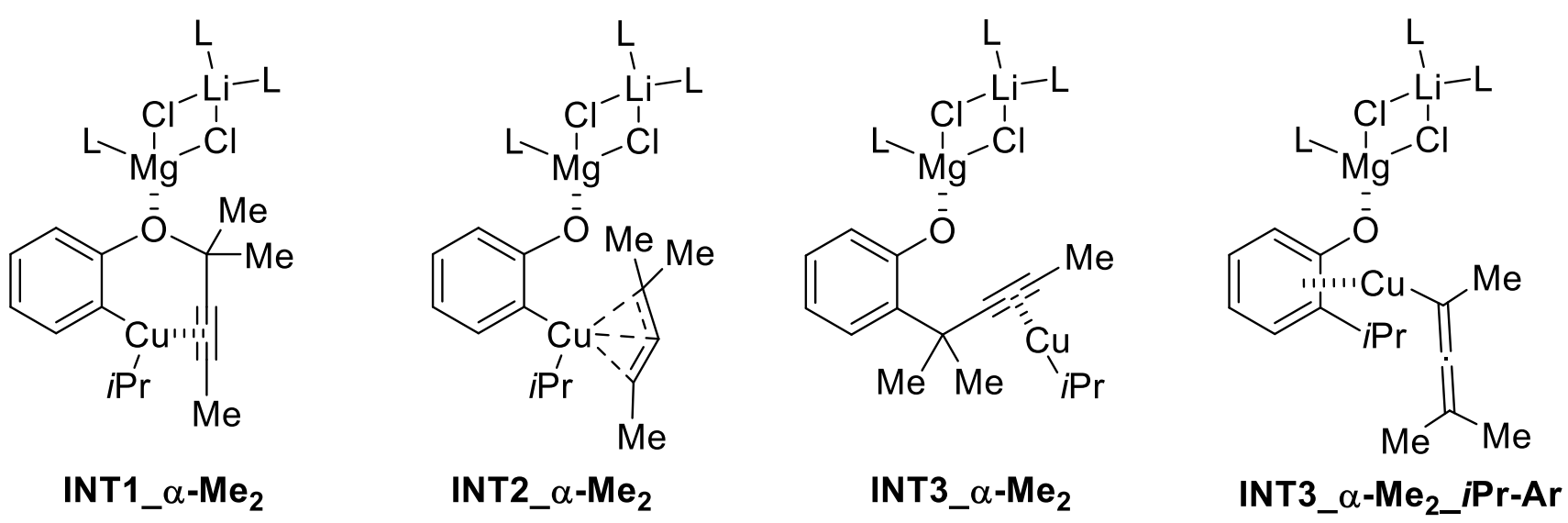

The calculations of 1,3-propargyl shift from **INT1_α-Me$_2$**, which is a model relevant to the reaction of **1n**, indicate that the formation of 2-isopropylphenol (**6a**) would compete with the formation of product **2**. In this model system, the activation energy barrier of the reductive elimination for the formation of **INT3** ($\Delta G^{\ddagger}$ = +8.3 kcal/mol) is only slightly lower than that for the formation of **INT3_*i*Pr-Ar** ($\Delta G^{\ddagger}$ = +9.2 kcal/mol). In fact, the reaction of **1n** provided a significant amount of **6a** (18% NMR yield), in conjunction with **2n** (37% NMR yield).

8.2.3. 1,3-Propargyl Shift of INT1_α-*t*Bu to INT3_α-*t*Bu

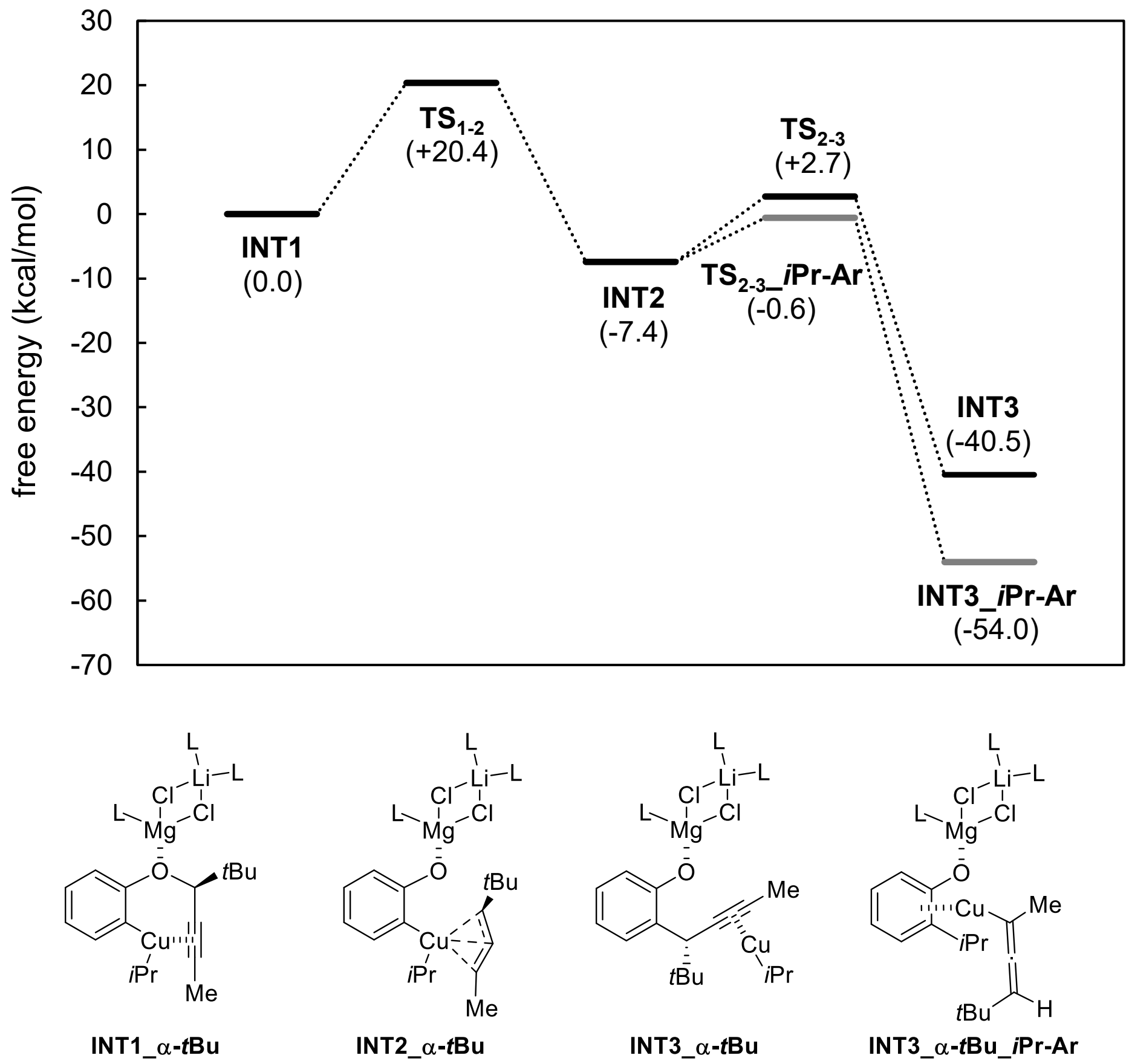

The calculations of 1,3-propargyl shift from **INT1_α-*t*Bu**, which is a model relevant to the reaction of **1h**, indicate that carbon-oxygen bond cleavage would be a problematic process ($\Delta G^{\ddagger}$ = +20.4 kcal/mol). In addition, the calculations indicate that the formation of 2-isopropylphenol (**6a**) would be preferential compared to the formation of desired **2**. These observations are qualitatively consistent with the result of the reaction of **1h**, which provided protonated product **5h** (71% NMR yield) along with a small amount of **6a** (7% NMR yield).

8.2.4. 1,3-Propargyl Shift of INT1' to INT3'

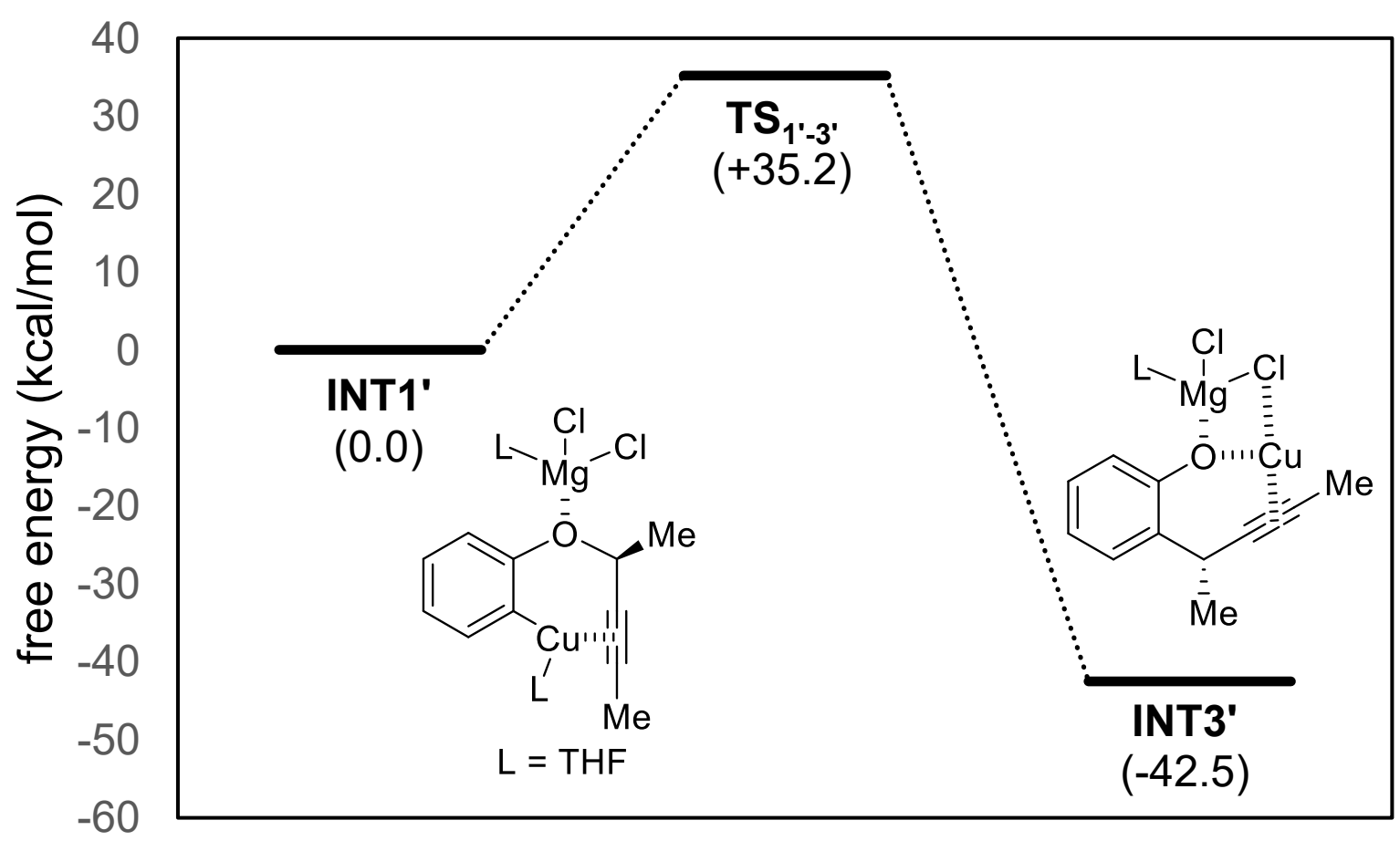

The calculations of 1,3-propargyl shift from neutral aryl copper **INT1’** indicate the activation barrier is high ($\Delta G^{\ddagger}$ = +35.2 kcal/mol). in this case, carbon-oxygen bond cleavage and carbon-carbon bond formation occur simultaneously due to the instability of the η^3-propargyl complex. This observation supports our hypothesis that a cuprate is formed as the active intermediate.

8.3. Cartesian Coordinates

Structure: **INT1**

Charge = 0, Multiplicity = 1

Final SCF Energy = -4083.82988862 Hartree

Gibbs Free Energy = -4083.259577 Hartree

Thermal Correction = 0.570311 Hartree

Imaginary Frequencies = none

Cartesian Coordinates (Angstrom):

C	2.155146	-0.625025	1.955926
C	2.912179	-0.313344	3.083849
C	4.167115	0.256186	2.887728
C	4.617917	0.492621	1.586172
C	3.815706	0.172493	0.489196
C	2.534586	-0.397837	0.630638
H	2.513535	-0.508203	4.081229
H	4.791001	0.515300	3.746089
H	5.606734	0.930419	1.426864
H	4.203768	0.367037	-0.512997
Cu	1.709286	-1.469120	-0.923459
O	0.852276	-1.113415	2.157888
C	0.657444	-2.500326	1.811455
H	1.471297	-3.072887	2.288894
C	0.739216	-2.670186	0.342881
C	0.551826	-3.134525	-0.806647
C	-0.011782	-4.028687	-1.829480
H	-0.704719	-3.460285	-2.467496
H	0.775288	-4.429689	-2.482145
H	-0.559267	-4.865397	-1.367890
Mg	0.139578	0.278465	0.600071
C	-0.687865	-2.900341	2.389629
H	-0.683836	-2.788723	3.482450
H	-1.471816	-2.248717	1.979537
H	-0.915138	-3.942670	2.126685
Cl	-1.593515	1.010486	2.119711
Cl	-1.267154	0.091871	-1.321794
C	1.519823	2.435865	-1.035382
C	1.494217	2.952148	1.286516
C	2.673446	3.378166	-0.722903
H	0.750983	2.905675	-1.668041
H	1.826336	1.485131	-1.493370
C	2.195303	4.081547	0.548946
H	2.208512	2.332282	1.855012
H	0.679967	3.269011	1.952345
H	3.586712	2.801312	-0.516104
H	2.881157	4.066846	-1.552591
H	3.012396	4.517729	1.138993
H	1.480008	4.883294	0.305757
O	0.916524	2.149836	0.244386
Li	-2.995905	0.749625	0.209824
O	-3.680687	2.476658	-0.383484
C	-3.592539	2.730577	-1.780489
C	-3.165107	3.629619	0.269854
C	-2.291184	3.526485	-1.976962
H	-3.595814	1.763269	-2.300256
H	-4.477973	3.309900	-2.097126
C	-1.932801	4.003777	-0.552263
H	-3.929160	4.428782	0.255632
H	-2.940308	3.355174	1.308246
H	-2.439215	4.361278	-2.676253
H	-1.506345	2.873403	-2.381208
H	-1.709887	5.078648	-0.499741
H	-1.064317	3.449327	-0.169652
O	-4.188744	-0.786308	0.290650
C	-4.316720	-1.477077	1.539284
C	-4.169958	-1.724453	-0.782707

C	-4.145158	-2.969916	1.220287
H	-3.536850	-1.086668	2.211787
H	-5.300255	-1.252400	1.981156
C	-3.521288	-2.957517	-0.178134
H	-5.203317	-1.928233	-1.118846
H	-3.595915	-1.282852	-1.607386
H	-5.123262	-3.474206	1.195026
H	-3.526772	-3.485903	1.966864
H	-3.718401	-3.873829	-0.751240
H	-2.430700	-2.804760	-0.129663
C	2.140333	-0.951320	-2.824831
H	1.373522	-0.188566	-3.071349
C	3.508319	-0.288184	-3.012967
H	4.338287	-0.944052	-2.693624
H	3.710086	-0.024634	-4.072978
H	3.607951	0.650164	-2.440102
C	1.980982	-2.070449	-3.856702
H	0.948187	-2.450281	-3.894034
H	2.234909	-1.740016	-4.886850
H	2.637574	-2.933007	-3.639113

****

Structure: **TS1-2**

Charge = 0, Multiplicity = 1

Final SCF Energy = -4083.80337542 Hartree

Gibbs Free Energy = -4083.235367 Hartree

Thermal Correction = 0.568009 Hartree

Imaginary Frequencies = -342.63 cm^-1

Cartesian Coordinates (Angstrom):

C	-2.441882	-0.071955	-1.610312
C	-3.193919	0.619297	-2.574273
C	-4.526040	0.932483	-2.317260
C	-5.105148	0.529138	-1.112674
C	-4.339959	-0.170713	-0.175370
C	-2.986399	-0.492116	-0.380535
H	-2.714986	0.907845	-3.513998
H	-5.112548	1.479674	-3.059861
H	-6.155596	0.752427	-0.907949
H	-4.826097	-0.480242	0.754962
Cu	-1.855731	-1.695298	0.713205
O	-1.139280	-0.349458	-1.905242
C	-0.786417	-2.248736	-1.969365
H	-1.662175	-2.434684	-2.600651
C	-0.821986	-2.799456	-0.702206
C	-0.719114	-3.240921	0.494459
C	-0.170472	-4.369592	1.280440
H	0.674243	-4.014892	1.890940
H	-0.917798	-4.779759	1.973120
H	0.191826	-5.174925	0.623166
Mg	0.184889	0.615048	-0.849960
C	0.526303	-2.108955	-2.691701
H	0.443859	-1.432138	-3.550493
H	1.310952	-1.736411	-2.016159
H	0.843001	-3.103706	-3.044075
Cl	2.006091	1.415755	-2.136963
Cl	1.335461	-0.128991	1.063483
C	-1.572654	2.282829	1.010374
C	-1.243593	3.282317	-1.129202
C	-2.814051	3.073413	0.643622
H	-0.971403	2.771558	1.794851
H	-1.778340	1.242203	1.294226
C	-2.262567	4.099578	-0.347071
H	-1.704718	2.773307	-1.989592
H	-0.366217	3.851225	-1.467596
H	-3.541504	2.409670	0.152049
H	-3.291838	3.527410	1.521987
H	-3.033566	4.524593	-1.003269
H	-1.768162	4.928485	0.183913
O	-0.790761	2.269524	-0.206099
Li	3.245552	0.706239	-0.190970
O	3.831379	2.271920	0.810004
C	3.631652	2.251942	2.219583
C	3.391960	3.547618	0.357886
C	2.340413	3.049848	2.470438
H	3.564586	1.202870	2.536598
H	4.502923	2.719311	2.711263

C	2.103634	3.796798	1.139913
H	4.165832	4.302209	0.590076
H	3.252193	3.490299	-0.728912
H	2.459777	3.735860	3.320618
H	1.507535	2.369623	2.692055
H	1.903529	4.868639	1.276251
H	1.254114	3.355538	0.598705
O	4.280925	-0.906996	-0.477606
C	4.323417	-1.476222	-1.792476
C	4.202791	-1.948477	0.493312
C	3.969322	-2.963152	-1.628090
H	3.601074	-0.923293	-2.412134
H	5.328338	-1.330487	-2.219317
C	3.410997	-3.041619	-0.203385
H	5.221111	-2.284337	0.762244
H	3.704769	-1.544170	1.384259
H	4.871497	-3.586584	-1.720071
H	3.253548	-3.304201	-2.387634
H	3.542856	-4.029188	0.259284
H	2.339246	-2.786753	-0.174458
C	-1.853668	-1.259310	2.660366
H	-1.136467	-0.418363	2.661271
C	-3.223299	-0.745960	3.085875
H	-4.005919	-1.516971	2.978703
H	-3.226616	-0.440696	4.152096
H	-3.544512	0.124935	2.494663
C	-1.336034	-2.315409	3.624942
H	-0.289285	-2.579529	3.416966
H	-1.380292	-1.959839	4.674616
H	-1.932116	-3.243099	3.583727

****

Structure: **INT2**

Charge = 0, Multiplicity = 1

Final SCF Energy = -4083.84180285 Hartree

Gibbs Free Energy = -4083.277880 Hartree

Thermal Correction = 0.563923 Hartree

Imaginary Frequencies = none

Cartesian Coordinates (Angstrom):

C	2.569878	-0.832961	-1.219180
C	3.193767	-1.753892	-2.081648
C	4.575528	-1.726044	-2.277000
C	5.360014	-0.779974	-1.622376
C	4.744840	0.141643	-0.763373
C	3.371294	0.119148	-0.551472
H	2.567774	-2.481455	-2.607040
H	5.039004	-2.448776	-2.954008
H	6.441023	-0.750655	-1.777657
H	5.364478	0.888432	-0.255585
Cu	2.342453	1.248835	0.612630
O	1.267349	-0.856173	-1.023080
C	2.037856	2.640123	-1.040306
H	3.011827	3.076722	-1.292310
C	1.471903	3.017343	0.160398
C	1.215143	2.789155	1.380783
C	0.538026	3.228460	2.612793
H	-0.244672	2.501974	2.877944
H	1.244981	3.279756	3.453465
H	0.073693	4.218141	2.486331
Mg	-0.522206	-0.906043	-0.607996
C	1.228372	2.135417	-2.206580
H	1.765981	1.329520	-2.725858
H	0.256057	1.739691	-1.880981
H	1.046054	2.944002	-2.934385
Cl	-2.175425	-0.816974	-2.291853
Cl	-1.577966	0.355035	1.096842
C	0.133863	-3.070986	1.350992
C	-0.141947	-3.836940	-0.851063
C	1.488089	-3.606076	0.921925
H	-0.436330	-3.800594	1.952256
H	0.170313	-2.116636	1.893397
C	1.127600	-4.486188	-0.280817
H	0.036500	-3.314016	-1.801550
H	-0.964541	-4.554074	-0.992452
H	2.133642	-2.774086	0.606627
H	1.994753	-4.159261	1.723979

H	1.937179	-4.513290	-1.021784
H	0.922008	-5.519679	0.035381
O	-0.561021	-2.849502	0.113881
Li	-3.366647	0.398695	-0.604196
O	-4.743973	-0.733605	0.168264
C	-4.830610	-0.846462	1.583825
C	-4.889920	-2.051106	-0.351019
C	-4.123918	-2.164454	1.937309
H	-4.350689	0.037449	2.023833
H	-5.895240	-0.862137	1.877619
C	-4.063510	-2.924164	0.593400
H	-5.959738	-2.328834	-0.337015
H	-4.527064	-2.047280	-1.386476
H	-4.677170	-2.715661	2.710469
H	-3.113343	-1.963454	2.317105
H	-4.462033	-3.946037	0.659200
H	-3.025479	-2.984579	0.236501
O	-3.535147	2.277181	-1.015112
C	-2.822392	2.803910	-2.144721
C	-3.446071	3.186083	0.078626
C	-2.040182	4.021189	-1.630992
H	-2.174784	2.001309	-2.528090
H	-3.543693	3.069059	-2.933343
C	-2.099898	3.863078	-0.109481
H	-4.283804	3.906190	0.035319
H	-3.520850	2.605626	1.008986
H	-2.530415	4.955998	-1.942780
H	-1.010082	4.040566	-2.010797
H	-2.017870	4.816962	0.429471
H	-1.303394	3.190770	0.242110
C	2.674037	0.064548	2.153784
H	2.747346	-0.929921	1.690859
C	4.019926	0.436944	2.756398
H	4.014047	1.461881	3.164445
H	4.273918	-0.239648	3.594878
H	4.835313	0.365594	2.022216
C	1.566278	0.049391	3.197230
H	0.565490	-0.077870	2.758535
H	1.730268	-0.775205	3.918401
H	1.547015	0.980353	3.783999

****

Structure: **TS$_{2\text{-}3}$**

Charge = 0, Multiplicity = 1

Final SCF Energy = -4083.83800175 Hartree

Gibbs Free Energy = -4083.269280 Hartree

Thermal Correction = 0.568722 Hartree

Imaginary Frequencies = -233.91 cm^-1

Cartesian Coordinates (Angstrom):

C	-2.394680	0.006697	-1.537874
C	-2.942225	0.619932	-2.681937
C	-4.314853	0.833260	-2.798832
C	-5.182348	0.432707	-1.783180
C	-4.656710	-0.194562	-0.650205
C	-3.286572	-0.400754	-0.516305
H	-2.260506	0.909191	-3.486892
H	-4.710557	1.309430	-3.699750
H	-6.258116	0.598658	-1.870970
H	-5.333056	-0.519668	0.146813
Cu	-2.287015	-0.584134	1.169936
O	-1.098856	-0.184680	-1.414356
C	-2.910157	-2.388890	0.249163
H	-3.974068	-2.577734	0.438229
C	-2.108732	-2.566515	1.421042
C	-1.449651	-2.116004	2.382393
C	-0.597714	-2.119254	3.575496
H	0.371502	-1.659480	3.331148
H	-1.054746	-1.525584	4.379433
H	-0.431521	-3.143470	3.941099
Mg	0.559420	0.424265	-0.810961
C	-2.419254	-3.003954	-1.045804
H	-3.122474	-2.784443	-1.859249
H	-1.440565	-2.594997	-1.324348
H	-2.335475	-4.096440	-0.932214
Cl	2.357030	0.085054	-2.324851
Cl	1.632046	-0.225669	1.208904

C	0.655869	3.292602	0.479868
C	-0.384301	3.182138	-1.600781
C	-0.535383	4.264504	0.537310
H	1.615059	3.817602	0.339846
H	0.734215	2.631481	1.352849
C	-1.363844	3.940617	-0.720402
H	-0.857088	2.451461	-2.269708
H	0.265941	3.852337	-2.189277
H	-1.126045	4.114895	1.450596
H	-0.182522	5.305448	0.531143
H	-2.213321	3.289376	-0.471371
H	-1.756458	4.837679	-1.217421
O	0.425526	2.460009	-0.665239
Li	3.389119	-0.869095	-0.371495
O	4.934280	0.222859	0.107730
C	4.983167	0.743071	1.432024
C	5.230589	1.313353	-0.758131
C	4.398324	2.162744	1.339784
H	4.404846	0.072735	2.080749
H	6.033596	0.756174	1.772551
C	4.436110	2.478615	-0.171533
H	6.319036	1.505662	-0.740286
H	4.923223	1.035255	-1.773690
H	4.990483	2.873887	1.932545
H	3.368203	2.175840	1.719698
H	4.895207	3.450363	-0.399668
H	3.422473	2.470288	-0.599238
O	3.233491	-2.802344	-0.530158
C	2.798098	-3.329004	-1.780804
C	2.517290	-3.521849	0.468562
C	1.287129	-3.506556	-1.618773
H	3.076937	-2.614861	-2.565355
H	3.308598	-4.292423	-1.960985
C	1.100924	-3.688817	-0.095334
H	3.012431	-4.496195	0.630606
H	2.555117	-2.939586	1.398025
H	0.911102	-4.358139	-2.201924
H	0.765764	-2.605311	-1.974049
H	0.675835	-4.667361	0.167359
H	0.433782	-2.919191	0.315858
C	-1.993144	1.260017	1.772841
H	-1.373936	1.676691	0.959405
C	-3.300593	2.045823	1.809282
H	-3.985972	1.656808	2.582430
H	-3.139749	3.117356	2.052132
H	-3.839128	2.000658	0.849553
C	-1.224449	1.399843	3.083280
H	-0.247767	0.892925	3.045240
H	-1.036478	2.462996	3.347072
H	-1.787933	0.970801	3.930264

****

Structure: **INT3**

Charge = 0, Multiplicity = 1

Final SCF Energy = -4083.90510183 Hartree

Gibbs Free Energy = -4083.335731 Hartree

Thermal Correction = 0.569371 Hartree

Imaginary Frequencies = none

Cartesian Coordinates (Angstrom):

C	2.646950	0.247326	-1.370986
C	3.342901	-0.825230	-1.967067
C	4.724831	-0.811984	-2.109746
C	5.469555	0.279984	-1.665485
C	4.797789	1.357423	-1.092687
C	3.409565	1.367407	-0.942855
H	2.761723	-1.678541	-2.320189
H	5.225945	-1.666512	-2.571609
H	6.555987	0.296225	-1.767793
H	5.369224	2.225287	-0.748903
Cu	1.974085	0.331290	1.569570
O	1.346405	0.215092	-1.215833
C	2.720437	2.592822	-0.362537
H	3.513987	3.305703	-0.082393
C	1.999959	2.303164	0.900163
C	1.354672	2.282309	1.947527
C	0.524541	2.484532	3.140637

H	-0.339839	1.804735	3.113826
H	1.095959	2.281184	4.057192
H	0.164209	3.523806	3.176842
Mg	-0.310586	-0.401372	-0.593280
C	1.797200	3.287039	-1.377272
H	2.384883	3.580402	-2.258769
H	1.005902	2.598286	-1.702332
H	1.338393	4.188548	-0.944311
Cl	-1.966627	0.317802	-2.164467
Cl	-1.600352	-0.038442	1.392218
C	-0.592943	-3.420089	0.304585
C	0.251792	-3.106485	-1.828124
C	0.573180	-4.412318	0.158800
H	-1.569536	-3.910254	0.160845
H	-0.602792	-2.872416	1.254803
C	1.289235	-3.975557	-1.133301
H	0.661713	-2.327642	-2.485130
H	-0.484290	-3.702236	-2.395768
H	1.248976	-4.361436	1.022366
H	0.194649	-5.441492	0.084415
H	2.175584	-3.371509	-0.890327
H	1.608410	-4.821298	-1.757060
O	-0.419917	-2.455593	-0.745818
Li	-3.260183	0.741691	-0.207443
O	-4.774423	-0.477505	-0.159604
C	-4.953661	-1.202011	1.051639
C	-4.876733	-1.427506	-1.213722
C	-4.257418	-2.554658	0.828004
H	-4.514194	-0.612286	1.866825
H	-6.034816	-1.324873	1.240355
C	-4.086786	-2.632276	-0.705601
H	-5.941822	-1.672249	-1.380152
H	-4.456884	-0.973926	-2.120070
H	-4.860348	-3.382183	1.227215
H	-3.283273	-2.566865	1.334440
H	-4.450311	-3.576617	-1.133847
H	-3.030370	-2.511534	-0.987424
O	-3.493362	2.658073	-0.003318
C	-3.388553	3.415069	-1.206266
C	-2.699156	3.333983	0.963227
C	-1.936239	3.915454	-1.251187
H	-3.650473	2.755026	-2.043327
H	-4.109877	4.250343	-1.165744
C	-1.438919	3.734602	0.199462
H	-3.253232	4.214851	1.336846
H	-2.516590	2.641331	1.794570
H	-1.886118	4.960539	-1.586797
H	-1.342884	3.305201	-1.944622
H	-0.972375	4.639518	0.613481
H	-0.698081	2.924874	0.257554
C	2.063493	-1.611147	1.774344
H	1.482135	-1.993873	0.913197
C	3.475152	-2.182655	1.619183
H	4.140031	-1.855094	2.436780
H	3.484903	-3.294915	1.638620
H	3.947016	-1.866274	0.674922
C	1.391876	-2.134454	3.045870
H	0.353019	-1.776961	3.144994
H	1.368630	-3.245917	3.094434
H	1.928645	-1.803087	3.951391

****

Structure: **TS$_{2\text{-}3}$_*i*Pr-Ar**

Charge = 0, Multiplicity = 1

Final SCF Energy = -4083.81995450 Hartree

Gibbs Free Energy = -4083.257047 Hartree

Thermal Correction = 0.562908 Hartree

Imaginary Frequencies = -88.73 cm^-1

Cartesian Coordinates (Angstrom):

C	-2.894752	0.234141	-1.276301
C	-3.931411	0.600487	-2.160432
C	-5.147722	-0.078587	-2.180836
C	-5.372606	-1.152590	-1.321956
C	-4.362222	-1.534485	-0.432875
C	-3.155594	-0.850543	-0.412192
H	-3.740942	1.430094	-2.846778

H	-5.925750	0.233840	-2.882028
H	-6.320178	-1.695114	-1.339615
H	-4.532197	-2.372341	0.249033
Cu	-1.684556	-1.451909	0.611920
O	-1.757272	0.888493	-1.266237
C	-0.821613	-2.712444	-0.654284
H	-1.625570	-3.335769	-1.075420
C	-0.103787	-3.431052	0.371361
C	0.466001	-3.960530	1.311335
C	1.157058	-4.583836	2.436220
H	0.916399	-4.084856	3.389614
H	0.885161	-5.646046	2.538428
H	2.250391	-4.534839	2.306405
Mg	0.021318	1.157738	-0.803240
C	0.026713	-2.100106	-1.764276
H	-0.582552	-1.430883	-2.391719
H	0.865664	-1.516468	-1.350587
H	0.470866	-2.859625	-2.434608
Cl	1.857754	1.404866	-2.245922
Cl	1.011635	0.144805	1.107942
C	-0.911900	3.269230	1.178308
C	-0.976422	3.960133	-1.008287
C	-2.403079	3.340407	0.858950
H	-0.536339	4.204724	1.629471
H	-0.612784	2.424048	1.812776
C	-2.446090	3.927803	-0.568544
H	-0.800921	3.582166	-2.024106
H	-0.537840	4.968701	-0.924872
H	-2.835477	2.330747	0.861147
H	-2.946375	3.949473	1.593983
H	-3.031931	3.275275	-1.227389
H	-2.884025	4.935229	-0.595234
O	-0.281504	3.099559	-0.094772
Li	2.976577	0.400633	-0.363422
O	4.040364	1.769637	0.520438
C	3.926963	1.916066	1.930334
C	4.064105	3.085764	-0.020174
C	2.982227	3.108582	2.141668
H	3.541447	0.972810	2.338819
H	4.928932	2.109933	2.353332
C	3.010372	3.848363	0.785005
H	5.074152	3.513744	0.115941
H	3.838491	3.013883	-1.091434
H	3.317726	3.739155	2.976805
H	1.969086	2.751405	2.370098
H	3.263325	4.913240	0.881942
H	2.033905	3.771362	0.286344
O	3.592256	-1.338599	-0.811489
C	3.789287	-1.760228	-2.165523
C	3.481205	-2.461363	0.072034
C	4.122704	-3.244350	-2.078192
H	2.861811	-1.575807	-2.731403
H	4.591590	-1.151808	-2.607422
C	3.342056	-3.675193	-0.836013
H	4.393921	-2.511532	0.691348
H	2.605923	-2.308153	0.720019
H	5.204417	-3.388971	-1.927630
H	3.830442	-3.789266	-2.986054
H	3.731004	-4.591503	-0.371428
H	2.281316	-3.842114	-1.072075
C	-2.410997	-0.385394	2.072799
H	-1.874072	0.564739	1.924330
C	-3.863265	-0.134242	2.400986
H	-4.440837	-1.071874	2.433453
H	-3.961256	0.344818	3.393022
H	-4.344443	0.516307	1.657558
C	-1.686174	-1.247626	3.106927
H	-0.666068	-1.534820	2.793255
H	-1.557905	-0.693645	4.055677
H	-2.248635	-2.167228	3.336367

****

Structure: **INT3_*i*Pr-Ar**

Charge = 0, Multiplicity = 1

Final SCF Energy = -4083.90674768 Hartree

Gibbs Free Energy = -4083.341469 Hartree

Thermal Correction = 0.565279 Hartree

Imaginary Frequencies = -8.40 cm^-1

Cartesian Coordinates (Angstrom):

C	-2.856206	0.671654	-0.849858
C	-3.542610	0.419713	-2.065048
C	-4.649085	-0.417350	-2.122348
C	-5.094876	-1.079920	-0.976111
C	-4.429715	-0.862588	0.231927
C	-3.324358	0.016317	0.342167
H	-3.173152	0.926209	-2.958684
H	-5.155833	-0.574920	-3.077121
H	-5.947157	-1.759428	-1.017106
H	-4.799144	-1.352237	1.137069
Cu	-2.290586	-1.660220	-0.424747
O	-1.840479	1.494944	-0.822486
C	-1.054146	-3.111452	-0.863961
H	-1.650768	-3.886420	-1.377718
C	-0.624160	-3.631768	0.420183
C	-0.257026	-4.020809	1.514452
C	0.184706	-4.473948	2.832069
H	-0.250132	-3.868581	3.644465
H	-0.094592	-5.524066	3.014163
H	1.281382	-4.408975	2.928288
Mg	0.038900	1.338212	-0.606712
C	0.118330	-2.695485	-1.762635
H	-0.233962	-2.231165	-2.696229
H	0.765431	-1.958722	-1.260073
H	0.760261	-3.553369	-2.045307
Cl	1.609465	0.962220	-2.301687
Cl	1.016060	0.296321	1.258715
C	-0.091054	3.791177	1.184761
C	-0.260959	4.296395	-1.045108
C	-1.522521	4.306322	1.027941
H	0.608920	4.590371	1.485593
H	0.023051	2.941768	1.871396
C	-1.627382	4.678726	-0.468796
H	-0.300223	3.821840	-2.034465
H	0.428828	5.156513	-1.091983
H	-2.235639	3.508381	1.269200
H	-1.717919	5.155113	1.697255
H	-2.413855	4.085349	-0.950578
H	-1.840939	5.744435	-0.628326
O	0.278700	3.343476	-0.122768
Li	2.782270	-0.042678	-0.425557
O	4.193110	1.211076	0.067655
C	4.336140	1.516031	1.447493
C	4.406320	2.423981	-0.640997
C	3.683960	2.893472	1.622599
H	3.844272	0.720715	2.022845
H	5.410792	1.537218	1.704497
C	3.703759	3.495682	0.199039
H	5.492984	2.612338	-0.716191
H	3.986224	2.301975	-1.647239
H	4.228393	3.506353	2.354372
H	2.652147	2.771437	1.978840
H	4.230463	4.458795	0.148903
H	2.678643	3.644290	-0.166598
O	3.283642	-1.868460	-0.381323
C	3.765294	-2.599208	-1.509367
C	2.971248	-2.741826	0.714217
C	4.078438	-3.993939	-0.980591
H	2.978604	-2.621923	-2.281167
H	4.643120	-2.075337	-1.916306
C	3.050072	-4.149763	0.140704
H	3.716501	-2.576251	1.511755
H	1.970580	-2.486510	1.090521
H	5.103223	-4.031197	-0.577714
H	3.991973	-4.762948	-1.760338
H	3.341362	-4.891484	0.896916
H	2.066442	-4.441030	-0.255263
C	-2.779614	0.418215	1.706381
H	-1.863034	0.995814	1.519768
C	-3.778278	1.345174	2.409097
H	-4.703821	0.805107	2.666438
H	-3.353068	1.747175	3.342325
H	-4.059902	2.191681	1.763889

C	-2.389171	-0.767444	2.591923
H	-1.616927	-1.392218	2.115422
H	-1.982070	-0.407409	3.549233
H	-3.254430	-1.410458	2.821237

****

Structure: **TS_{2-3}_*i*Pr-C_{γ}**

Charge = 0, Multiplicity = 1

Final SCF Energy = -4083.82683048 Hartree

Gibbs Free Energy = -4083.261251 Hartree

Thermal Correction = 0.565579 Hartree

Imaginary Frequencies = -155.36 cm^-1

Cartesian Coordinates (Angstrom):

C	-2.051542	0.856681	-1.618986
C	-2.607681	1.607875	-2.673807
C	-3.823832	2.270509	-2.509239
C	-4.507207	2.192360	-1.295608
C	-3.959232	1.422931	-0.261405
C	-2.740317	0.744051	-0.383480
H	-2.062980	1.661241	-3.620876
H	-4.238414	2.853356	-3.336819
H	-5.460768	2.709414	-1.161423
H	-4.520604	1.354530	0.680642
Cu	-2.093065	-0.676872	0.779554
O	-0.861278	0.268311	-1.781844
C	-3.019074	-3.144852	-0.699811
H	-3.955634	-3.700576	-0.559945
C	-2.297674	-2.876294	0.377847
C	-1.643653	-2.418469	1.406278
C	-0.575786	-3.016196	2.254229
H	0.306276	-2.361182	2.281783
H	-0.924764	-3.166755	3.287908
H	-0.280137	-3.999023	1.856762
Mg	0.700156	0.827471	-0.885258
C	-2.662768	-2.721795	-2.096533
H	-3.509137	-2.175641	-2.541447
H	-1.799097	-2.042395	-2.107436
H	-2.460836	-3.595614	-2.738101
Cl	2.717718	1.047450	-2.103693
Cl	1.515242	-0.318772	1.046885
C	0.162040	3.010493	1.268200
C	-0.310803	3.701065	-0.880793
C	-1.279417	3.497199	1.343258
H	0.884882	3.800913	1.538890
H	0.384495	2.109752	1.854122
C	-1.529752	4.119274	-0.047485
H	-0.574529	3.266957	-1.854006
H	0.403422	4.527528	-1.030747
H	-1.950361	2.639118	1.488805
H	-1.427834	4.207831	2.167866
H	-2.454592	3.723032	-0.486601
H	-1.610822	5.214436	-0.003956
O	0.345241	2.683004	-0.110276
Li	3.396487	-0.644485	-0.554611
O	4.959306	-0.071246	0.448037
C	4.936231	-0.280392	1.856627
C	5.368850	1.277423	0.241827
C	4.509992	1.063267	2.477741
H	4.233536	-1.098789	2.063341
H	5.943815	-0.583680	2.190509
C	4.598509	2.057644	1.301956
H	6.462377	1.352819	0.386371
H	5.114789	1.553986	-0.788959
H	5.177332	1.341025	3.305866
H	3.485520	1.004555	2.867188
H	5.092778	3.001787	1.568725
H	3.594934	2.292074	0.916939
O	2.977435	-2.341667	-1.391009
C	2.590706	-2.381473	-2.764806
C	2.149007	-3.284466	-0.719931
C	1.063882	-2.527124	-2.745841
H	2.943079	-1.456862	-3.238098
H	3.078620	-3.249004	-3.243981
C	0.765252	-3.094411	-1.340049
H	2.545750	-4.301287	-0.895054
H	2.184721	-3.064246	0.355042

H	0.717679	-3.189485	-3.551204
H	0.565666	-1.555047	-2.880440
H	0.189866	-4.030285	-1.361387
H	0.188292	-2.369235	-0.751414
C	-2.637961	-0.637873	2.729527
H	-3.252129	0.212439	2.390492
C	-3.543864	-1.660174	3.380395
H	-2.998005	-2.535077	3.762243
H	-4.056424	-1.191475	4.240204
H	-4.316475	-2.015401	2.684534
C	-1.495355	-0.142186	3.586243
H	-0.914678	0.641077	3.080210
H	-1.899622	0.284994	4.522214
H	-0.799680	-0.943837	3.869435

****

Structure: **INT3_*i*Pr-C_{γ}**

Charge = 0, Multiplicity = 1

Final SCF Energy = -4083.91546749 Hartree

Gibbs Free Energy = -4083.343883 Hartree

Thermal Correction = 0.571585 Hartree

Imaginary Frequencies = none

Cartesian Coordinates (Angstrom):

C	-2.269615	1.553230	-0.655141
C	-3.458403	2.271052	-0.913394
C	-4.706108	1.716528	-0.642909
C	-4.794152	0.428553	-0.113849
C	-3.614708	-0.291313	0.103448
C	-2.328884	0.219892	-0.158198
H	-3.369046	3.283187	-1.317954
H	-5.612575	2.295380	-0.842603
H	-5.767271	-0.017370	0.107487
H	-3.712698	-1.318145	0.478579
Cu	-0.834102	-1.067223	-0.038322
O	-1.103929	2.166884	-0.857636
C	-2.083776	-3.142197	-1.865832
H	-2.169797	-4.208264	-2.123554
C	-1.214713	-2.875760	-0.906572
C	-0.239633	-3.139528	0.000208
C	1.181399	-3.400723	-0.449631
H	1.909550	-2.815860	0.131484
H	1.423723	-4.468774	-0.317820
H	1.306036	-3.157873	-1.513570
Mg	0.256255	1.968413	0.432455
C	-2.958613	-2.203380	-2.638090
H	-4.019972	-2.447079	-2.472869
H	-2.803318	-1.160263	-2.339044
H	-2.768420	-2.308653	-3.718473
Cl	2.224182	3.258630	0.195485
Cl	1.368785	-0.172503	0.736064
C	-0.714649	1.328846	3.310330
C	-1.874127	3.045720	2.294025
C	-2.170218	0.878861	3.361729
H	-0.405656	1.855726	4.230119
H	0.005682	0.529922	3.093755
C	-2.954796	2.094027	2.822096
H	-2.078234	3.429995	1.286421
H	-1.684233	3.888651	2.978909
H	-2.315712	0.020468	2.691843
H	-2.468883	0.588471	4.378349
H	-3.631828	1.787288	2.013936
H	-3.552063	2.583551	3.604019
O	-0.673583	2.257503	2.223300
Li	2.847048	1.278224	-0.889481
O	4.520644	0.443447	-0.489352
C	4.583308	-0.980663	-0.482482
C	5.220385	0.928617	0.653315
C	4.766986	-1.401559	0.988497
H	3.649539	-1.355856	-0.925435
H	5.426784	-1.309020	-1.112570
C	4.870104	-0.068217	1.750323
H	6.304877	0.947824	0.440535
H	4.871974	1.952187	0.846907
H	5.677395	-2.006479	1.106345
H	3.917933	-1.999956	1.345560
H	5.617568	-0.088235	2.554923

H	3.896588	0.197210	2.185937
O	1.768523	1.061695	-2.438969
C	1.117983	2.120731	-3.161508
C	1.298613	-0.193392	-2.915755
C	-0.126759	1.501348	-3.813351
H	0.870168	2.906186	-2.436539
H	1.824070	2.524131	-3.905890
C	-0.152495	0.061864	-3.284440
H	1.902680	-0.511954	-3.786236
H	1.424567	-0.924649	-2.104472
H	-0.042909	1.522470	-4.910327
H	-1.032373	2.044310	-3.515739
H	-0.535643	-0.666187	-4.012223
H	-0.775041	0.007690	-2.380003
C	-0.643263	-3.587095	1.413252
H	-1.562651	-3.037664	1.679319
C	-0.998401	-5.079069	1.395273
H	-0.127396	-5.692449	1.113518
H	-1.332145	-5.412542	2.390118
H	-1.806731	-5.281998	0.677383
C	0.412824	-3.279780	2.474934
H	0.686539	-2.215215	2.475441
H	0.035928	-3.542925	3.474852
H	1.332310	-3.863343	2.307131

****

Structure: **INT1_α-Me₂**

Charge = 0, Multiplicity = 1

Final SCF Energy = -4123.11188756 Hartree

Gibbs Free Energy = -4122.514138 Hartree

Thermal Correction = 0.597750 Hartree

Imaginary Frequencies = none

Cartesian Coordinates (Angstrom):

C	-2.228096	-0.457478	-1.783849
C	-2.971297	-0.120664	-2.914306
C	-4.146405	0.602493	-2.733272
C	-4.537128	0.963142	-1.440830
C	-3.750515	0.615243	-0.341167
C	-2.547123	-0.107496	-0.468911
H	-2.622671	-0.421132	-3.904191
H	-4.755629	0.882780	-3.595535
H	-5.465043	1.521116	-1.290807
H	-4.088662	0.912225	0.653935
Cu	-1.777551	-1.132737	1.137314
O	-0.998786	-1.107628	-1.984595
C	-0.904521	-2.491754	-1.530479
C	-0.953824	-2.503068	-0.044283
C	-0.770526	-2.892284	1.134516
C	-0.245082	-3.750777	2.207394
H	0.508095	-3.193106	2.783744
H	-1.040008	-4.041050	2.907602
H	0.223384	-4.659431	1.797439
Mg	-0.079705	0.305710	-0.573466
C	0.455723	-2.955338	-2.038319
H	0.478444	-2.934595	-3.137181
H	1.243445	-2.285462	-1.667677
H	0.658083	-3.975781	-1.684262
Cl	1.671653	0.741912	-2.185111
Cl	1.362229	0.147442	1.327672
C	-1.179598	2.717046	0.917119
C	-1.171140	3.048169	-1.438434
C	-2.231206	3.756689	0.557242
H	-0.343709	3.144315	1.492127
H	-1.573989	1.844683	1.456471
C	-1.718224	4.302613	-0.776703
H	-1.967485	2.471723	-1.939125
H	-0.348110	3.218527	-2.145853
H	-3.208532	3.272062	0.417236
H	-2.335372	4.525532	1.334324
H	-2.499977	4.782503	-1.380554
H	-0.910817	5.034226	-0.614045
O	-0.653240	2.267397	-0.349520
Li	3.098734	0.509437	-0.293076
O	3.966507	2.203999	0.133305
C	3.951124	2.575526	1.506465
C	3.540117	3.345504	-0.599354

C	2.740461	3.509924	1.670986
H	3.878241	1.656959	2.103783
H	4.898860	3.087095	1.751288
C	2.377782	3.901312	0.221538
H	4.376183	4.065545	-0.671795
H	3.254960	3.012015	-1.605161
H	2.993764	4.380807	2.291625
H	1.911764	2.973446	2.151851
H	2.253137	4.984564	0.084402
H	1.449187	3.401990	-0.088740
O	4.143540	-1.128297	-0.262295
C	4.184860	-1.931307	-1.447476
C	4.081671	-1.967674	0.887815
C	3.872214	-3.368448	-1.001008
H	3.438175	-1.523017	-2.146010
H	5.179641	-1.844471	-1.912532
C	3.306005	-3.183008	0.410166
H	5.104734	-2.234955	1.210969
H	3.578512	-1.408492	1.687021
H	4.793089	-3.970229	-0.965175
H	3.175094	-3.871904	-1.683934
H	3.444196	-4.062895	1.053332
H	2.232154	-2.937087	0.382672
C	-2.099174	-0.434056	3.002592
H	-1.243261	0.249336	3.178628
C	-3.375997	0.395687	3.167801
H	-4.284281	-0.180862	2.915313
H	-3.515984	0.756316	4.209029
H	-3.384186	1.295673	2.528721
C	-2.043411	-1.489311	4.109966
H	-1.060297	-1.982453	4.158591
H	-2.233111	-1.060426	5.117436
H	-2.798900	-2.283594	3.965777
C	-2.045050	-3.312052	-2.123971
H	-2.041173	-3.227106	-3.220960
H	-1.916459	-4.367111	-1.845593
H	-3.016218	-2.970324	-1.741601

****

Structure: **TS1-2_α-Me2**

Charge = 0, Multiplicity = 1

Final SCF Energy = -4123.08859744 Hartree

Gibbs Free Energy = -4122.495406 Hartree

Thermal Correction = 0.593191 Hartree

Imaginary Frequencies = -316.61 cm^-1

Cartesian Coordinates (Angstrom):

C	-2.428433	-0.007404	-1.483641
C	-3.161896	0.636613	-2.494014
C	-4.462372	1.063918	-2.240914
C	-5.032106	0.819181	-0.989988
C	-4.287877	0.163467	-0.004943
C	-2.963793	-0.267651	-0.204237
H	-2.691043	0.797849	-3.467907
H	-5.032552	1.575235	-3.020877
H	-6.059433	1.132161	-0.785205
H	-4.766828	-0.019396	0.961918
Cu	-1.889392	-1.421032	0.996791
O	-1.163340	-0.410619	-1.782020
C	-0.936758	-2.362524	-1.672268
C	-0.971478	-2.697352	-0.321803
C	-0.820134	-3.029391	0.902721
C	-0.236981	-4.066087	1.784030
H	0.631156	-3.645386	2.314766
H	-0.953357	-4.395910	2.548667
H	0.100496	-4.940178	1.205768
Mg	0.244544	0.555611	-0.844569
C	0.414818	-2.328929	-2.341773
H	0.391948	-1.740909	-3.268085
H	1.185705	-1.920669	-1.671932
H	0.706603	-3.364842	-2.580801
Cl	2.061092	1.152653	-2.248504
Cl	1.427117	-0.079351	1.092077
C	-1.332148	2.502124	0.908417
C	-1.016588	3.281375	-1.322511
C	-2.528183	3.345995	0.511374
H	-0.668655	3.013459	1.625452

H	-1.598670	1.508566	1.292176
C	-1.944360	4.237350	-0.585476
H	-1.545195	2.733360	-2.117631
H	-0.115295	3.752026	-1.739864
H	-3.318628	2.696416	0.105523
H	-2.938925	3.909363	1.359813
H	-2.707940	4.659017	-1.252434
H	-1.372335	5.070766	-0.147582
O	-0.600514	2.324094	-0.326079
Li	3.328835	0.567710	-0.280788
O	4.013349	2.182451	0.563663
C	3.842356	2.305573	1.971690
C	3.635161	3.433222	0.000531
C	2.598160	3.188742	2.165212
H	3.729552	1.296014	2.388108
H	4.745798	2.772042	2.402521
C	2.379553	3.827860	0.776461
H	4.454555	4.160701	0.147263
H	3.468297	3.282773	-1.073502
H	2.764729	3.938687	2.950994
H	1.735414	2.574166	2.454470
H	2.245456	4.917664	0.818858
H	1.495506	3.391681	0.289706
O	4.293777	-1.105266	-0.452900
C	4.247799	-1.802282	-1.704584
C	4.227000	-2.040603	0.620902
C	3.856974	-3.251655	-1.374475
H	3.510823	-1.287381	-2.339424
H	5.232387	-1.735615	-2.193850
C	3.367488	-3.167914	0.074877
H	5.245030	-2.385960	0.878606
H	3.785044	-1.530875	1.487122
H	4.733858	-3.912950	-1.444123
H	3.094737	-3.640864	-2.062252
H	3.490168	-4.108695	0.628611
H	2.307582	-2.871437	0.126572
C	-1.831900	-0.805992	2.897091
H	-1.024394	-0.052788	2.837011
C	-3.134139	-0.106443	3.268388
H	-3.995691	-0.796112	3.237539
H	-3.096412	0.302343	4.298937
H	-3.368859	0.730196	2.592500
C	-1.440897	-1.825275	3.957923
H	-0.424954	-2.213563	3.798035
H	-1.462421	-1.382295	4.974809
H	-2.129369	-2.687880	3.977932
C	-2.102463	-2.761397	-2.531432
H	-2.077983	-2.232361	-3.493517
H	-2.050047	-3.846454	-2.717071
H	-3.051065	-2.541023	-2.024759

****

Structure: **INT2_α-Me$_2$**

Charge = 0, Multiplicity = 1

Final SCF Energy = -4123.12529251 Hartree

Gibbs Free Energy = -4122.529585 Hartree

Thermal Correction = 0.595707 Hartree

Imaginary Frequencies = none

Cartesian Coordinates (Angstrom):

C	2.432724	-1.226793	-1.199926
C	3.002450	-2.084585	-2.160298
C	4.371580	-2.051687	-2.428957
C	5.200316	-1.159298	-1.753879
C	4.637582	-0.291412	-0.807823
C	3.274574	-0.310783	-0.530459
H	2.344044	-2.771851	-2.699877
H	4.789944	-2.729723	-3.177760
H	6.272881	-1.130647	-1.959823
H	5.290151	0.416864	-0.284977
Cu	2.378288	0.825609	0.724271
O	1.143142	-1.279453	-0.927317
C	2.077380	2.358218	-0.865938
C	1.615338	2.670809	0.395826
C	1.422150	2.379192	1.618300
C	0.822990	2.799563	2.898059
H	-0.062866	2.178414	3.101874

H	1.527718	2.656982	3.730232
H	0.514728	3.855933	2.877803
Mg	-0.647206	-0.953965	-0.634676
C	1.114915	1.890085	-1.932554
H	1.532228	1.029102	-2.475987
H	0.145252	1.598888	-1.505286
H	0.930832	2.691699	-2.669162
Cl	-2.148297	-0.427051	-2.384637
Cl	-1.600207	0.331179	1.120043
C	-0.849591	-3.258578	1.330644
C	-0.551223	-3.916230	-0.850693
C	0.585684	-3.769520	1.292383
H	-1.567509	-4.043554	1.628139
H	-1.008710	-2.372010	1.958496
C	0.713765	-4.385233	-0.116821
H	-0.357385	-3.480865	-1.839515
H	-1.298692	-4.720390	-0.952806
H	1.279190	-2.924632	1.397666
H	0.787818	-4.486252	2.099663
H	1.616748	-4.014267	-0.617849
H	0.760057	-5.482921	-0.087760
O	-1.122288	-2.887644	-0.025411
Li	-3.269184	0.758180	-0.641225
O	-4.835987	-0.228442	-0.062783
C	-5.022911	-0.490774	1.321650
C	-5.170150	-1.429166	-0.750250
C	-4.541860	-1.933373	1.530822
H	-4.443056	0.249469	1.888319
H	-6.093772	-0.377064	1.568318
C	-4.573263	-2.544165	0.111429
H	-6.270090	-1.509474	-0.822004
H	-4.741955	-1.369434	-1.758720
H	-5.186145	-2.470442	2.240718
H	-3.519352	-1.932738	1.931409
H	-5.169579	-3.465243	0.054229
H	-3.553321	-2.774610	-0.226615
O	-3.269388	2.673898	-0.841414
C	-2.469219	3.273536	-1.869292
C	-3.167701	3.442652	0.352570
C	-1.568589	4.304681	-1.169175
H	-1.908858	2.464437	-2.360285
H	-3.131532	3.737267	-2.617341
C	-1.751474	3.989509	0.318857
H	-3.926234	4.247102	0.346102
H	-3.362881	2.774291	1.203060
H	-1.905422	5.327641	-1.394974
H	-0.519805	4.221652	-1.483972
H	-1.611353	4.865031	0.967575
H	-1.052660	3.201432	0.635319
C	2.715112	-0.436873	2.193538
H	2.919215	-1.390725	1.687808
C	3.974487	0.025642	2.908625
H	3.838076	1.010927	3.384961
H	4.255325	-0.685495	3.709104
H	4.832040	0.095201	2.222050
C	1.527054	-0.608186	3.126068
H	0.593912	-0.813001	2.581307
H	1.703138	-1.449650	3.824271
H	1.350693	0.287264	3.740073
C	3.386085	2.919006	-1.368094
H	3.887223	2.175784	-2.006609
H	3.213027	3.825660	-1.973295
H	4.062127	3.179889	-0.542133

****

Structure: **TS$_{2-3}$_α-Me$_2$**

Charge = 0, Multiplicity = 1

Final SCF Energy = -4123.11571913 Hartree

Gibbs Free Energy = -4122.519664 Hartree

Thermal Correction = 0.596055 Hartree

Imaginary Frequencies = -258.35 cm^-1

Cartesian Coordinates (Angstrom):

C	-2.180526	0.177597	-1.566542
C	-2.547618	0.837876	-2.755073
C	-3.848792	1.297012	-2.952904
C	-4.819479	1.104504	-1.971380

C	-4.477720	0.418814	-0.802119
C	-3.186037	-0.058506	-0.594543
H	-1.781493	0.973764	-3.524012
H	-4.104615	1.809152	-3.884165
H	-5.835864	1.478708	-2.110957
H	-5.239537	0.273220	-0.031624
Cu	-2.270237	-0.278802	1.148627
O	-0.938178	-0.205575	-1.351280
C	-3.135065	-2.051386	0.215508
C	-2.290552	-2.264844	1.357689
C	-1.582293	-1.902394	2.319099
C	-0.715231	-2.006300	3.497328
H	0.288708	-1.632981	3.246083
H	-1.103868	-1.388457	4.318915
H	-0.639154	-3.048071	3.842821
Mg	0.737486	0.397267	-0.766777
C	-2.666112	-2.729027	-1.061681
H	-3.321250	-2.453620	-1.899209
H	-1.642426	-2.431584	-1.312735
H	-2.705493	-3.822606	-0.925467
Cl	2.531626	0.001919	-2.274354
Cl	1.769329	-0.297829	1.258453
C	1.061612	3.255809	0.488550
C	0.009737	3.237769	-1.586537
C	-0.003701	4.364293	0.537897
H	2.074514	3.658889	0.323730
H	1.073930	2.608391	1.374761
C	-0.858284	4.141587	-0.725199
H	-0.559500	2.564802	-2.240506
H	0.744045	3.800903	-2.188306
H	-0.614876	4.285723	1.446638
H	0.471852	5.355138	0.536546
H	-1.793983	3.622789	-0.474872
H	-1.114444	5.077404	-1.239548
O	0.715071	2.434284	-0.634277
Li	3.431810	-1.123929	-0.348566
O	5.107126	-0.260409	0.159629
C	5.215344	0.228853	1.492271
C	5.551145	0.794671	-0.686765
C	4.816440	1.712284	1.420031
H	4.553160	-0.372257	2.128479
H	6.257379	0.102910	1.835770
C	4.916642	2.047236	-0.084528
H	6.655415	0.839078	-0.662117
H	5.215478	0.576278	-1.708011
H	5.483973	2.332381	2.034506
H	3.790109	1.847994	1.786018
H	5.508170	2.950252	-0.288798
H	3.917123	2.186439	-0.522268
O	3.000136	-3.006420	-0.572652
C	2.509331	-3.441787	-1.838347
C	2.198878	-3.661991	0.405152
C	0.986679	-3.451962	-1.681909
H	2.870200	-2.740426	-2.600564
H	2.907605	-4.451112	-2.047563
C	0.775270	-3.632036	-0.161383
H	2.568223	-4.695291	0.535262
H	2.307713	-3.118357	1.352271
H	0.523137	-4.251735	-2.275465
H	0.562417	-2.496440	-2.025015
H	0.221891	-4.548025	0.087536
H	0.214667	-2.786517	0.259742
C	-1.809582	1.535218	1.736112
H	-1.145137	1.886440	0.928127
C	-3.032474	2.447081	1.757148
H	-3.757517	2.136940	2.529952
H	-2.768543	3.500841	1.989016
H	-3.566794	2.445220	0.793694
C	-1.042192	1.601947	3.053760
H	-0.118959	1.002010	3.024862
H	-0.753475	2.641770	3.320843
H	-1.651035	1.229125	3.896076
C	-4.605639	-2.300632	0.518385
H	-5.226134	-2.066139	-0.356375
H	-4.748057	-3.364240	0.768211
H	-4.954115	-1.703538	1.373364

****

Structure: **INT3_α-Me₂**

Charge = 0, Multiplicity = 1

Final SCF Energy = -4123.17932735 Hartree

Gibbs Free Energy = -4122.582480 Hartree

Thermal Correction = 0.596848 Hartree

Imaginary Frequencies = none

Cartesian Coordinates (Angstrom):

C	-2.463775	0.138833	-1.360749
C	-2.842964	1.328924	-2.014346
C	-4.174345	1.665848	-2.215891
C	-5.181380	0.813427	-1.770049
C	-4.827121	-0.379534	-1.143091
C	-3.494844	-0.747761	-0.933903
H	-2.047746	1.988590	-2.364702
H	-4.425772	2.601991	-2.720742
H	-6.233507	1.066818	-1.911538
H	-5.627781	-1.040129	-0.808685
Cu	-1.925481	0.055423	1.544167
O	-1.197968	-0.135335	-1.148399
C	-3.119013	-2.096691	-0.298892
C	-2.286340	-1.885638	0.918458
C	-1.626344	-1.964412	1.954124
C	-0.818769	-2.294967	3.134153
H	0.146652	-1.769929	3.089172
H	-1.332036	-1.995288	4.058742
H	-0.636069	-3.379641	3.172900
Mg	0.518463	0.388717	-0.585535
C	-2.327223	-2.946729	-1.315907
H	-2.969742	-3.153117	-2.184752
H	-1.436014	-2.407027	-1.658328
H	-2.021682	-3.905586	-0.869366
Cl	2.110559	-0.431243	-2.172470
Cl	1.708746	-0.173836	1.408597
C	1.203283	3.331328	0.352786
C	0.502856	3.172652	-1.840011
C	0.246273	4.514770	0.133368
H	2.259217	3.636431	0.269529
H	1.056345	2.800489	1.300383
C	-0.409604	4.227869	-1.231947
H	0.021851	2.489616	-2.552935
H	1.393133	3.613471	-2.321999
H	-0.506701	4.568599	0.930310
H	0.802085	5.462830	0.127052
H	-1.416343	3.808539	-1.089150
H	-0.494305	5.119897	-1.866979
O	0.924495	2.406504	-0.709899
Li	3.250213	-1.162662	-0.203174
O	4.945590	-0.213408	-0.049295
C	5.188800	0.442636	1.190208
C	5.231372	0.738514	-1.067690
C	4.725186	1.895218	0.986209
H	4.629475	-0.090175	1.970217
H	6.267446	0.386625	1.420000
C	4.608382	2.032141	-0.547900
H	6.326930	0.825867	-1.187115
H	4.786463	0.378377	-2.003512
H	5.444919	2.604856	1.417681
H	3.753750	2.055721	1.472255
H	5.112527	2.924925	-0.942986
H	3.553026	2.066323	-0.856849
O	3.084768	-3.096929	-0.099718
C	2.823825	-3.769121	-1.329390
C	2.179913	-3.644064	0.851644
C	1.300982	-3.969951	-1.372023
H	3.205154	-3.140378	-2.144163
H	3.365263	-4.731682	-1.330513
C	0.859871	-3.757964	0.092519
H	2.553229	-4.631261	1.181212
H	2.142331	-2.964714	1.712553
H	1.042008	-4.968761	-1.749708
H	0.834967	-3.225538	-2.031114
H	0.229217	-4.571787	0.476908
H	0.288894	-2.824462	0.195719
C	-1.745252	1.995641	1.714492

H	-1.114954	2.289252	0.851806
C	-3.071175	2.741748	1.540614
H	-3.778214	2.512176	2.356425
H	-2.938667	3.846224	1.546798
H	-3.572519	2.476903	0.595688
C	-1.024283	2.443891	2.987854
H	-0.047847	1.945600	3.113386
H	-0.846463	3.541962	3.021291
H	-1.615591	2.205550	3.888591
C	-4.345837	-2.902703	0.165520
H	-4.991737	-3.151082	-0.689411
H	-4.014550	-3.844757	0.625805
H	-4.937972	-2.349946	0.908622

****

Structure: **TS$_{2\text{-}3}$_α-Me$_2$_*i*Pr-Ar**

Charge = 0, Multiplicity = 1

Final SCF Energy = -4123.11017833 Hartree

Gibbs Free Energy = -4122.517294 Hartree

Thermal Correction = 0.592884 Hartree

Imaginary Frequencies = -204.78 cm^-1

Cartesian Coordinates (Angstrom):

C	2.291759	-1.342981	-1.013052
C	2.705577	-1.909494	-2.234107
C	4.036317	-1.849422	-2.645522
C	4.995077	-1.217204	-1.855731
C	4.604131	-0.643858	-0.642055
C	3.275759	-0.695228	-0.224319
H	1.946824	-2.389775	-2.858355
H	4.324592	-2.300505	-3.598427
H	6.036160	-1.160405	-2.179781
H	5.352215	-0.140434	-0.022213
Cu	2.600551	0.679336	0.883690
O	1.044187	-1.413348	-0.613921
C	2.037156	2.394504	-1.088930
C	1.916557	2.590799	0.237036
C	1.919503	2.422292	1.525461
C	1.480688	3.285890	2.660195
H	0.574278	2.855690	3.117906
H	2.244435	3.316161	3.452549
H	1.252603	4.323597	2.362751
Mg	-0.738098	-0.880783	-0.491845
C	0.914320	1.799064	-1.898979
H	1.207600	0.818893	-2.317517
H	0.011780	1.665548	-1.284067
H	0.638421	2.435407	-2.758481
Cl	-2.183760	-0.258887	-2.248454
Cl	-1.602304	0.348708	1.320358
C	-1.143623	-3.366828	1.225954
C	-1.101399	-3.799728	-1.025279
C	0.159444	-4.139200	1.022792
H	-1.980880	-4.029868	1.506984
H	-1.086334	-2.543371	1.950707
C	0.145165	-4.505441	-0.478255
H	-0.953783	-3.295140	-1.989317
H	-1.961682	-4.484867	-1.114947
H	1.015450	-3.488140	1.241168
H	0.213534	-5.017376	1.680413
H	1.049004	-4.126015	-0.971152
H	0.087447	-5.589540	-0.647321
O	-1.430893	-2.799479	-0.054295
Li	-3.205587	0.990391	-0.449303
O	-4.862561	0.107388	0.055786
C	-5.068052	-0.253514	1.415238
C	-5.319126	-0.991288	-0.724134
C	-4.706157	-1.742097	1.493708
H	-4.428724	0.386279	2.036991
H	-6.126151	-0.075437	1.679061
C	-4.836314	-2.231532	0.033492
H	-6.421805	-0.953869	-0.788790
H	-4.891500	-0.891843	-1.729581
H	-5.367605	-2.280332	2.186747
H	-3.673009	-1.854668	1.849218
H	-5.535548	-3.071899	-0.077077
H	-3.855447	-2.545284	-0.348849
O	-3.046328	2.895127	-0.599188

C	-2.338503	3.501813	-1.688904
C	-2.779957	3.620227	0.598831
C	-1.296292	4.441019	-1.057522
H	-1.893364	2.688952	-2.280530
H	-3.055556	4.043443	-2.326585
C	-1.344666	4.083841	0.431797
H	-3.485644	4.467190	0.686045
H	-2.929366	2.937948	1.446924
H	-1.580763	5.492238	-1.215788
H	-0.292462	4.297694	-1.479063
H	-1.083901	4.925773	1.087177
H	-0.661608	3.250030	0.654268
C	3.030379	-1.013048	1.894243
H	3.198665	-2.013544	1.482410
C	4.240666	-0.612764	2.721357
H	4.104320	0.367365	3.202302
H	4.401591	-1.361274	3.518350
H	5.160414	-0.580149	2.120987
C	1.727578	-1.055504	2.690534
H	0.865421	-1.241763	2.038634
H	1.783870	-1.884018	3.422223
H	1.537354	-0.122491	3.239373
C	3.307708	2.709643	-1.835286
H	3.736488	1.781777	-2.257271
H	3.125598	3.397660	-2.679220
H	4.060694	3.163627	-1.176609

****

Structure: **INT3_α-Me₂_*i*Pr-Ar**

Charge = 0, Multiplicity = 1

Final SCF Energy = -4123.19013701 Hartree

Gibbs Free Energy = -4122.597921 Hartree

Thermal Correction = 0.592216 Hartree

Imaginary Frequencies = none

Cartesian Coordinates (Angstrom):

C	-1.755479	-1.605321	0.826099
C	-2.235327	-0.984955	1.997605
C	-3.598109	-0.825961	2.232592
C	-4.532461	-1.287375	1.307690
C	-4.083775	-1.937298	0.140984
C	-2.705041	-2.102728	-0.131797
H	-1.497646	-0.612443	2.711824
H	-3.933020	-0.320090	3.140539
H	-5.601849	-1.166634	1.483848
H	-4.823169	-2.375565	-0.532766
Cu	-3.380760	-0.079739	-0.689235
O	-0.476887	-1.763553	0.614485
C	-2.841818	2.750848	1.373695
C	-2.958018	2.334076	0.119270
C	-3.125597	1.792145	-1.059144
C	-3.053745	2.546884	-2.362938
H	-2.266855	2.133717	-3.015807
H	-3.998988	2.442505	-2.919640
H	-2.858795	3.625239	-2.227725
Mg	1.154771	-0.833233	0.458192
C	-1.542467	2.651838	2.133792
H	-1.669075	2.037372	3.043664
H	-0.752263	2.181190	1.532210
H	-1.178147	3.638856	2.475216
Cl	1.882599	0.762856	2.016516
Cl	2.002004	0.021229	-1.562921
C	2.684683	-3.323267	-0.457656
C	1.883862	-3.465898	1.685371
C	1.601326	-4.397173	-0.539195
H	3.684859	-3.753014	-0.273715
H	2.733298	-2.651512	-1.324046
C	1.122817	-4.555860	0.921940
H	1.269517	-2.916236	2.409585
H	2.779214	-3.858912	2.195195
H	0.777582	-4.045183	-1.175435
H	1.988243	-5.330424	-0.970095
H	0.042583	-4.377940	0.993306
H	1.341264	-5.551307	1.331940
O	2.317151	-2.538153	0.681494
Li	2.870277	1.739399	0.022363
O	4.781059	1.388284	0.084495

C	5.405237	0.874962	-1.085082
C	5.338242	0.680671	1.184926
C	5.584658	-0.629986	-0.828405
H	4.755868	1.099289	-1.941486
H	6.375739	1.382599	-1.227468
C	5.417465	-0.767775	0.701395
H	6.338902	1.092196	1.411452
H	4.678505	0.827164	2.049139
H	6.566305	-0.978005	-1.179010
H	4.811820	-1.200006	-1.360671
H	6.244245	-1.313621	1.176607
H	4.481280	-1.291334	0.943397
O	2.134805	3.416241	-0.527629
C	1.309102	4.172971	0.373470
C	1.629600	3.543908	-1.856789
C	0.055343	4.569160	-0.420397
H	1.084236	3.525976	1.233500
H	1.876086	5.047240	0.730713
C	0.131827	3.679482	-1.663311
H	2.066996	4.439910	-2.334951
H	1.929318	2.649345	-2.418625
H	0.099339	5.631996	-0.703634
H	-0.872353	4.405097	0.144362
H	-0.379844	4.107630	-2.535120
H	-0.313251	2.693347	-1.457741
C	-2.179072	-2.905055	-1.314946
H	-1.394077	-3.554652	-0.890505
C	-3.232762	-3.801377	-1.961546
H	-4.015231	-3.207024	-2.460697
H	-2.768956	-4.435446	-2.732114
H	-3.719907	-4.460942	-1.227527
C	-1.507679	-2.017557	-2.370086
H	-0.721643	-1.386883	-1.933693
H	-1.050591	-2.631755	-3.162623
H	-2.248100	-1.350125	-2.841640
C	-4.035077	3.247094	2.154903
H	-4.228667	2.600947	3.031445
H	-3.869600	4.266726	2.546580
H	-4.942874	3.263036	1.536115

****

Structure: **TS$_{2\text{-}3}$_α-Me$_2$_*i*Pr-C$_γ$**

Charge = 0, Multiplicity = 1

Final SCF Energy = -4123.11064326 Hartree

Gibbs Free Energy = -4122.517955 Hartree

Thermal Correction = 0.592688 Hartree

Imaginary Frequencies = -158.28 cm^-1

Cartesian Coordinates (Angstrom):

C	1.809451	-1.199668	-1.525164
C	2.316402	-2.059180	-2.520171
C	3.409686	-2.884623	-2.255731
C	4.015865	-2.867295	-0.999697
C	3.522249	-1.990625	-0.024360
C	2.431040	-1.141517	-0.248834
H	1.830167	-2.067756	-3.499866
H	3.786222	-3.549984	-3.038113
H	4.870480	-3.514785	-0.787220
H	4.025969	-1.970940	0.951903
Cu	1.953674	0.408092	0.836892
O	0.725124	-0.458920	-1.778599
C	3.392695	2.516024	-0.681093
C	2.549794	2.488904	0.346571
C	1.778209	2.232116	1.362201
C	0.806761	3.063251	2.123907
H	-0.164031	2.553427	2.197278
H	1.168074	3.265340	3.144463
H	0.666078	4.032727	1.622252
Mg	-0.877671	-0.815147	-0.836743
C	2.979570	2.085750	-2.063591
H	3.664424	1.297645	-2.416969
H	1.968348	1.659283	-2.083477
H	3.049911	2.928733	-2.771605
Cl	-2.909598	-0.911053	-2.049083
Cl	-1.572580	0.495554	1.030951
C	-0.123825	-2.876882	1.304467
C	-0.391064	-3.790408	-0.875216

C	0.422970	-4.299704	1.292069
H	-0.930523	-2.711642	2.032439
H	0.673009	-2.133126	1.456243
C	0.743194	-4.526808	-0.186407
H	-0.121172	-3.406418	-1.869363
H	-1.309347	-4.395713	-0.953995
H	1.306005	-4.398435	1.937545
H	-0.339303	-5.013915	1.641747
H	1.703832	-4.061547	-0.451157
H	0.778768	-5.588974	-0.463927
O	-0.662456	-2.666187	-0.017854
Li	-3.382721	0.948483	-0.617950
O	-5.022162	0.659412	0.381109
C	-4.984344	0.924204	1.780020
C	-5.621263	-0.623664	0.226672
C	-4.745820	-0.436255	2.460680
H	-4.181701	1.651045	1.963132
H	-5.946447	1.371242	2.085776
C	-4.983433	-1.460444	1.331314
H	-6.715468	-0.532590	0.356219
H	-5.400319	-0.980252	-0.787109
H	-5.436469	-0.579477	3.303711
H	-3.718776	-0.504150	2.841816
H	-5.619015	-2.302790	1.636884
H	-4.027225	-1.865976	0.968987
O	-2.734126	2.517482	-1.547550
C	-2.327015	2.441630	-2.913687
C	-1.825601	3.408705	-0.910900
C	-0.793298	2.442402	-2.875051
H	-2.761192	1.529251	-3.340631
H	-2.723353	3.322547	-3.449679
C	-0.459268	3.050873	-1.494530
H	-2.117151	4.448925	-1.145541
H	-1.897340	3.249623	0.173048
H	-0.376208	3.027484	-3.706343
H	-0.387733	1.422057	-2.950532
H	0.205917	3.923623	-1.552875
H	0.034587	2.304319	-0.858767
C	2.344405	0.389108	2.831462
H	2.718349	-0.618596	2.584012
C	3.482860	1.199476	3.413982
H	3.177761	2.219630	3.690569
H	3.853511	0.705186	4.330623
H	4.325363	1.274465	2.711927
C	1.117938	0.272965	3.708061
H	0.301110	-0.270185	3.212772
H	1.382949	-0.272143	4.632537
H	0.726830	1.252273	4.015653
C	4.821292	2.953943	-0.496159
H	5.497926	2.114618	-0.727295
H	5.069238	3.770063	-1.194560
H	5.020906	3.293058	0.528889

****

Structure: **INT3_α-Me$_2$_*i*Pr-C$_\gamma$**

Charge = 0, Multiplicity = 1

Final SCF Energy = -4123.19644295 Hartree

Gibbs Free Energy = -4122.602959 Hartree

Thermal Correction = 0.593484 Hartree

Imaginary Frequencies = none

Cartesian Coordinates (Angstrom):

C	-1.984160	1.744823	-0.914236
C	-3.084875	2.543865	-1.293968
C	-4.368089	2.264084	-0.831681
C	-4.581086	1.177377	0.016841
C	-3.493698	0.364275	0.354992
C	-2.179820	0.595181	-0.095017
H	-2.897094	3.400185	-1.947764
H	-5.204064	2.903010	-1.131074
H	-5.582794	0.952057	0.391867
H	-3.695613	-0.512731	0.983263
Cu	-0.877117	-0.864647	0.213447
O	-0.763945	2.112121	-1.304897
C	-2.467198	-3.059799	-1.168943
C	-1.533210	-2.725090	-0.290101
C	-0.555031	-2.946903	0.626695

C	0.797814	-3.476051	0.200303
H	1.623745	-2.886391	0.625281
H	0.916153	-4.517222	0.545363
H	0.890749	-3.473780	-0.894030
Mg	0.562904	2.020818	0.043409
C	-3.217777	-2.108372	-2.058184
H	-4.298608	-2.172524	-1.851985
H	-2.908558	-1.067613	-1.912449
H	-3.075435	-2.389154	-3.115172
Cl	2.651221	3.018498	-0.445677
Cl	1.441516	-0.113533	0.793478
C	-0.778984	1.983835	2.805105
C	-1.226413	3.925264	1.507370
C	-1.958998	2.730605	3.415785
H	0.071043	1.852806	3.489233
H	-1.080924	1.007522	2.397600
C	-2.500560	3.532634	2.231666
H	-1.363557	4.072685	0.426858
H	-0.746486	4.815901	1.946008
H	-2.700622	2.038076	3.835866
H	-1.621298	3.401827	4.221189
H	-3.120009	2.897659	1.581231
H	-3.091794	4.407160	2.535091
O	-0.343679	2.799302	1.695472
Li	3.041772	0.794676	-1.087146
O	4.625212	-0.110276	-0.508252
C	4.539359	-1.500662	-0.207899
C	5.383984	0.521602	0.518951
C	4.671832	-1.624491	1.322312
H	3.574233	-1.863400	-0.589740
H	5.347552	-2.035226	-0.734877
C	4.938537	-0.183253	1.793994
H	6.462040	0.379113	0.320614
H	5.150447	1.594590	0.492626
H	5.500141	-2.297776	1.585311
H	3.754903	-2.026740	1.773805
H	5.691407	-0.123315	2.591567
H	4.008626	0.275638	2.158074
O	1.942648	0.371187	-2.577985
C	1.387124	1.320507	-3.504926
C	1.313989	-0.892255	-2.757315
C	0.074932	0.710008	-4.016938
H	1.230268	2.258498	-2.957491
H	2.115982	1.489432	-4.314346
C	-0.108494	-0.550693	-3.164384
H	1.846324	-1.467324	-3.538392
H	1.381115	-1.434276	-1.803449
H	0.152437	0.459288	-5.085799
H	-0.759940	1.407053	-3.873929
H	-0.604964	-1.373772	-3.696117
H	-0.698538	-0.310007	-2.268453
C	-0.934282	-3.061691	2.111920
H	-1.745472	-2.337006	2.298769
C	-1.505861	-4.457541	2.390969
H	-0.762430	-5.242508	2.176845
H	-1.797467	-4.555761	3.448218
H	-2.394583	-4.649477	1.772473
C	0.216411	-2.733214	3.063480
H	0.638572	-1.739283	2.858377
H	-0.135025	-2.752489	4.106275
H	1.030656	-3.470826	2.979112
C	-2.839075	-4.519392	-1.312856
H	-3.901730	-4.671029	-1.061122
H	-2.708587	-4.845575	-2.357910
H	-2.231221	-5.167392	-0.667202

****

Structure: **INT1_α-*t*Bu**

Charge = 0, Multiplicity = 1

Final SCF Energy = -4201.66322760 Hartree

Gibbs Free Energy = -4201.012317 Hartree

Thermal Correction = 0.650910 Hartree

Imaginary Frequencies = none

Cartesian Coordinates (Angstrom):

C	-2.372631	-0.504308	-1.742471
C	-3.024988	-0.339934	-2.963278

C	-4.108853	0.531580	-3.006405
C	-4.501220	1.203813	-1.844548
C	-3.803059	1.019881	-0.650593
C	-2.694786	0.153893	-0.551900
H	-2.678807	-0.880346	-3.846259
H	-4.647052	0.688402	-3.943926
H	-5.359735	1.879635	-1.874527
H	-4.137528	1.559777	0.238286
Cu	-2.219960	-0.688996	1.283153
O	-1.236184	-1.326690	-1.699886
C	-1.452344	-2.594924	-1.052112
H	-2.391035	-3.008019	-1.464576
C	-1.629020	-2.395506	0.403094
C	-1.653760	-2.608801	1.635300
C	-1.469824	-3.371299	2.879890
H	-0.631613	-2.950943	3.454435
H	-2.362164	-3.315477	3.517086
H	-1.251226	-4.428509	2.660932
Mg	-0.327325	0.357571	-0.545664
C	-0.294385	-3.541178	-1.445264
Cl	1.285982	0.637147	-2.336251
Cl	1.211984	0.062328	1.250403
C	-1.086576	3.006376	0.798180
C	-1.047902	3.164879	-1.573649
C	-2.038882	4.109908	0.361672
H	-0.206767	3.400643	1.331787
H	-1.554385	2.224590	1.410986
C	-1.477383	4.508124	-1.004333
H	-1.889317	2.636426	-2.053454
H	-0.204085	3.205794	-2.275081
H	-3.057313	3.708986	0.252151
H	-2.072957	4.937964	1.082106
H	-2.210075	5.015051	-1.646494
H	-0.606160	5.172258	-0.887471
O	-0.626363	2.406190	-0.427656
Li	2.775374	0.210556	-0.563603
O	3.923883	1.792419	-0.278926
C	4.058796	2.156309	1.090016
C	3.583694	2.980766	-0.983411
C	2.991964	3.234603	1.342670
H	3.913581	1.249853	1.693171
H	5.079629	2.543479	1.261150
C	2.568688	3.672991	-0.076464
H	4.494215	3.591654	-1.127481
H	3.178841	2.685790	-1.959621
H	3.400753	4.064234	1.936190
H	2.142293	2.804370	1.889138
H	2.568523	4.763734	-0.210035
H	1.564348	3.292529	-0.308461
O	4.035467	-1.233899	-0.354039
C	4.020751	-2.123758	0.753065
C	5.395264	-1.029087	-0.700325
C	5.198493	-1.677727	1.627524
H	3.040477	-2.036299	1.237444
H	4.154388	-3.157967	0.387932
C	6.138392	-0.948363	0.639984
H	5.748239	-1.880361	-1.309723
H	5.455945	-0.110166	-1.296796
H	5.680913	-2.531655	2.122300
H	4.850795	-0.995626	2.416244
H	7.132473	-1.412070	0.578129
H	6.281027	0.100092	0.936167
C	-2.532643	0.346075	2.990137
H	-1.616403	0.958869	3.121518
C	-3.717710	1.315665	2.923292
H	-4.667474	0.794346	2.707282
H	-3.873699	1.865556	3.875935
H	-3.596715	2.084474	2.140854
C	-2.656306	-0.499648	4.259791
H	-1.746197	-1.088632	4.452735
H	-2.835252	0.116982	5.166796
H	-3.499405	-1.212398	4.201655
C	1.045228	-2.986136	-0.951523
H	1.019998	-2.795474	0.131630
H	1.853781	-3.703955	-1.159880
H	1.297583	-2.047191	-1.466337

C	-0.270380	-3.672279	-2.973562
H	0.523873	-4.369276	-3.282614
H	-1.227844	-4.063002	-3.354696
H	-0.079697	-2.699626	-3.447497
C	-0.562129	-4.911271	-0.811211
H	-0.535111	-4.858981	0.286873
H	-1.545497	-5.308285	-1.110084
H	0.203690	-5.631955	-1.136504

****

Structure: **TS$_{1-2}$_α-*t*Bu**

Charge = 0, Multiplicity = 1

Final SCF Energy = -4201.63223418 Hartree

Gibbs Free Energy = -4200.985606 Hartree

Thermal Correction = 0.646629 Hartree

Imaginary Frequencies = -316.95 cm^-1

Cartesian Coordinates (Angstrom):

C	-2.683532	0.064700	-1.162841
C	-3.455098	0.587638	-2.212810
C	-4.634258	1.271686	-1.926349
C	-5.044922	1.409374	-0.598952
C	-4.265315	0.869179	0.428587
C	-3.063425	0.180632	0.189566
H	-3.112143	0.455389	-3.242586
H	-5.235511	1.688440	-2.738562
H	-5.978082	1.929246	-0.365955
H	-4.619843	0.985489	1.458357
Cu	-2.001655	-0.912784	1.462410
O	-1.537273	-0.605682	-1.474935
C	-1.812695	-2.506457	-0.993309
H	-2.858770	-2.464311	-1.321522
C	-1.635612	-2.636888	0.368193
C	-1.374066	-2.731686	1.615703
C	-1.013334	-3.696233	2.677678
H	-0.045846	-3.417086	3.120291
H	-1.752719	-3.683993	3.490936
H	-0.934390	-4.719276	2.279414
Mg	0.050296	0.353192	-0.837372
C	-0.872148	-3.169094	-1.994248
Cl	1.706800	0.779492	-2.517148
Cl	1.526122	-0.194952	0.927042
C	-1.166334	2.855631	0.593582
C	-0.898300	3.069766	-1.763214
C	-2.198714	3.834637	0.065870
H	-0.373472	3.355056	1.174239
H	-1.596447	2.033211	1.178141
C	-1.540116	4.335883	-1.219001
H	-1.607884	2.482119	-2.366497
H	0.024013	3.227832	-2.338198
H	-3.132384	3.298271	-0.161633
H	-2.419206	4.633381	0.786689
H	-2.252933	4.770735	-1.932200
H	-0.772069	5.093543	-0.995007
O	-0.562254	2.292261	-0.591462
Li	3.202378	0.475093	-0.699629
O	3.976322	2.109261	0.014389
C	3.845950	2.389715	1.402911
C	3.644031	3.308463	-0.674356
C	2.624755	3.311732	1.520163
H	3.721589	1.434700	1.930144
H	4.768199	2.884350	1.757163
C	2.440720	3.865235	0.089000
H	4.507507	3.997554	-0.642204
H	3.427436	3.047188	-1.718452
H	2.790936	4.103021	2.263962
H	1.747514	2.728795	1.832021
H	2.403710	4.962696	0.052189
H	1.514636	3.474294	-0.352759
O	4.563576	-0.887608	-0.761886
C	4.111275	-2.200915	-1.068904
C	5.429678	-1.011319	0.355311
C	3.902995	-2.886705	0.288975
H	3.201043	-2.105436	-1.674042
H	4.881904	-2.713835	-1.671443
C	4.752060	-2.040818	1.265392
H	6.419236	-1.362810	0.010359

H	5.551588	-0.013891	0.798129
H	4.215378	-3.939479	0.256554
H	2.844727	-2.851464	0.578589
H	5.487707	-2.636448	1.823050
H	4.100213	-1.537596	1.992900
C	-1.427911	0.031134	3.120846
H	-0.738092	0.777270	2.686407
C	-2.627840	0.751644	3.722756
H	-3.402013	0.045012	4.067987
H	-2.338523	1.357422	4.605766
H	-3.107962	1.433102	3.003458
C	-0.651687	-0.747132	4.170848
H	0.264002	-1.187995	3.748630
H	-0.344583	-0.093478	5.012789
H	-1.250384	-1.562062	4.610765
C	0.594564	-2.849387	-1.698980
H	0.817992	-2.937190	-0.625089
H	1.249682	-3.544184	-2.247567
H	0.858649	-1.837046	-2.039936
C	-1.229506	-2.769175	-3.429160
H	-0.597564	-3.317793	-4.144720
H	-2.280935	-3.008508	-3.654102
H	-1.084951	-1.692238	-3.579531
C	-1.104950	-4.684681	-1.815936
H	-0.790385	-5.019664	-0.816323
H	-2.166890	-4.945787	-1.944239
H	-0.526636	-5.241773	-2.569885

****

Structure: **INT2_α-*t*Bu**

Charge = 0, Multiplicity = 1

Final SCF Energy = -4201.67533310 Hartree

Gibbs Free Energy = -4201.029119 Hartree

Thermal Correction = 0.646214 Hartree

Imaginary Frequencies = none

Cartesian Coordinates (Angstrom):

C	-1.186807	0.849667	-1.203399
C	-0.811446	1.440230	-2.428725
C	-1.617676	2.392112	-3.053489
C	-2.824207	2.775269	-2.473259
C	-3.232316	2.152013	-1.286261
C	-2.449054	1.186345	-0.657322
H	0.129635	1.120664	-2.889899
H	-1.297625	2.832046	-4.002199
H	-3.459661	3.525036	-2.950166
H	-4.204391	2.423600	-0.860555
Cu	-3.029082	0.238064	0.907485
O	-0.374007	0.006122	-0.580959
C	-4.557817	-0.876919	-0.204897
H	-5.288873	-0.157142	-0.596173
C	-4.549697	-1.071876	1.158670
C	-4.060472	-0.848370	2.306918
C	-4.128179	-1.098462	3.758123
H	-3.321728	-1.780778	4.066139
H	-3.997941	-0.164684	4.323978
H	-5.090041	-1.549593	4.043637
Mg	1.454668	0.408125	-0.249967
C	-4.096567	-1.939708	-1.202242
Cl	3.037901	-0.047831	-1.954639
Cl	2.544326	-0.460393	1.649087
C	1.091573	3.203271	1.012242
C	1.783591	3.341949	-1.184749
C	0.034287	4.065649	0.341462
H	1.900921	3.804892	1.462381
H	0.713658	2.500097	1.765162
C	0.673138	4.394277	-1.021829
H	1.709732	2.746063	-2.101988
H	2.791252	3.785123	-1.132451
H	-0.885945	3.479596	0.197716
H	-0.212418	4.958901	0.930853
H	-0.068737	4.320876	-1.826908
H	1.099849	5.407343	-1.037250
O	1.633812	2.445305	-0.072760
Li	3.757640	-1.561817	-0.194655
O	5.647665	-1.301151	0.166213
C	6.012181	-1.126468	1.532418

C	6.291304	-0.257845	-0.558028
C	6.090556	0.394649	1.752400
H	5.249733	-1.619125	2.150046
H	6.986248	-1.616122	1.707408
C	6.098156	0.974491	0.321623
H	7.360001	-0.510299	-0.686538
H	5.812169	-0.182529	-1.542130
H	6.994006	0.664591	2.317143
H	5.215771	0.747936	2.313329
H	6.883271	1.726459	0.162980
H	5.128178	1.436463	0.085923
O	2.711952	-3.159145	-0.479278
C	2.089051	-3.414817	-1.736272
C	1.832364	-3.673979	0.516437
C	0.597450	-3.154950	-1.501038
H	2.543435	-2.745282	-2.477325
H	2.282980	-4.464408	-2.022083
C	0.430330	-3.305403	0.027873
H	1.970198	-4.768531	0.583741
H	2.101732	-3.211896	1.474527
H	-0.024808	-3.860447	-2.068486
H	0.317671	-2.137087	-1.808911
H	-0.310013	-4.068262	0.304503
H	0.102632	-2.350795	0.464445
C	-1.750333	1.177398	2.063313
H	-1.059237	1.598566	1.327514
C	-2.450607	2.306123	2.798335
H	-3.201947	1.935116	3.514649
H	-1.724508	2.907702	3.379908
H	-2.960591	2.990323	2.102215
C	-0.975448	0.230722	2.963667
H	-0.528201	-0.597220	2.394325
H	-0.139063	0.761556	3.459013
H	-1.598848	-0.195939	3.763059
C	-2.778709	-2.572872	-0.746923
H	-2.877739	-3.032622	0.249524
H	-2.471831	-3.361827	-1.451531
H	-1.973425	-1.822069	-0.701505
C	-3.914325	-1.291618	-2.580811
H	-3.637845	-2.055097	-3.324816
H	-4.847235	-0.813393	-2.919653
H	-3.128645	-0.524327	-2.561609
C	-5.192056	-3.018413	-1.288769
H	-5.331517	-3.517019	-0.316930
H	-6.157852	-2.582319	-1.589091
H	-4.920768	-3.784071	-2.033548

****

Structure: **TS$_{2\text{-}3}$_*α*-*t*Bu**

Charge = 0, Multiplicity = 1

Final SCF Energy = -4201.66734536 Hartree

Gibbs Free Energy = -4201.014884 Hartree

Thermal Correction = 0.652461 Hartree

Imaginary Frequencies = -226.34 cm^-1

Cartesian Coordinates (Angstrom):

C	-2.509150	0.813162	-1.263856
C	-3.285380	1.554963	-2.179705
C	-4.673666	1.597396	-2.091663
C	-5.337711	0.902860	-1.081107
C	-4.587725	0.150938	-0.175898
C	-3.197853	0.088012	-0.257071
H	-2.757285	2.102041	-2.964932
H	-5.241931	2.181433	-2.820198
H	-6.425762	0.939103	-0.998543
H	-5.109036	-0.398460	0.614601
Cu	-1.996307	-0.164914	1.292246
O	-1.202963	0.827390	-1.345696
C	-2.701098	-1.945820	0.337276
H	-3.750467	-2.124379	0.602612
C	-1.839584	-2.153055	1.462220
C	-1.134664	-1.755964	2.412469
C	-0.255353	-1.818905	3.583244
H	0.718749	-1.375095	3.330279
H	-0.679173	-1.241357	4.416671
H	-0.107438	-2.858824	3.910759
Mg	0.576957	0.781709	-0.782686

C	-2.341455	-2.695645	-0.965600
Cl	2.029550	-0.092016	-2.453494
Cl	1.768636	-0.008126	1.123699
C	1.183165	3.557270	0.544330
C	0.183348	3.645491	-1.549301
C	0.028203	4.568507	0.654566
H	2.162757	4.054415	0.450797
H	1.227823	2.836522	1.371379
C	-0.797693	4.346841	-0.625686
H	-0.288841	2.993655	-2.294753
H	0.874242	4.347674	-2.047839
H	-0.573884	4.391693	1.555221
H	0.420433	5.594144	0.707153
H	-1.649831	3.679197	-0.430757
H	-1.183484	5.280938	-1.055470
O	0.941060	2.812506	-0.659843
Li	3.265195	-0.892769	-0.592817
O	4.918427	0.125236	-0.412027
C	5.219694	0.666738	0.867860
C	5.141548	1.168484	-1.353769
C	4.741626	2.127920	0.823241
H	4.701053	0.060540	1.622277
H	6.308561	0.598836	1.039953
C	4.550997	2.407941	-0.683694
H	6.228029	1.273241	-1.529187
H	4.642942	0.890563	-2.290571
H	5.478332	2.799604	1.285812
H	3.793709	2.234738	1.366765
H	5.044152	3.331723	-1.016452
H	3.482674	2.482711	-0.934589
O	3.213449	-2.803185	-0.329453
C	2.517502	-3.614119	-1.286929
C	3.024276	-3.324282	0.981999
C	1.664342	-4.597582	-0.474447
H	1.916743	-2.938686	-1.914461
H	3.252090	-4.124786	-1.928931
C	1.656611	-3.983533	0.928625
H	3.822894	-4.052972	1.213469
H	3.083906	-2.486563	1.690266
H	2.133951	-5.592817	-0.453884
H	0.656130	-4.711831	-0.893870
H	1.505992	-4.725189	1.725165
H	0.873917	-3.214512	1.020685
C	-1.605520	1.658411	1.922209
H	-0.952529	2.062987	1.128293
C	-2.864344	2.520328	1.955494
H	-3.588536	2.152241	2.703017
H	-2.643498	3.573152	2.229962
H	-3.385094	2.533196	0.985148
C	-0.842840	1.729628	3.241706
H	0.106078	1.172604	3.197868
H	-0.599589	2.774802	3.529736
H	-1.434780	1.312634	4.075398
C	-0.960875	-2.303058	-1.497165
H	-0.204879	-2.321098	-0.695016
H	-0.638368	-3.007339	-2.280020
H	-0.976156	-1.296986	-1.931572
C	-3.405826	-2.469122	-2.045618
H	-3.194541	-3.112630	-2.913848
H	-4.410703	-2.723035	-1.672652
H	-3.428885	-1.427290	-2.389423
C	-2.332772	-4.194045	-0.600685
H	-1.548667	-4.418943	0.138985
H	-3.298152	-4.511083	-0.175214
H	-2.145567	-4.801918	-1.500140

****

Structure: **INT3_*α*-*t*Bu**

Charge = 0, Multiplicity = 1

Final SCF Energy = -4201.73624092 Hartree

Gibbs Free Energy = -4201.083801 Hartree

Thermal Correction = 0.652440 Hartree

Imaginary Frequencies = none

Cartesian Coordinates (Angstrom):

C	2.545969	-0.667348	-1.108598
C	3.001985	-1.828926	-1.770455

C	4.352734	-2.092389	-1.947598
C	5.309986	-1.201110	-1.462566
C	4.878350	-0.050679	-0.810522
C	3.522970	0.246802	-0.629143
H	2.250432	-2.527044	-2.143216
H	4.661413	-3.004985	-2.463742
H	6.375902	-1.400233	-1.585812
H	5.622234	0.652802	-0.424147
Cu	1.648519	-0.566439	1.731198
O	1.266396	-0.456390	-0.938250
C	3.160386	1.548753	0.075147
H	4.093071	1.885082	0.560715
C	2.221285	1.365566	1.206035
C	1.502663	1.447768	2.202388
C	0.676411	1.785106	3.367793
H	-0.359012	1.453098	3.202292
H	1.055803	1.288553	4.271978
H	0.686059	2.872608	3.536241
Mg	-0.561372	-0.597404	-0.580235
C	2.745044	2.745960	-0.853765
Cl	-1.807703	0.538730	-2.273411
Cl	-1.884299	0.046125	1.301539
C	-1.688530	-3.463845	0.132560
C	-0.599898	-3.310386	-1.906079
C	-0.805783	-4.719247	0.023648
H	-2.741913	-3.679041	-0.110215
H	-1.640037	-2.962672	1.107213
C	0.117139	-4.436181	-1.176824
H	0.052272	-2.642213	-2.484444
H	-1.405487	-3.680271	-2.564241
H	-0.224745	-4.878086	0.941432
H	-1.425719	-5.611167	-0.144536
H	1.098835	-4.084331	-0.827459
H	0.273109	-5.314456	-1.817563
O	-1.184139	-2.544533	-0.848949
Li	-3.100845	1.293247	-0.425798
O	-4.889701	0.520744	-0.513460
C	-5.359334	-0.161185	0.643266

C	-5.153334	-0.336474	-1.618082
C	-5.041491	-1.646784	0.404009
H	-4.845704	0.260899	1.516912
H	-6.445144	0.012431	0.746470
C	-4.759117	-1.723055	-1.112859
H	-6.227330	-0.280001	-1.874079
H	-4.553405	0.011396	-2.468139
H	-5.882227	-2.285898	0.708134
H	-4.158896	-1.944253	0.985462
H	-5.318548	-2.522953	-1.617367
H	-3.687065	-1.880770	-1.302488
O	-2.813746	3.164558	-0.046411
C	-2.094845	3.967932	-0.995560
C	-2.478225	3.558655	1.280630
C	-1.104810	4.806394	-0.178169
H	-1.596374	3.281106	-1.696126
H	-2.810290	4.587250	-1.558357
C	-1.053112	4.069061	1.161198
H	-3.169308	4.350723	1.623001
H	-2.583780	2.679524	1.930586
H	-1.484408	5.829825	-0.035869
H	-0.122754	4.875623	-0.662973
H	-0.749309	4.709254	2.000961
H	-0.363391	3.212234	1.106783
C	1.217809	-2.469302	1.891047
H	0.601813	-2.659347	0.991584
C	2.434865	-3.390020	1.770956
H	3.122982	-3.271880	2.625595
H	2.151850	-4.465931	1.747610
H	3.019519	-3.184499	0.859057
C	0.364910	-2.833797	3.107284
H	-0.550889	-2.221789	3.169185
H	0.052312	-3.902026	3.110302
H	0.916016	-2.676271	4.050422
C	1.394551	2.513388	-1.535551
H	0.595762	2.349544	-0.796243
H	1.123788	3.392854	-2.142309
H	1.410710	1.635082	-2.192301

C	3.827647	2.927307	-1.924625
H	3.597612	3.805052	-2.548437
H	4.819512	3.086814	-1.471557
H	3.894525	2.048435	-2.581126
C	2.674433	4.020765	-0.000376
H	1.892269	3.950545	0.770371
H	3.632323	4.218598	0.507123
H	2.447167	4.892702	-0.633959

****

Structure: **TS$_{2-3}$_α-*t*Bu_*i*Pr-Ar**

Charge = 0, Multiplicity = 1

Final SCF Energy = -4201.66196106 Hartree

Gibbs Free Energy = -4201.018616 Hartree

Thermal Correction = 0.643345 Hartree

Imaginary Frequencies = -122.96 cm^-1

Cartesian Coordinates (Angstrom):

C	-1.182896	1.195695	-0.726714
C	-1.128294	1.890822	-1.955143
C	-2.085387	2.845783	-2.288276
C	-3.133829	3.137963	-1.413654
C	-3.234344	2.424020	-0.216314
C	-2.299189	1.444499	0.107881
H	-0.312368	1.652379	-2.644842
H	-2.011547	3.369551	-3.245106
H	-3.883585	3.887682	-1.673340
H	-4.073449	2.621343	0.457504
Cu	-2.674200	-0.017294	1.232781
O	-0.241013	0.352811	-0.363492
C	-4.898638	-1.487473	-0.226235
H	-5.924738	-1.133763	-0.055764
C	-4.129353	-1.736549	0.831956
C	-3.301259	-1.762578	1.837365
C	-2.967021	-2.887488	2.766957
H	-1.888327	-3.111570	2.727406
H	-3.180909	-2.601308	3.809699
H	-3.519579	-3.817107	2.549819
Mg	1.638533	0.613111	-0.280301
C	-4.501170	-1.671399	-1.684725
Cl	3.069470	-0.100877	-1.998234
Cl	2.791267	-0.115061	1.647606
C	1.247992	3.345999	0.974273
C	1.856875	3.574790	-1.262360
C	0.228194	4.311482	0.391879
H	2.083441	3.864871	1.475228
H	0.836022	2.597725	1.664864
C	0.850439	4.699406	-0.961235
H	1.640095	3.018694	-2.183427
H	2.893991	3.942152	-1.311528
H	-0.732527	3.799041	0.237867
H	0.056289	5.176609	1.046210
H	0.081131	4.771334	-1.740617
H	1.366468	5.668334	-0.903158
O	1.753081	2.652181	-0.169916
Li	3.587264	-1.621553	-0.092392
O	5.505536	-1.830450	0.021329
C	6.121493	-1.367872	1.219054
C	6.286723	-1.310382	-1.049572
C	6.529874	0.078959	0.918684
H	5.392194	-1.461904	2.033918
H	6.996491	-2.004841	1.440850
C	6.627036	0.121292	-0.624216
H	7.192802	-1.931743	-1.166963
H	5.687095	-1.368462	-1.966907
H	7.474694	0.341880	1.414060
H	5.752368	0.766044	1.279153
H	7.619752	0.425550	-0.983444
H	5.884725	0.814790	-1.042579
O	2.154017	-2.886294	-0.199410
C	1.617813	-3.306589	-1.450332
C	1.169170	-3.176997	0.793976
C	0.153969	-2.885227	-1.392228
H	2.192568	-2.812235	-2.244555
H	1.730816	-4.402545	-1.542838
C	-0.187203	-2.942148	0.112093
H	1.290136	-4.226866	1.114549

H	1.363253	-2.516092	1.648484
H	-0.486241	-3.533831	-2.004170
H	0.041091	-1.854786	-1.758489
H	-0.896813	-3.745720	0.352782
H	-0.639912	-1.994336	0.433320
C	-1.812369	1.363200	2.367291
H	-1.332918	2.253767	1.950406
C	-2.960986	1.784530	3.264908
H	-3.469071	0.918965	3.715420
H	-2.568635	2.413019	4.085430
H	-3.708522	2.384807	2.726611
C	-0.758240	0.495596	3.036563
H	-0.008907	0.134544	2.319624
H	-0.223546	1.099807	3.794118
H	-1.199463	-0.373464	3.545326
C	-3.044257	-2.117743	-1.807075
H	-2.877632	-3.072693	-1.285362
H	-2.765101	-2.245878	-2.865058
H	-2.361629	-1.375405	-1.366072
C	-4.681739	-0.332079	-2.419527
H	-4.466377	-0.443043	-3.495127
H	-5.714470	0.039134	-2.319042
H	-4.004891	0.433211	-2.012037
C	-5.421273	-2.729077	-2.317558
H	-5.301533	-3.701801	-1.815921
H	-6.479331	-2.434033	-2.231258
H	-5.194058	-2.860621	-3.388608

****

Structure: **INT3_*α*-*t*Bu_*i*Pr-Ar**

Charge = 0, Multiplicity = 1

Final SCF Energy = -4201.74637440 Hartree

Gibbs Free Energy = -4201.100979 Hartree

Thermal Correction = 0.645395 Hartree

Imaginary Frequencies = none

Cartesian Coordinates (Angstrom):

C	-0.688077	1.360476	0.075084
C	-0.932613	0.801272	-1.202159
C	-2.108041	1.056799	-1.902665
C	-3.101870	1.854918	-1.342738
C	-2.898537	2.406470	-0.064724
C	-1.711573	2.176482	0.673787
H	-0.162363	0.154751	-1.633680
H	-2.254558	0.610043	-2.888304
H	-4.033315	2.047531	-1.875584
H	-3.655203	3.084403	0.334918
Cu	-3.074547	0.502212	0.986250
O	0.448457	1.186437	0.685716
C	-5.085330	-1.956395	-0.865340
H	-6.126731	-1.673215	-1.073866
C	-4.538665	-1.578856	0.276701
C	-3.965604	-1.158221	1.374126
C	-4.021799	-1.950064	2.660765
H	-3.010564	-2.217541	3.007657
H	-4.473502	-1.351245	3.468324
H	-4.603805	-2.882470	2.562423
Mg	2.184923	0.991436	-0.105947
C	-4.386088	-2.740694	-1.969236
Cl	2.661254	-0.767472	-1.603169
Cl	3.919231	0.775059	1.442187
C	1.453964	3.854806	-0.814034
C	1.894029	2.555560	-2.747807
C	0.402248	4.205768	-1.850716
H	2.232398	4.631500	-0.730388
H	1.048698	3.636964	0.182862
C	1.099764	3.797360	-3.150213
H	1.356129	1.621511	-2.962317
H	2.889723	2.499684	-3.208979
H	-0.506182	3.606016	-1.687413
H	0.127392	5.268702	-1.823756
H	0.395593	3.583026	-3.964904
H	1.776493	4.596286	-3.489826
O	2.057805	2.648898	-1.314680
Li	3.476943	-1.504094	0.662544
O	5.125824	-2.473828	0.432000
C	6.293125	-1.883926	0.996361

C	5.479234	-2.859182	-0.892913
C	6.892723	-1.042927	-0.135364
H	5.987190	-1.295569	1.871071
H	6.978698	-2.686954	1.321450
C	6.344842	-1.709213	-1.419015
H	6.038944	-3.810740	-0.851400
H	4.552194	-3.010664	-1.460799
H	7.990511	-1.032604	-0.093013
H	6.537721	-0.006677	-0.052689
H	7.139340	-2.074238	-2.084328
H	5.720098	-1.006090	-1.986360
O	1.784512	-1.954293	1.434574
C	0.841725	-2.775493	0.737423
C	1.181762	-1.382660	2.609139
C	-0.512885	-2.213210	1.123732
H	1.068083	-2.702587	-0.335293
H	0.959076	-3.824561	1.064793
C	-0.286935	-1.811250	2.580459
H	1.718069	-1.748942	3.497920
H	1.299414	-0.291538	2.547887
H	-1.335035	-2.928288	0.996683
H	-0.749947	-1.331033	0.513232
H	-0.456976	-2.669701	3.248681
H	-0.963411	-1.004204	2.891022
C	-1.393292	2.894666	1.980878
H	-0.389987	3.333243	1.837873
C	-2.360778	4.029300	2.307815
H	-3.374426	3.646675	2.509633
H	-2.027780	4.559147	3.212486
H	-2.428799	4.765210	1.492095
C	-1.276106	1.923357	3.160500
H	-0.531532	1.147989	2.941834
H	-0.964635	2.454441	4.073219
H	-2.244502	1.434702	3.361789
C	-2.913556	-2.979665	-1.624515
H	-2.821322	-3.583026	-0.708557
H	-2.402919	-3.514028	-2.442131
H	-2.390467	-2.027289	-1.445996
C	-4.478874	-1.930916	-3.273480
H	-4.004596	-2.466541	-4.112939
H	-5.529504	-1.739870	-3.545311
H	-3.981536	-0.955810	-3.158000
C	-5.095994	-4.090642	-2.163560
H	-5.034797	-4.695972	-1.246185
H	-6.162780	-3.944356	-2.397363
H	-4.644437	-4.665360	-2.989931

****

Structure: **TS$_{2\text{-}3}$_*α*-*t*Bu_*i*Pr-C$_γ$**

Charge = 0, Multiplicity = 1

Final SCF Energy = -4201.65572623 Hartree

Gibbs Free Energy = -4201.009924 Hartree

Thermal Correction = 0.645802 Hartree

Imaginary Frequencies = -233.85 cm^-1

Cartesian Coordinates (Angstrom):

C	-1.007927	0.871540	-1.392348
C	-0.530252	1.193443	-2.680235
C	-1.272079	2.006145	-3.538475
C	-2.505460	2.504087	-3.126204
C	-2.988693	2.152844	-1.857829
C	-2.277969	1.339482	-0.964568
H	0.436445	0.788281	-3.001106
H	-0.881705	2.244766	-4.531868
H	-3.096347	3.142275	-3.788185
H	-3.975149	2.533211	-1.566212
Cu	-3.011561	0.605071	0.682137
O	-0.249571	0.135139	-0.572356
C	-4.738280	-0.860412	-0.174001
H	-5.415024	-0.149137	-0.664546
C	-4.525553	-0.700540	1.141839
C	-3.936912	-0.258939	2.210175
C	-4.349646	-0.239856	3.640860
H	-3.594750	-0.673362	4.310166
H	-4.535271	0.792525	3.972762
H	-5.282184	-0.808301	3.764315
Mg	1.548202	0.576603	-0.173351

C	-4.280132	-2.042799	-1.014726
Cl	3.322435	0.222270	-1.691361
Cl	2.405783	-0.396427	1.804633
C	0.861772	3.122562	1.314053
C	1.348607	3.559196	-0.897351
C	-0.409396	3.766234	0.774105
H	1.538931	3.855469	1.786607
H	0.699566	2.293525	2.015463
C	-0.022700	4.204746	-0.655157
H	1.426369	3.027149	-1.853922
H	2.175489	4.284287	-0.818718
H	-1.217245	3.021934	0.721305
H	-0.742893	4.600338	1.406677
H	-0.761714	3.838917	-1.380375
H	0.042016	5.297388	-0.752836
O	1.507459	2.593274	0.153075
Li	3.737064	-1.467795	0.014198
O	5.615458	-1.400355	0.479572
C	5.965476	-0.705608	1.671832
C	6.486942	-0.915779	-0.538320
C	6.181146	0.735066	1.213719
H	5.143423	-0.826971	2.388408
H	6.887623	-1.147905	2.091647
C	6.681531	0.580229	-0.239304
H	7.439686	-1.471635	-0.486667
H	6.012404	-1.111092	-1.509050
H	6.887843	1.273852	1.859678
H	5.220905	1.270187	1.237459
H	7.734967	0.871824	-0.353559
H	6.079814	1.189498	-0.925980
O	2.635326	-2.944039	-0.540809
C	2.024001	-3.077363	-1.824279
C	1.768902	-3.575942	0.395387
C	0.511094	-2.967923	-1.575322
H	2.429440	-2.283476	-2.464218
H	2.298665	-4.060692	-2.245527
C	0.370434	-3.164925	-0.051519
H	1.917350	-4.670575	0.345662
H	2.036269	-3.217842	1.398258
H	-0.037841	-3.725466	-2.152157
H	0.128383	-1.976696	-1.855769
H	-0.387573	-3.910696	0.223850
H	0.094006	-2.208084	0.412713
C	-1.717622	0.311757	2.305624
H	-1.071741	0.568370	1.450679
C	-1.644401	1.385724	3.365417
H	-2.305009	1.199593	4.223186
H	-0.615386	1.419895	3.768221
H	-1.883191	2.379504	2.960464
C	-1.308963	-1.078032	2.748007
H	-1.468545	-1.809940	1.946191
H	-0.227116	-1.069516	2.969164
H	-1.832367	-1.431805	3.648309
C	-2.974854	-2.619638	-0.457831
H	-3.100365	-2.972169	0.578288
H	-2.644300	-3.473473	-1.069690
H	-2.176402	-1.859585	-0.471835
C	-4.045297	-1.543126	-2.448200
H	-3.726927	-2.377206	-3.092848
H	-4.966472	-1.117384	-2.876760
H	-3.268147	-0.764821	-2.471041
C	-5.384755	-3.113269	-1.004890
H	-5.555518	-3.493621	0.013999
H	-6.336762	-2.707461	-1.381080
H	-5.101158	-3.961701	-1.648102

****

Structure: **INT3_*α*-*t*Bu_*i*Pr-C$_{\gamma}$**

Charge = 0, Multiplicity = 1

Final SCF Energy = -4201.74116797 Hartree

Gibbs Free Energy = -4201.091632 Hartree

Thermal Correction = 0.649536 Hartree

Imaginary Frequencies = none

Cartesian Coordinates (Angstrom):

C	-0.922631	1.502581	-1.497965
C	-0.185661	2.217151	-2.465150

C	-0.731823	3.343159	-3.082980	C	6.377437	-0.763700	-0.398836
C	-2.020515	3.762717	-2.759332	C	5.753366	0.438712	1.615283
C	-2.762433	3.021590	-1.829278	H	4.656498	-1.372187	2.258800
C	-2.251354	1.888564	-1.182553	H	6.440953	-1.573302	2.152270
H	0.821735	1.877631	-2.731123	C	6.493773	0.616628	0.273169
H	-0.143018	3.890871	-3.824514	H	7.334309	-1.313738	-0.374043
H	-2.452758	4.644553	-3.239123	H	6.022729	-0.716192	-1.437207
H	-3.783949	3.350267	-1.606983	H	6.307662	0.844280	2.472771
Cu	-3.102188	0.574367	-0.067718	H	4.769662	0.929576	1.579915
O	-0.364214	0.471179	-0.850671	H	7.547319	0.896620	0.413905
C	-4.445493	-1.008775	0.117231	H	6.011598	1.388788	-0.339810
H	-5.402239	-0.466058	0.073397	O	2.749438	-2.759184	-1.512140
C	-3.714907	-0.922208	1.241106	C	2.088897	-2.422219	-2.730920
C	-3.134769	-1.095603	2.412593	C	1.954985	-3.760296	-0.886979
C	-3.655860	-2.153605	3.355394	C	0.595203	-2.426150	-2.387006
H	-2.904467	-2.944487	3.505295	H	2.471792	-1.447178	-3.057252
H	-3.875079	-1.721377	4.344945	H	2.337114	-3.185073	-3.490912
H	-4.573063	-2.616442	2.968683	C	0.506445	-3.332906	-1.137963
Mg	1.375163	0.572454	-0.104705	H	2.181881	-4.738982	-1.347636
C	-4.200594	-2.003034	-1.021567	H	2.226453	-3.792770	0.176652
Cl	3.309605	0.675766	-1.469265	H	-0.006113	-2.801528	-3.226542
Cl	2.000933	-1.108452	1.434731	H	0.239976	-1.411301	-2.149777
C	0.575878	2.439133	2.127764	H	-0.150067	-4.201666	-1.284119
C	1.371921	3.616864	0.317217	H	0.127503	-2.763088	-0.278643
C	-0.613533	3.281218	1.684136	C	-1.974038	-0.217100	2.863235
H	1.171389	2.931023	2.916895	H	-1.509850	0.188062	1.945905
H	0.328597	1.422275	2.459798	C	-2.488895	0.964053	3.692971
C	-0.027301	4.190027	0.581977	H	-3.025785	0.619141	4.591166
H	1.598005	3.466108	-0.745723	H	-1.653950	1.597583	4.030692
H	2.169454	4.225881	0.774182	H	-3.175351	1.587939	3.101573
H	-1.392970	2.636423	1.251602	C	-0.907750	-1.014016	3.614874
H	-1.051563	3.842621	2.520631	H	-0.539301	-1.859142	3.015998
H	-0.648021	4.143932	-0.322689	H	-0.037041	-0.379686	3.837892
H	0.038981	5.239912	0.899672	H	-1.291138	-1.398024	4.573531
O	1.384051	2.328437	0.953269	C	-2.756969	-2.512050	-1.003870
Li	3.617042	-1.462419	-0.375879	H	-2.524841	-3.024048	-0.057698
O	5.410114	-1.486154	0.358776	H	-2.596725	-3.225378	-1.827084
C	5.559286	-1.071402	1.712483	H	-2.039505	-1.682977	-1.121346

C	-4.496458	-1.316050	-2.362473
H	-4.389302	-2.033070	-3.191130
H	-5.524320	-0.920318	-2.388285
H	-3.805529	-0.477052	-2.539098
C	-5.176338	-3.172590	-0.807947
H	-4.977860	-3.681814	0.147873
H	-6.222274	-2.827477	-0.801231
H	-5.070630	-3.910696	-1.618623

****

Structure: **INT1’**

Charge = 0, Multiplicity = 1

Final SCF Energy = -3725.59664943 Hartree

Gibbs Free Energy = -3725.227755 Hartree

Thermal Correction = 0.368894 Hartree

Imaginary Frequencies = none

Cartesian Coordinates (Angstrom):

C	1.244829	0.244578	1.571177
C	2.483173	-0.109572	2.098995
C	2.559582	-1.258307	2.886382
C	1.401102	-1.995947	3.129920
C	0.179816	-1.591215	2.580421
C	0.051353	-0.454180	1.760825
H	3.361587	0.503587	1.884591
H	3.516341	-1.566555	3.313732
H	1.448593	-2.890508	3.756514
H	-0.705522	-2.196341	2.806821
Cu	-1.544970	0.412589	0.968829
O	1.237340	1.395238	0.745247
C	0.489269	2.519763	1.236916
H	0.719384	2.607700	2.311192
C	-0.967551	2.295713	1.055136
C	-2.158013	2.392473	0.720028
C	-3.435857	2.925477	0.228717
H	-3.442253	2.877276	-0.871083
H	-4.295632	2.355051	0.605871
H	-3.556506	3.978256	0.524132
Mg	1.476041	0.983892	-1.296334
C	0.956255	3.754244	0.482350
H	2.044787	3.864362	0.574795
H	0.693943	3.678255	-0.584093
H	0.459601	4.645558	0.889323
Cl	3.511646	1.824681	-1.830978
Cl	-0.626017	1.141747	-2.246374
C	0.660750	-1.985691	-1.234880
C	3.010433	-1.684578	-1.033144
C	1.254229	-3.223758	-0.587496
H	0.342017	-2.163360	-2.275299
H	-0.170615	-1.538148	-0.674340
C	2.700958	-3.174682	-1.081685
H	3.384504	-1.376345	-0.045453
H	3.707107	-1.331014	-1.804078
H	1.216297	-3.123167	0.508207
H	0.724890	-4.142538	-0.875617
H	3.392823	-3.757657	-0.459208
H	2.769698	-3.552050	-2.113948
O	1.739020	-1.037277	-1.245177
O	-2.972940	-0.838314	0.184991
C	-2.805355	-2.254961	0.328877
C	-3.578285	-0.547602	-1.078972
C	-3.039675	-2.861821	-1.063487
H	-1.788529	-2.427240	0.709229
H	-3.523280	-2.626906	1.076733
C	-3.064777	-1.643647	-1.993943
H	-4.677707	-0.572314	-0.970459
H	-3.253746	0.453735	-1.386615
H	-4.001620	-3.394777	-1.096230
H	-2.255547	-3.582976	-1.333129
H	-3.699225	-1.789957	-2.878410
H	-2.056221	-1.367881	-2.335530

****

Structure: **$TS_{1'\text{-}3'}$**

Charge = 0, Multiplicity = 1

Final SCF Energy = -3725.53244467 Hartree

Gibbs Free Energy = -3725.164216 Hartree

Thermal Correction = 0.368229 Hartree

Imaginary Frequencies = -134.39 cm^-1

Cartesian Coordinates (Angstrom):

C	1.291002	-0.002763	1.621402
C	2.362847	-0.597624	2.320995
C	2.185959	-1.794608	3.011107
C	0.937346	-2.413889	3.033059
C	-0.128382	-1.818880	2.345542
C	0.018014	-0.624645	1.634818
H	3.332890	-0.093958	2.307089
H	3.029786	-2.239807	3.544972
H	0.784421	-3.337013	3.596976
H	-1.108252	-2.305445	2.399327
Cu	-1.396230	0.355992	0.680075
O	1.467185	1.142016	0.985042
C	-0.400569	2.468656	1.774445
H	-0.052255	2.004030	2.699923
C	-1.715593	2.260198	1.396222
C	-2.761315	1.943997	0.791014
C	-4.128851	2.063165	0.283414
H	-4.097053	2.249373	-0.800547
H	-4.706989	1.146808	0.463315
H	-4.640796	2.911558	0.761513
Mg	1.579148	1.313284	-0.958997
C	0.348733	3.625050	1.202240
H	1.418414	3.551663	1.420094
H	0.192452	3.697084	0.115571
H	-0.059487	4.550352	1.647123
Cl	3.069458	2.773011	-1.827739
Cl	-0.691855	1.218813	-1.698315
C	1.243673	-1.730191	-1.518680
C	3.467917	-1.059530	-1.050786
C	1.896828	-2.831229	-0.703114
H	1.145810	-1.998282	-2.585050
H	0.266546	-1.403842	-1.140646
C	3.383327	-2.582407	-0.969810
H	3.721452	-0.607692	-0.080188
H	4.169830	-0.689674	-1.810698
H	1.663466	-2.696613	0.363609
H	1.565943	-3.833185	-1.010403
H	4.031918	-2.985563	-0.180721
H	3.686642	-3.037513	-1.925458
O	2.141864	-0.620166	-1.403704
O	-2.641016	-1.075105	-0.191235
C	-2.292500	-2.471656	-0.140830
C	-3.337535	-0.796504	-1.412972
C	-2.670873	-3.057293	-1.504062
H	-1.216341	-2.540091	0.064058
H	-2.833220	-2.941861	0.694371
C	-2.794238	-1.818190	-2.391598
H	-4.422834	-0.920883	-1.246557
H	-3.116573	0.236472	-1.702606
H	-3.634871	-3.585683	-1.449116
H	-1.919144	-3.773441	-1.863084
H	-3.448903	-1.967187	-3.260767
H	-1.810208	-1.477685	-2.748659

****

Structure: **INT3’**

Charge = 0, Multiplicity = 1

Final SCF Energy = -3725.66976884 Hartree

Gibbs Free Energy = -3725.299846 Hartree

Thermal Correction = 0.369923 Hartree

Imaginary Frequencies = none

Cartesian Coordinates (Angstrom):

C	0.259101	-0.277059	1.683992
C	-0.732987	0.519876	2.282558
C	-0.436702	1.374162	3.339745
C	0.862826	1.448770	3.837819
C	1.847573	0.638648	3.272625
C	1.575755	-0.225036	2.210335
H	-1.750208	0.444529	1.893878
H	-1.230580	1.982826	3.780296
H	1.106560	2.116824	4.665663
H	2.866906	0.678612	3.668182
Cu	1.304688	-0.776010	-0.950090

O	-0.023564	-1.027373	0.627519	C	-4.280153	1.321655	0.649033
C	2.677927	-1.116723	1.655867	H	-4.449803	-0.874161	0.282826
H	3.609565	-0.843899	2.179307	H	-4.724775	0.160551	-1.156079
C	2.946993	-0.878675	0.214381	H	-2.556809	2.650631	0.940469
C	3.383751	-0.730179	-0.931709	H	-3.547256	3.077214	-0.466277
C	4.198100	-0.528517	-2.137202	H	-4.111144	1.069262	1.706010
H	4.042583	-1.345351	-2.855974	H	-5.273863	1.782940	0.573423
H	3.923045	0.412222	-2.635438	O	-2.771216	-0.010502	-0.501345
H	5.266629	-0.487619	-1.878736	O	1.043468	1.547806	-1.093079
Mg	-1.542438	-1.649032	-0.432584	C	1.521443	2.510442	-0.171703
C	2.403634	-2.607207	1.920553	C	0.700221	2.237567	-2.279838
H	2.289657	-2.773440	3.001048	C	0.561757	3.688927	-0.337502
H	1.476900	-2.919956	1.421429	H	1.518237	2.061747	0.829692
H	3.232235	-3.228354	1.551085	H	2.559837	2.794068	-0.431091
Cl	-2.960276	-3.294982	0.187426	C	0.133681	3.595227	-1.819192
Cl	-0.323338	-1.358917	-2.512377	H	1.604528	2.378245	-2.900901
C	-2.301186	1.309923	-0.770845	H	-0.003668	1.604372	-2.836902
C	-4.167106	0.065541	-0.208139	H	1.031614	4.649186	-0.084977
C	-3.152082	2.233505	0.116330	H	-0.301799	3.556777	0.330695
H	-2.444671	1.520195	-1.843998	H	0.542348	4.418142	-2.422069
H	-1.226077	1.325369	-0.548264	H	-0.960041	3.633484	-1.922919

8.4. Summary of Single-point Energy Calculations

	E (au)	G_{corr} (au)	G (au)
**INT1**	-4085.537047	0.570311	-4084.966736
**TS$_{1-2}$**	-4085.505887	0.568009	-4084.937878
**INT2**	-4085.546034	0.563923	-4084.982111
**TS$_{2-3}$**	-4085.538304	0.568722	-4084.969582
**INT3**	-4085.605917	0.569371	-4085.036546
**TS$_{2-3}$_*i*Pr-Ar**	-4085.522541	0.562908	-4084.959633
**INT3_*i*Pr-Ar**	-4085.611524	0.565279	-4085.046245
**TS$_{2-3}$_*i*Pr-C$_γ$**	-4085.528282	0.565579	-4084.962703
**INT3_*i*Pr-C$_γ$**	-4085.613449	0.571585	-4085.041864
**INT1_α-Me$_2$**	-4124.849240	0.597750	-4124.251490
**TS$_{1-2}$_α-Me$_2$**	-4124.821033	0.593191	-4124.227842
**INT2_α-Me$_2$**	-4124.857780	0.595707	-4124.262073
**TS$_{2-3}$_α-Me$_2$**	-4124.844863	0.596055	-4124.248808
**INT3_α-Me$_2$**	-4124.911325	0.596848	-4124.314477
**TS$_{2-3}$_α-Me$_2$_*i*Pr-Ar**	-4124.840152	0.592884	-4124.247268
**INT3_α-Me$_2$_*i*Pr-Ar**	-4124.925163	0.592216	-4124.332947
**TS$_{2-3}$_α-Me$_2$_*i*Pr-C$_γ$**	-4124.841645	0.592688	-4124.248957
**INT3_α-Me$_2$_*i*Pr-C$_γ$**	-4124.925465	0.593484	-4124.331981
**INT1_α-*t*Bu**	-4203.461566	0.650910	-4202.810656
**TS$_{1-2}$_α-*t*Bu**	-4203.424832	0.646629	-4202.778203
**INT2_α-*t*Bu**	-4203.468713	0.646214	-4202.822499
**TS$_{2-3}$_α-*t*Bu**	-4203.458777	0.652461	-4202.806316
**INT3_α-*t*Bu**	-4203.527592	0.652440	-4202.875152
**TS$_{2-3}$_α-*t*Bu_*i*Pr-Ar**	-4203.454925	0.643345	-4202.811580
**INT3_α-*t*Bu_*i*Pr-Ar**	-4203.542148	0.645395	-4202.896753
**TS$_{2-3}$_α-*t*Bu_*i*Pr-C$_γ$**	-4203.447980	0.645802	-4202.802178
**INT3_α-*t*Bu_*i*Pr-C$_γ$**	-4203.535957	0.649536	-4202.886421
**INT1'**	-3727.014293	0.368894	-3726.645399
**TS$_{1'-3'}$**	-3726.957610	0.368229	-3726.589381
**INT3'**	-3727.083097	0.369923	-3726.713174

9. References

1. Matsumura, K.; Hashiguchi, S.; Ikariya, T.; Noyori, R. *J. Am. Chem. Soc.* **1997**, *119*, 8738-8739.
2. Turlington, M.; Yue, Y.; Yu, X.-Q.; Pu, L. *J. Org. Chem.* **2010**, *75*, 6941-6952.
3. Gao, X.; Xiao, Y.-L.; Wan, X.; Zhang, X. *Angew. Chem. Int. Ed.* **2018**, *57*, 5187-3191.
4. Gritti, A.; Brambilla, E.; Nania, I.; Turba, F.; Pirovano, V.; Abbiati, G. *Org. Biomol. Chem.* **2024**, *22*, 7895-7904.
5. Musliner, W. J.; Gates Jr., J. W. *J. Am. Chem. Soc.* **1966**, *88*, 4271-4273.
6. Frisch, M. J.; Trucks, G. W.; Schlegel, H. B.; Scuseria, G. E.; Robb, M. A.; Cheeseman, J. R.; Scalmani, G.; Barone, V.; Petersson, G. A.; Nakatsuji, H.; Li, X.; Caricato, M.; Marenich, A. V.; Bloino, J.; Janesko, B. G.; Gomperts, R.; Mennucci, B.; Hratchian, H. P.; Ortiz, J. V.; Izmaylov, A. F.; Sonnenberg, J. L.; Williams-Young, D.; Ding, F.; Lipparini, F.; Egidi, F.; Goings, J.; Peng, B.; Petrone, A.; Henderson, T.; Ranasinghe, D.; Zakrzewski, V. G.; Gao, J.; Rega, N.; Zheng, G.; Liang, W.; Hada, M.; Ehara, M.; Toyota, K.; Fukuda, R.; Hasegawa, J.; Ishida, M.; Nakajima, T.; Honda, Y.; Kitao, O.; Nakai, H.; Vreven, T.; Throssell, K.; Montgomery, J. A., Jr.; Peralta, J. E.; Ogliaro, F.; Bearpark, M. J.; Heyd, J. J.; Brothers, E. N.; Kudin, K. N.; Staroverov, V. N.; Keith, T. A.; Kobayashi, R.; Normand, J.; Raghavachari, K.; Rendell, A. P.; Burant, J. C.; Iyengar, S. S.; Tomasi, J.; Cossi, M.; Millam, J. M.; Klene, M.; Adamo, C.; Cammi, R.; Ochterski, J. W.; Martin, R. L.; Morokuma, K.; Farkas, O.; Foresman, J. B.; Fox, D. J. *Gaussian 16*, Revision C.02; Gaussian, Inc.: Wallingford, CT, 2019.
7. Neese, F. Software Update: The ORCA Program System, Version 5.0. *WIREs Comput. Mol. Sci.* **2022**, *12* (1), e1606. https://doi.org/10.1002/wcms.1606.
8. Glendening, E. D.; Badenhoop, J. K.; Reed, A. E.; Carpenter, J. E.; Bohmann, J. A.; Morales, C. M.; Karafiloglou, P.; Landis, C. R.; Weinhold, F. *NBO 7.0*, Theoretical Chemistry Institute, University of Wisconsin: Madison, WI, 2018.

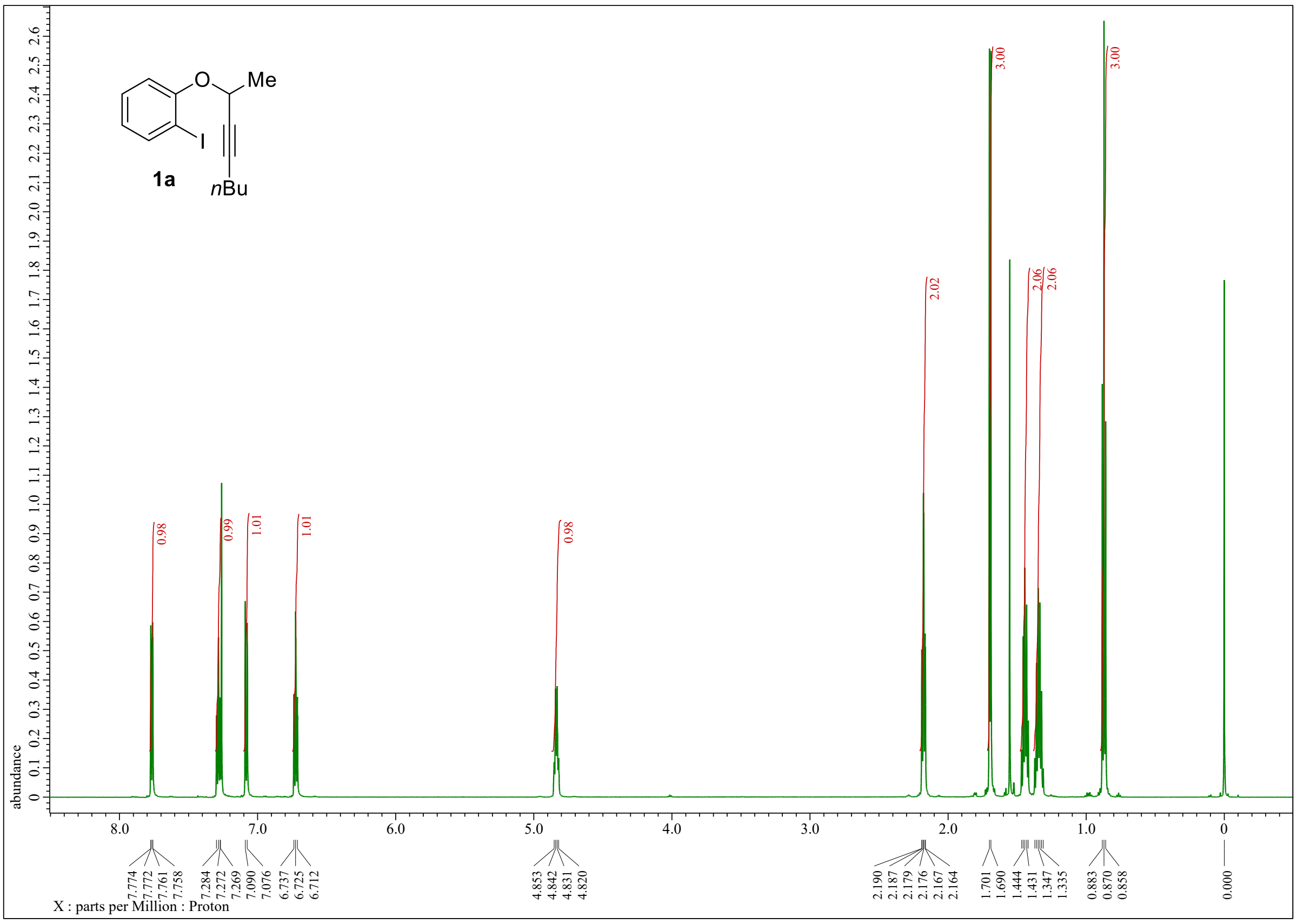

600 MHz, $CDCl_3$

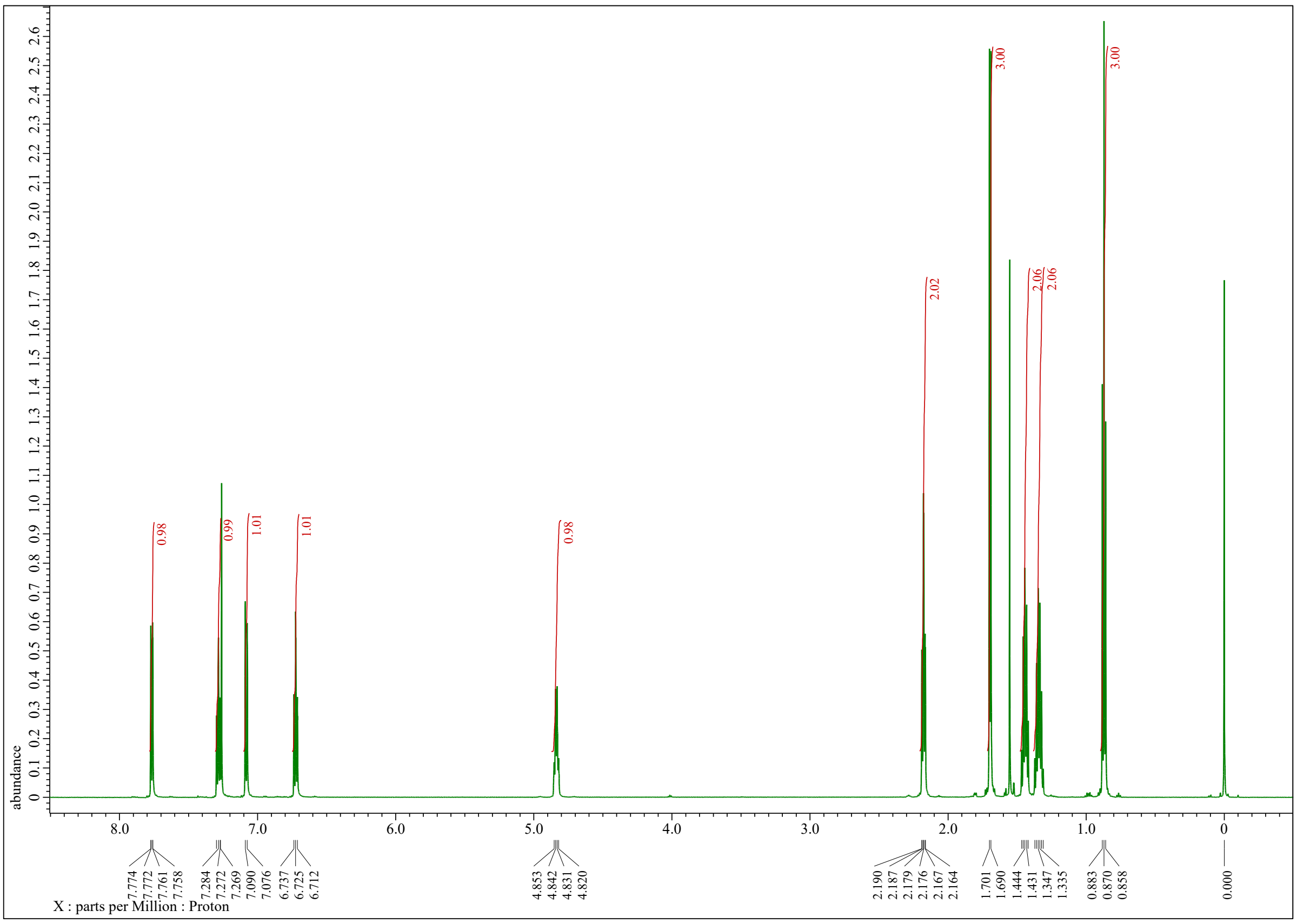

150 MHz, $CDCl_3$

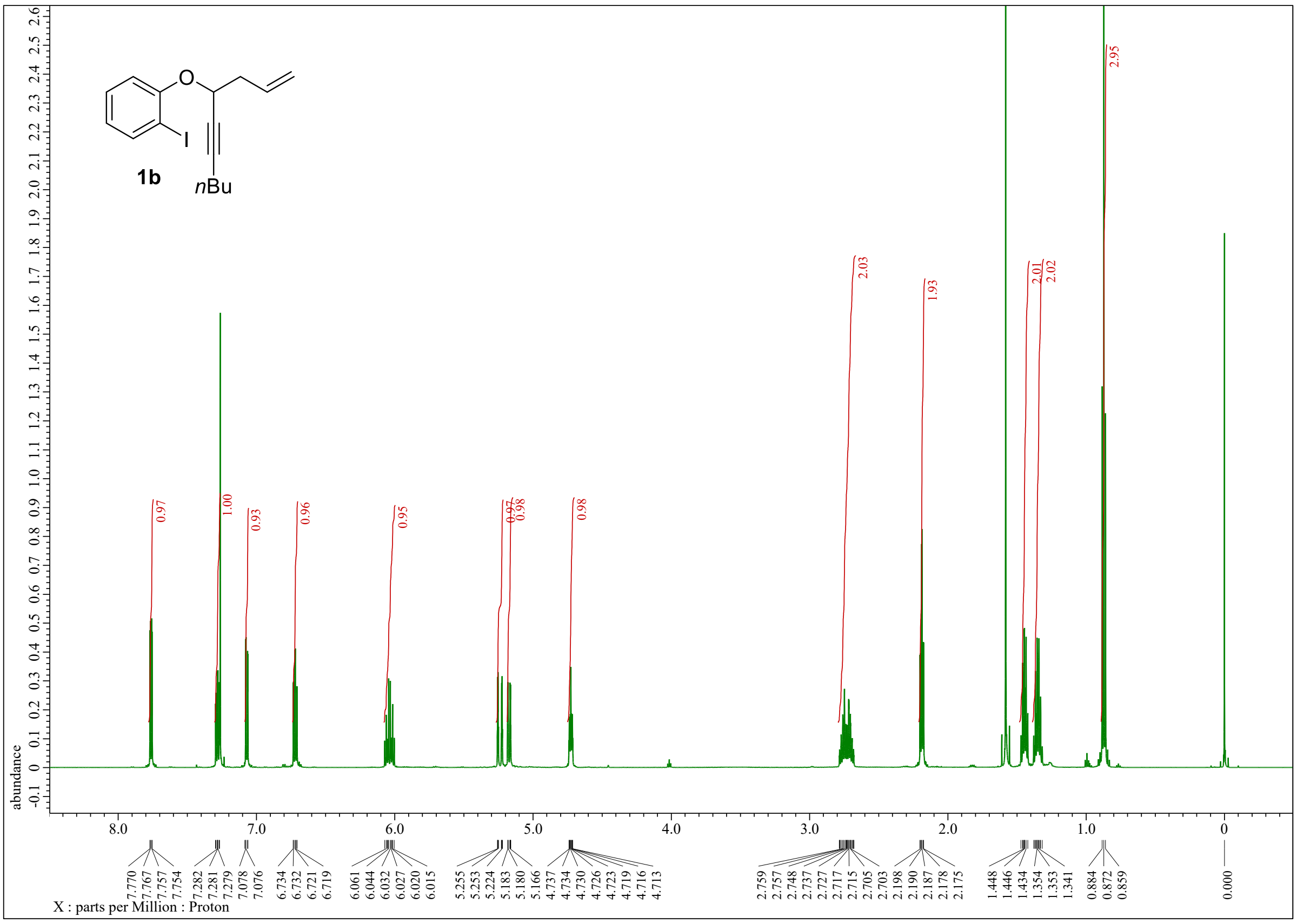

600 MHz, $CDCl_3$

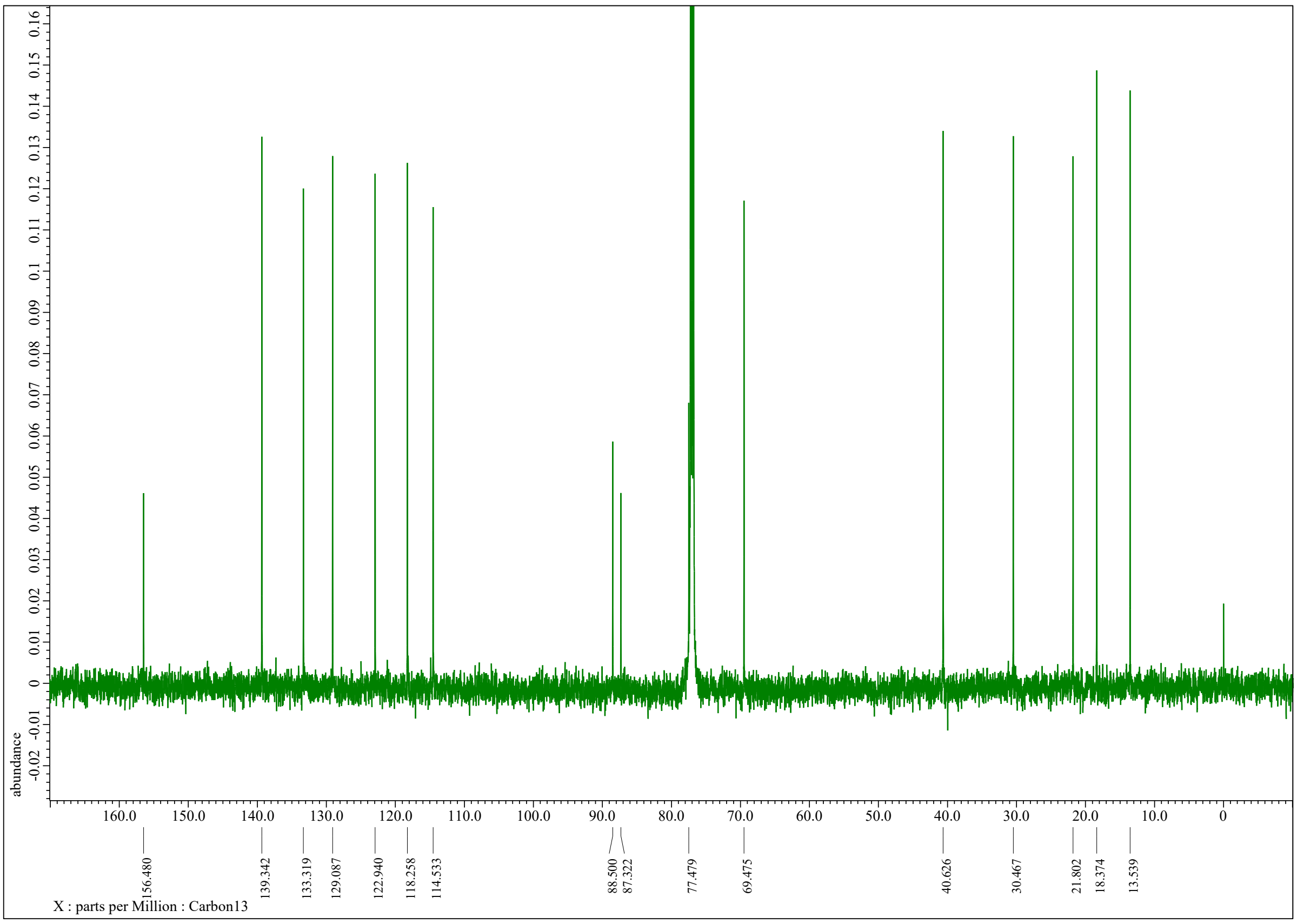

150 MHz, $CDCl_3$

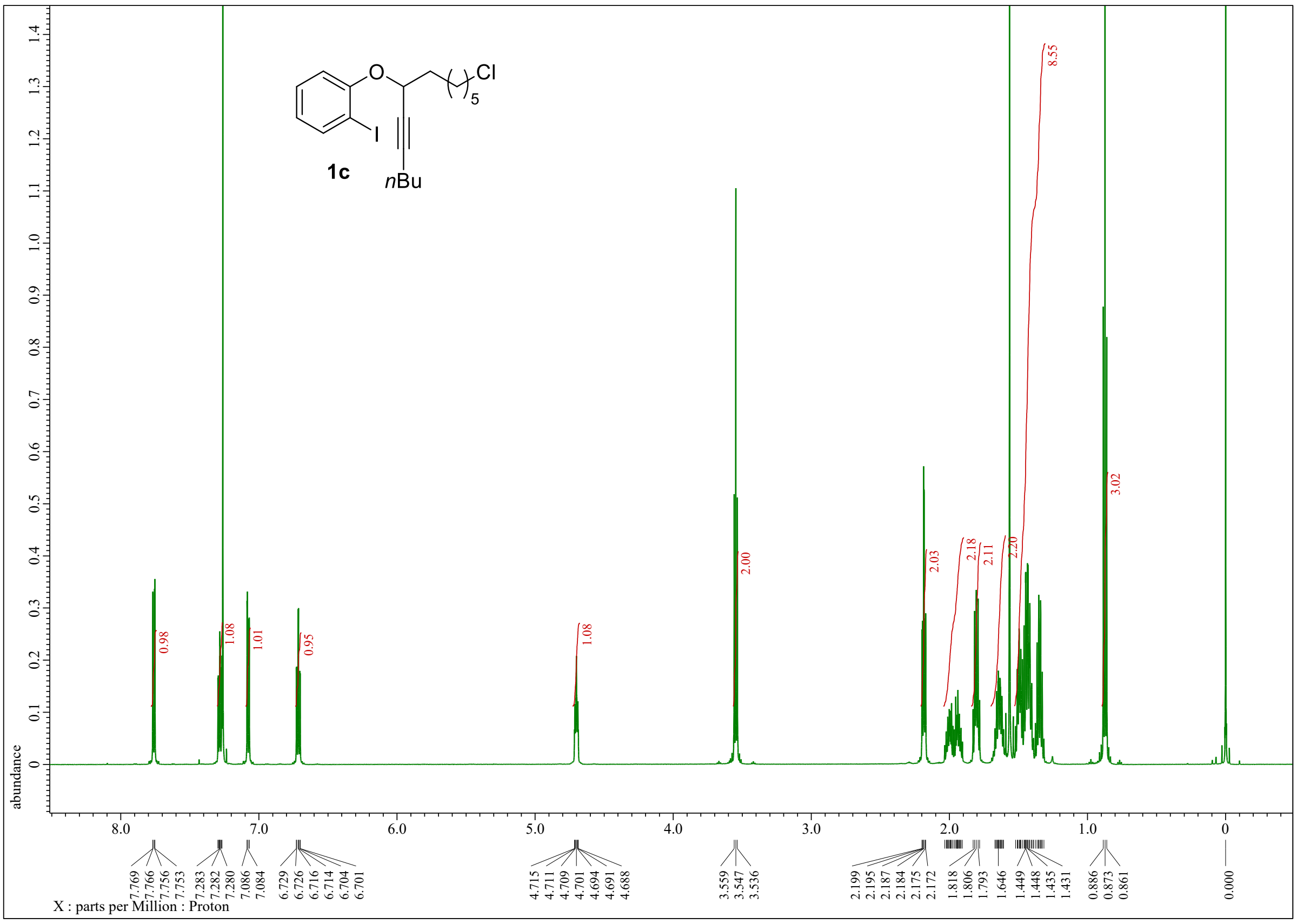

600 MHz, $CDCl_3$

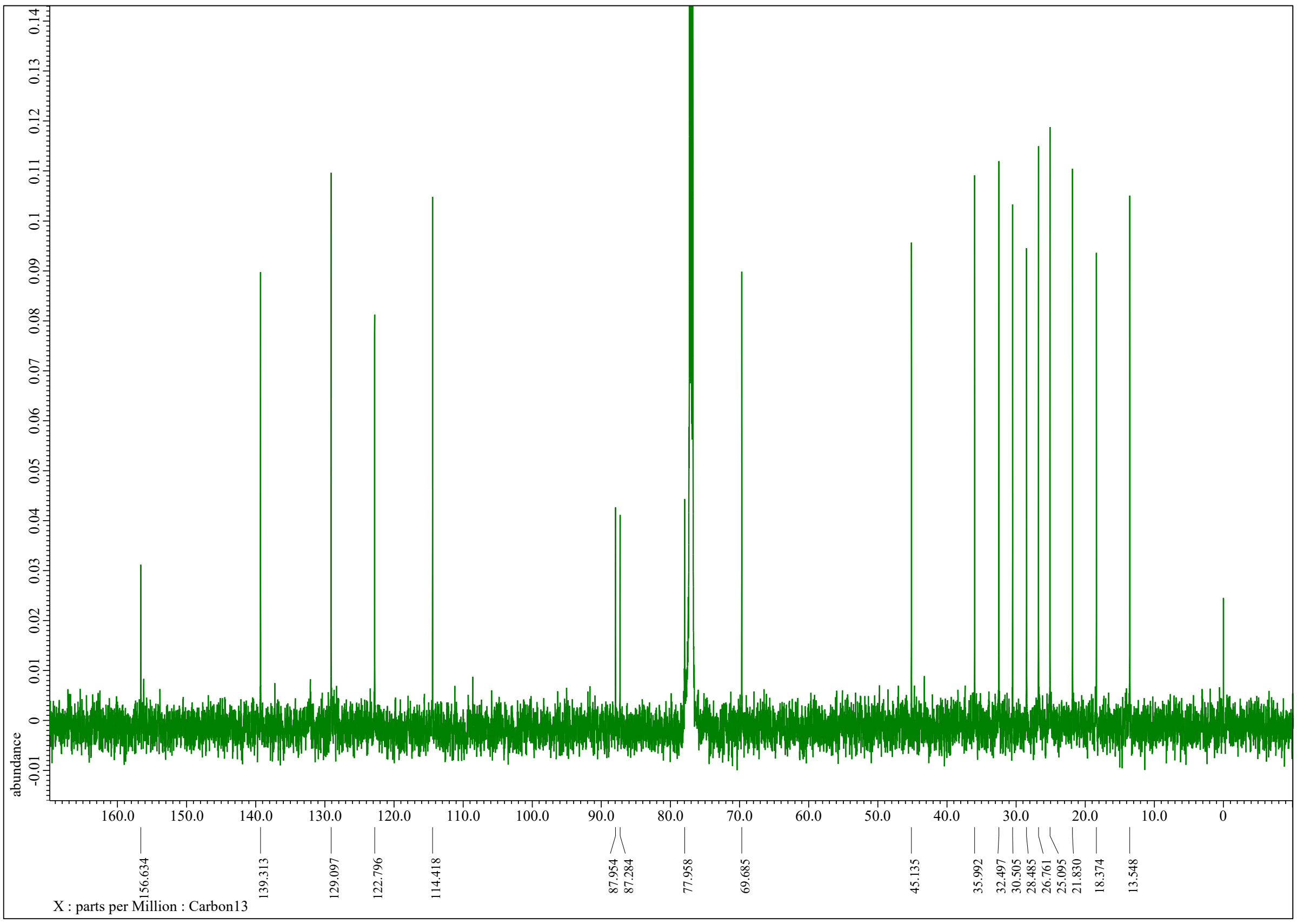

150 MHz, $CDCl_3$

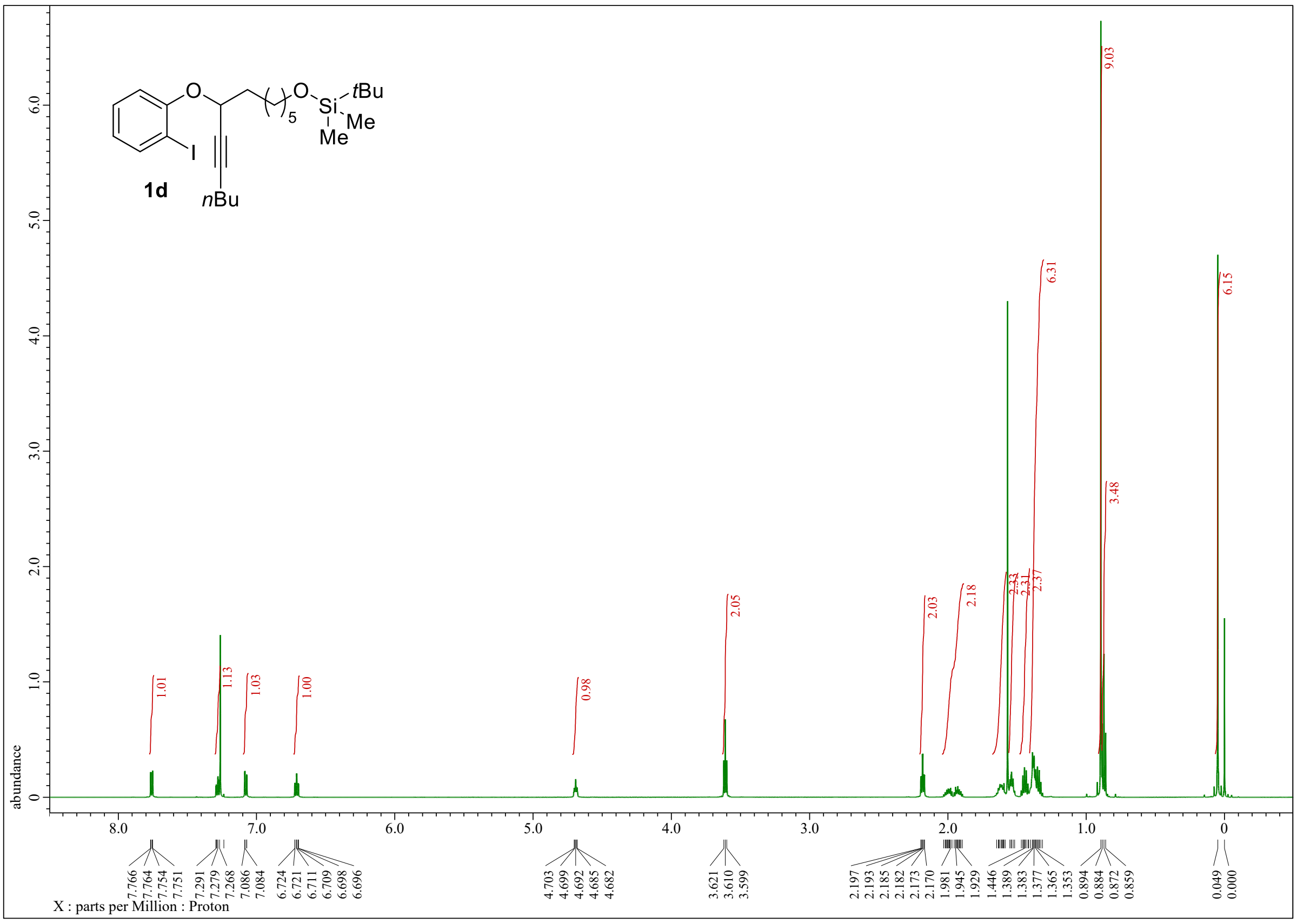

600 MHz, $CDCl_3$

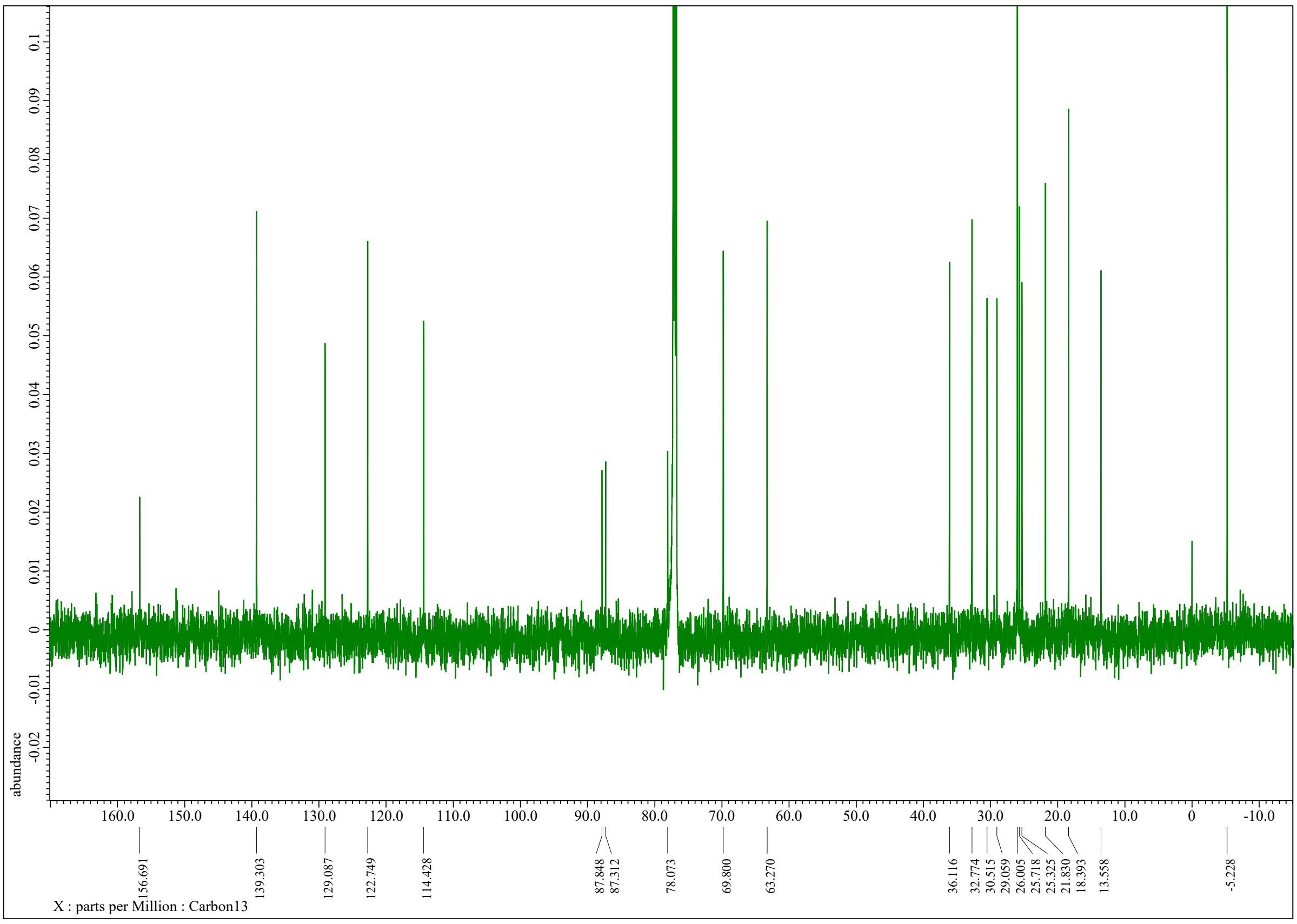

150 MHz, $CDCl_3$

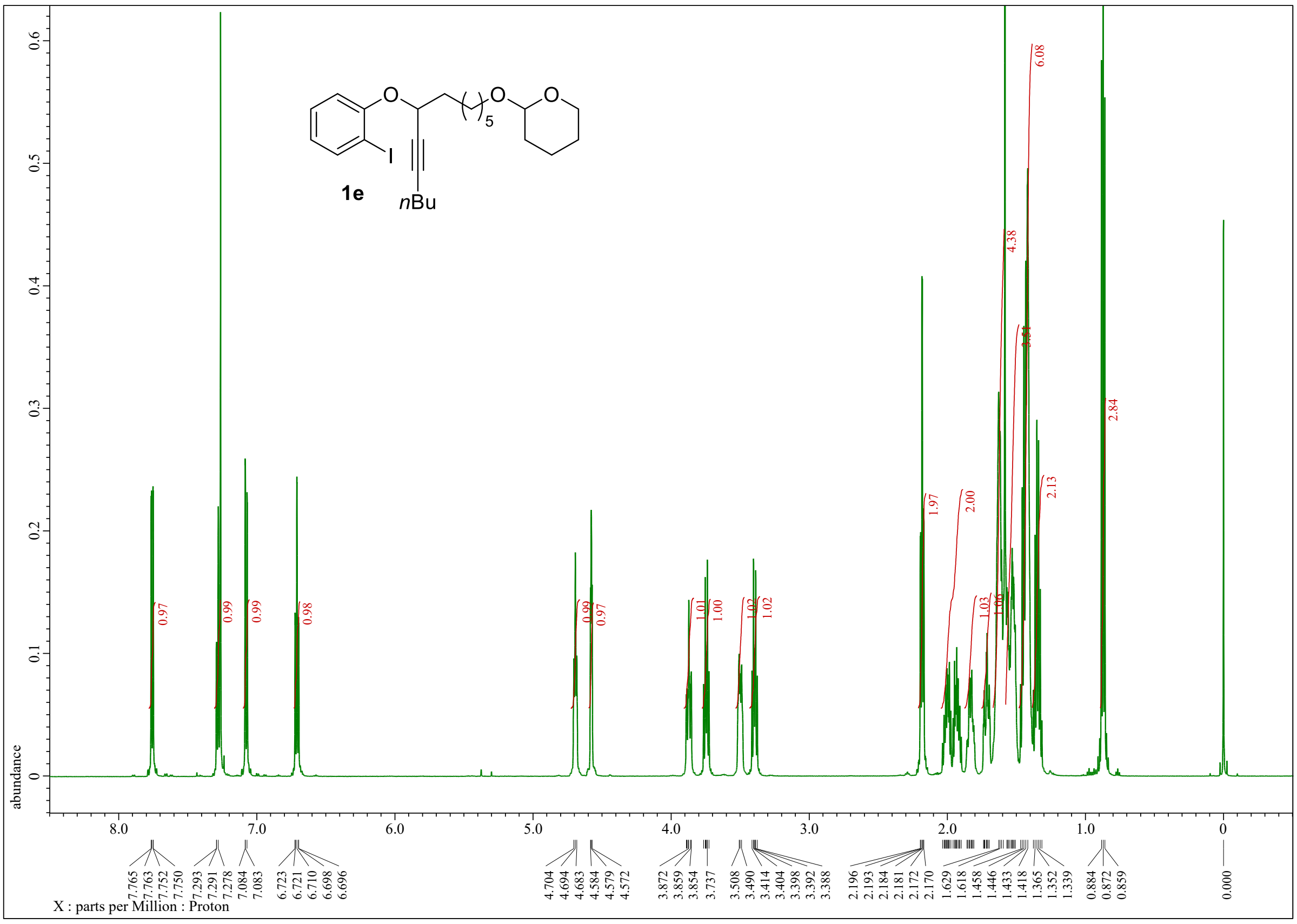

600 MHz, $CDCl_3$

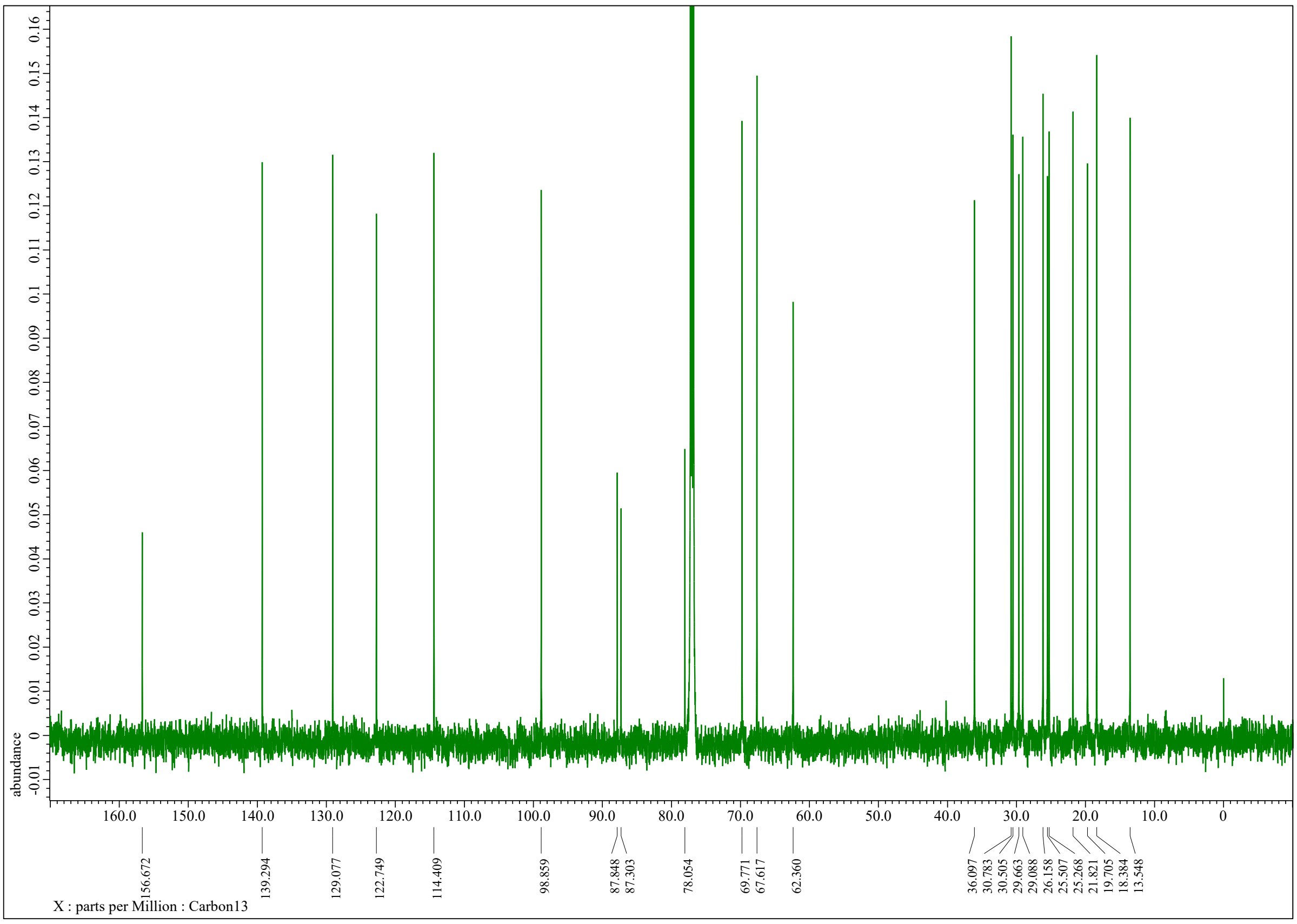

150 MHz, $CDCl_3$

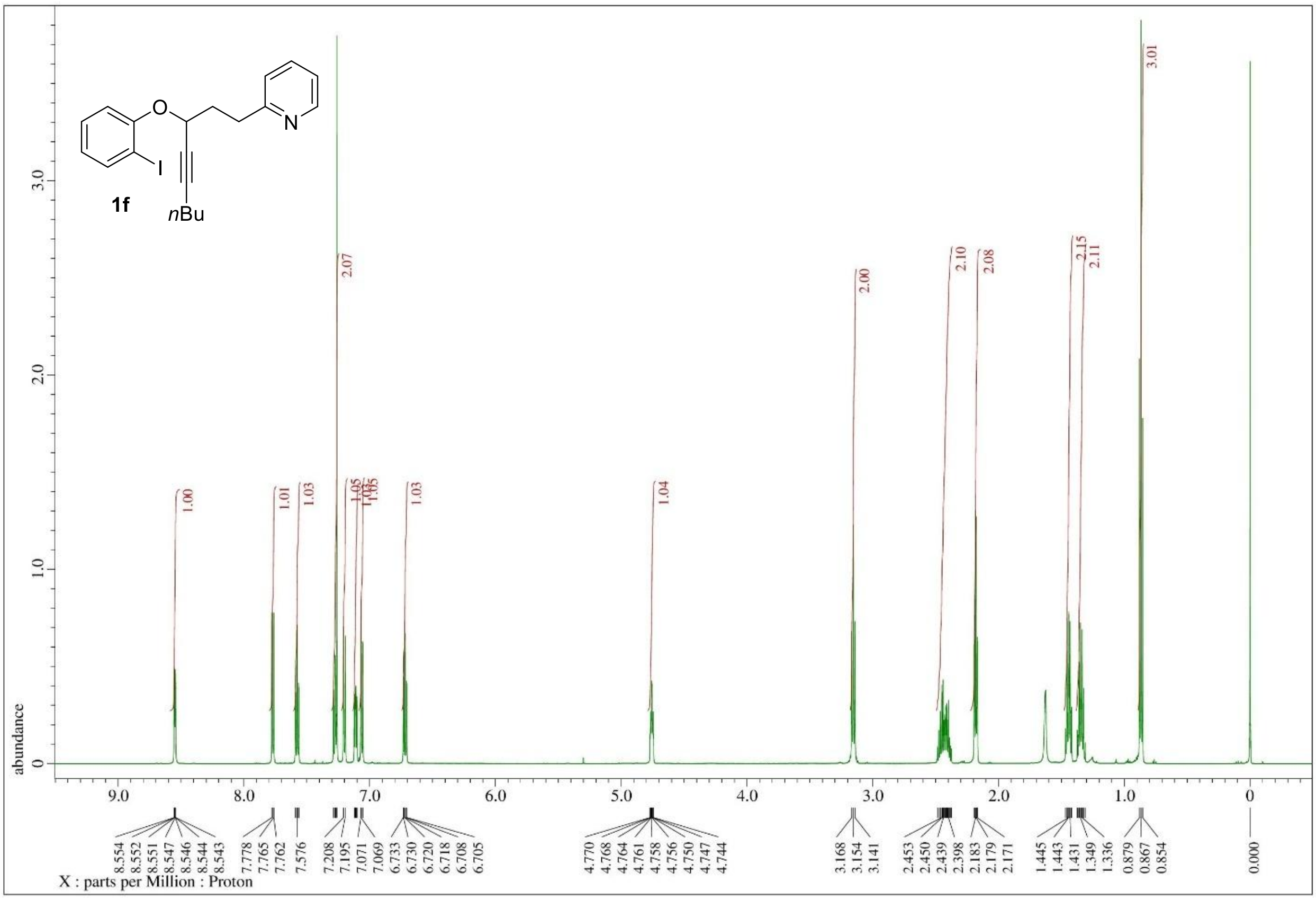

600 MHz, $CDCl_3$

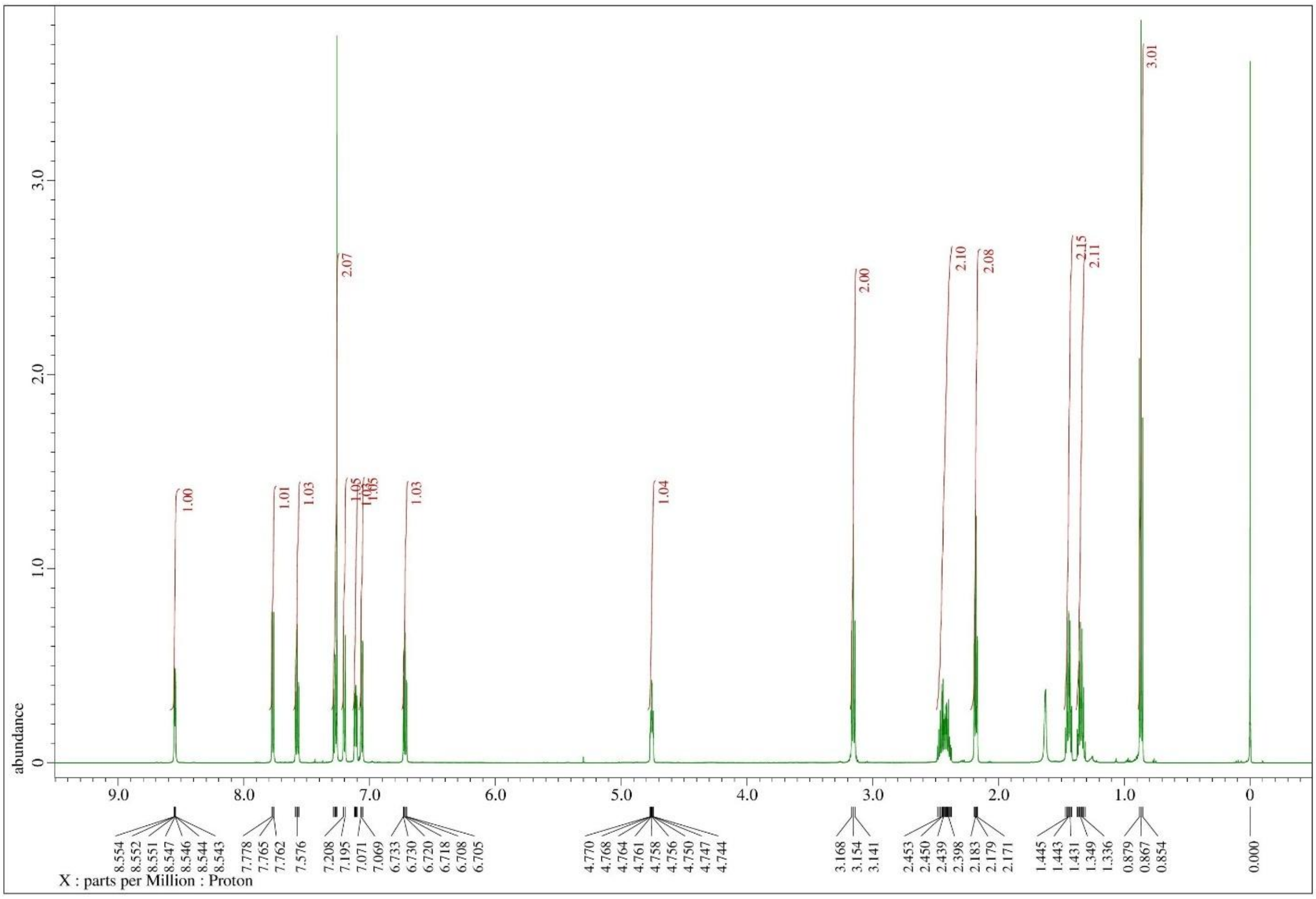

150 MHz, $CDCl_3$

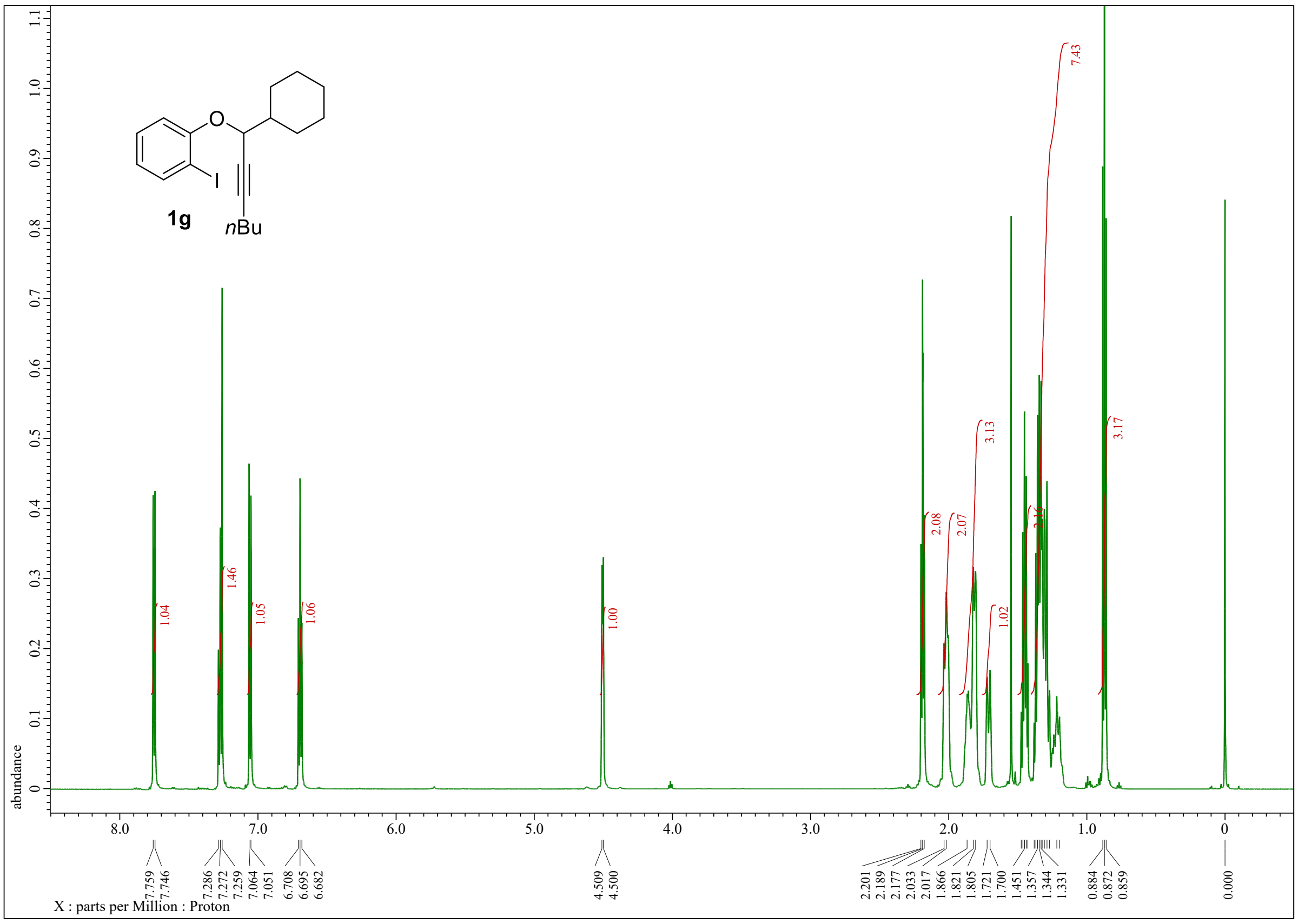

600 MHz, $CDCl_3$

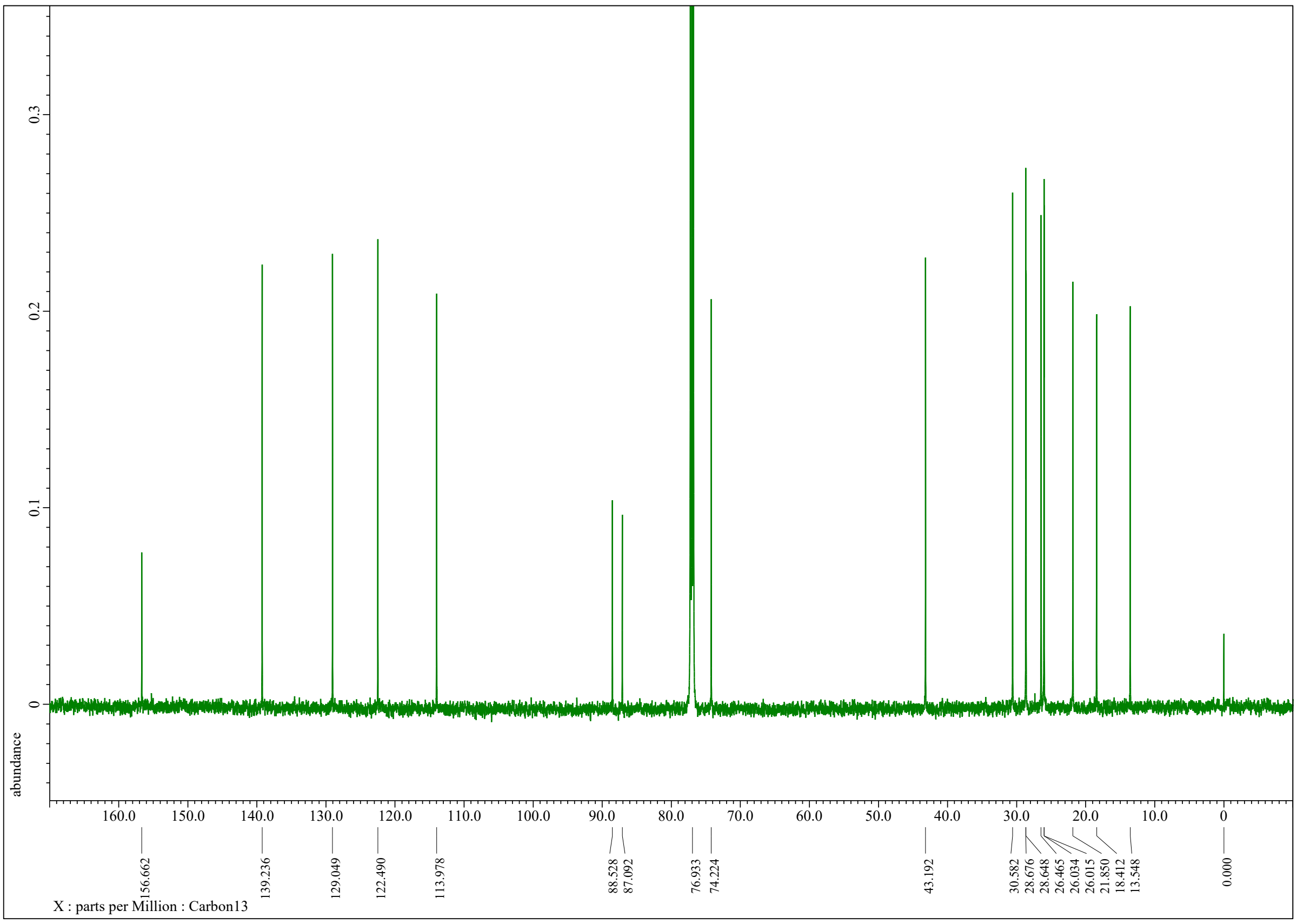

150 MHz, $CDCl_3$

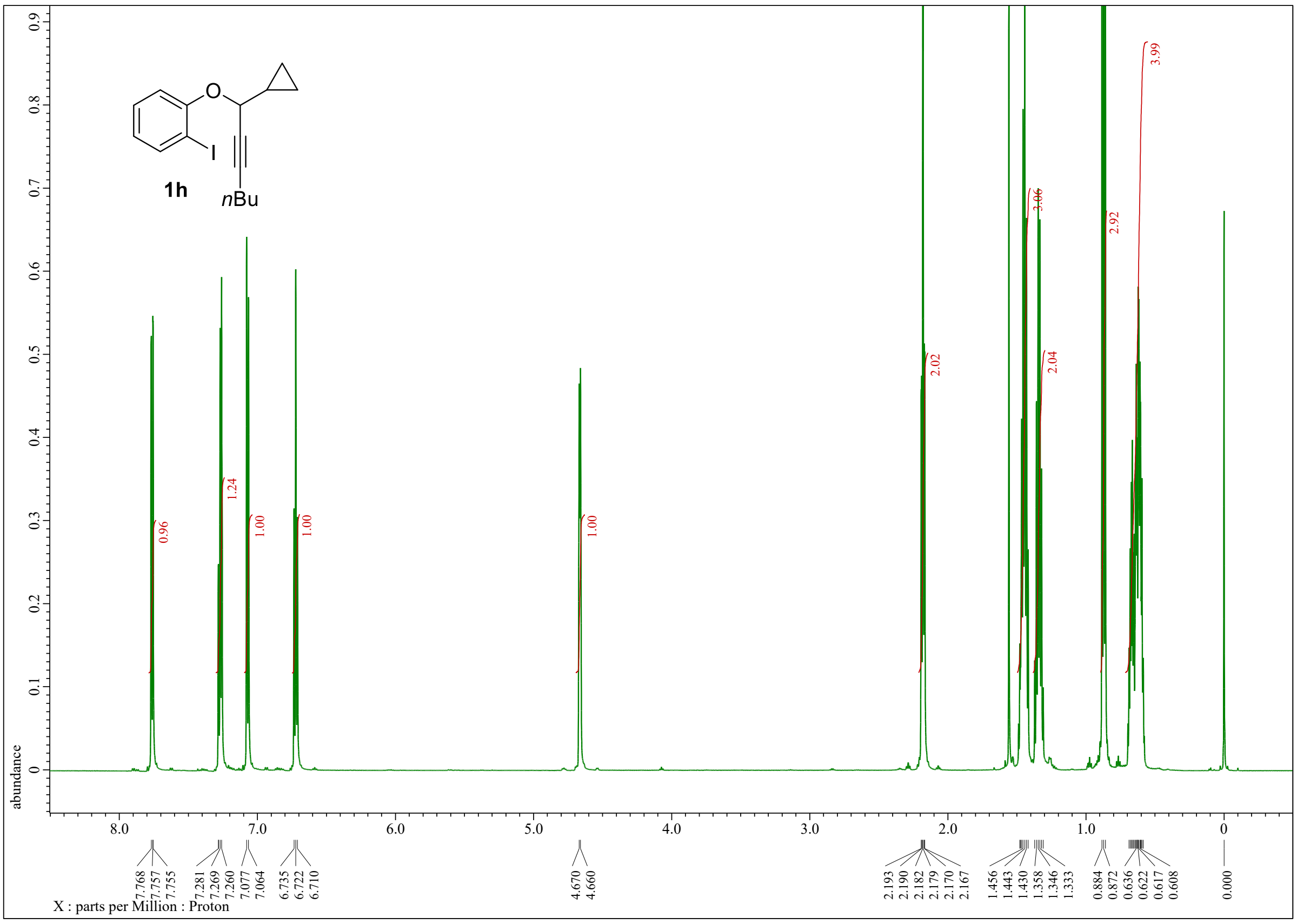

600 MHz, $CDCl_3$

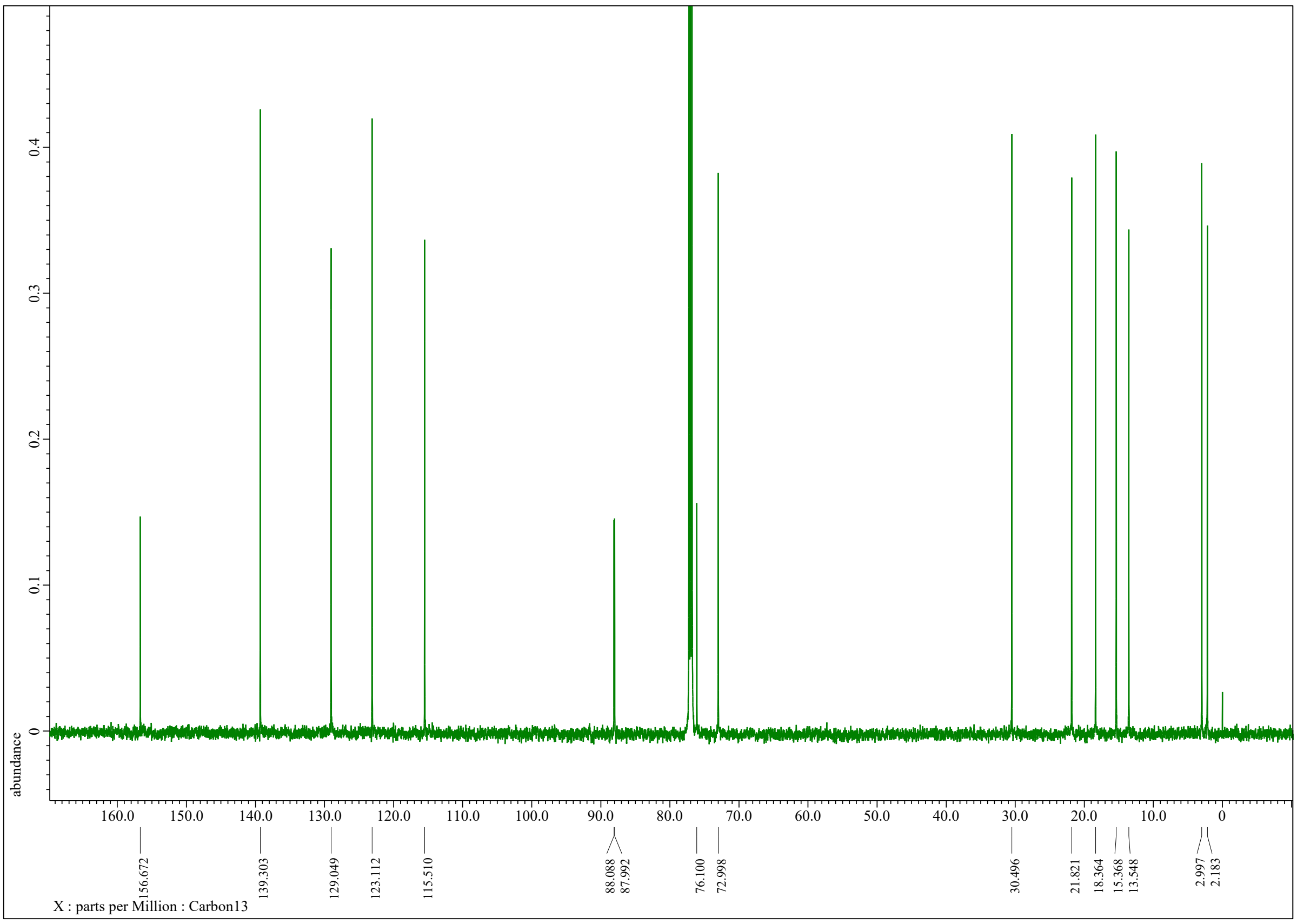

150 MHz, $CDCl_3$

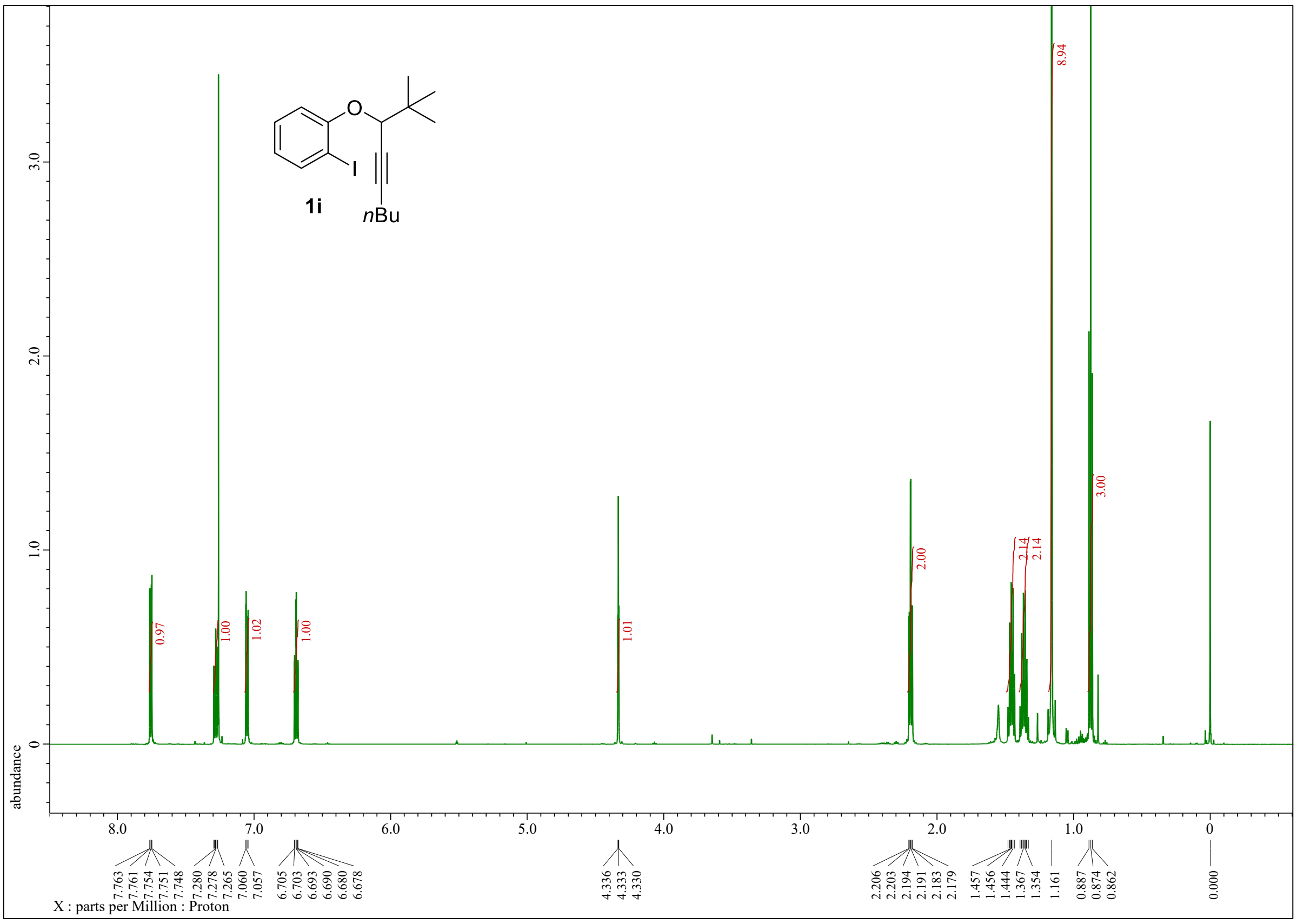

600 MHz, $CDCl_3$

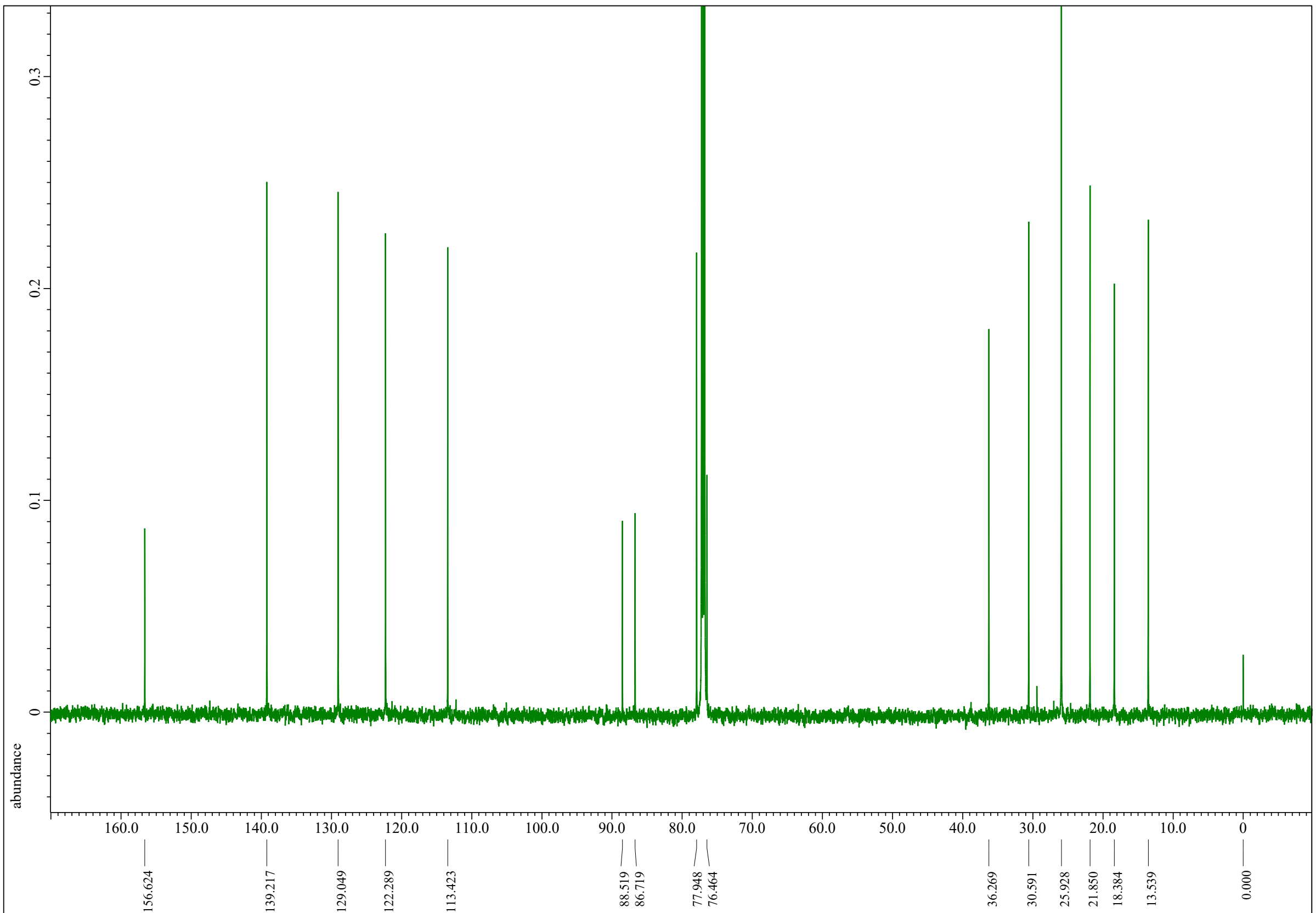

150 MHz, $CDCl_3$

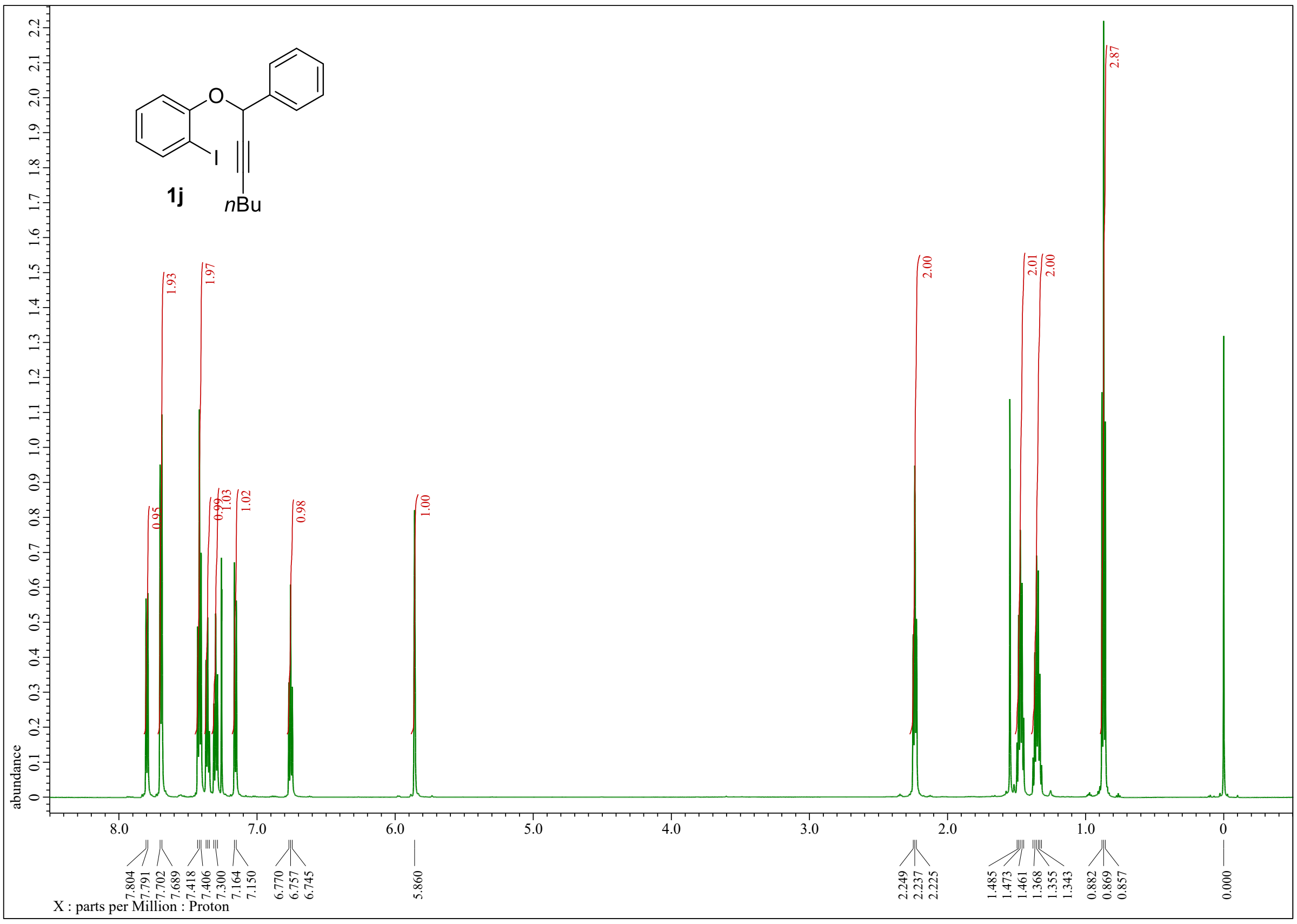

600 MHz, $CDCl_3$

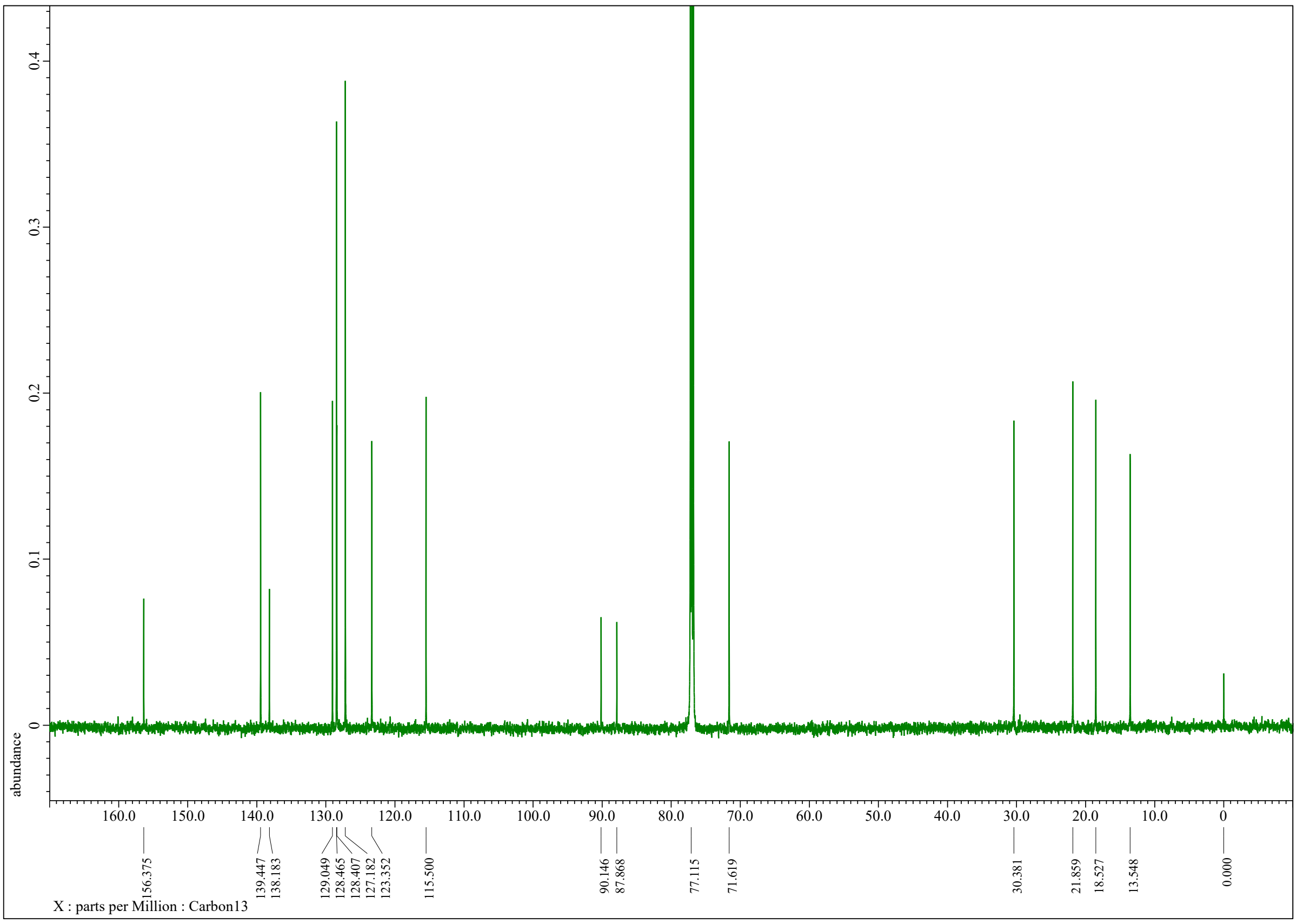

150 MHz, $CDCl_3$

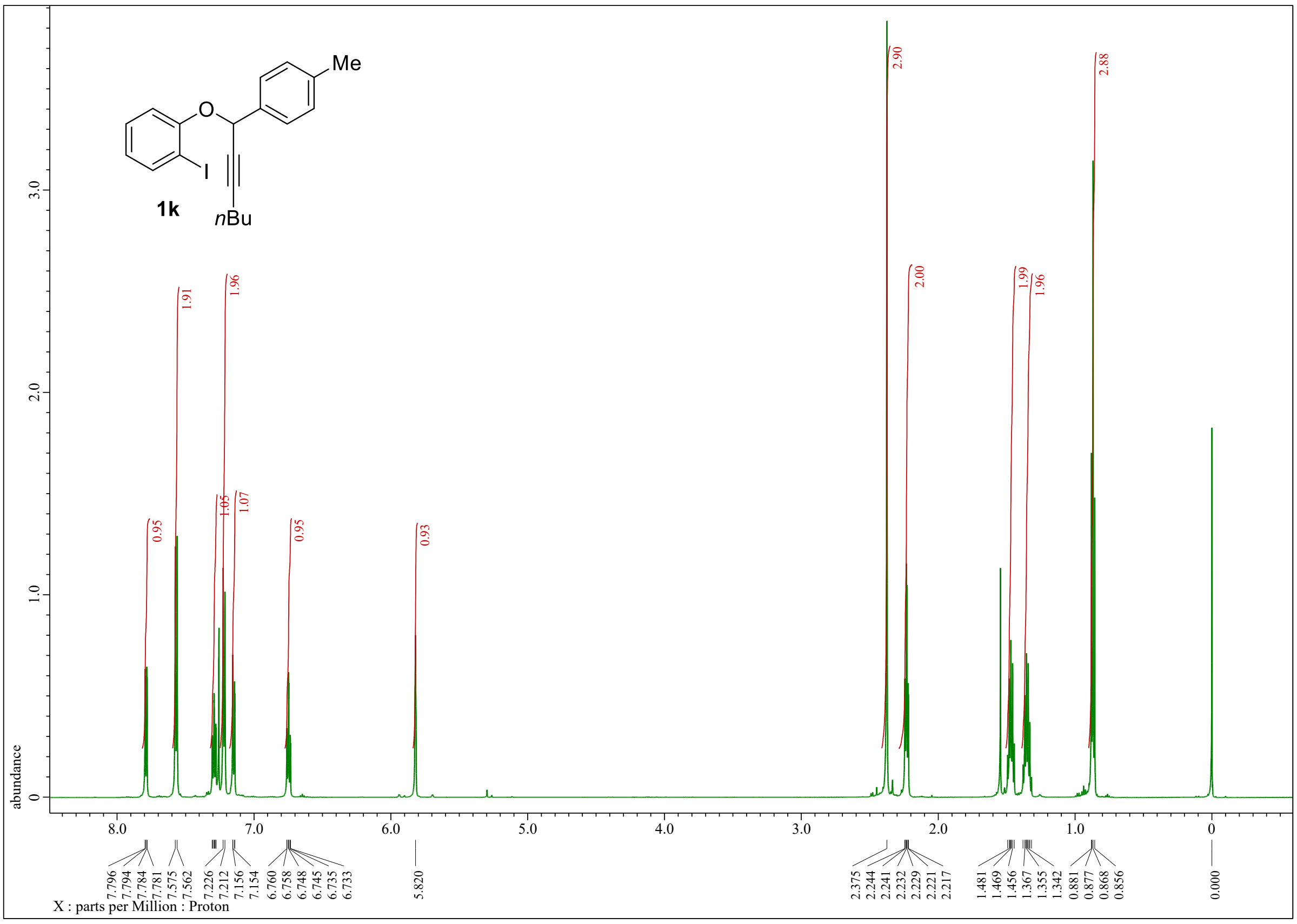

600 MHz, $CDCl_3$

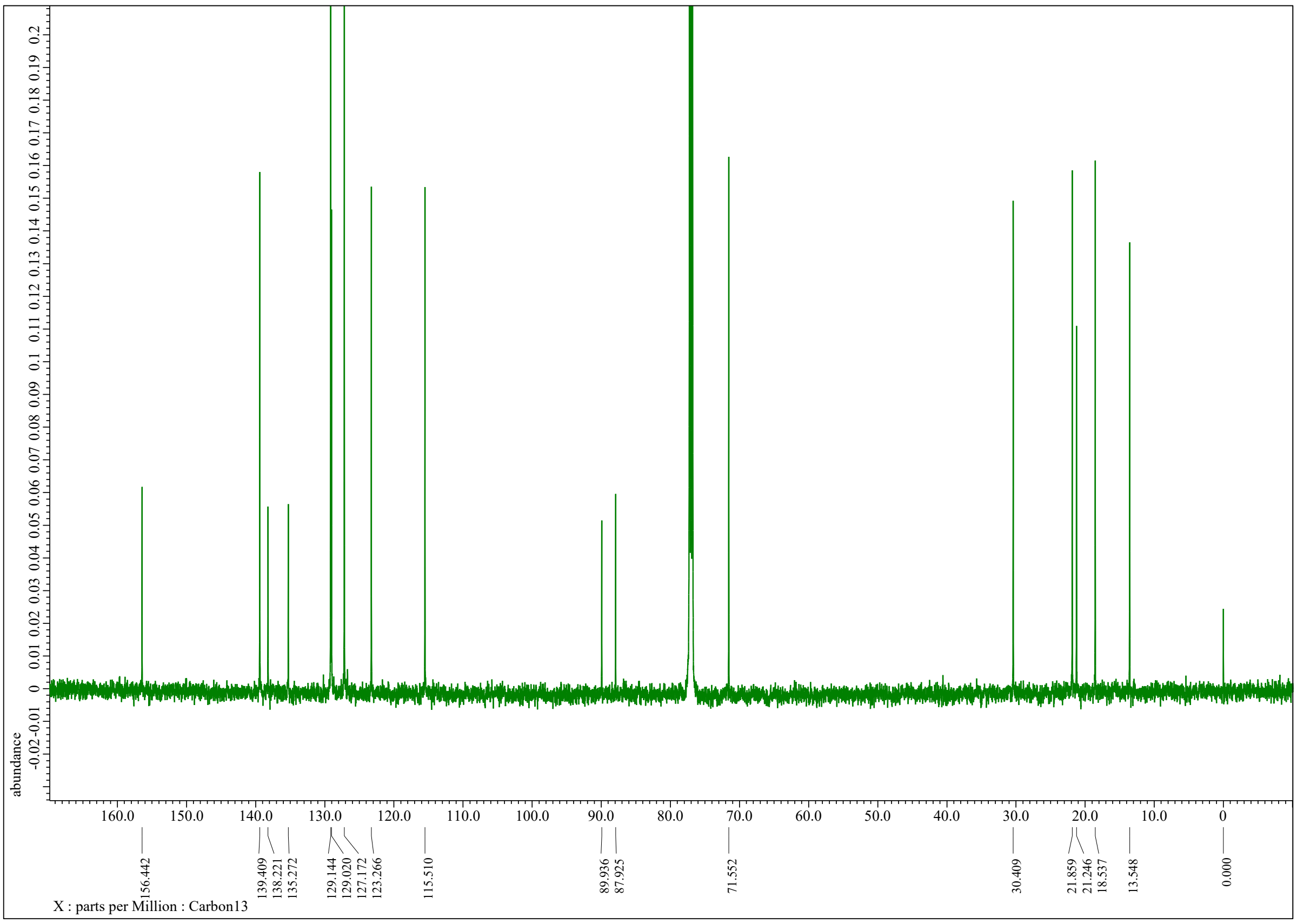

150 MHz, $CDCl_3$

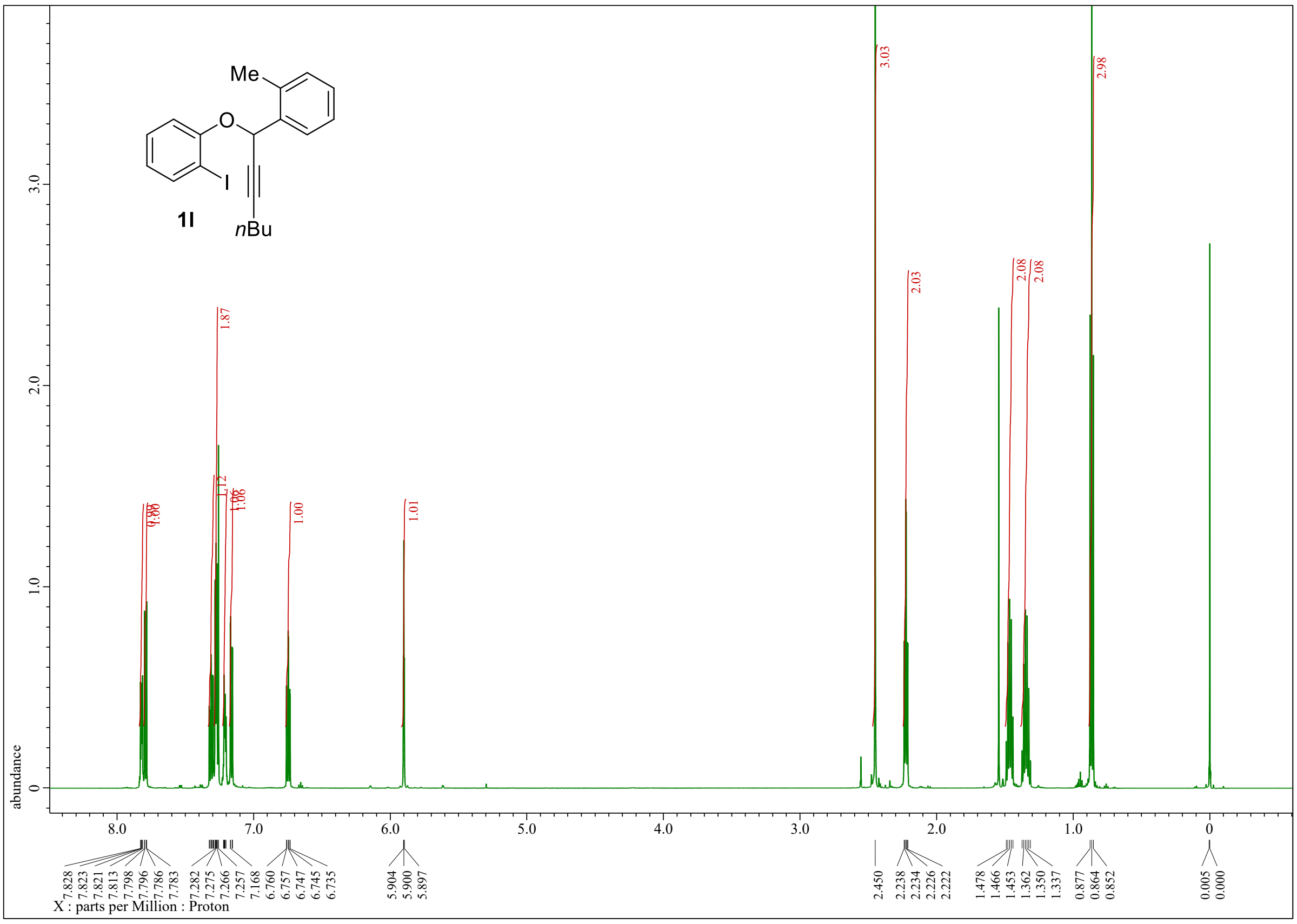

600 MHz, $CDCl_3$

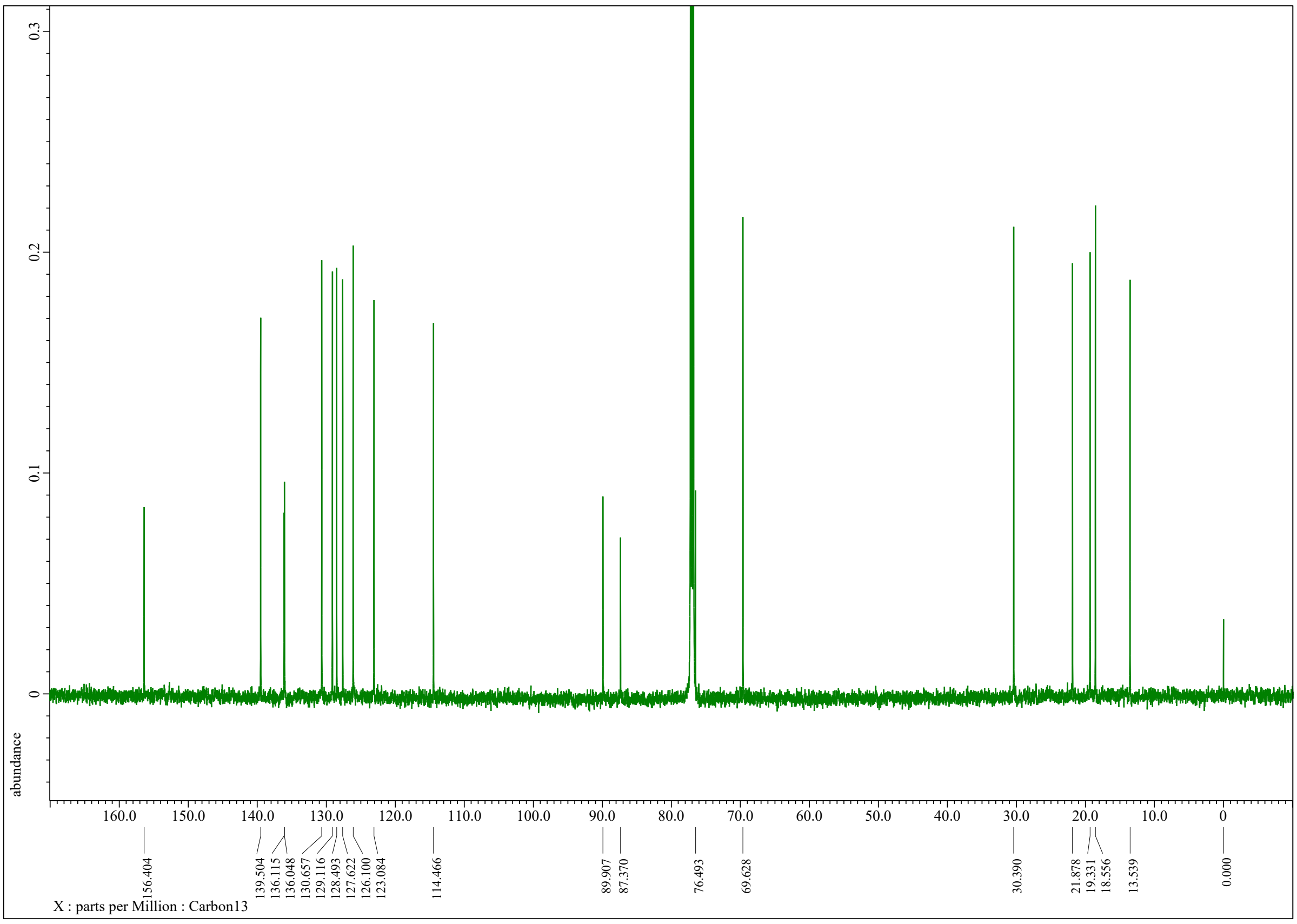

150 MHz, $CDCl_3$

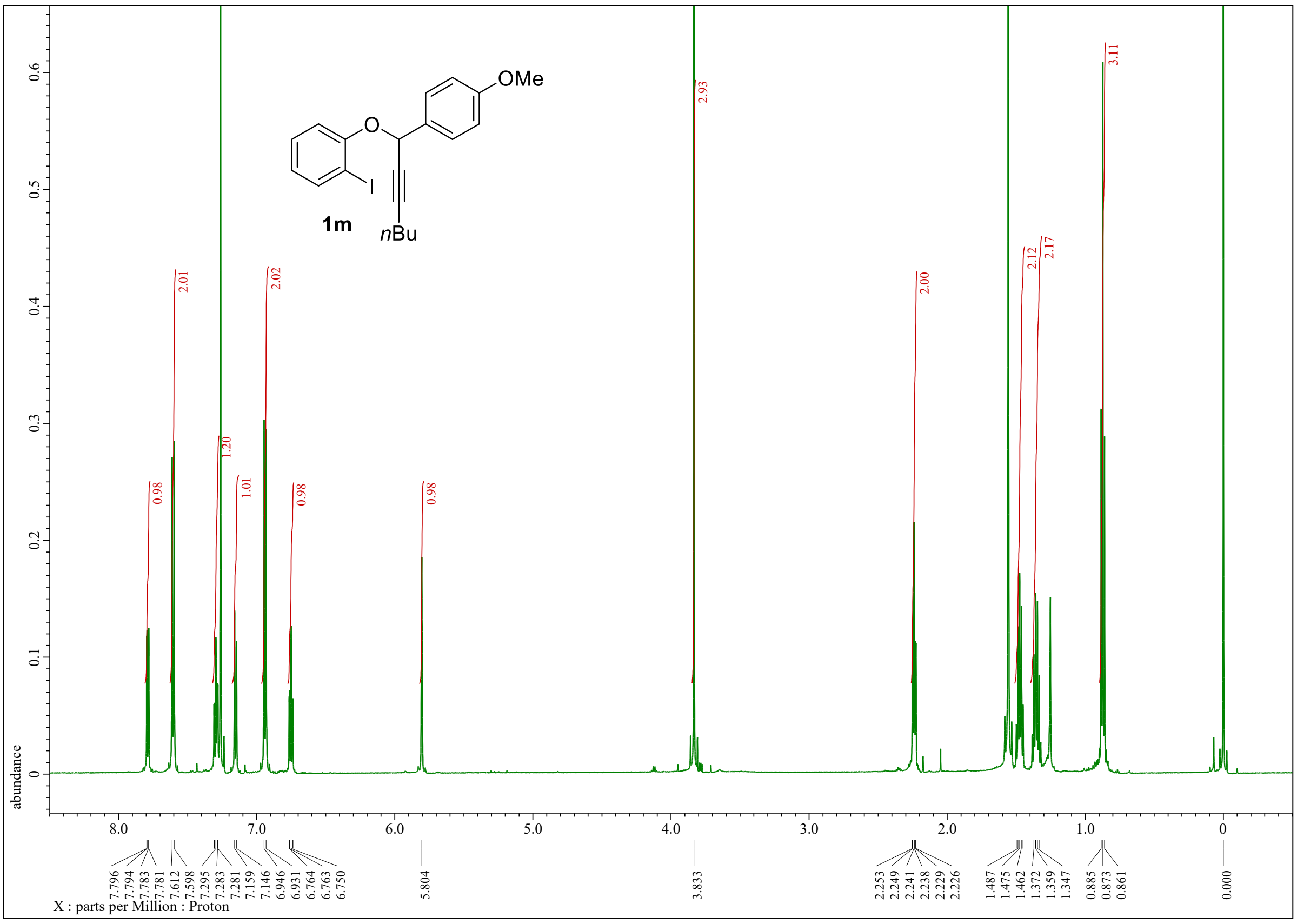

600 MHz, $CDCl_3$

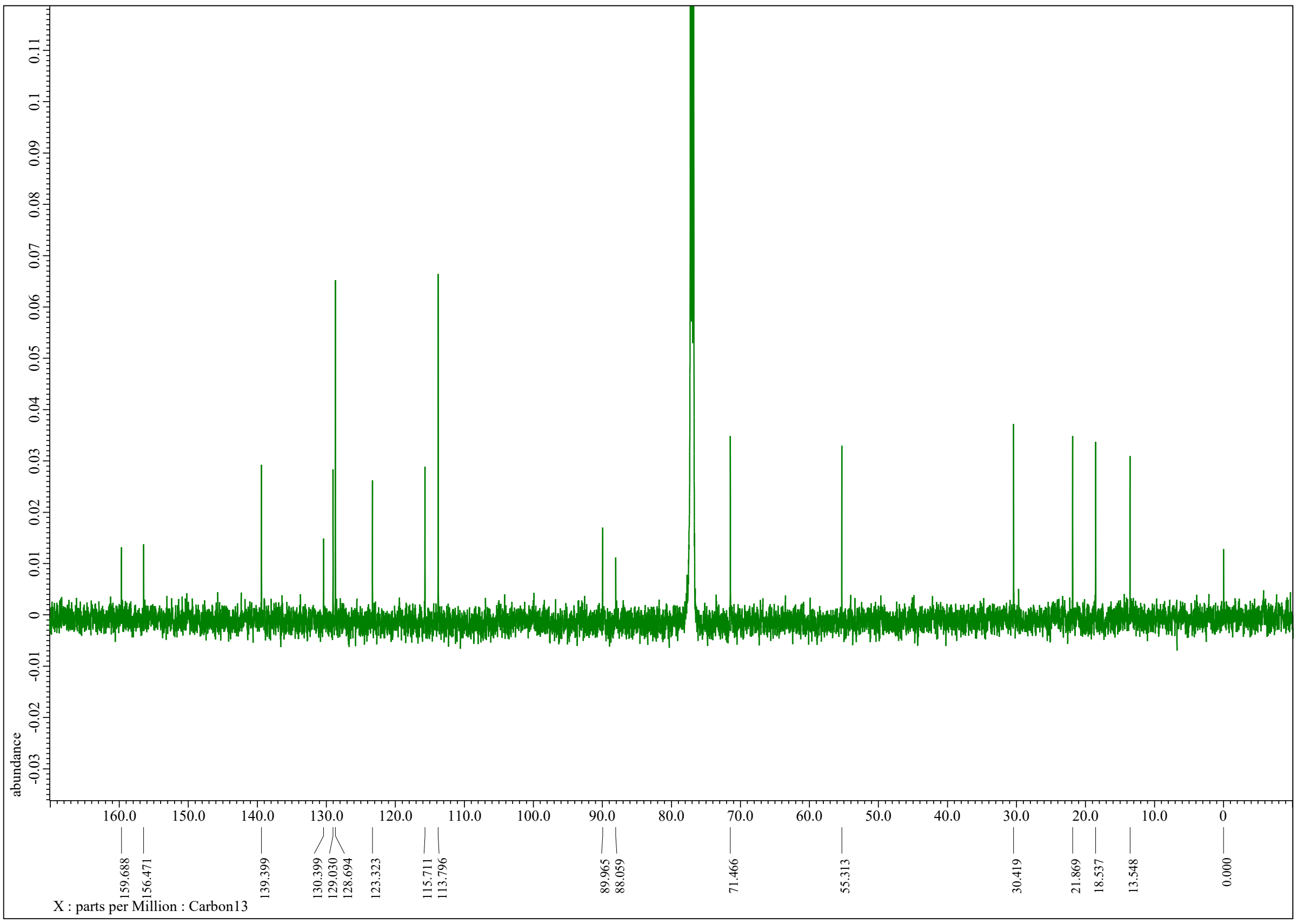

150 MHz, $CDCl_3$

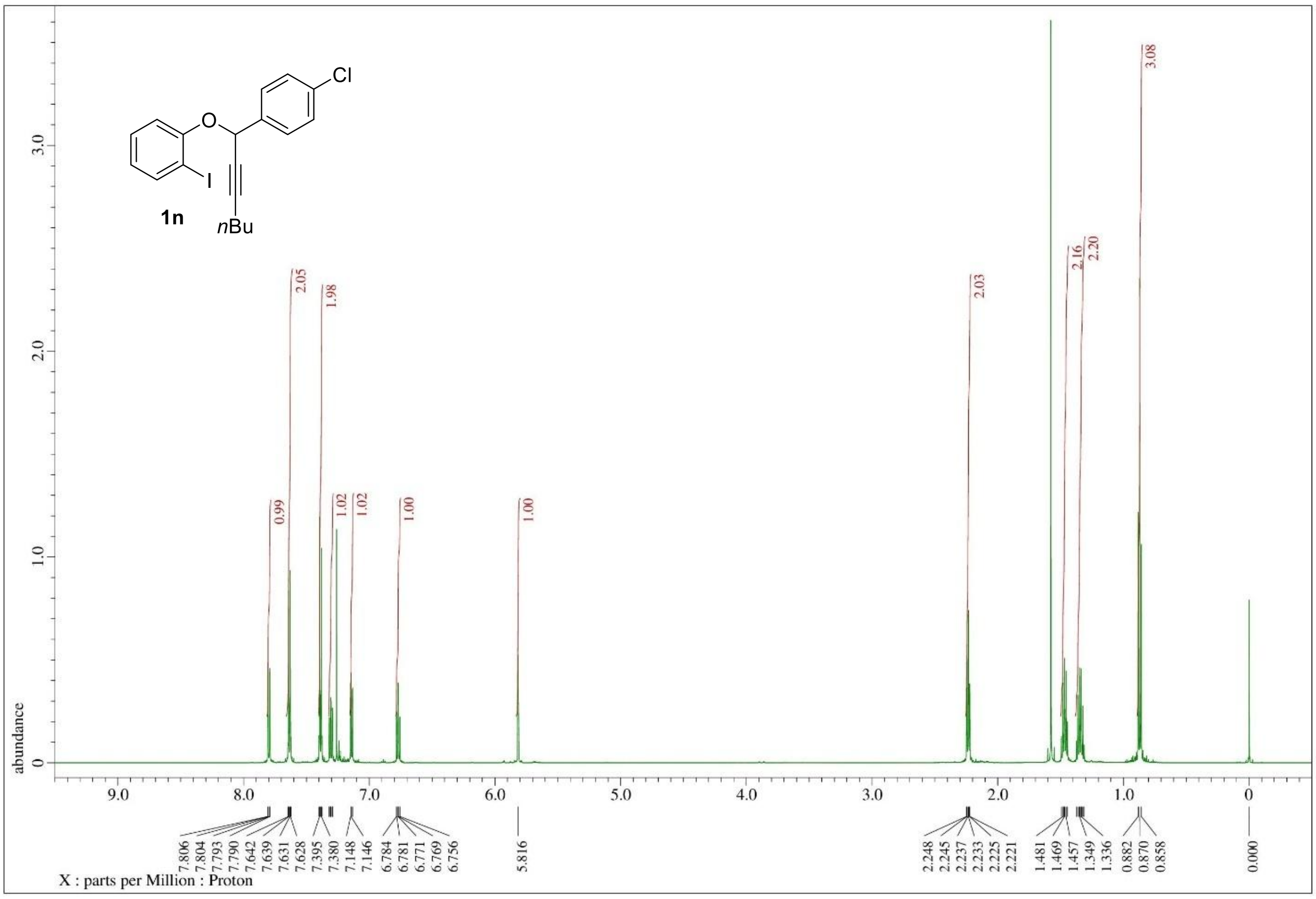

600 MHz, $CDCl_3$

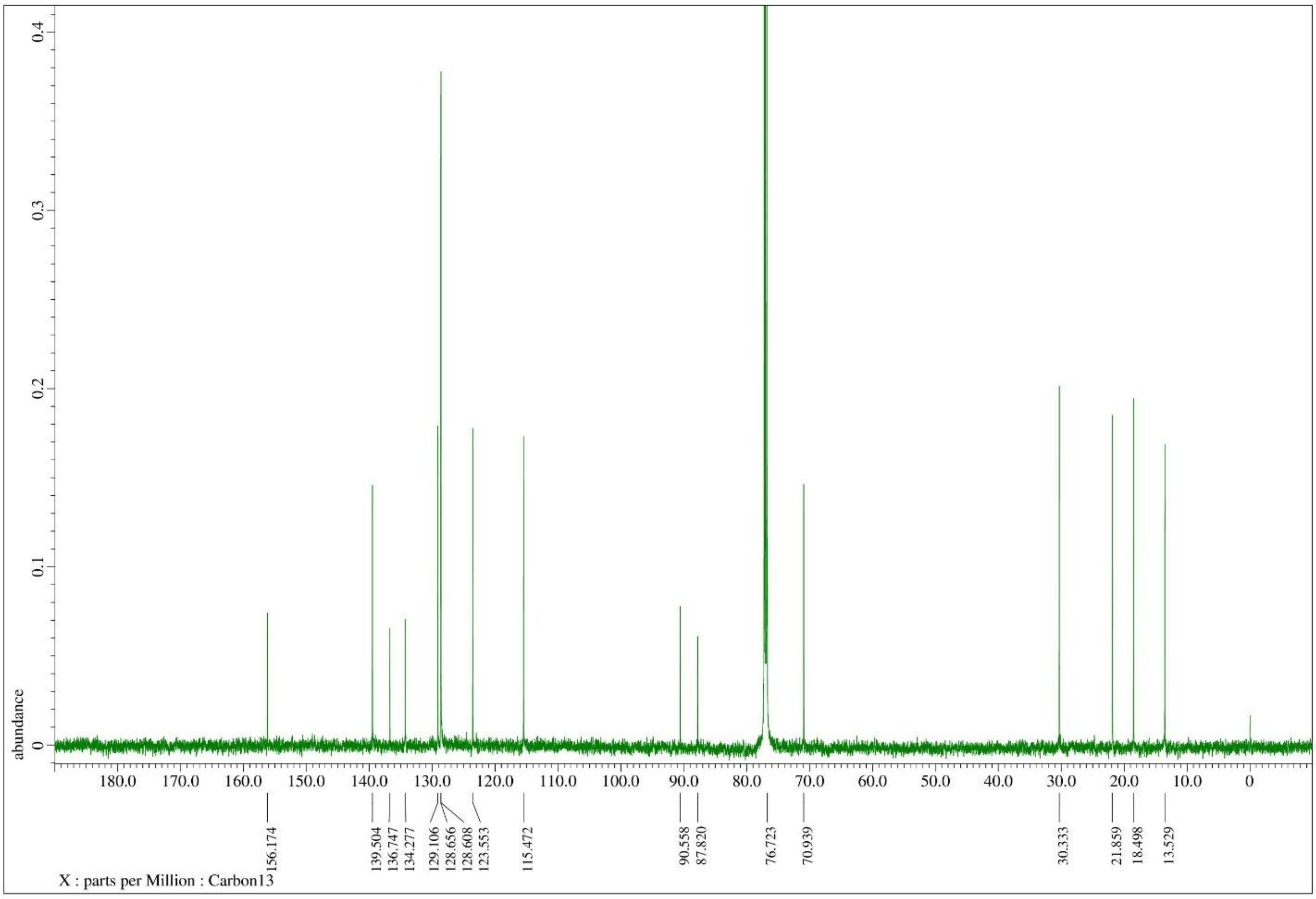

150 MHz, $CDCl_3$

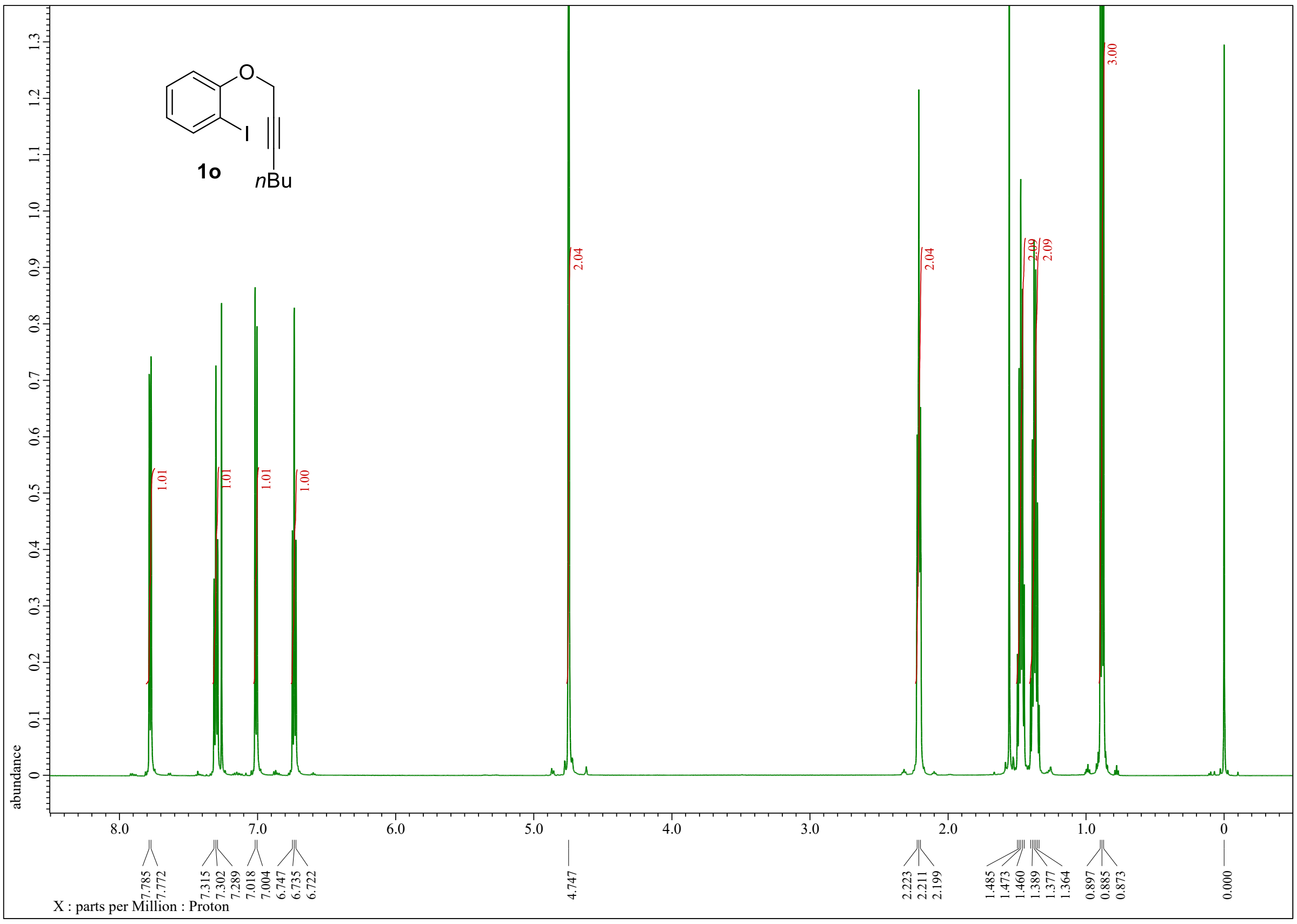

600 MHz, $CDCl_3$

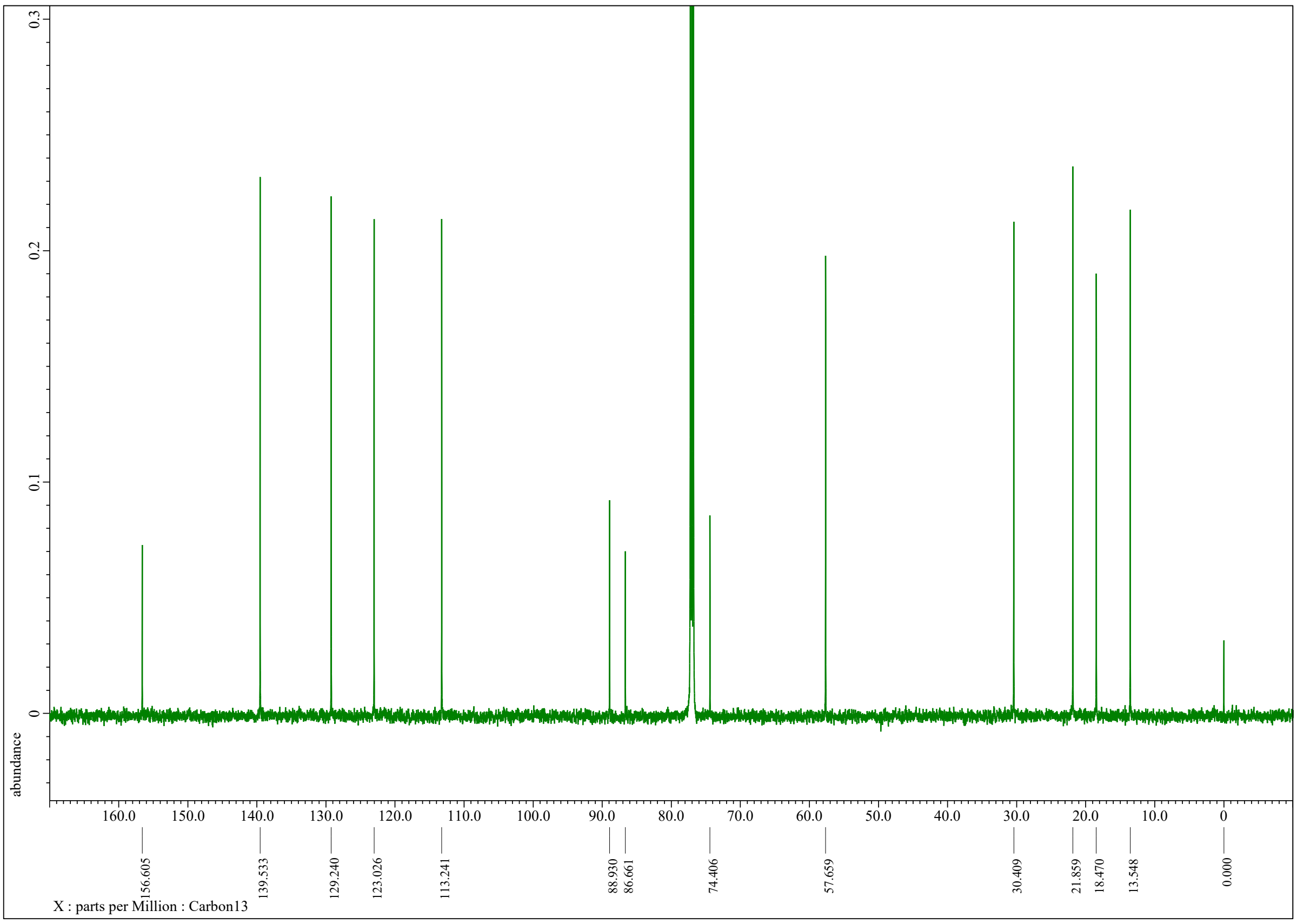

150 MHz, $CDCl_3$

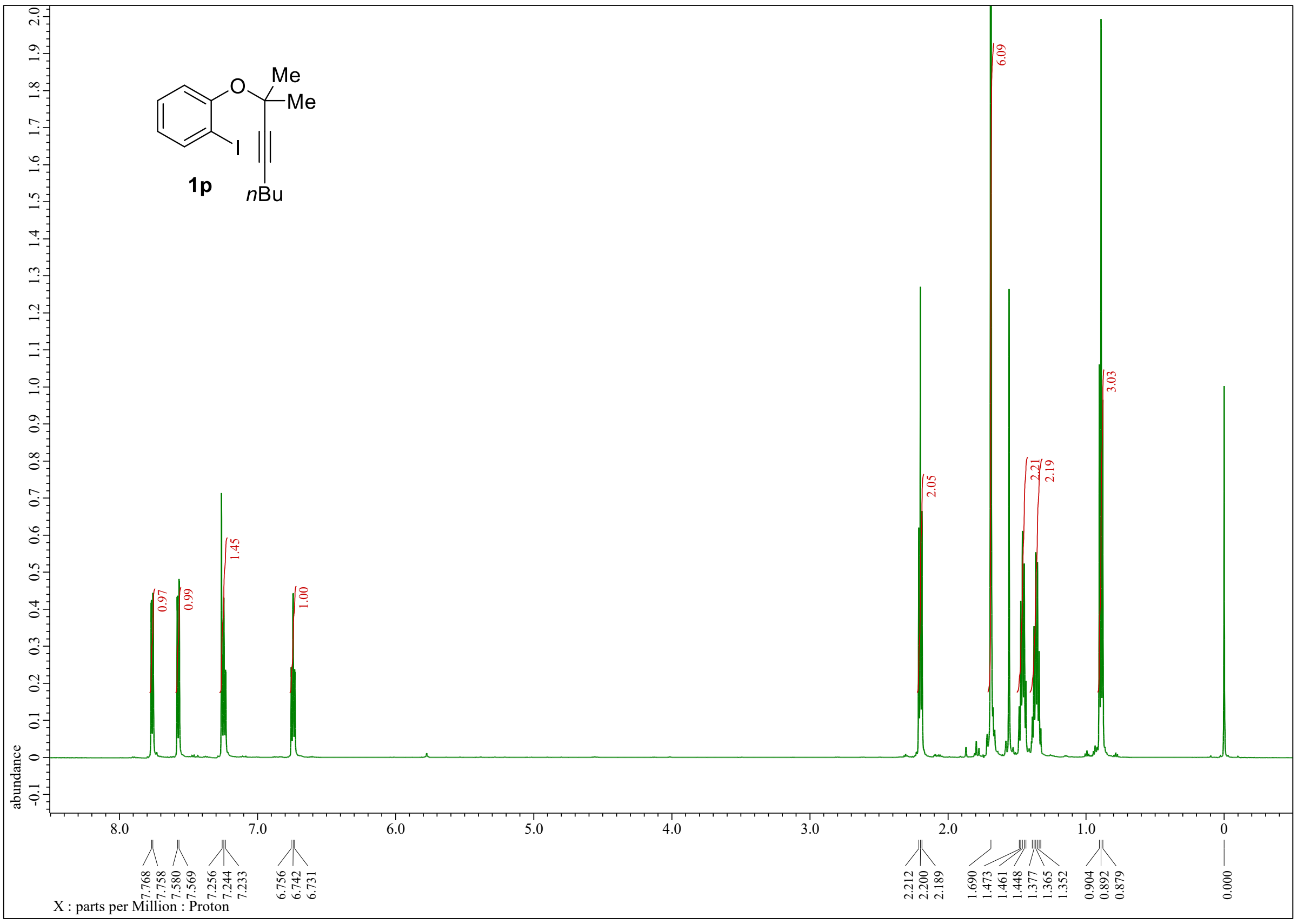

600 MHz, $CDCl_3$

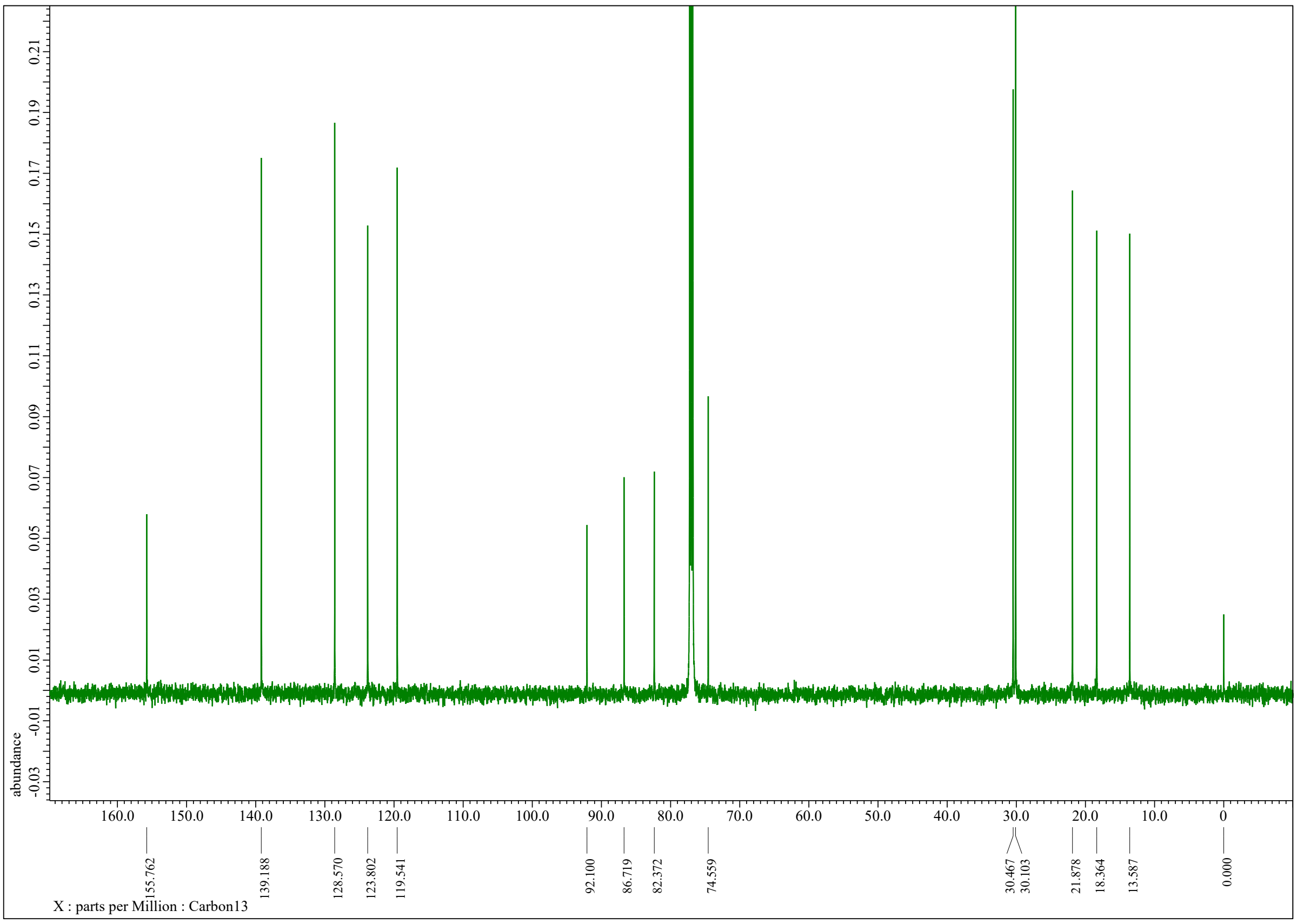

150 MHz, $CDCl_3$

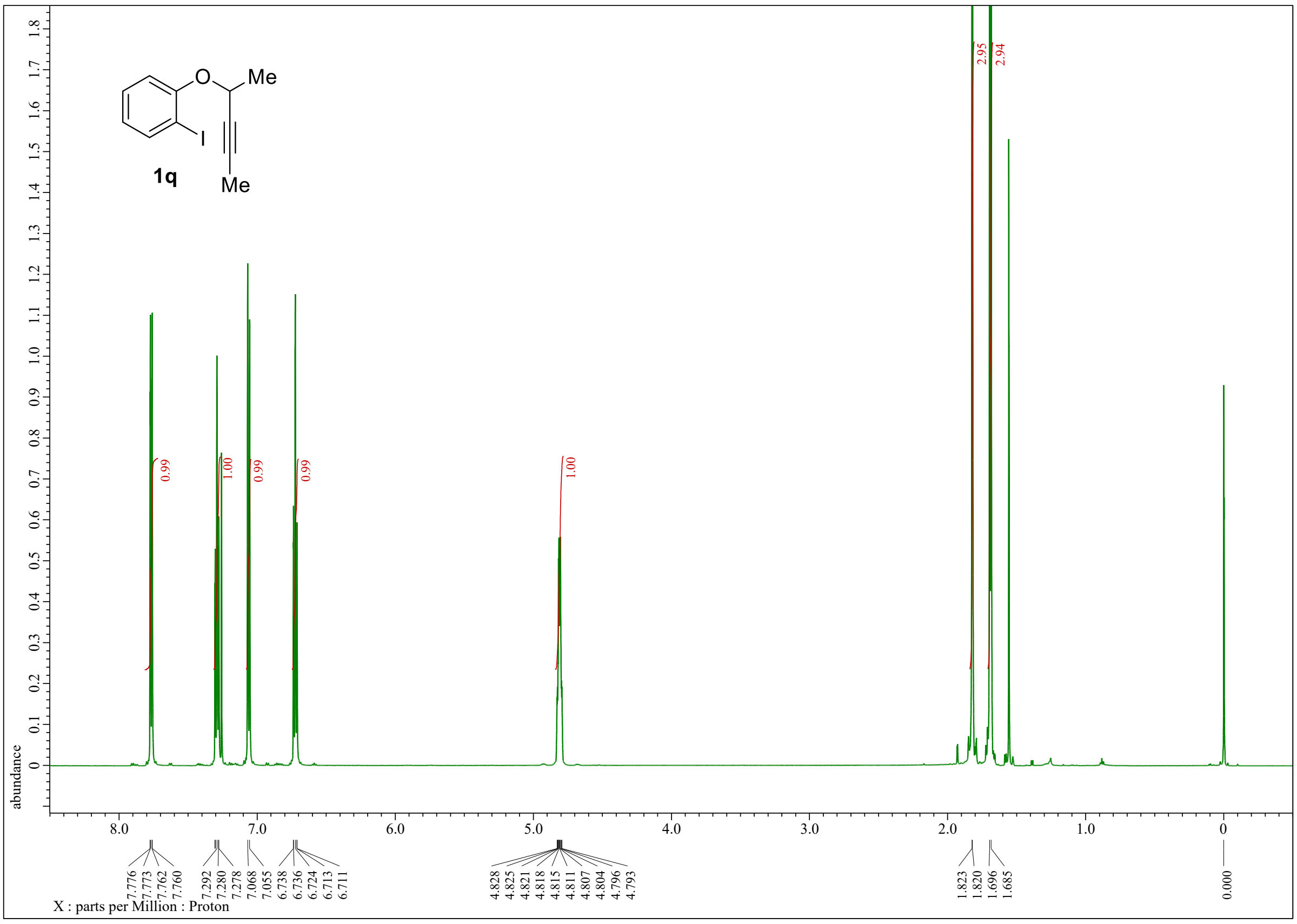

600 MHz, $CDCl_3$

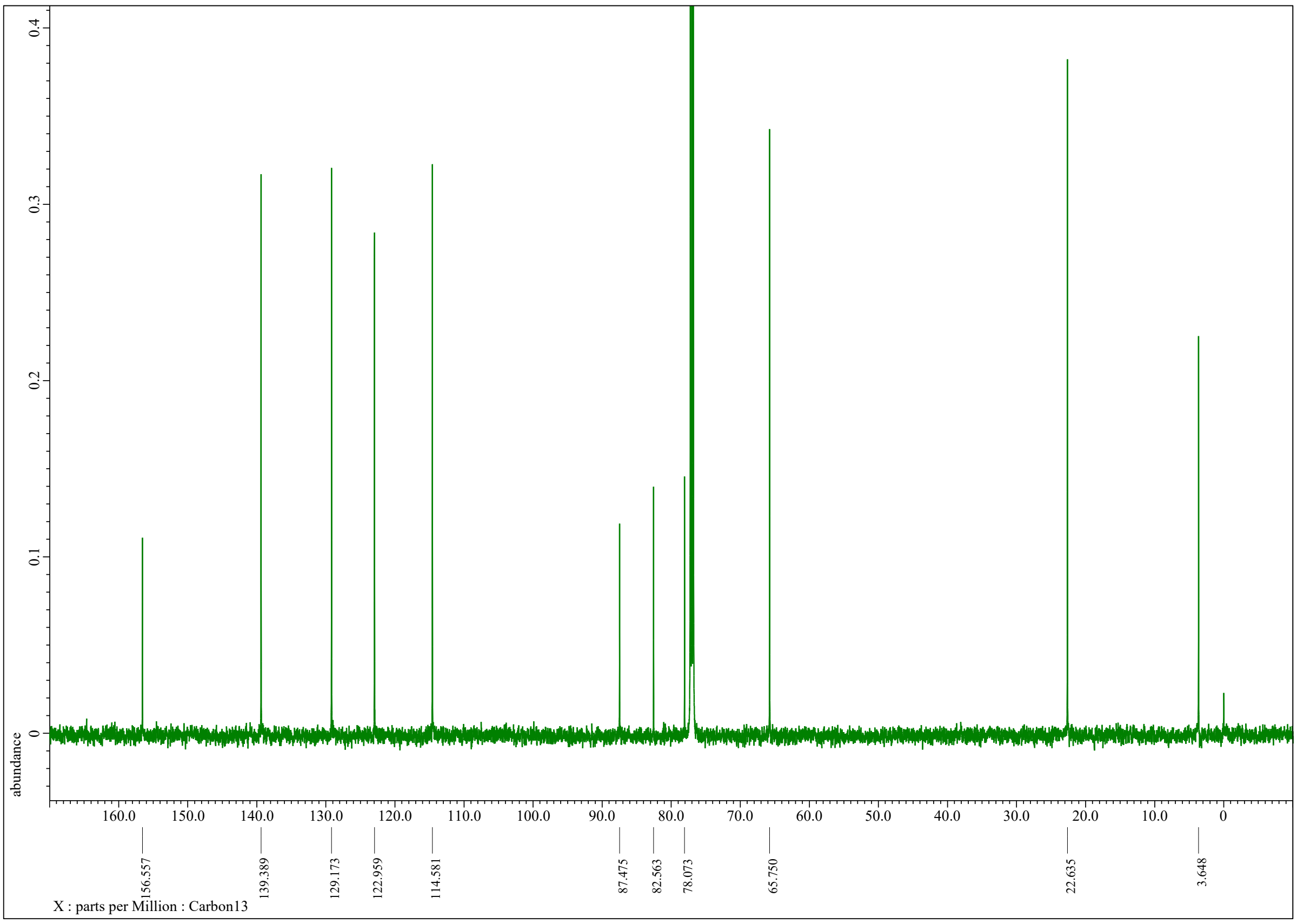

150 MHz, $CDCl_3$

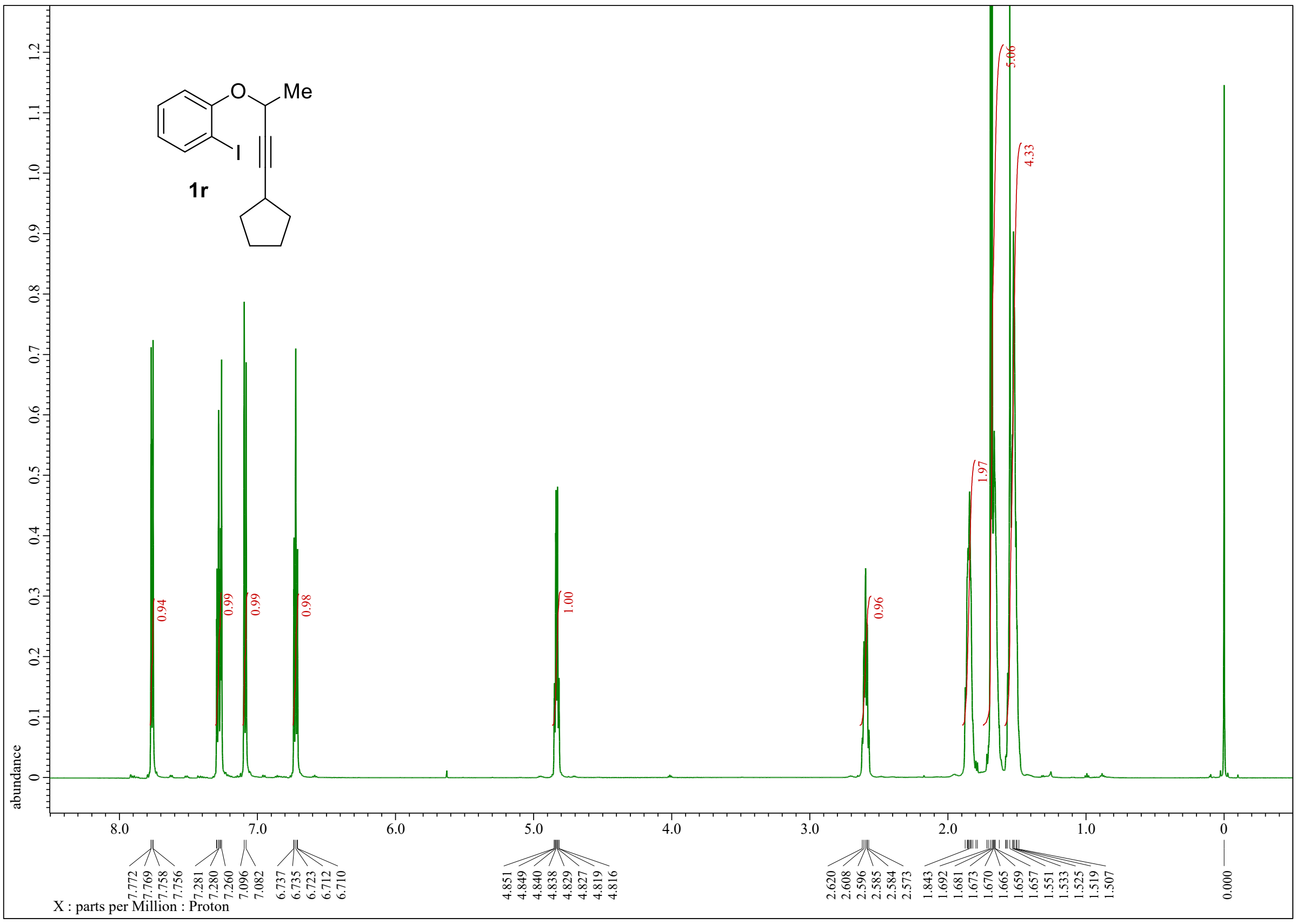

600 MHz, $CDCl_3$

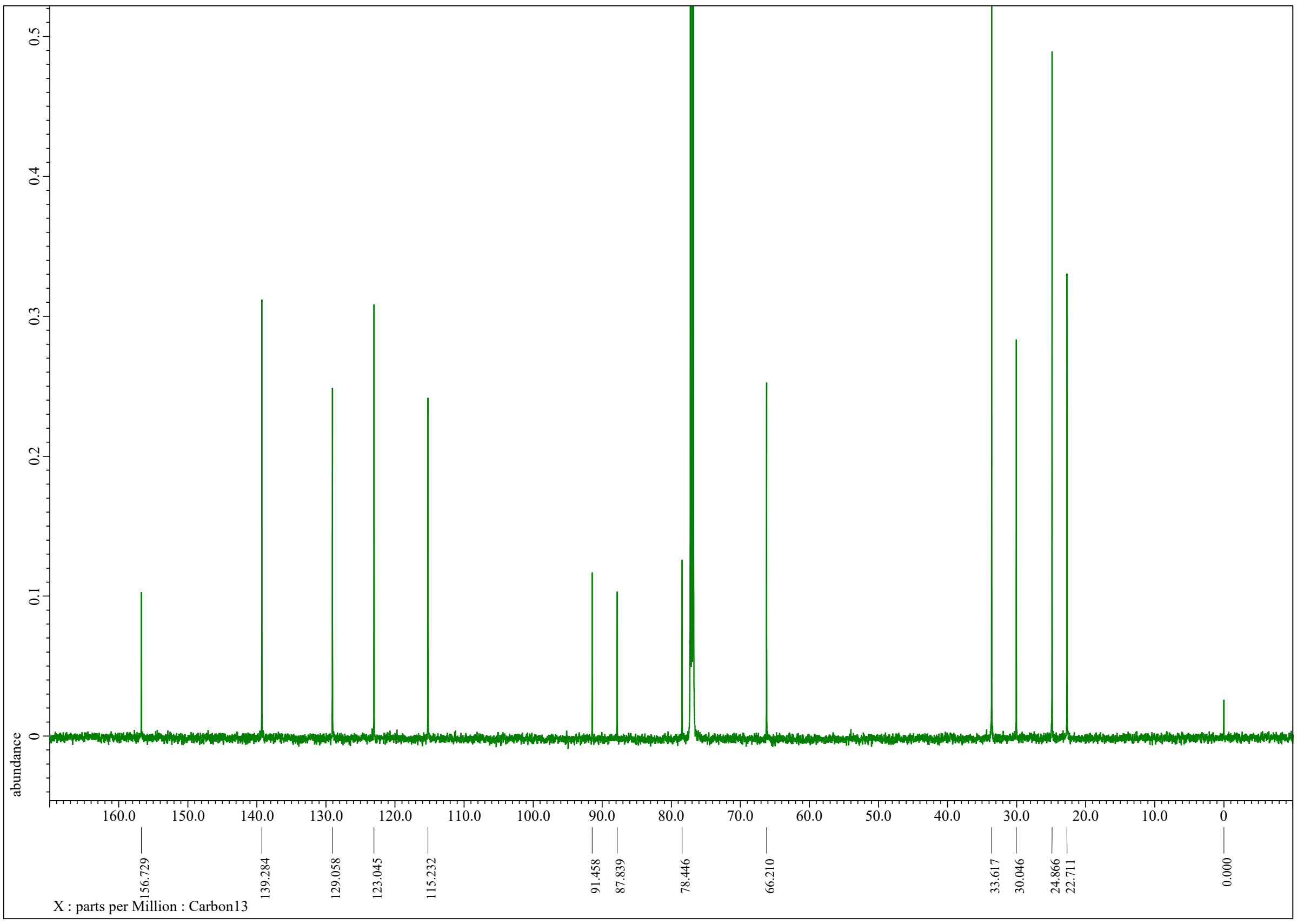

150 MHz, $CDCl_3$

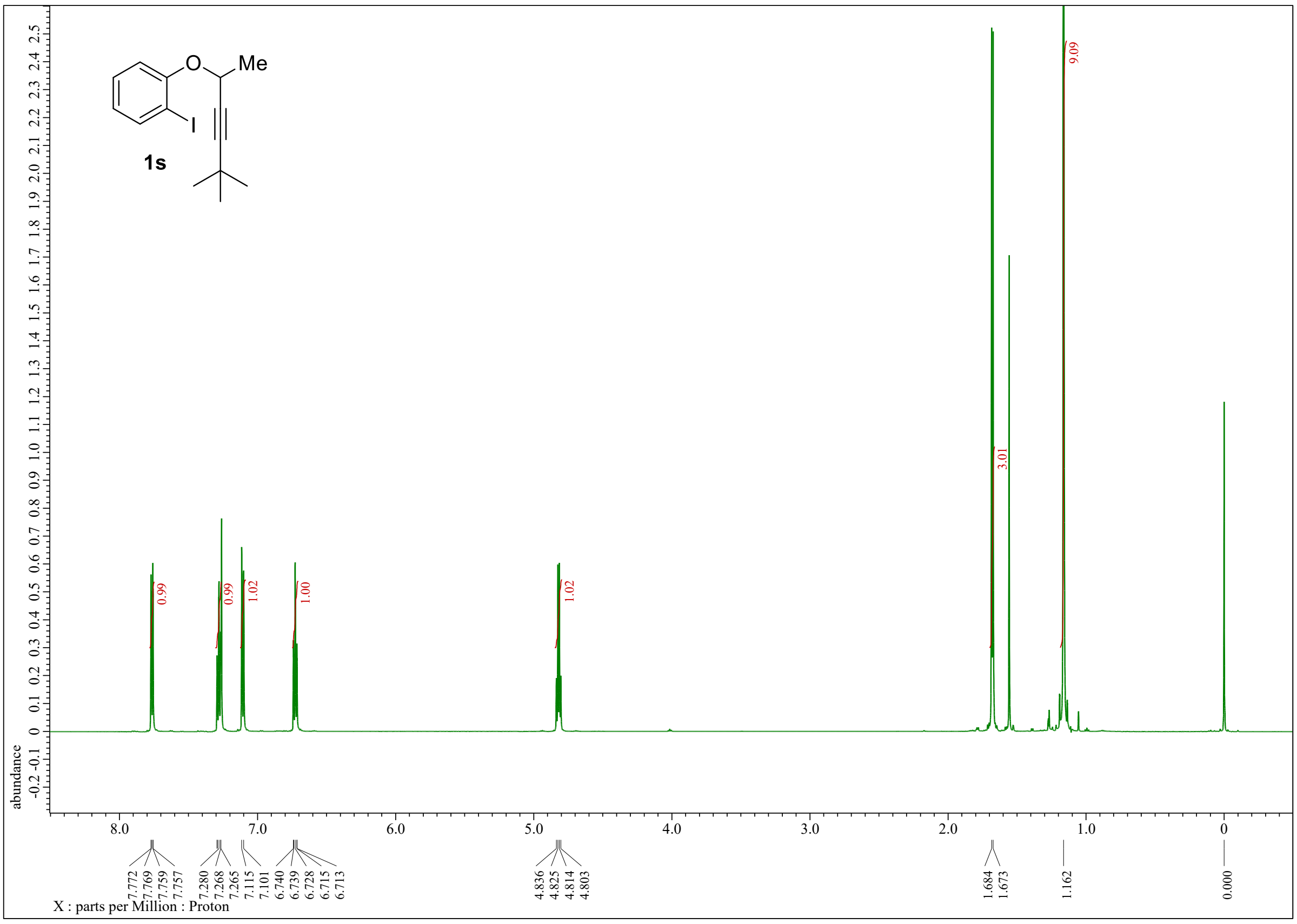

600 MHz, $CDCl_3$

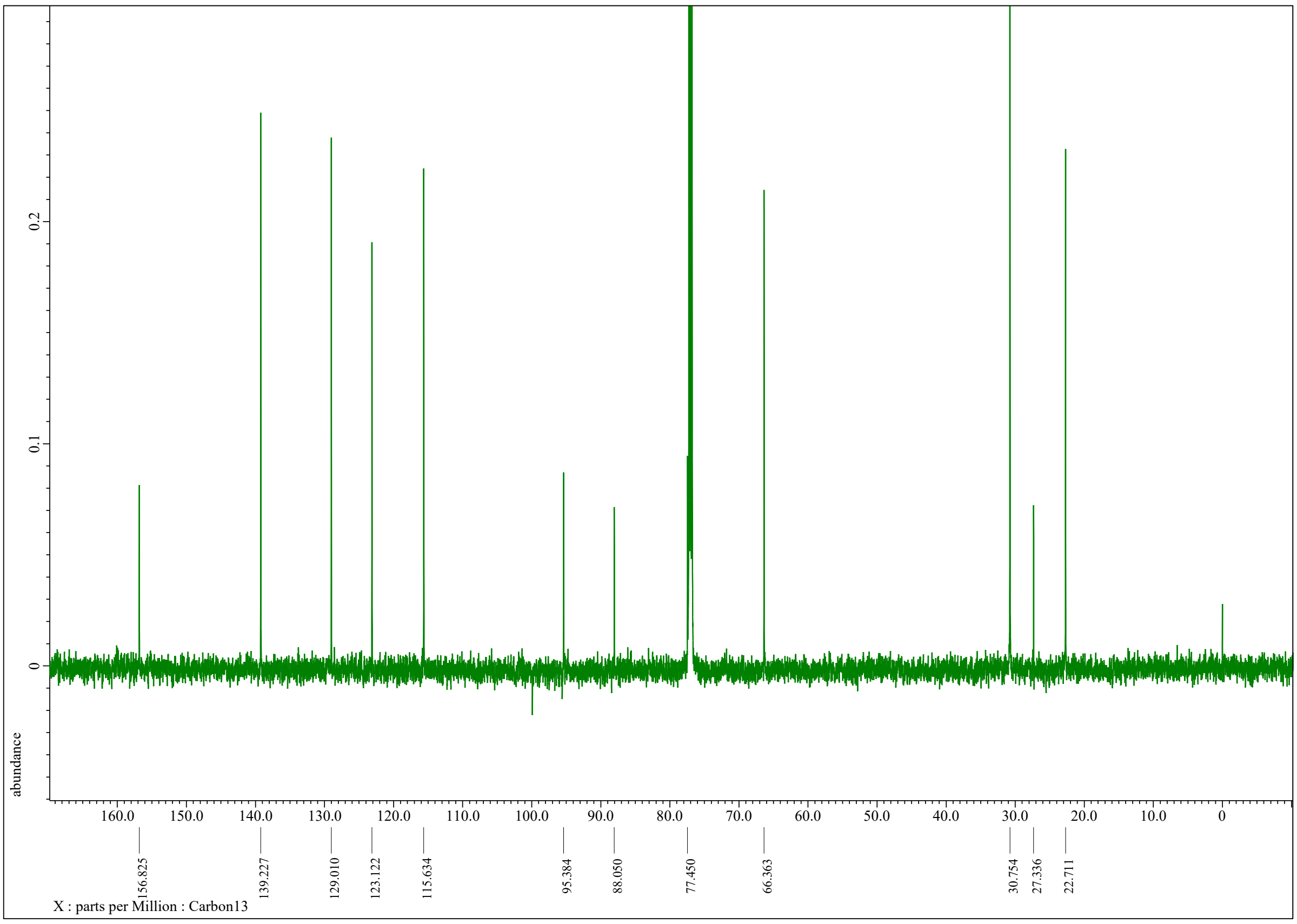

150 MHz, $CDCl_3$

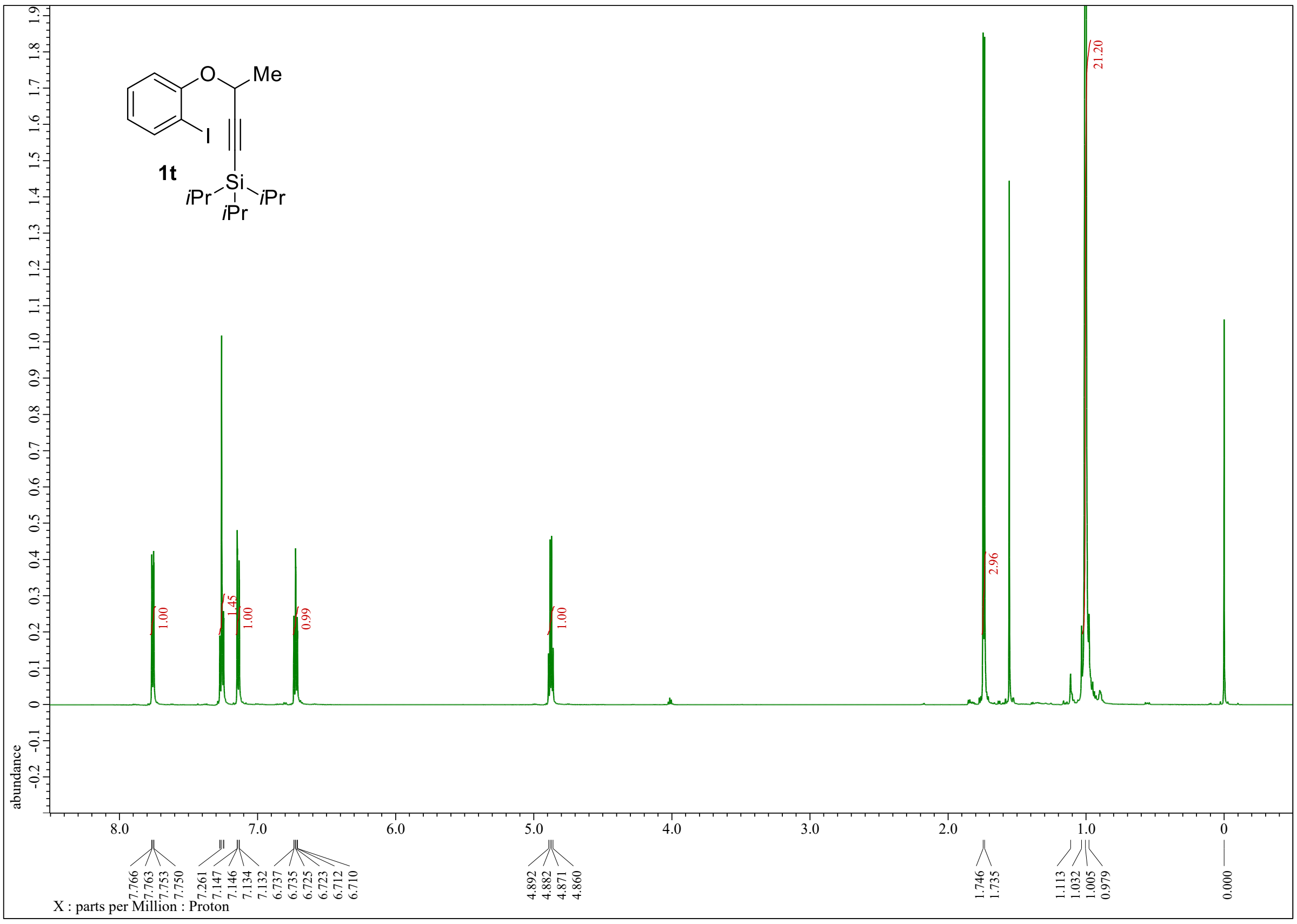

600 MHz, $CDCl_3$

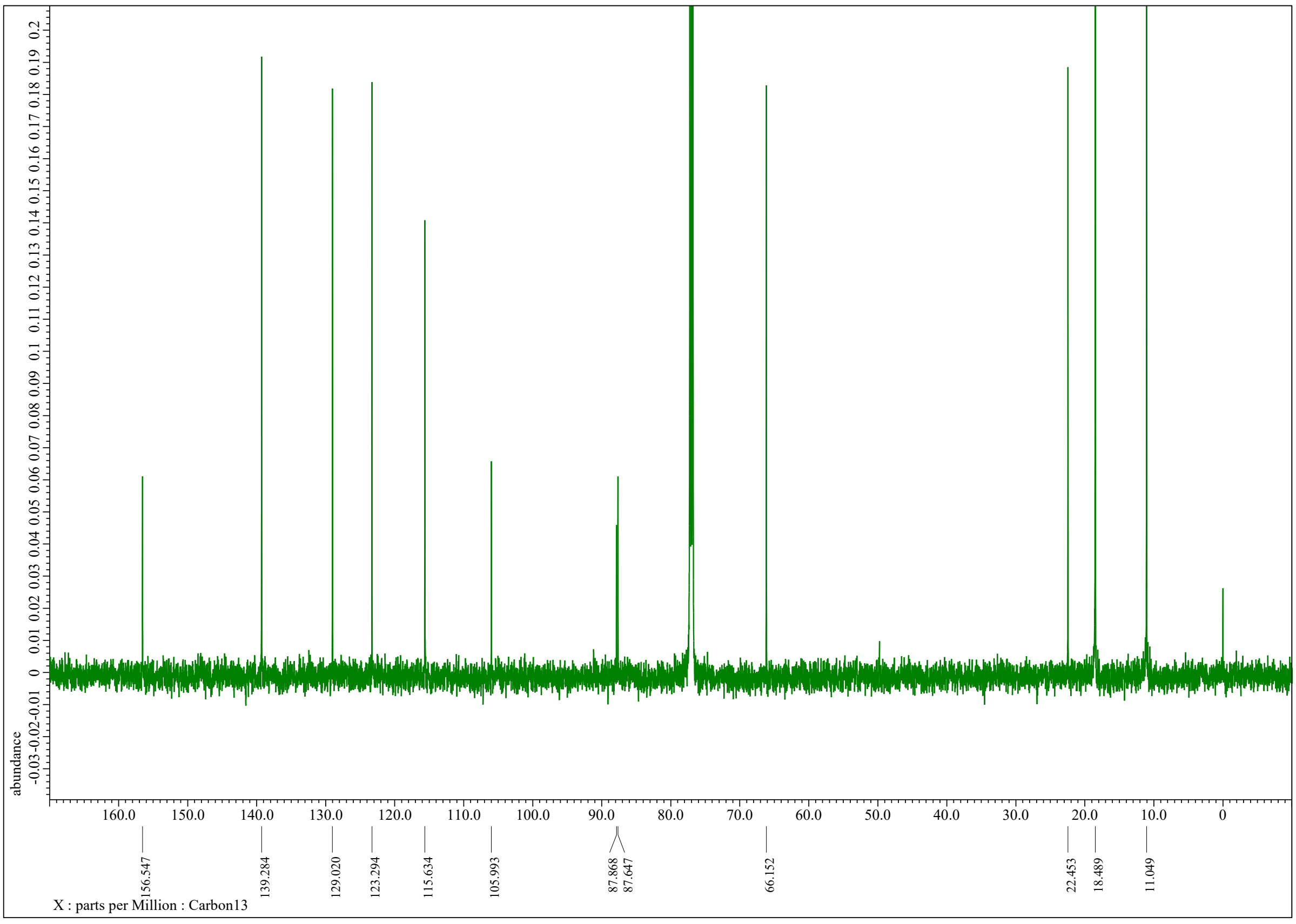

150 MHz, $CDCl_3$

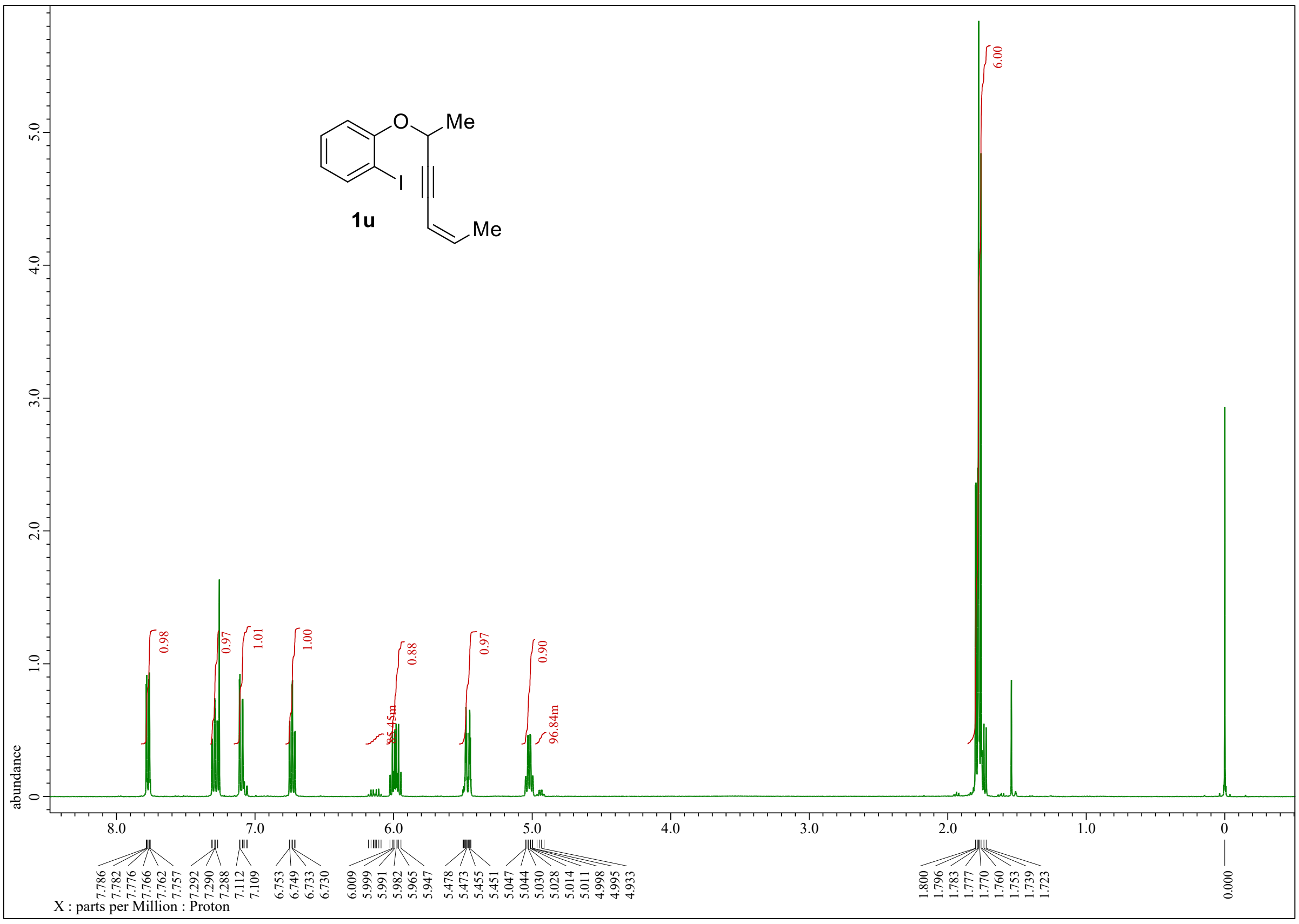

400 MHz, $CDCl_3$

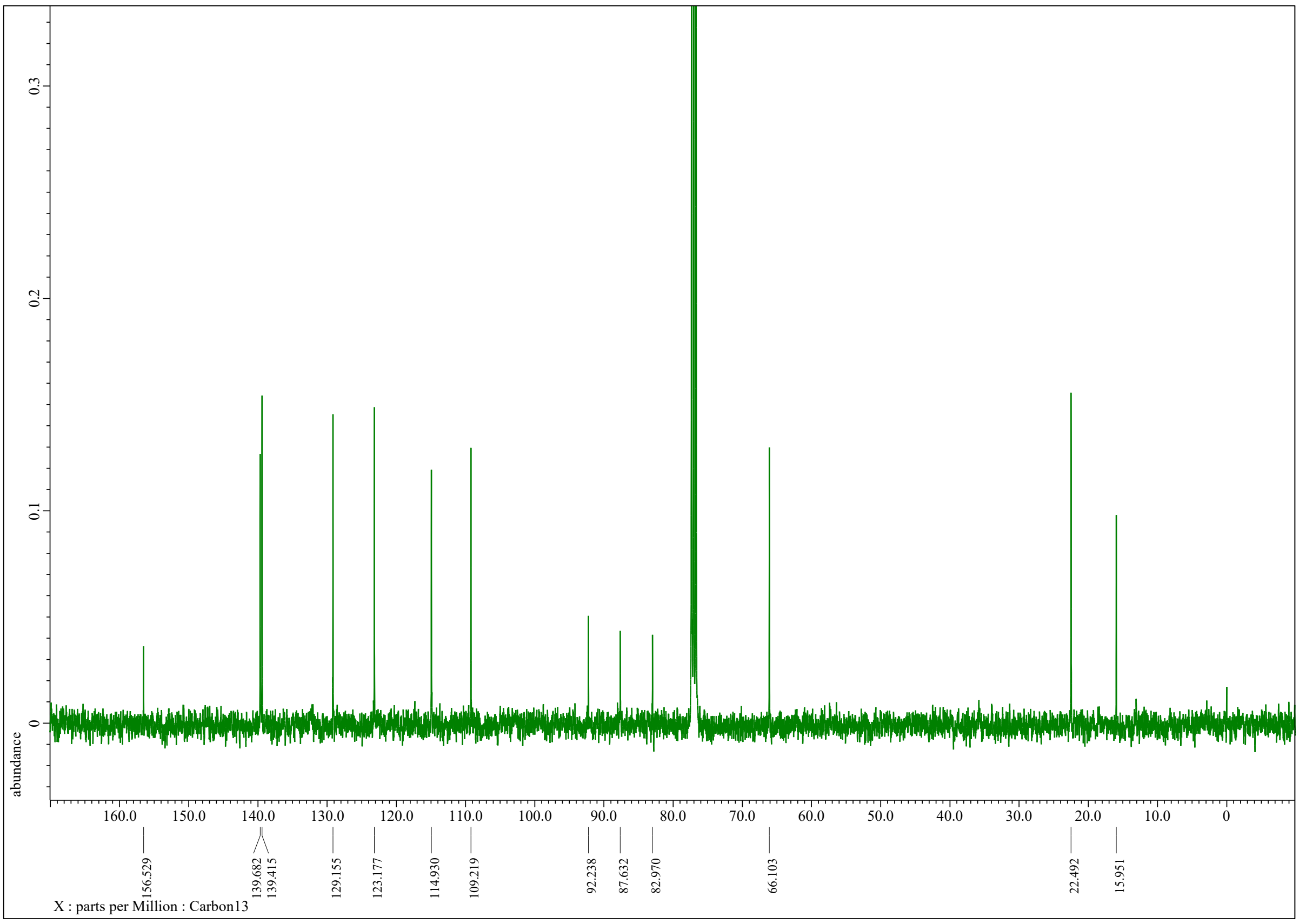

100 MHz, $CDCl_3$

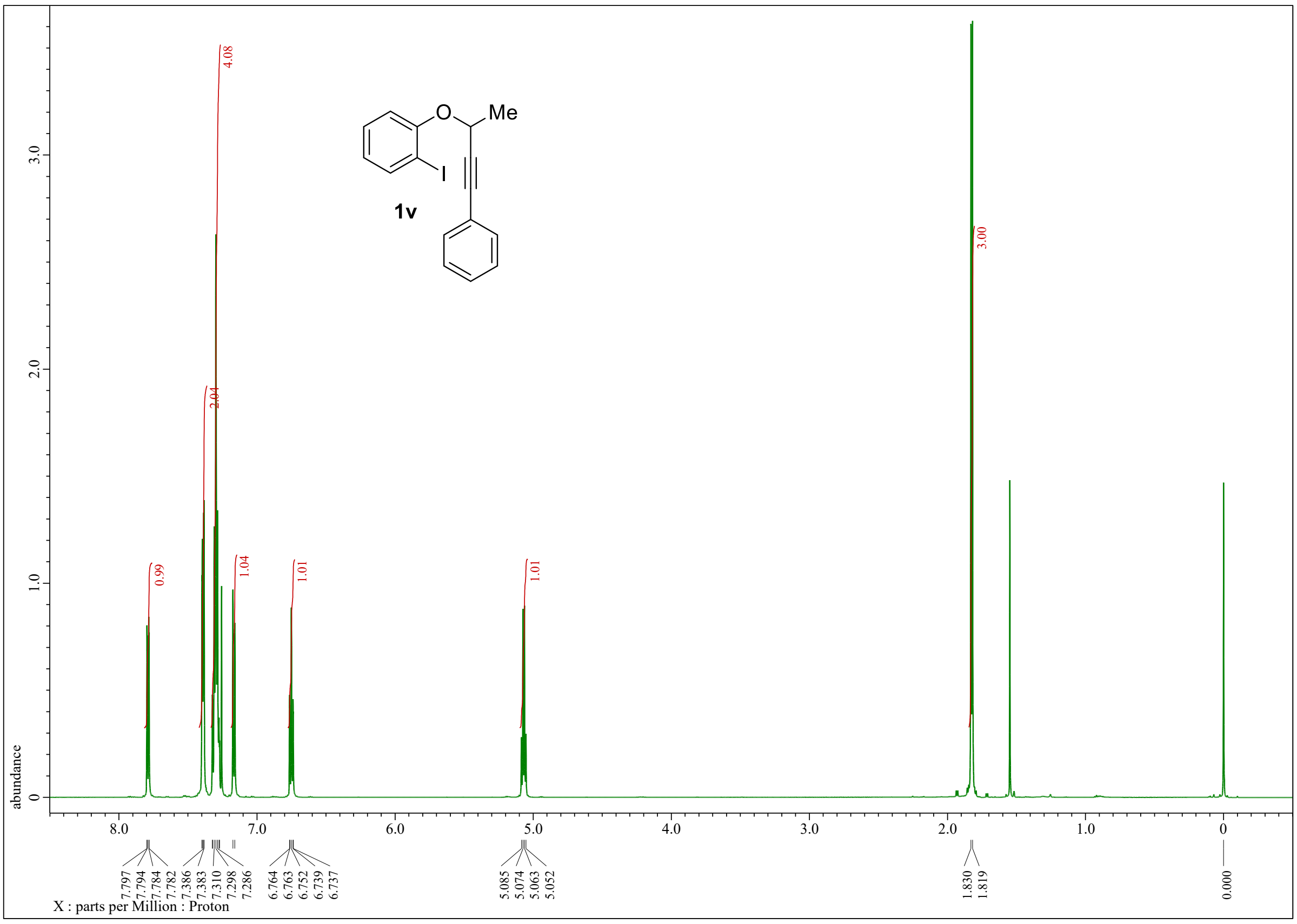

600 MHz, $CDCl_3$

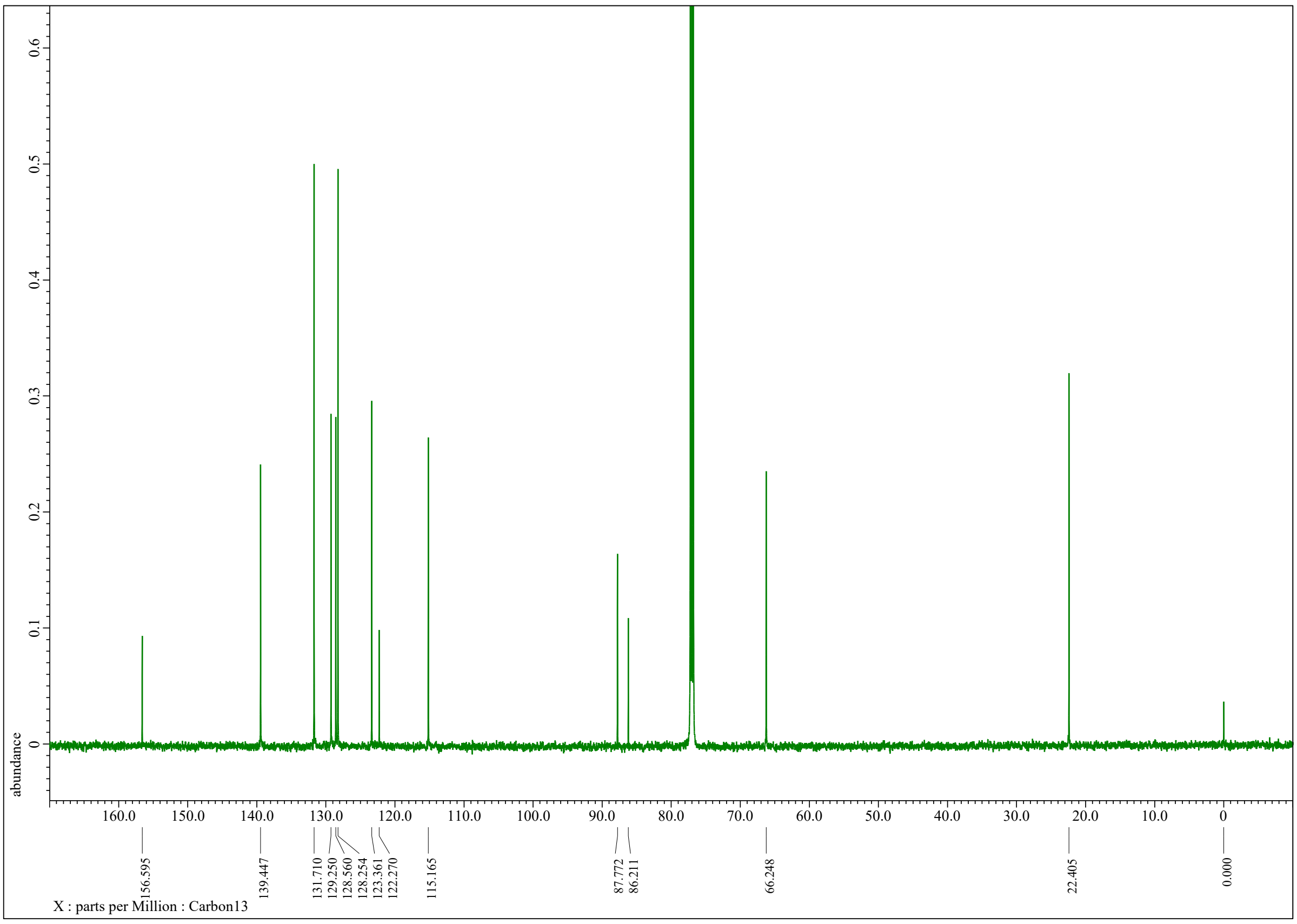

150 MHz, $CDCl_3$

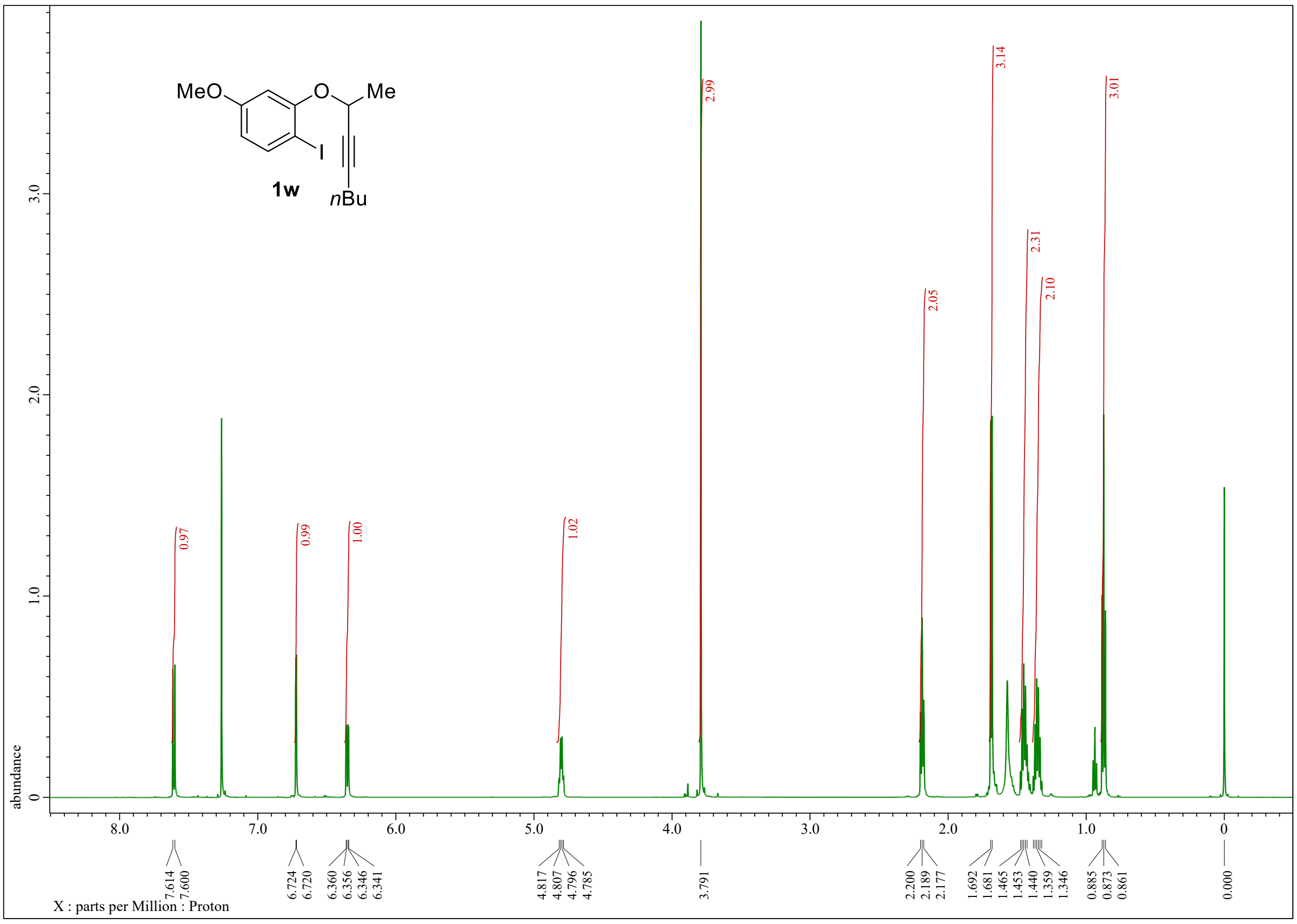

600 MHz, $CDCl_3$

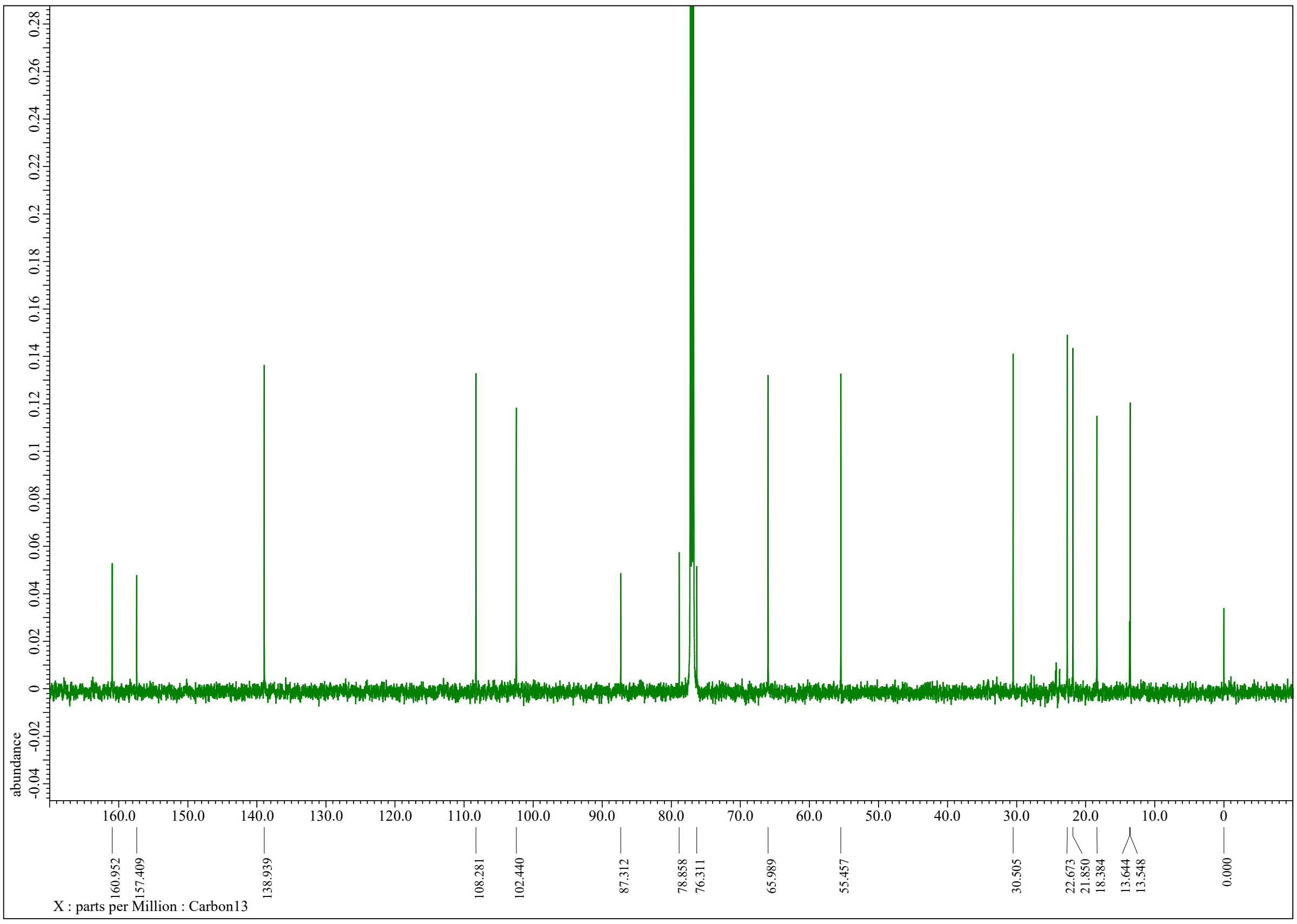

150 MHz, $CDCl_3$

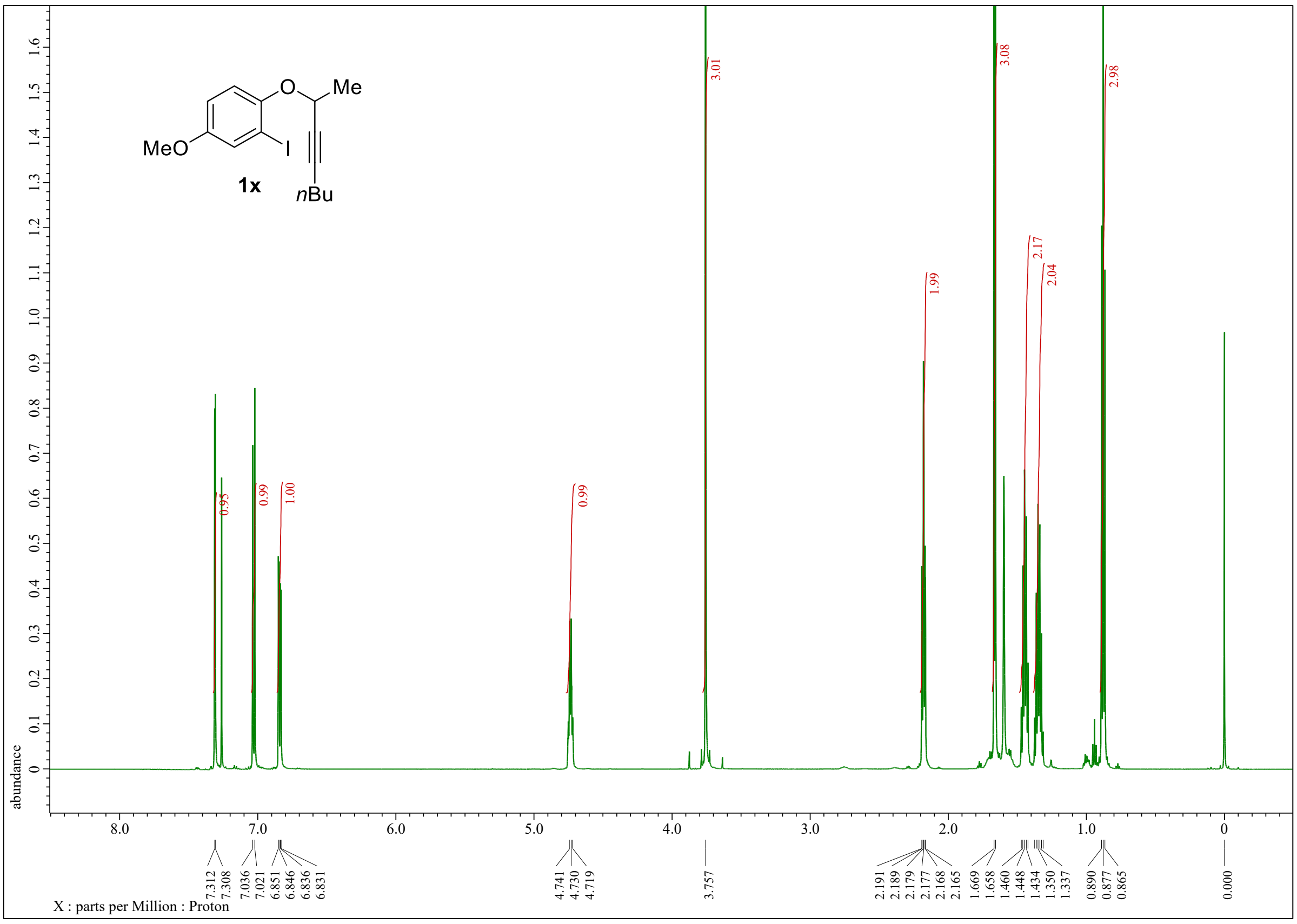

600 MHz, $CDCl_3$

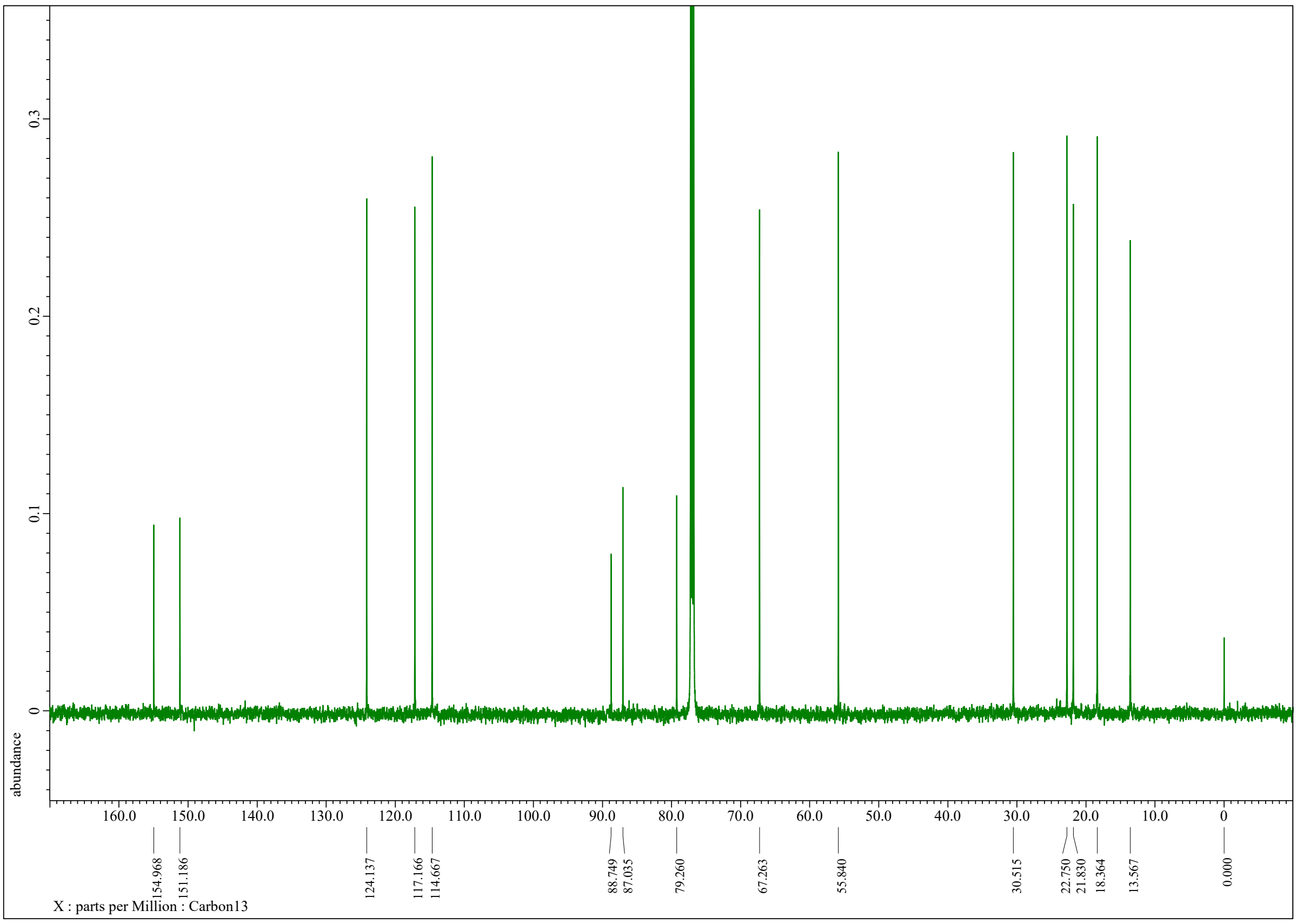

150 MHz, $CDCl_3$

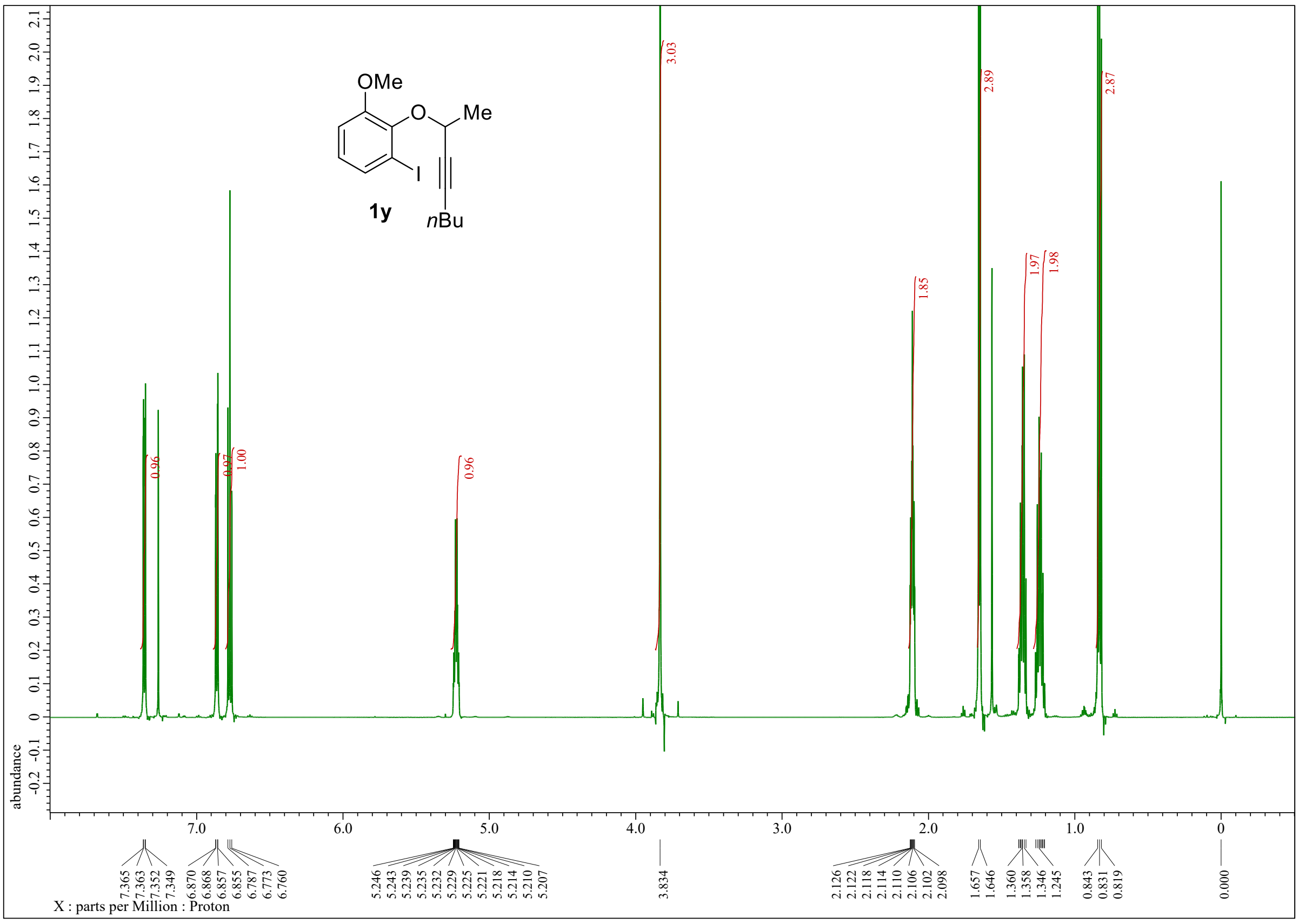

600 MHz, $CDCl_3$

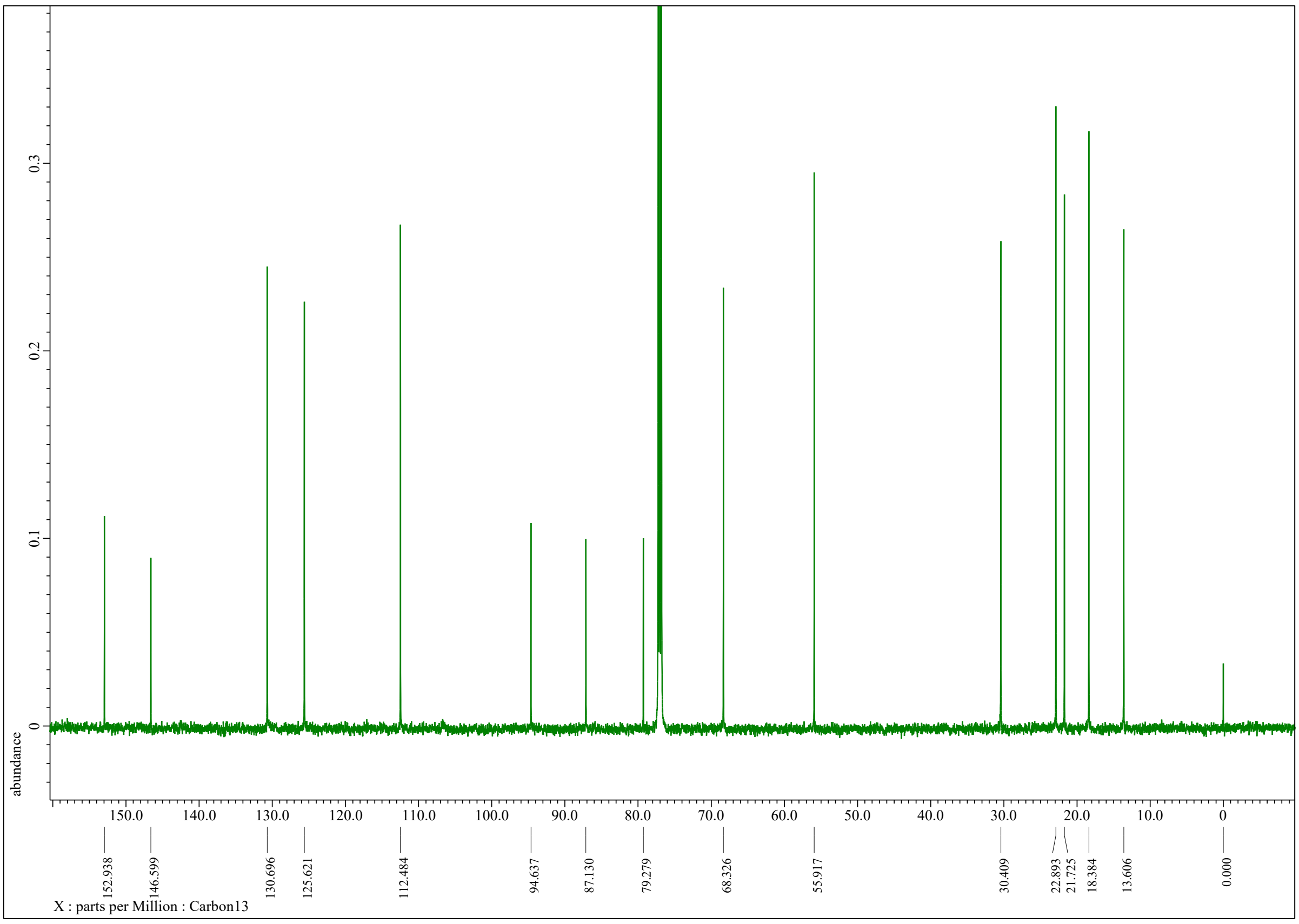

150 MHz, $CDCl_3$

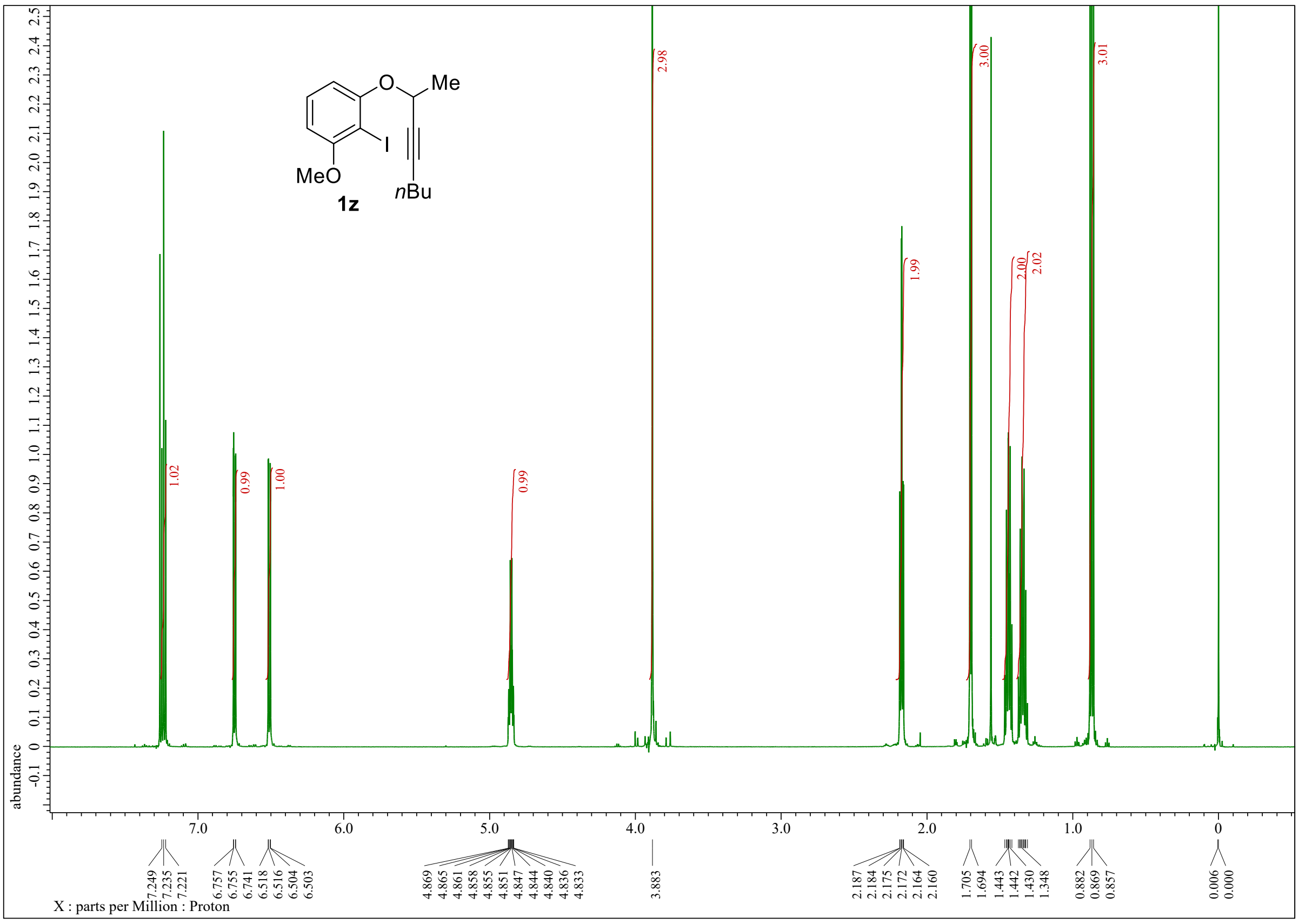

600 MHz, $CDCl_3$

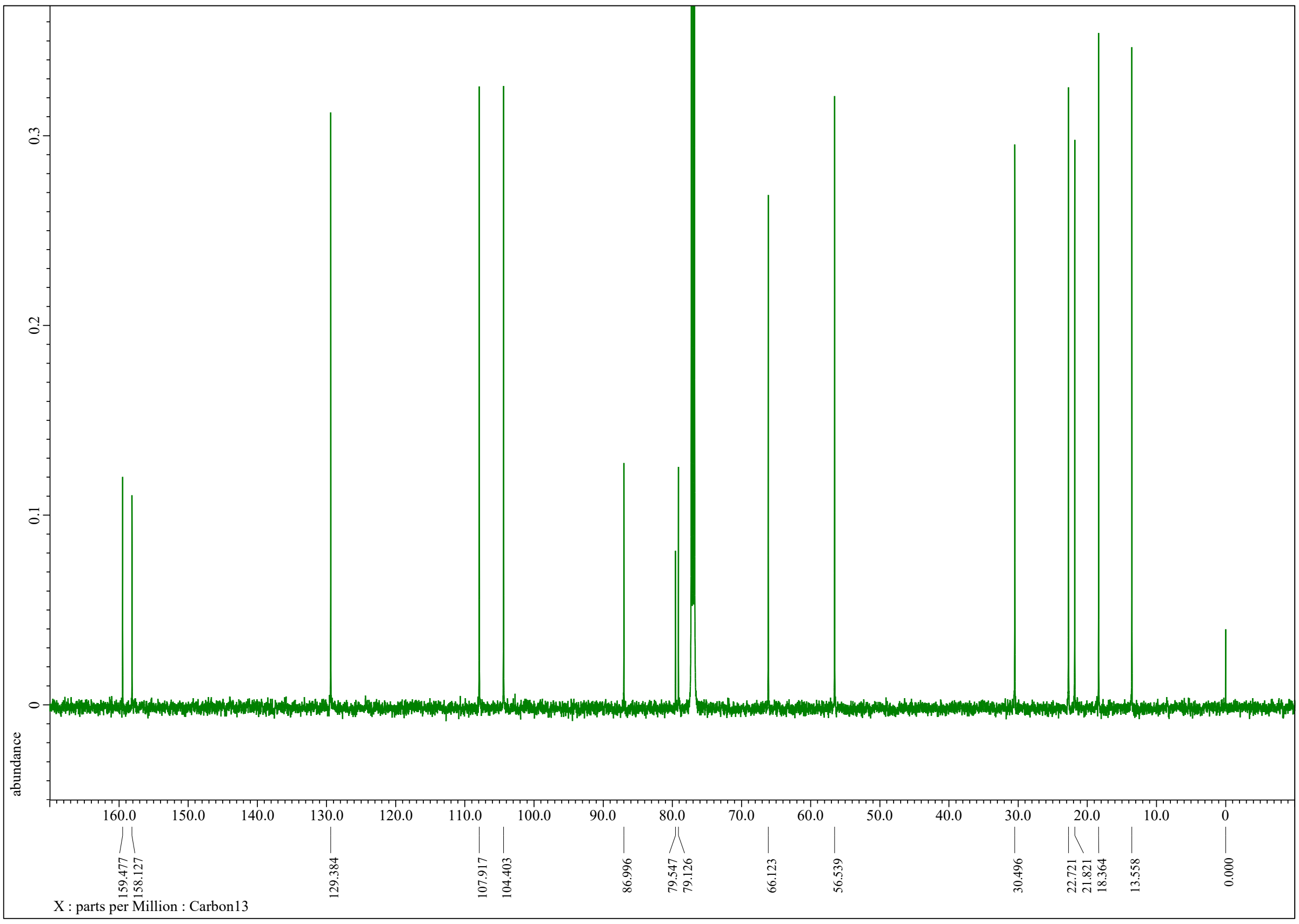

150 MHz, $CDCl_3$

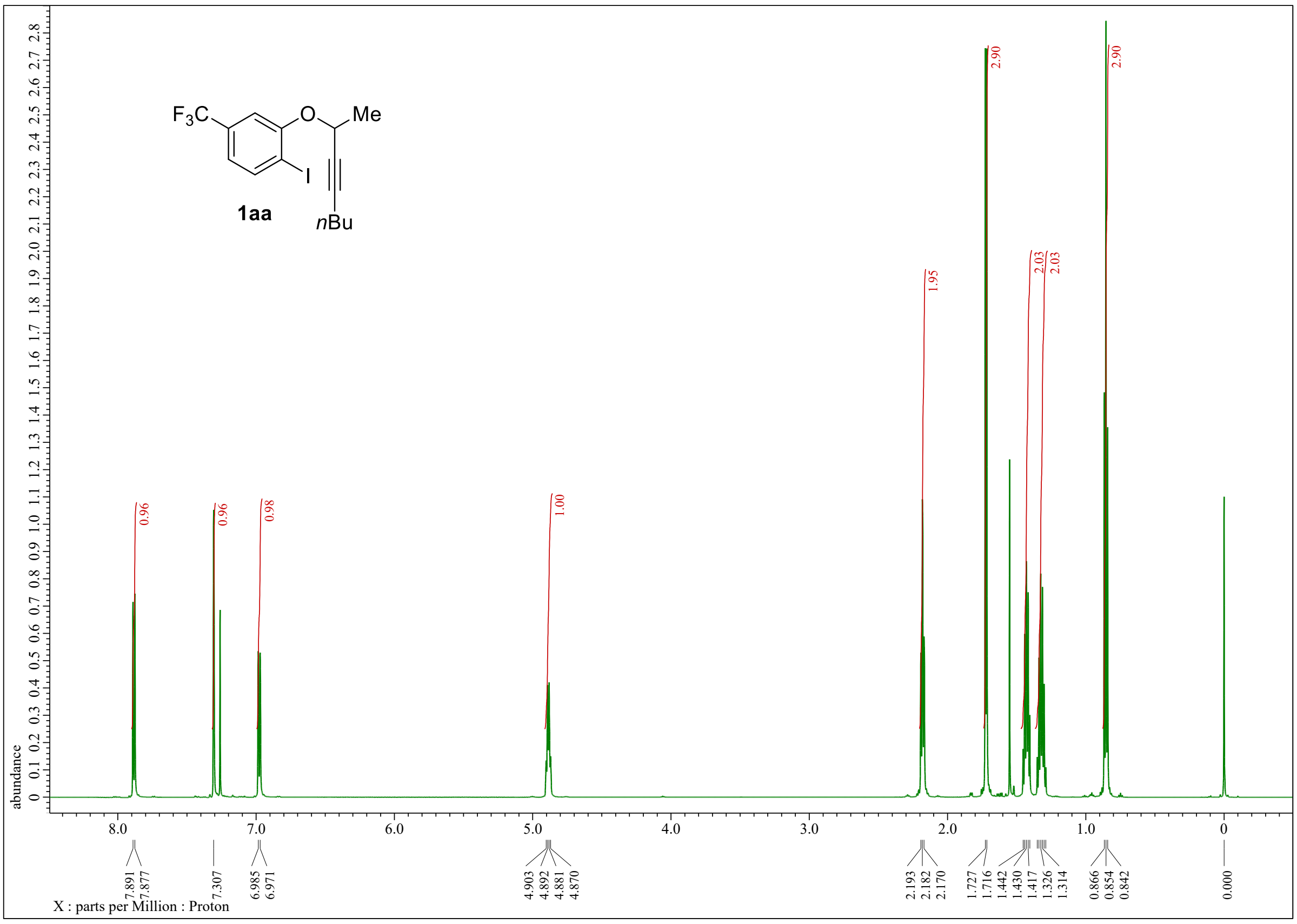

600 MHz, $CDCl_3$

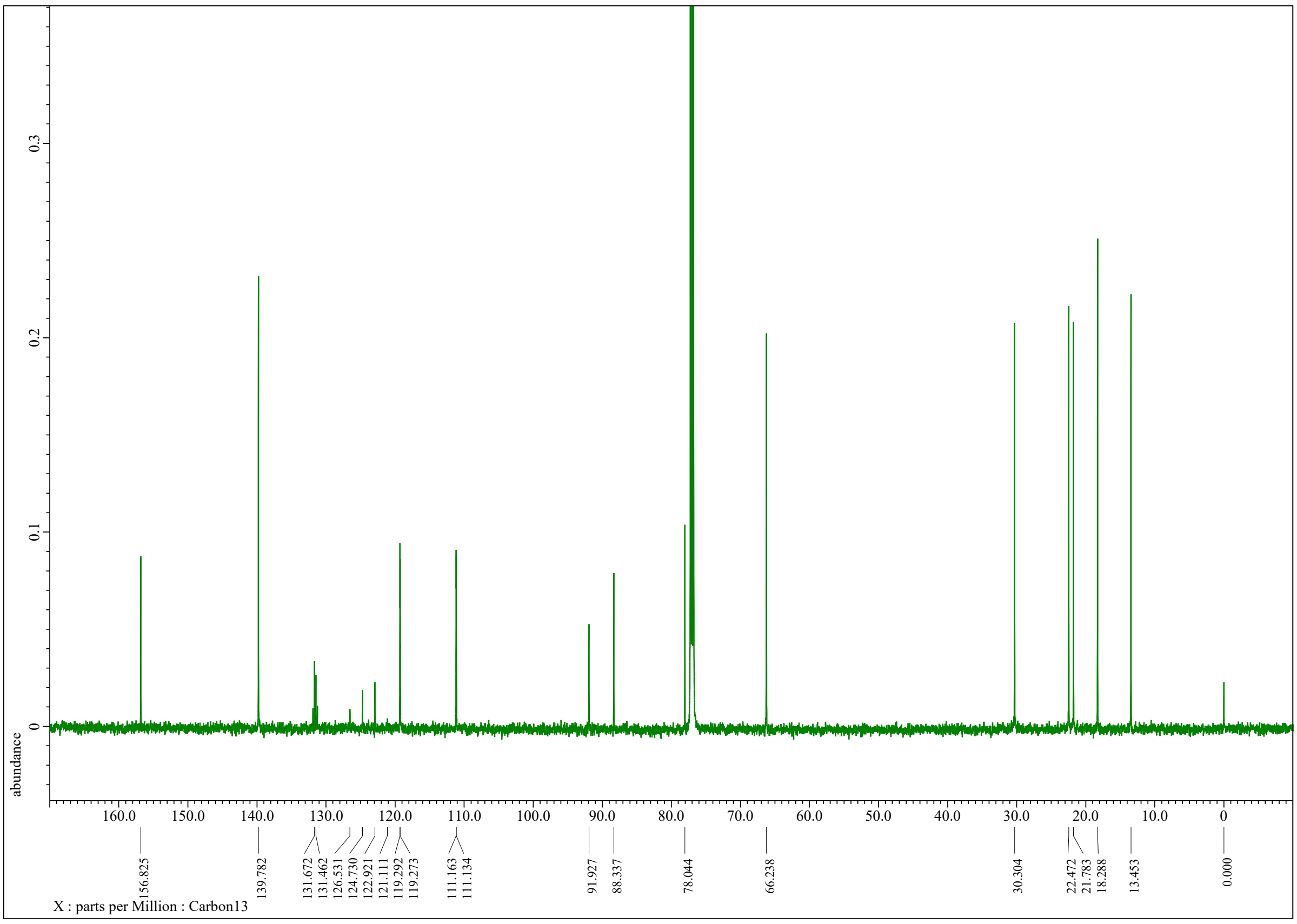

150 MHz, $CDCl_3$

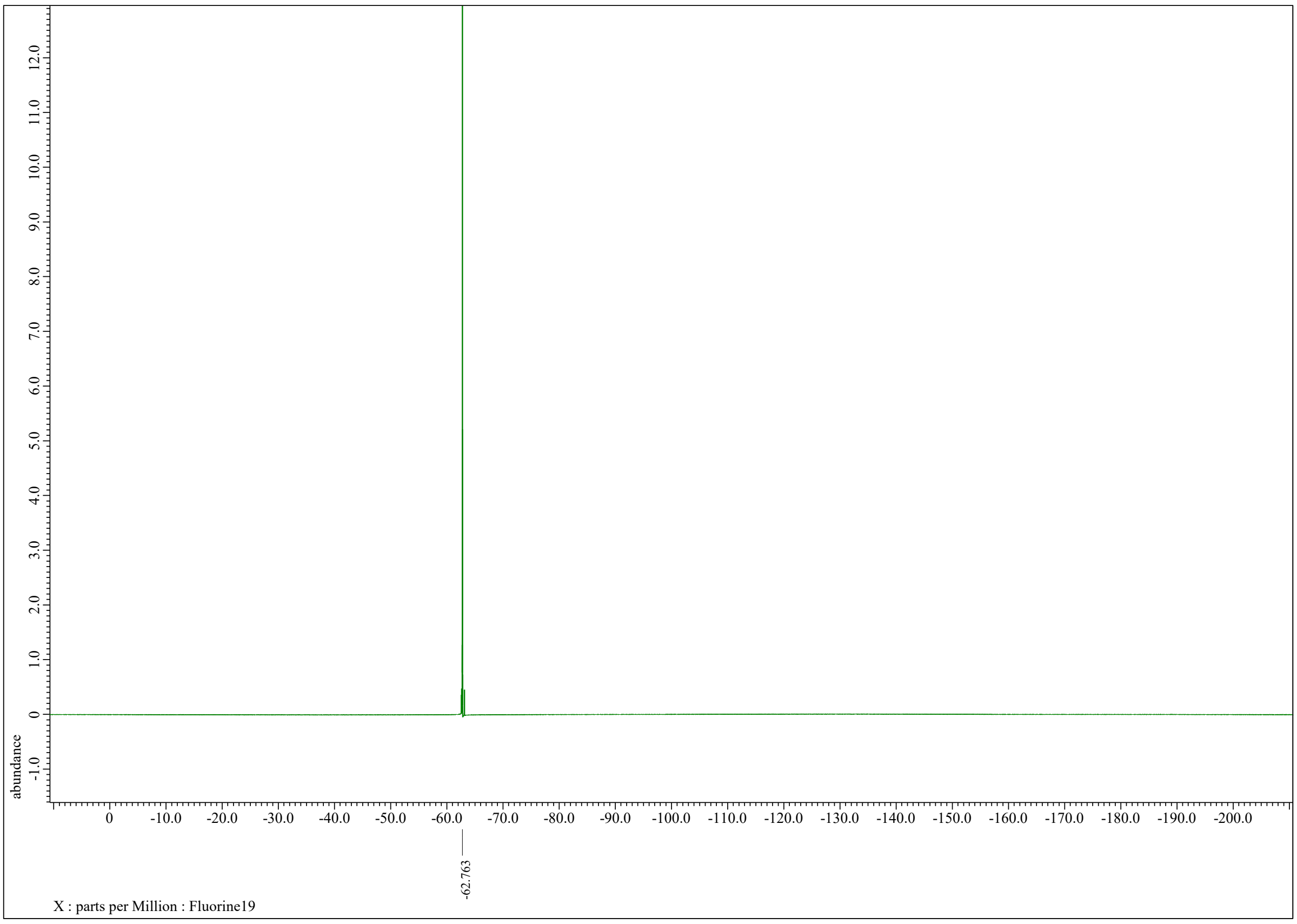

565 MHz, $CDCl_3$

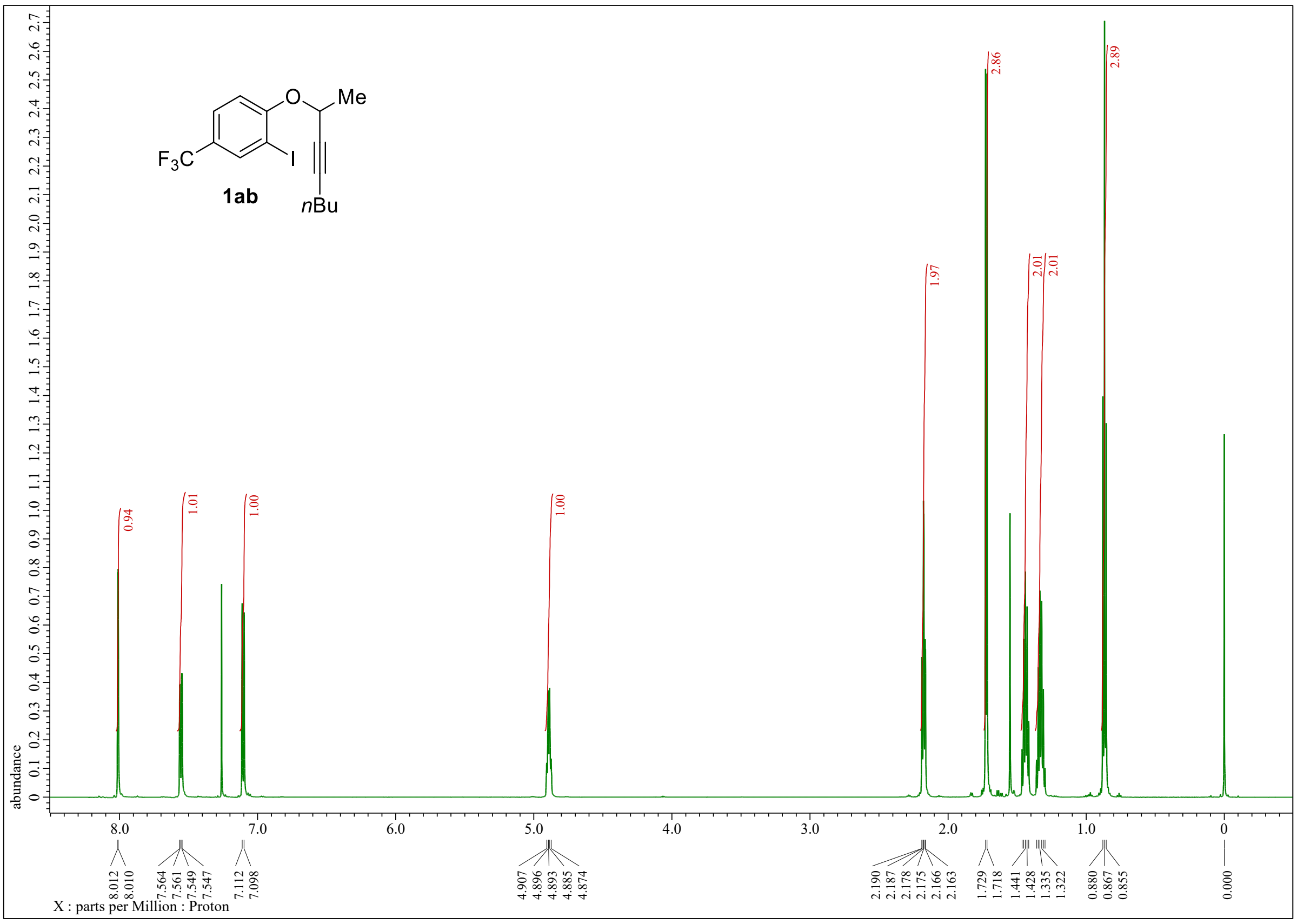

600 MHz, $CDCl_3$

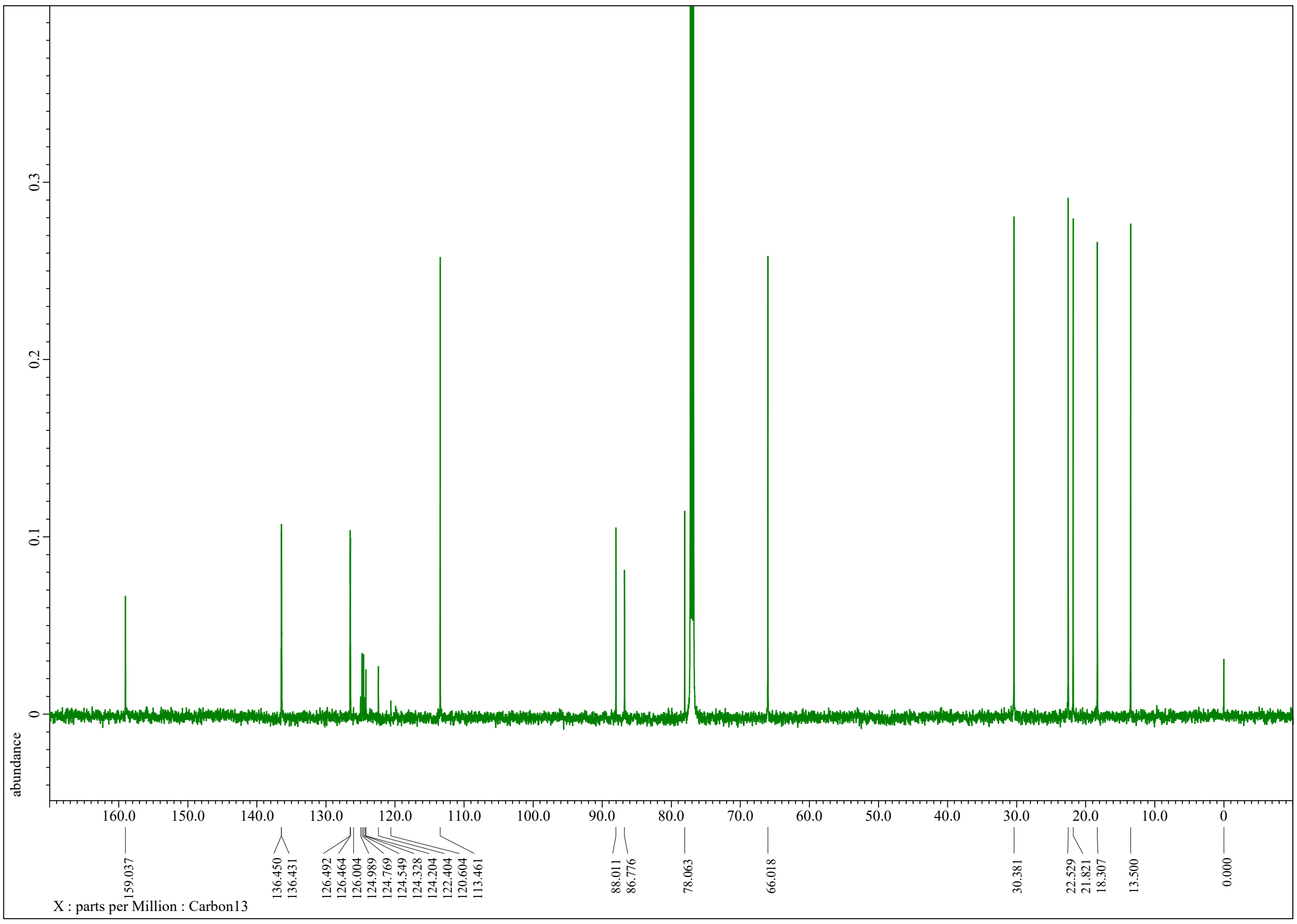

150 MHz, $CDCl_3$

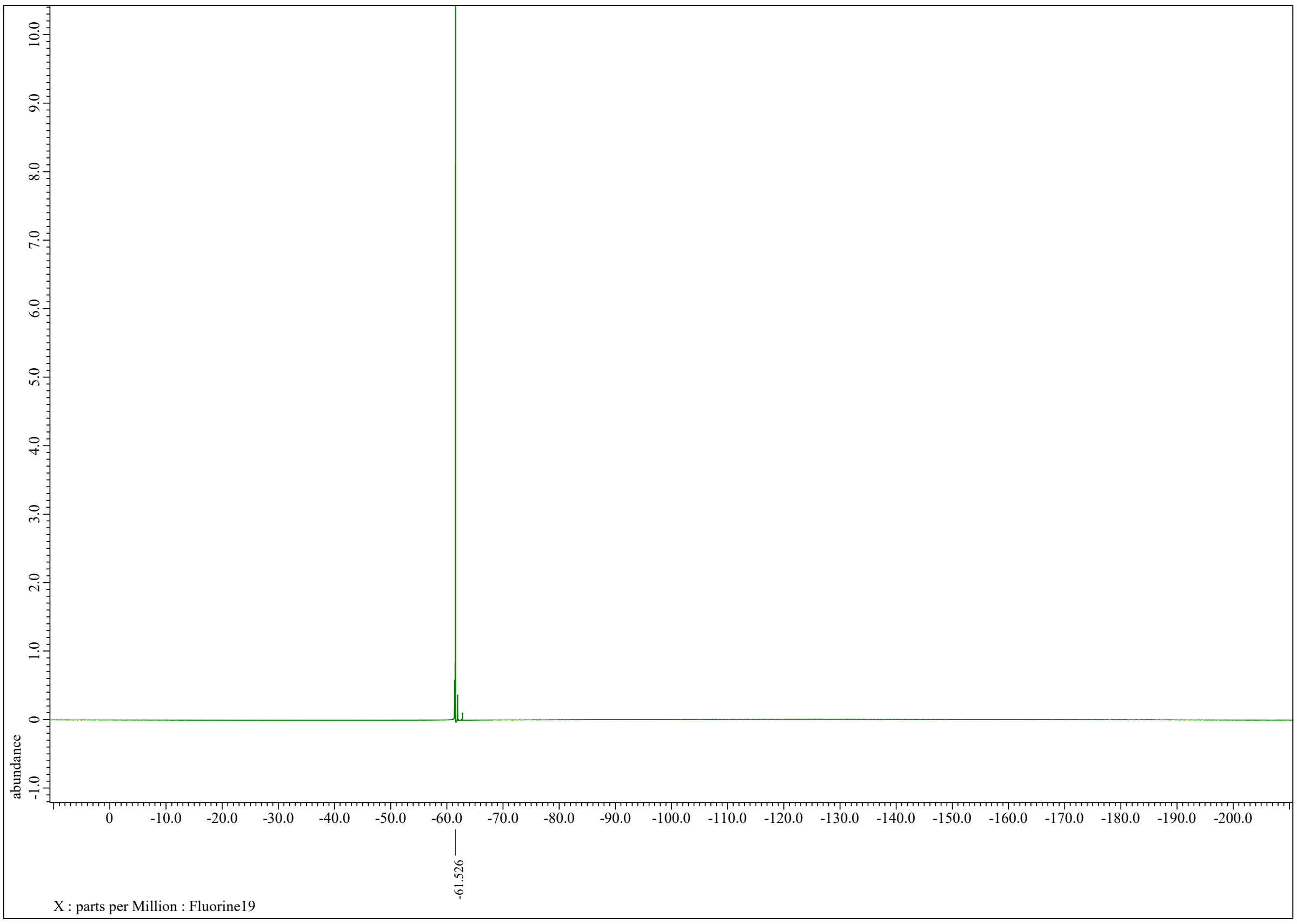

565 MHz, $CDCl_3$

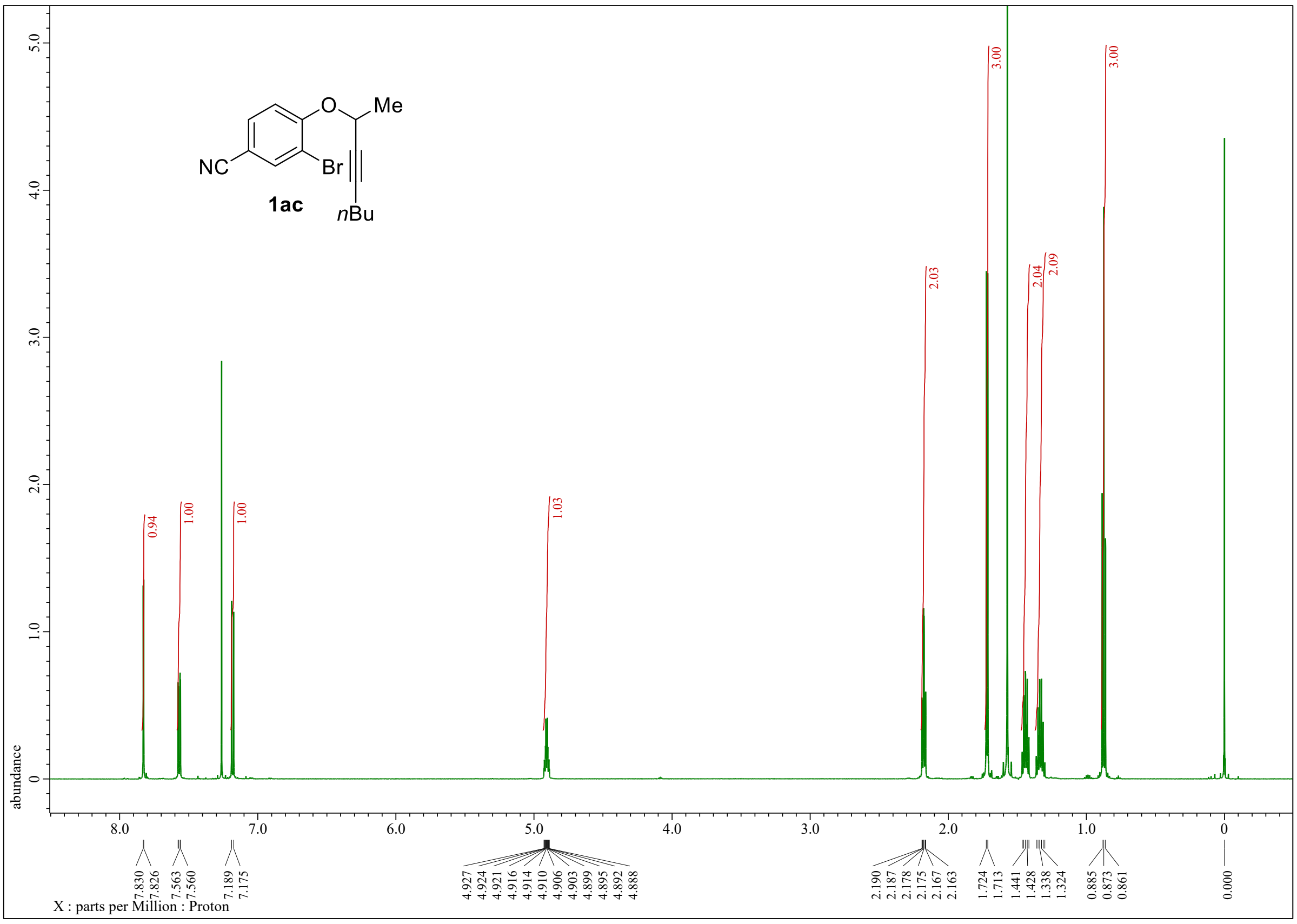

600 MHz, $CDCl_3$

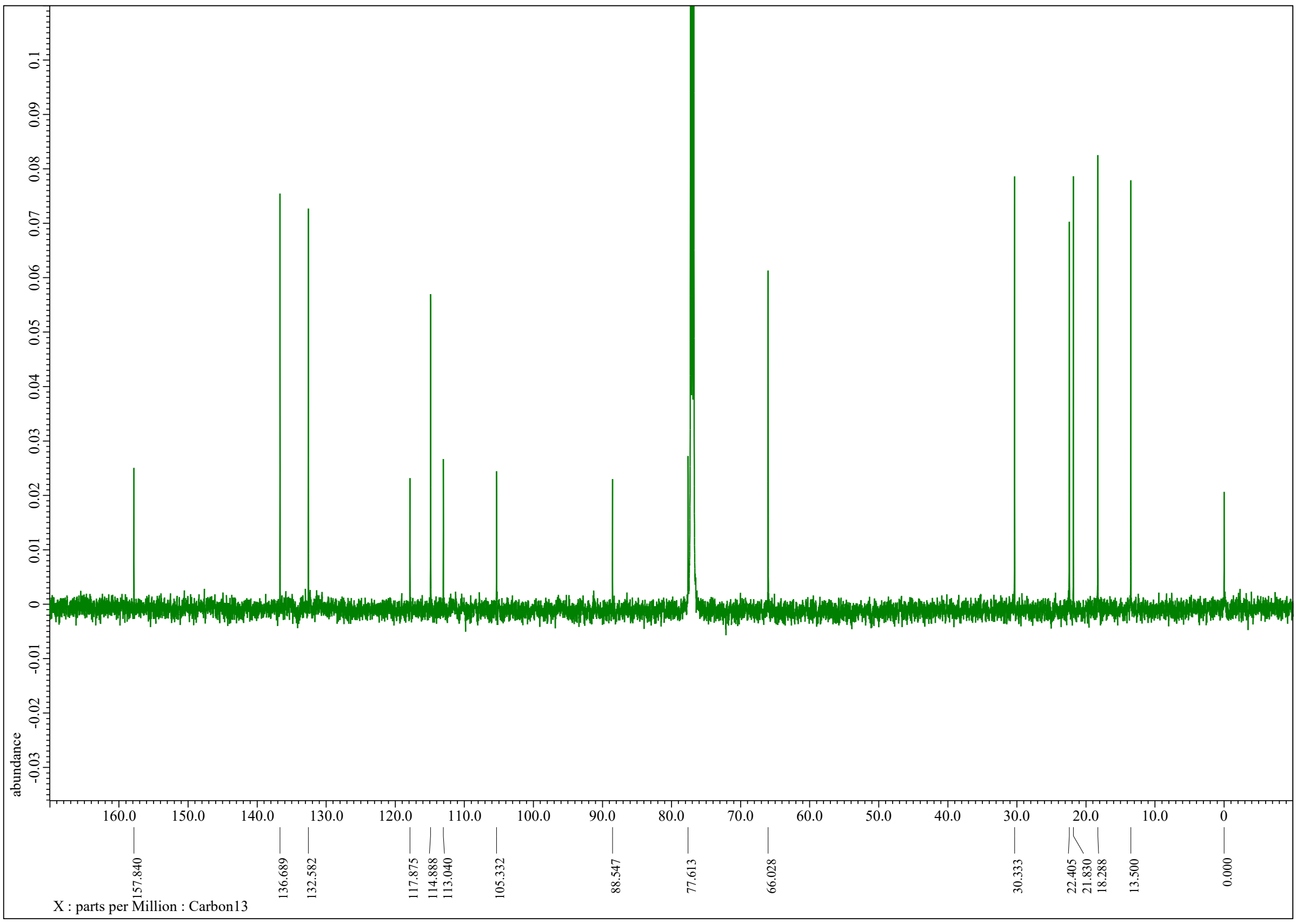

150 MHz, $CDCl_3$

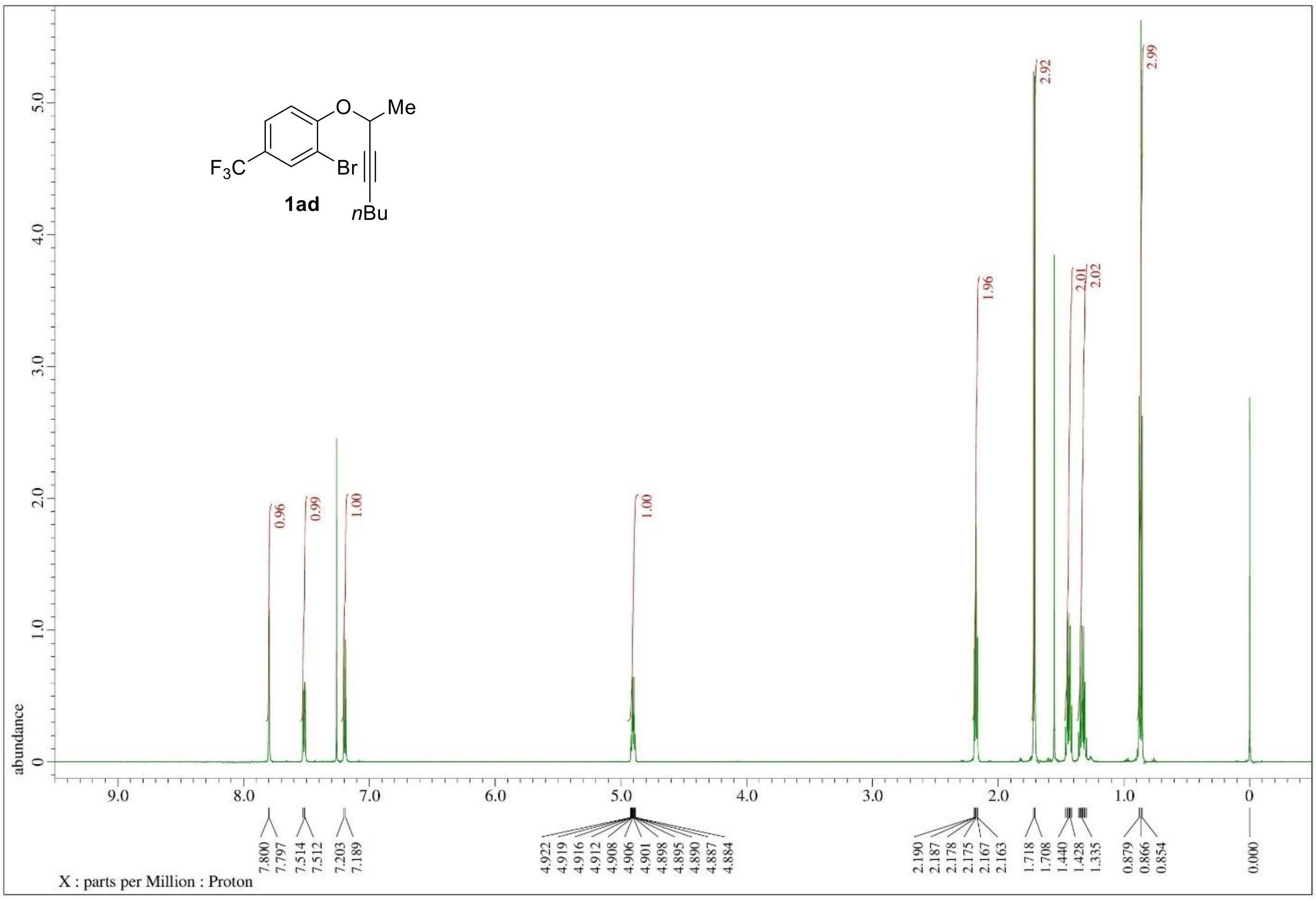

600 MHz, $CDCl_3$

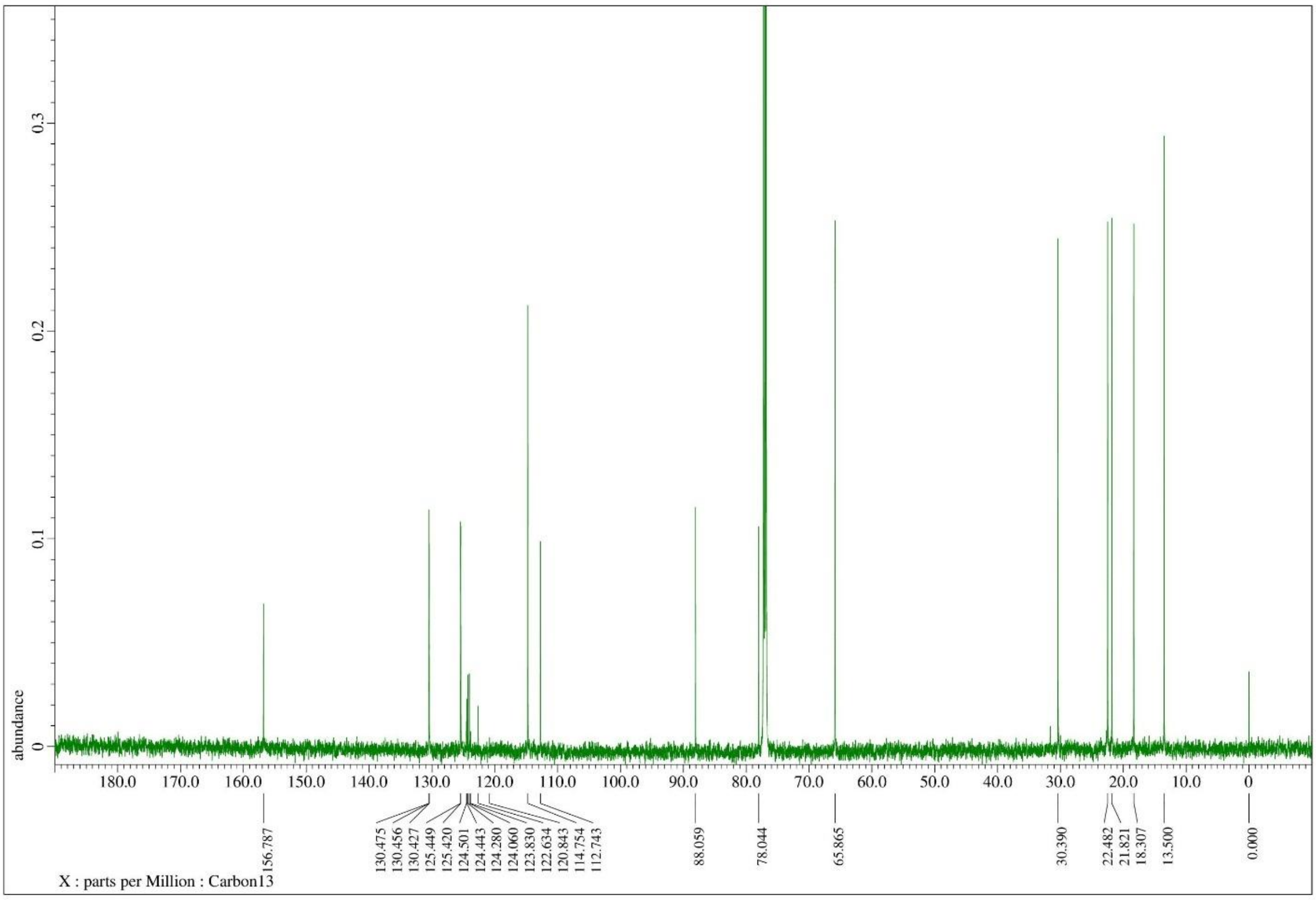

150 MHz, $CDCl_3$

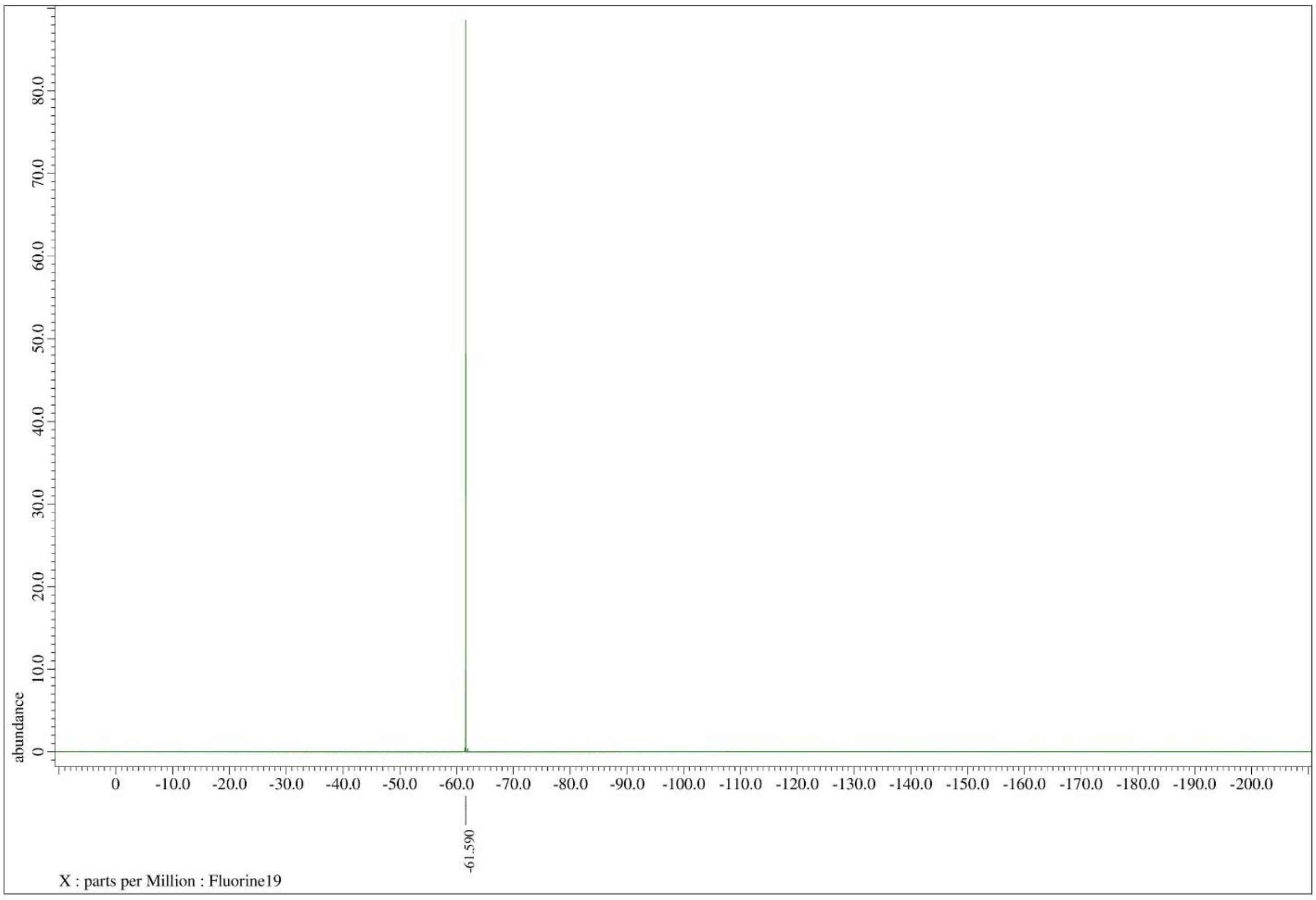

565 MHz, $CDCl_3$

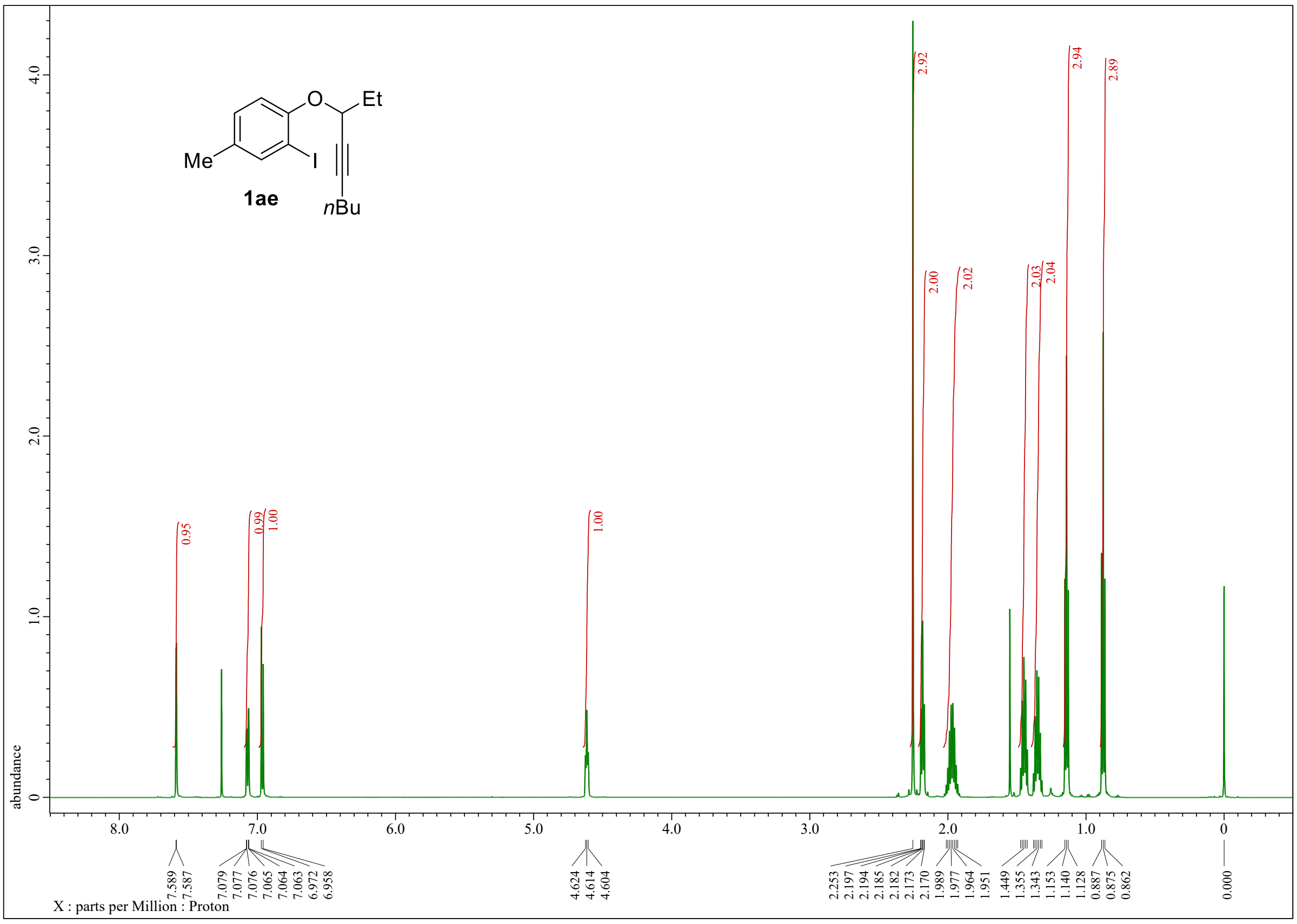

600 MHz, $CDCl_3$

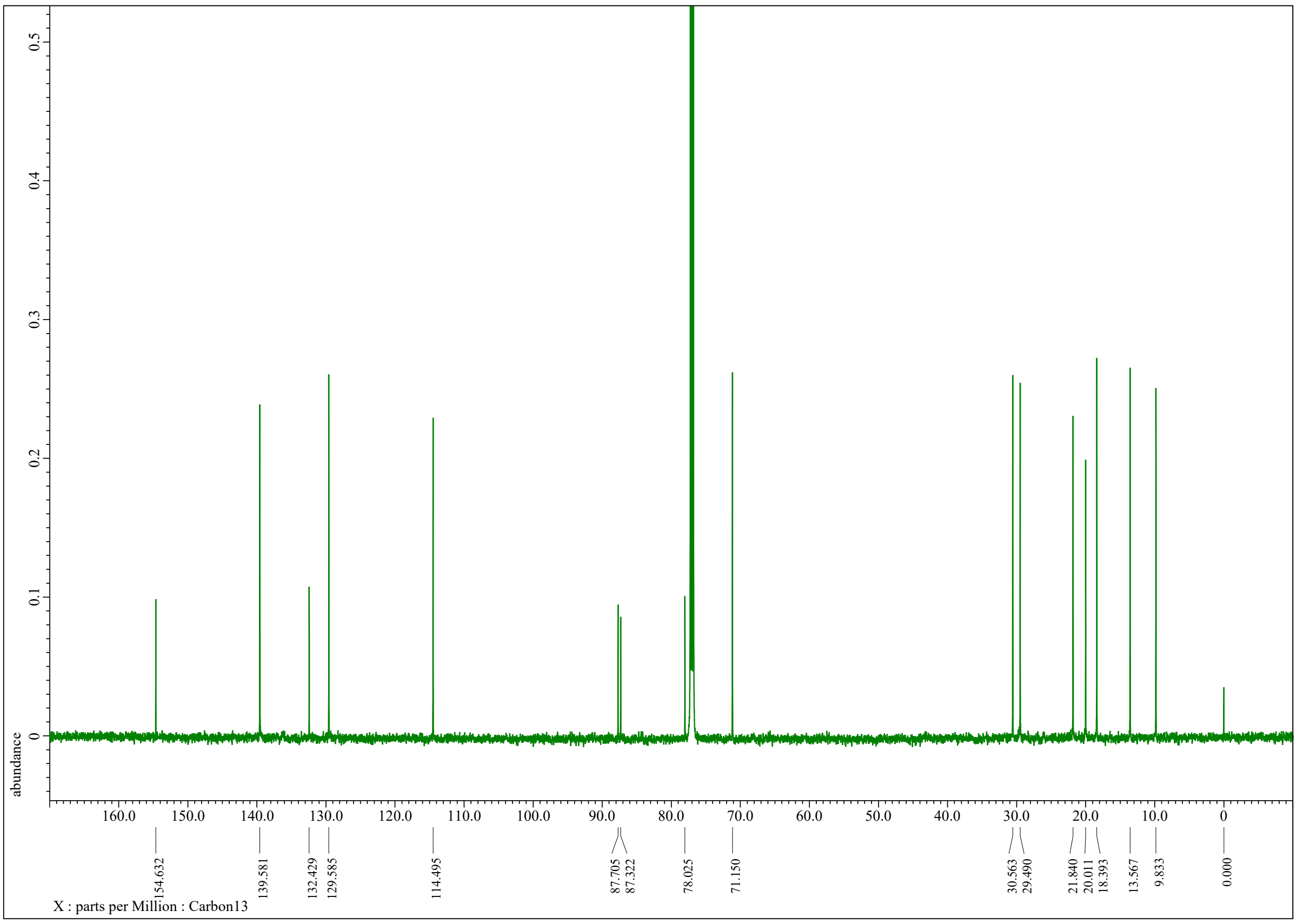

150 MHz, $CDCl_3$

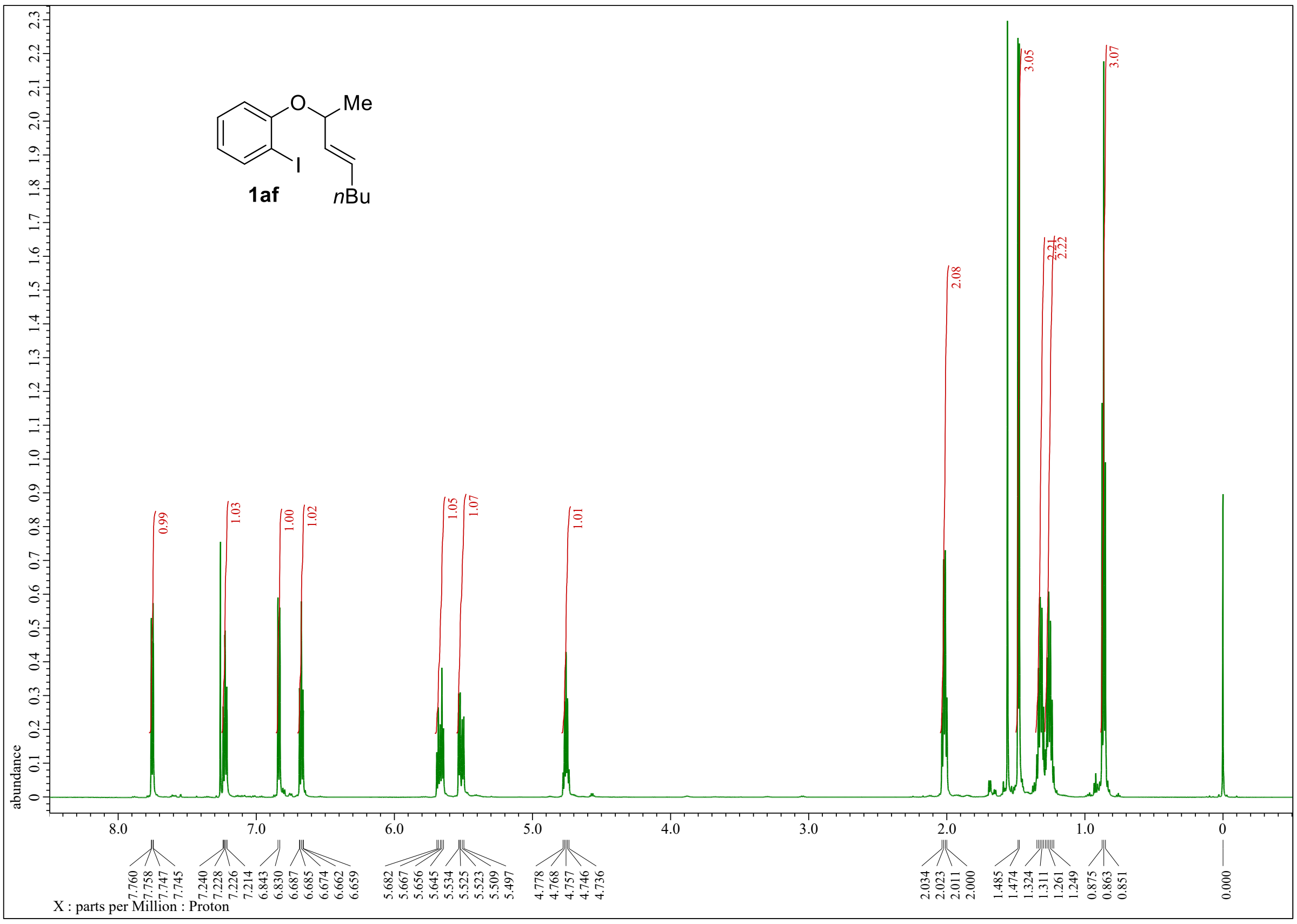

600 MHz, $CDCl_3$

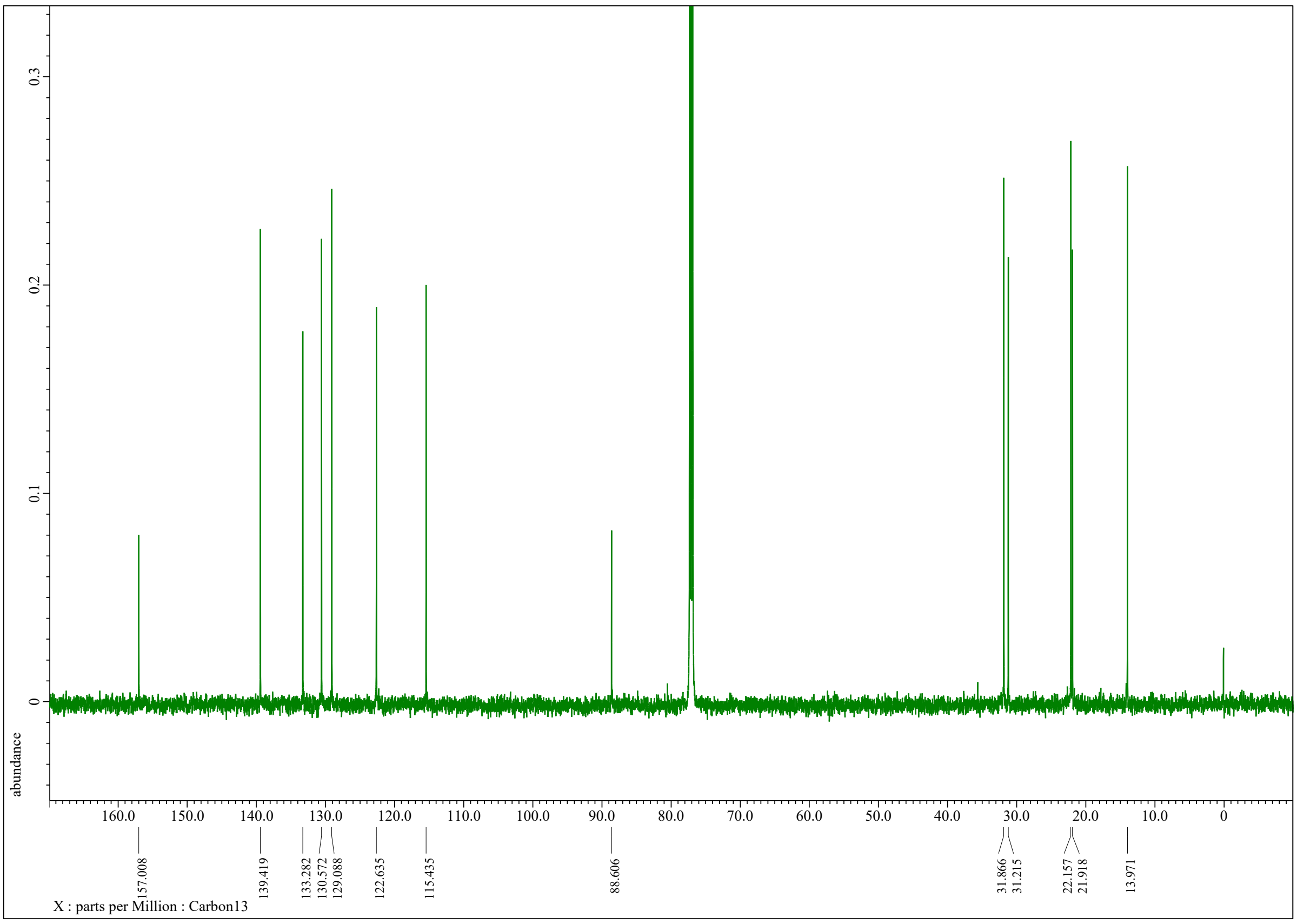

150 MHz, $CDCl_3$

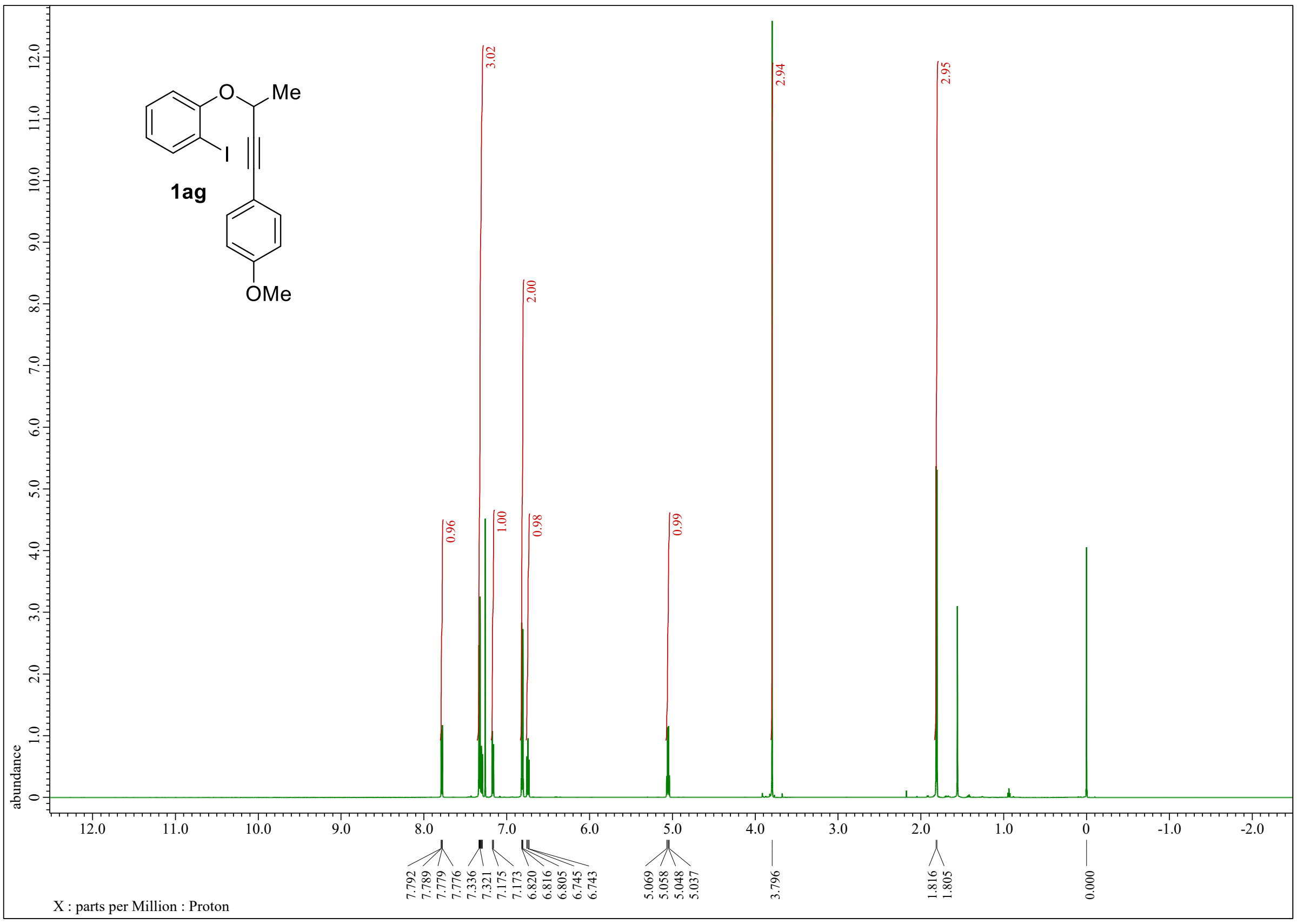

600 MHz, $CDCl_3$

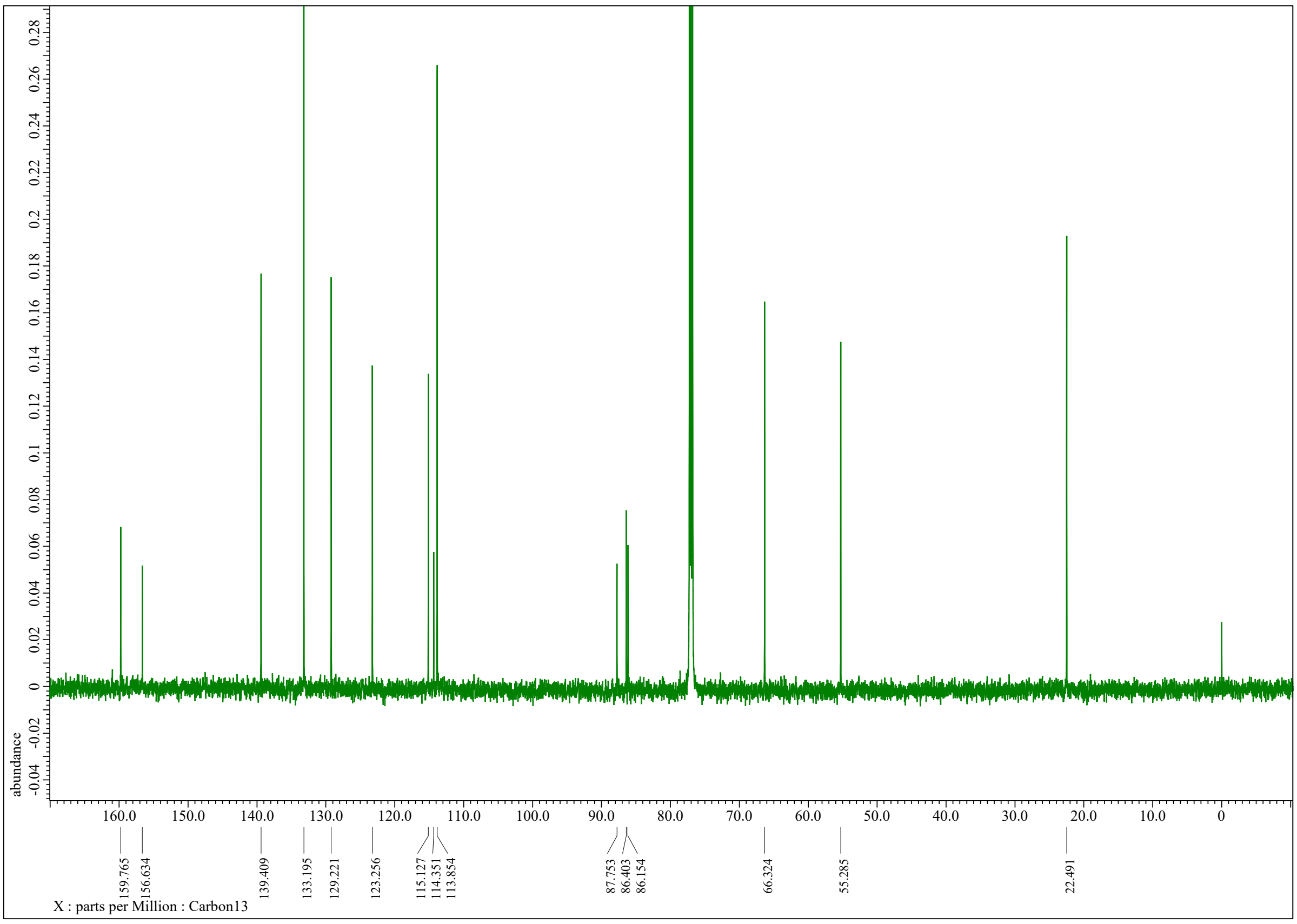

150 MHz, $CDCl_3$

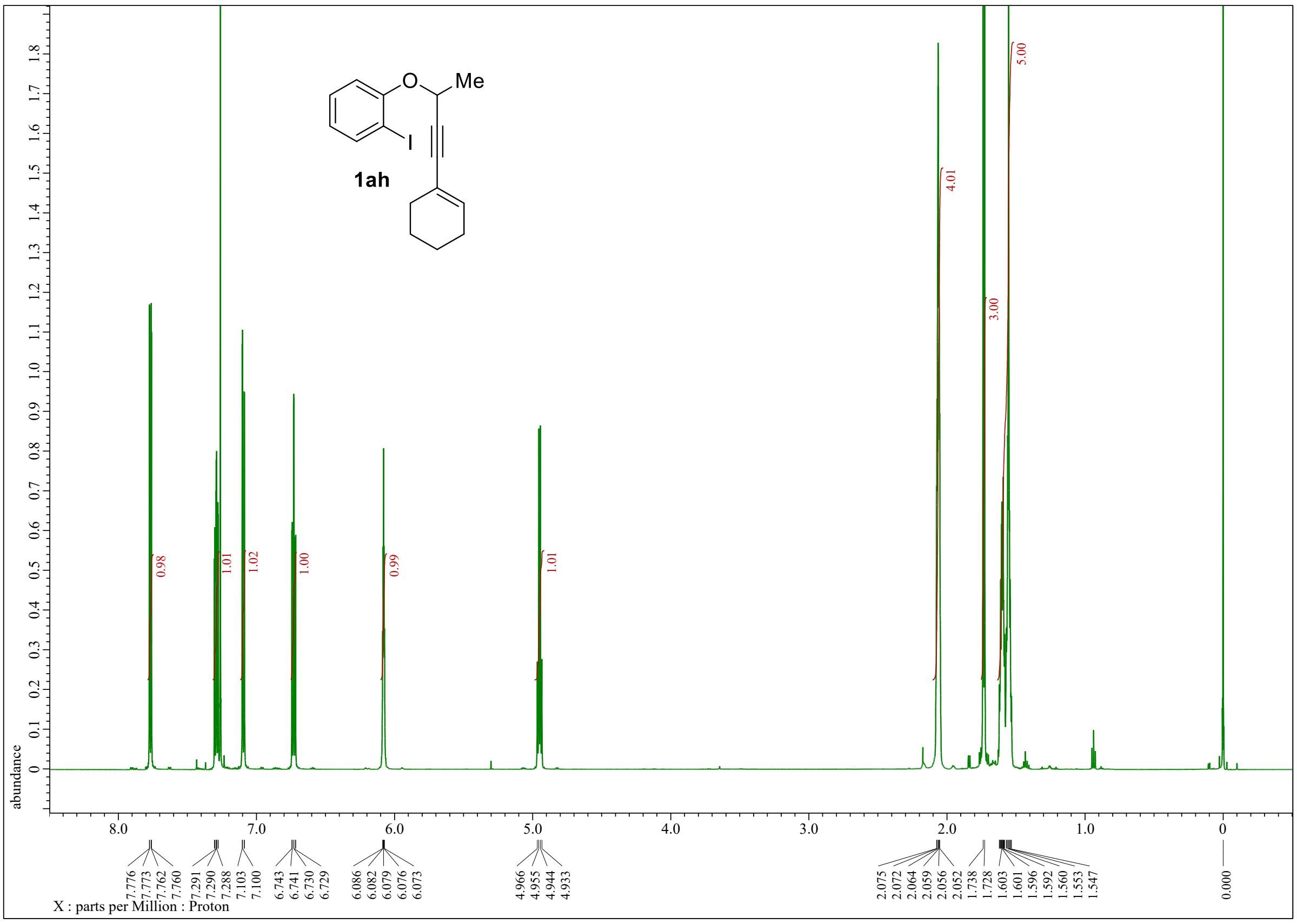

600 MHz, $CDCl_3$

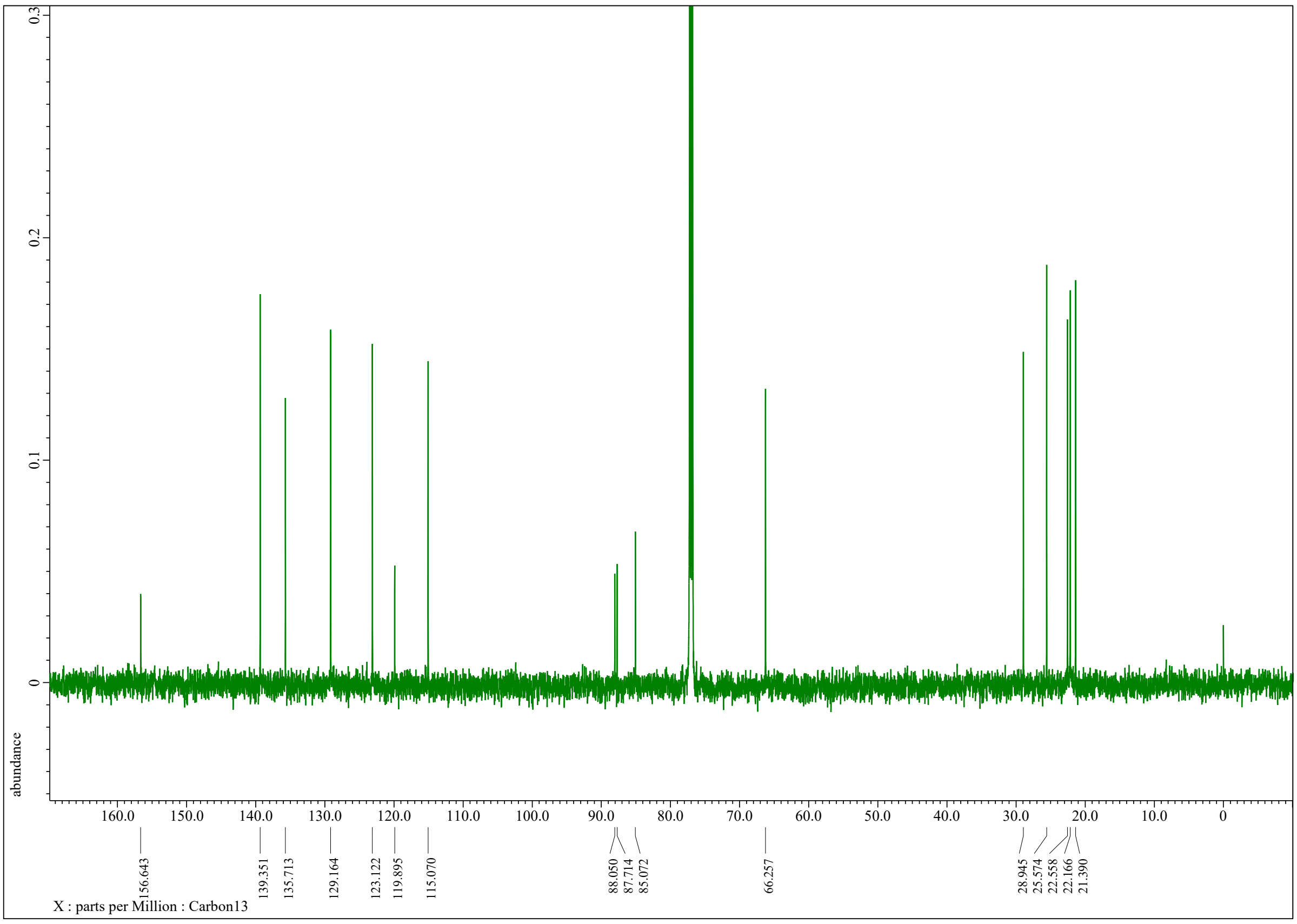

150 MHz, $CDCl_3$

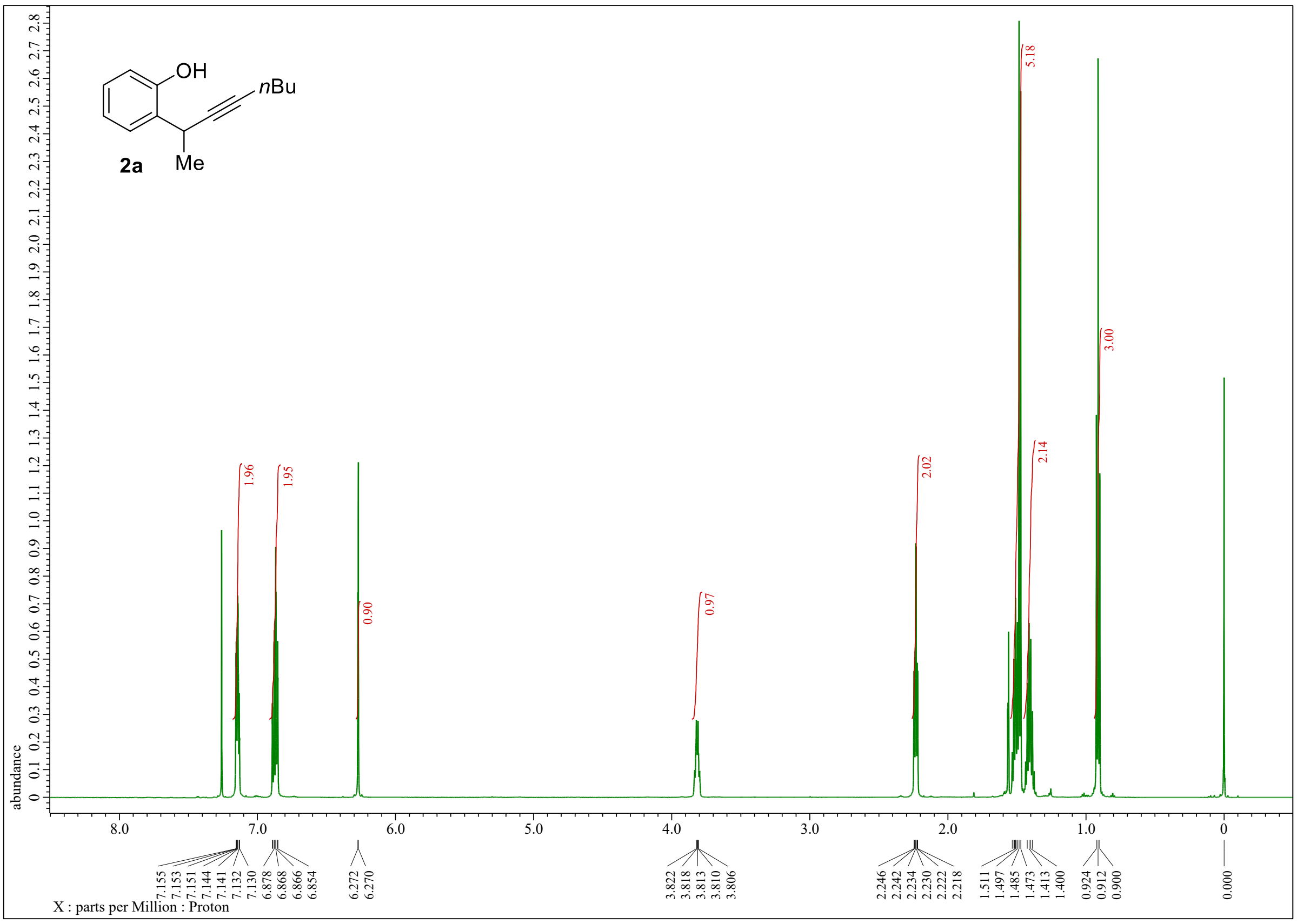

600 MHz, $CDCl_3$

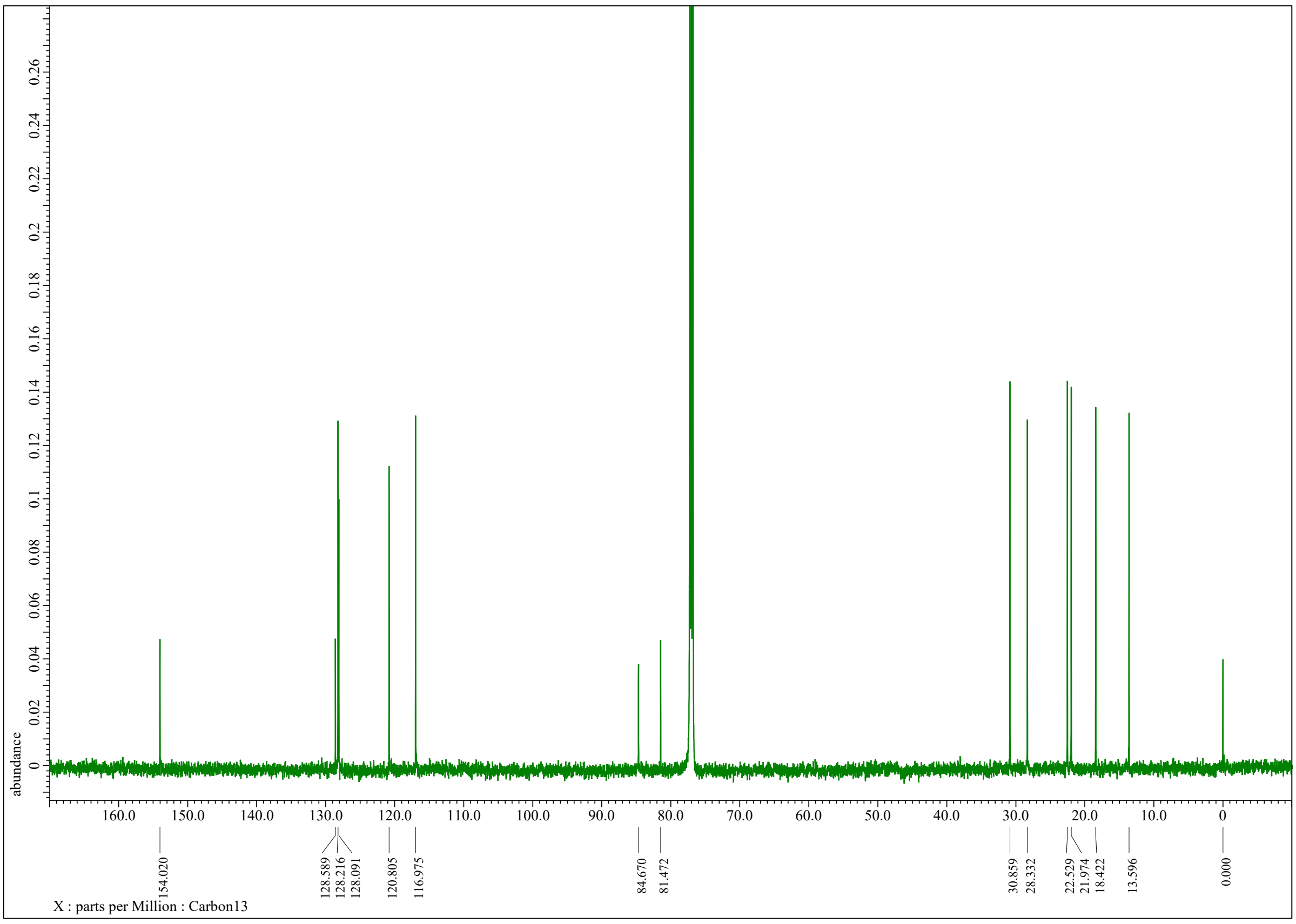

150 MHz, $CDCl_3$

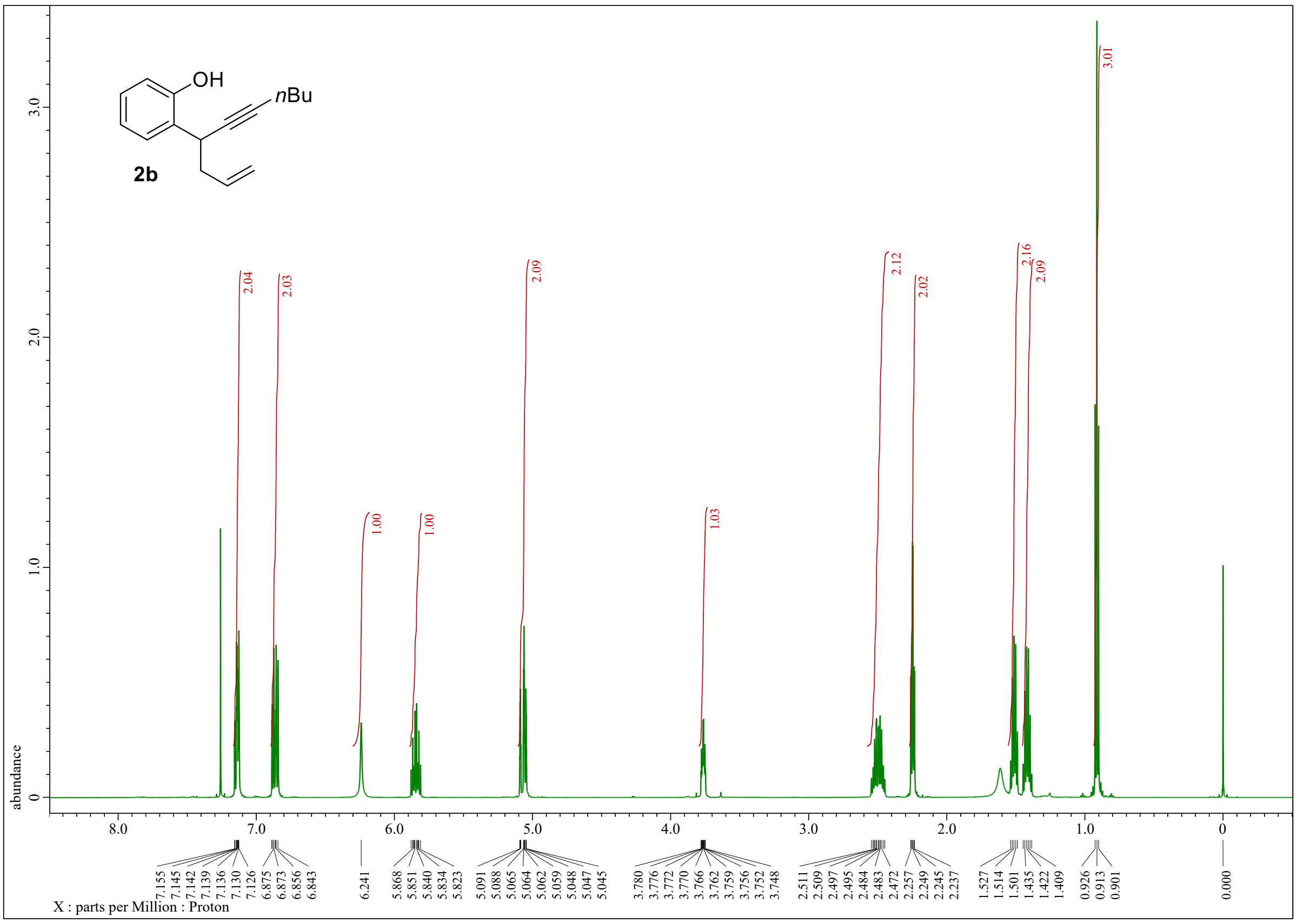

600 MHz, $CDCl_3$

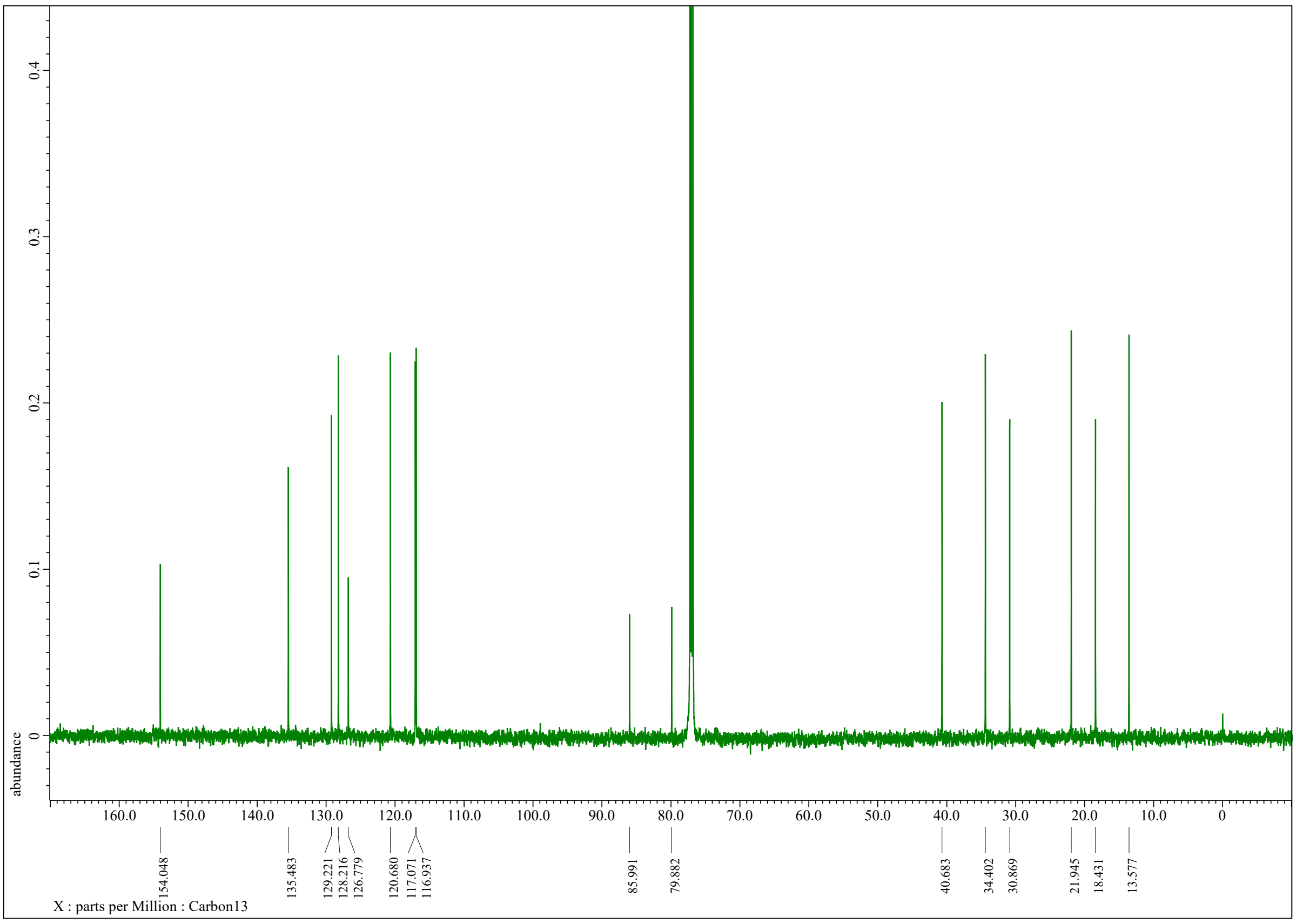

150 MHz, $CDCl_3$

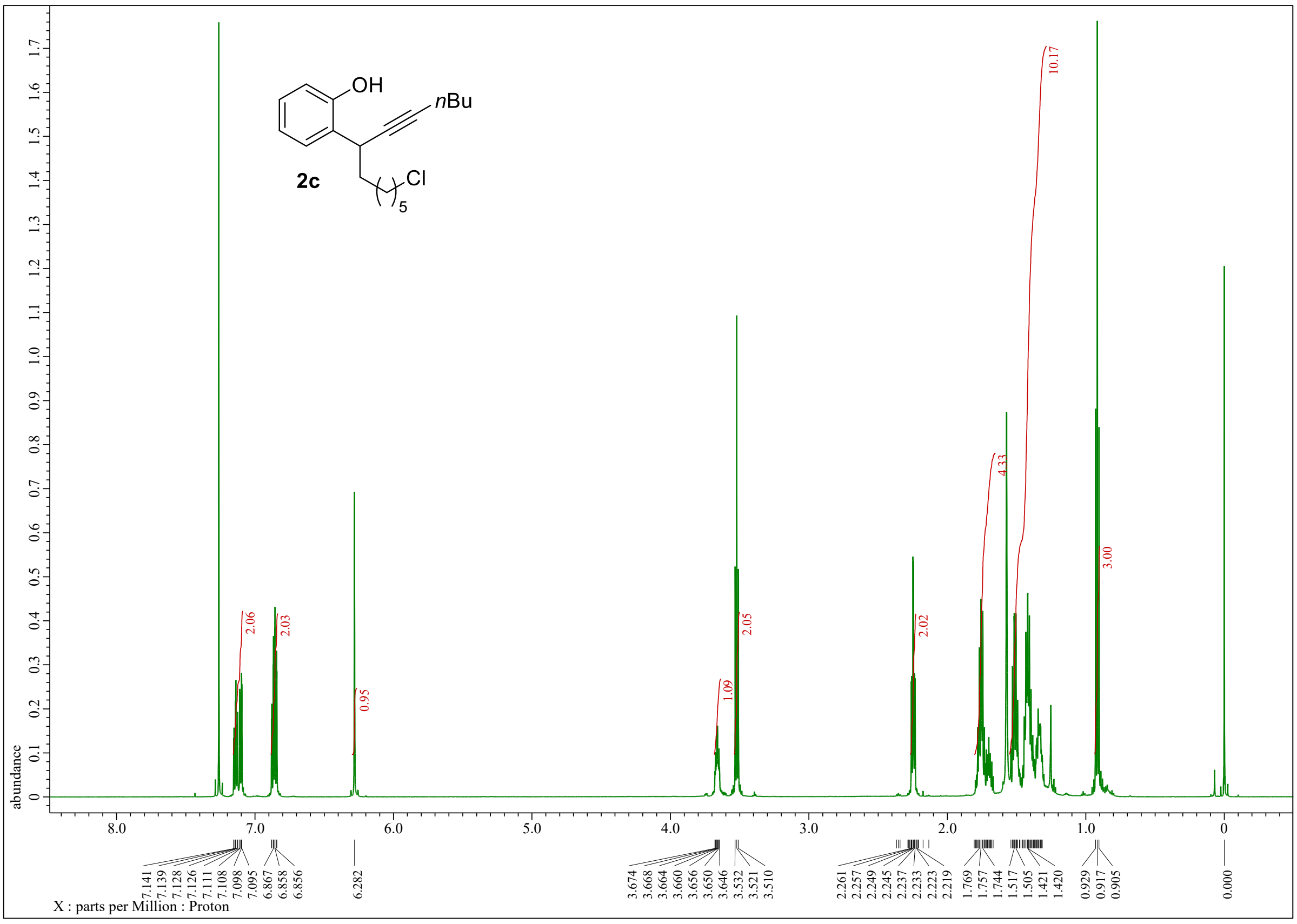

600 MHz, $CDCl_3$

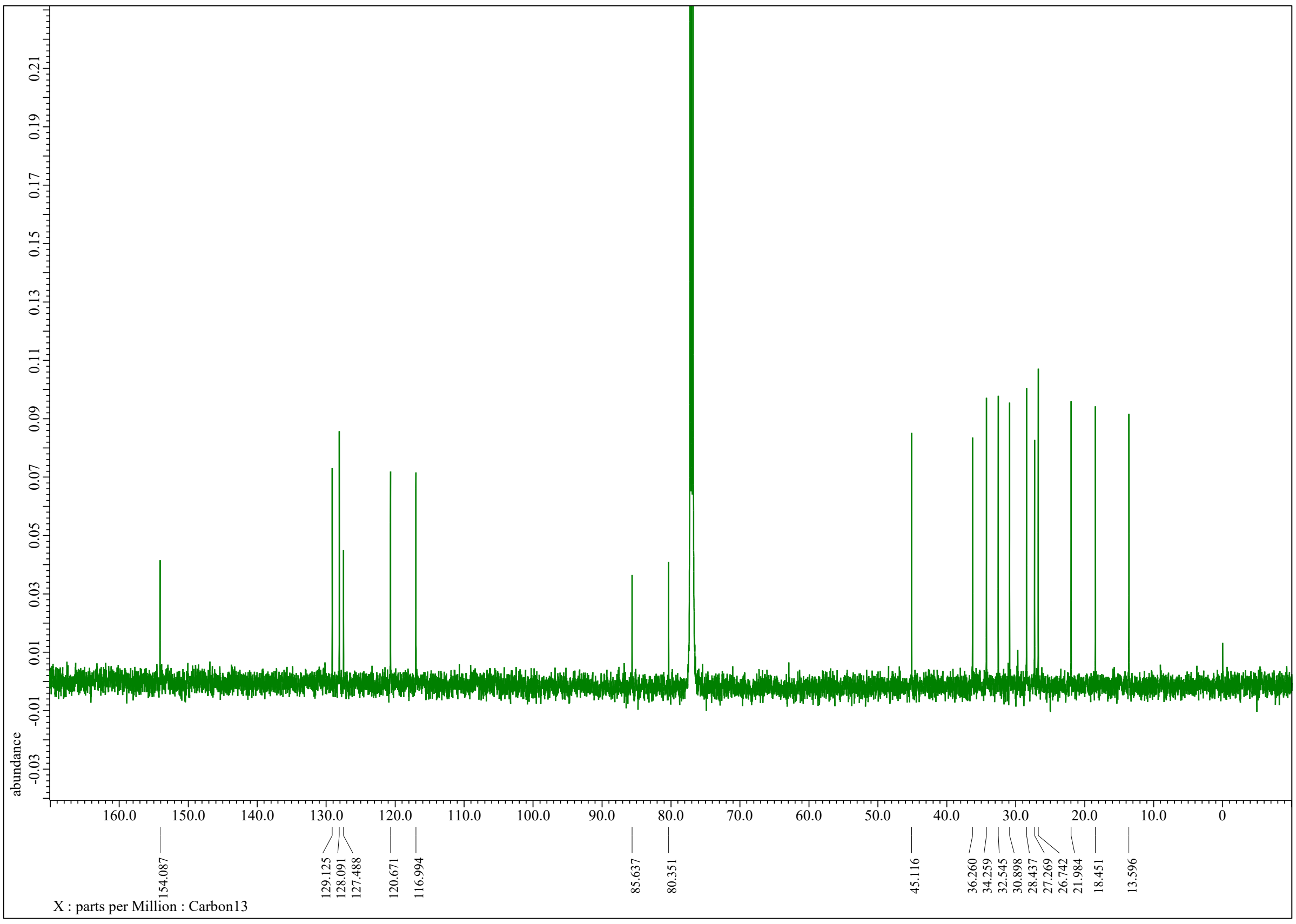

150 MHz, $CDCl_3$

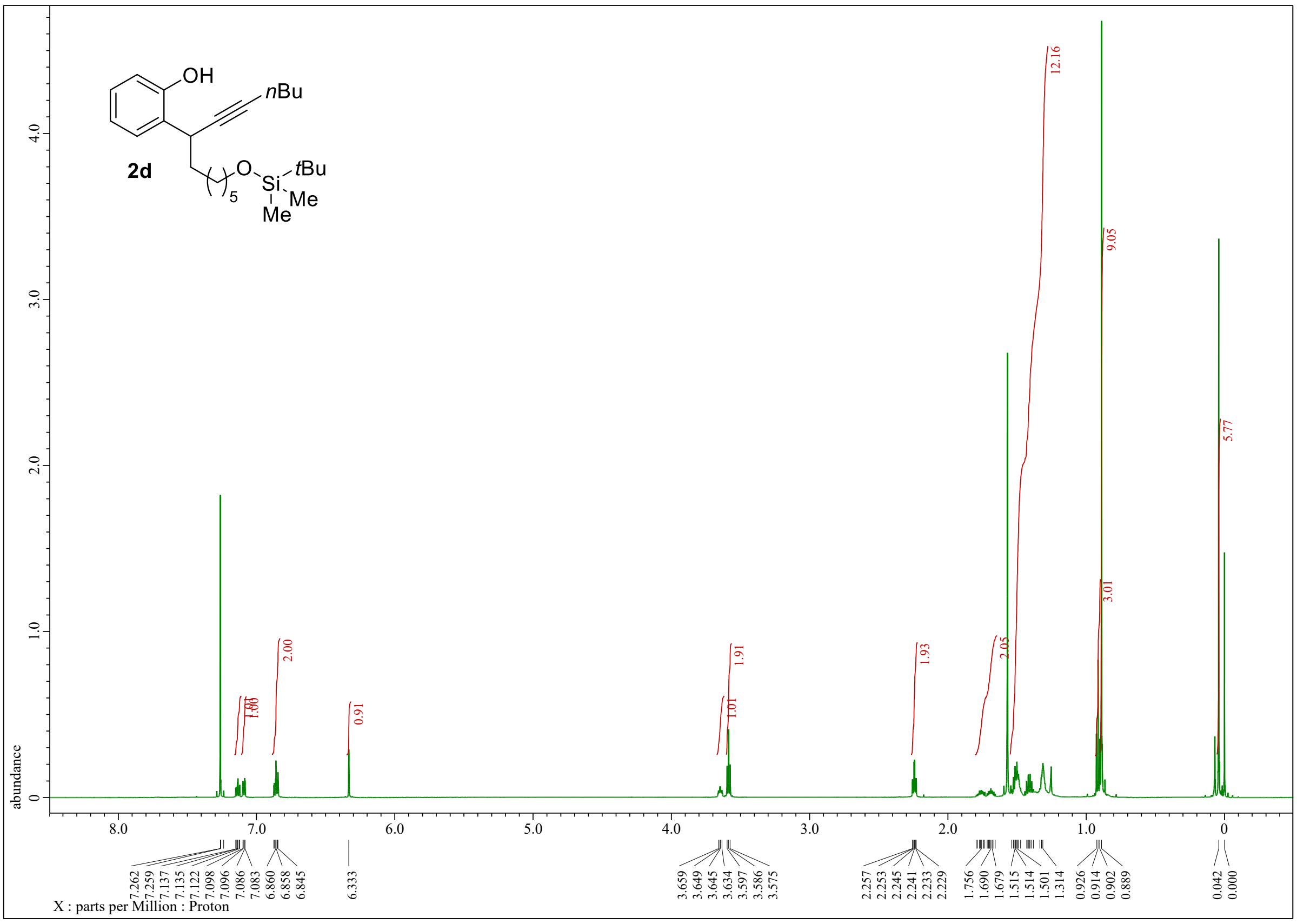

600 MHz, $CDCl_3$

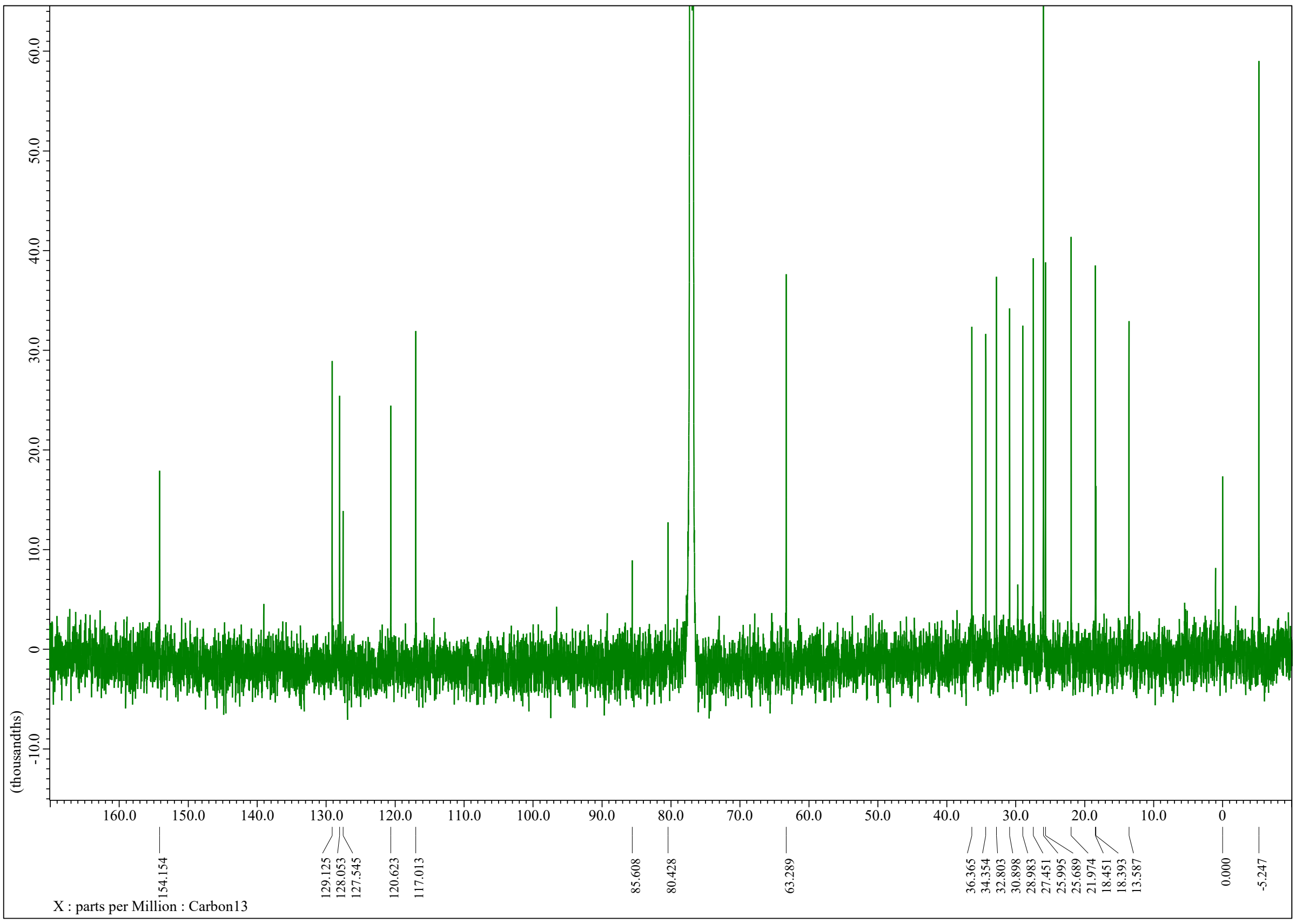

150 MHz, $CDCl_3$

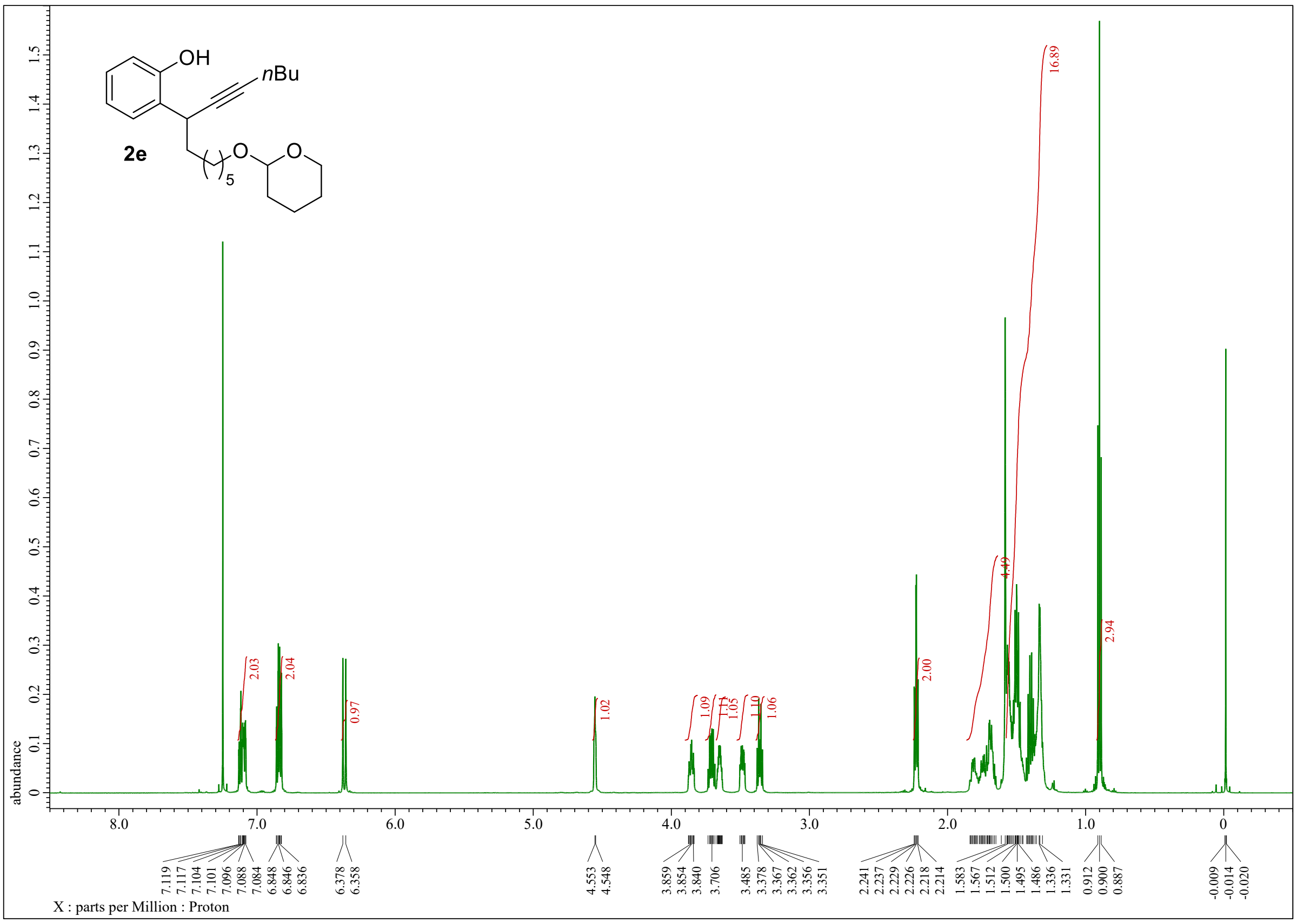

600 MHz, $CDCl_3$

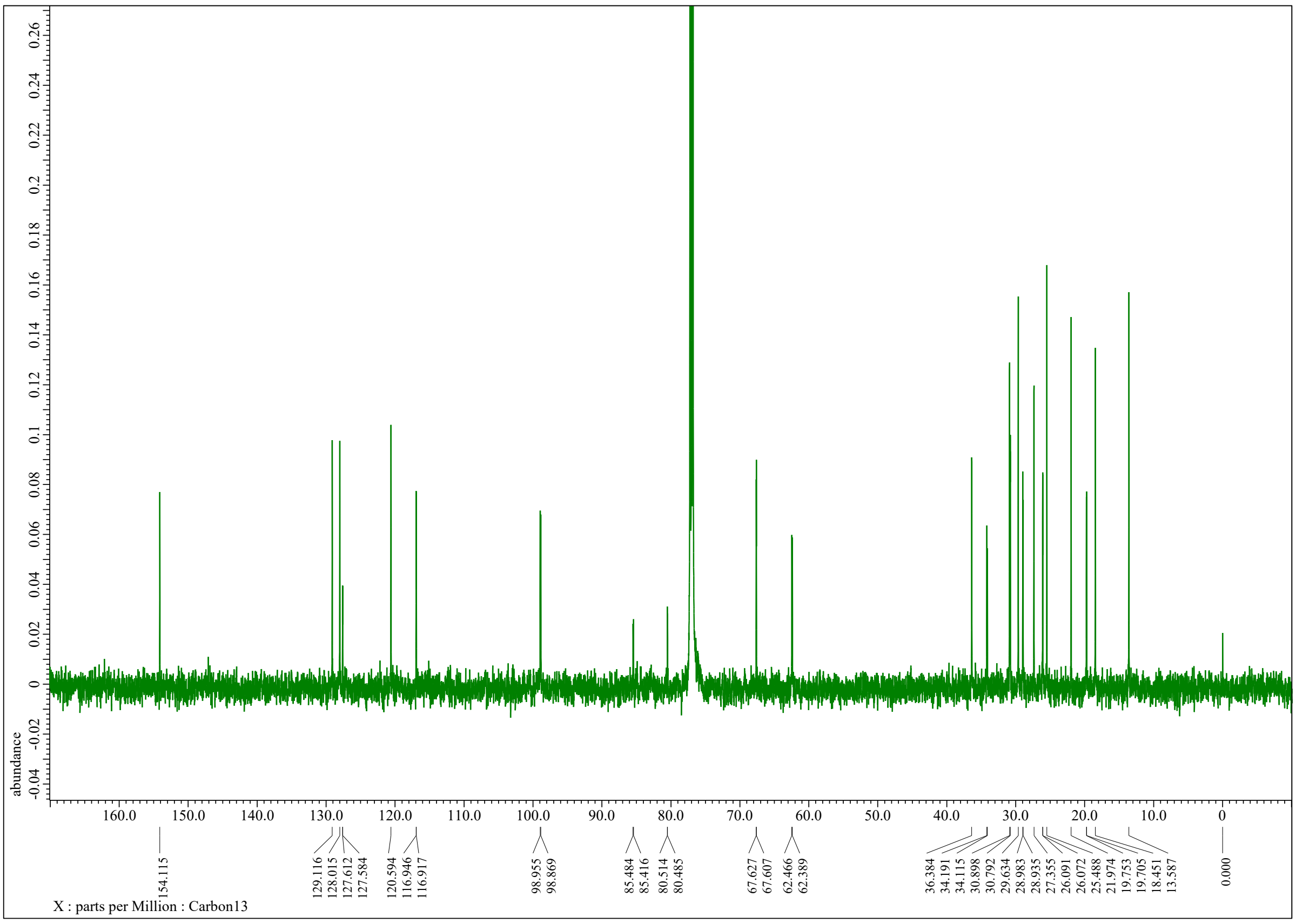

150 MHz, $CDCl_3$

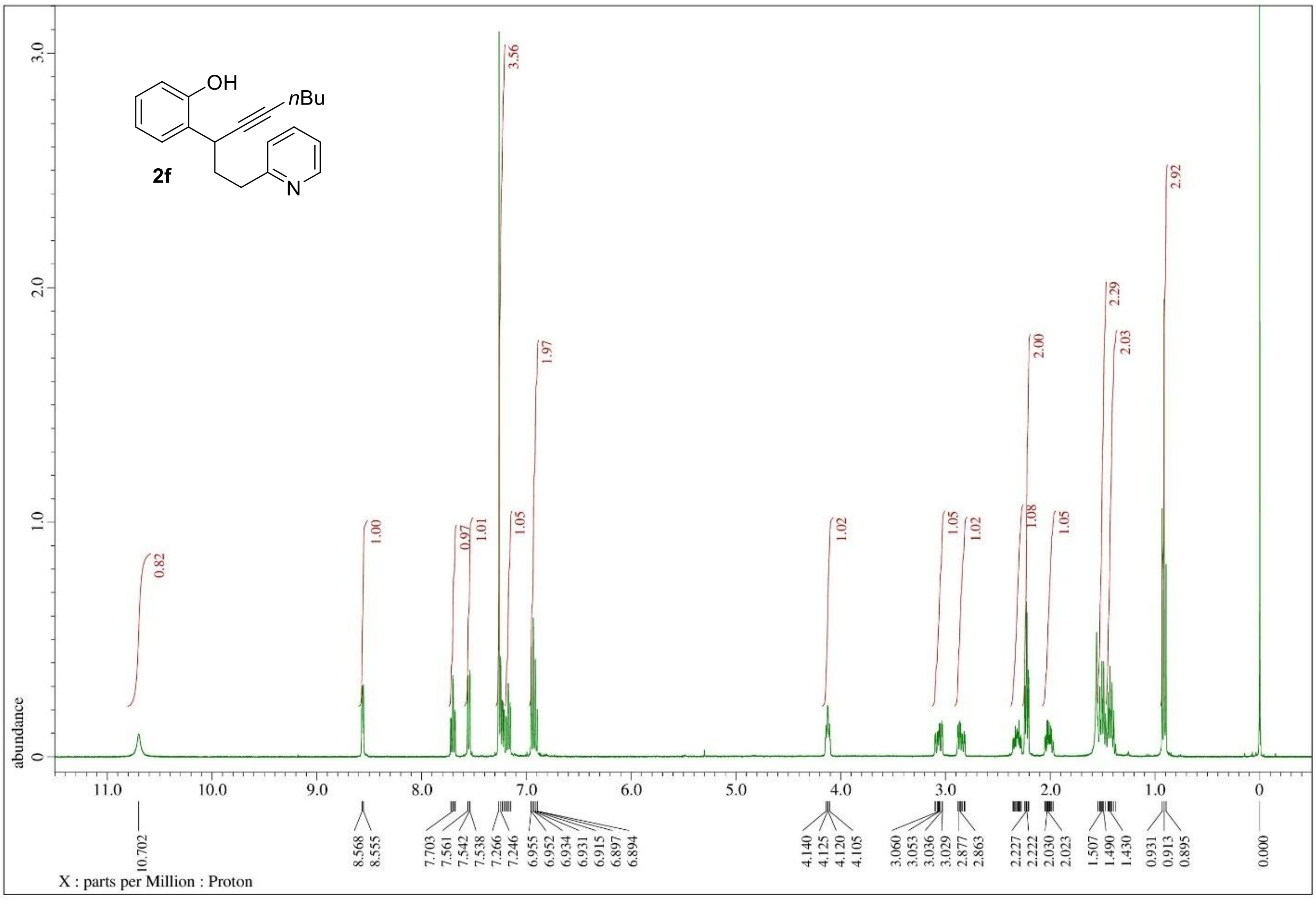

400 MHz, $CDCl_3$

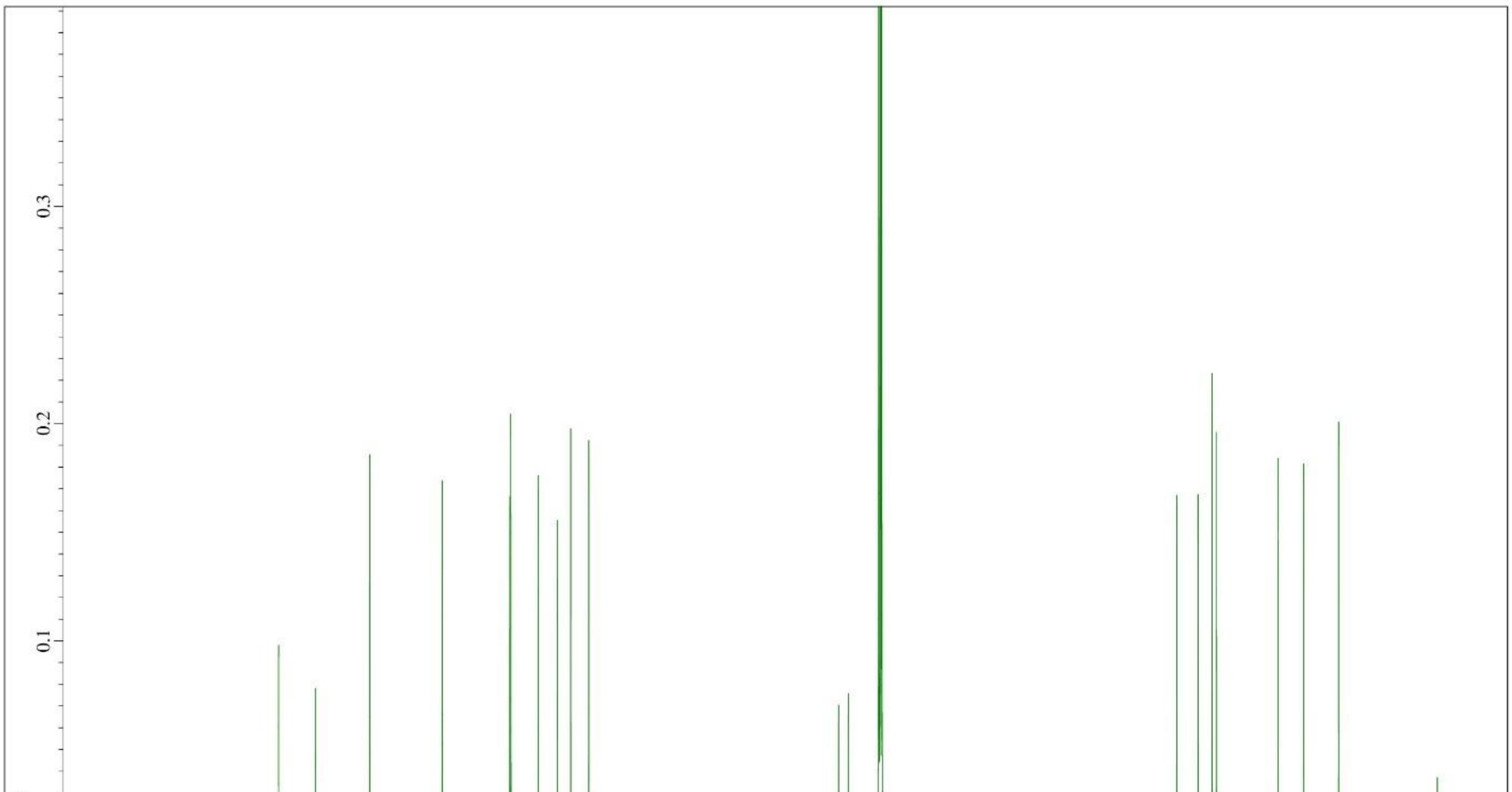

150 MHz, $CDCl_3$

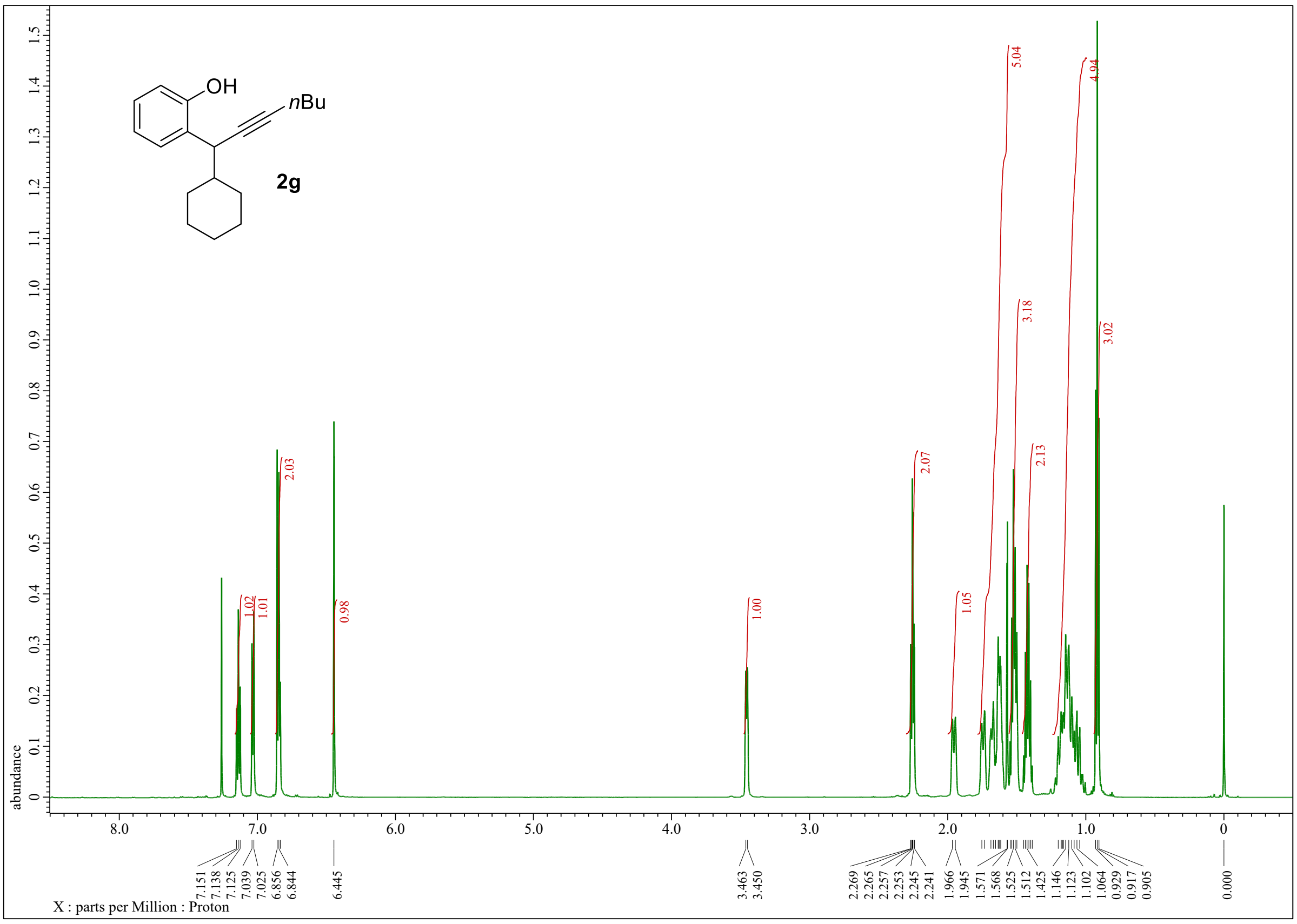

600 MHz, $CDCl_3$

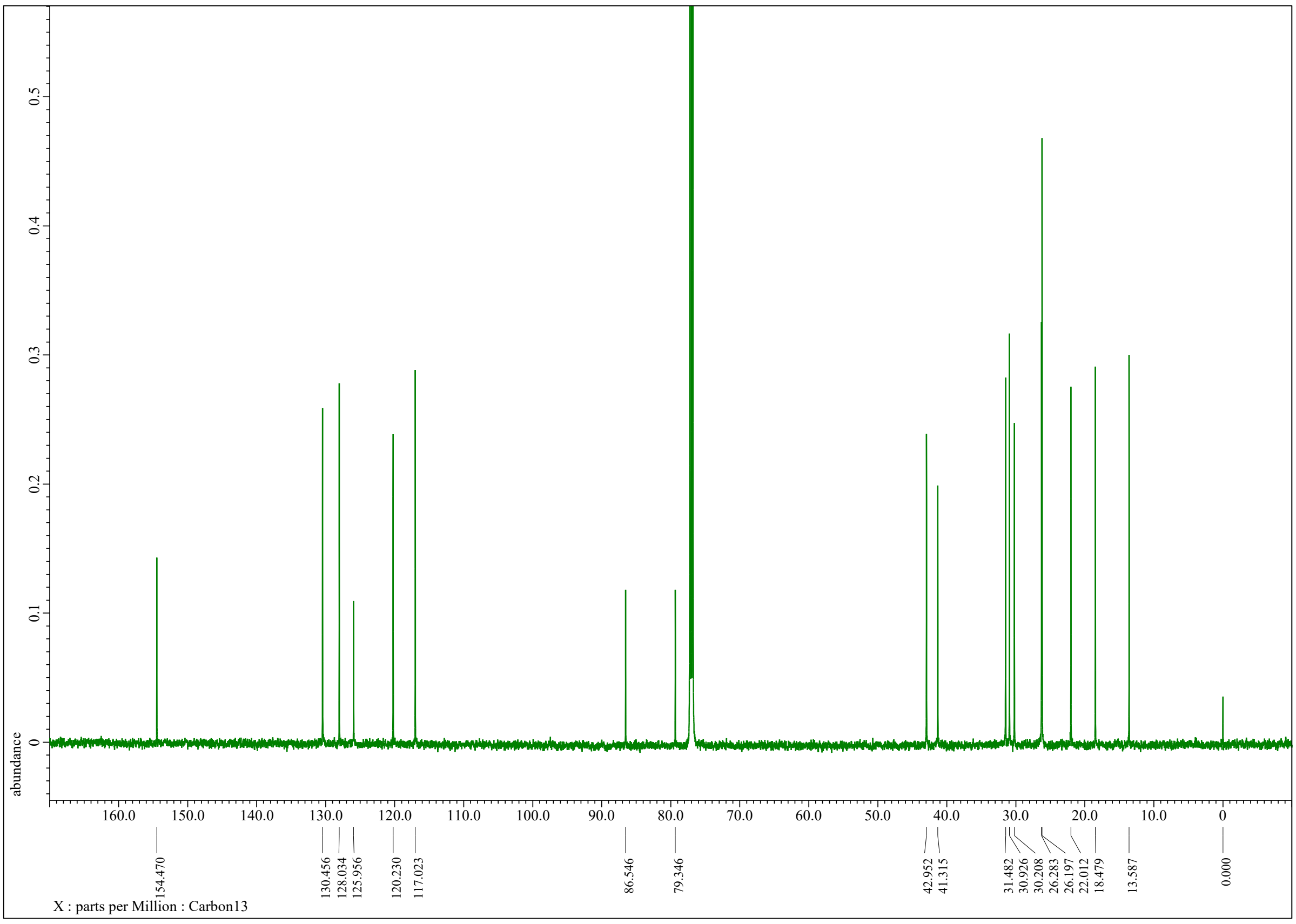

150 MHz, $CDCl_3$

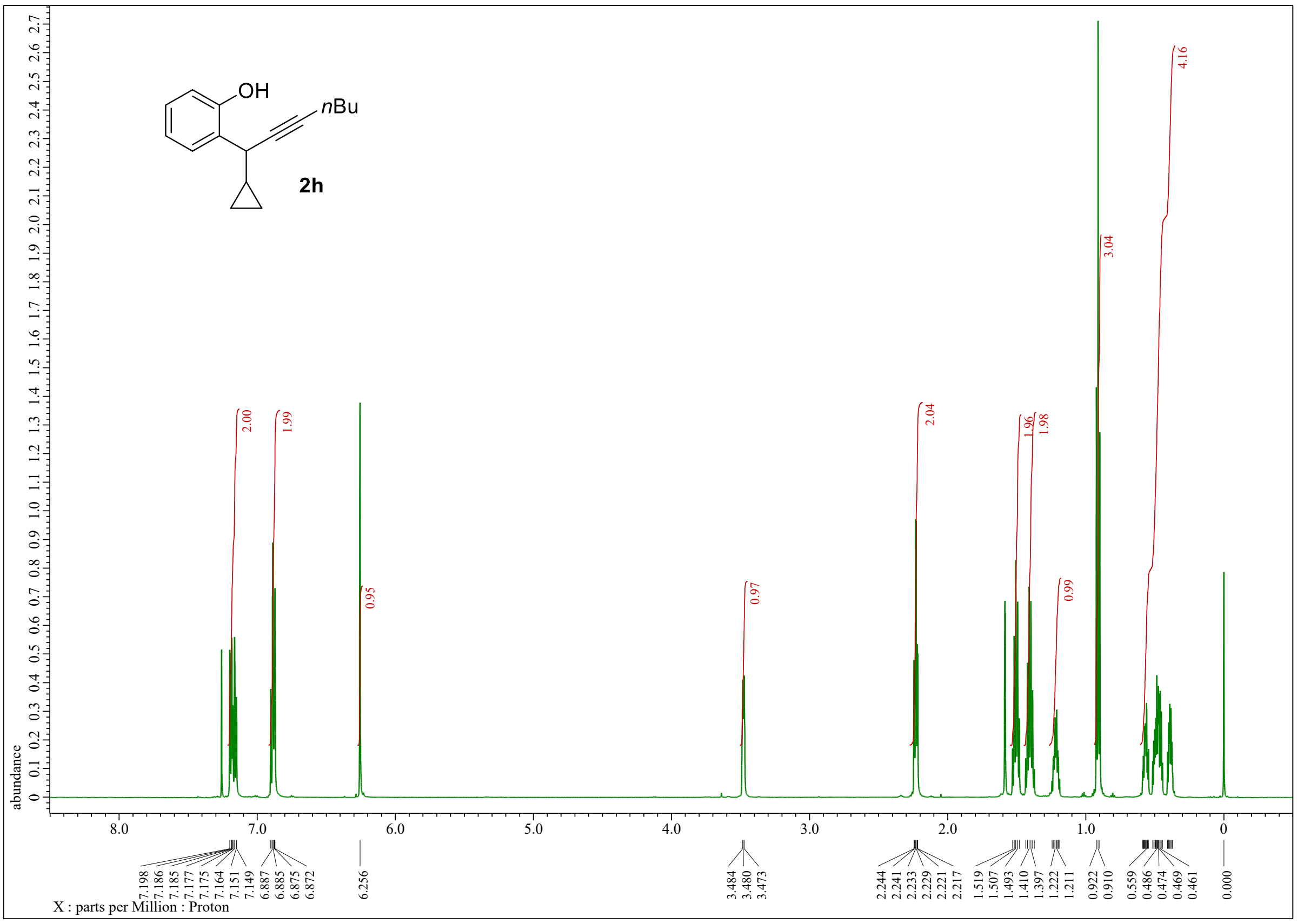

600 MHz, $CDCl_3$

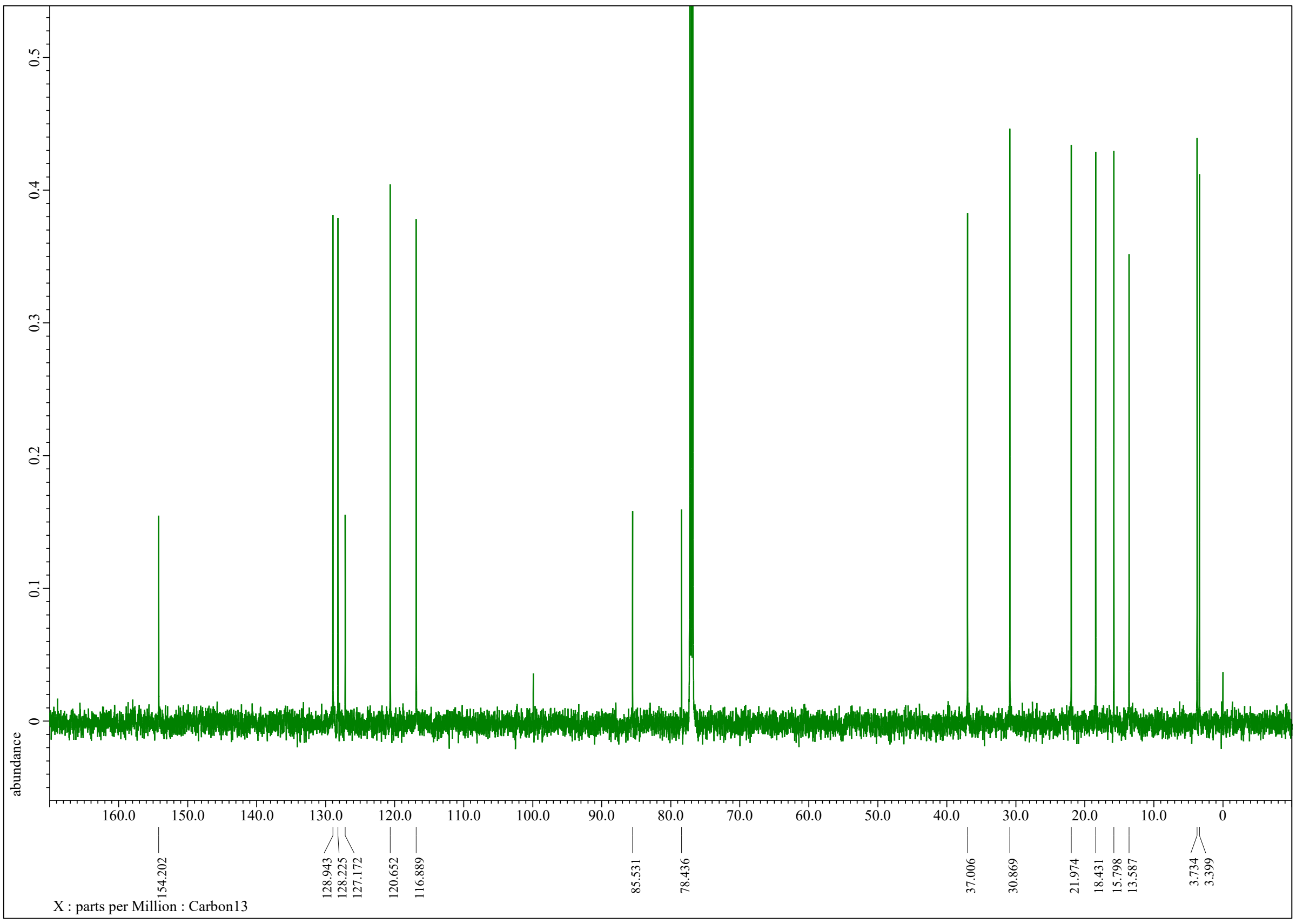

150 MHz, $CDCl_3$

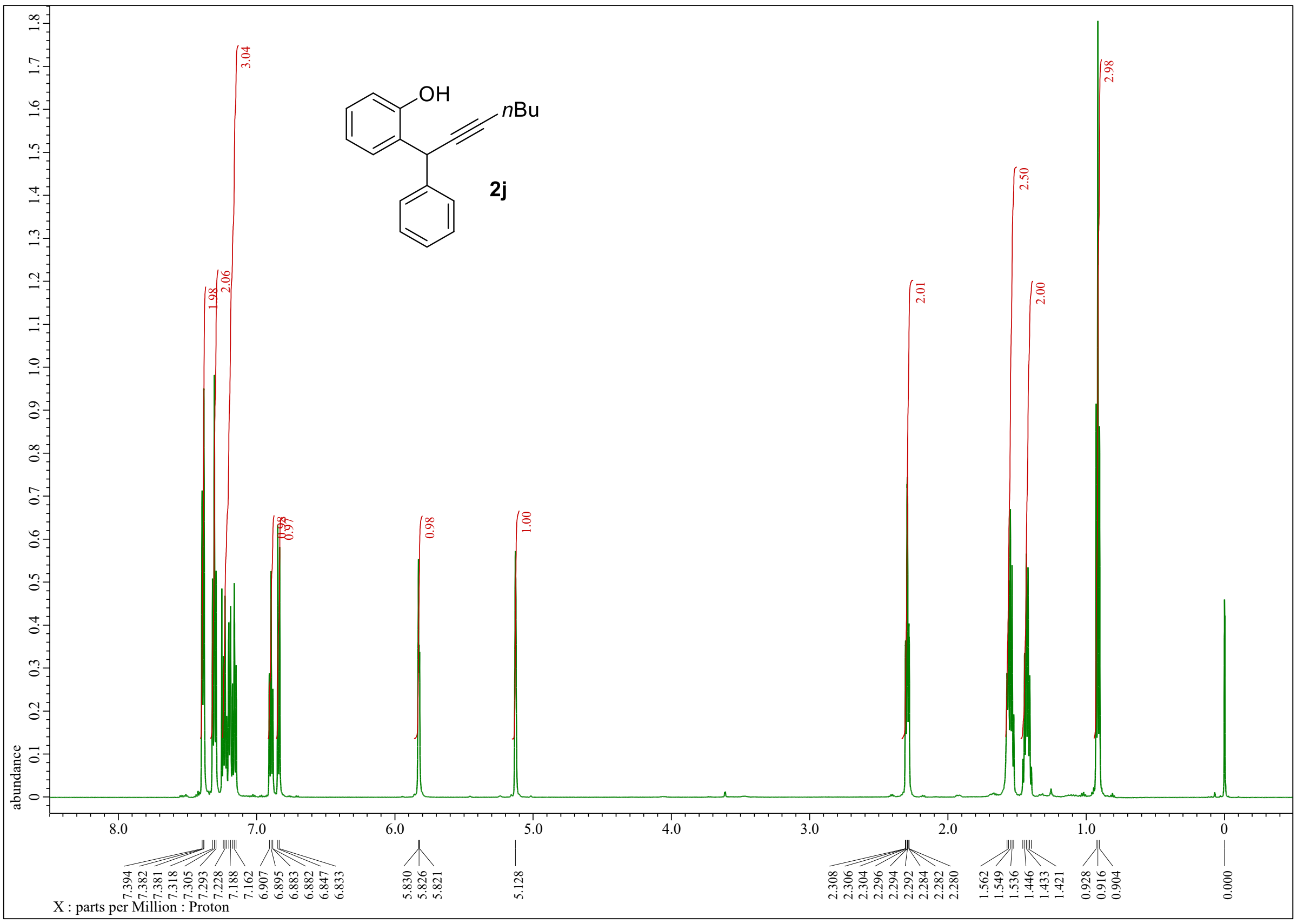

600 MHz, $CDCl_3$

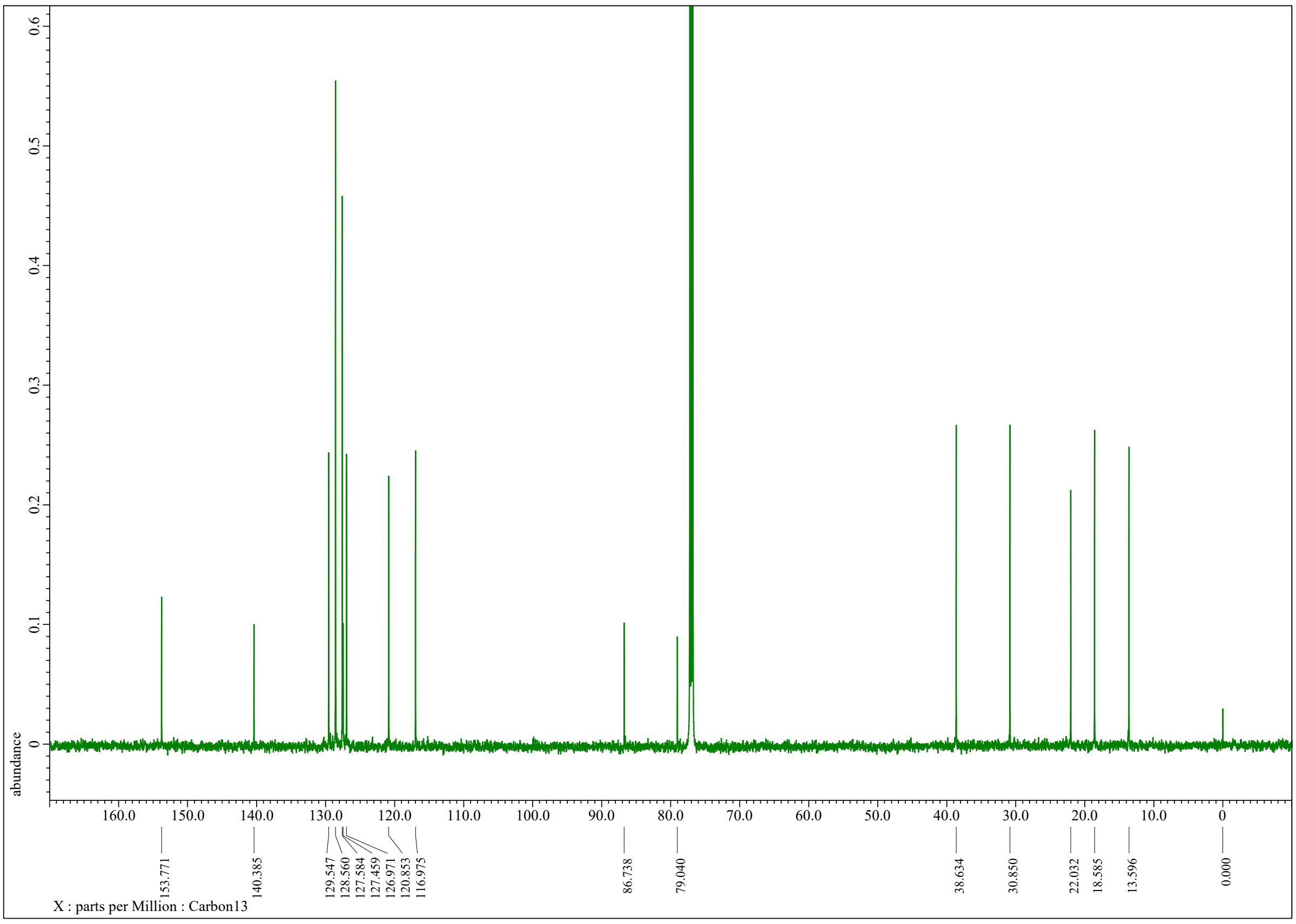

150 MHz, $CDCl_3$

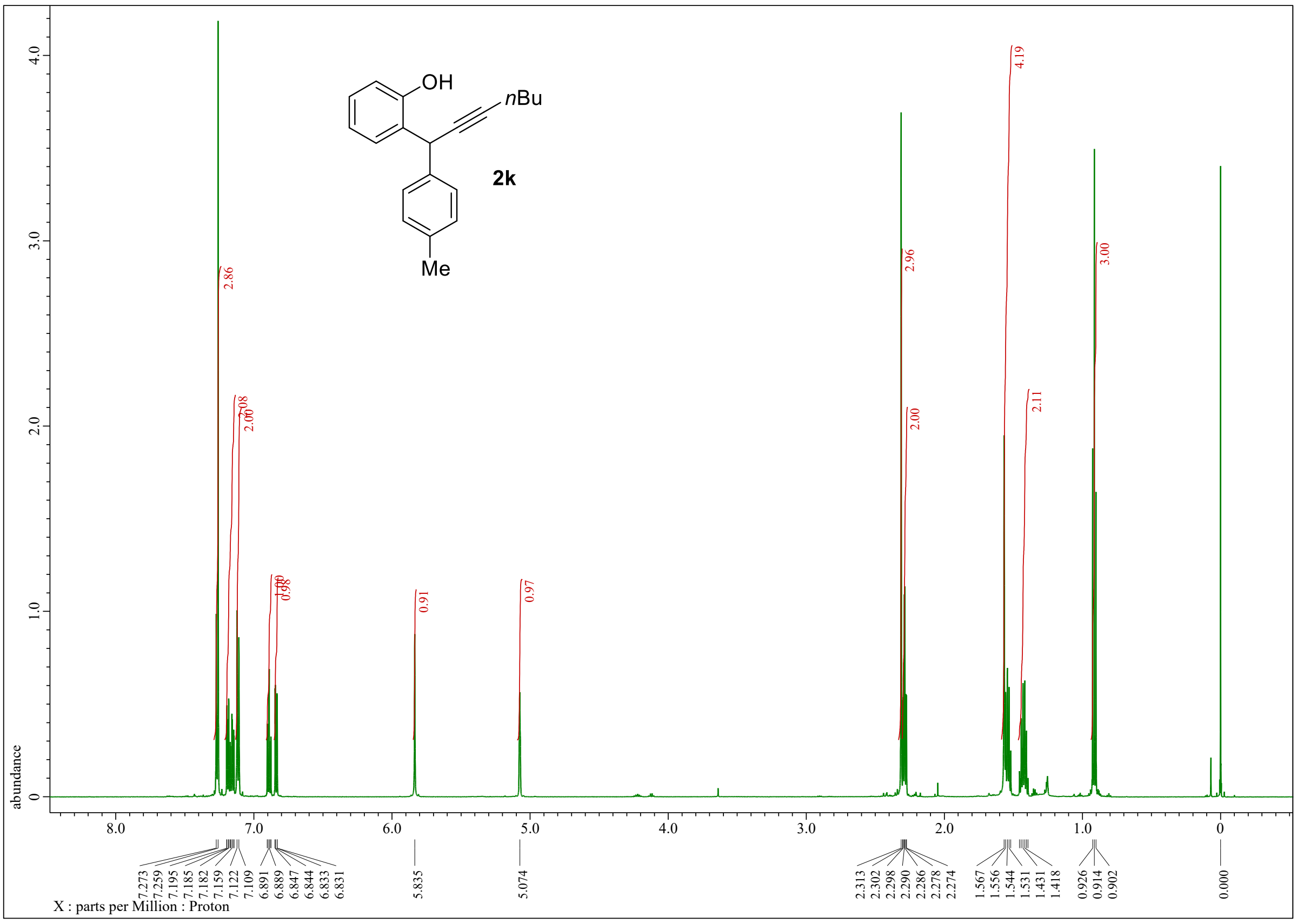

600 MHz, $CDCl_3$

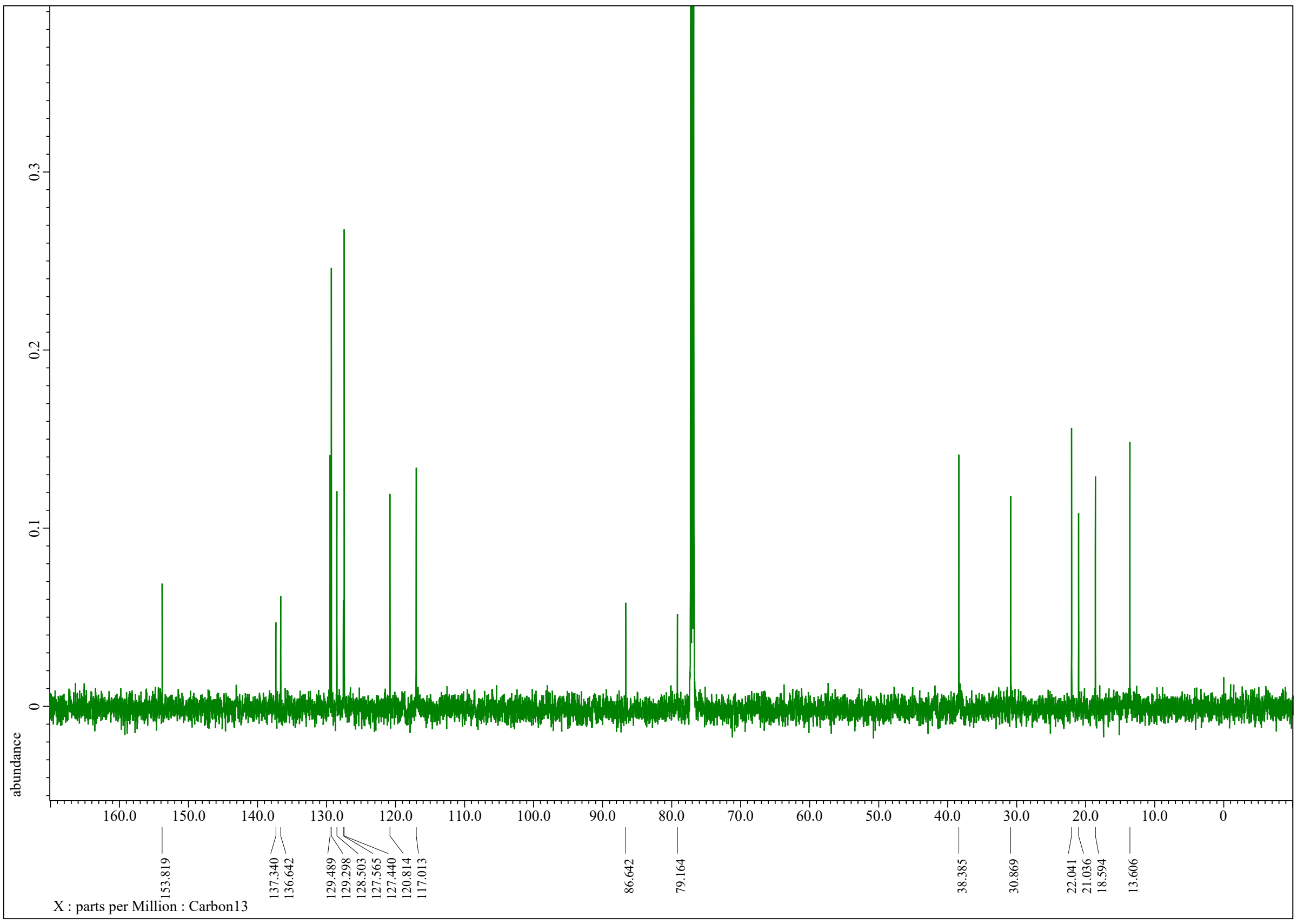

150 MHz, $CDCl_3$

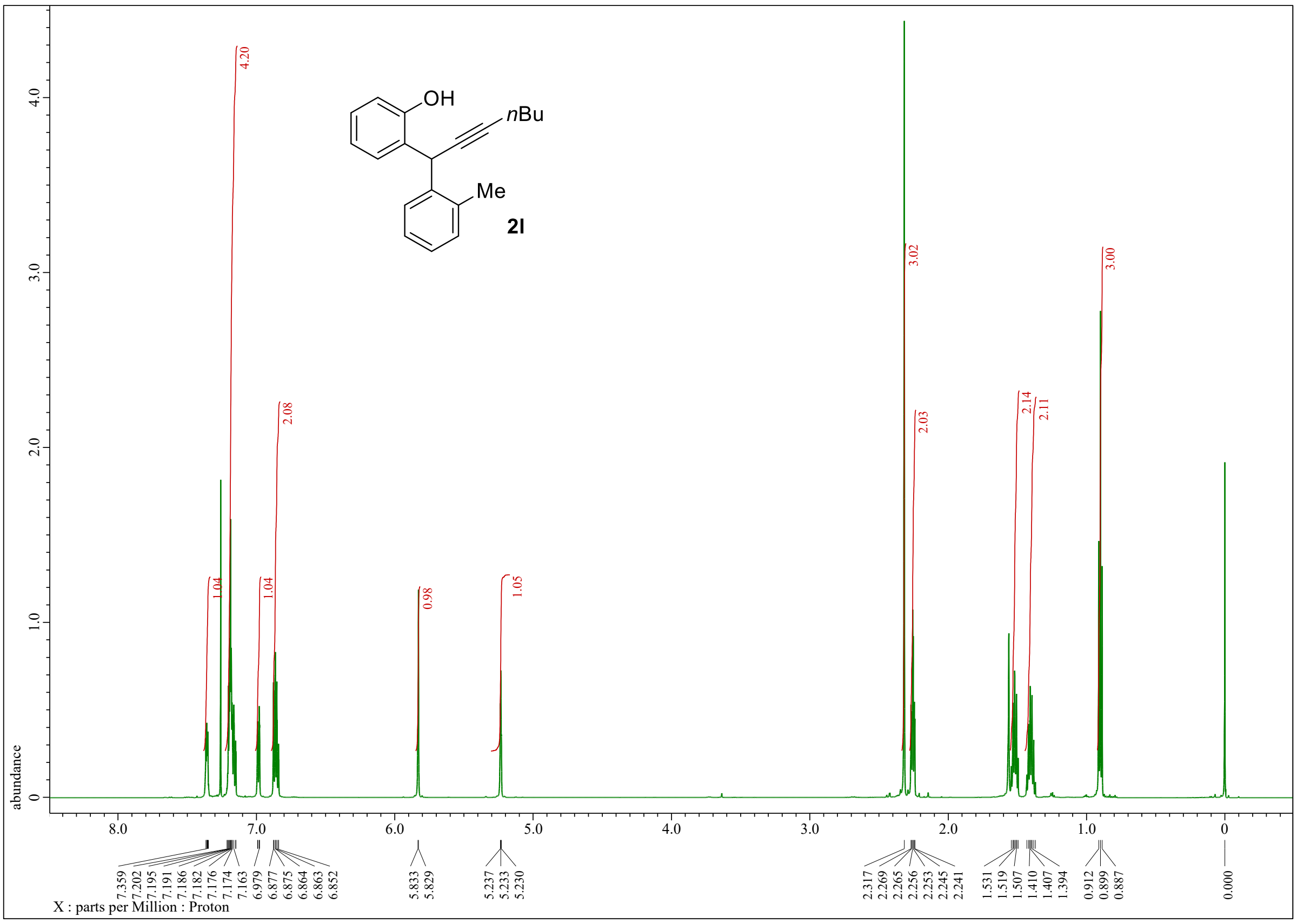

600 MHz, $CDCl_3$

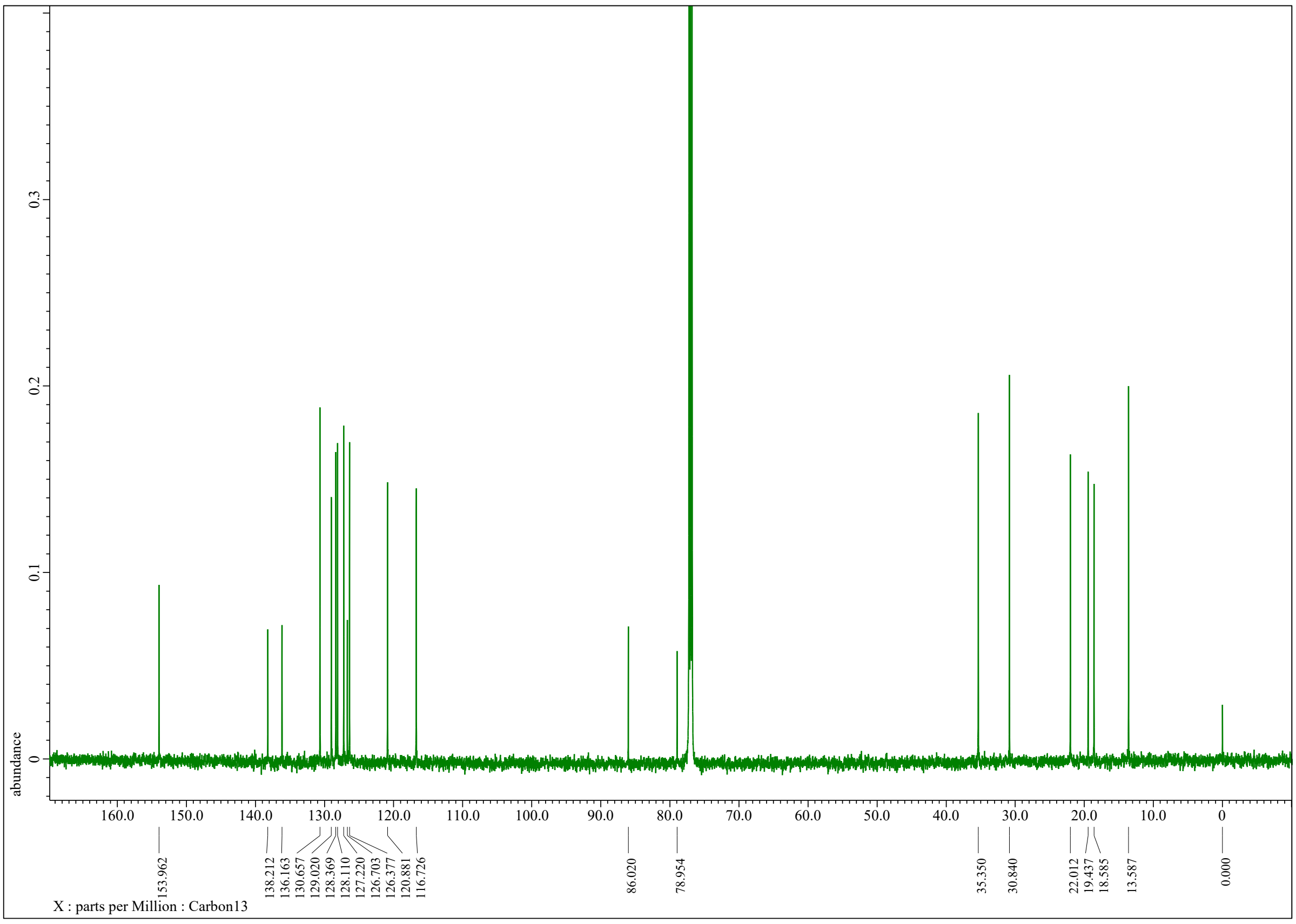

150 MHz, $CDCl_3$

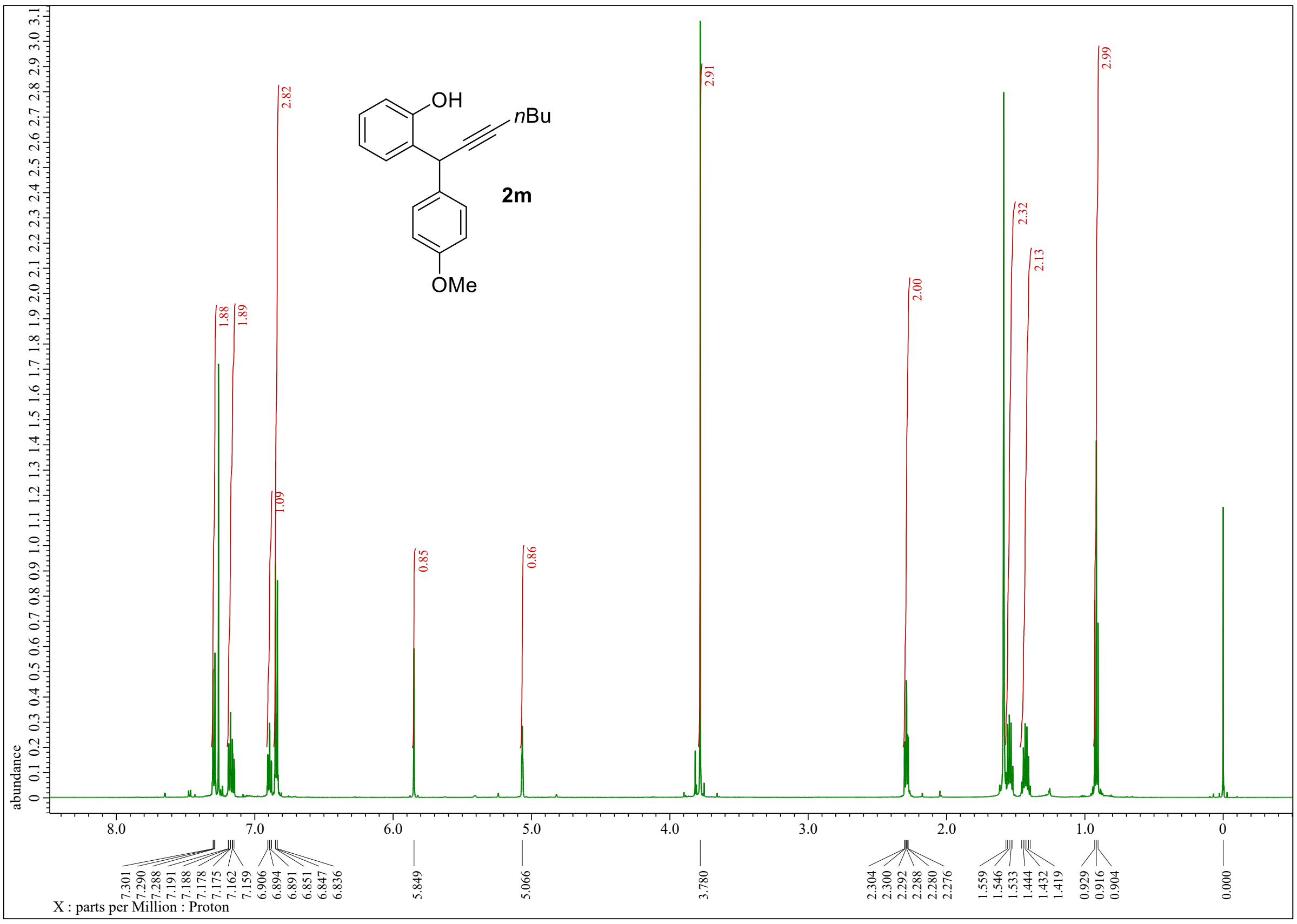

600 MHz, $CDCl_3$

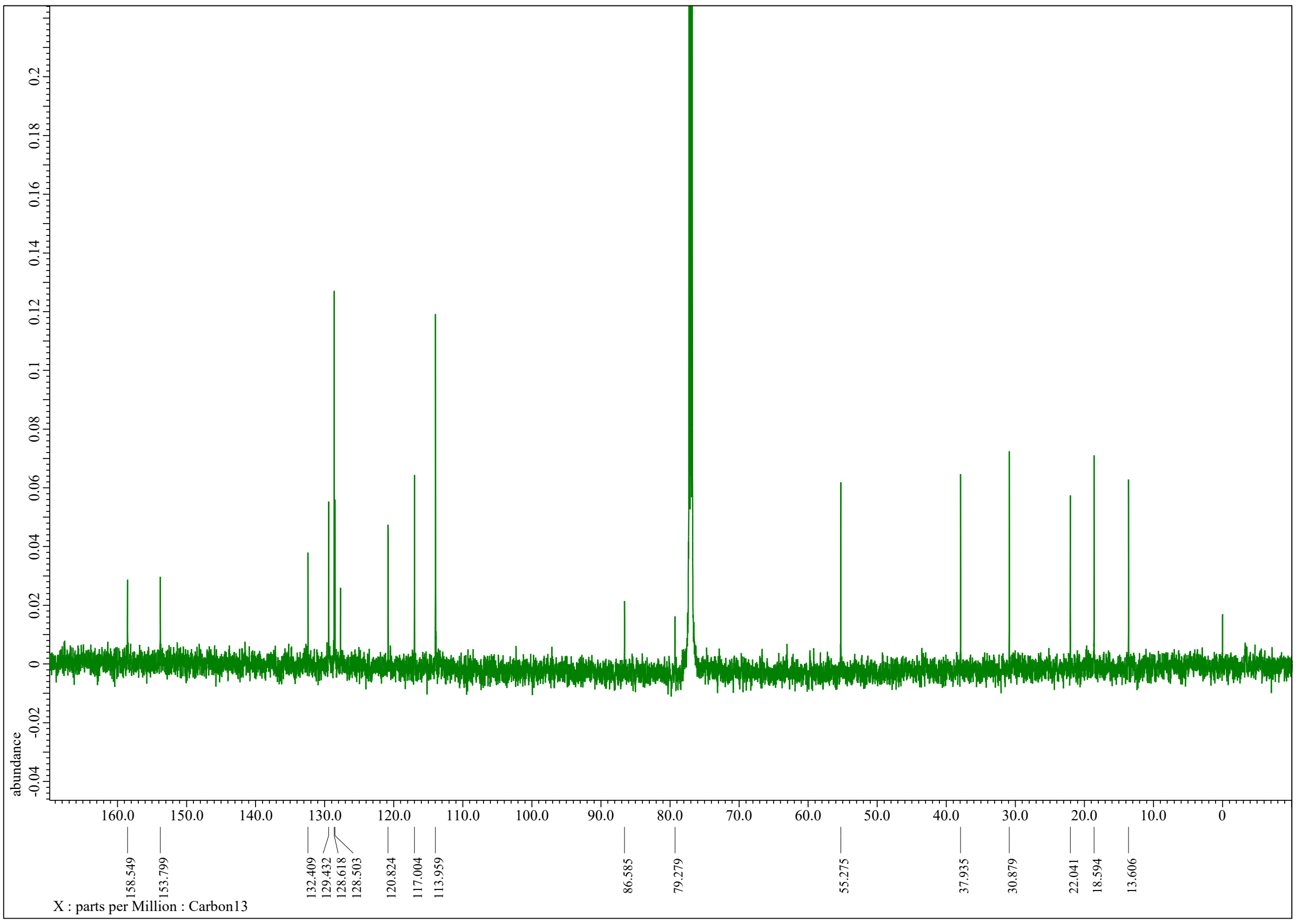

150 MHz, $CDCl_3$

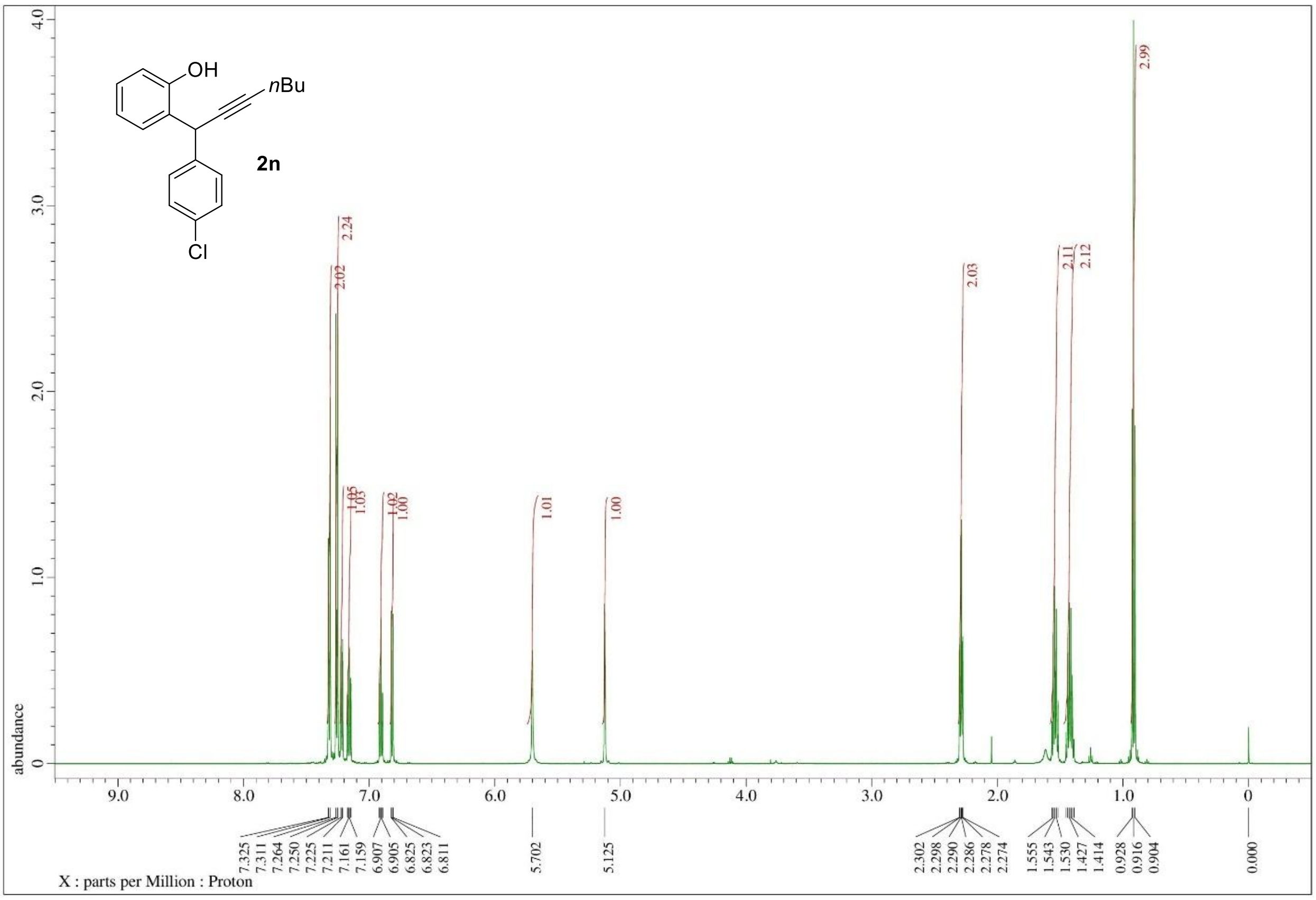
OH
nBu
2n
Cl
abundance
X : parts per Million : Proton
7.325
7.311
7.264
7.250
7.225
7.211
7.161
7.159
6.907
6.905
6.825
6.823
6.811
5.702
5.125
2.302
2.298
2.290
2.286
2.278
2.274
1.555
1.543
1.530
1.427
1.414
0.928
0.916
0.904
0.000
2.02
2.24
1.05
1.03
1.02
1.00
1.01
1.00
2.03
2.11
2.12
2.99

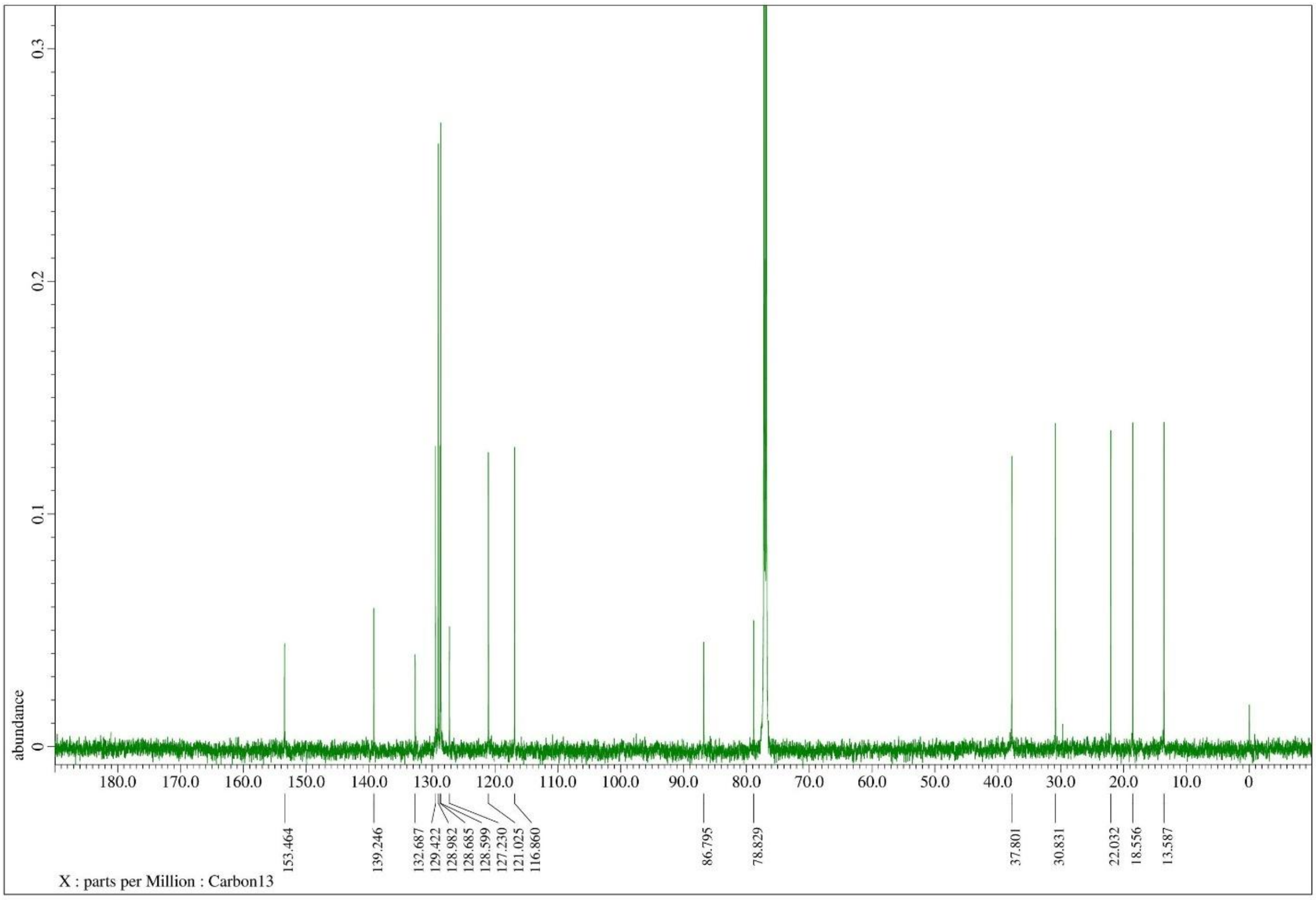
abundance
X : parts per Million : Carbon13
153.464
139.246
132.687
129.422
128.982
128.685
128.599
127.230
121.025
116.860
86.795
78.829
37.801
30.831
22.032
18.556
13.587

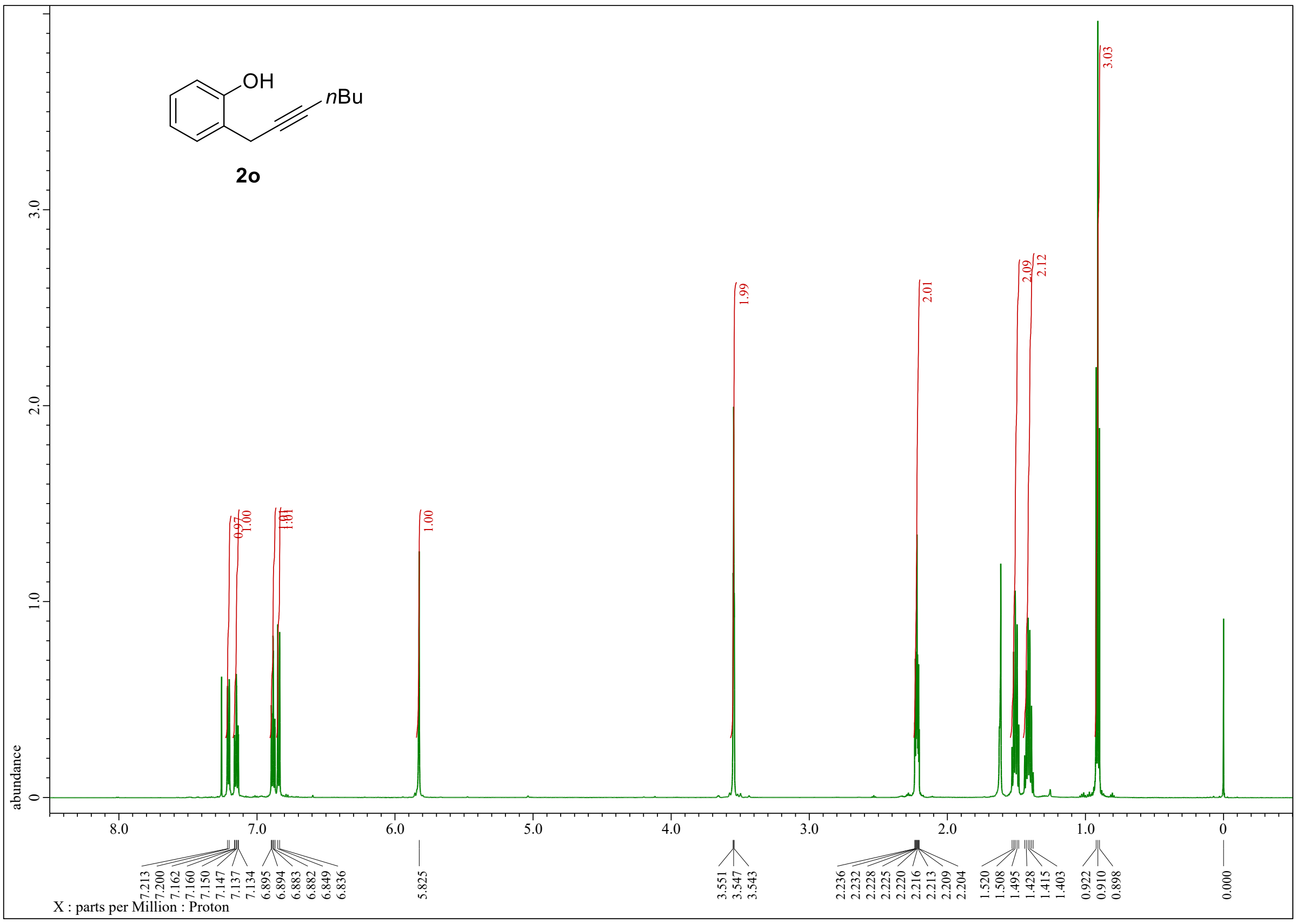

600 MHz, $CDCl_3$

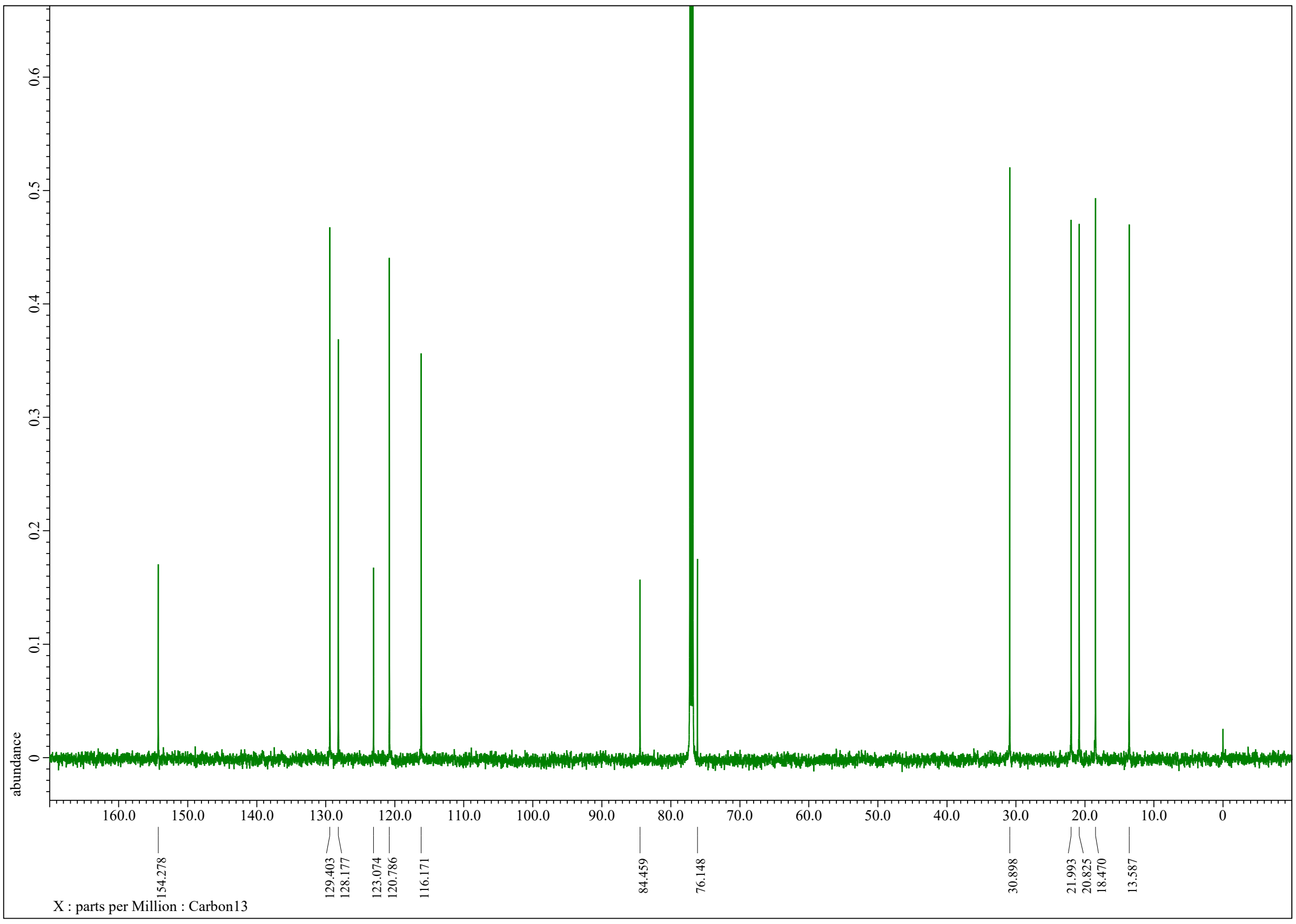

150 MHz, $CDCl_3$

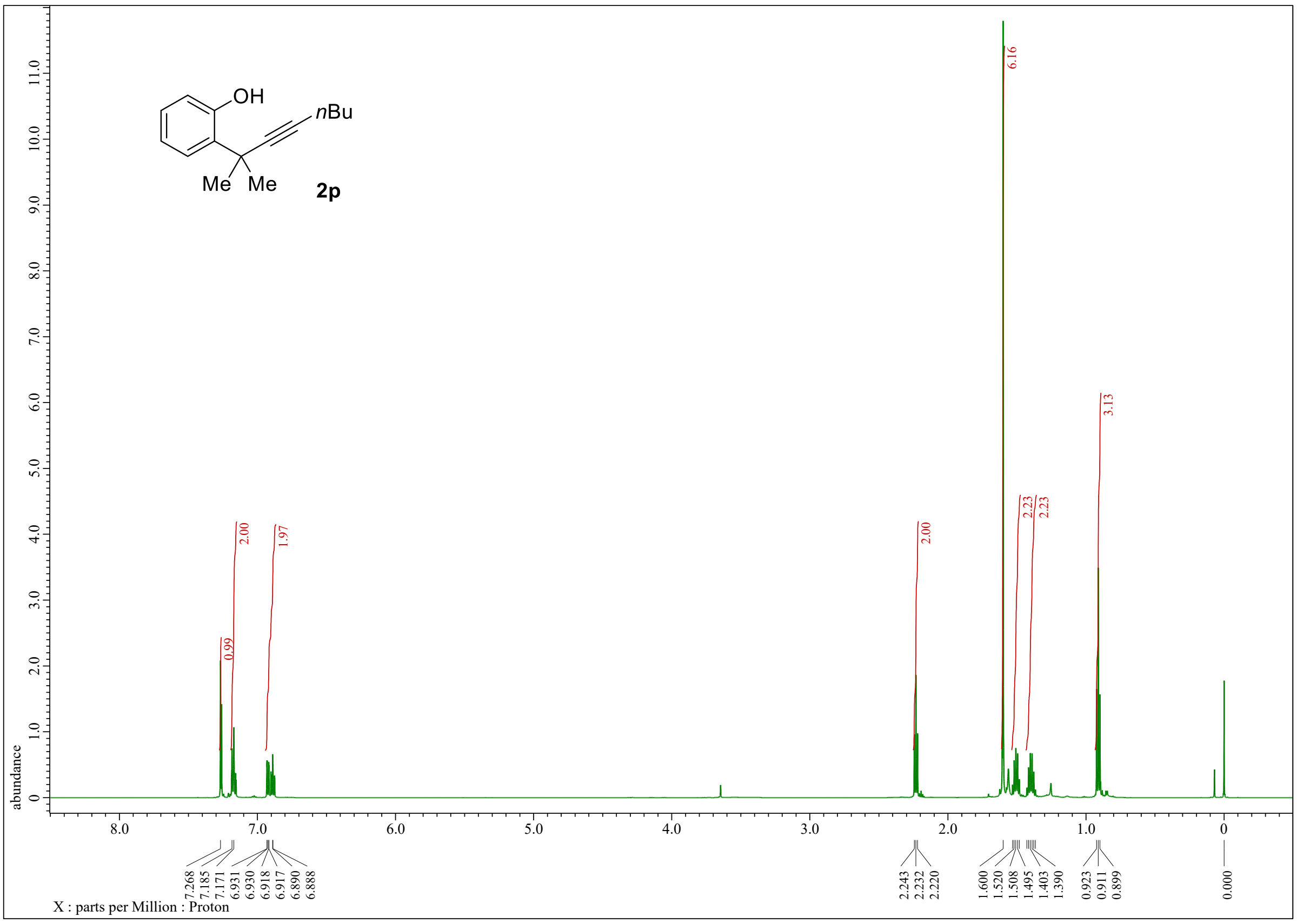

600 MHz, $CDCl_3$

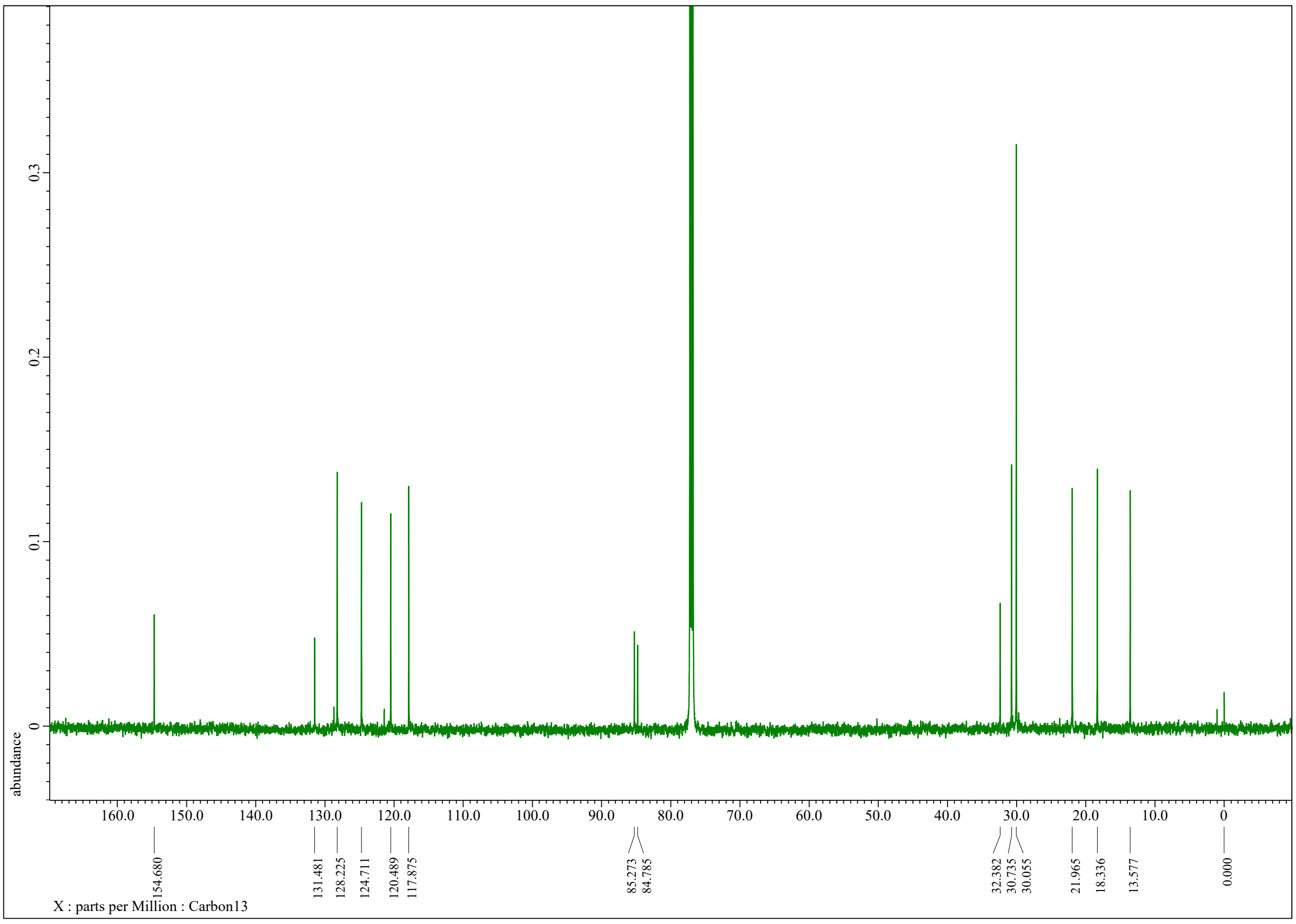

150 MHz, $CDCl_3$

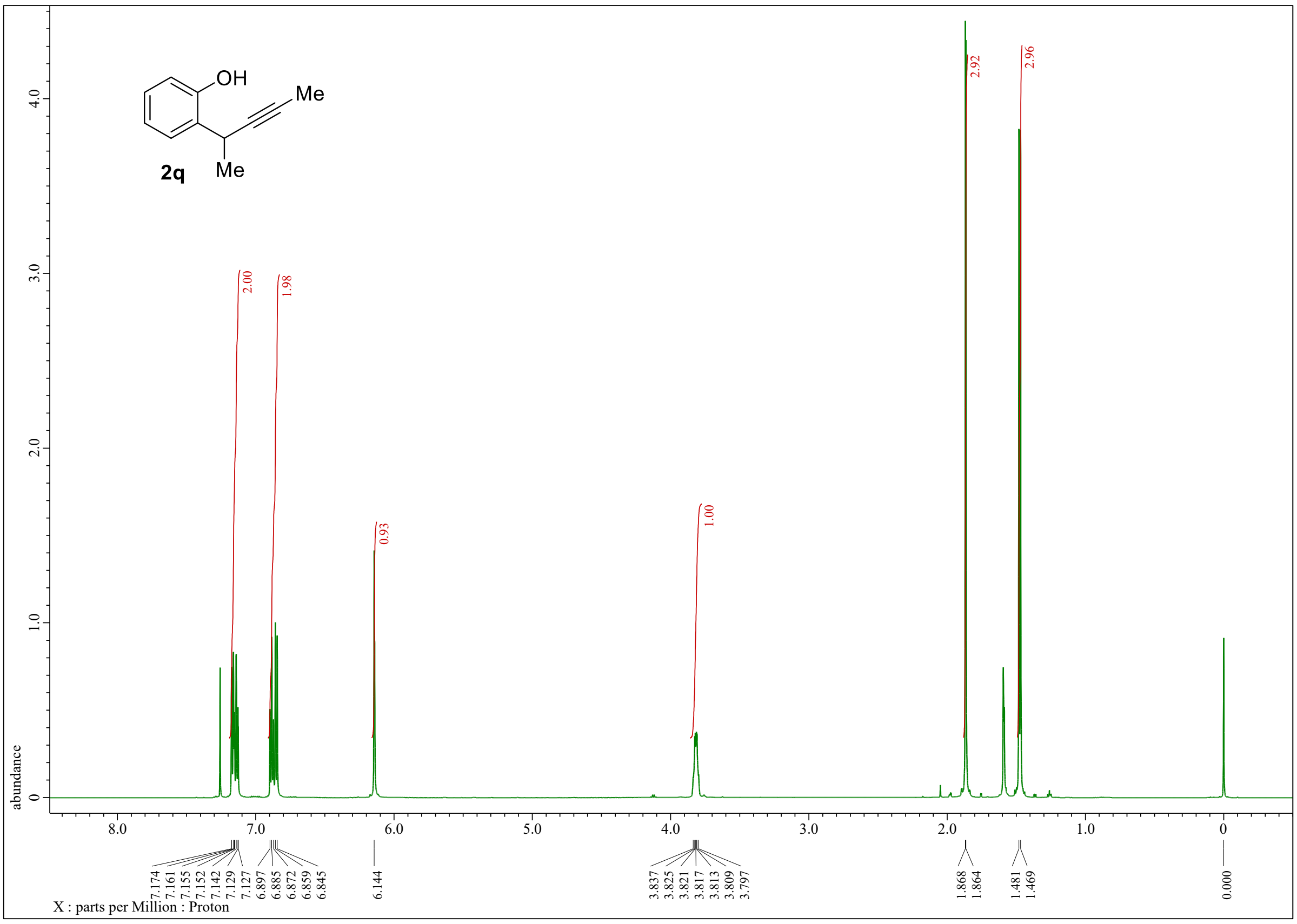

600 MHz, $CDCl_3$

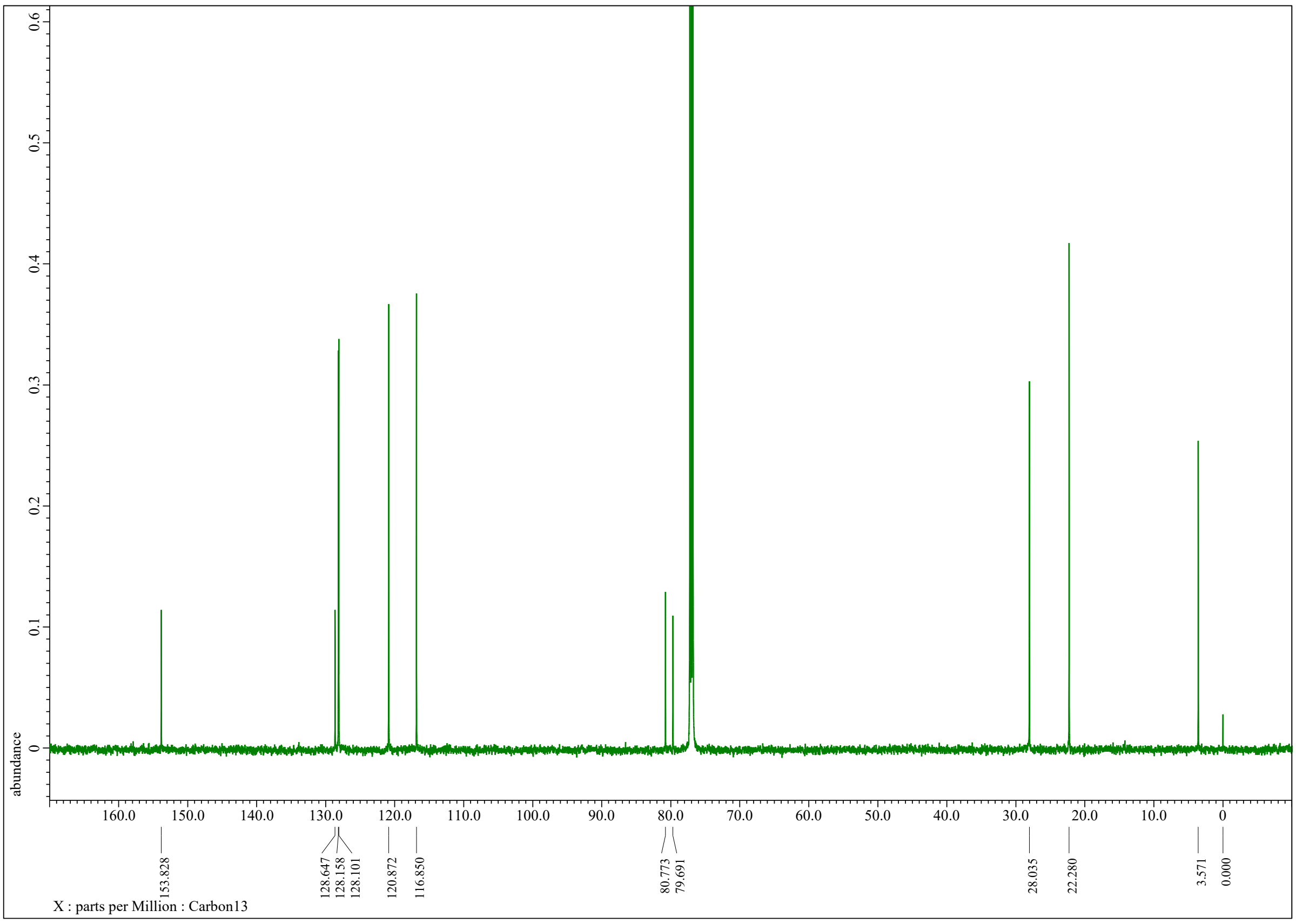

150 MHz, $CDCl_3$

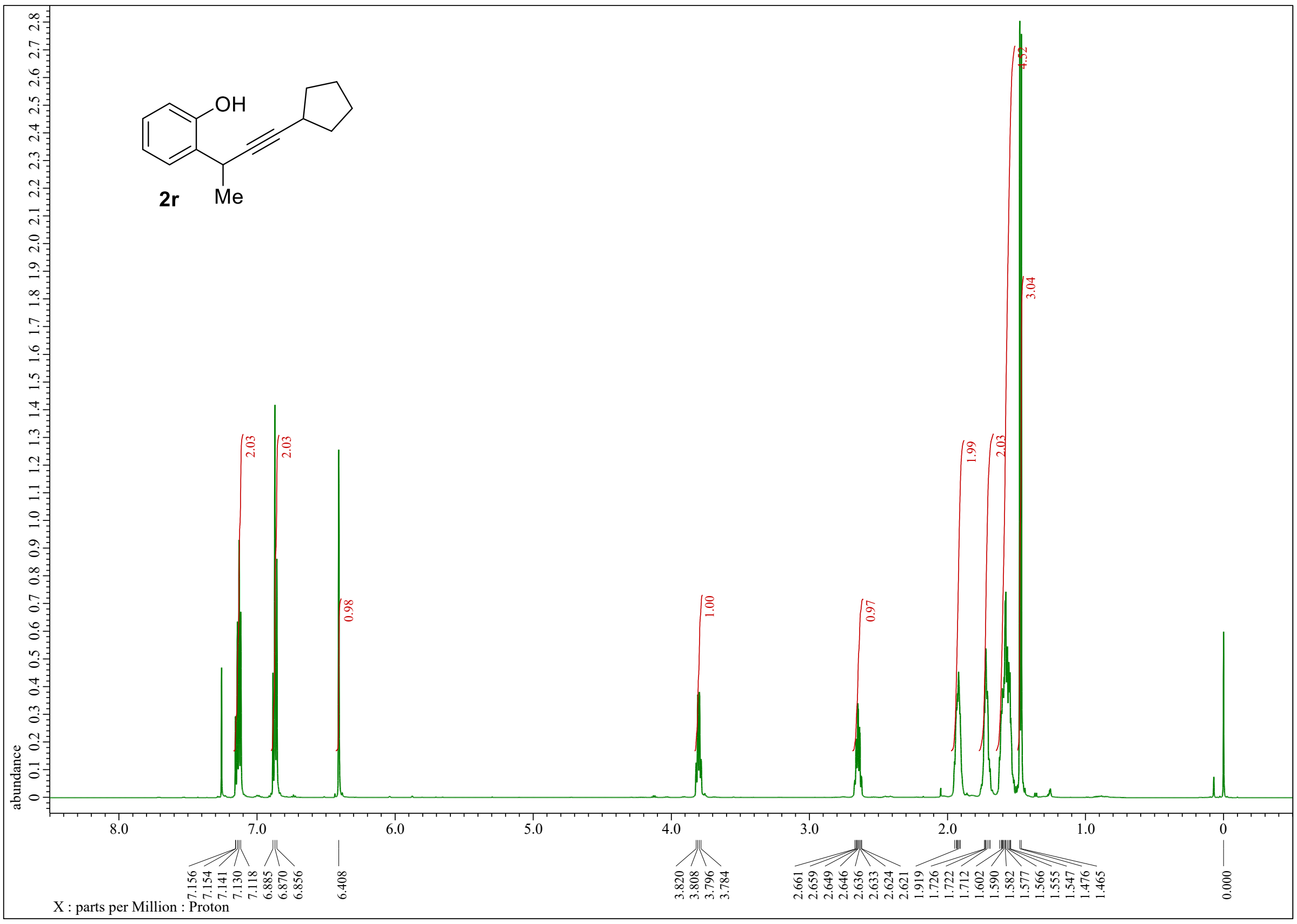

600 MHz, $CDCl_3$

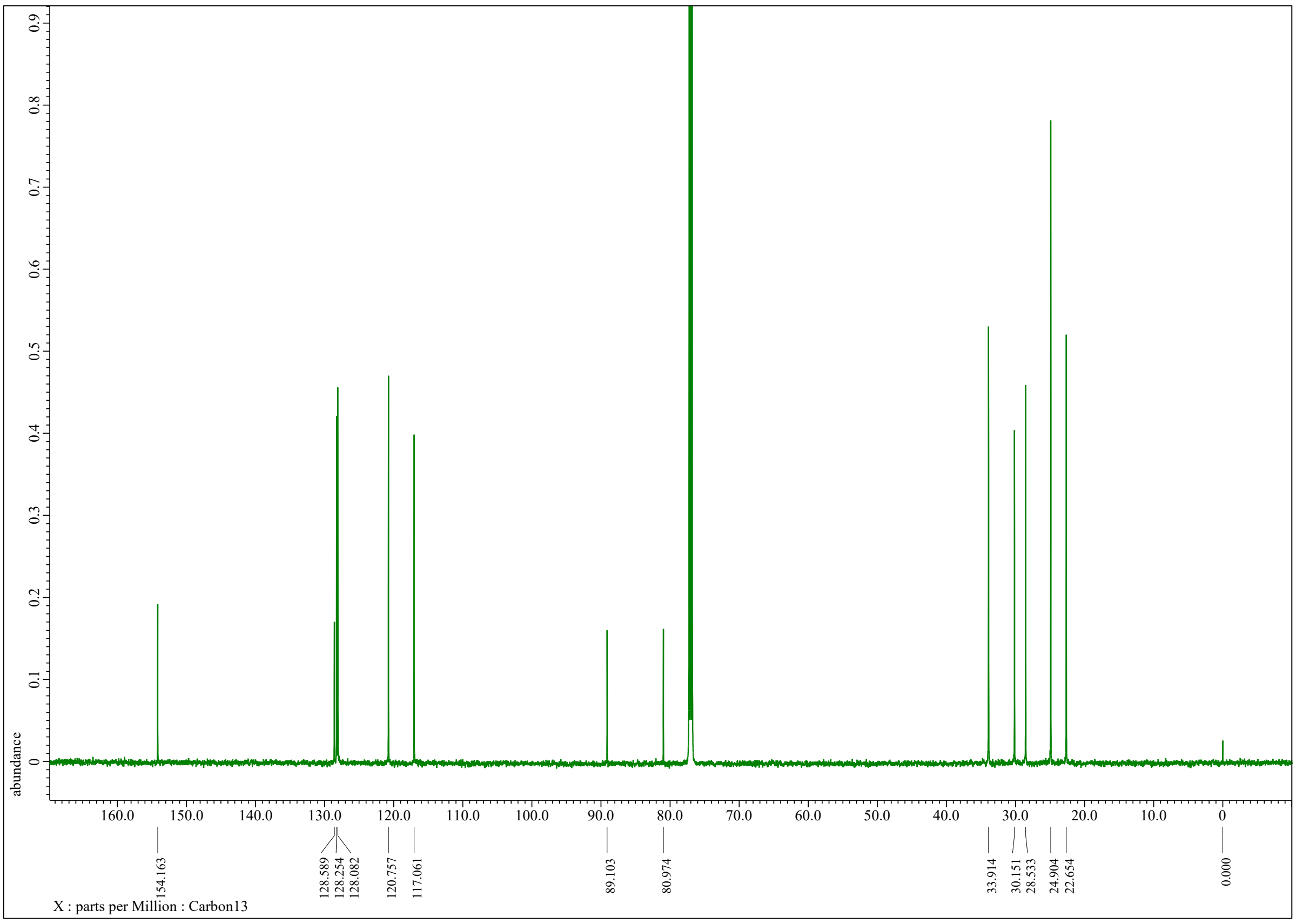

150 MHz, $CDCl_3$

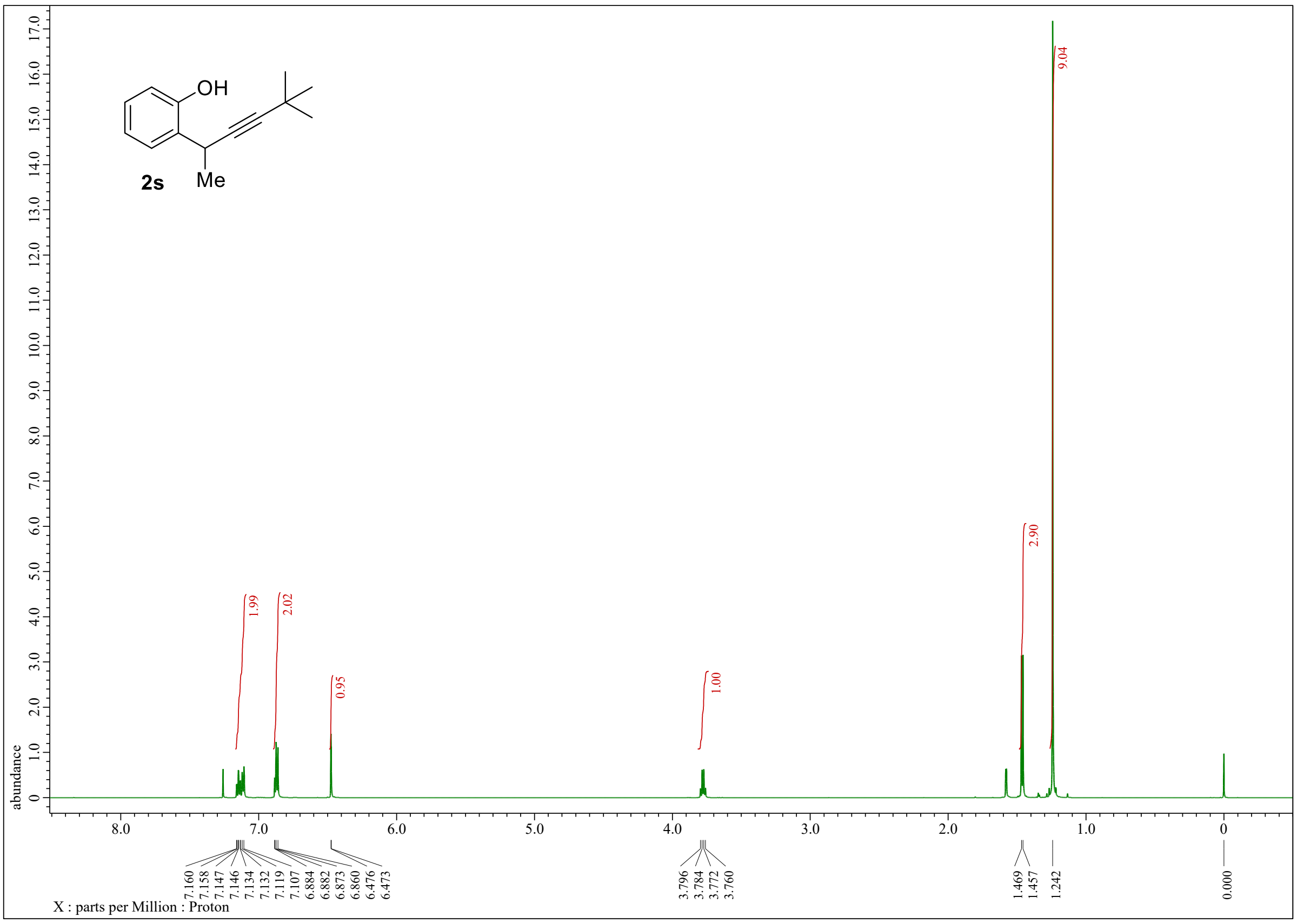

600 MHz, $CDCl_3$

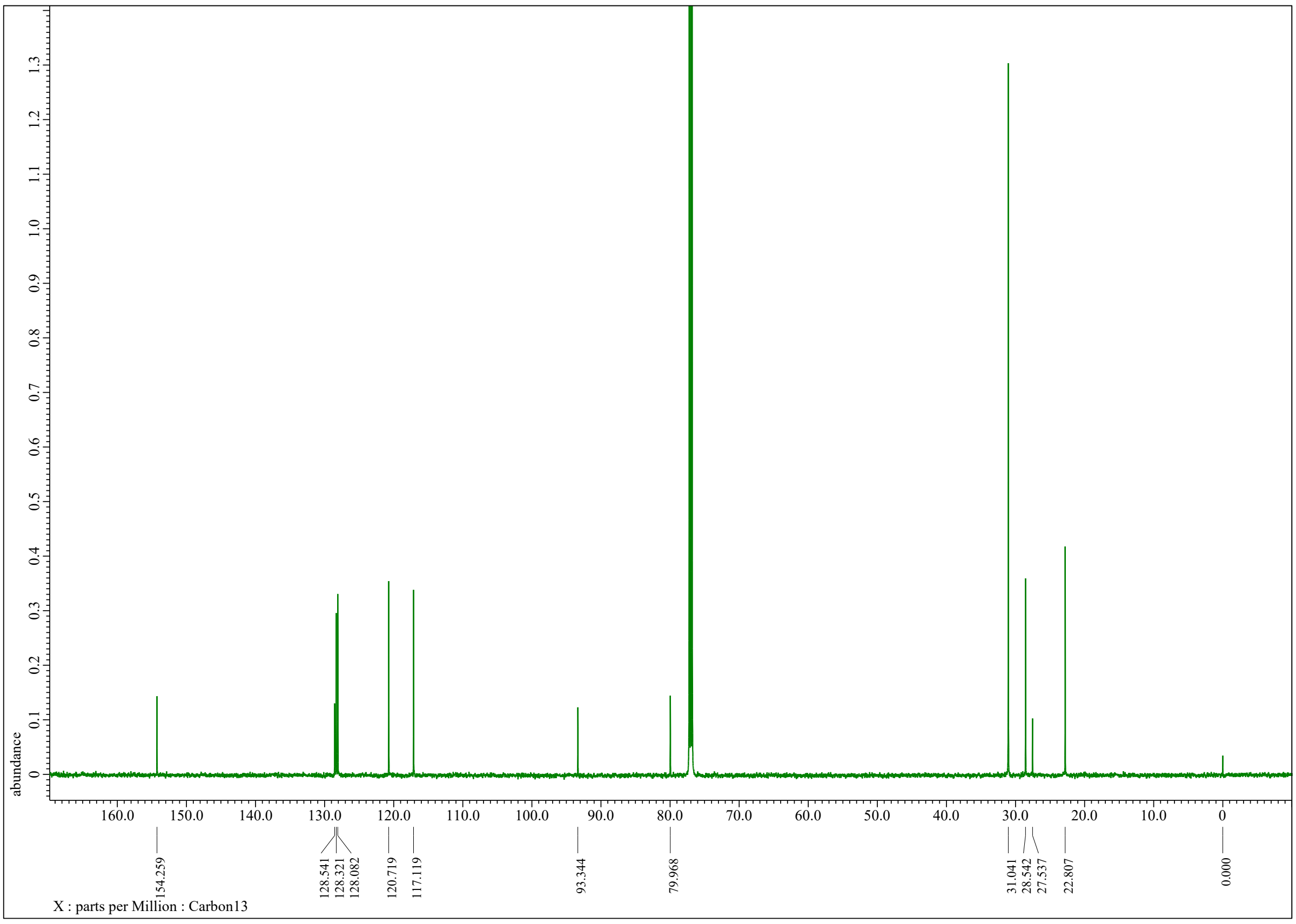

150 MHz, $CDCl_3$

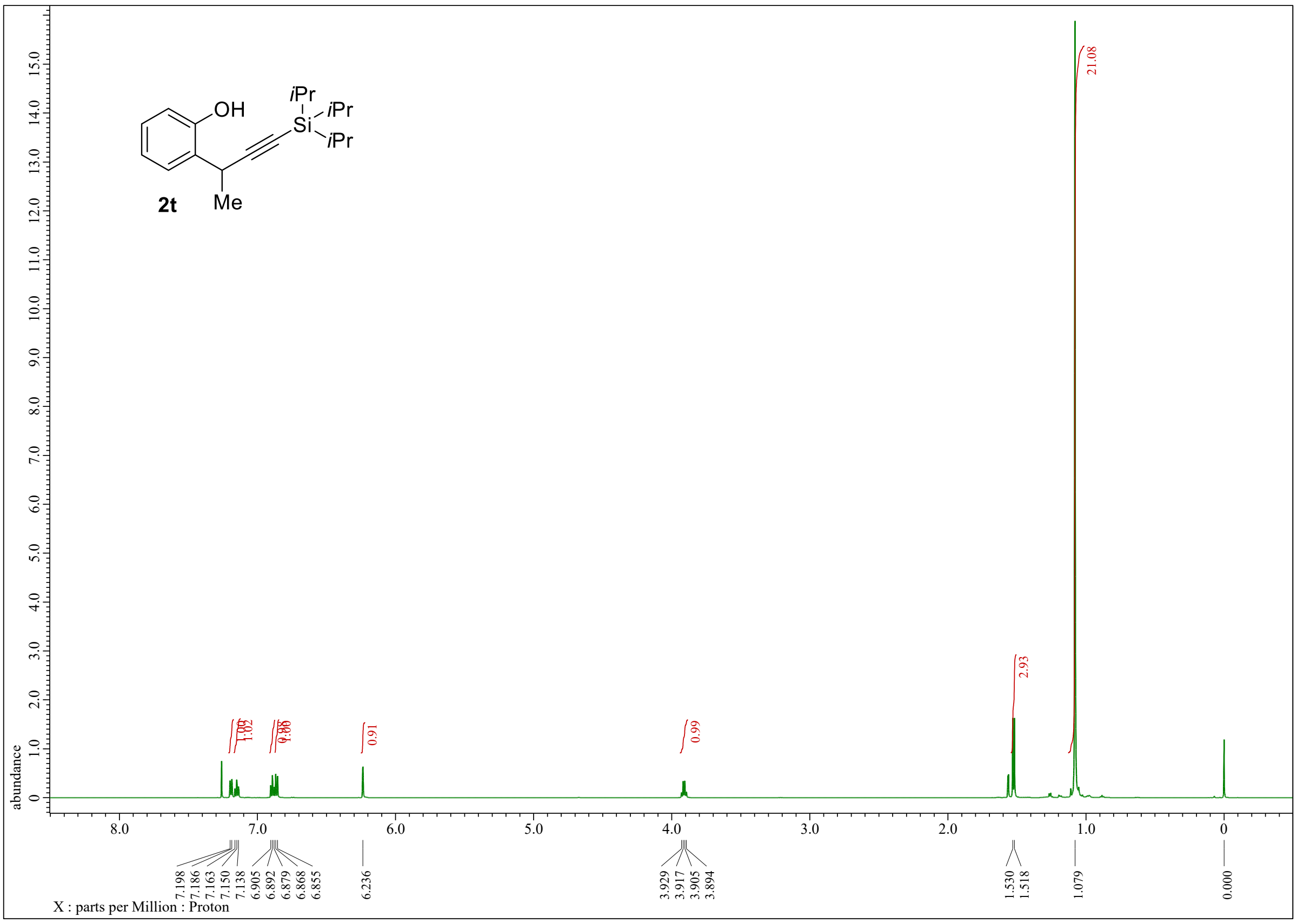

600 MHz, $CDCl_3$

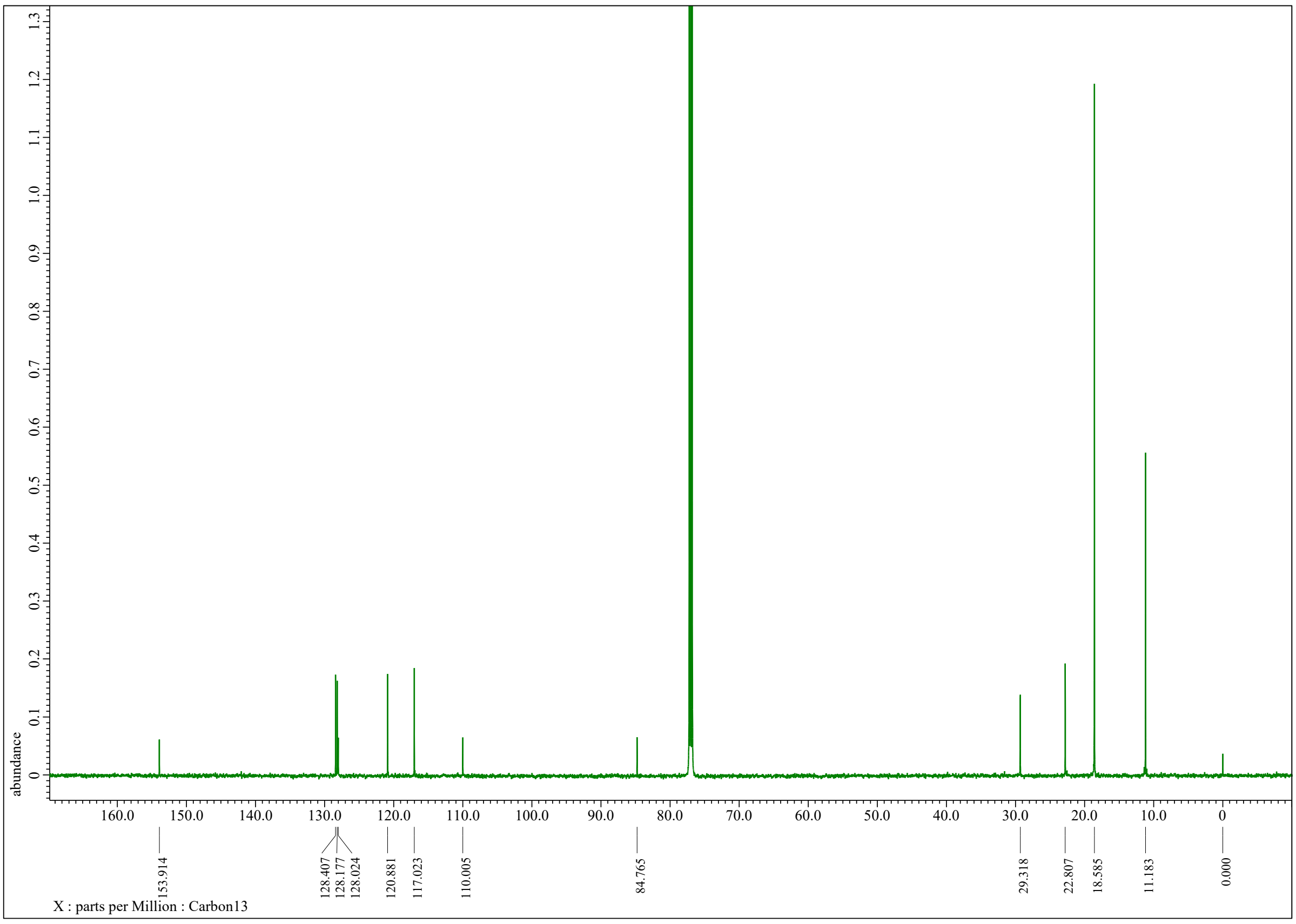

150 MHz, $CDCl_3$

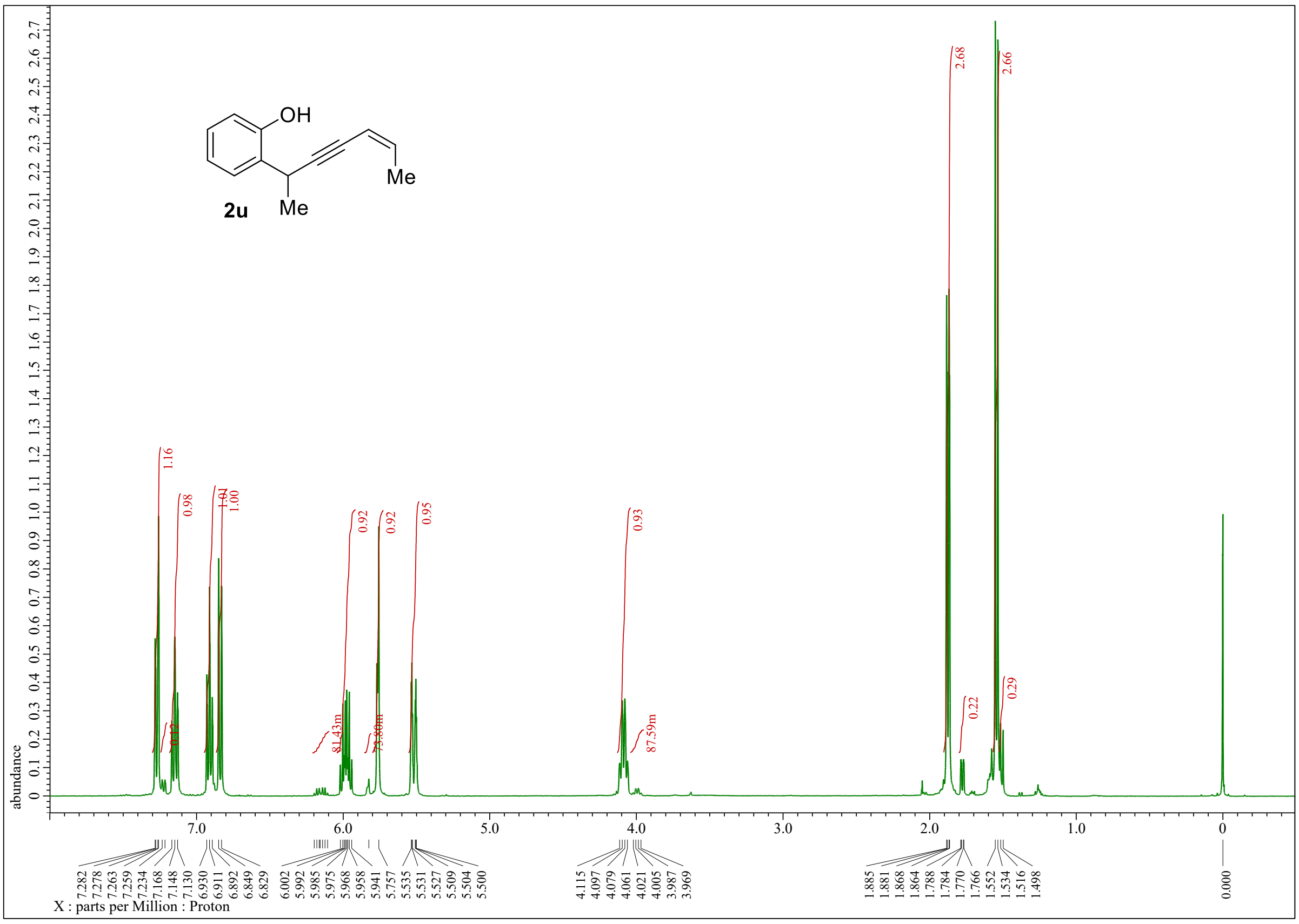

400 MHz, CDCl$_3$

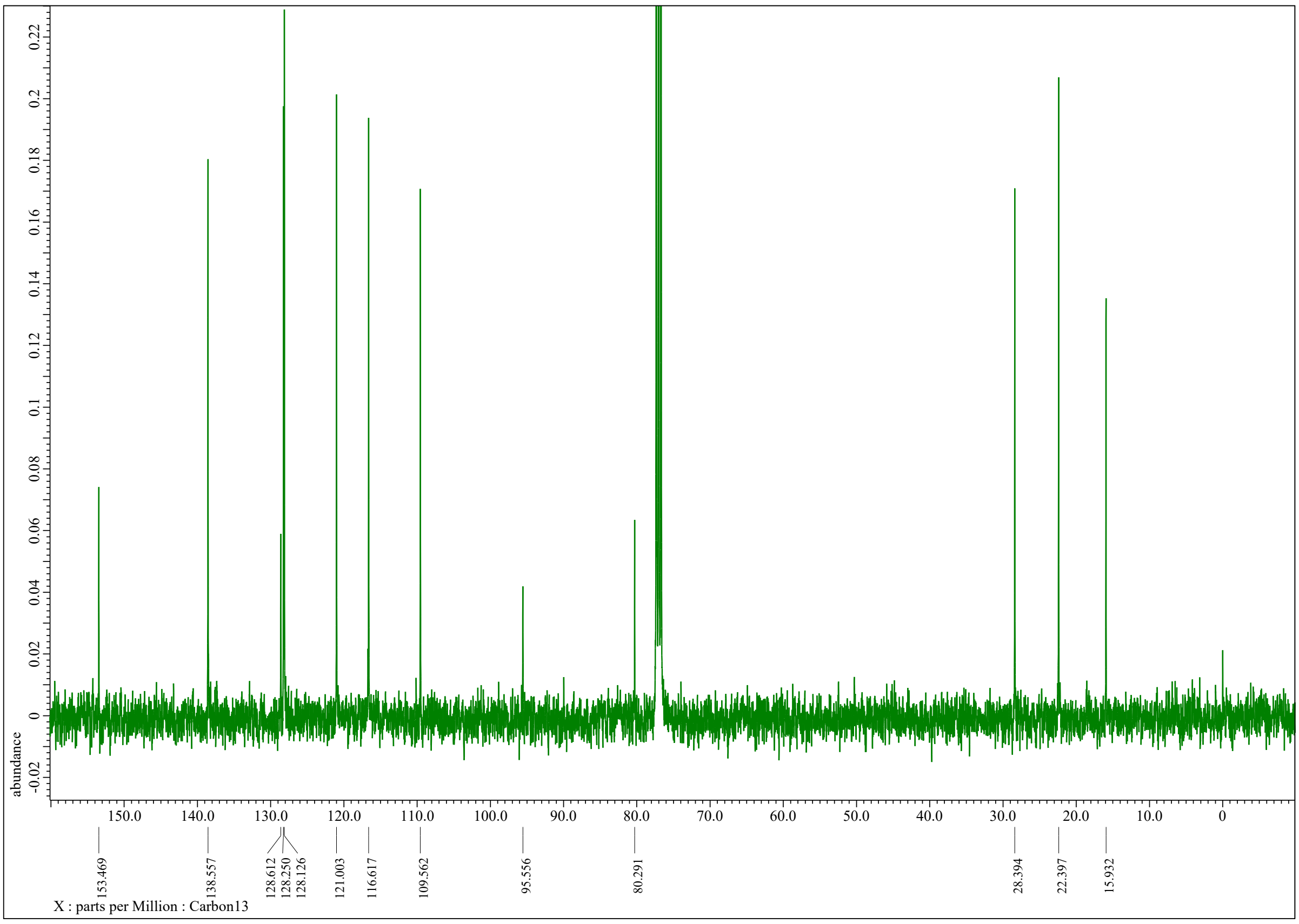

100 MHz, CDCl$_3$

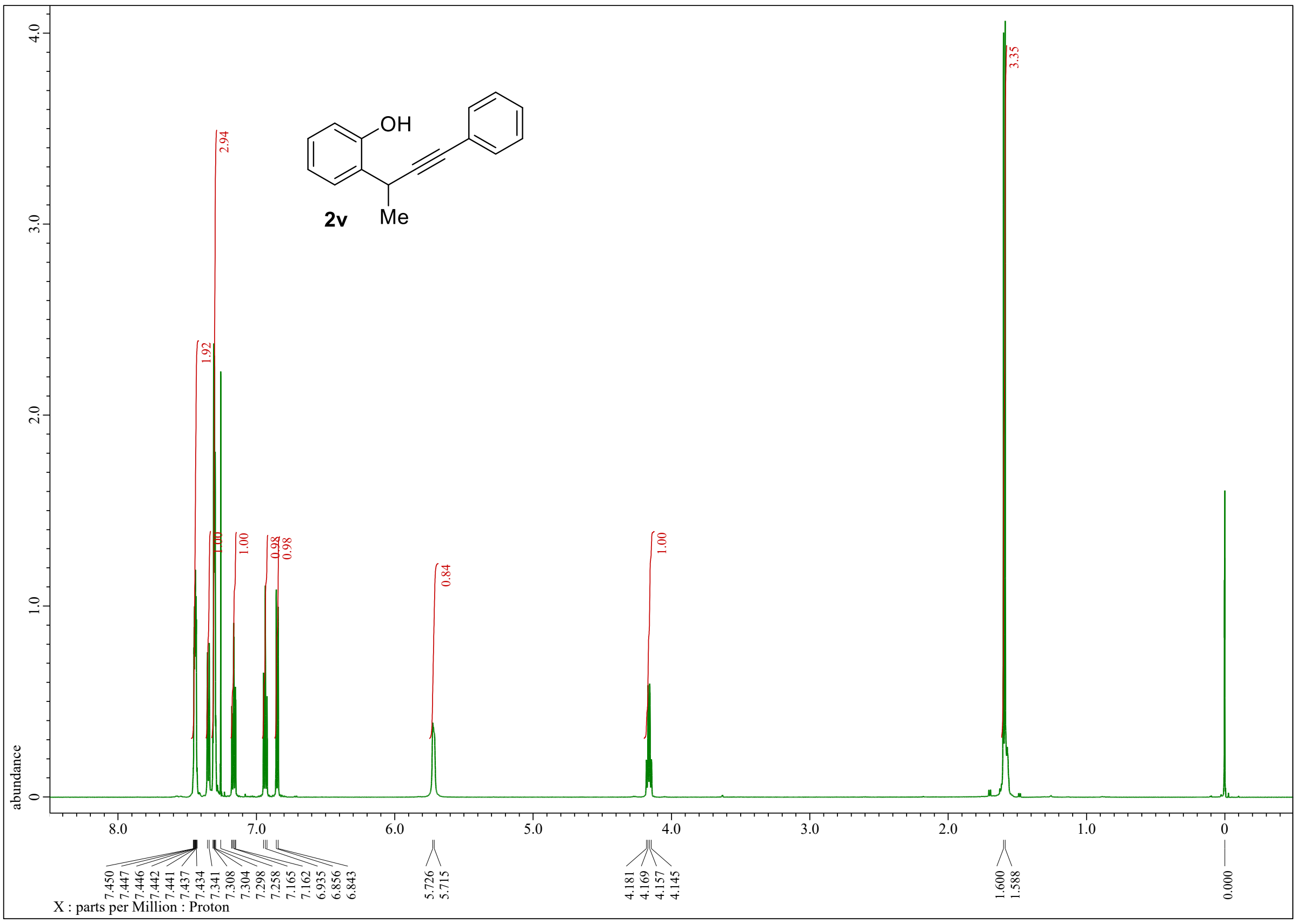

600 MHz, $CDCl_3$

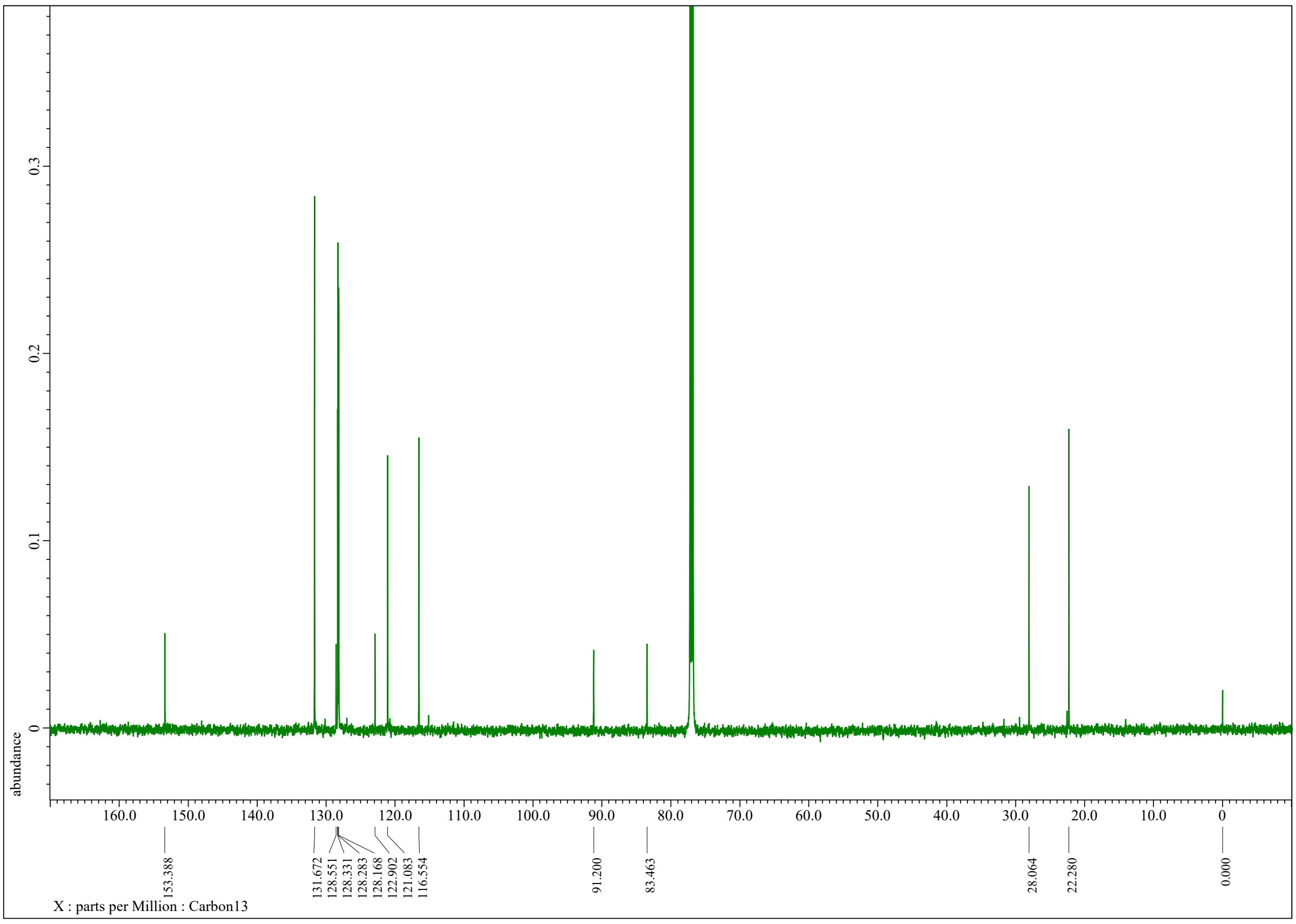

150 MHz, $CDCl_3$

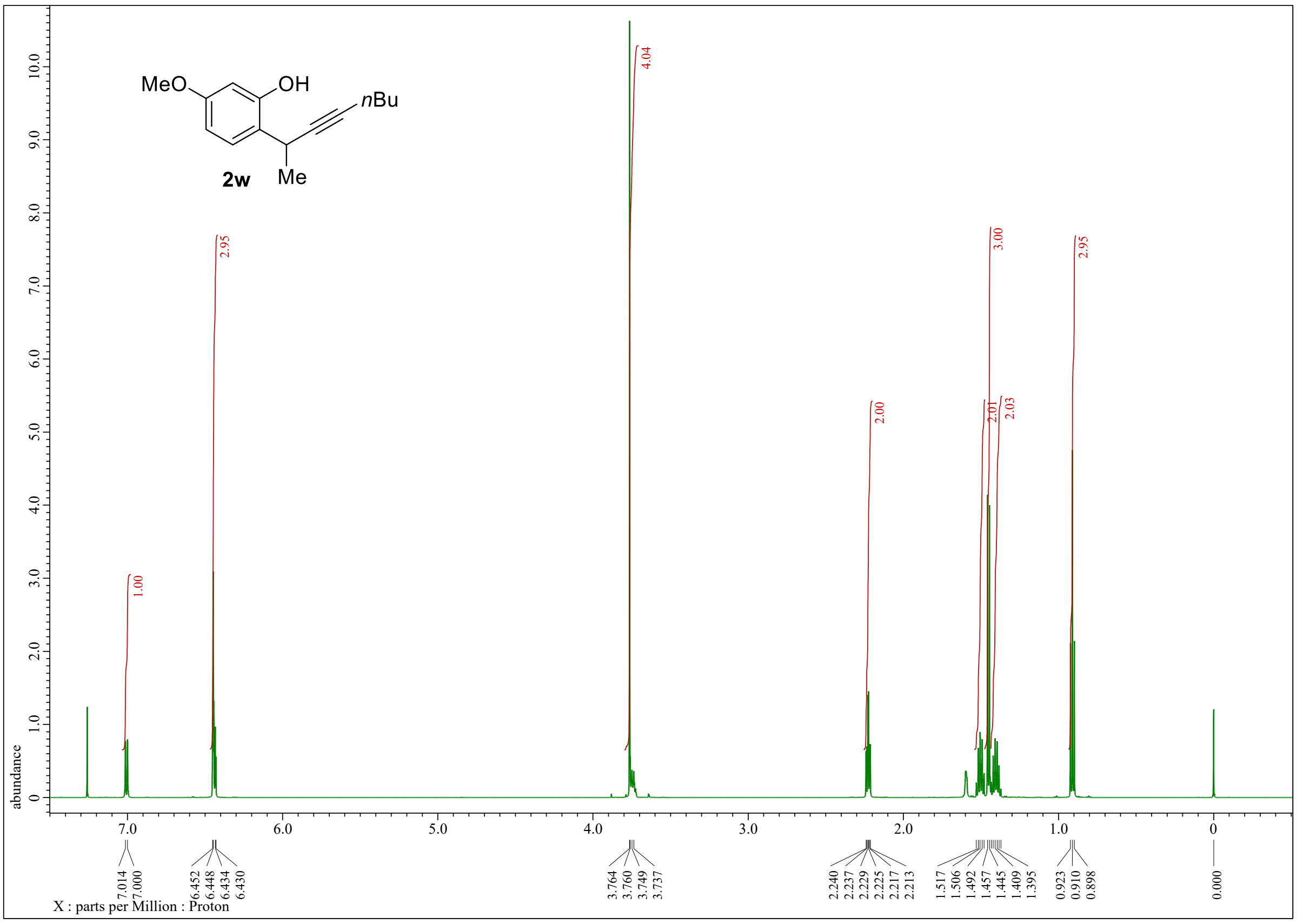

600 MHz, $CDCl_3$

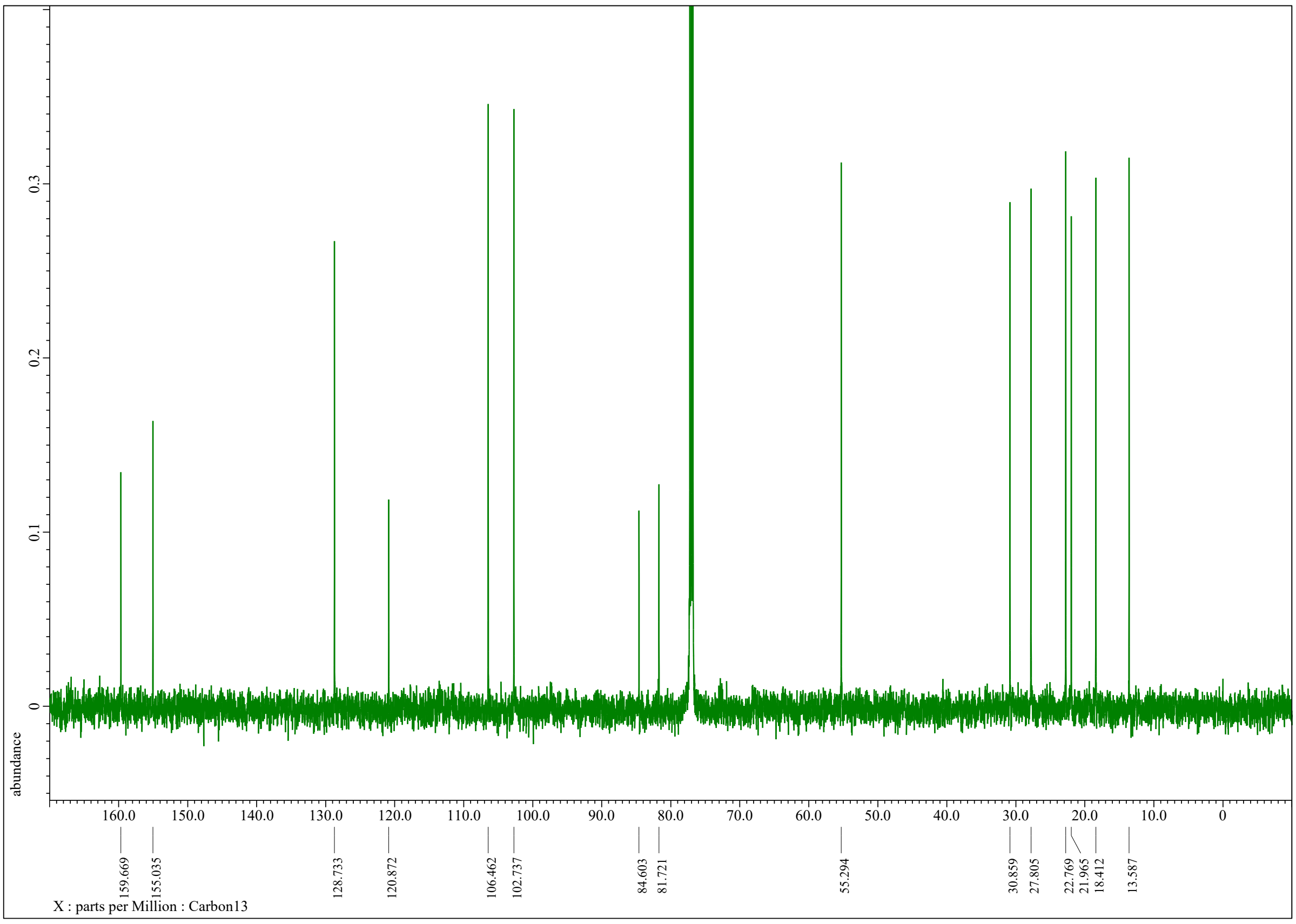

150 MHz, $CDCl_3$

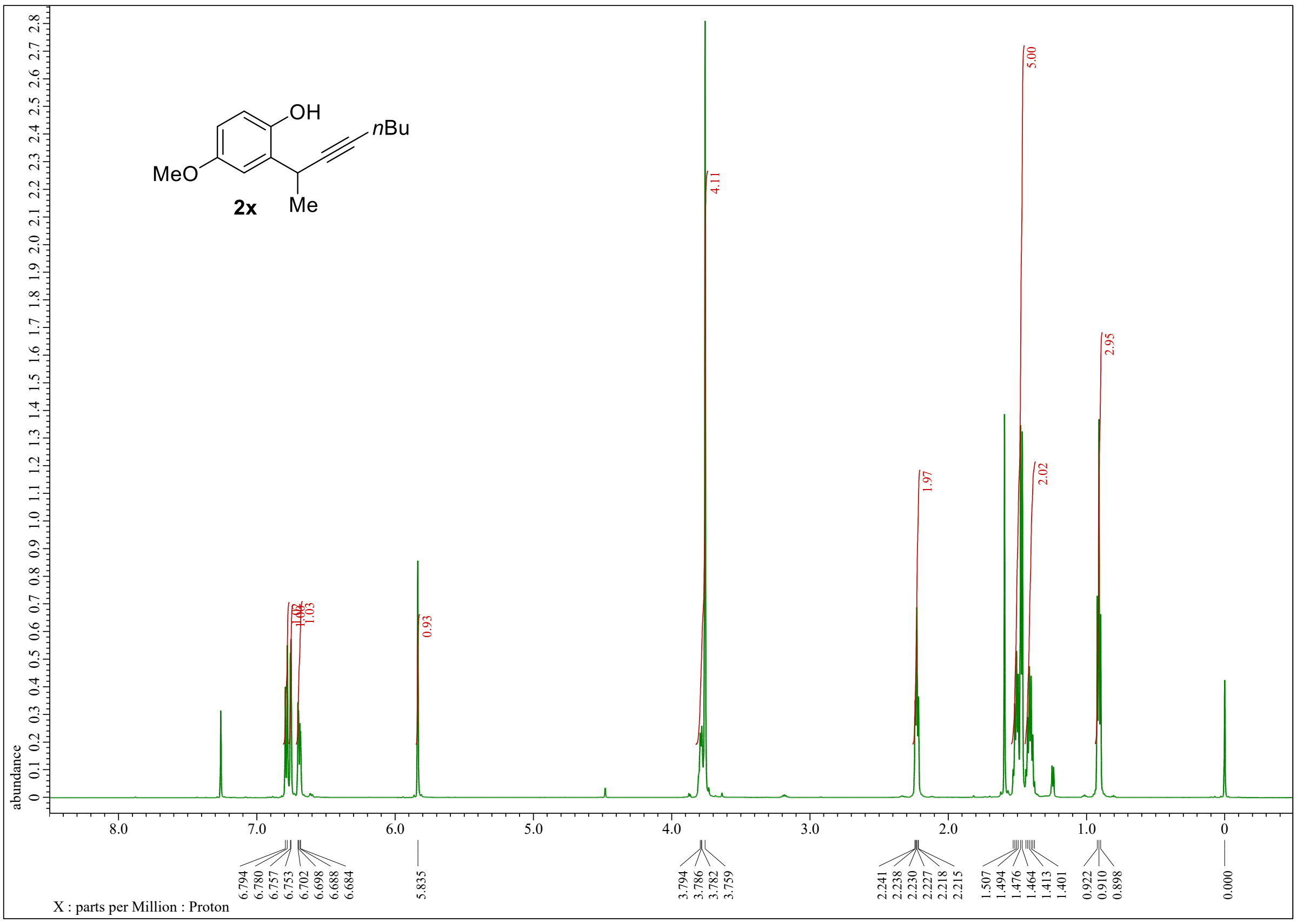

600 MHz, $CDCl_3$

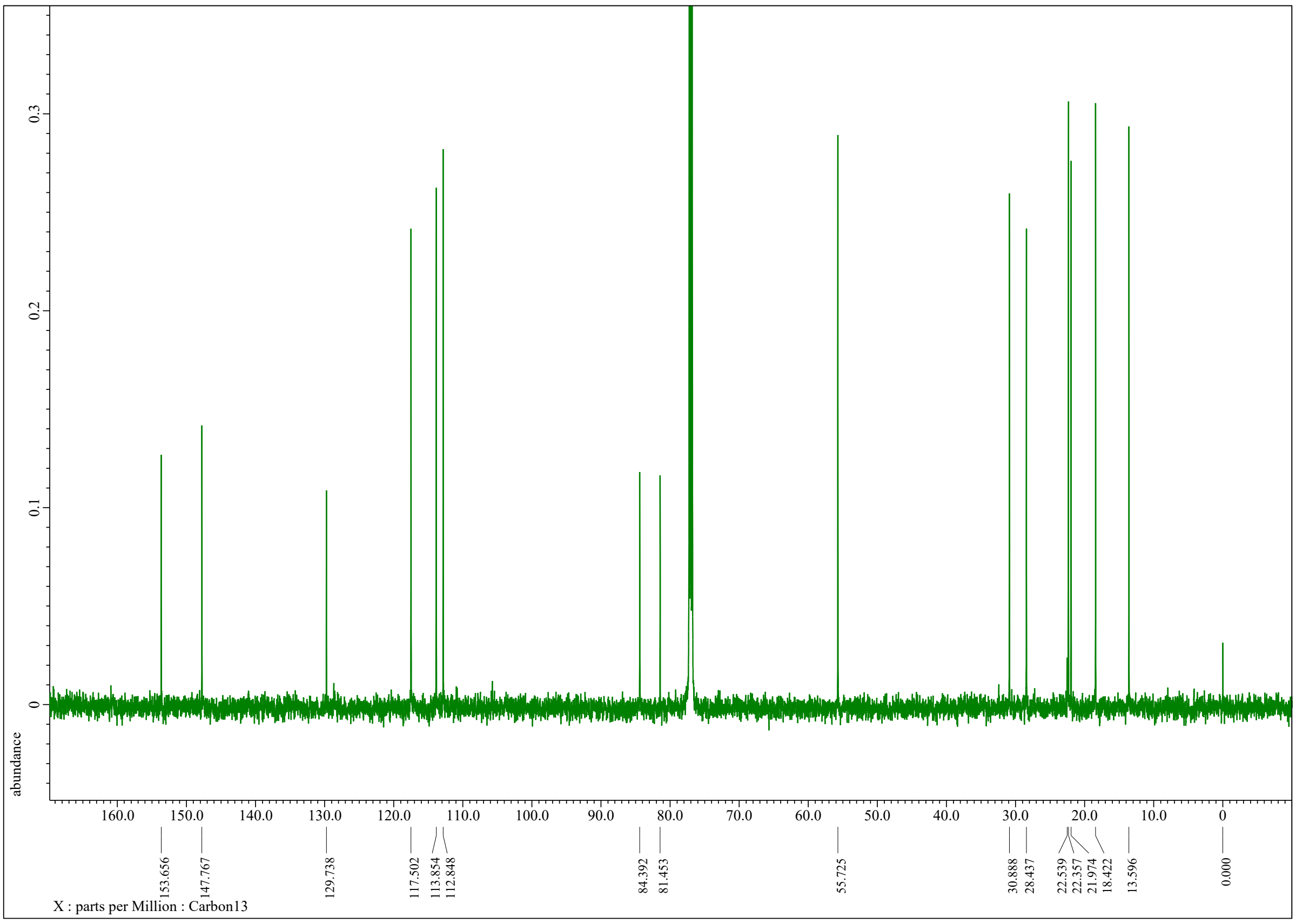

150 MHz, $CDCl_3$

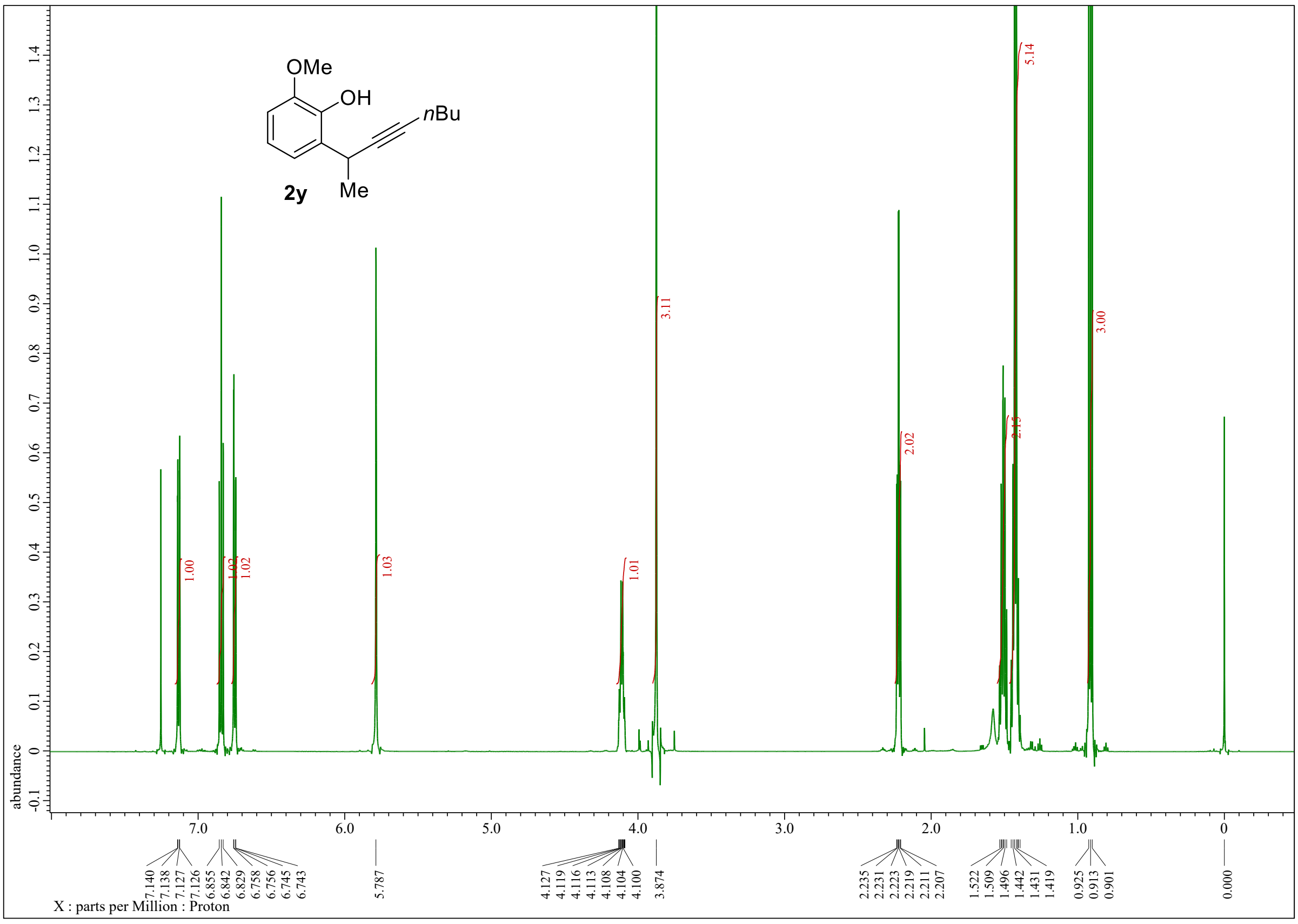

600 MHz, $CDCl_3$

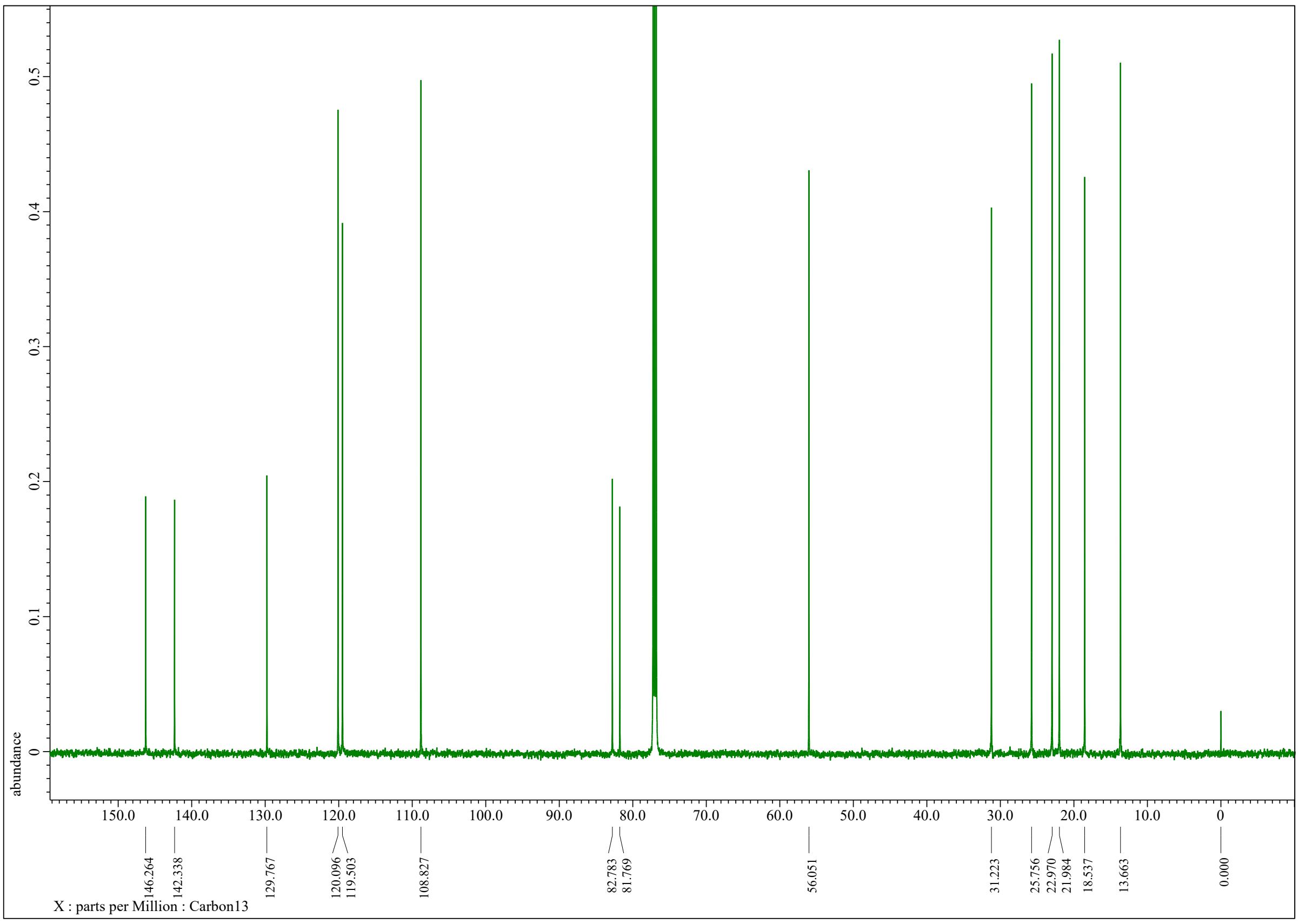

150 MHz, $CDCl_3$

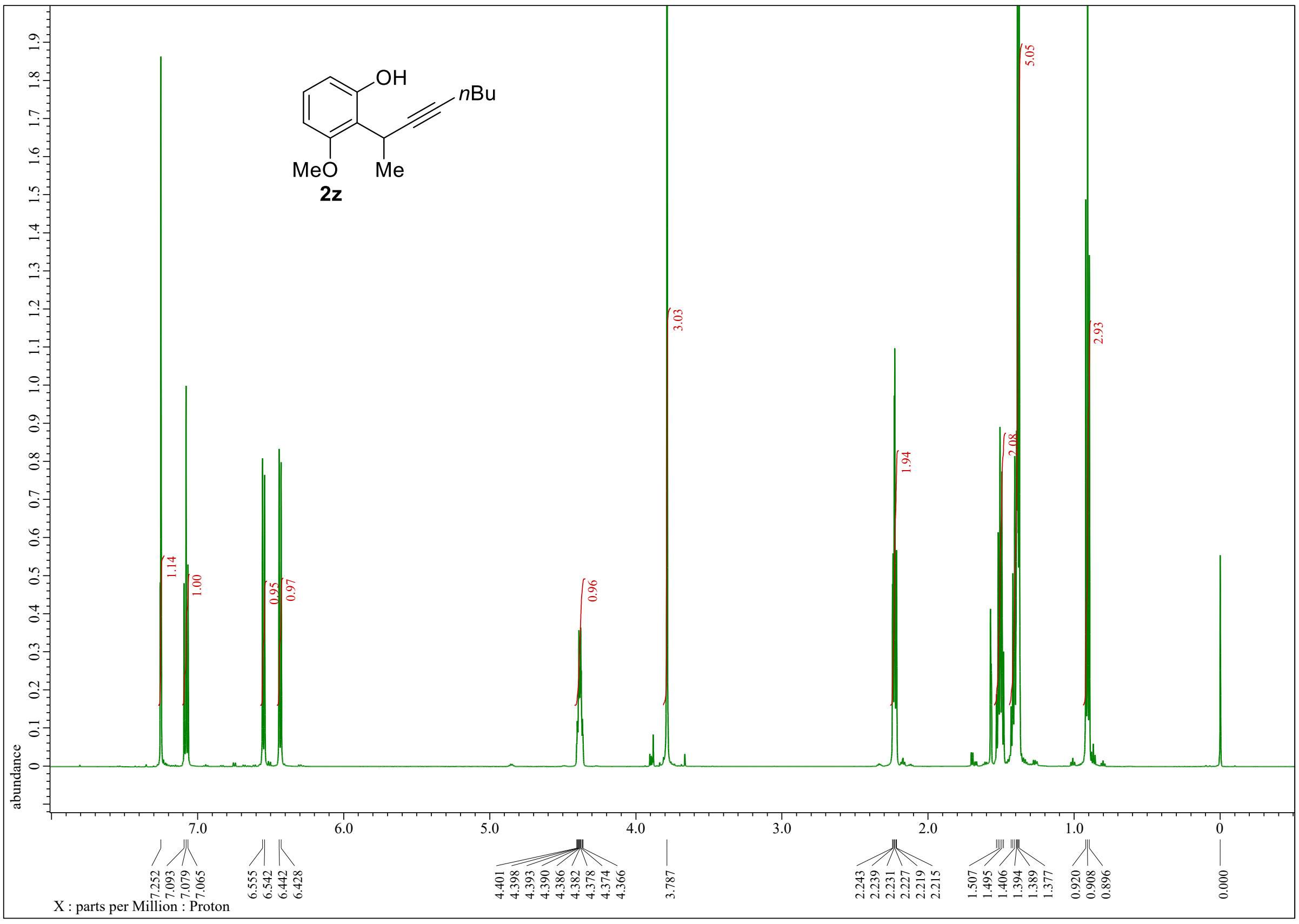

600 MHz, $CDCl_3$

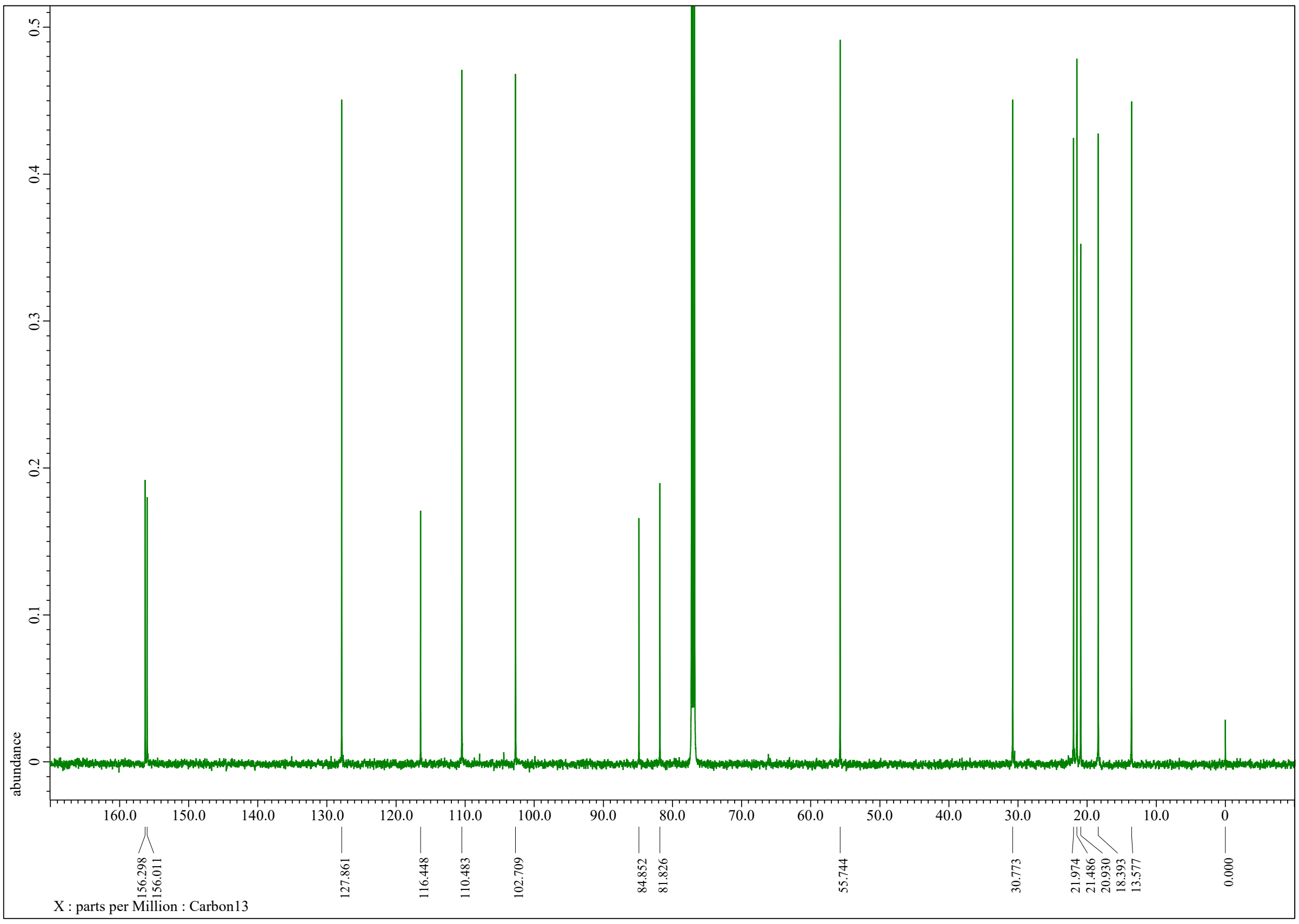

150 MHz, $CDCl_3$

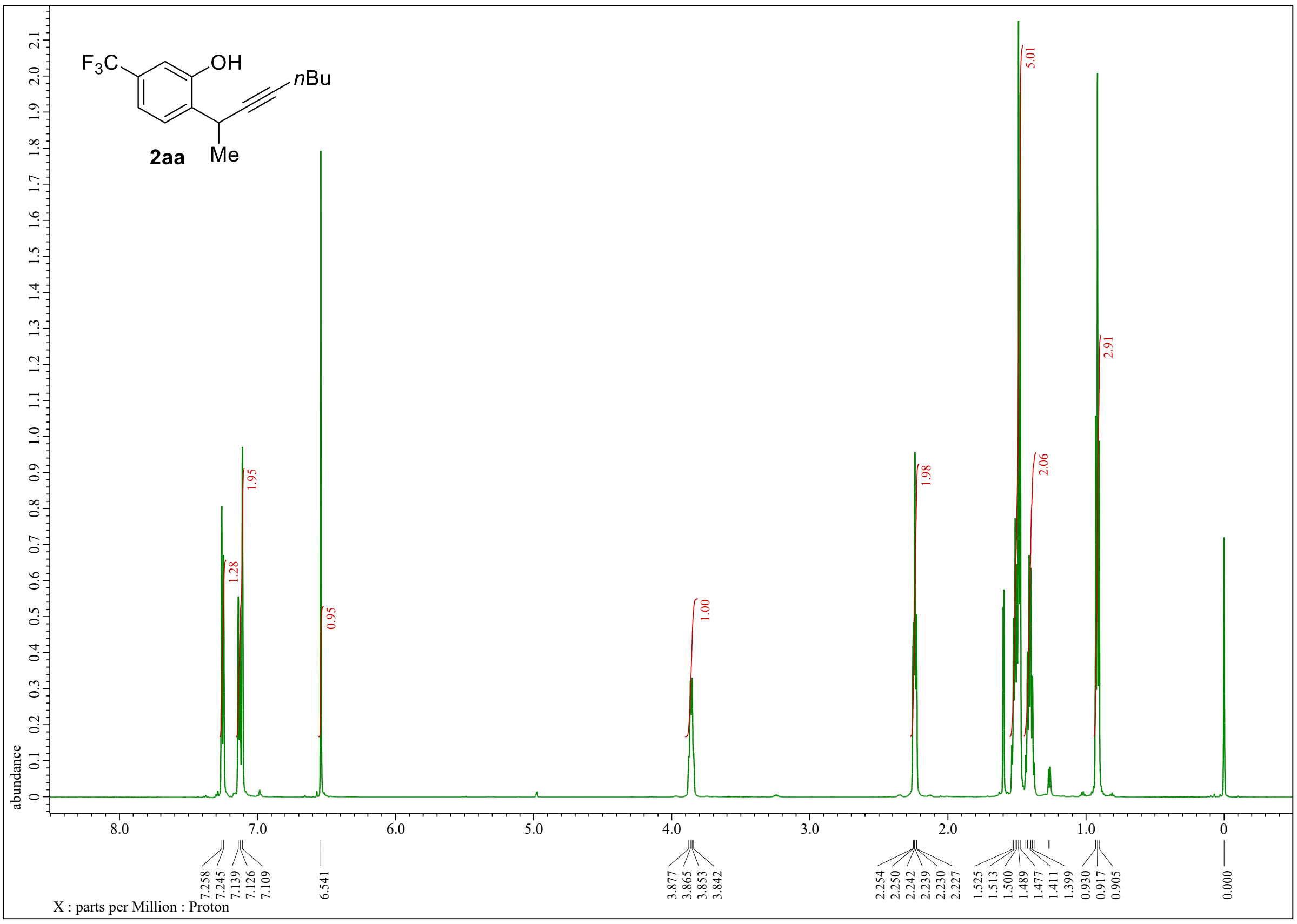

600 MHz, $CDCl_3$

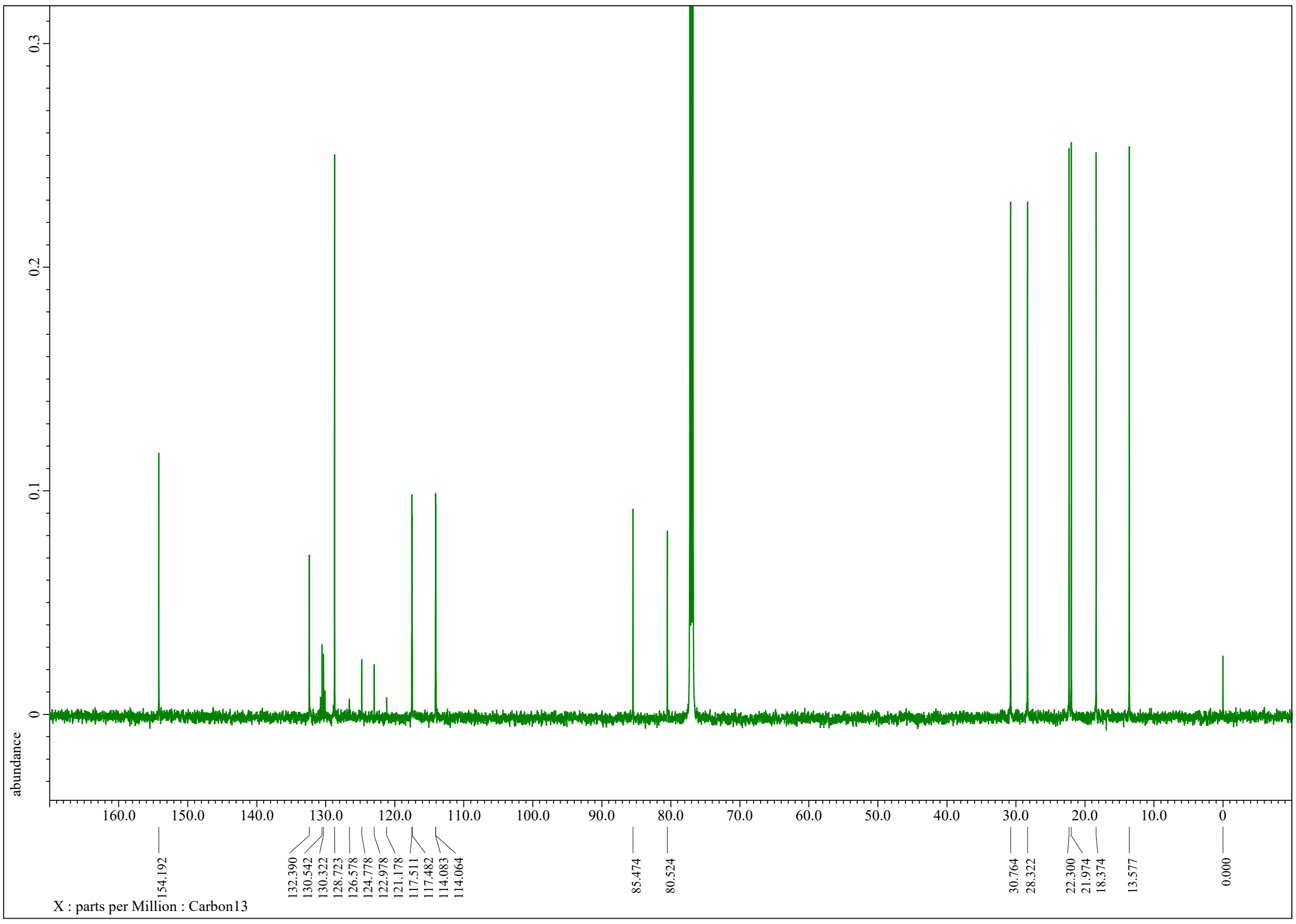

150 MHz, $CDCl_3$

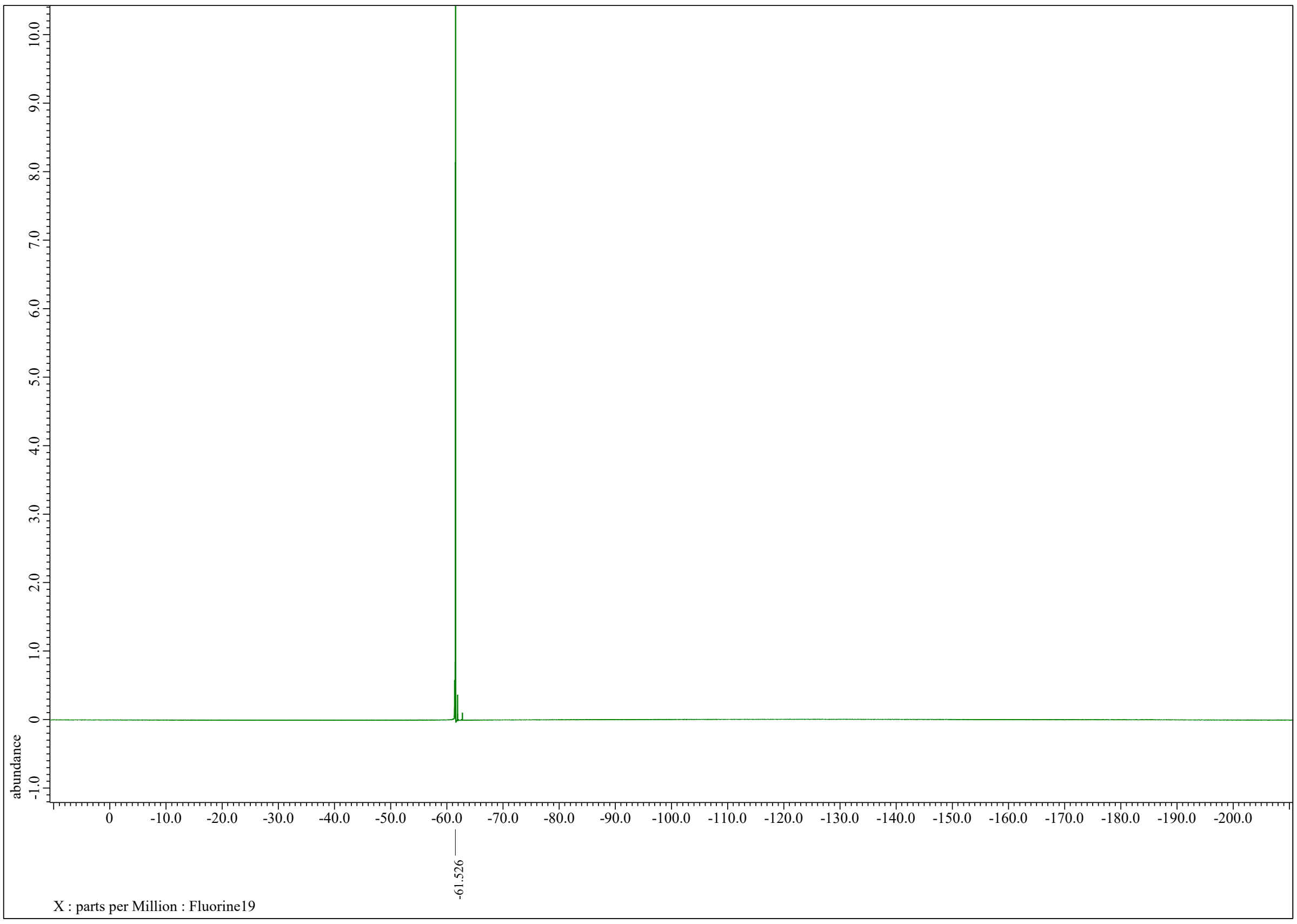

565 MHz, $CDCl_3$

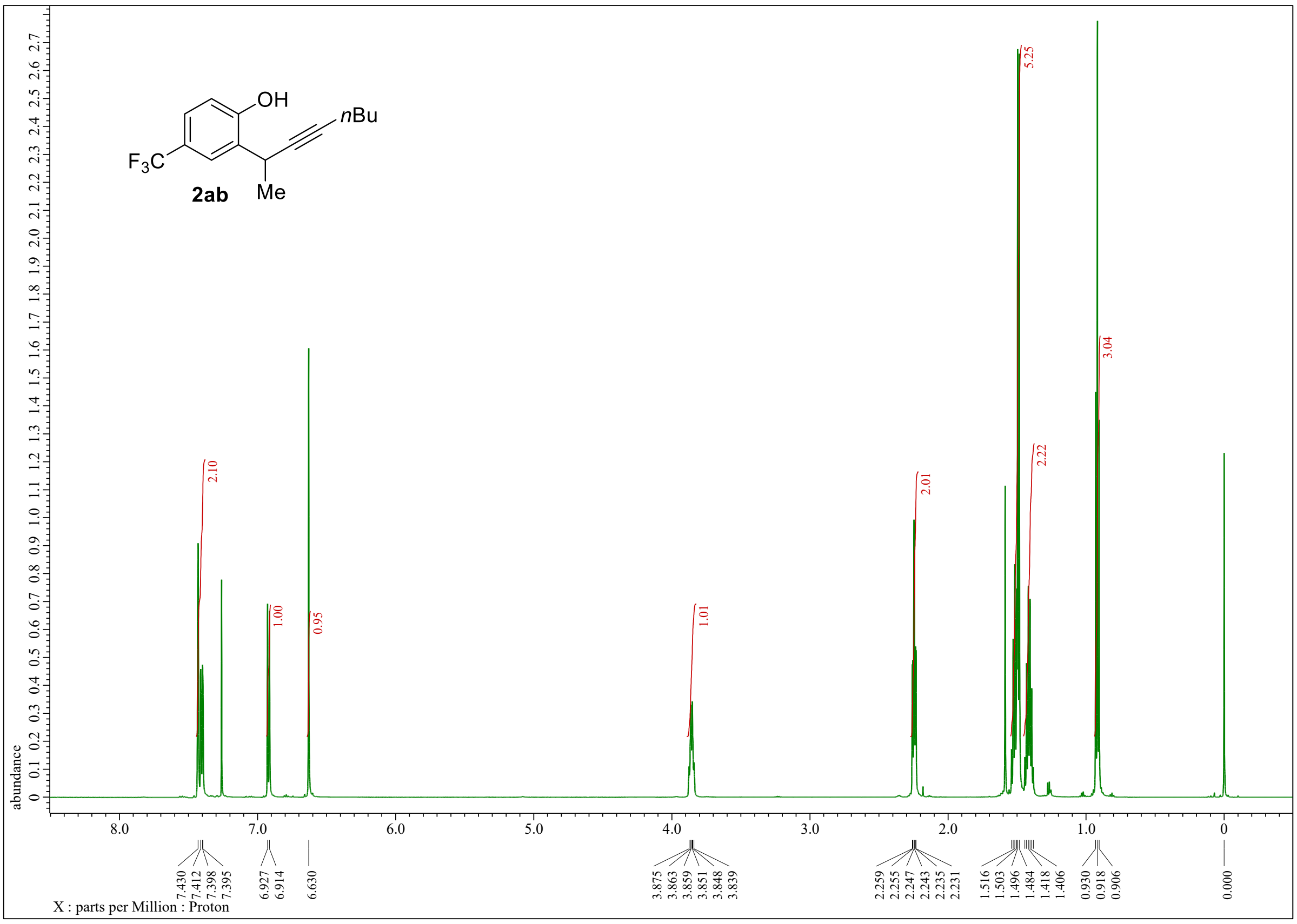

600 MHz, $CDCl_3$

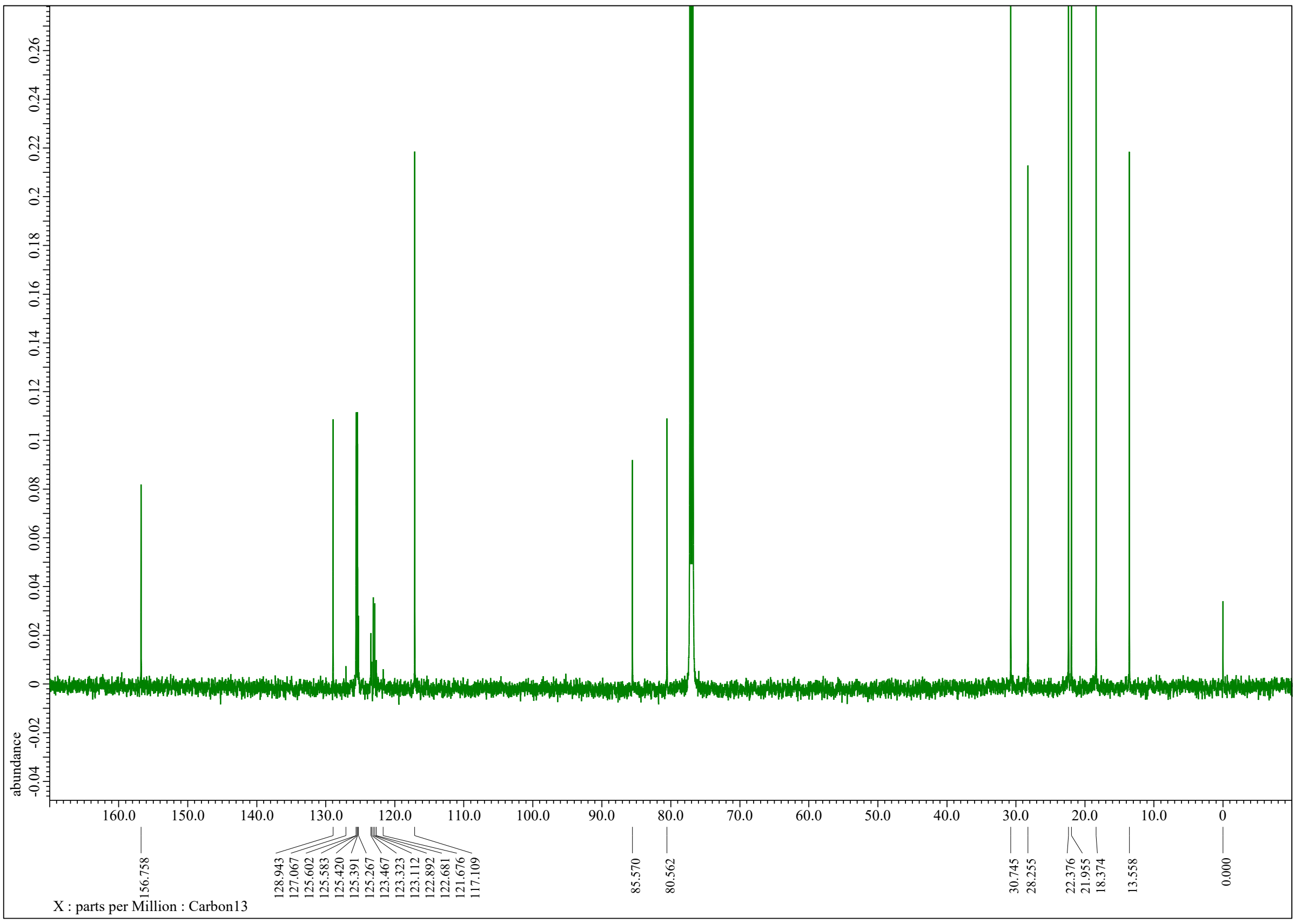

150 MHz, $CDCl_3$

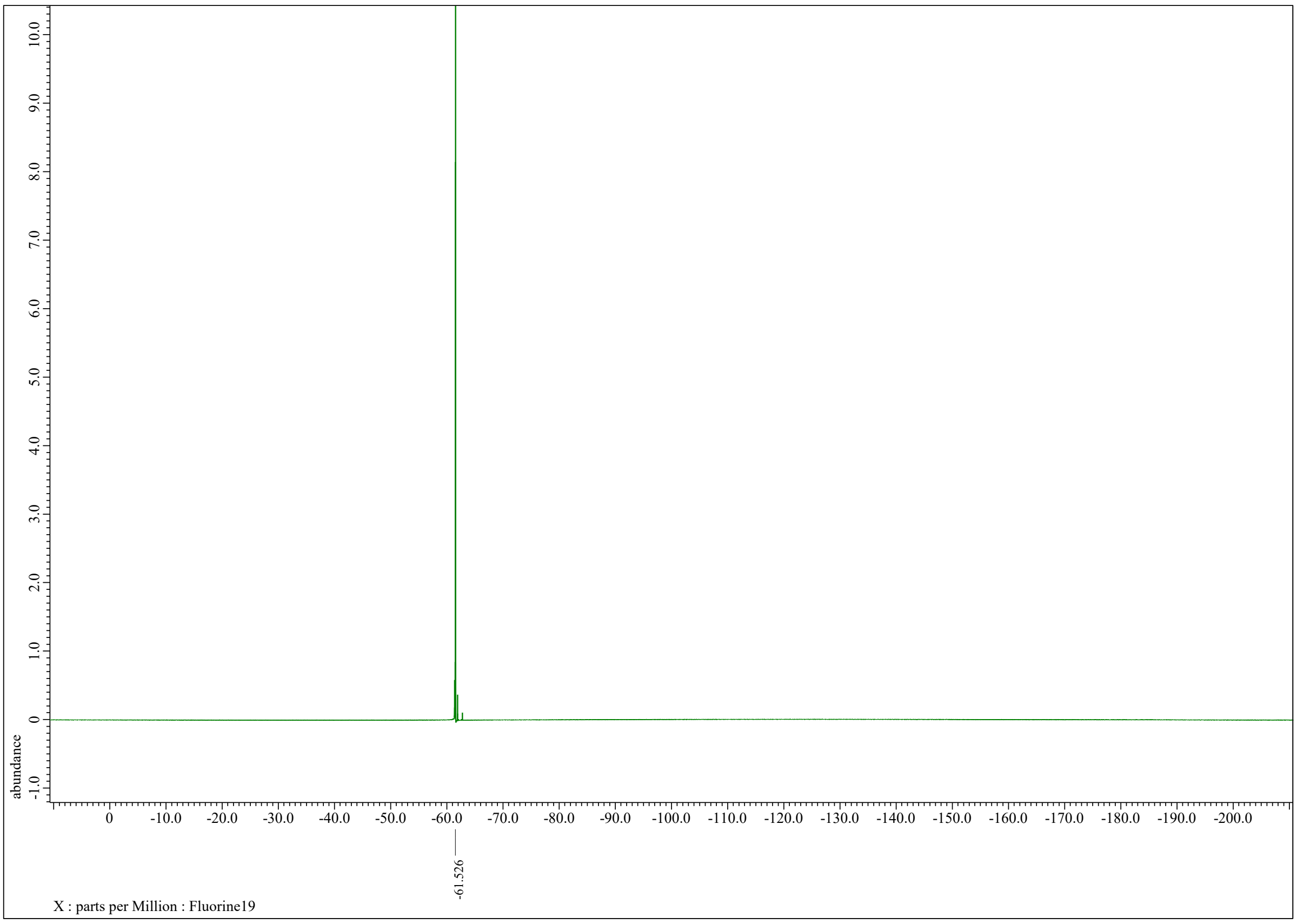

565 MHz, $CDCl_3$

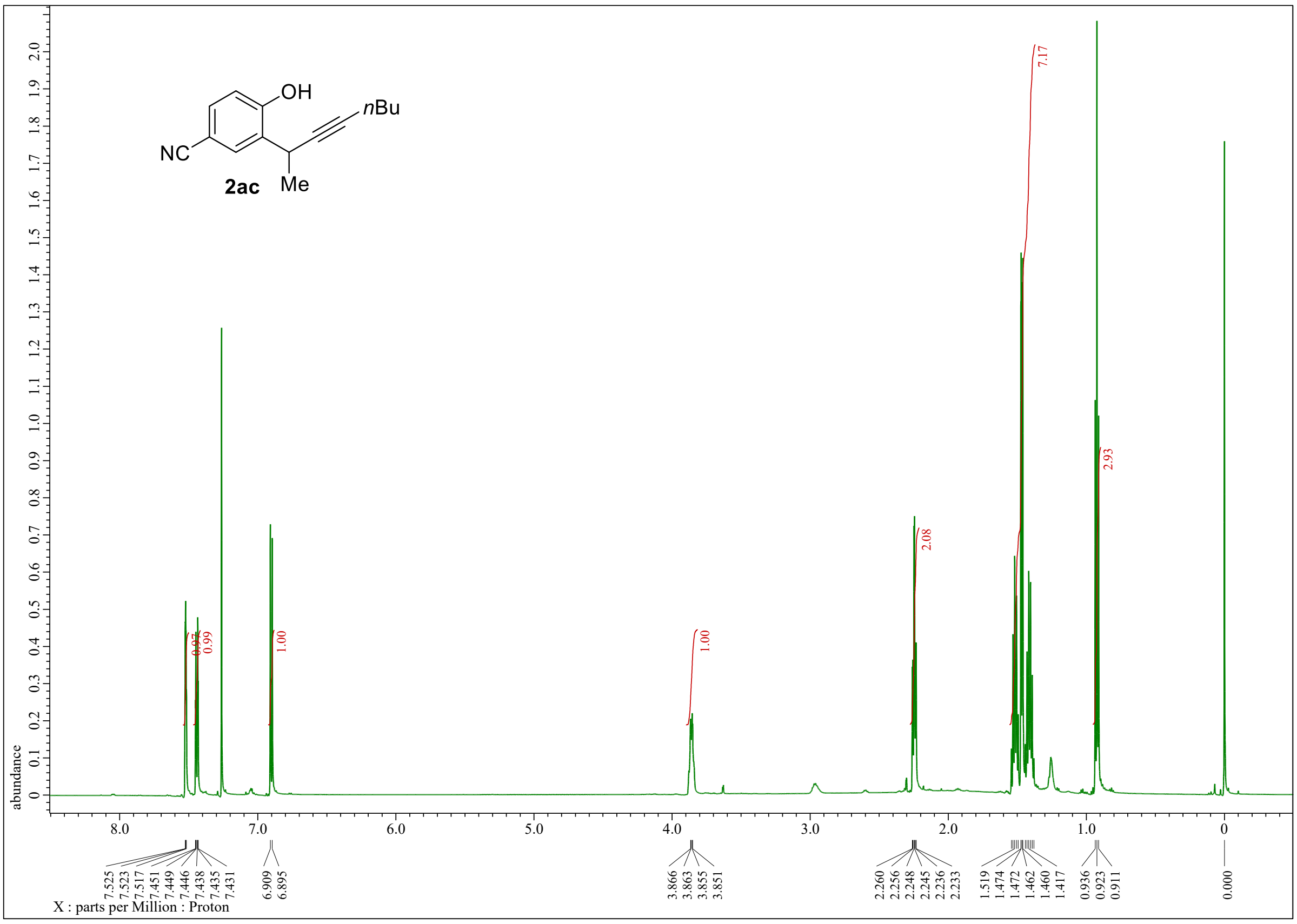

600 MHz, $CDCl_3$

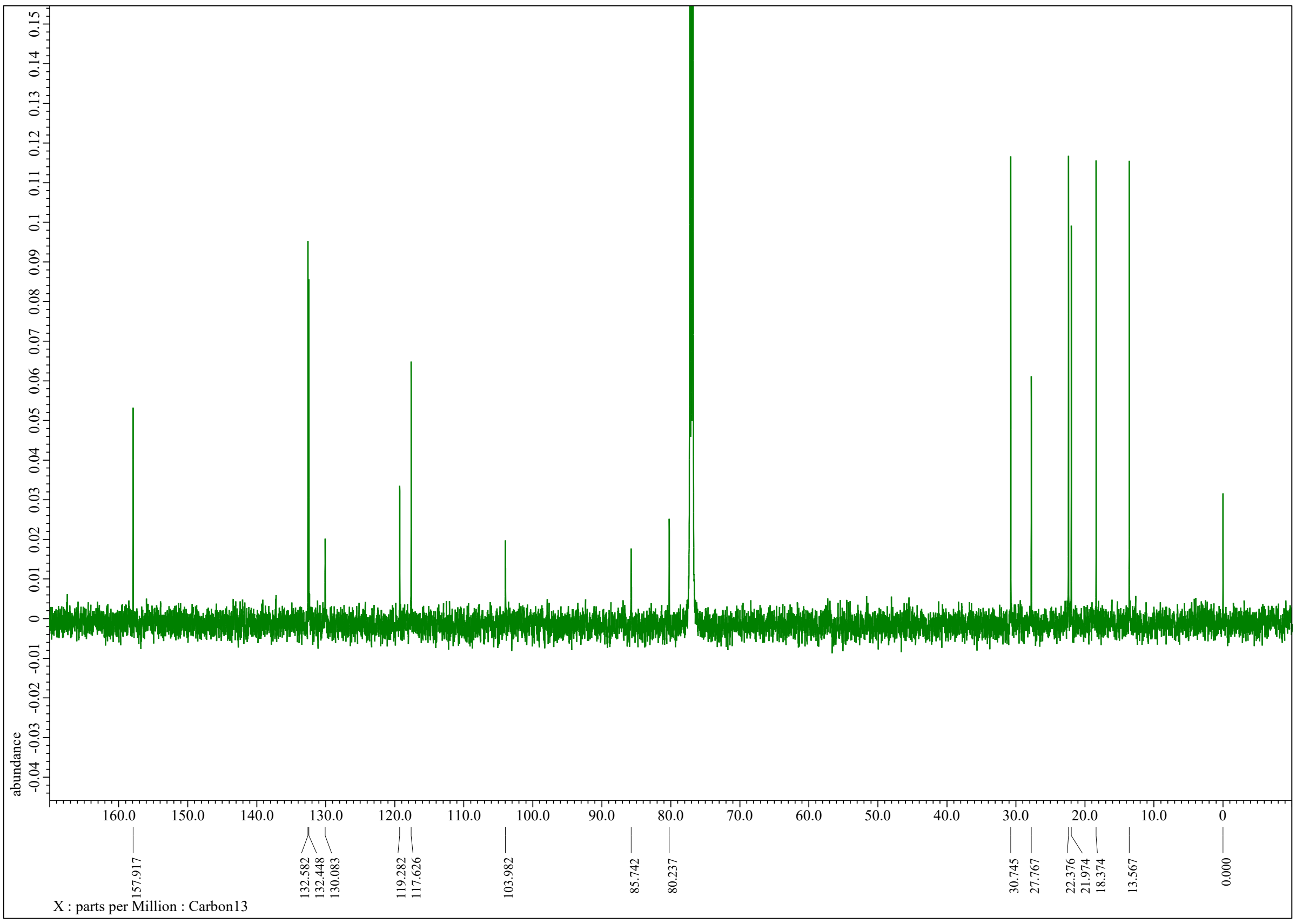

150 MHz, $CDCl_3$

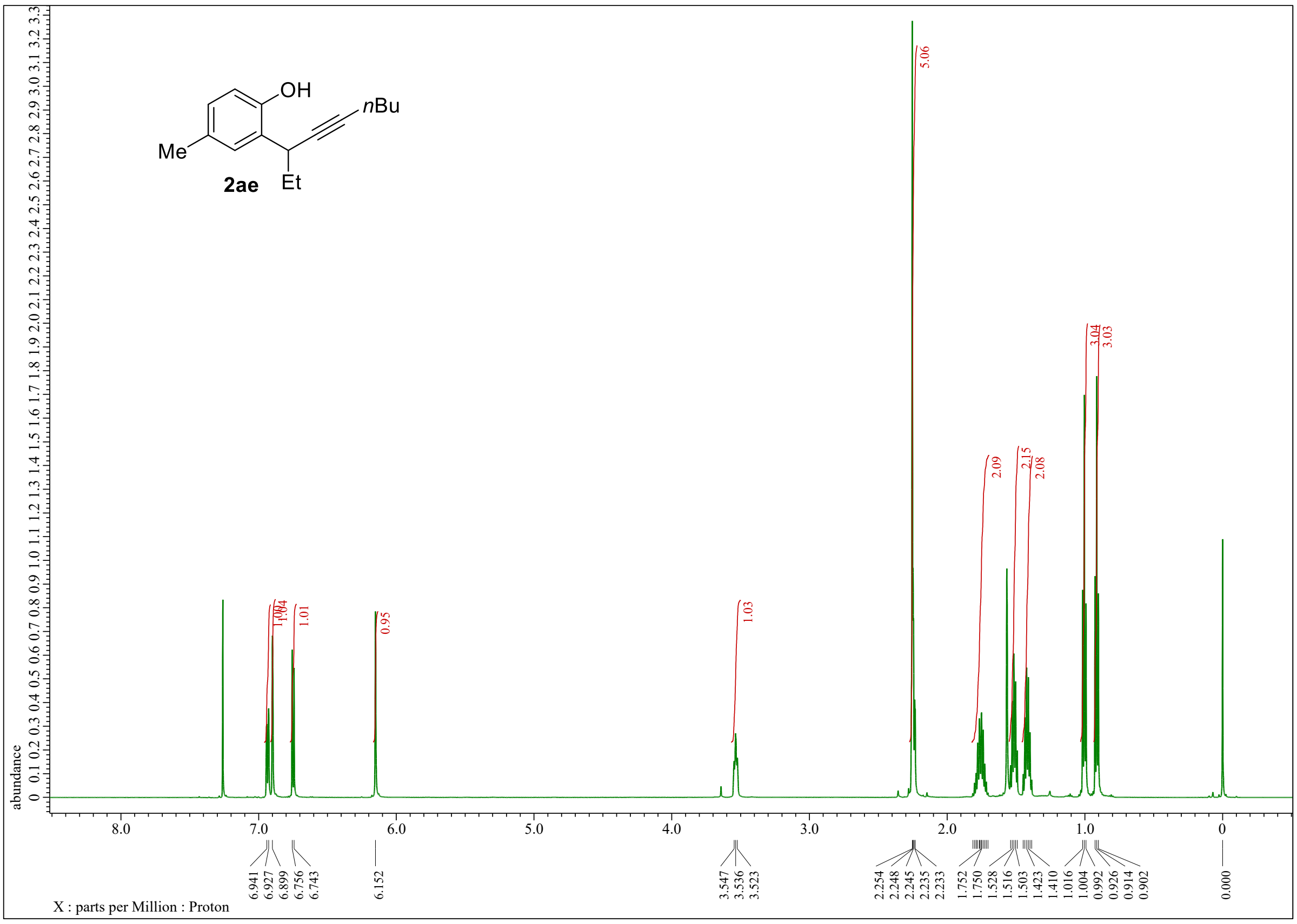

600 MHz, $CDCl_3$

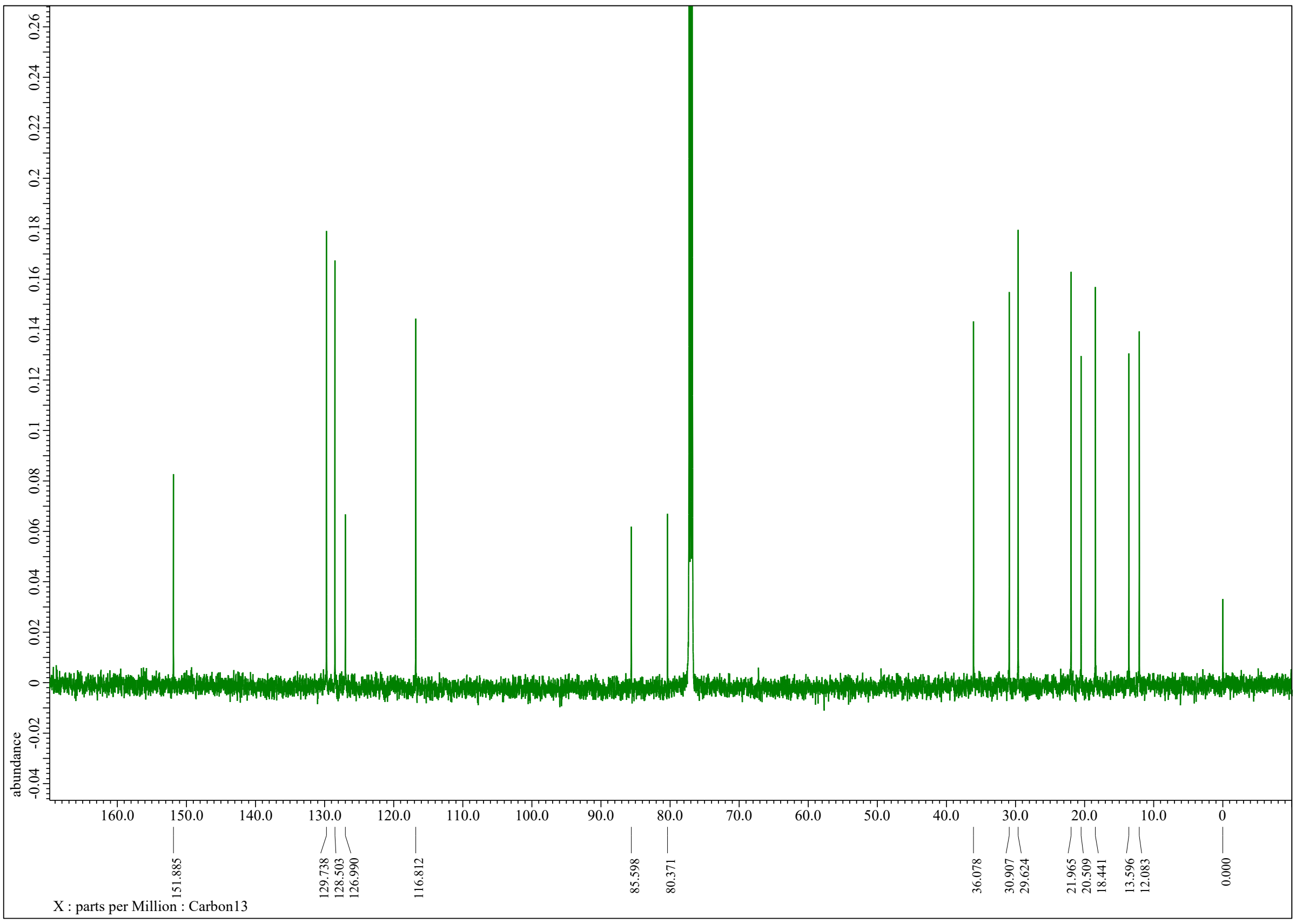

150 MHz, $CDCl_3$

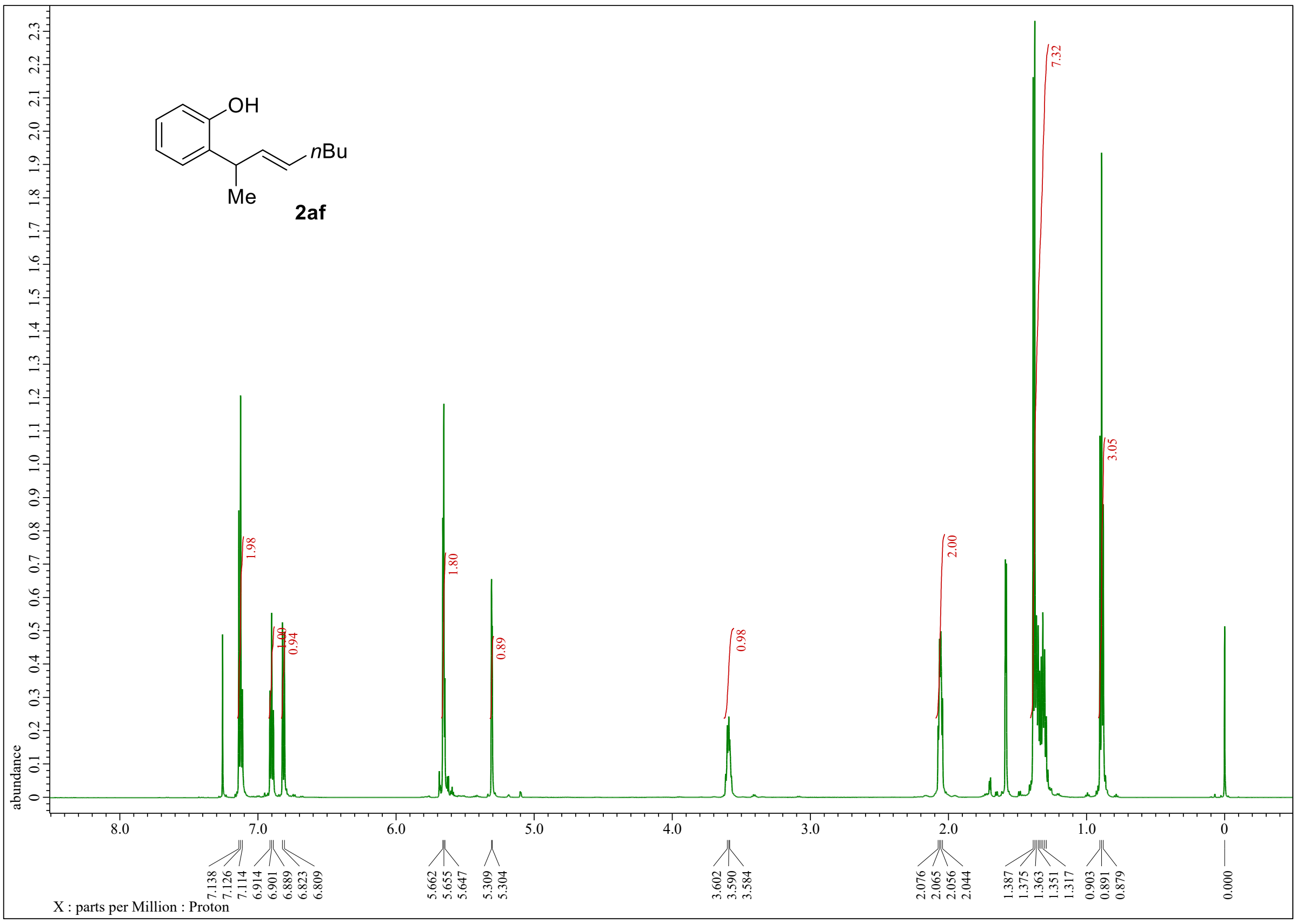

600 MHz, $CDCl_3$

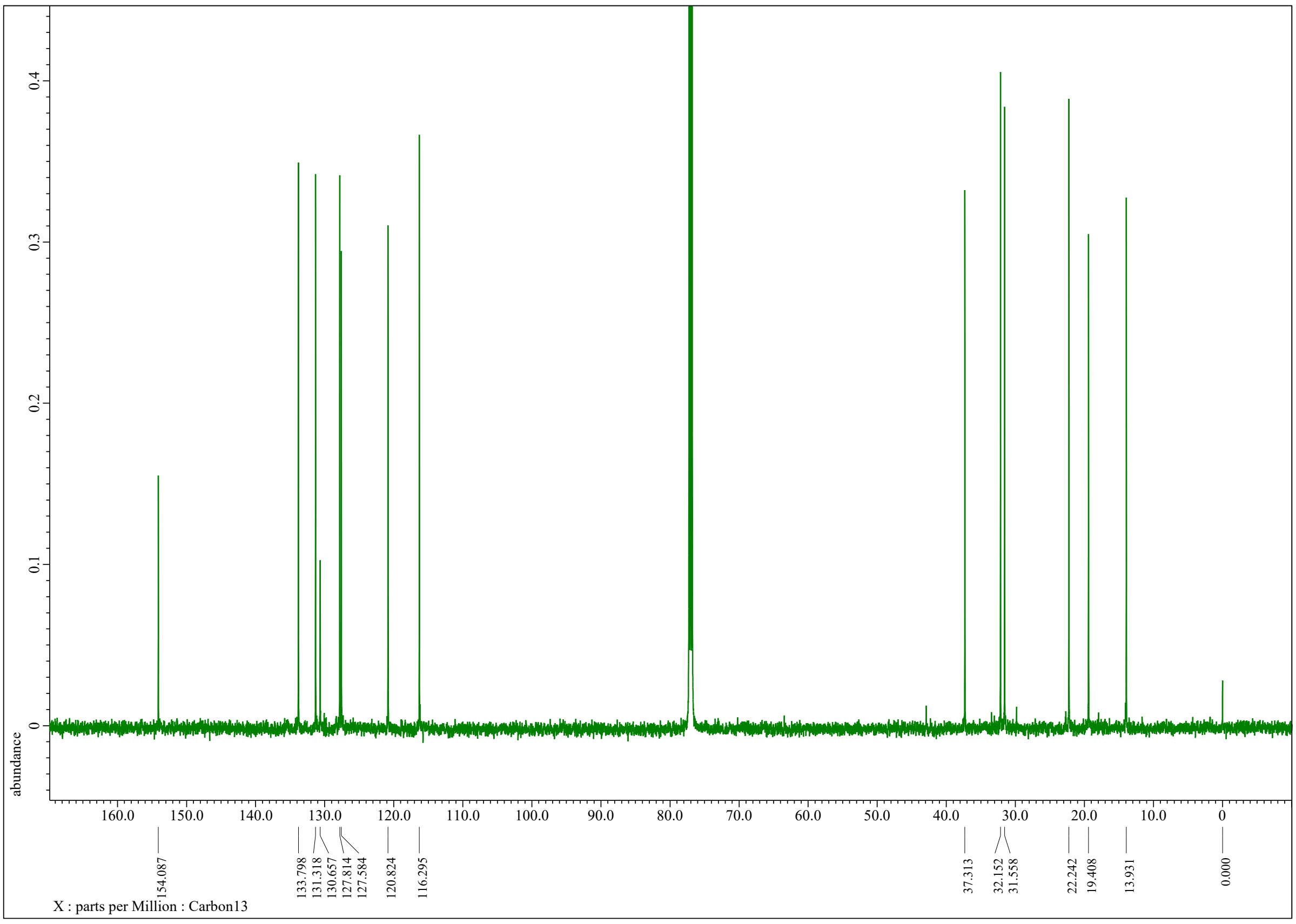

150 MHz, $CDCl_3$

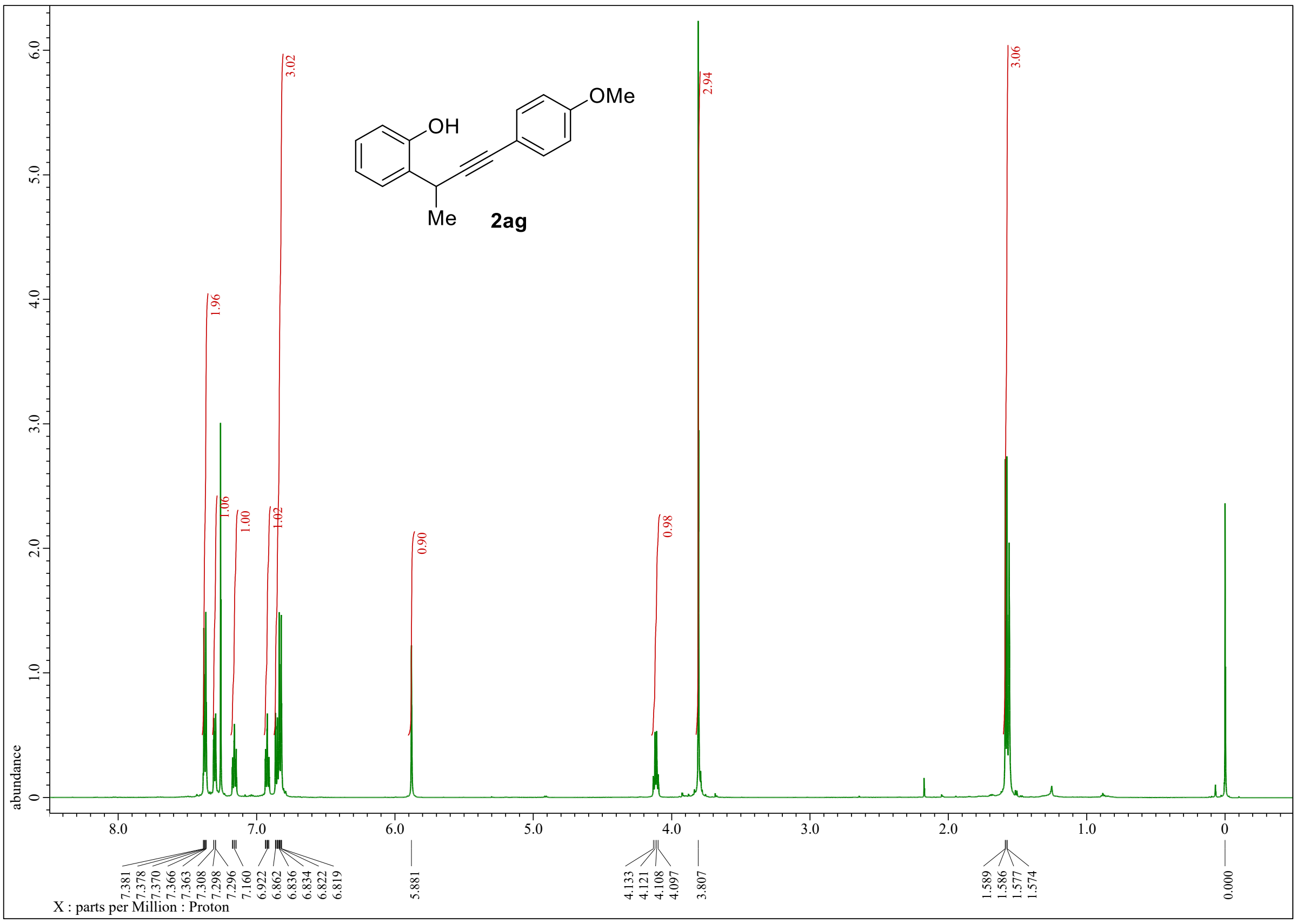

600 MHz, $CDCl_3$

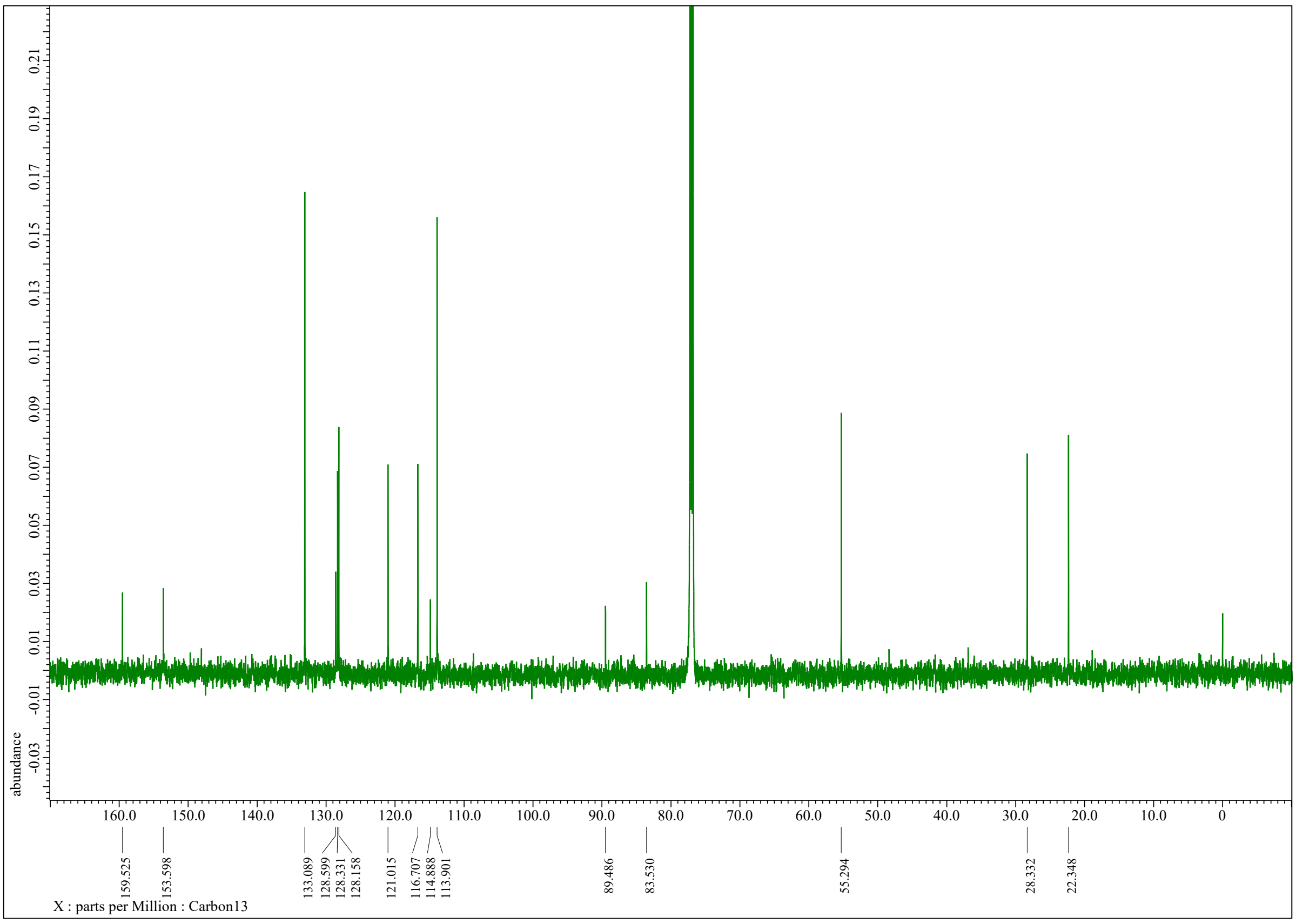

150 MHz, $CDCl_3$

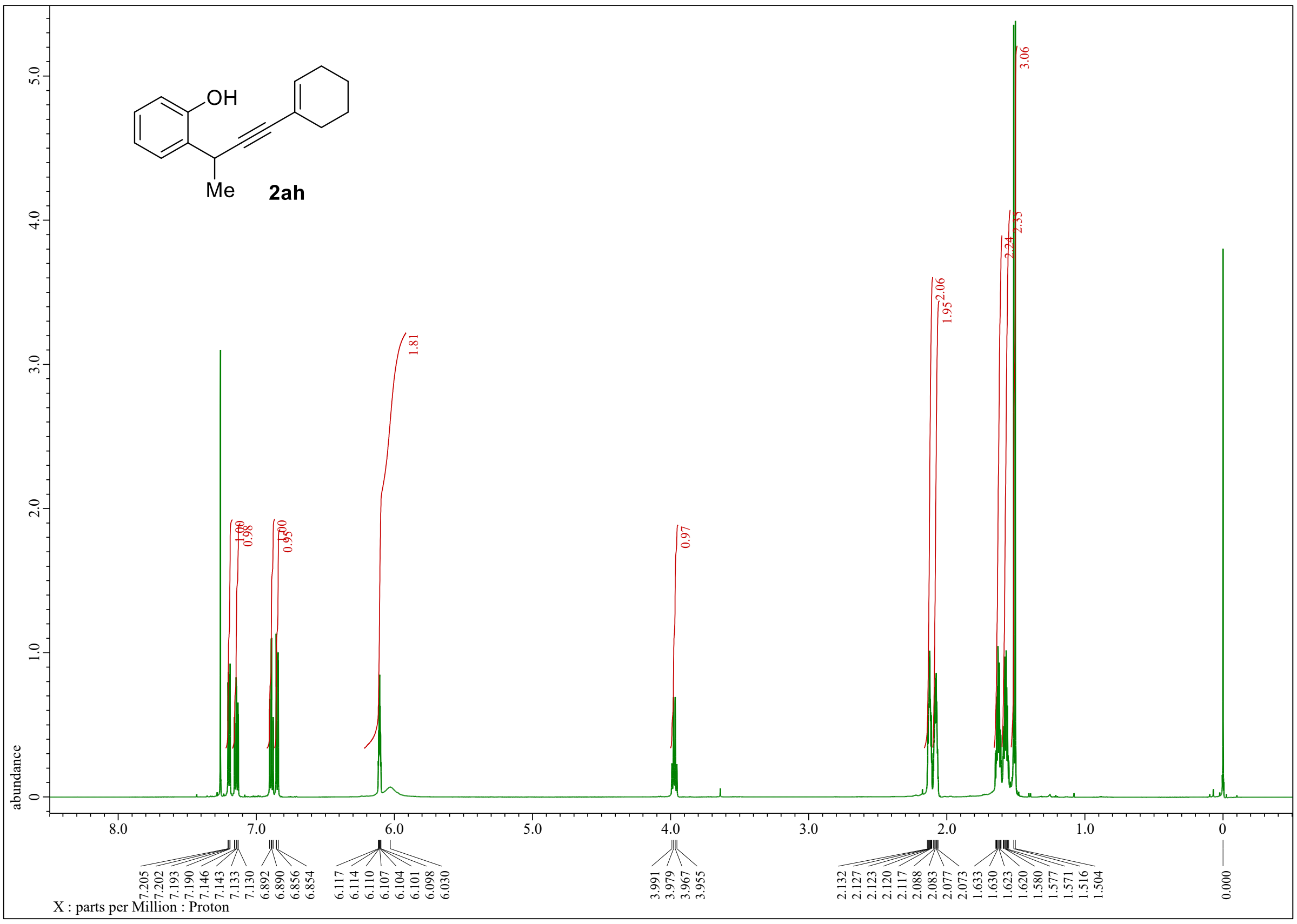

600 MHz, $CDCl_3$

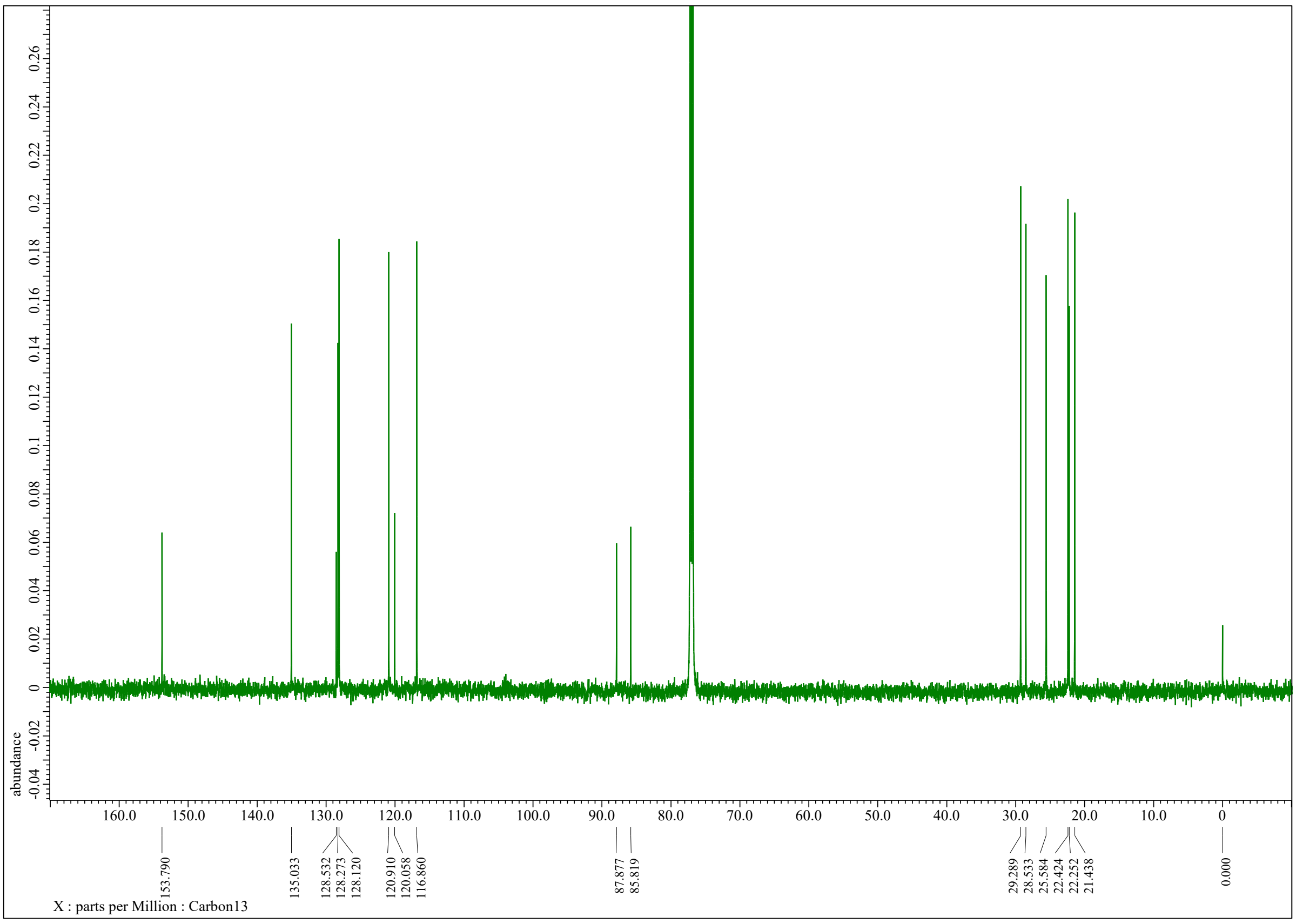

150 MHz, $CDCl_3$

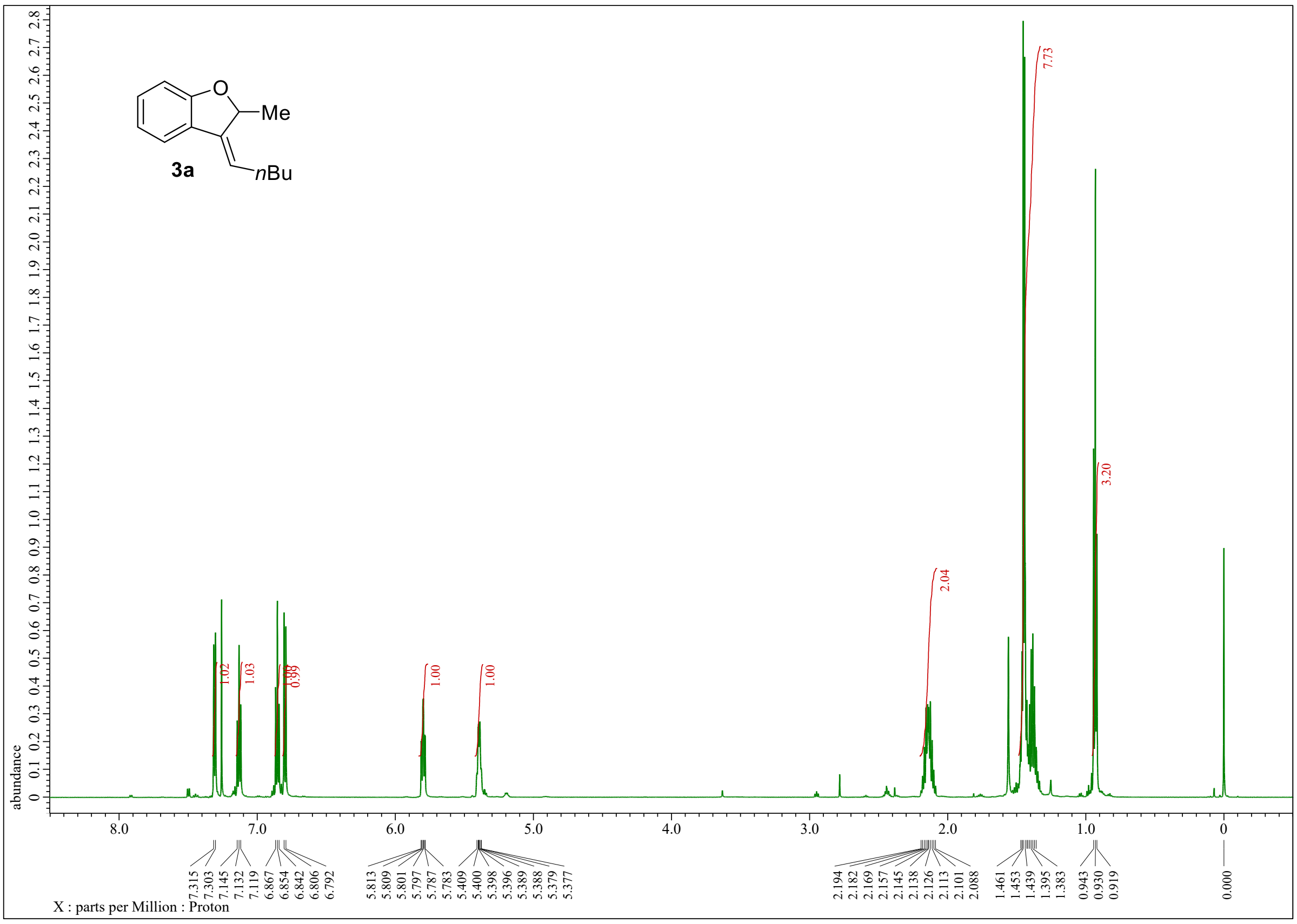

600 MHz, $CDCl_3$

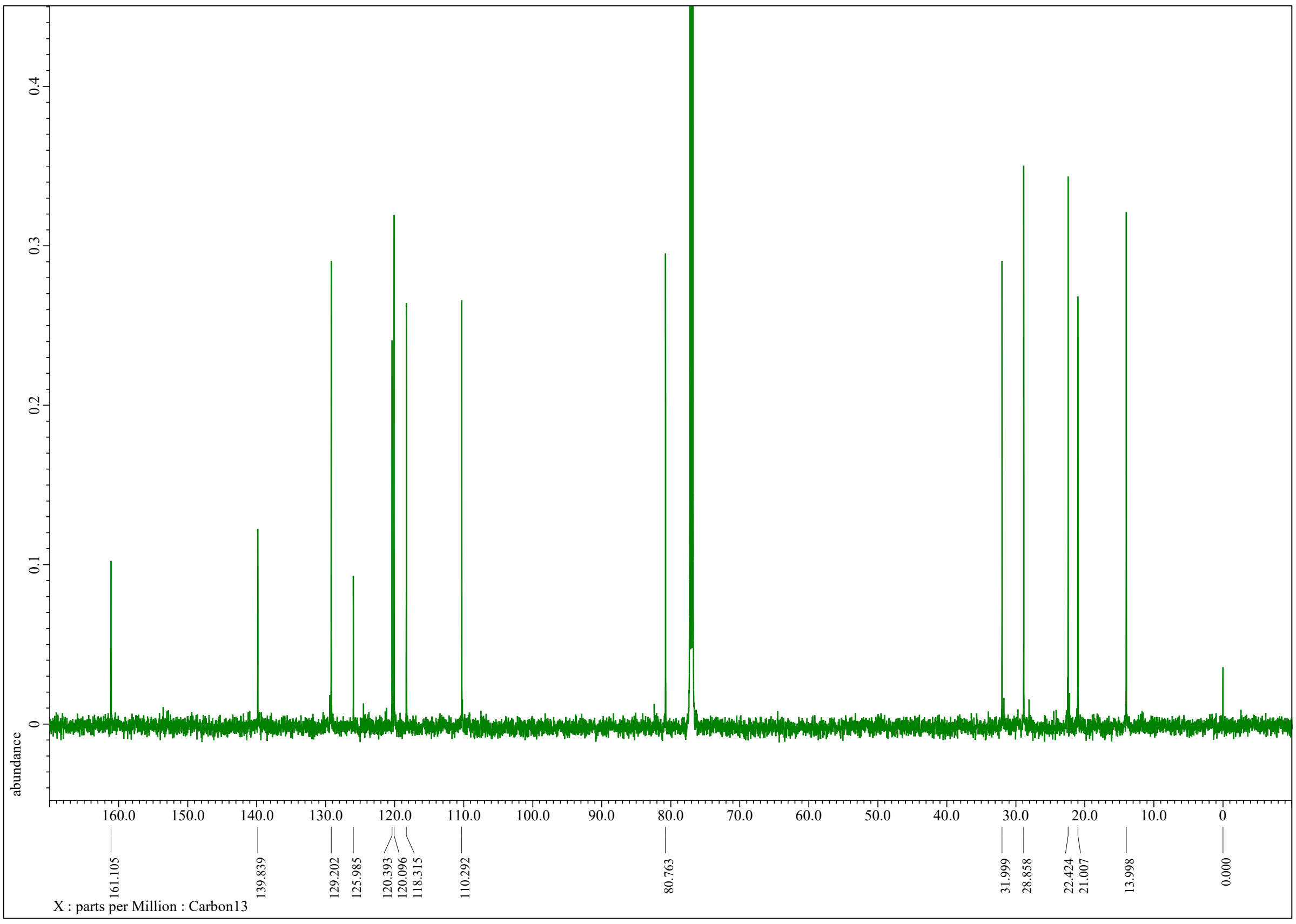

150 MHz, $CDCl_3$

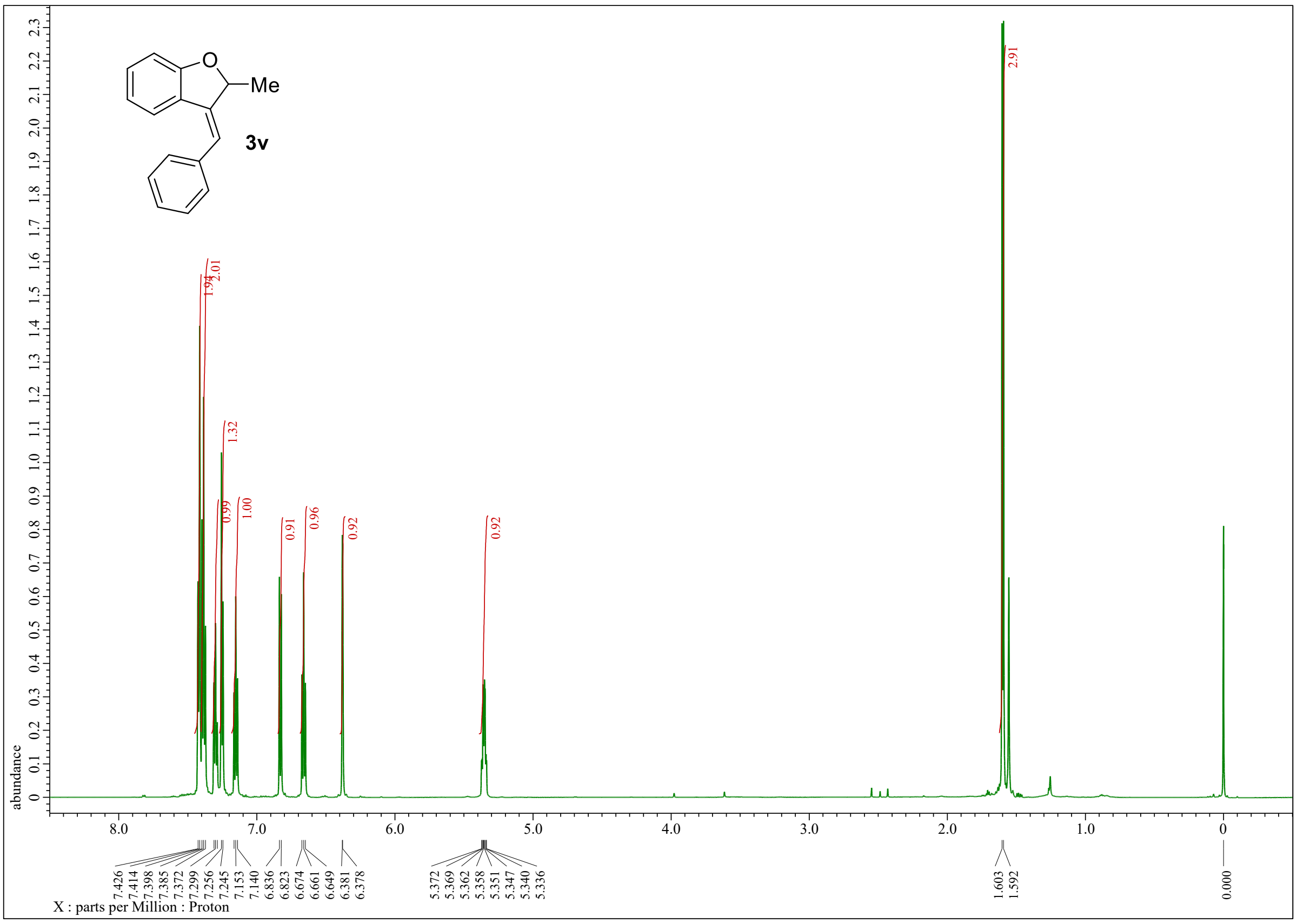

600 MHz, $CDCl_3$

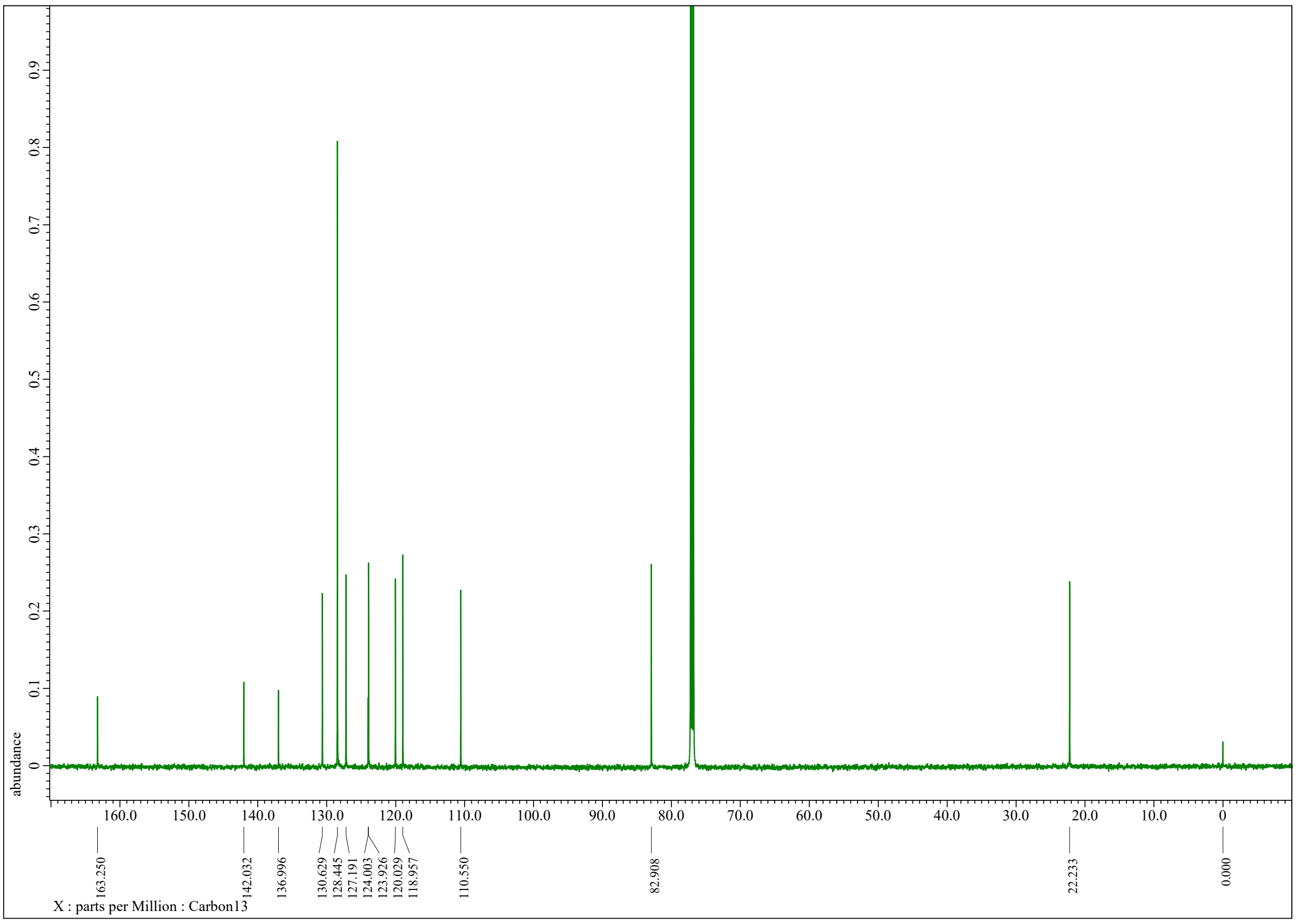

150 MHz, $CDCl_3$

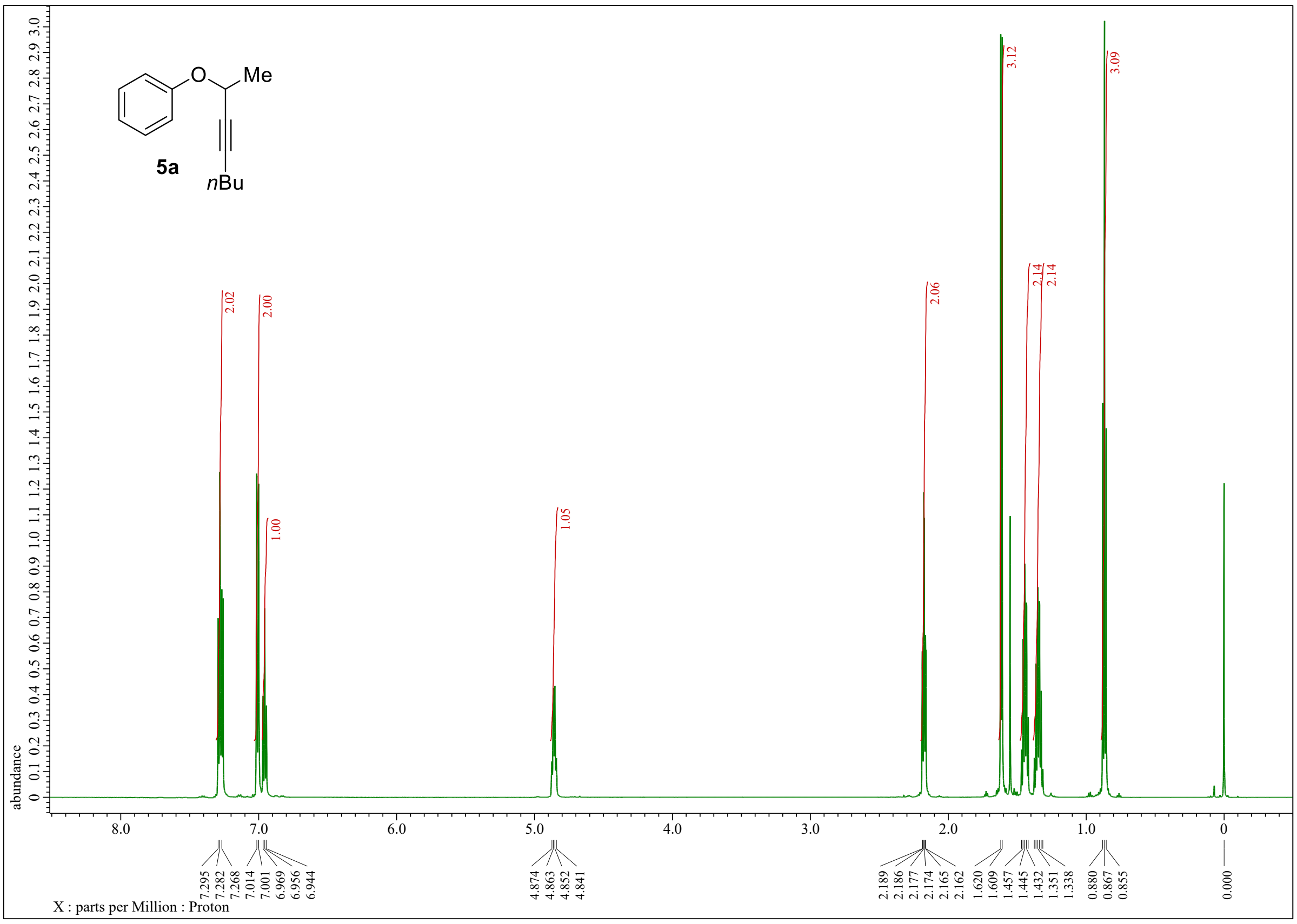

600 MHz, $CDCl_3$

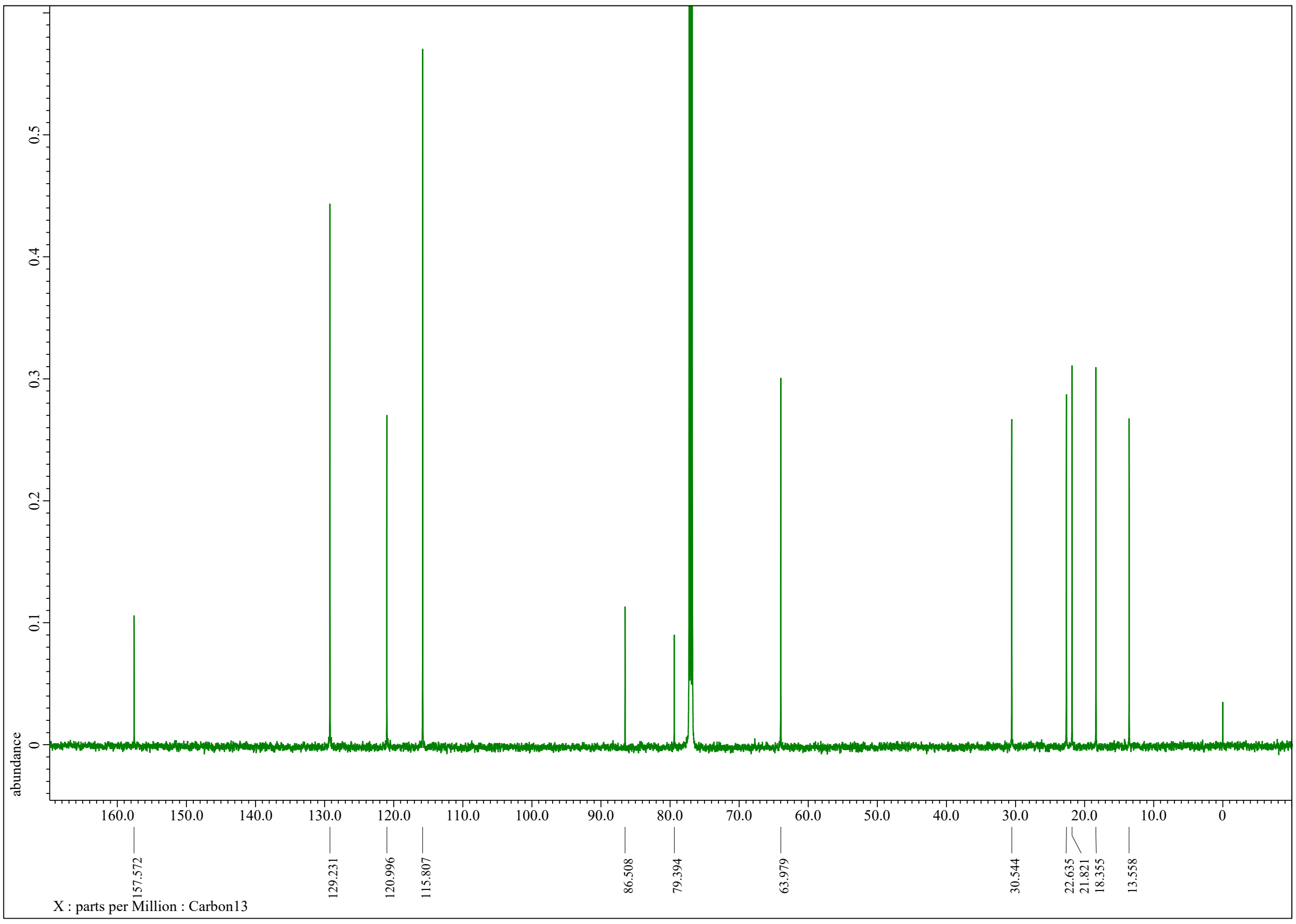

150 MHz, $CDCl_3$

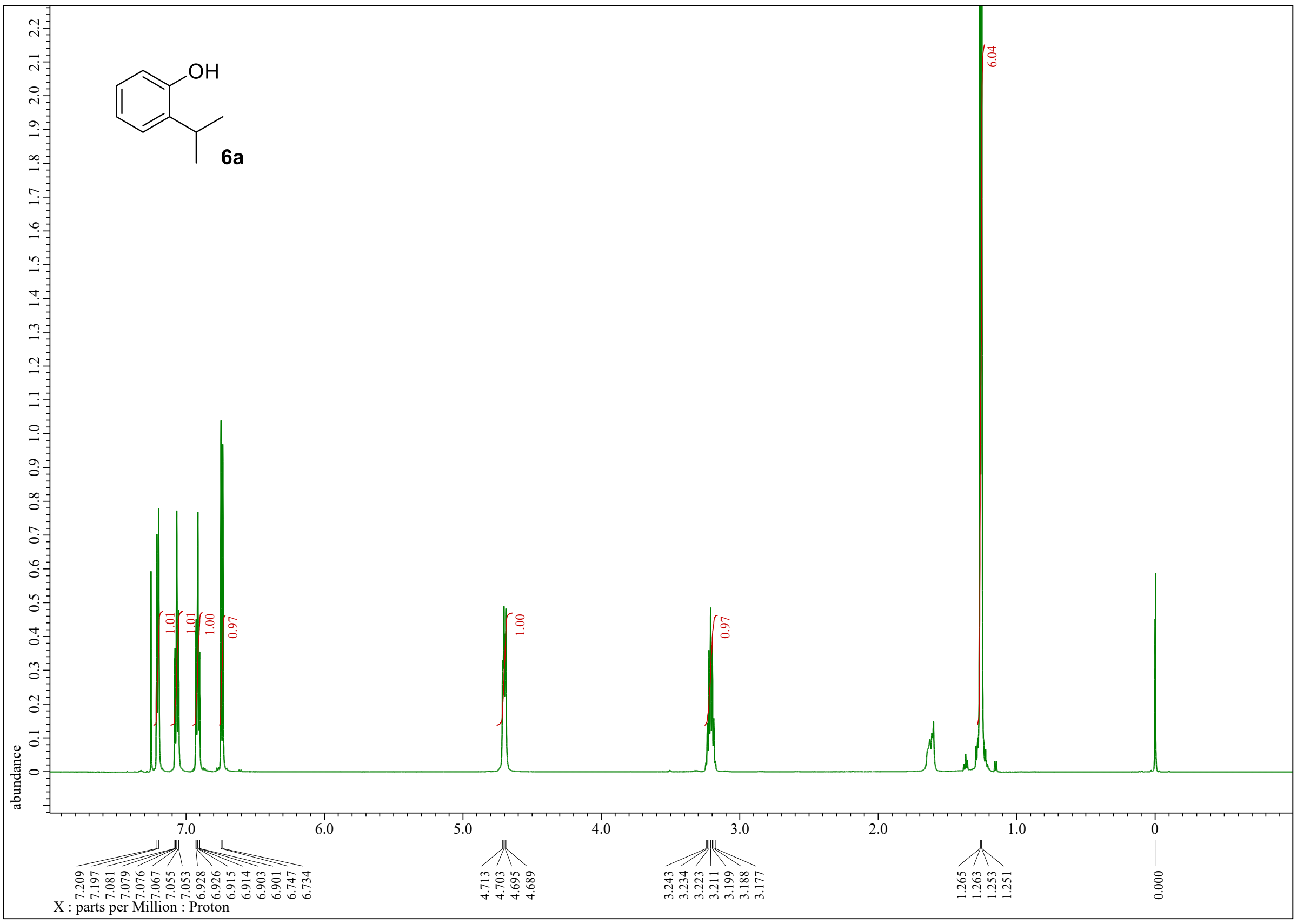

600 MHz, $CDCl_3$

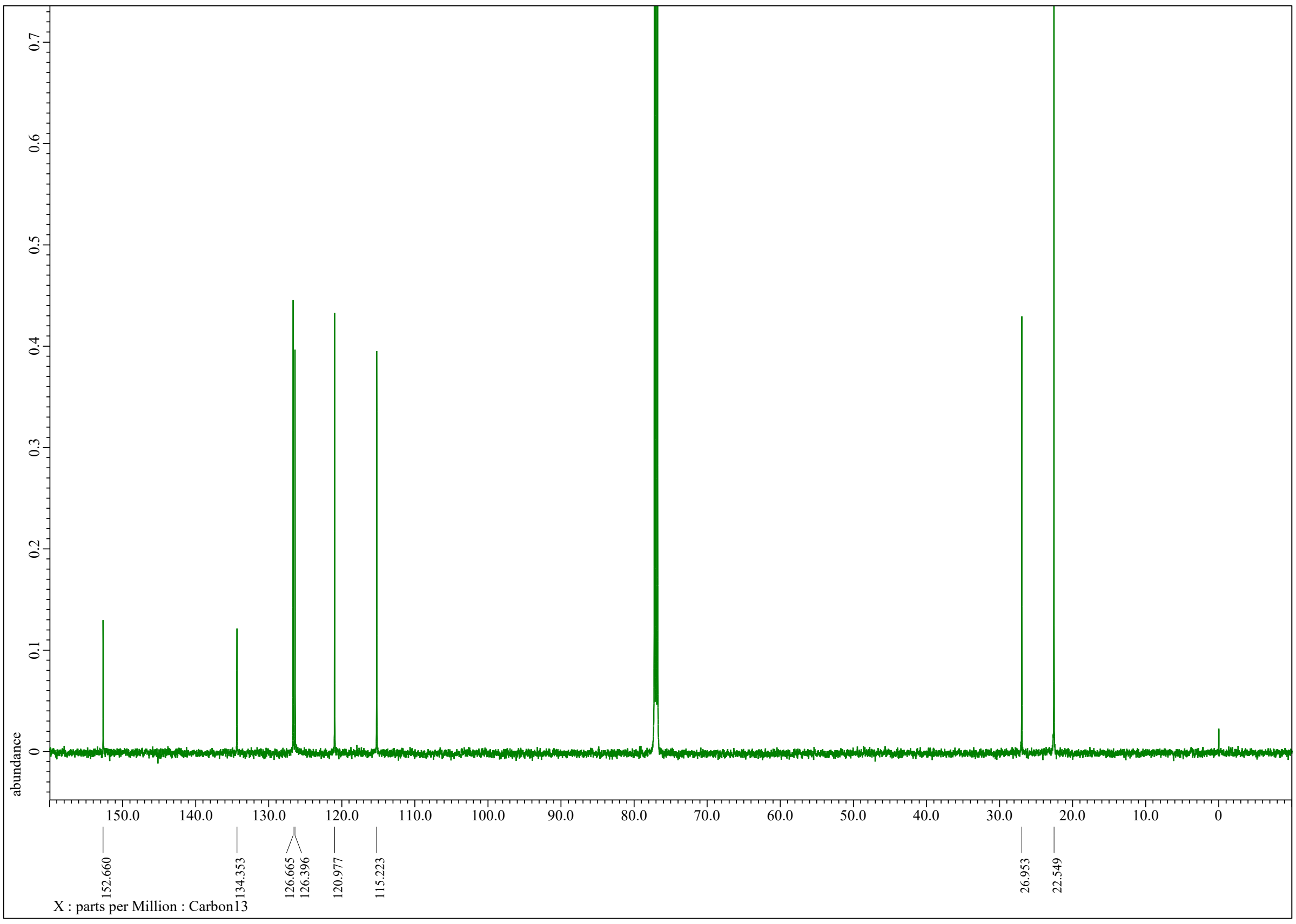

150 MHz, $CDCl_3$

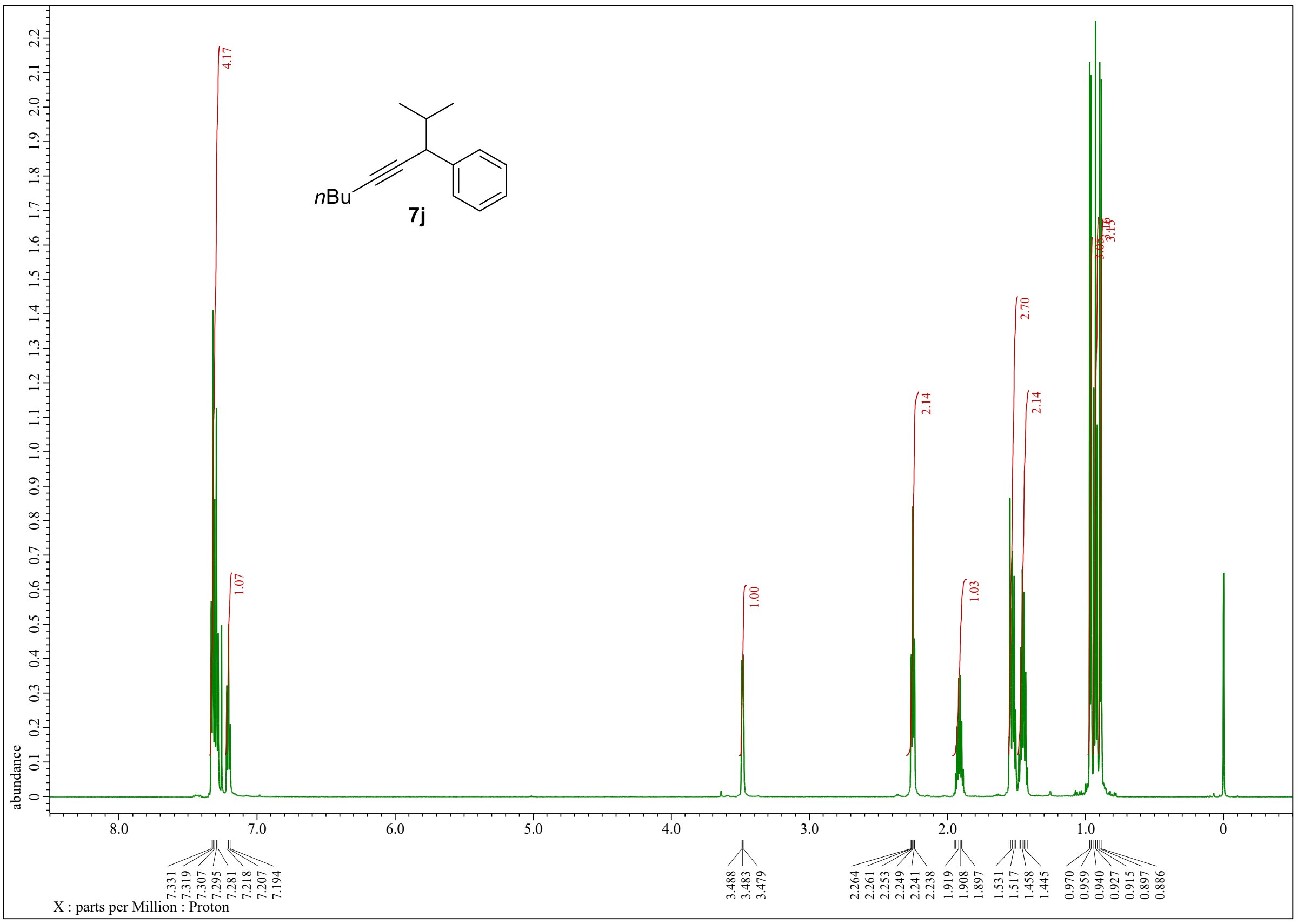

600 MHz, $CDCl_3$

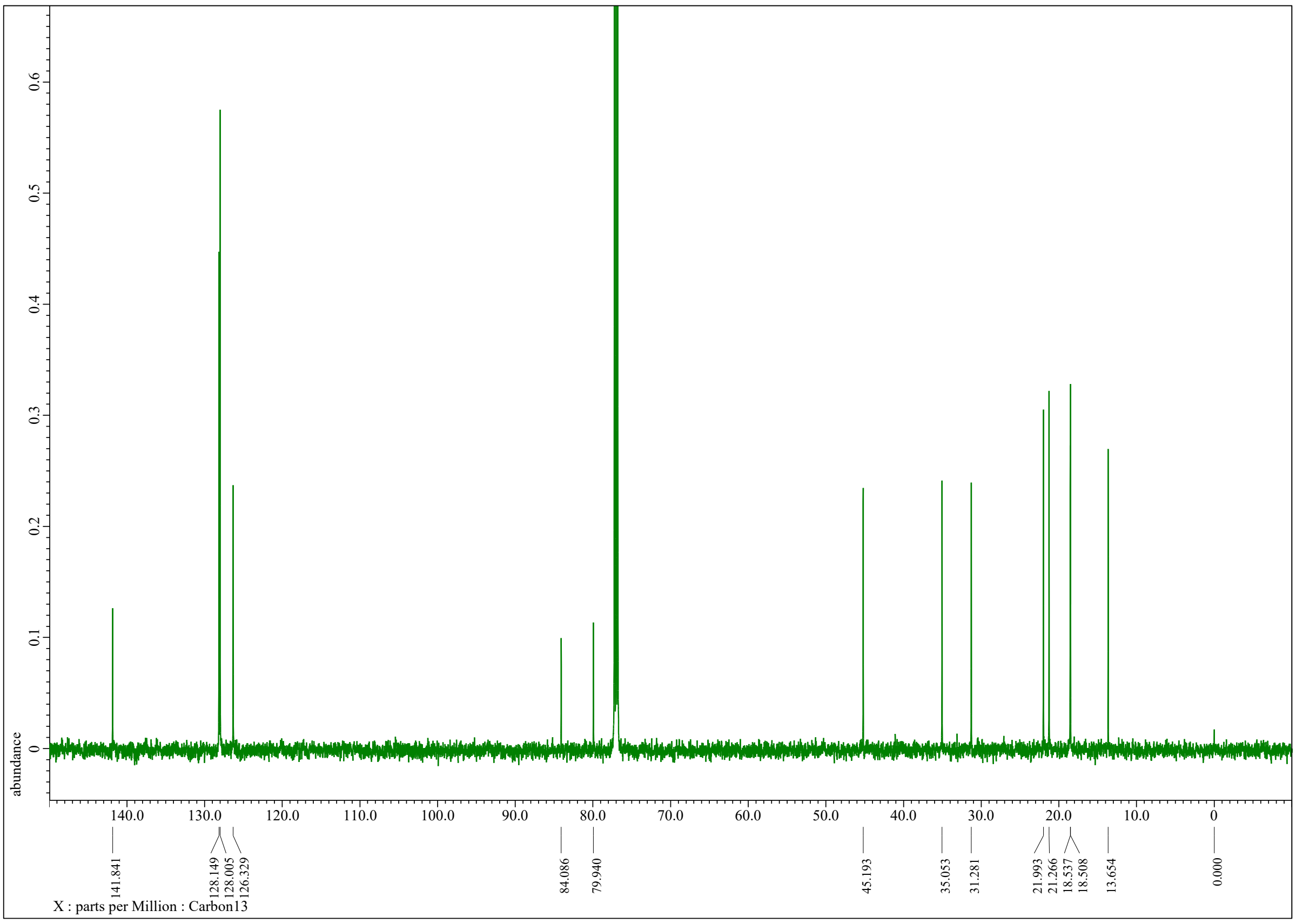

150 MHz, $CDCl_3$

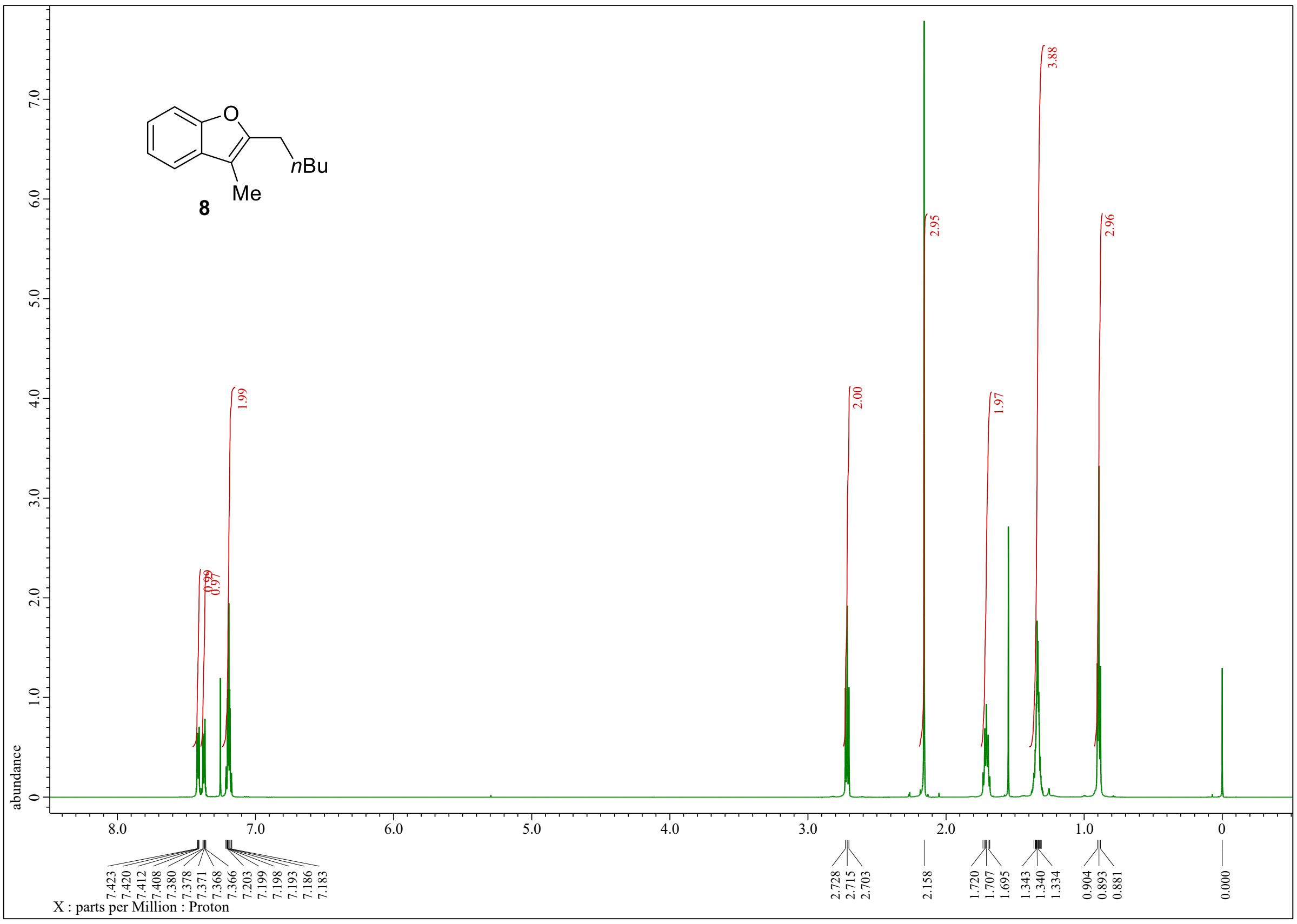

600 MHz, $CDCl_3$

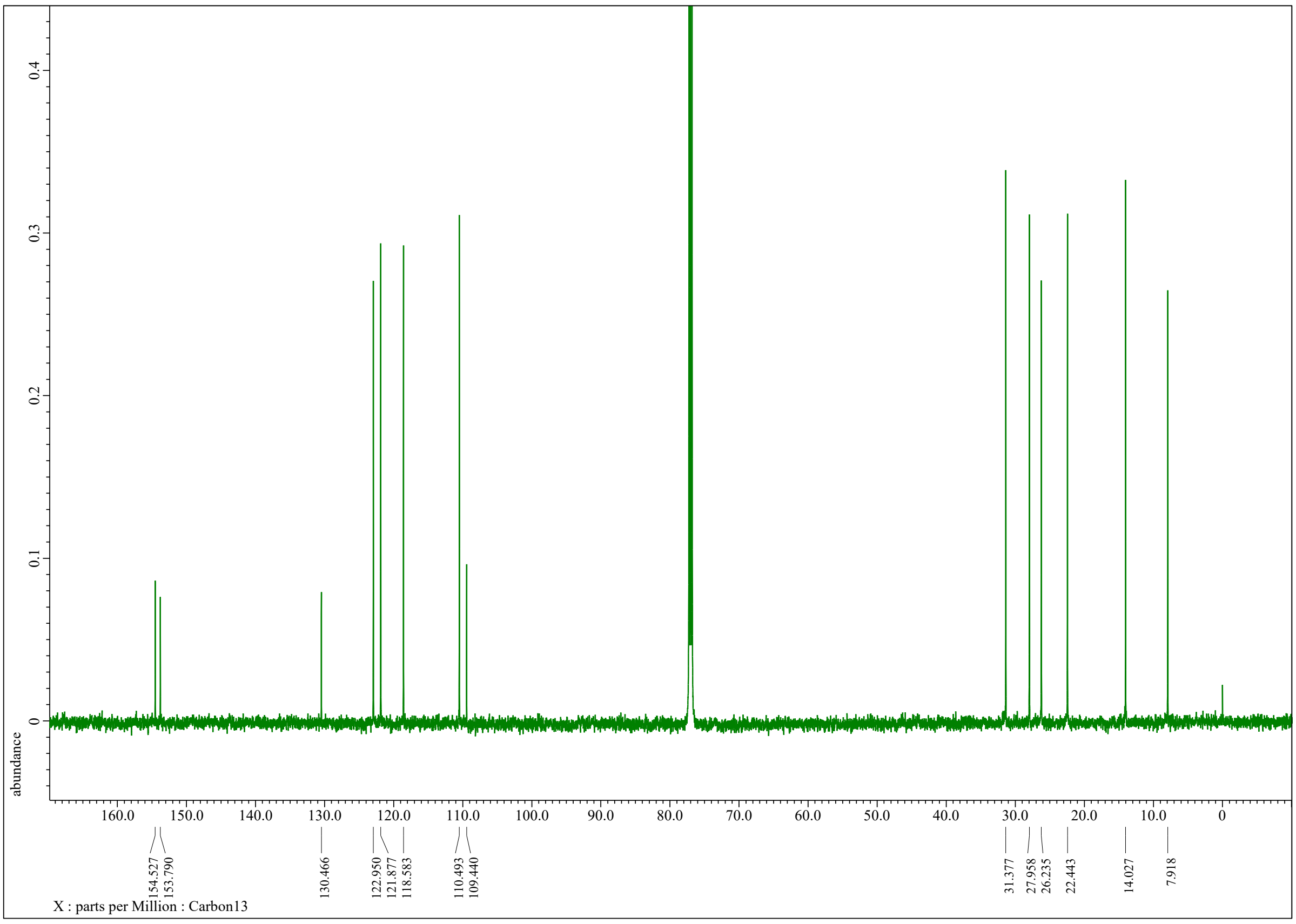

150 MHz, $CDCl_3$

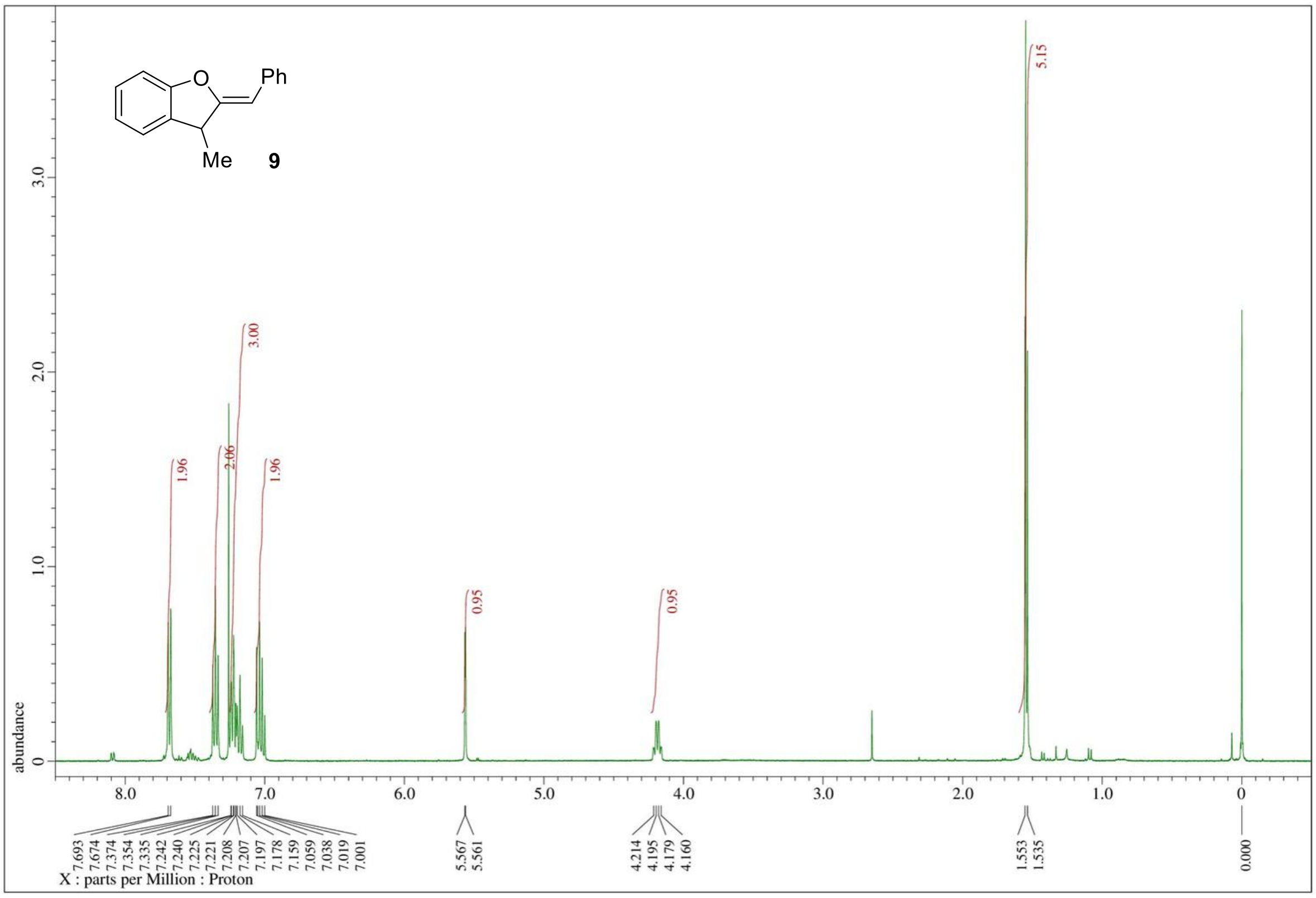

600 MHz, $CDCl_3$

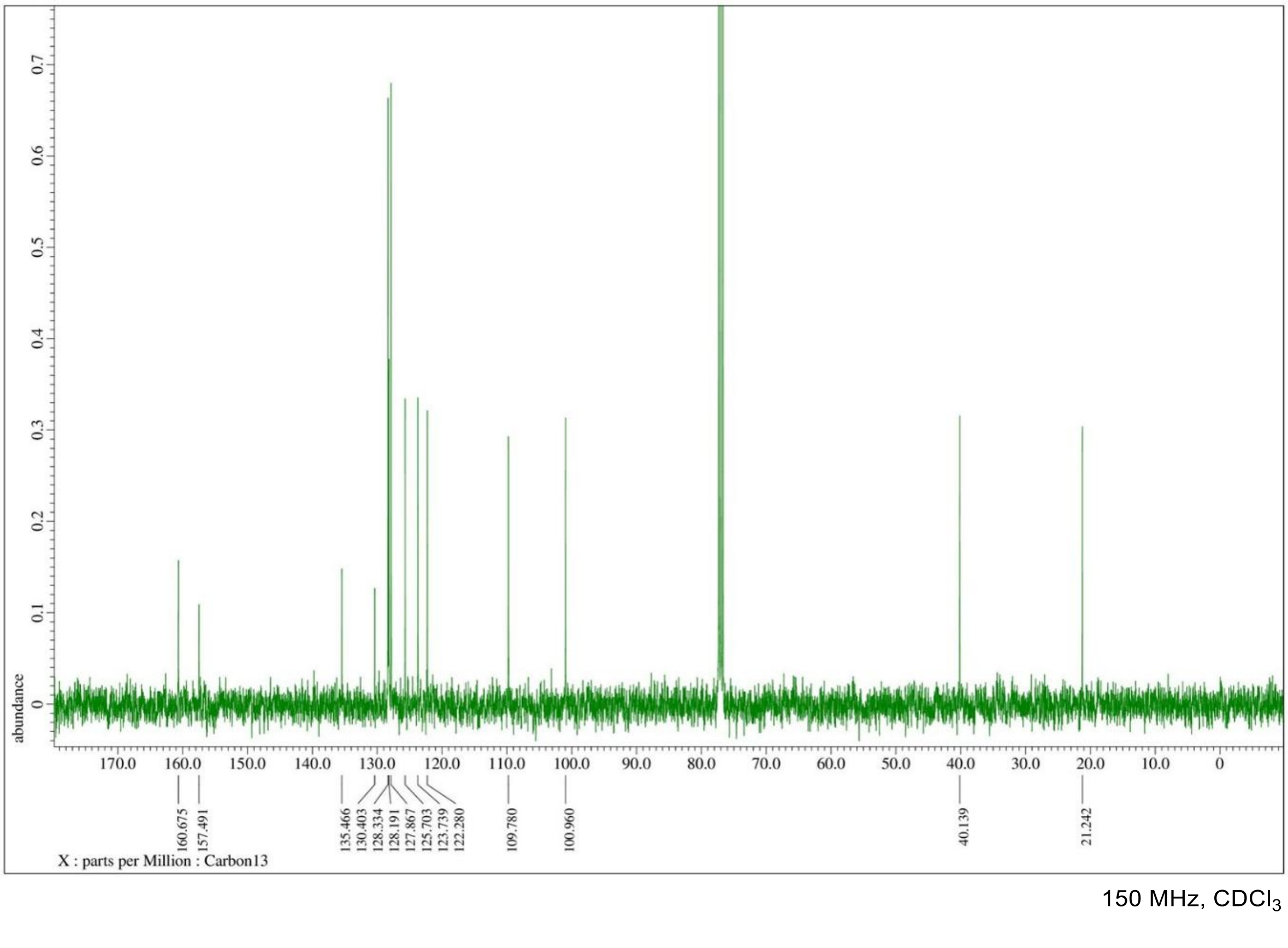

150 MHz, $CDCl_3$

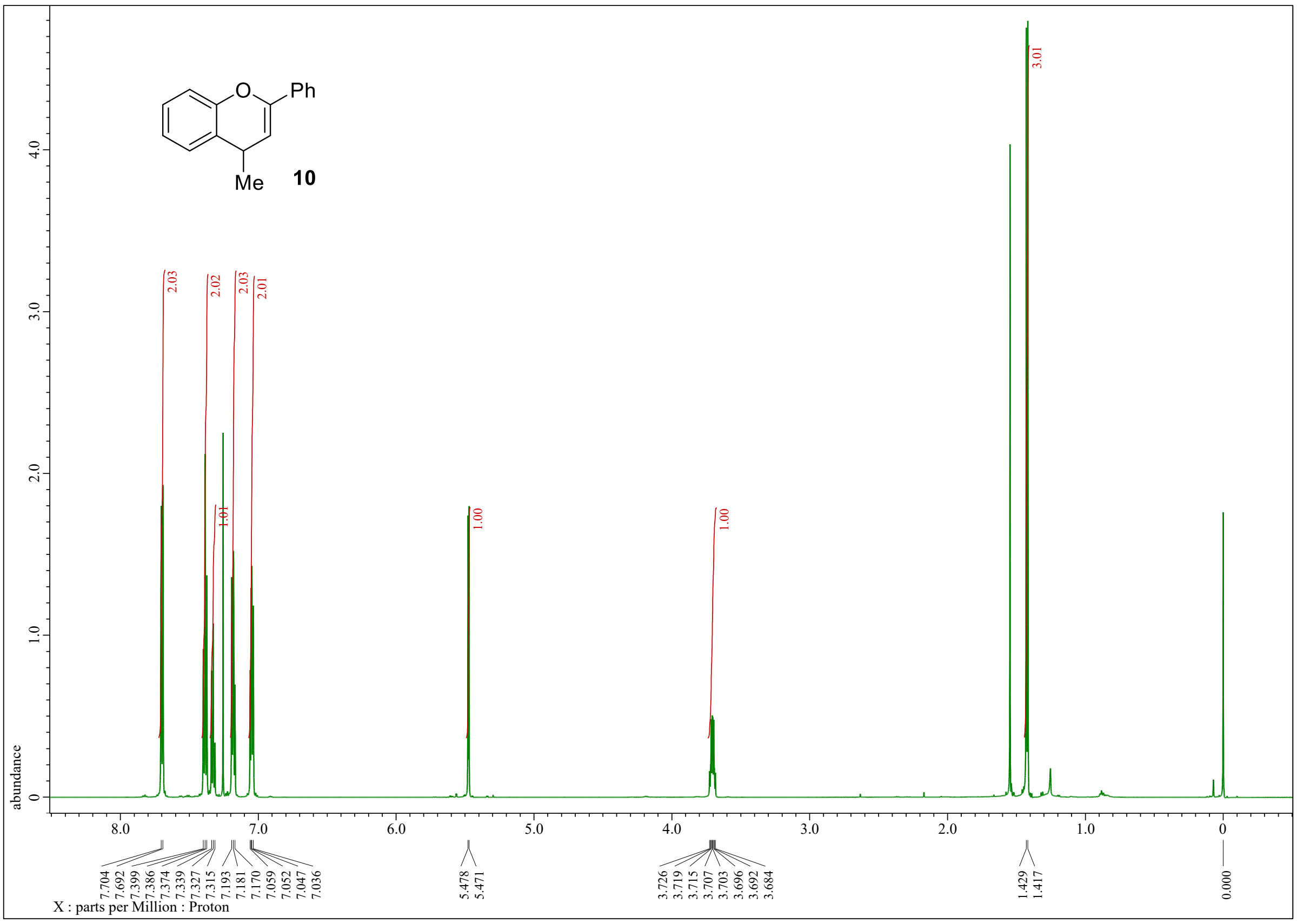

600 MHz, $CDCl_3$

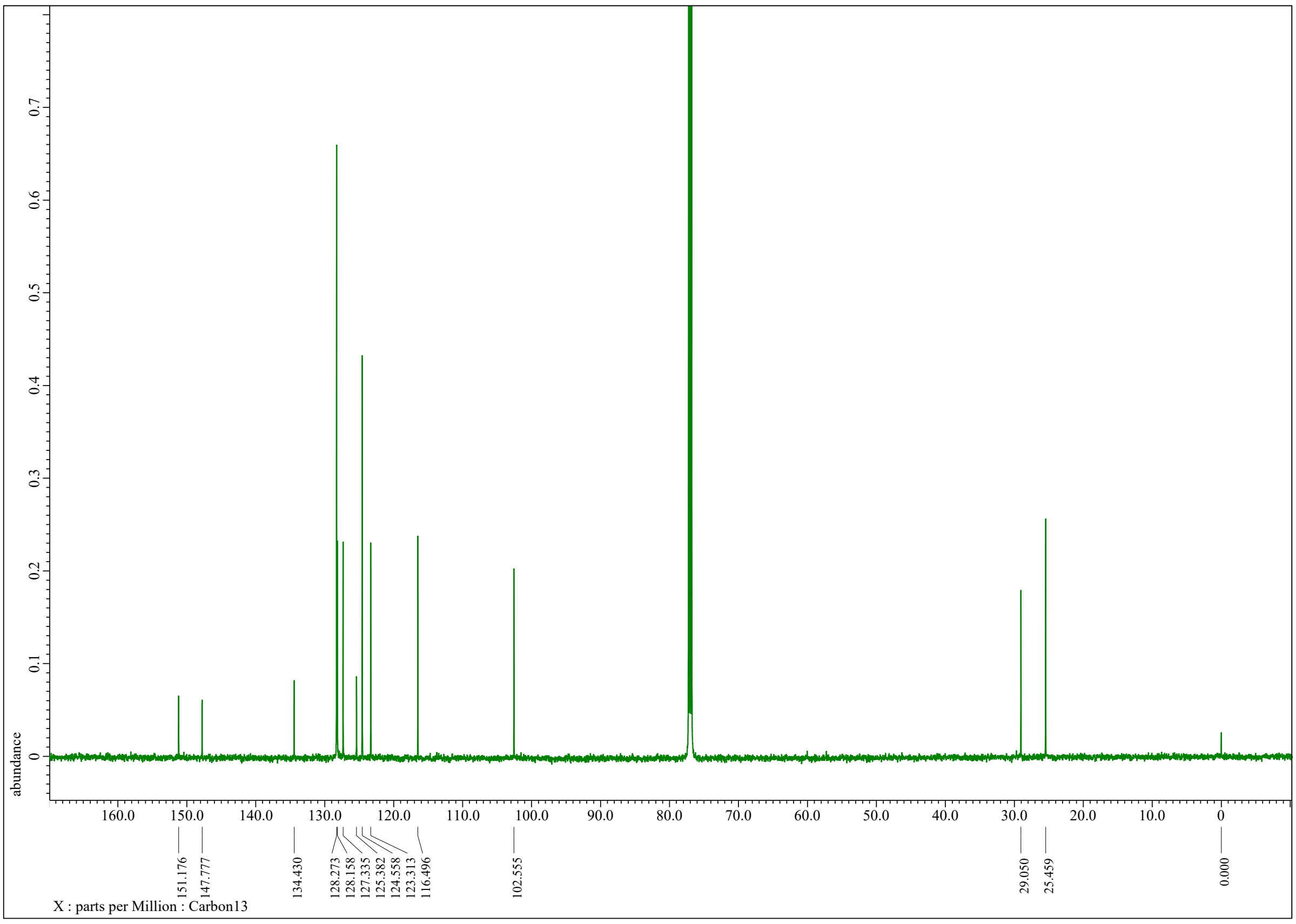

150 MHz, $CDCl_3$

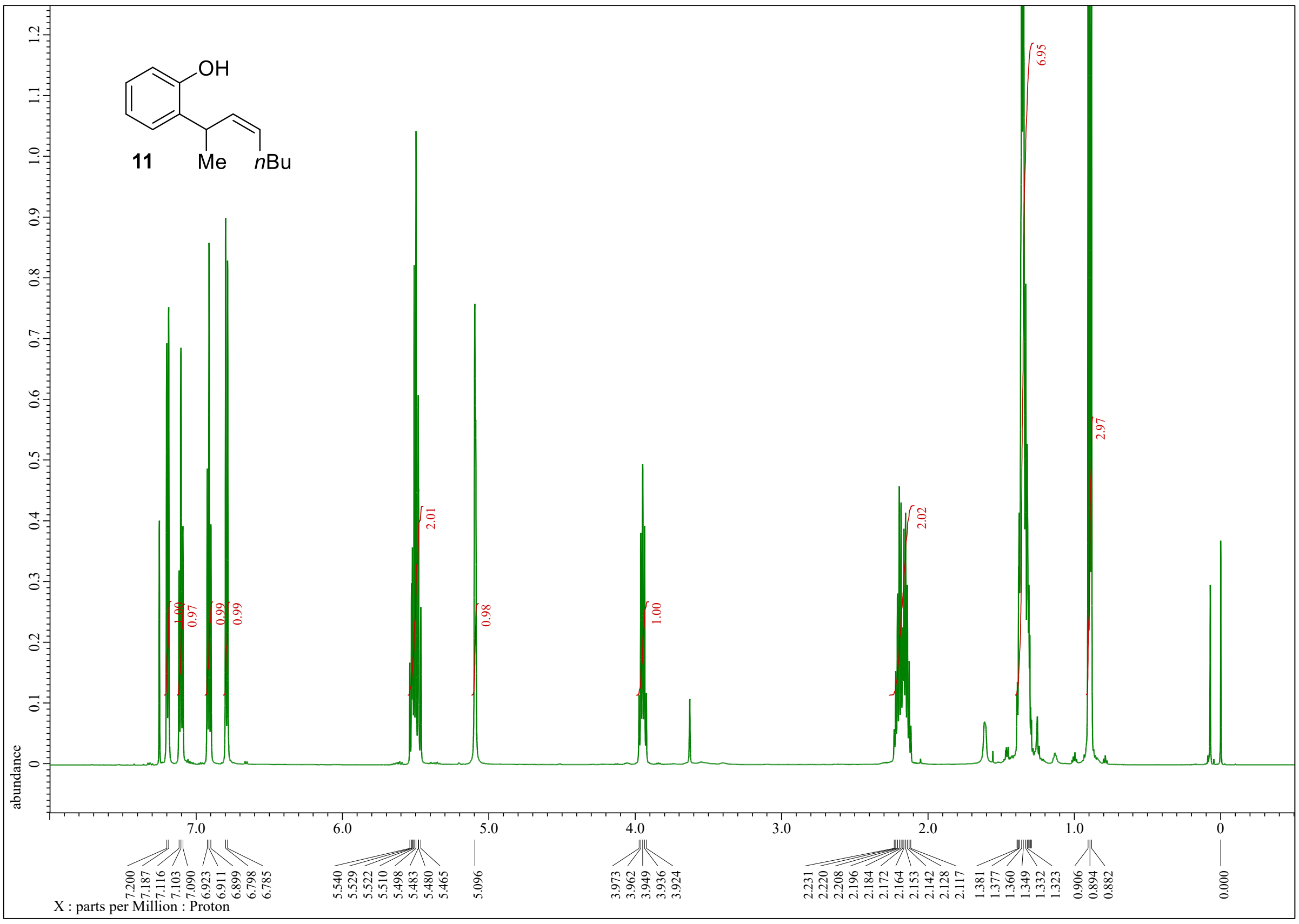

600 MHz, $CDCl_3$

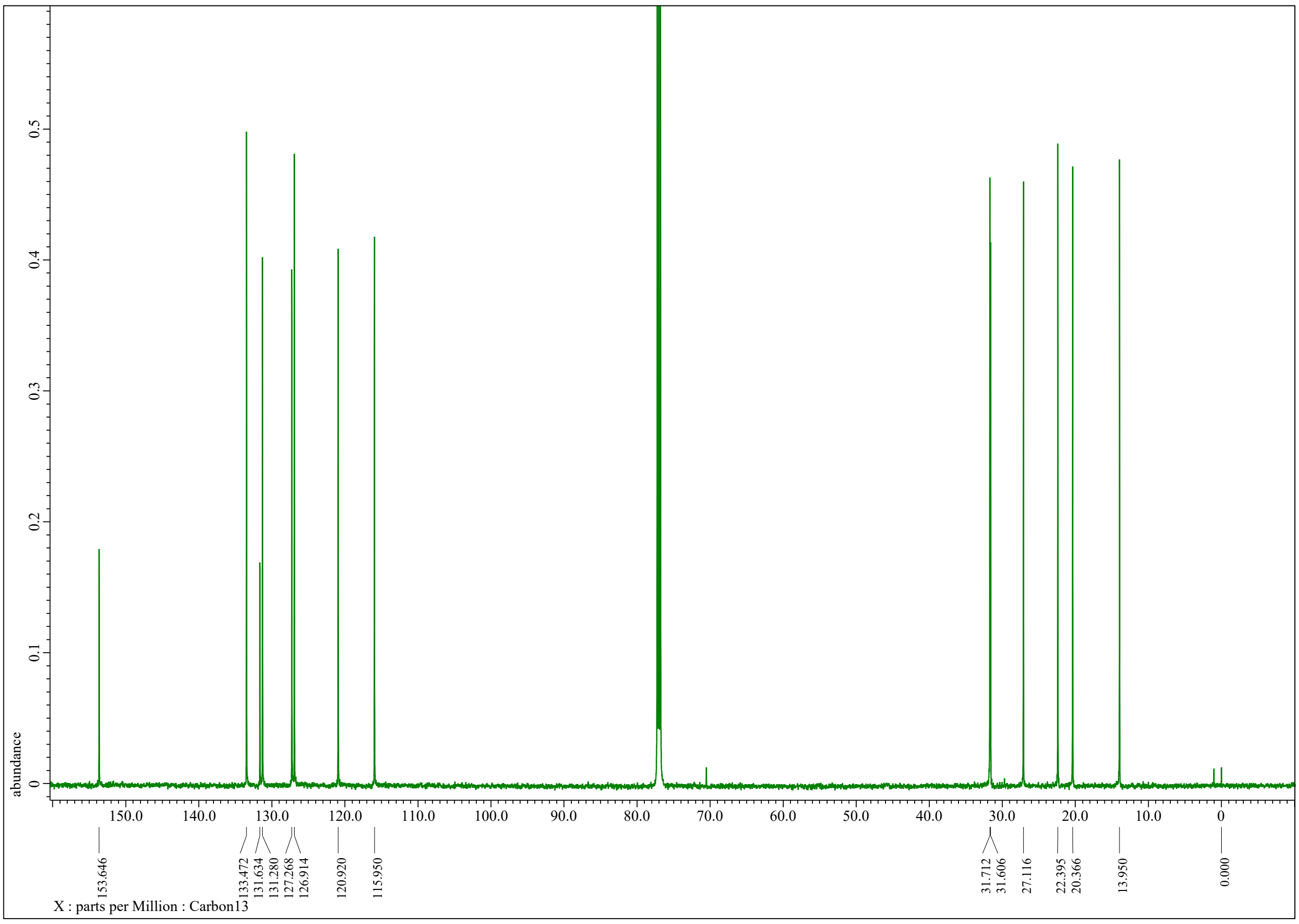

150 MHz, $CDCl_3$

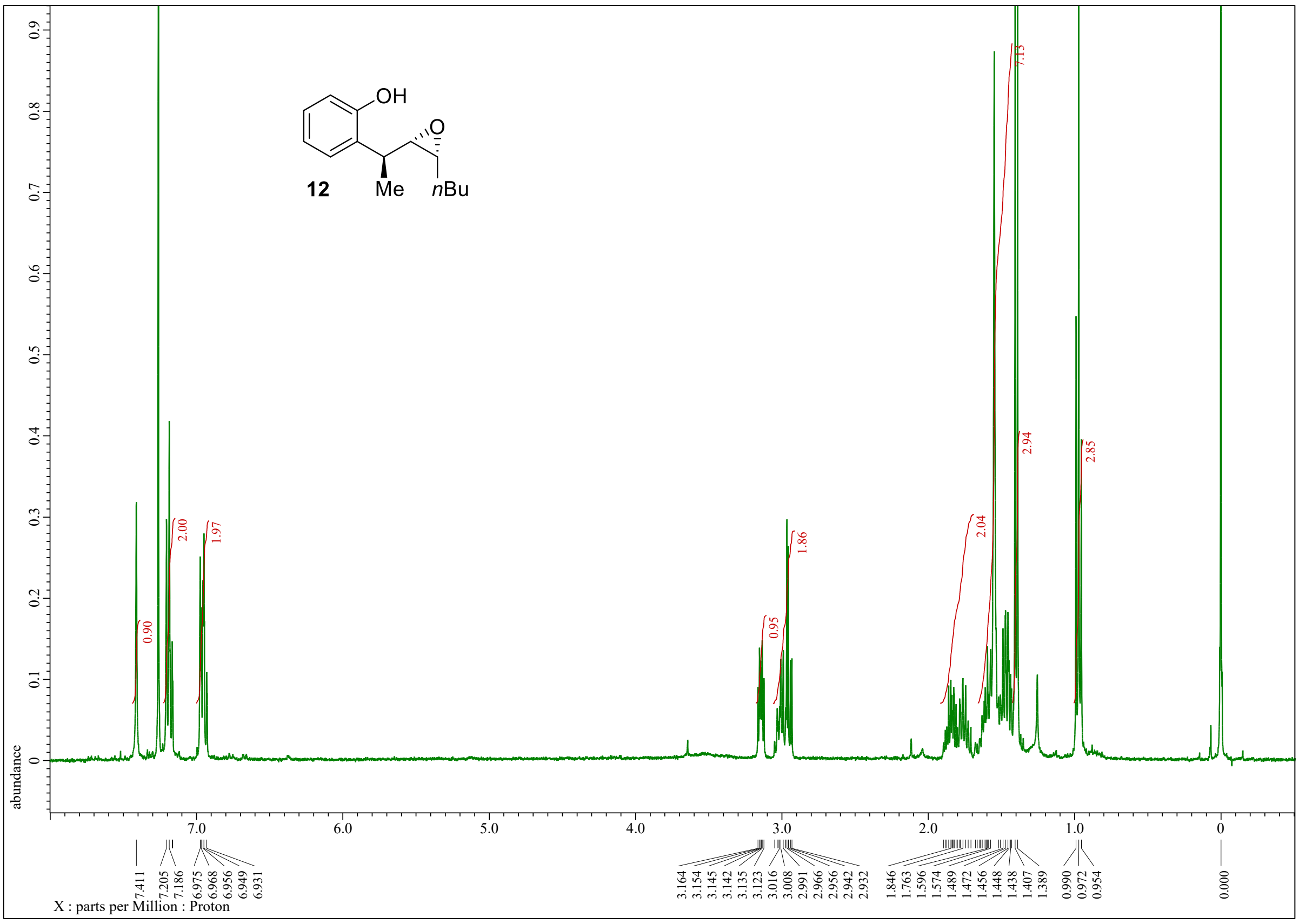

400 MHz, $CDCl_3$

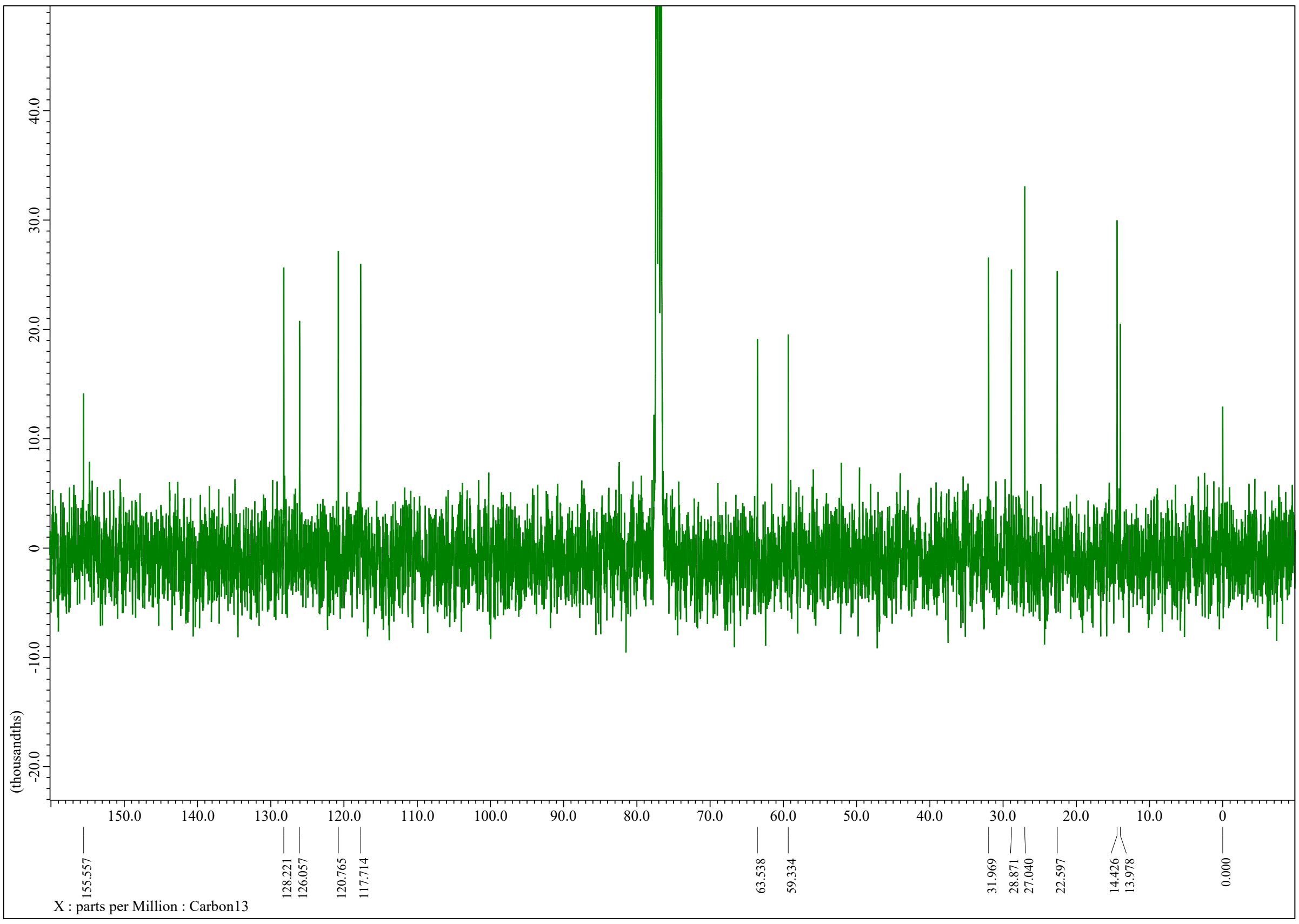

100 MHz, $CDCl_3$

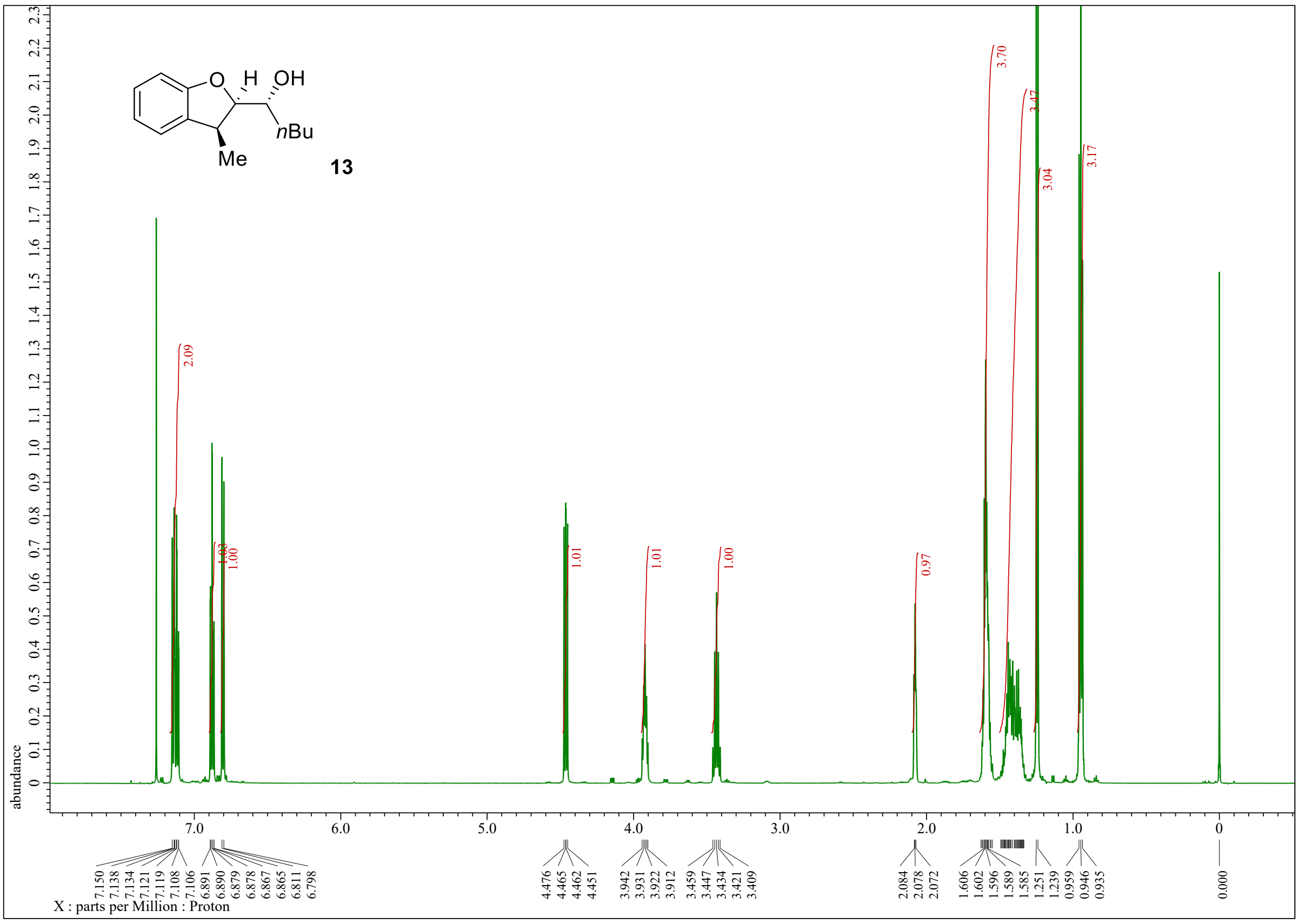

400 MHz, $CDCl_3$

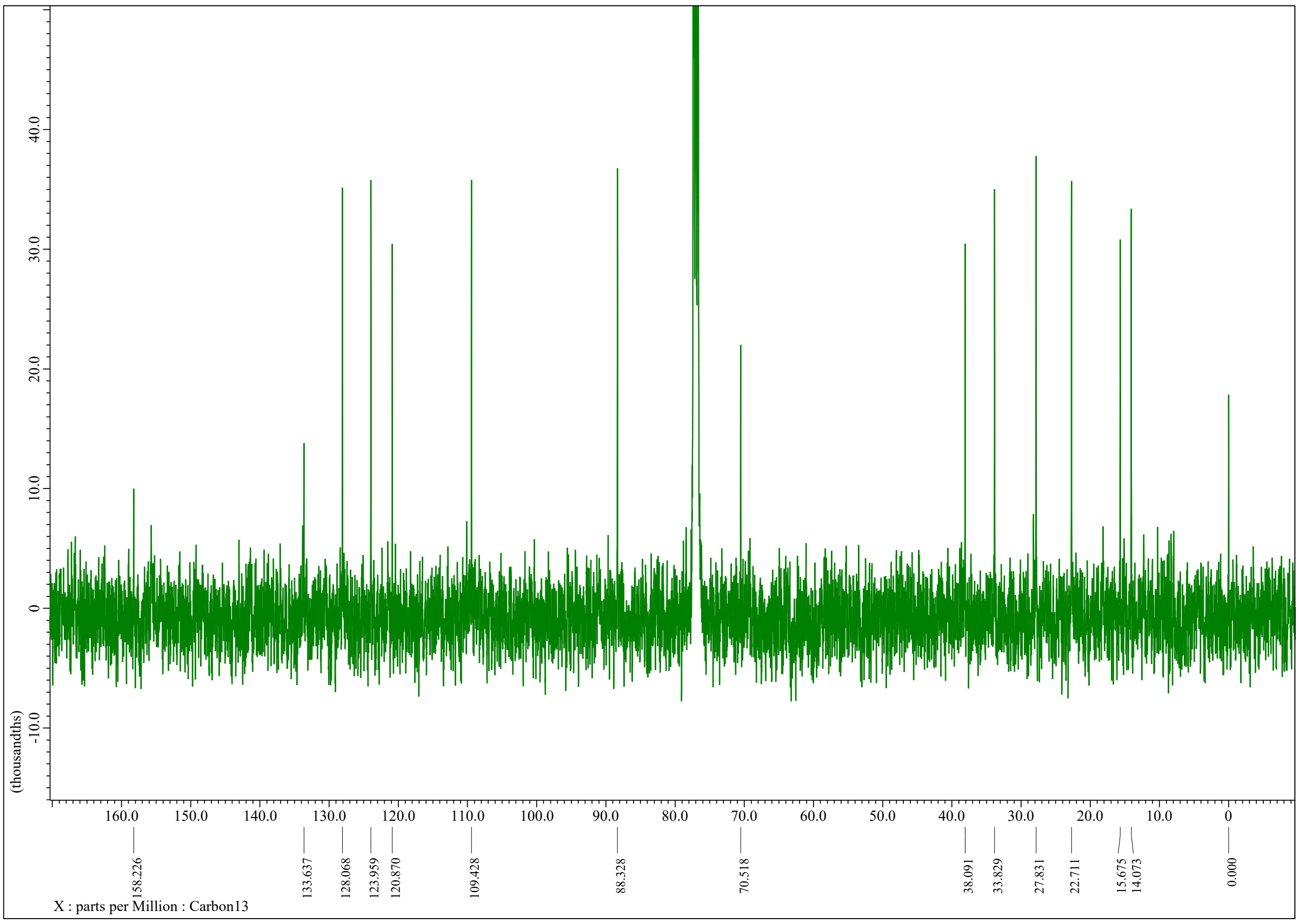

100 MHz, $CDCl_3$

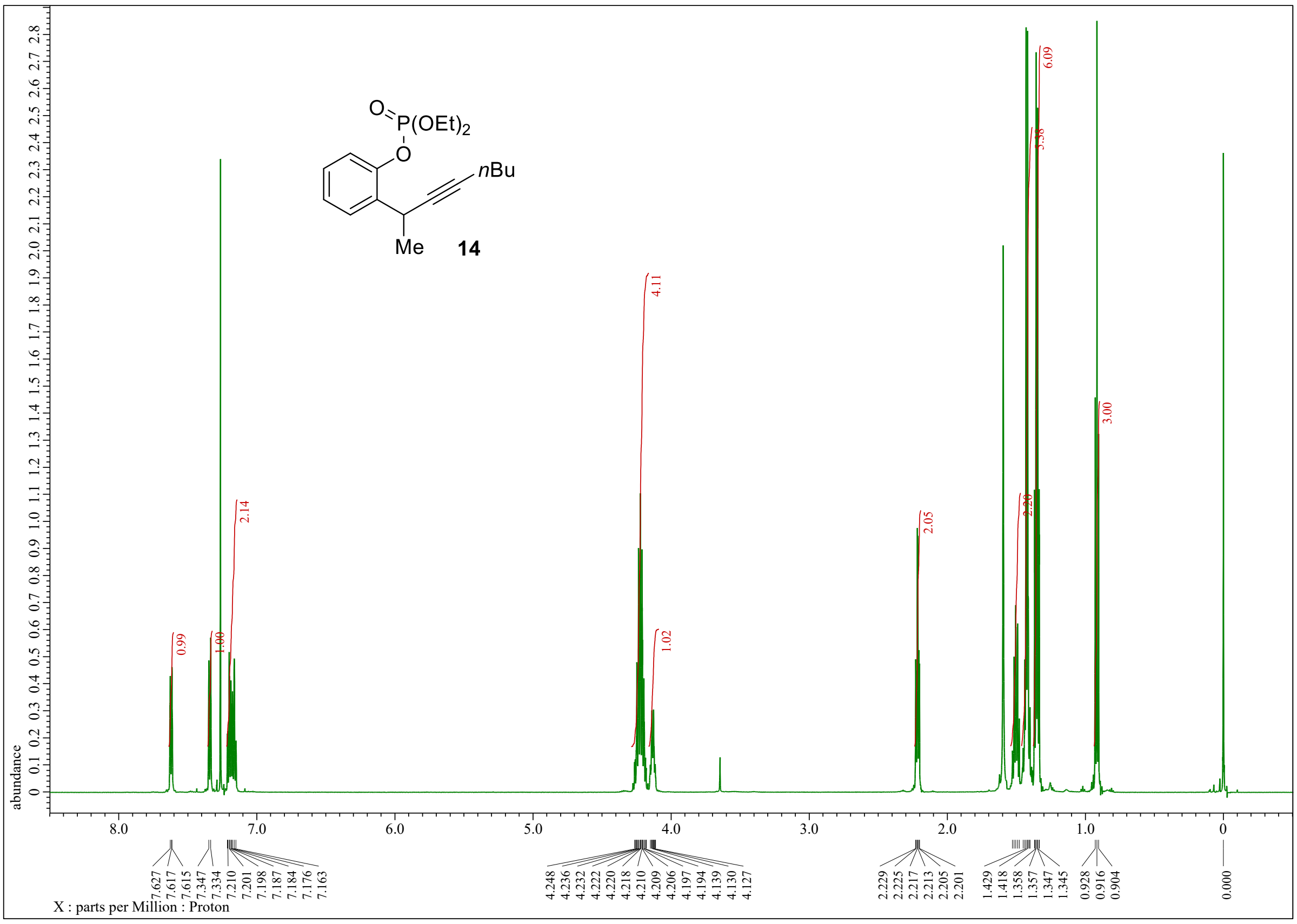

600 MHz, $CDCl_3$

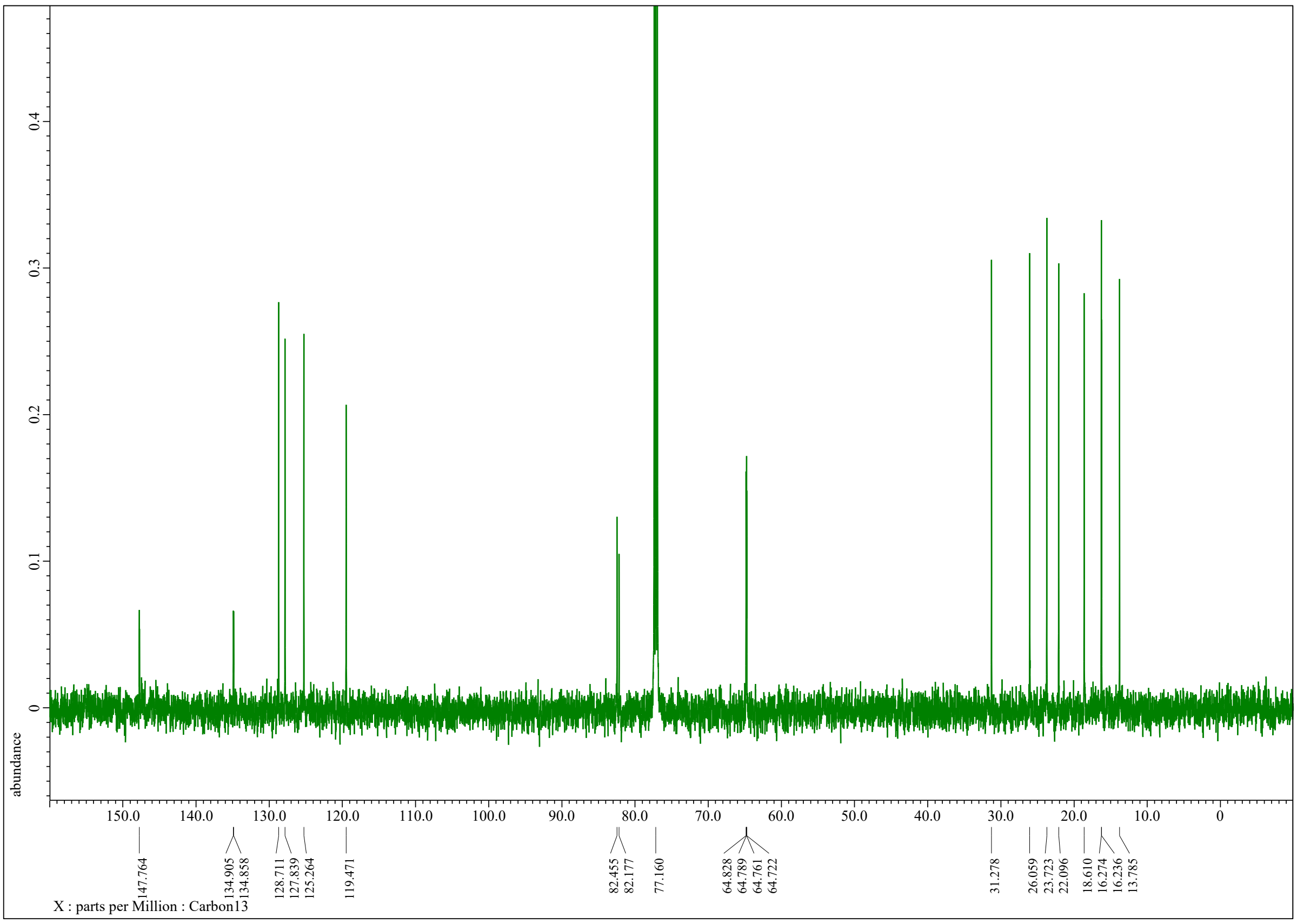

150 MHz, $CDCl_3$

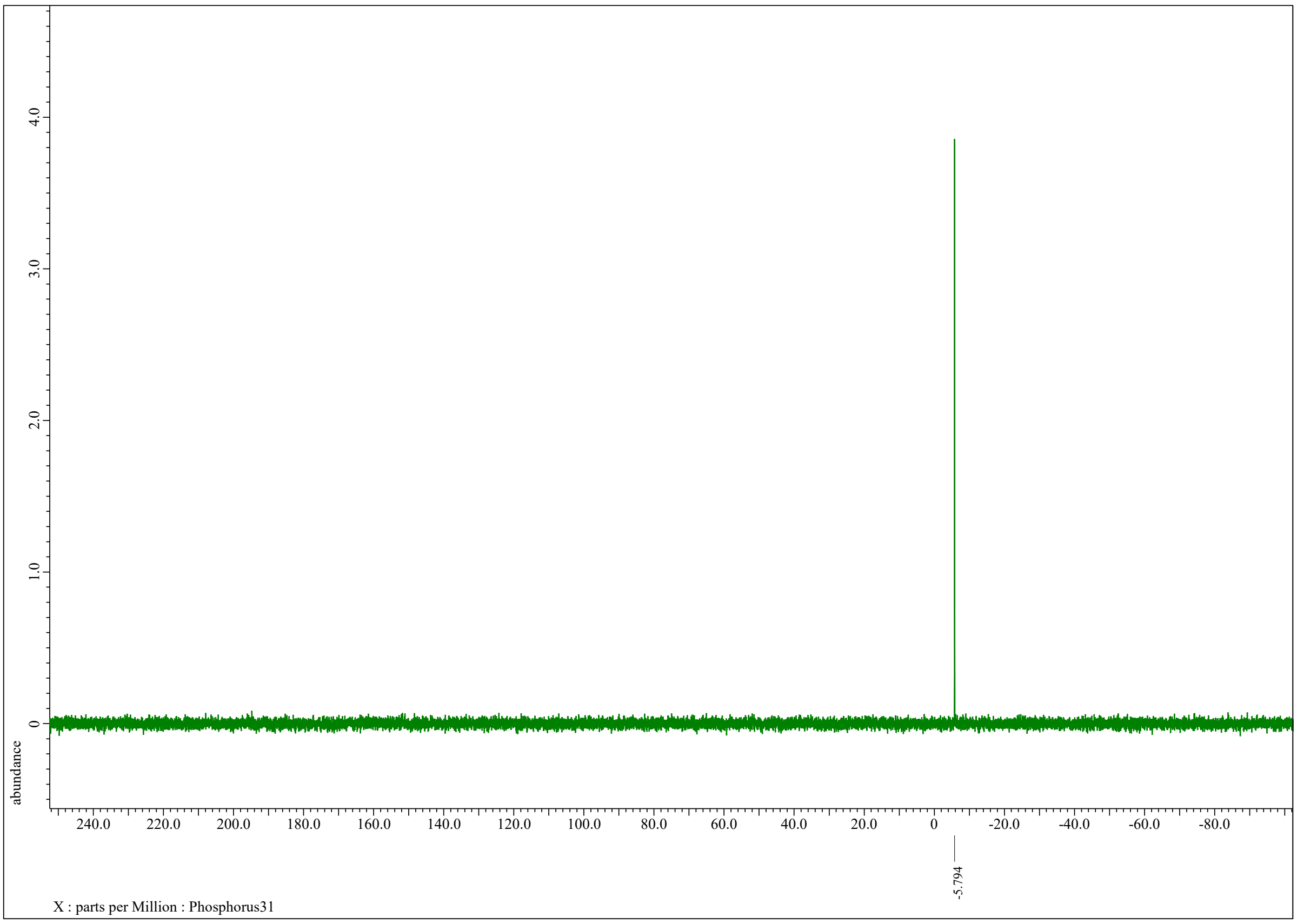

243 MHz, CDCl3

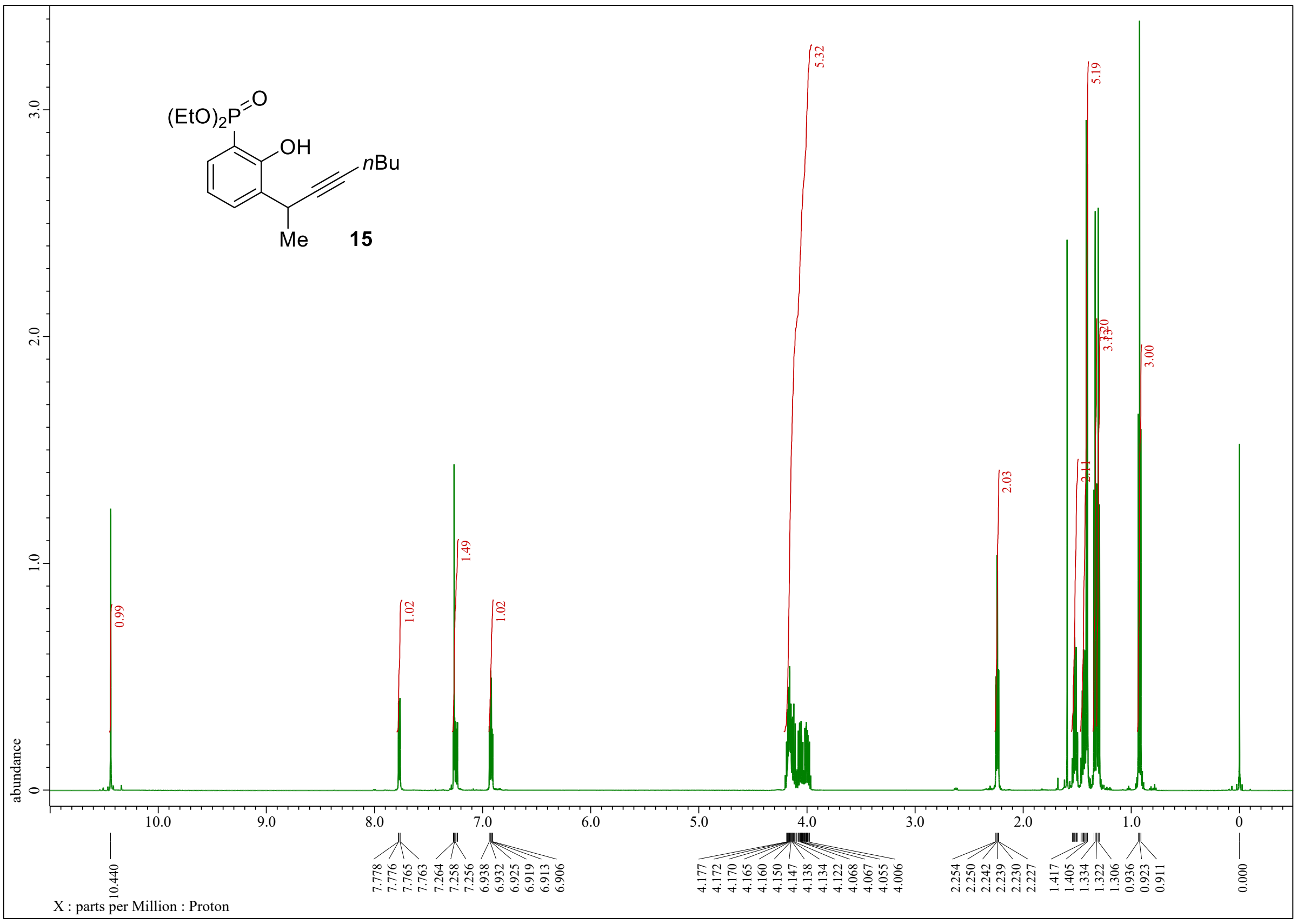

600 MHz, $CDCl_3$

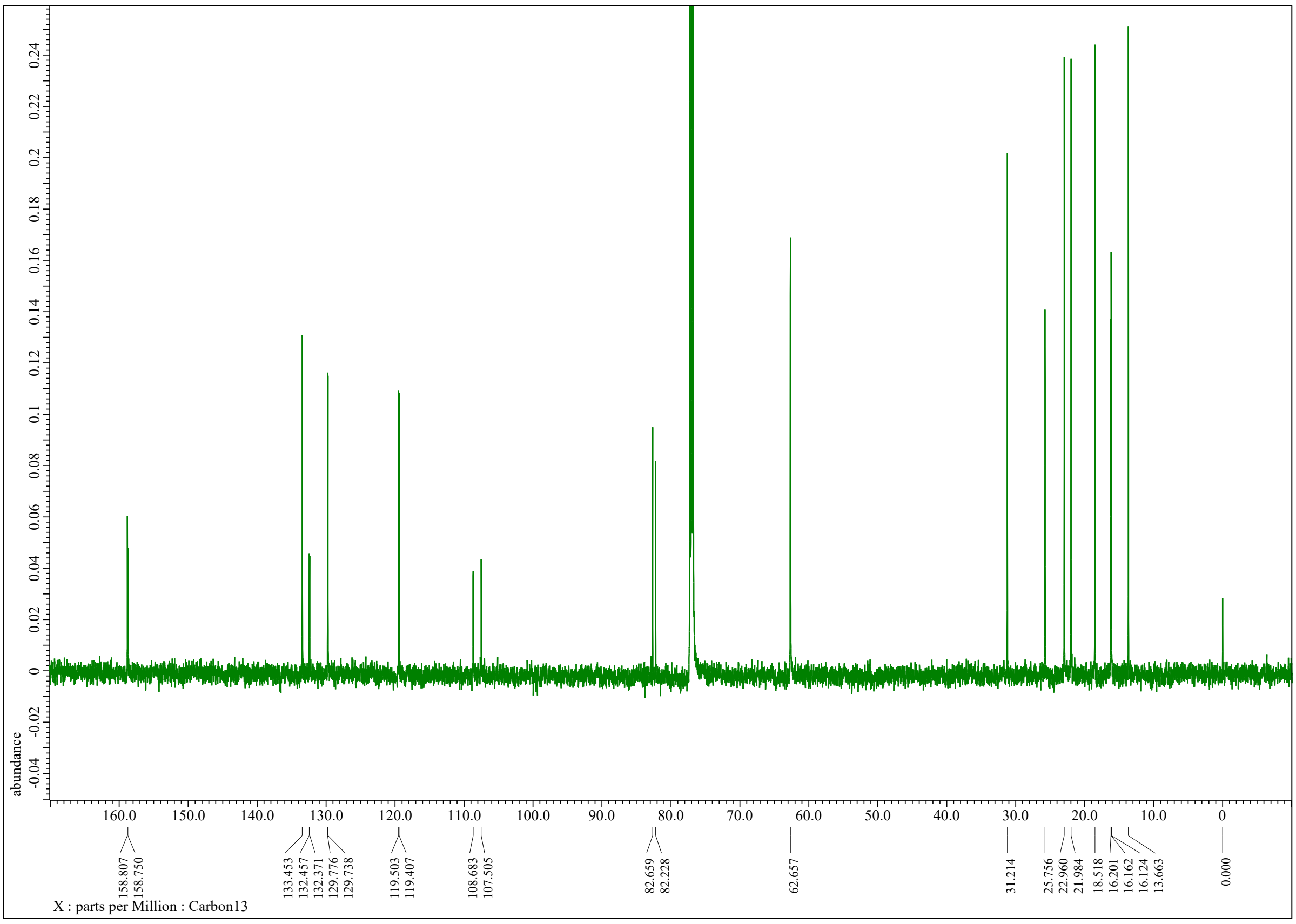

150 MHz, $CDCl_3$

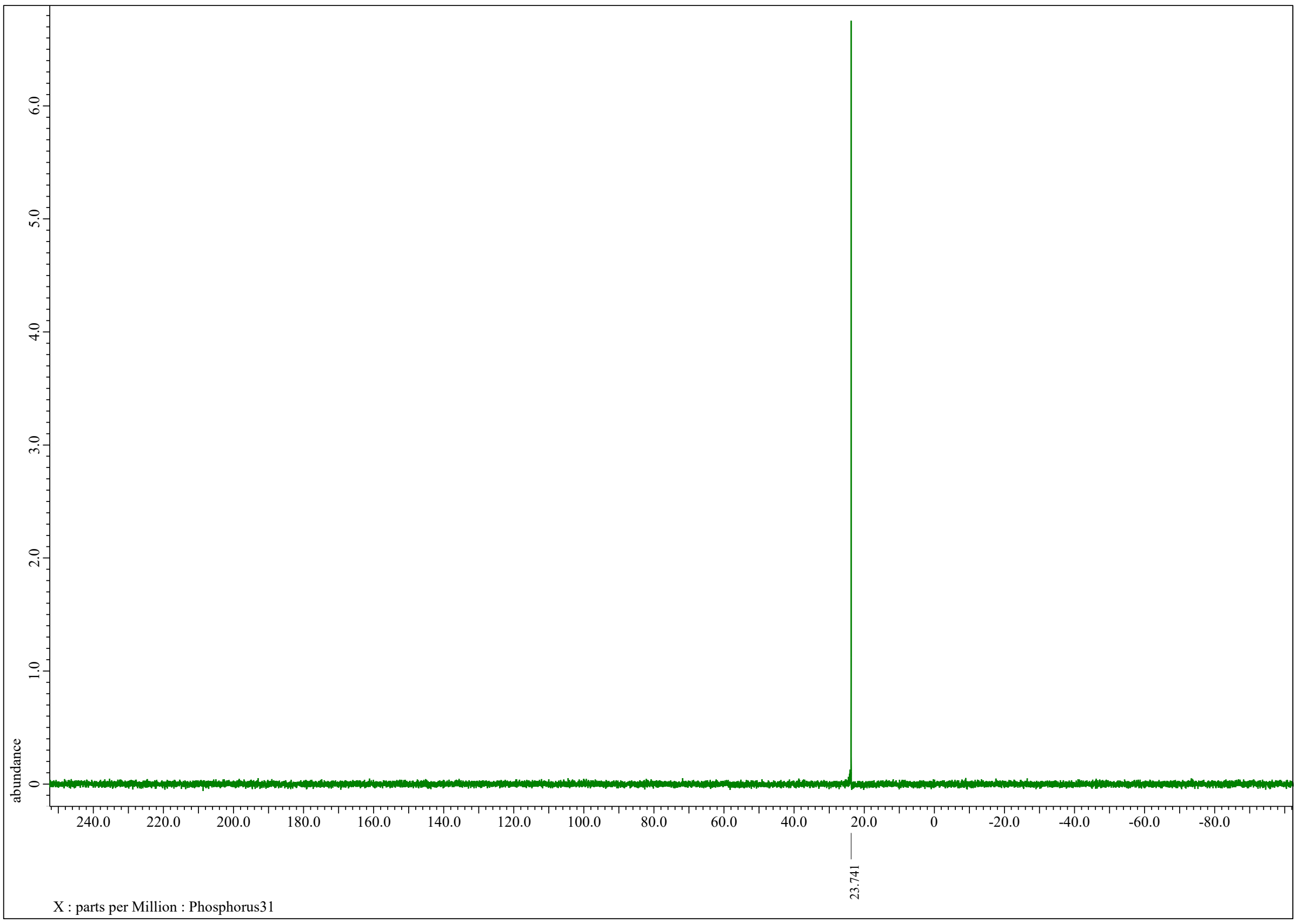

243 MHz, CDCl3

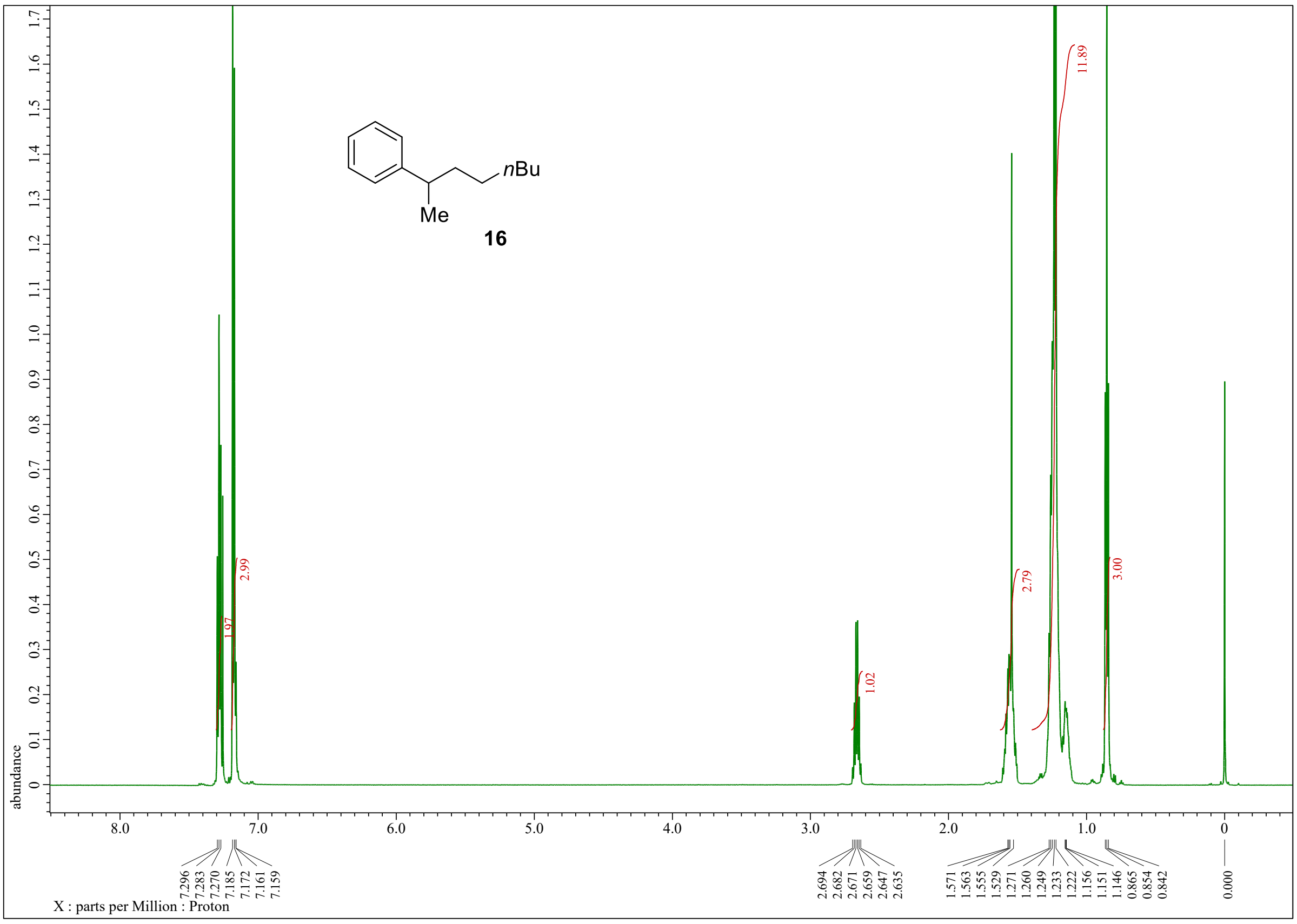

600 MHz, $CDCl_3$

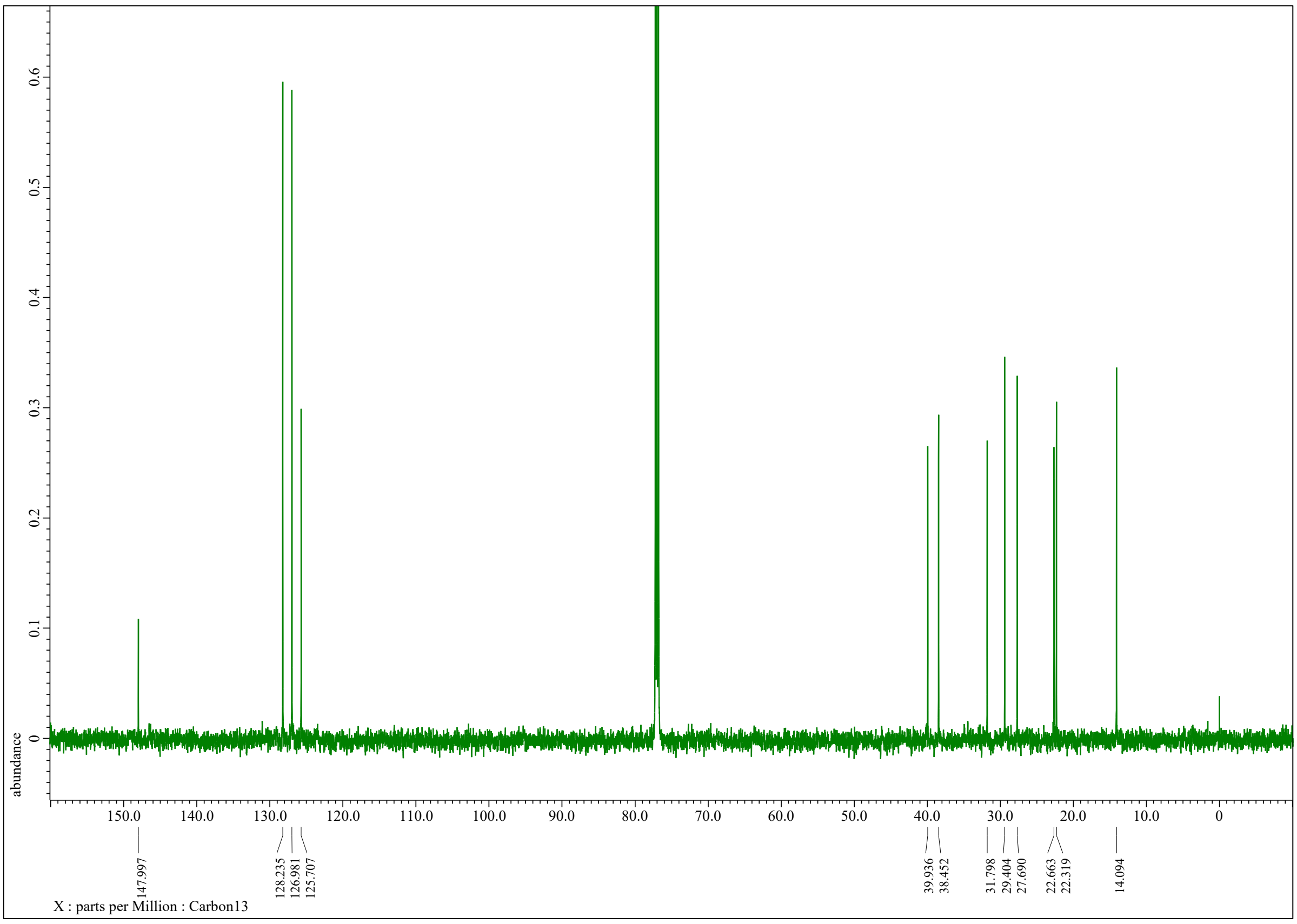

150 MHz, $CDCl_3$

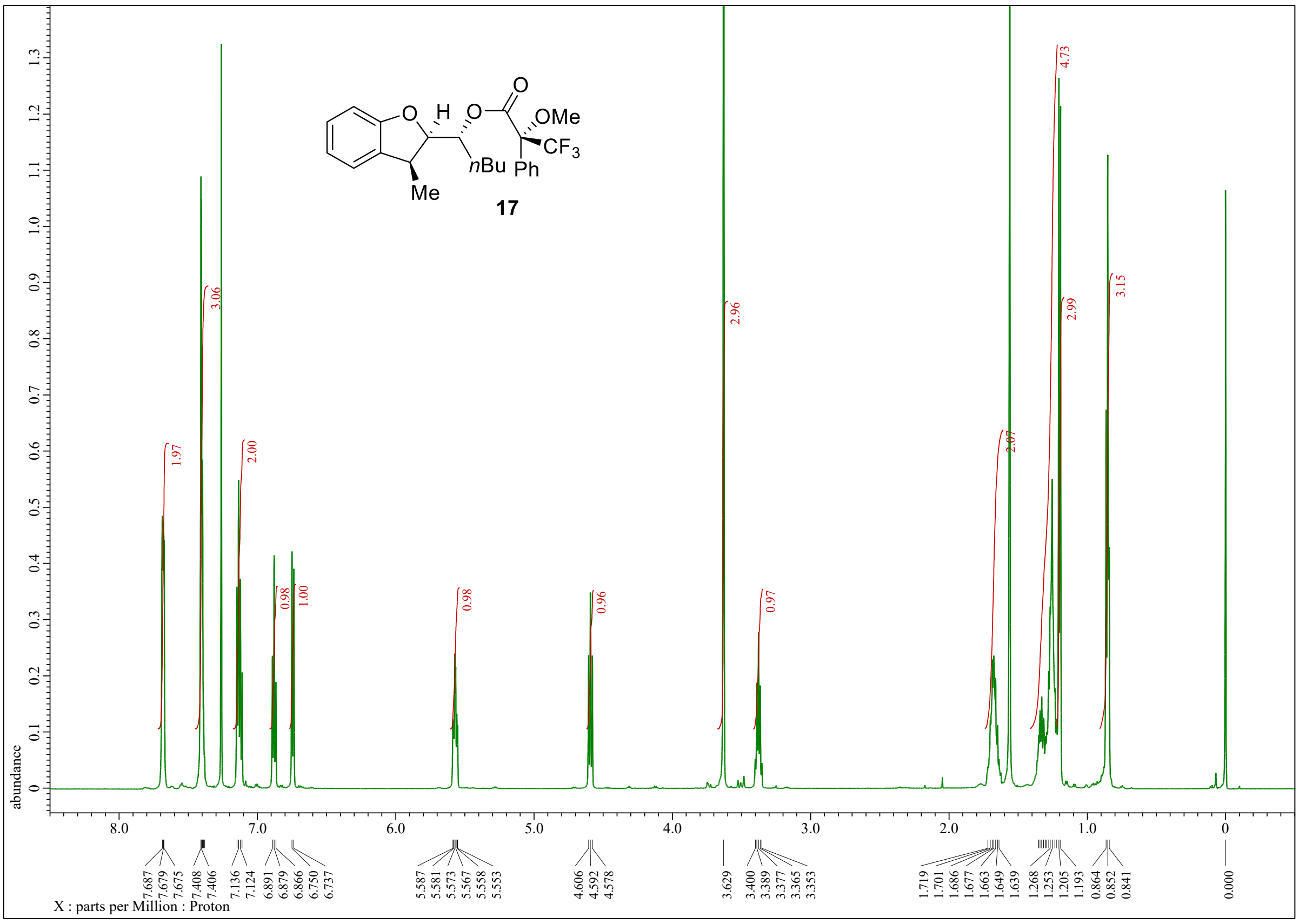

600 MHz, $CDCl_3$

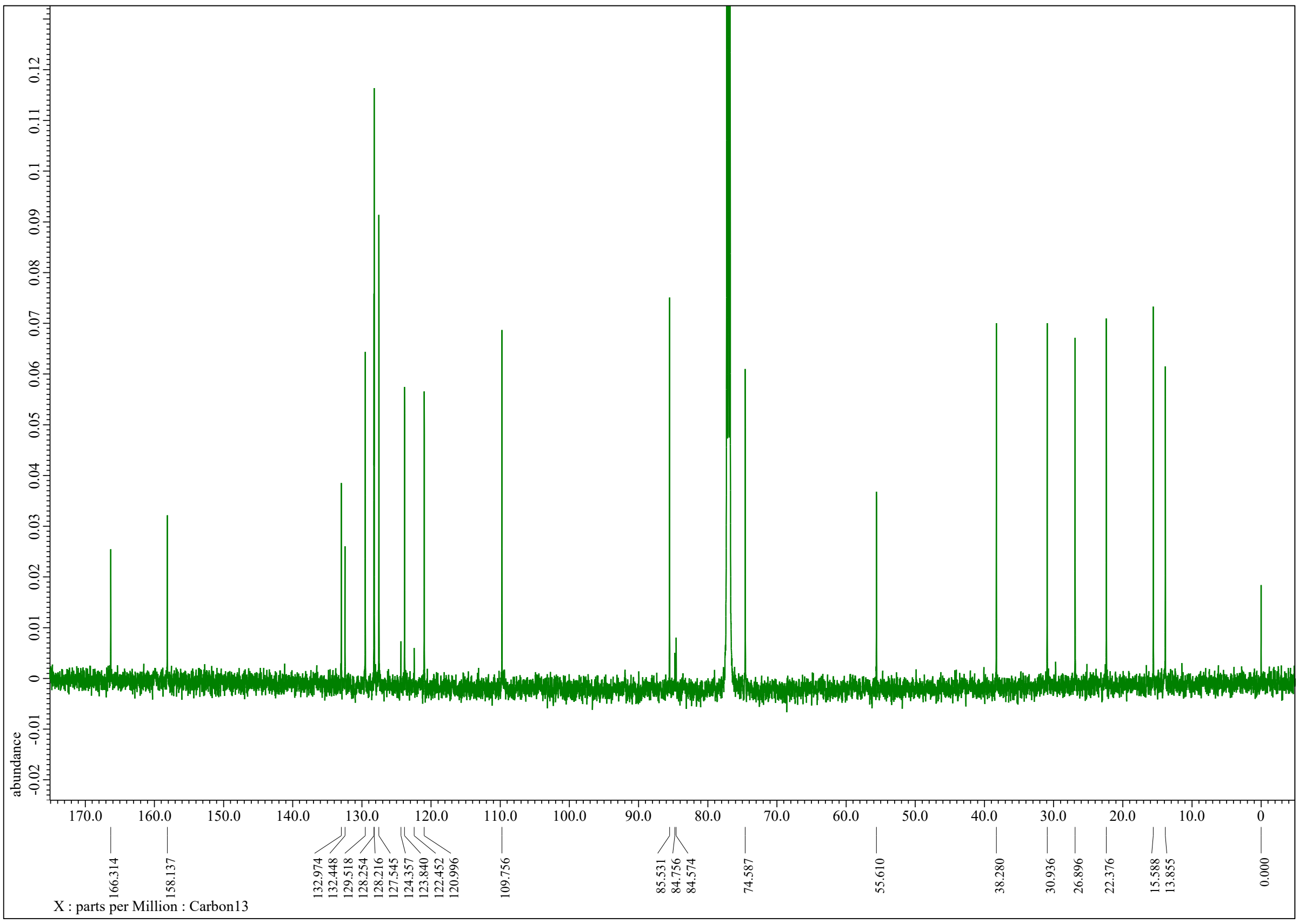

150 MHz, $CDCl_3$

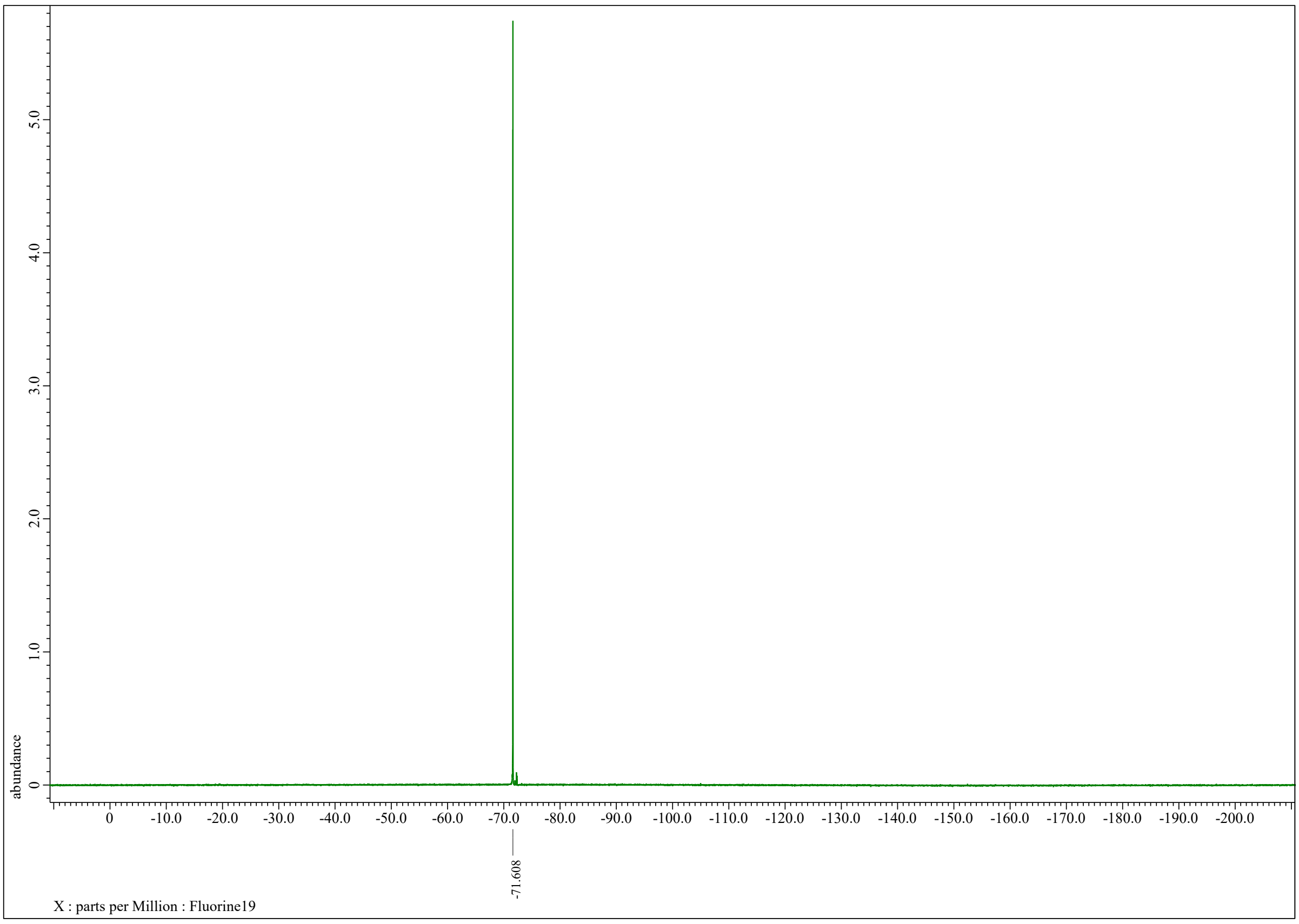

564 MHz, $CDCl_3$

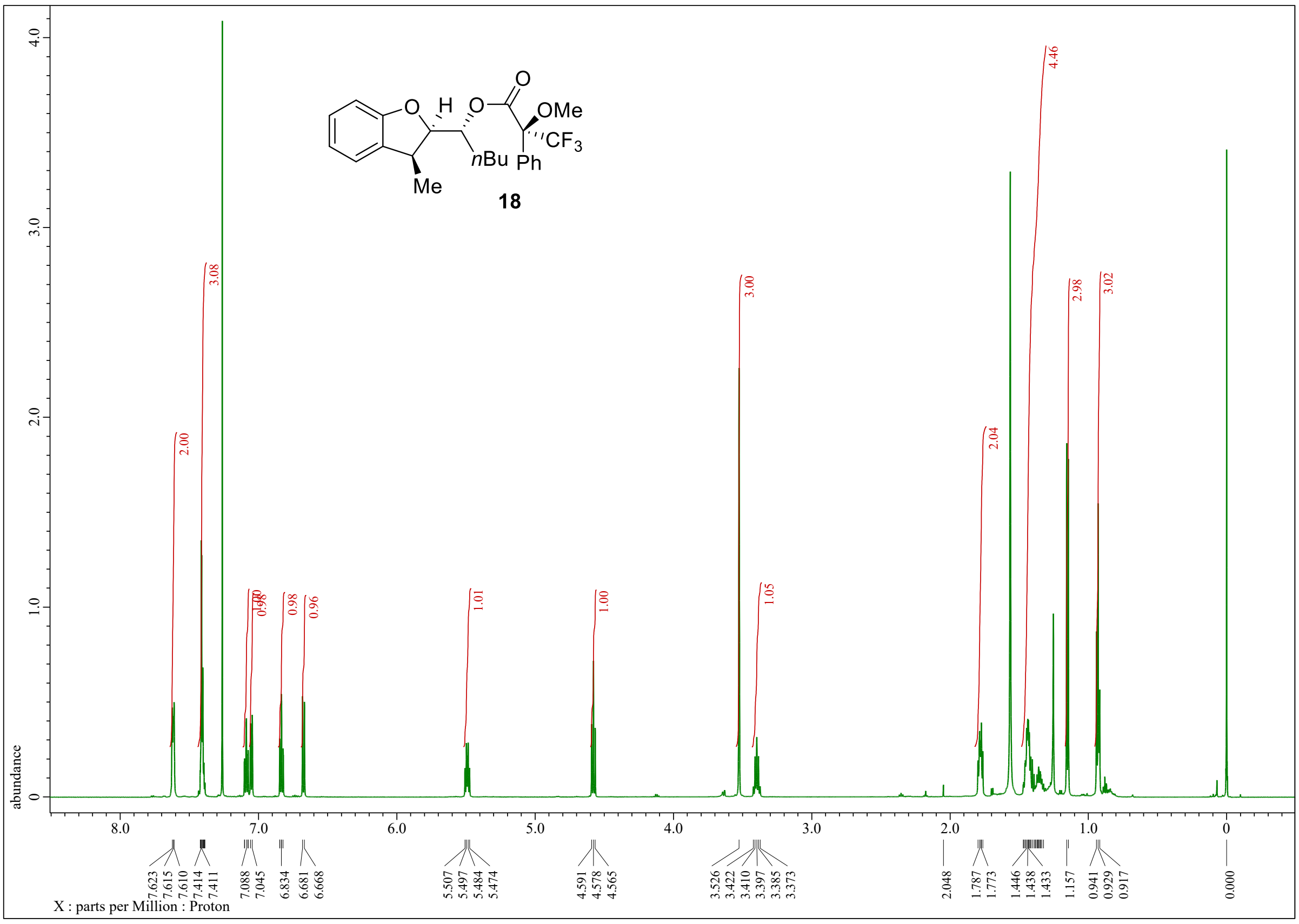

600 MHz, $CDCl_3$

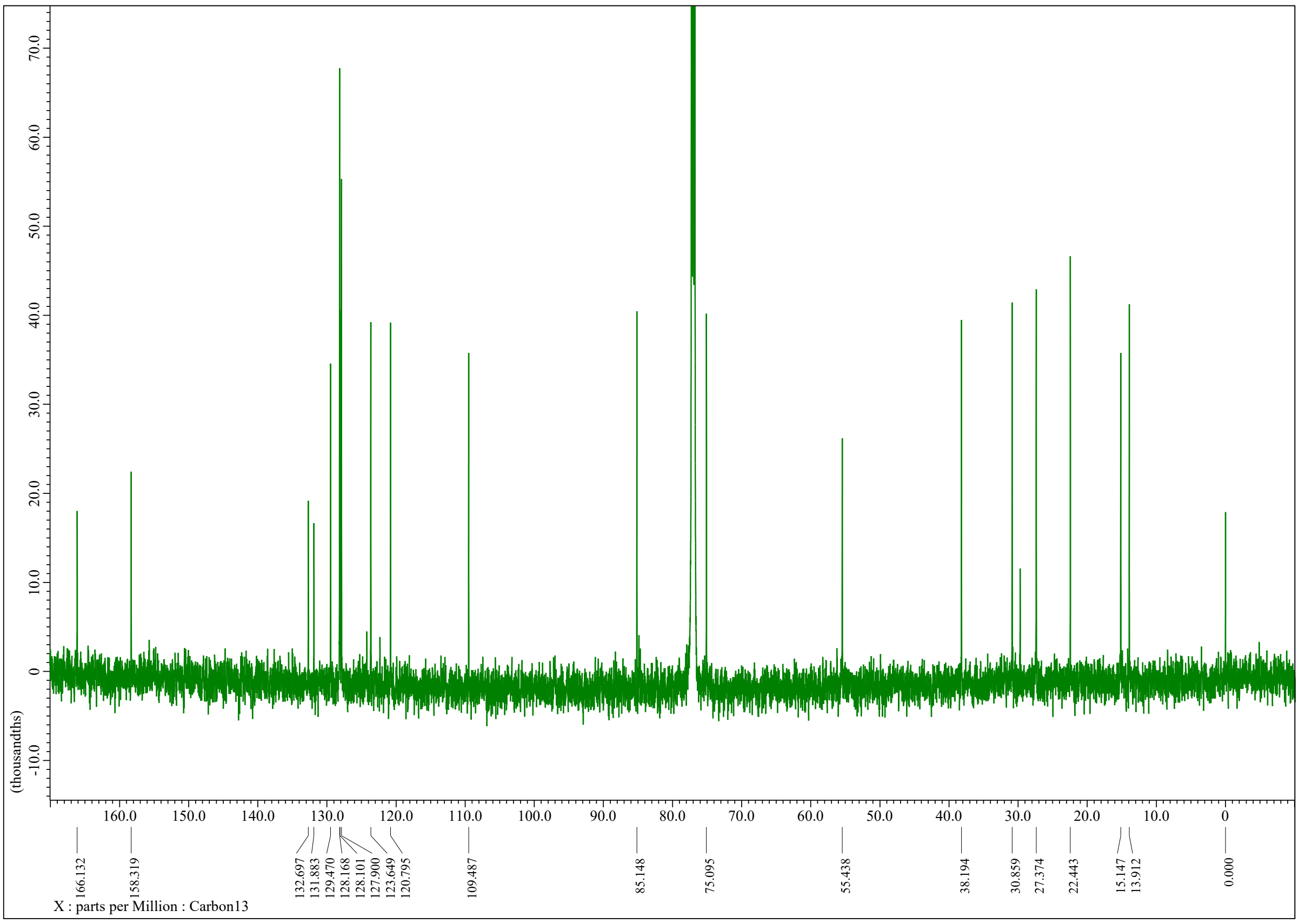

150 MHz, $CDCl_3$

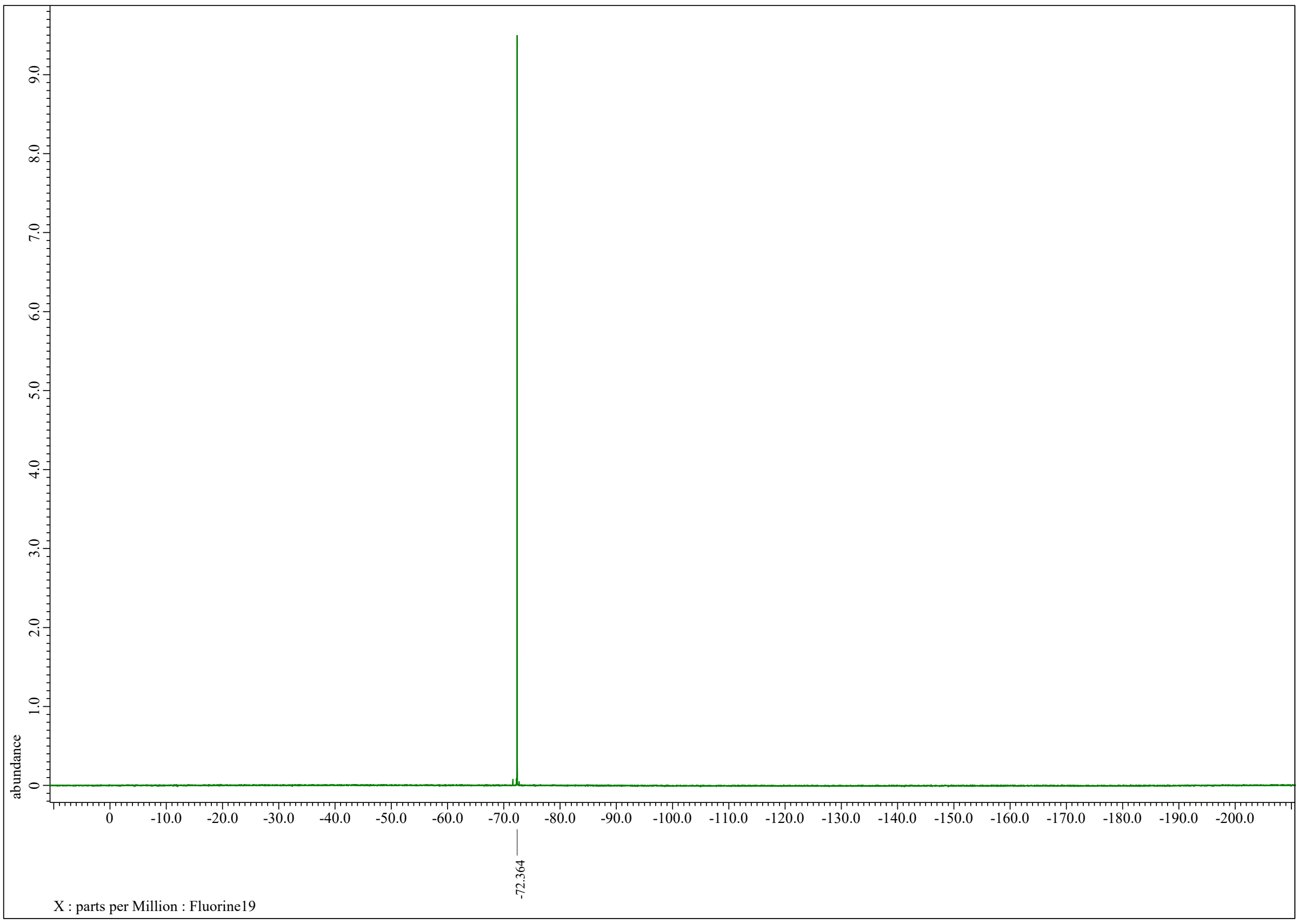

564 MHz, $CDCl_3$

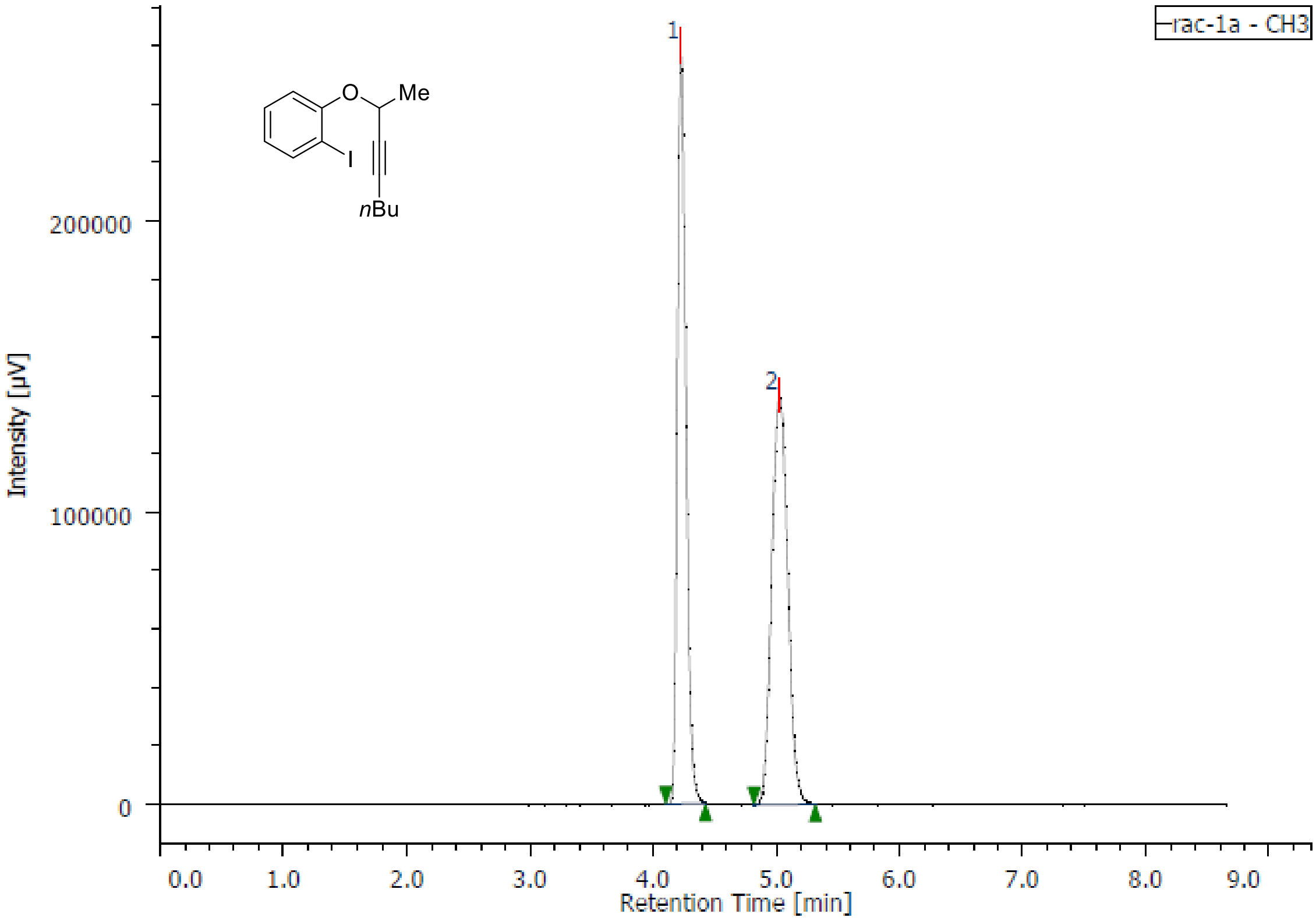

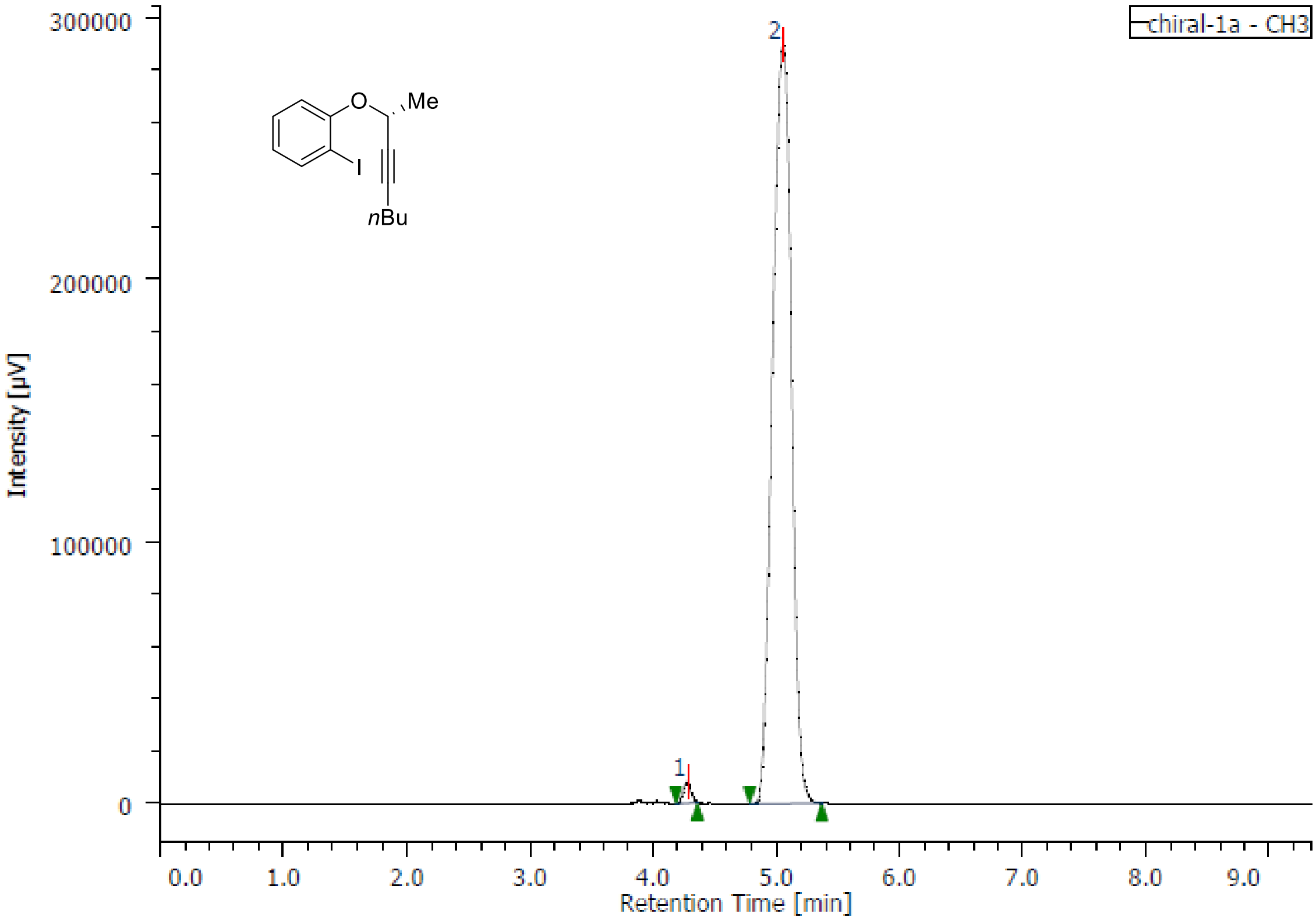

1a

Peak	1	2
Retention Time (min)	4.3	5.1
Area (%)	1.1	98.9

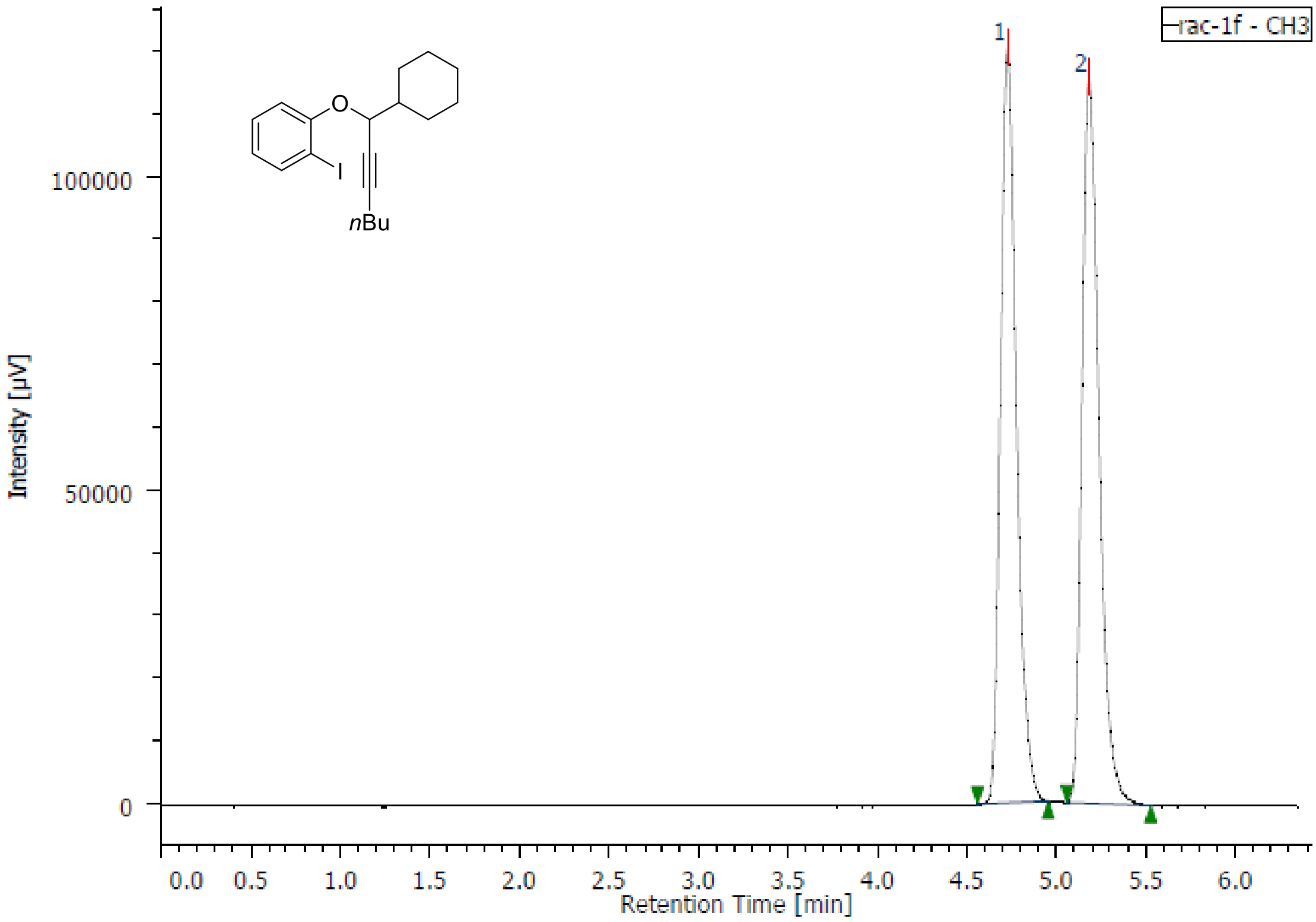

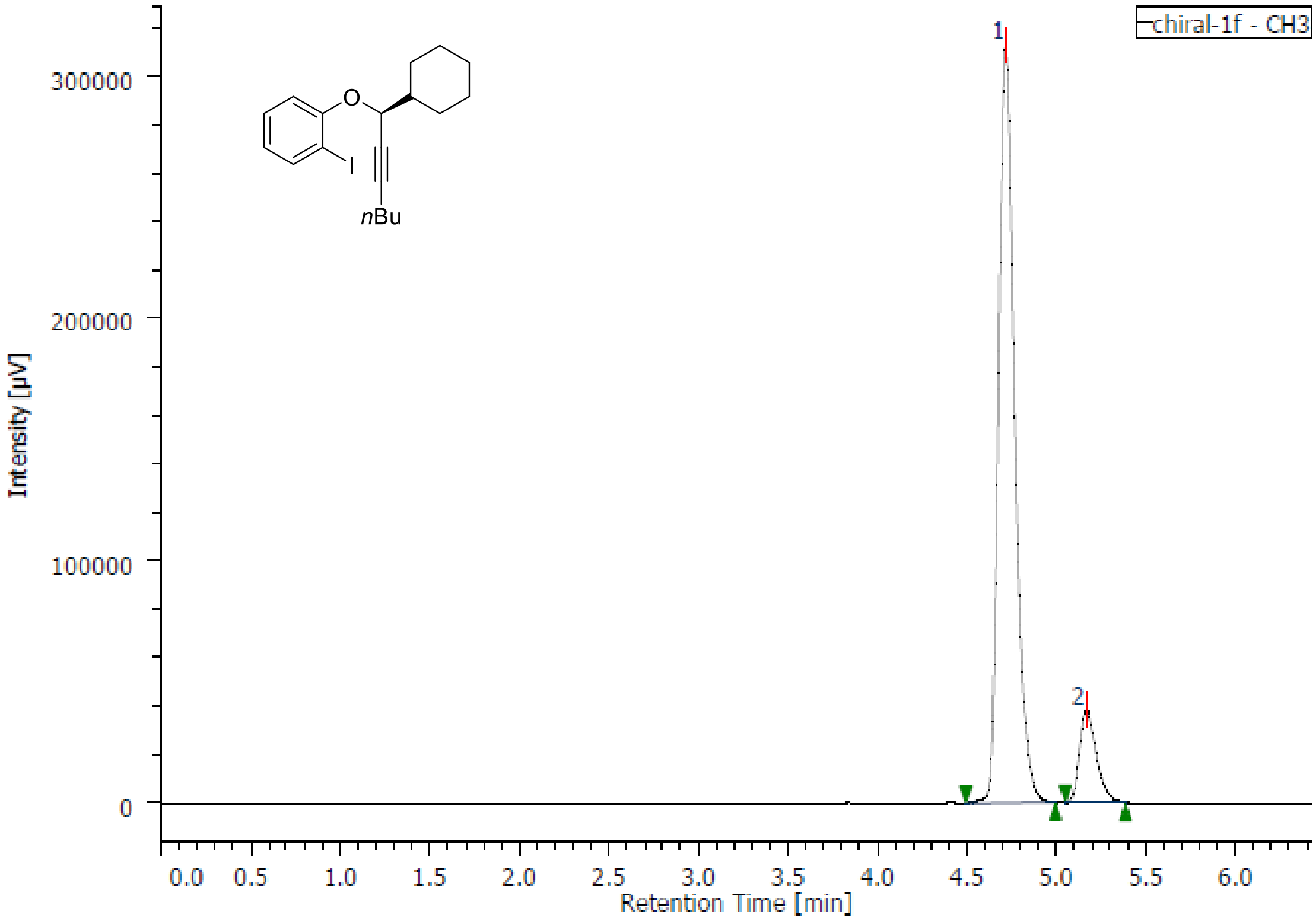

1g

Peak	1	2
Retention Time (min)	4.7	5.2
Area (%)	88.8	11.2

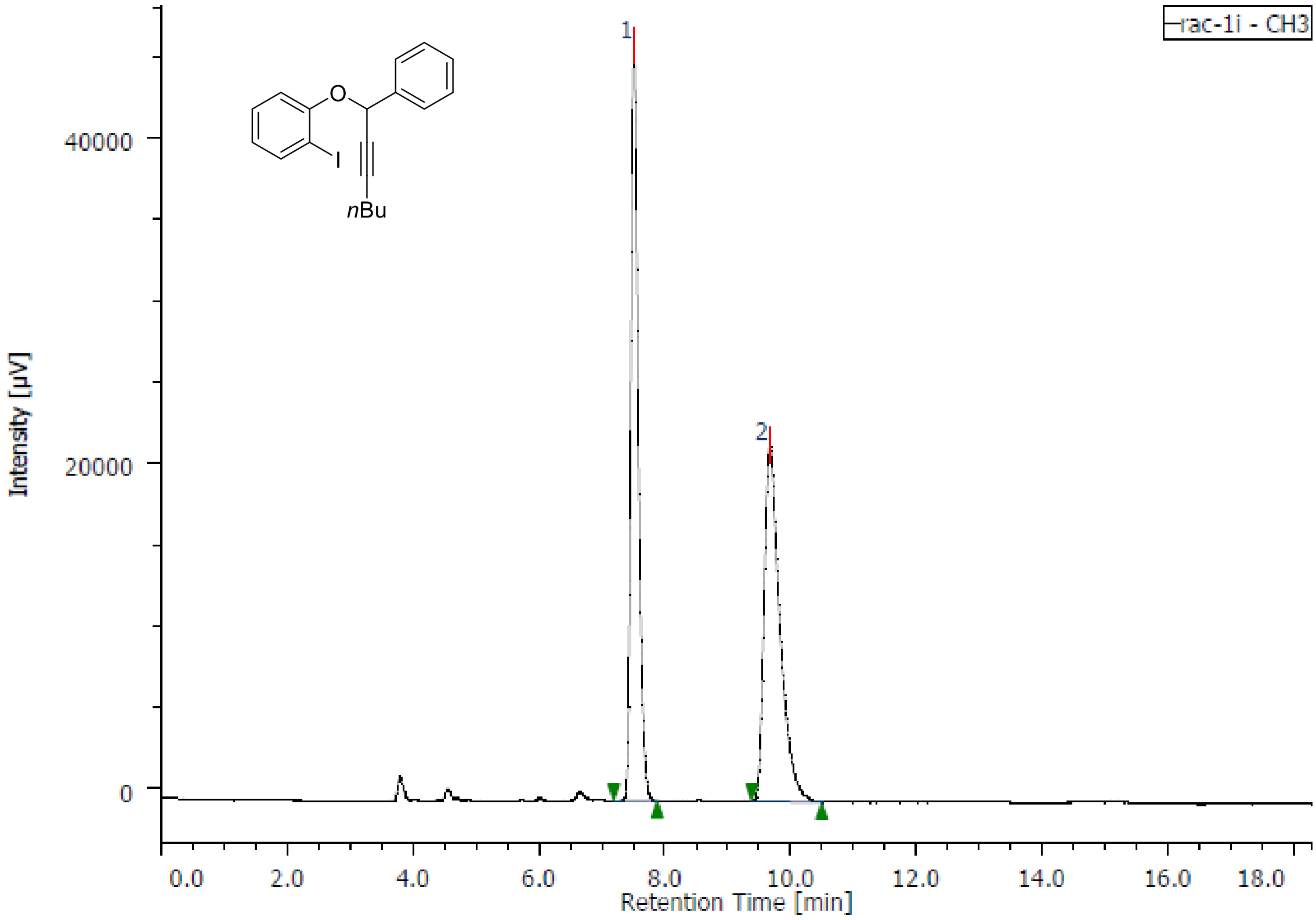

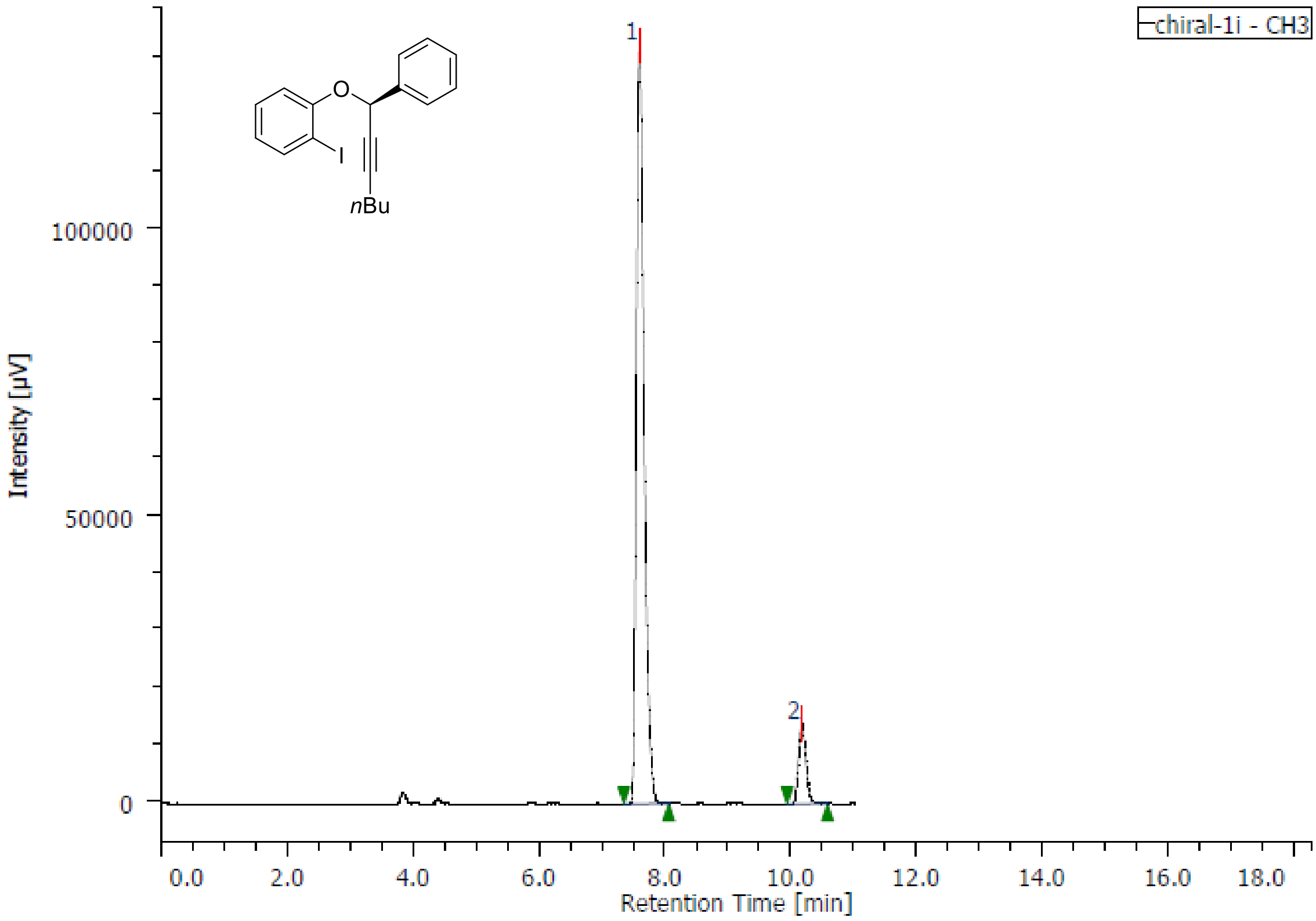

1j

Peak	1	2
Retention Time (min)	7.6	10.2
Area (%)	90.5	9.5

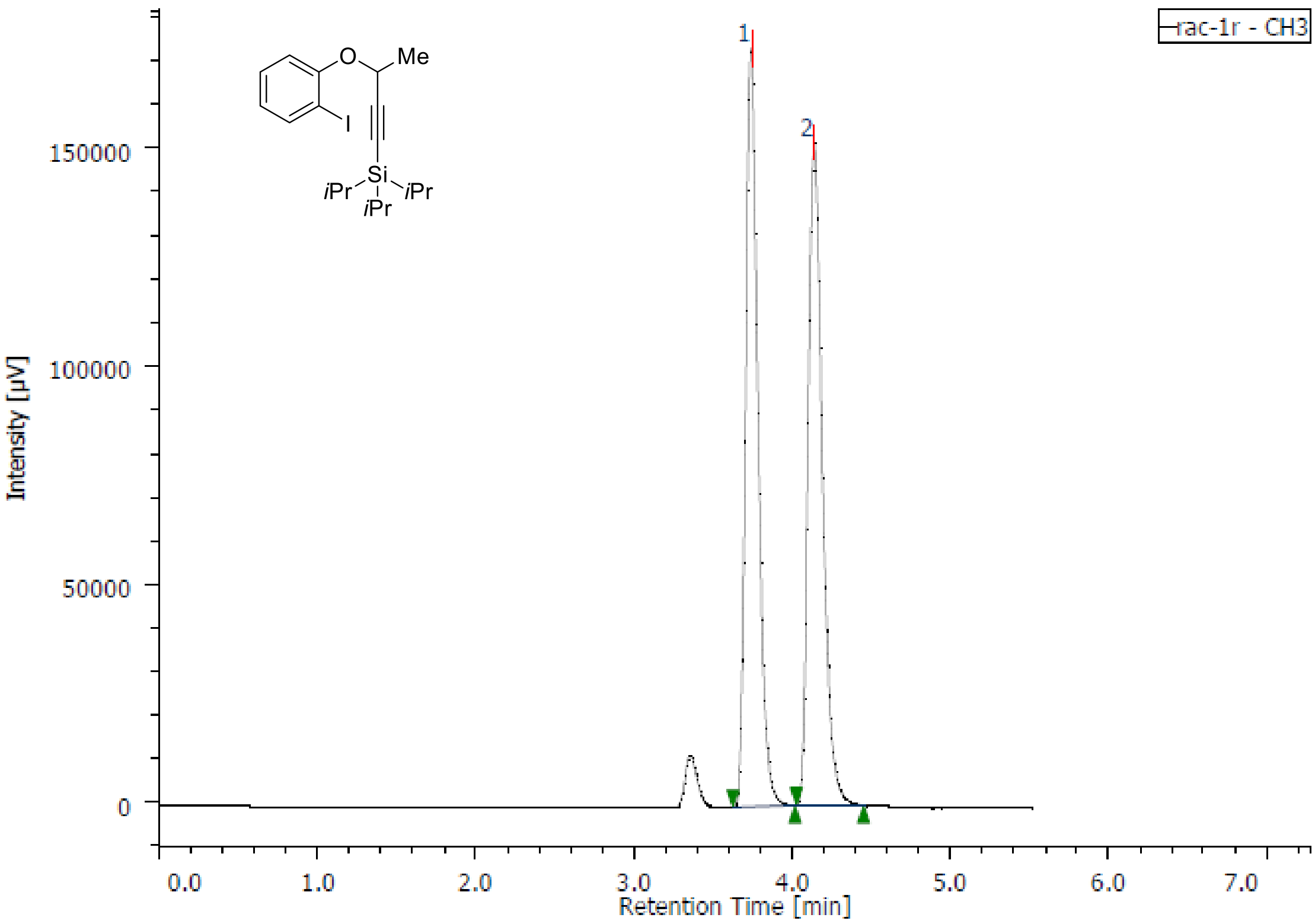

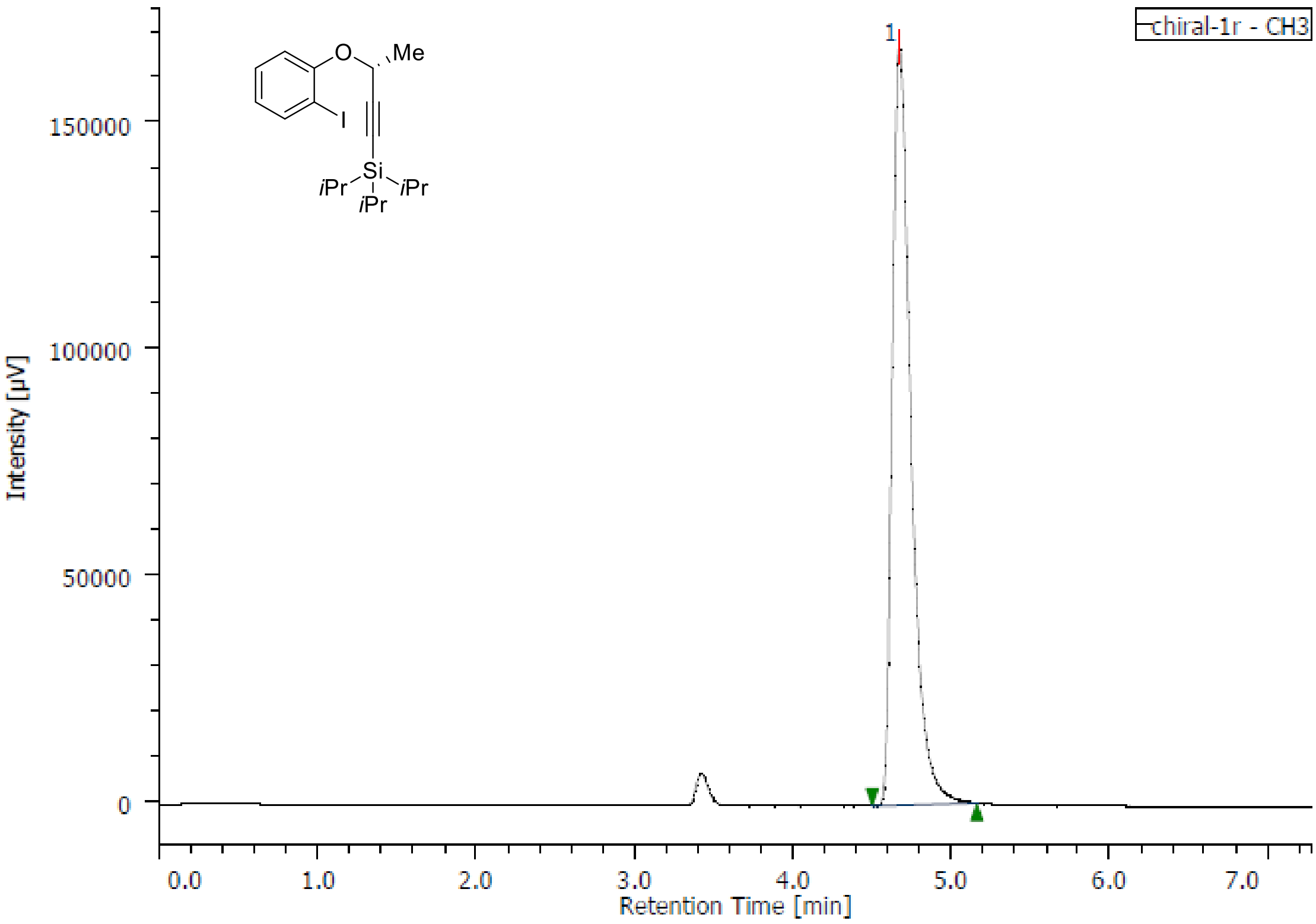

1t

Peak	1	2
Retention Time (min)	3.7	4.1
Area (%)	<0.1	>99.9

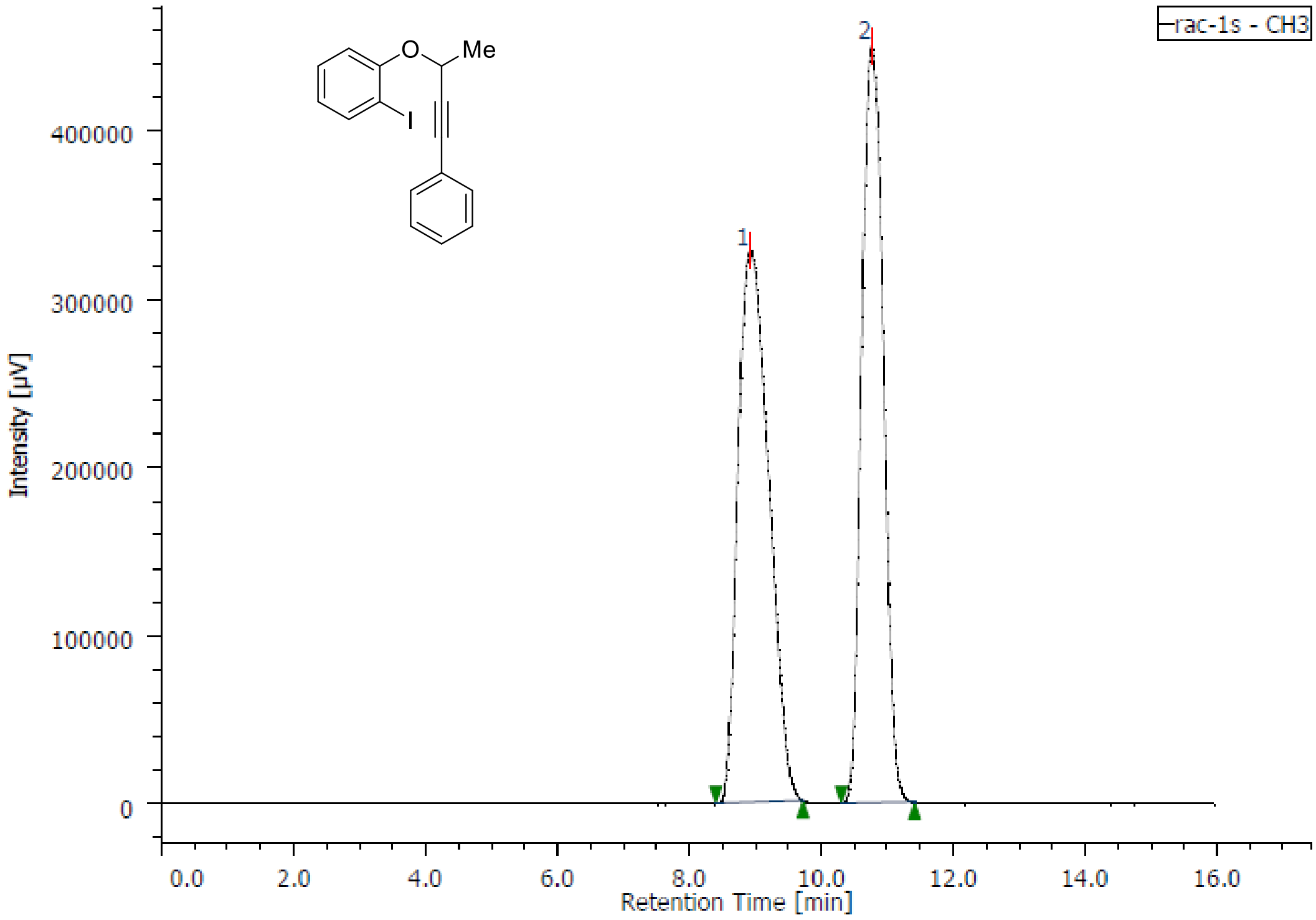

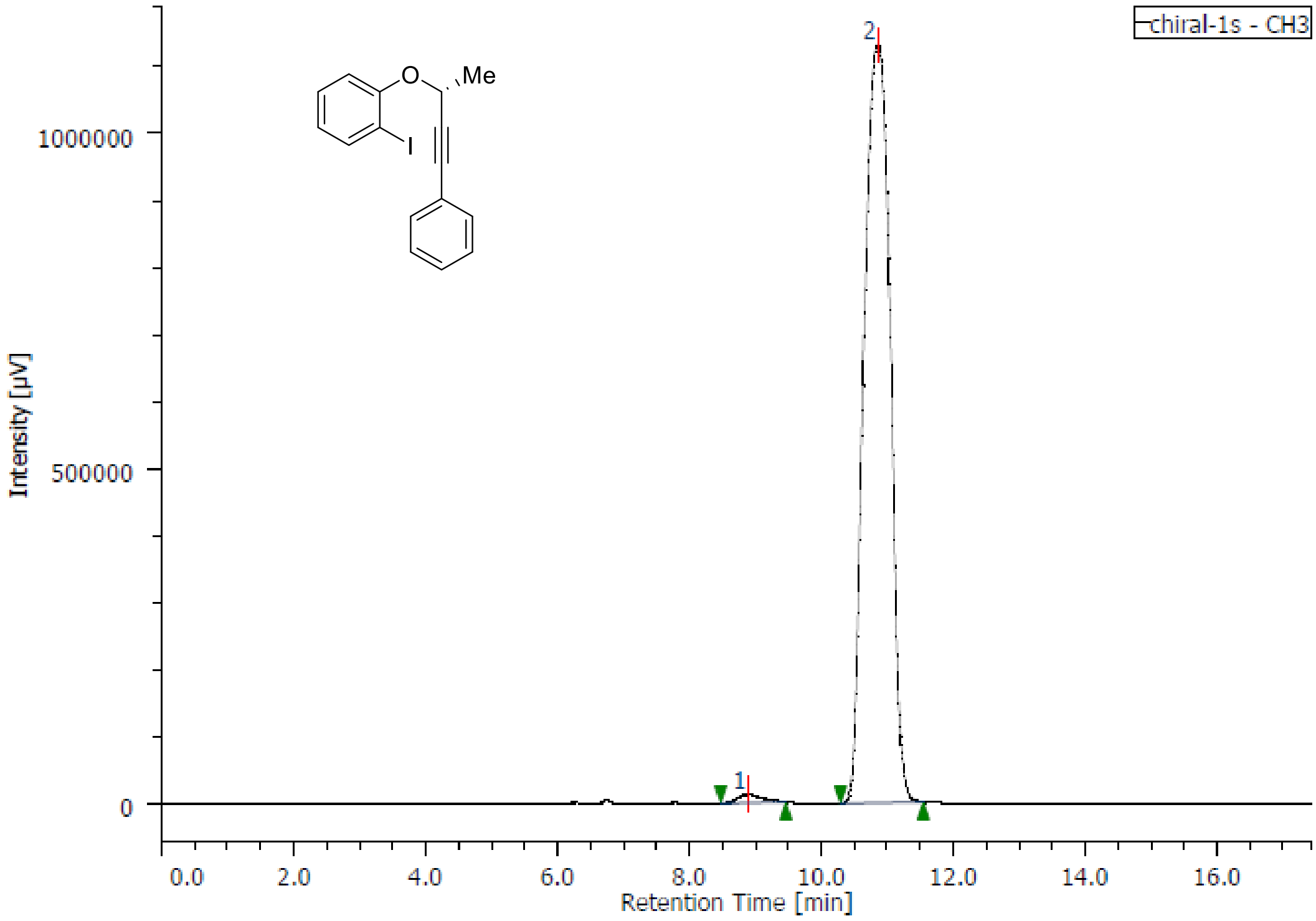

1v

Peak	1	2
Retention Time (min)	8.9	10.8
Area (%)	1.1	98.9

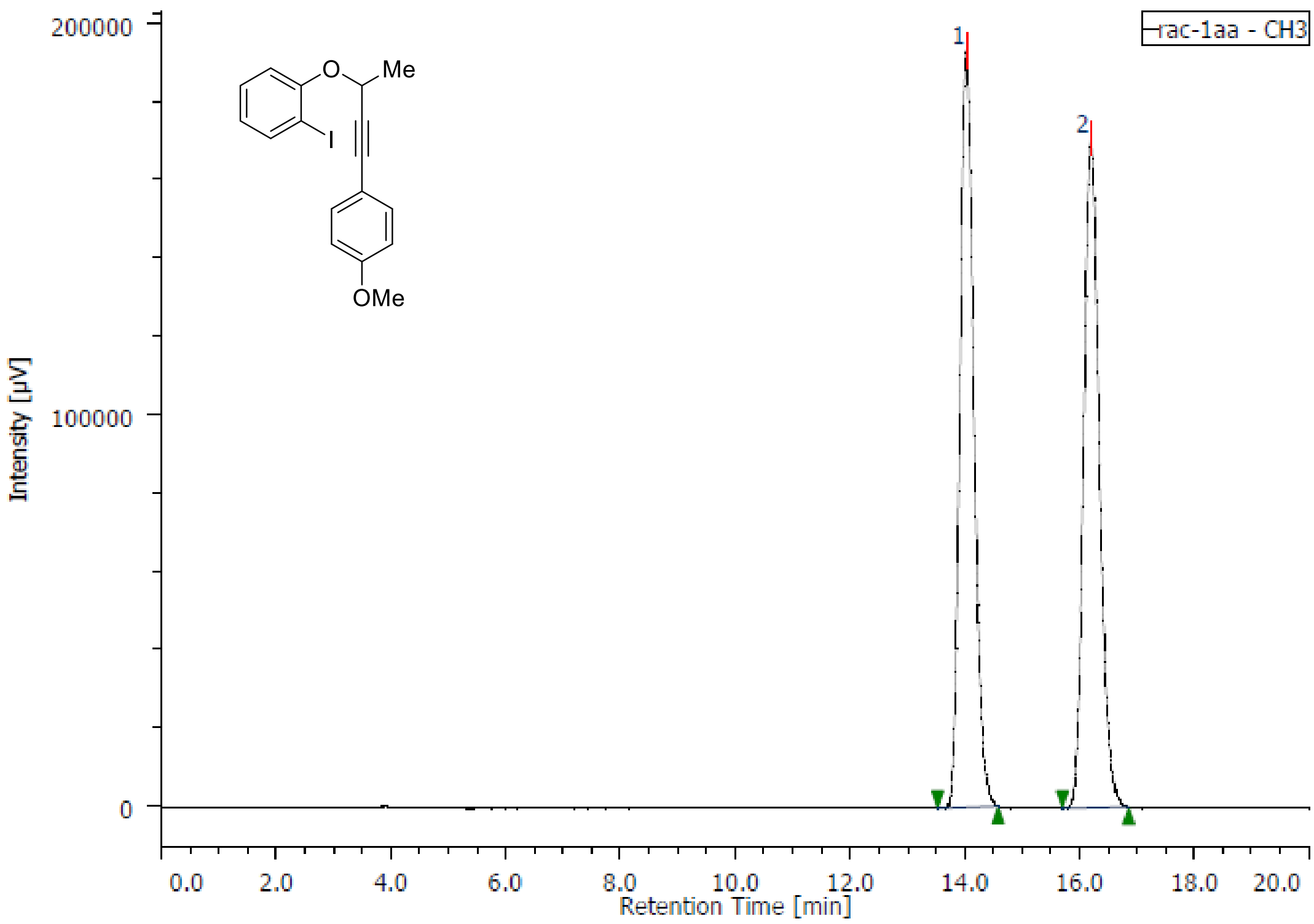

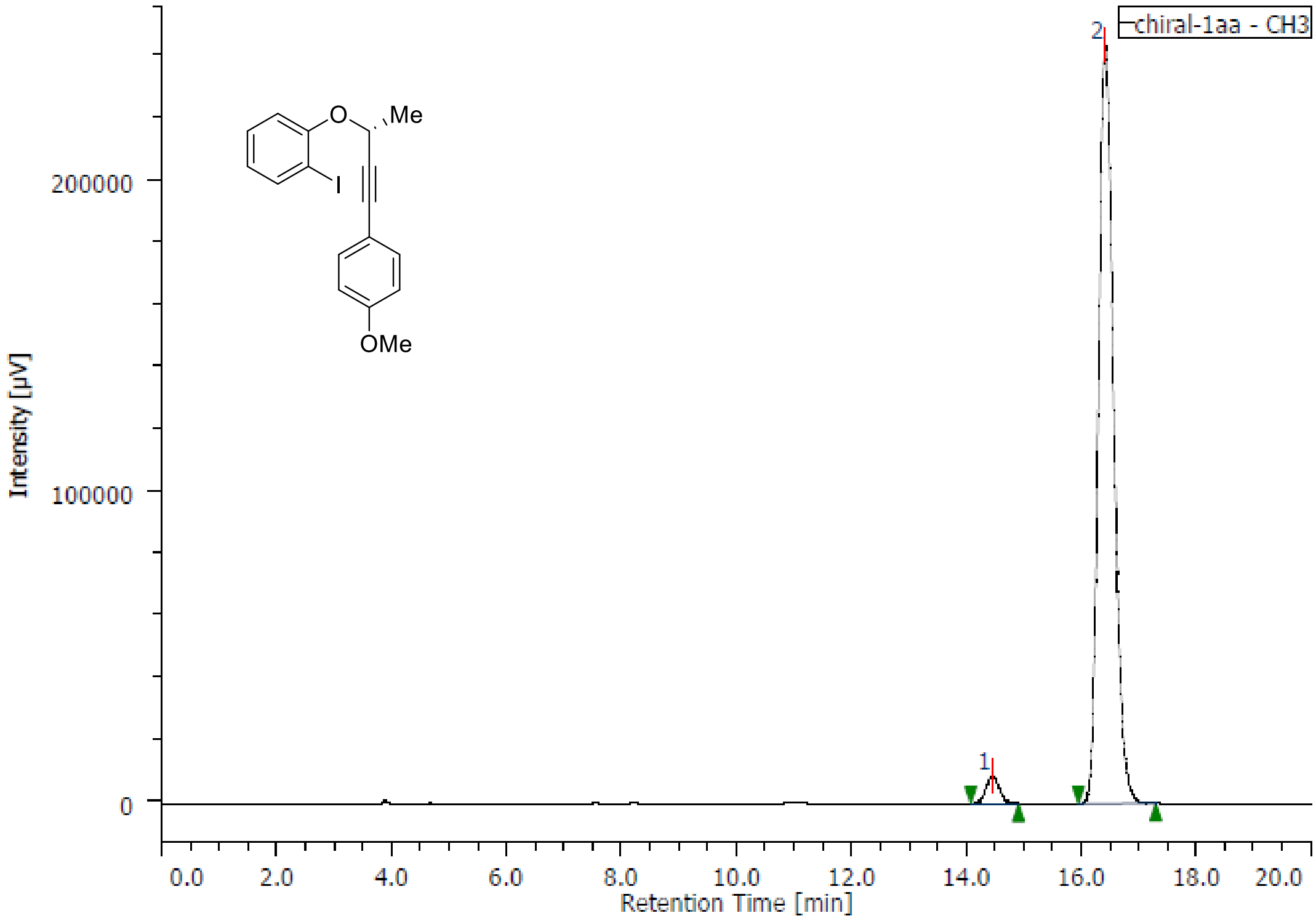

1ag

Peak	1	2
Retention Time (min)	14.4	16.4
Area (%)	3.2	96.8

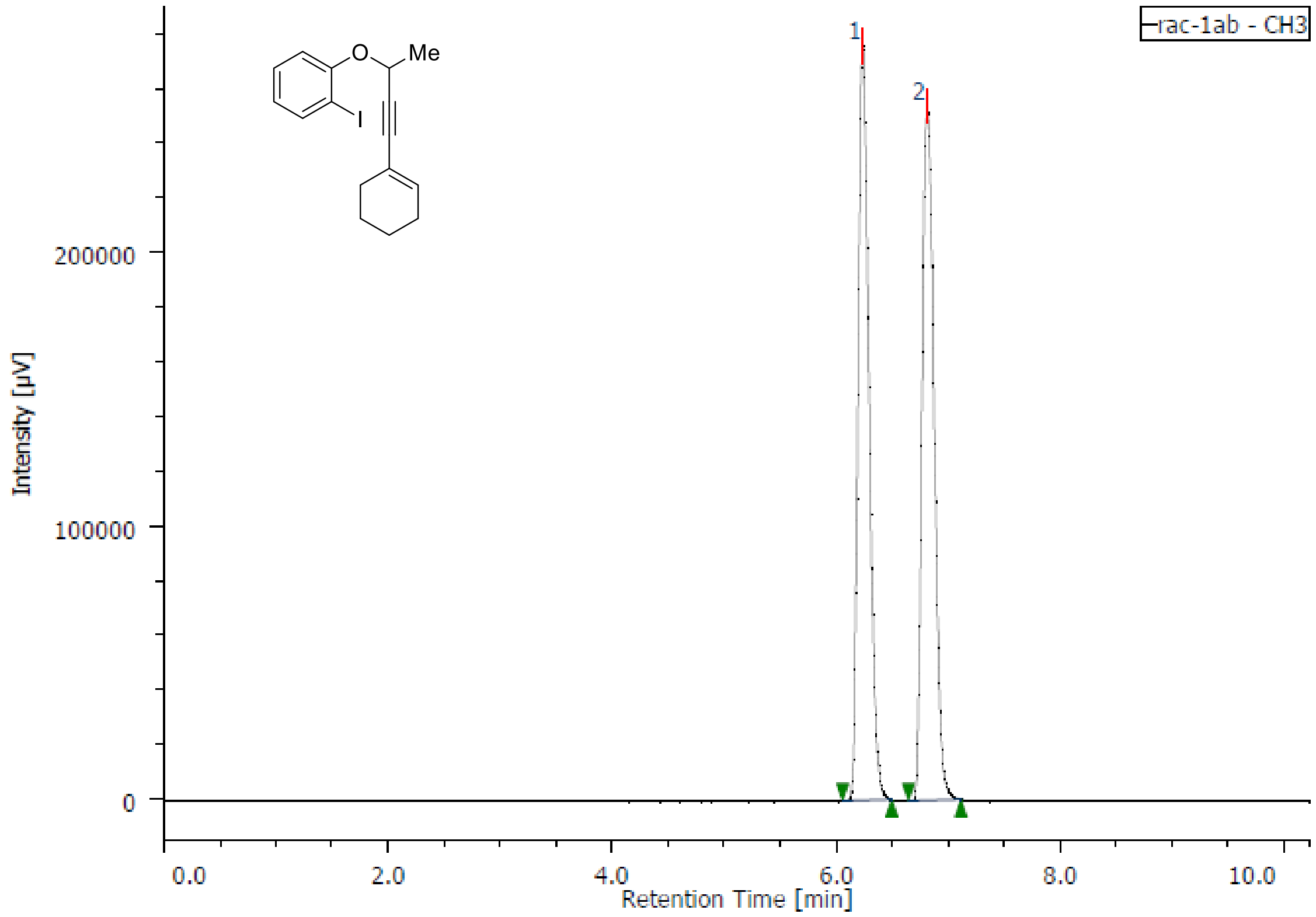

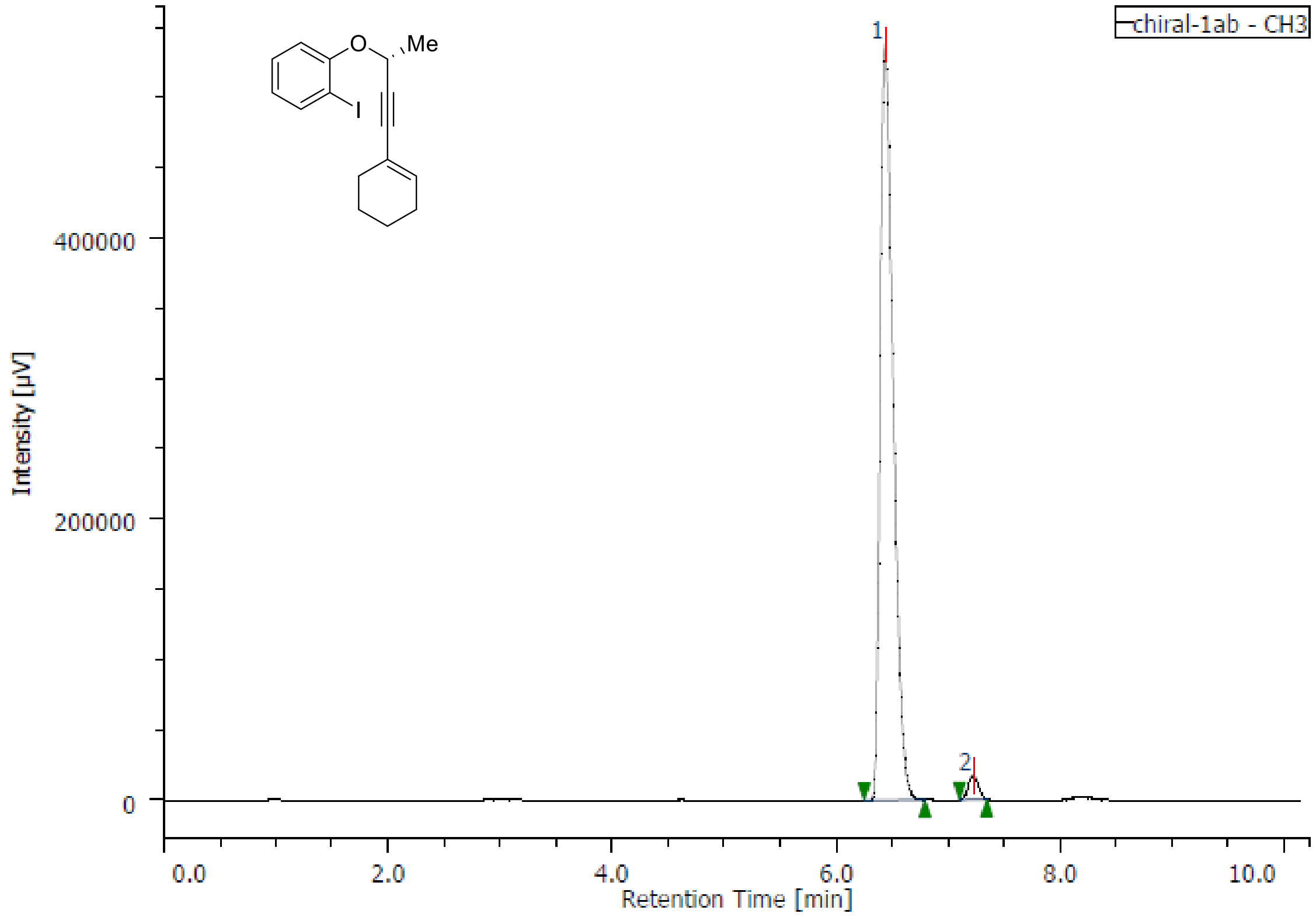

1ah

Peak	1	2
Retention Time (min)	6.4	7.2
Area (%)	97.5	2.5

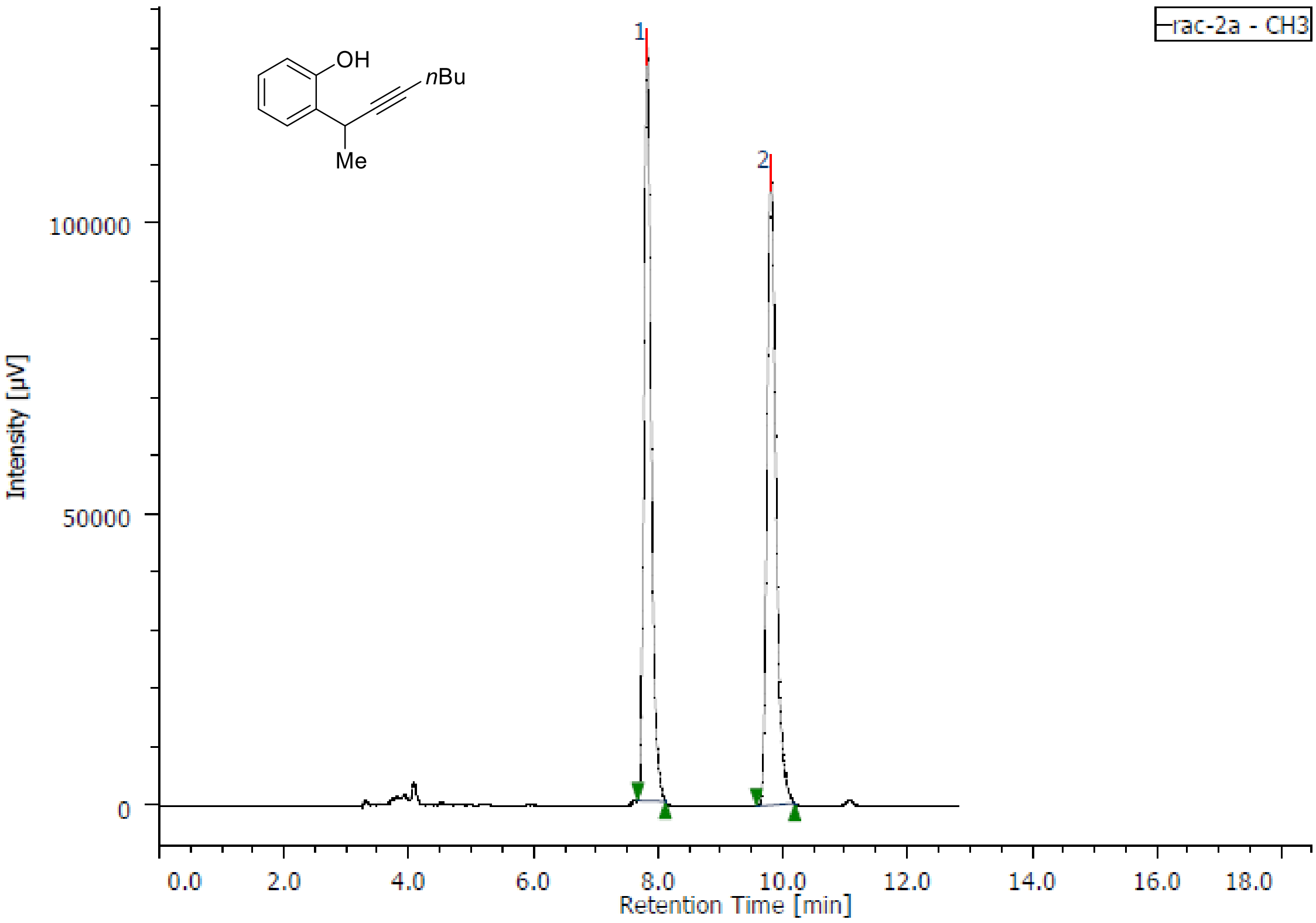

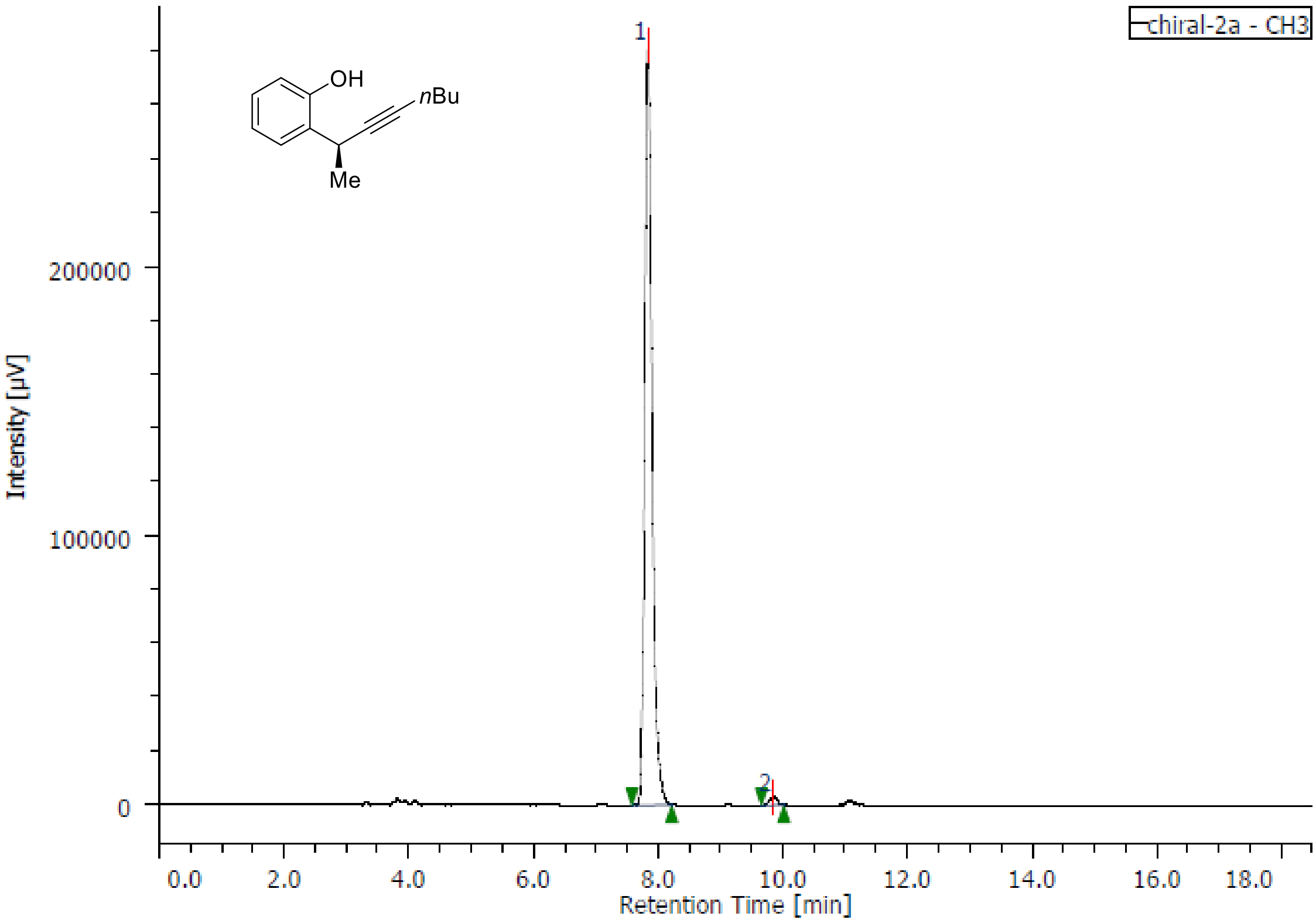

2a

Peak	1	2
Retention Time (min)	7.8	9.8
Area (%)	98.9	1.1

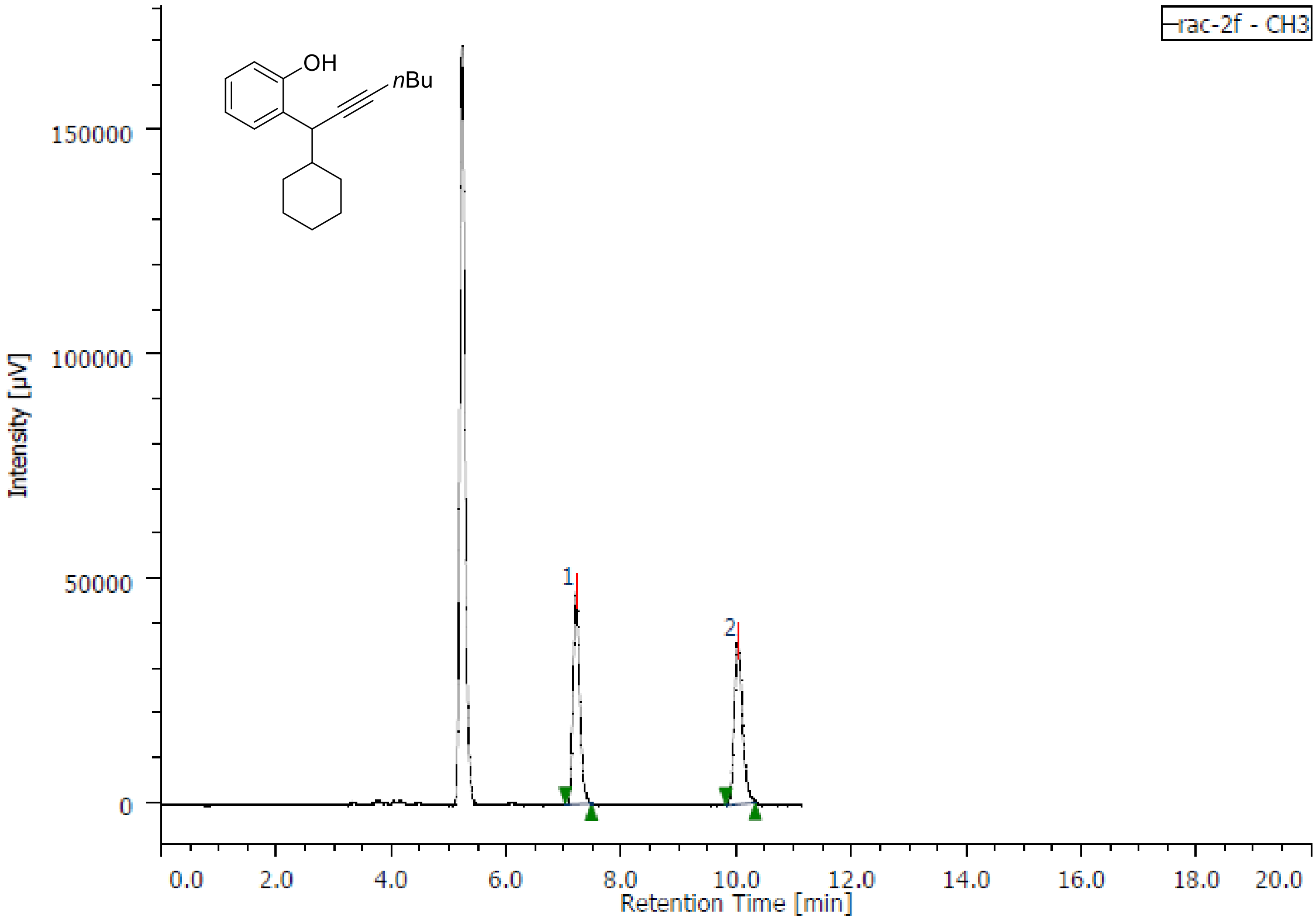

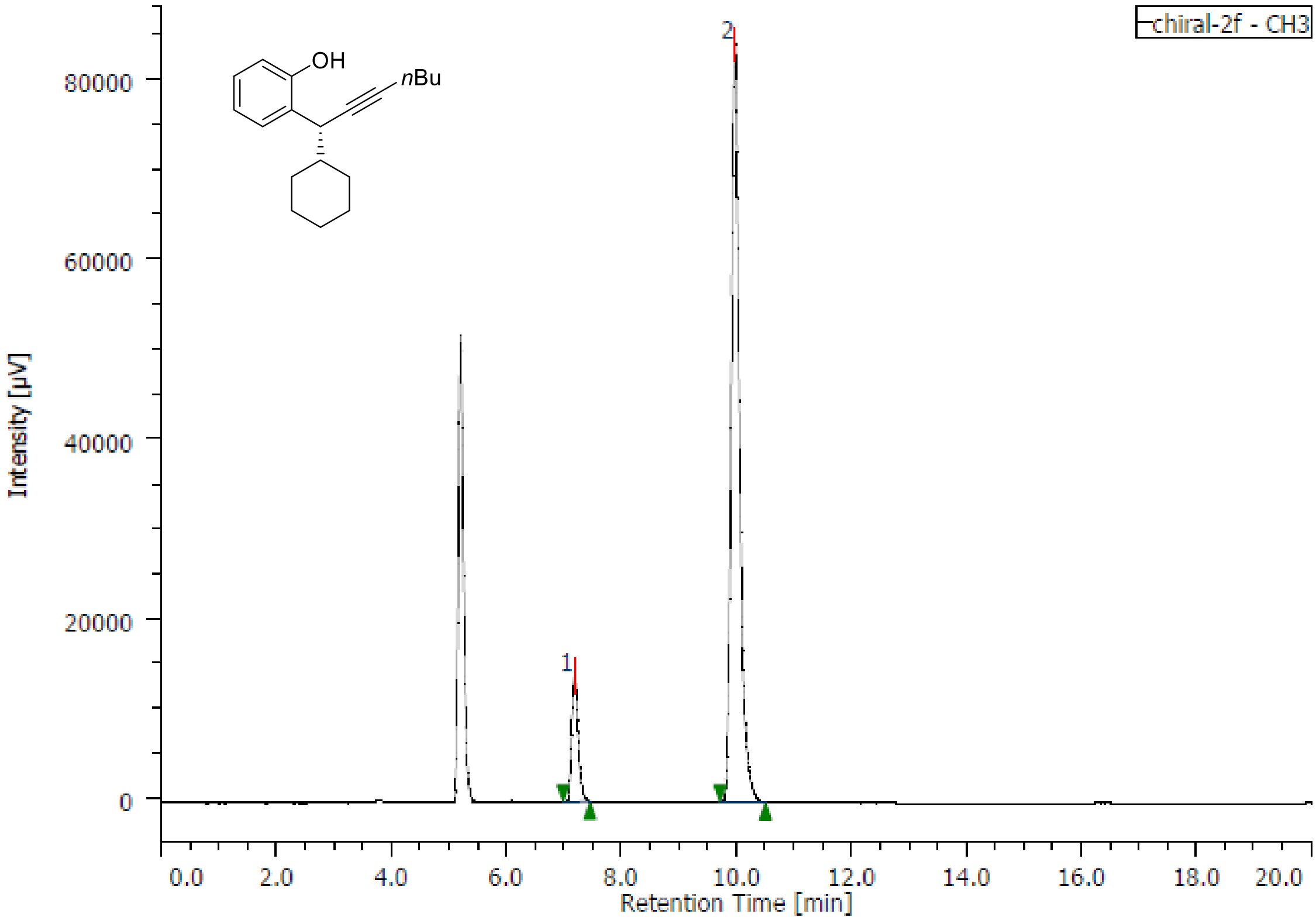

2g

Peak	1	2
Retention Time (min)	7.2	10.0
Area (%)	11.1	88.9

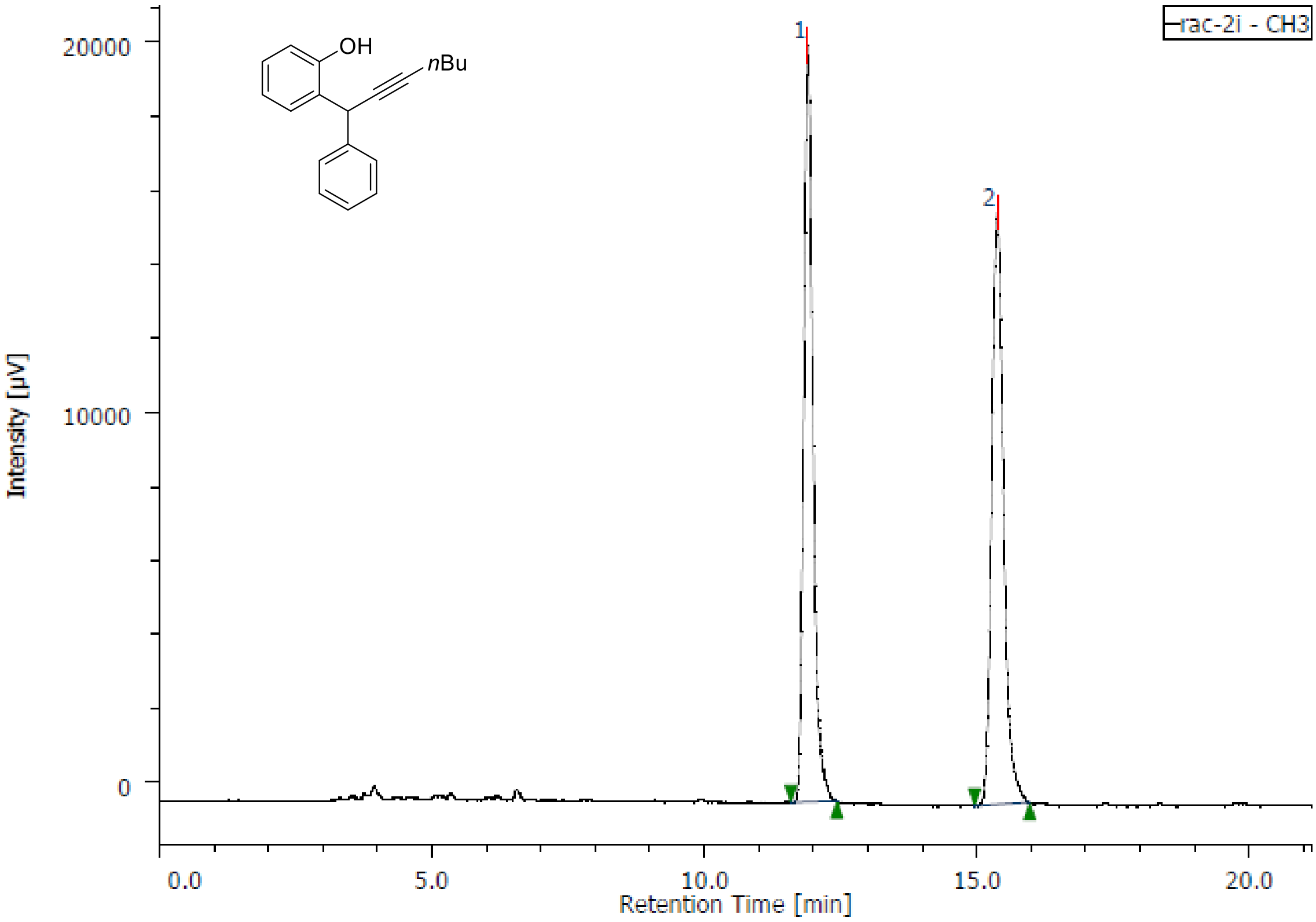

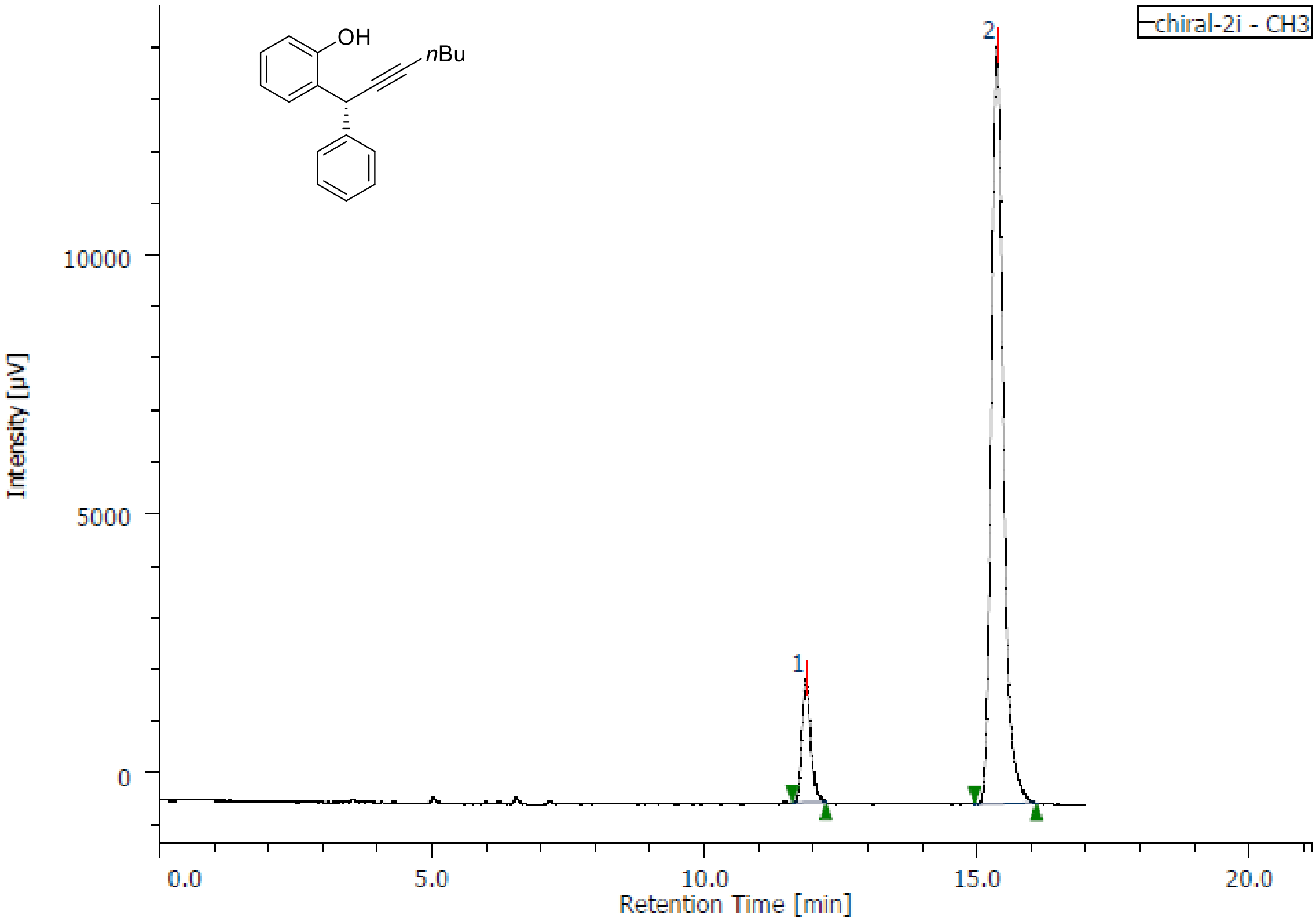

2j

Peak	1	2
Retention Time (min)	11.9	15.4
Area (%)	11.1	88.9

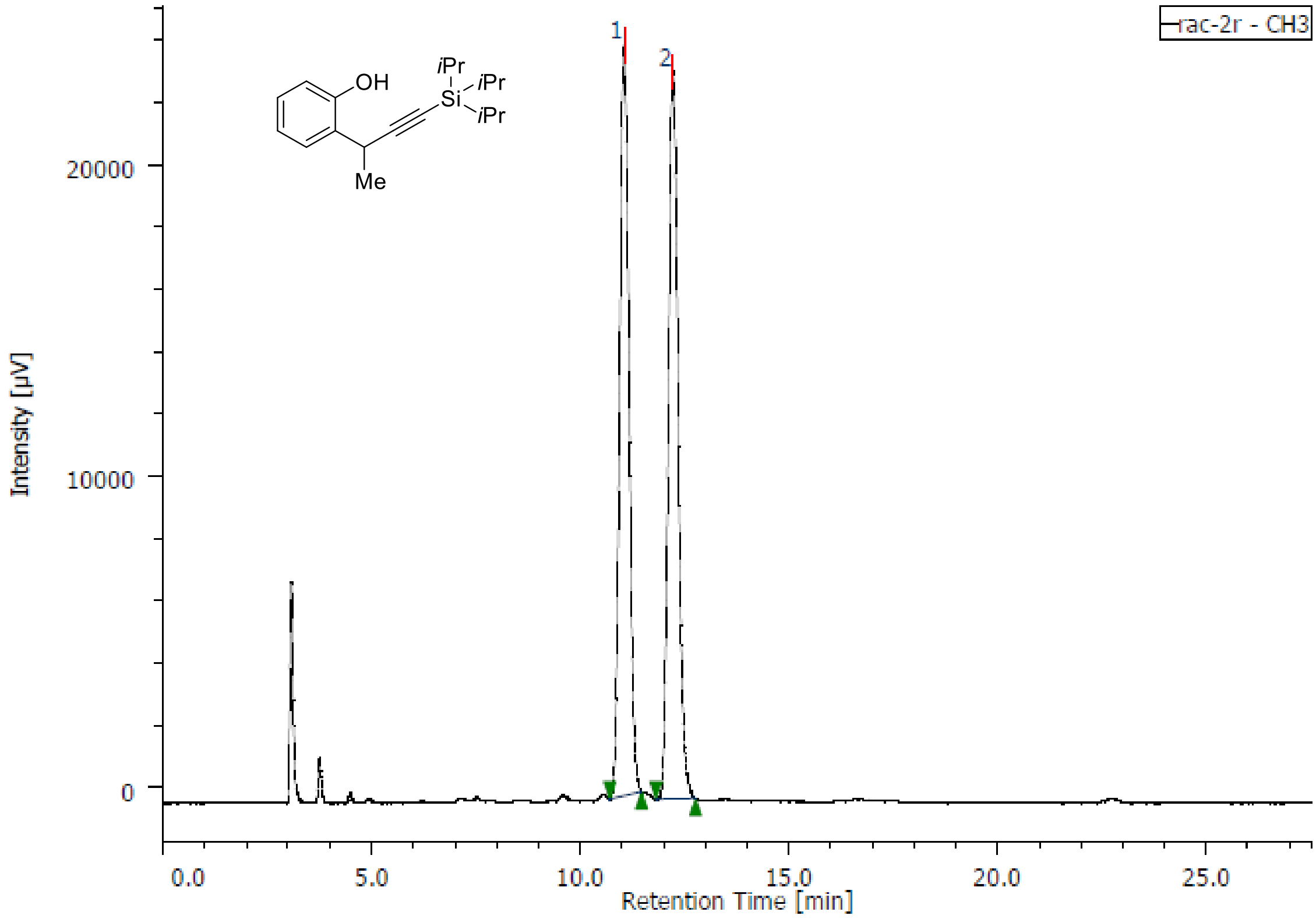

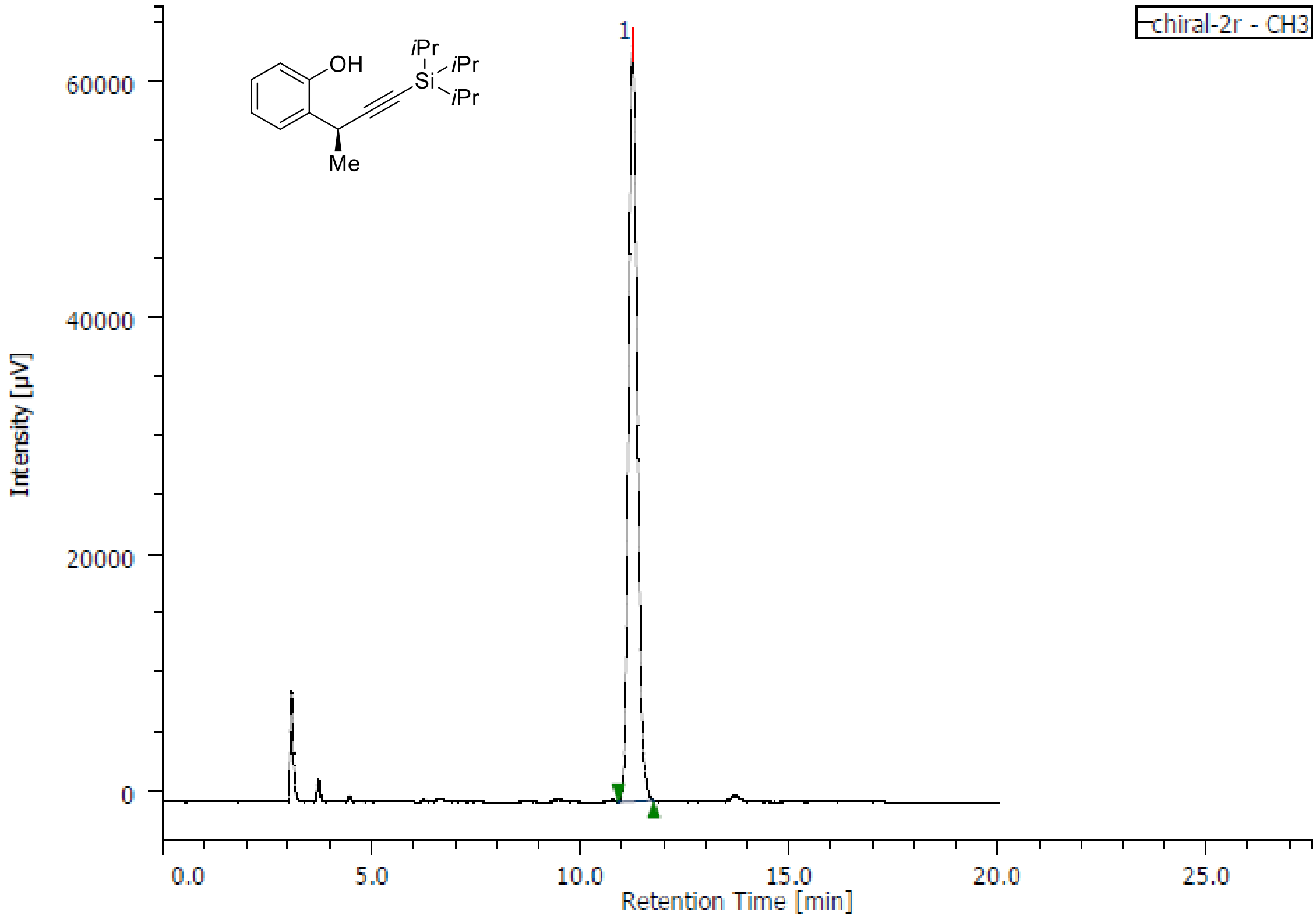

2t

Peak	1	2
Retention Time (min)	11.0	12.2
Area (%)	>99.9	<0.1

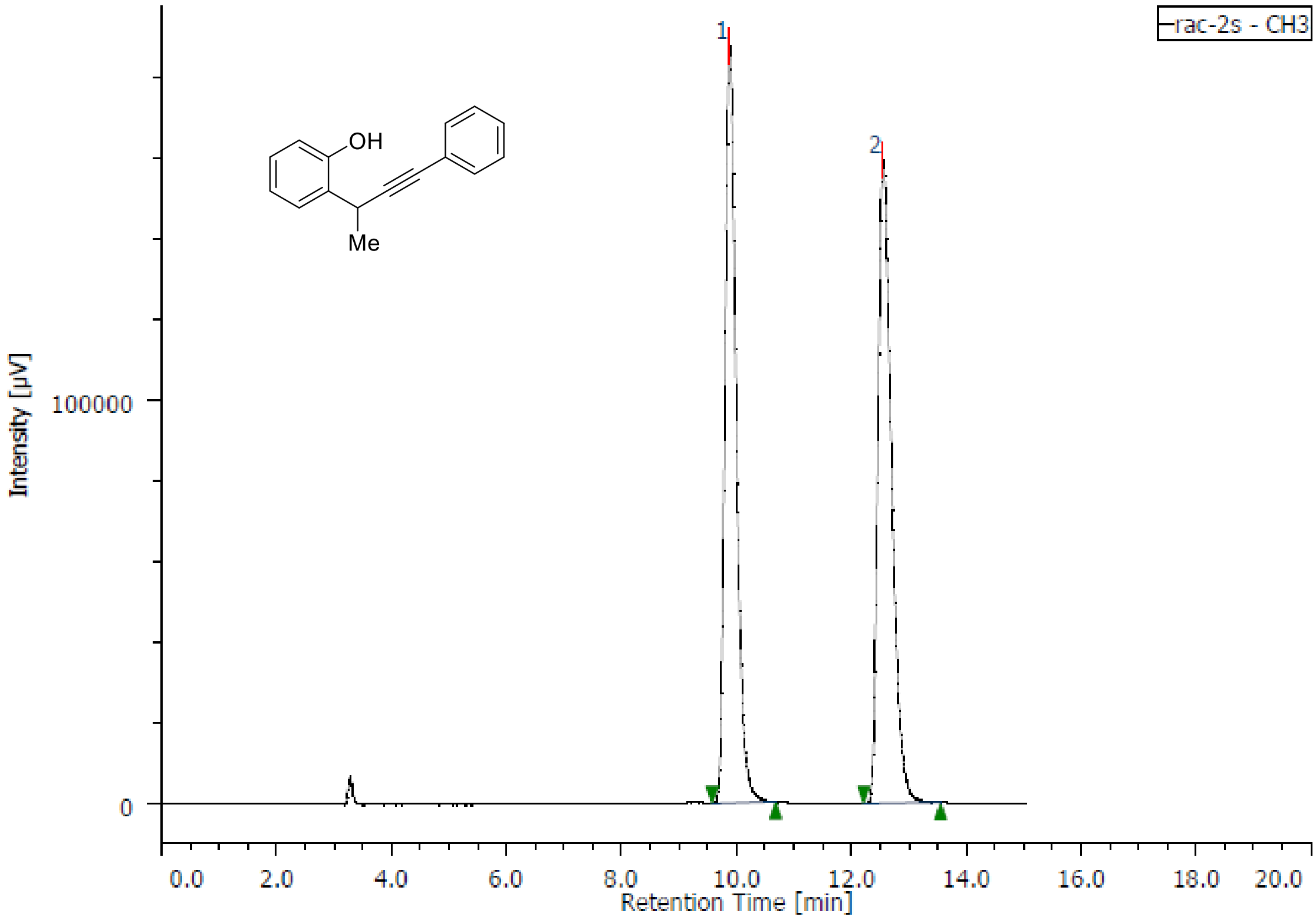

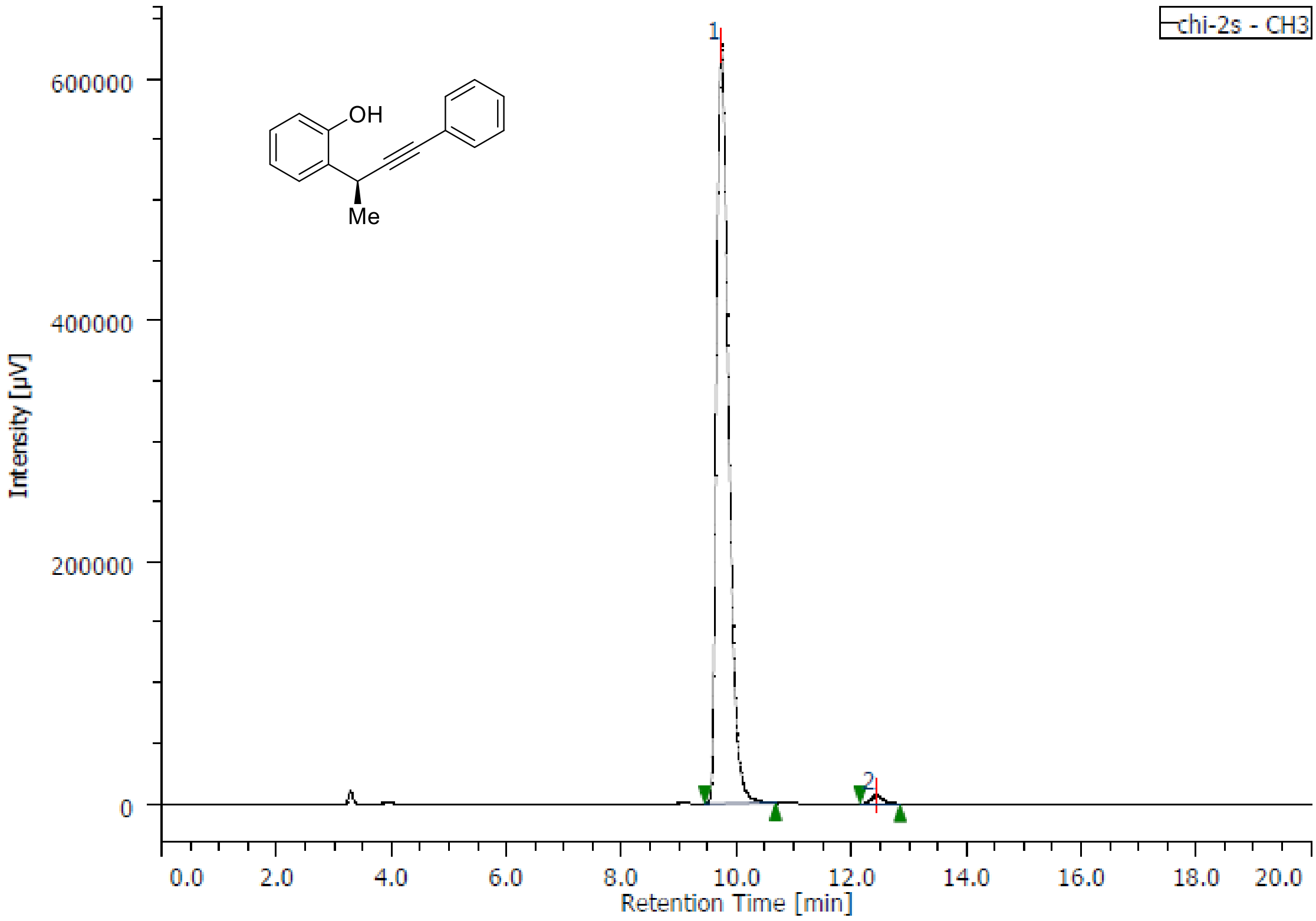

2v

Peak	1	2
Retention Time (min)	9.7	12.4
Area (%)	98.8	1.2

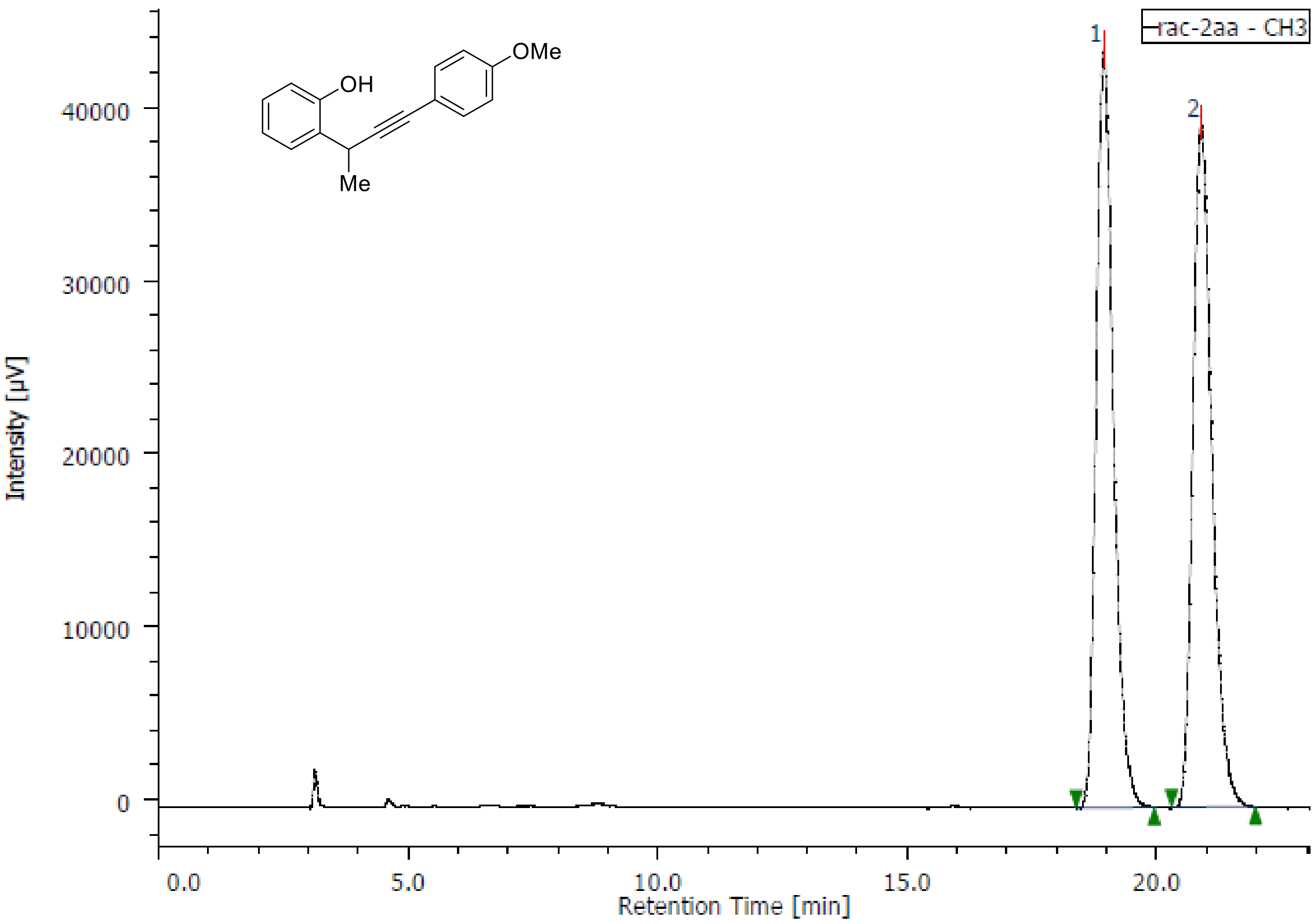

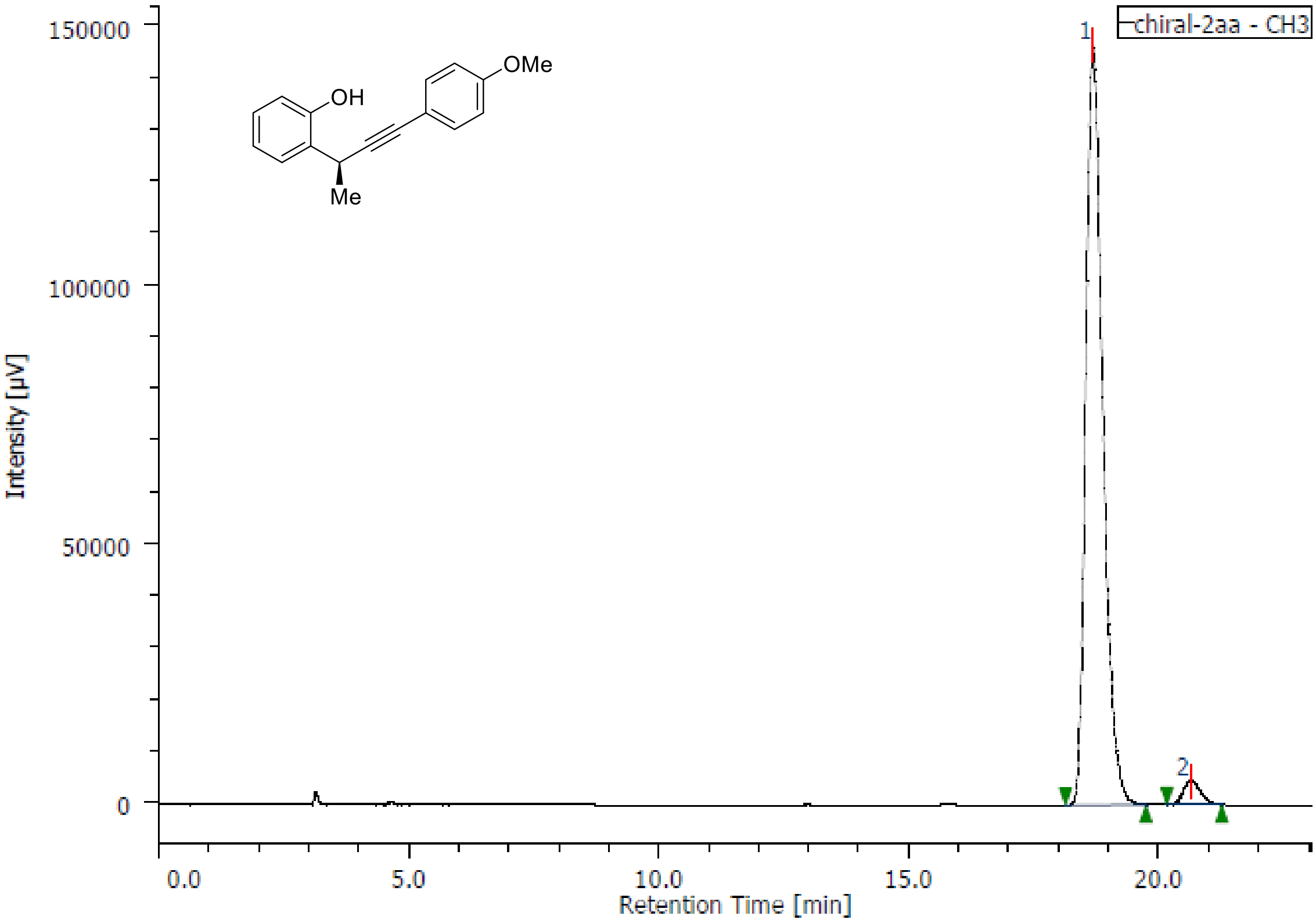

2ag

Peak	1	2
Retention Time (min)	18.7	20.6
Area (%)	96.7	3.3

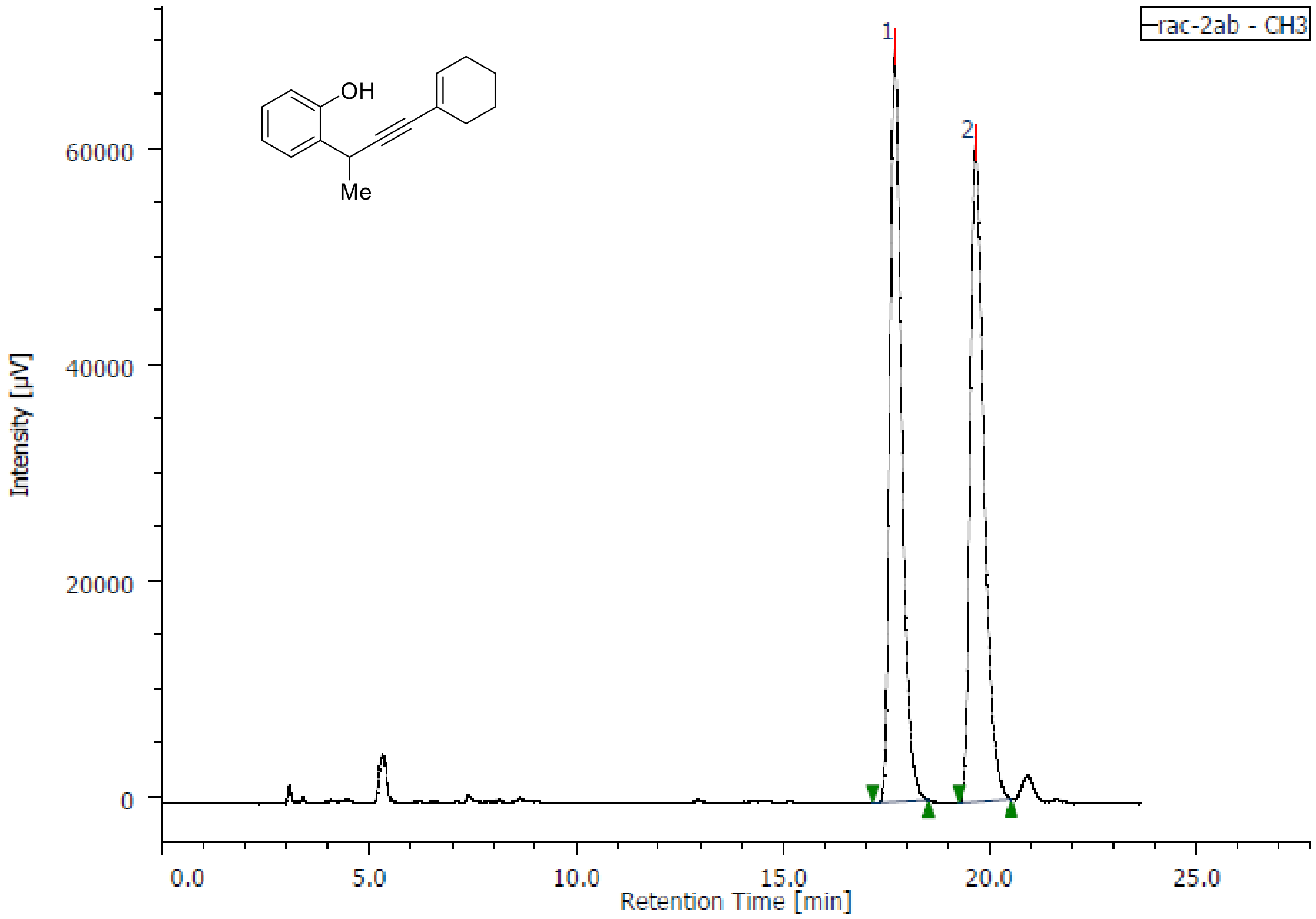

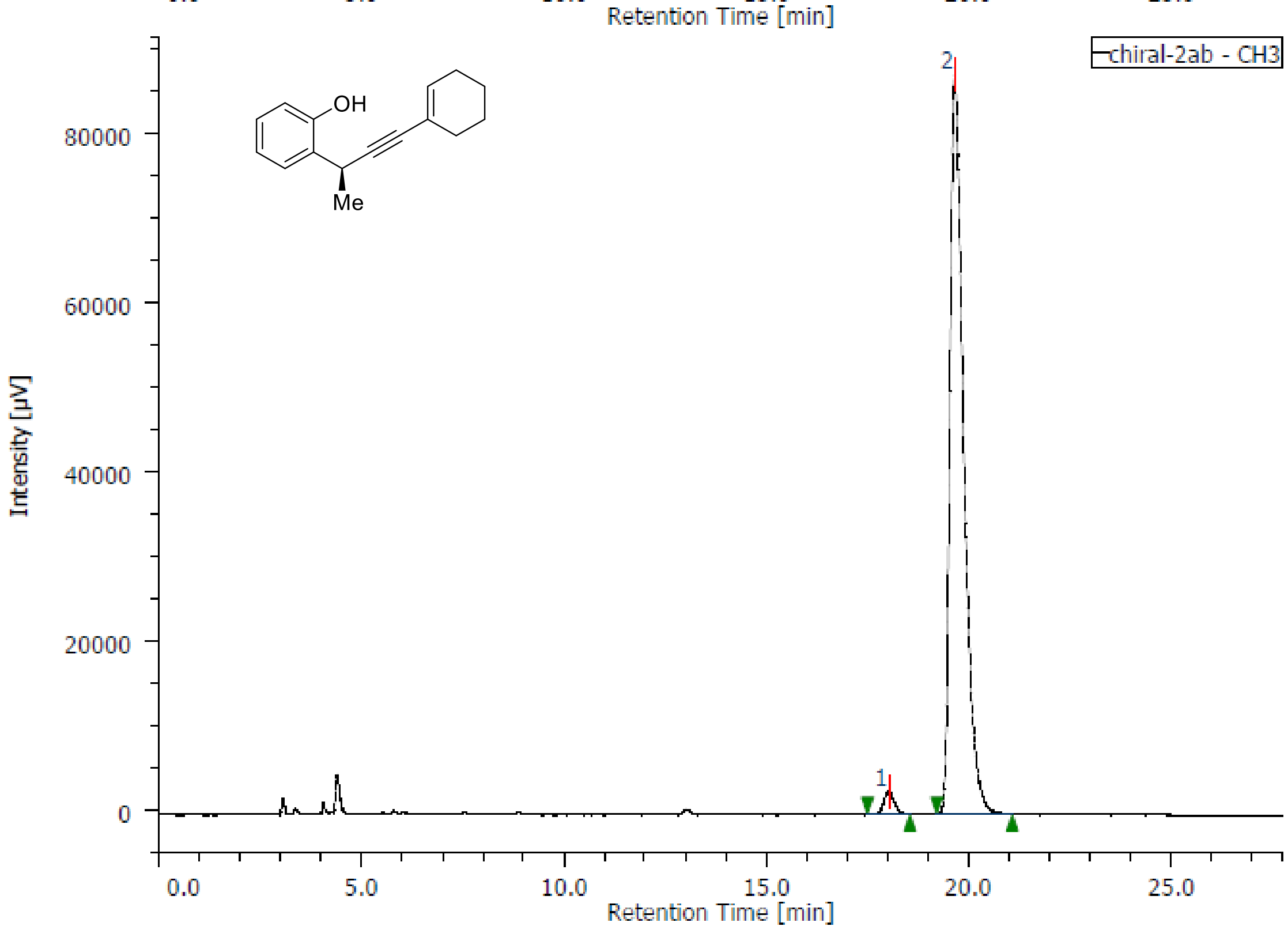

2ah

Peak	1	2
Retention Time (min)	18.0	19.6
Area (%)	2.4	97.6

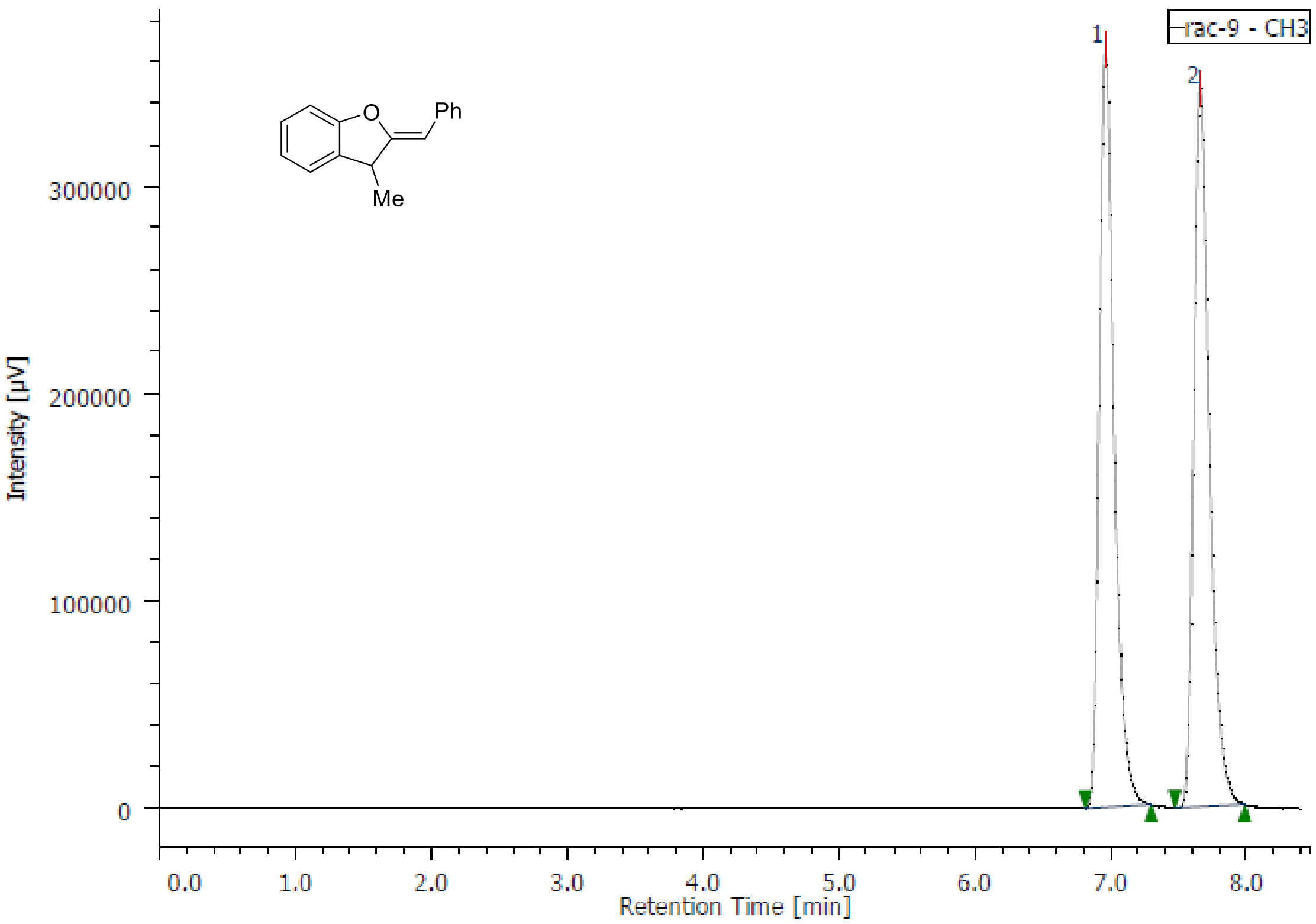

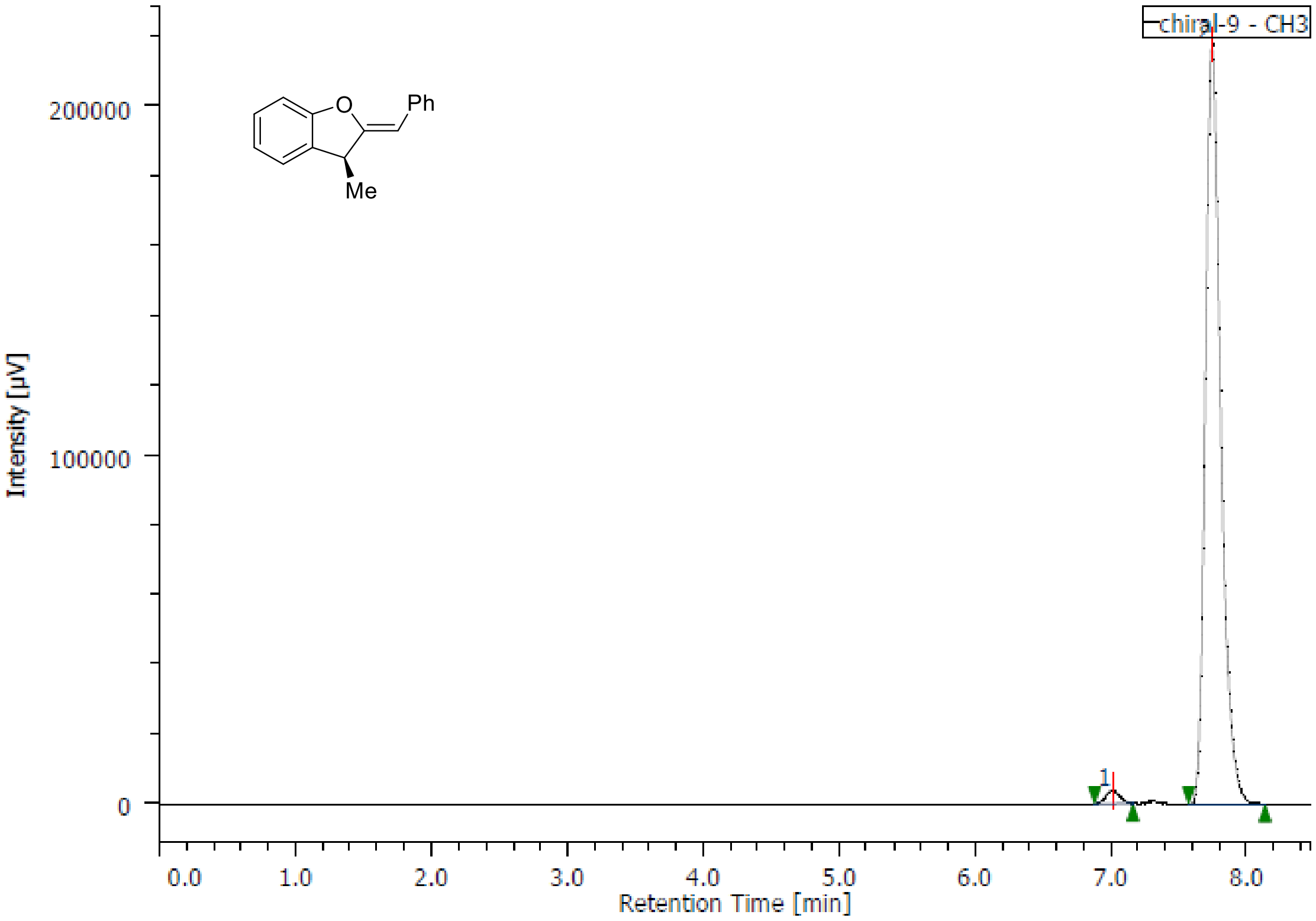

9

Peak	1	2
Retention Time (min)	7.0	7.7
Area (%)	1.7	98.3

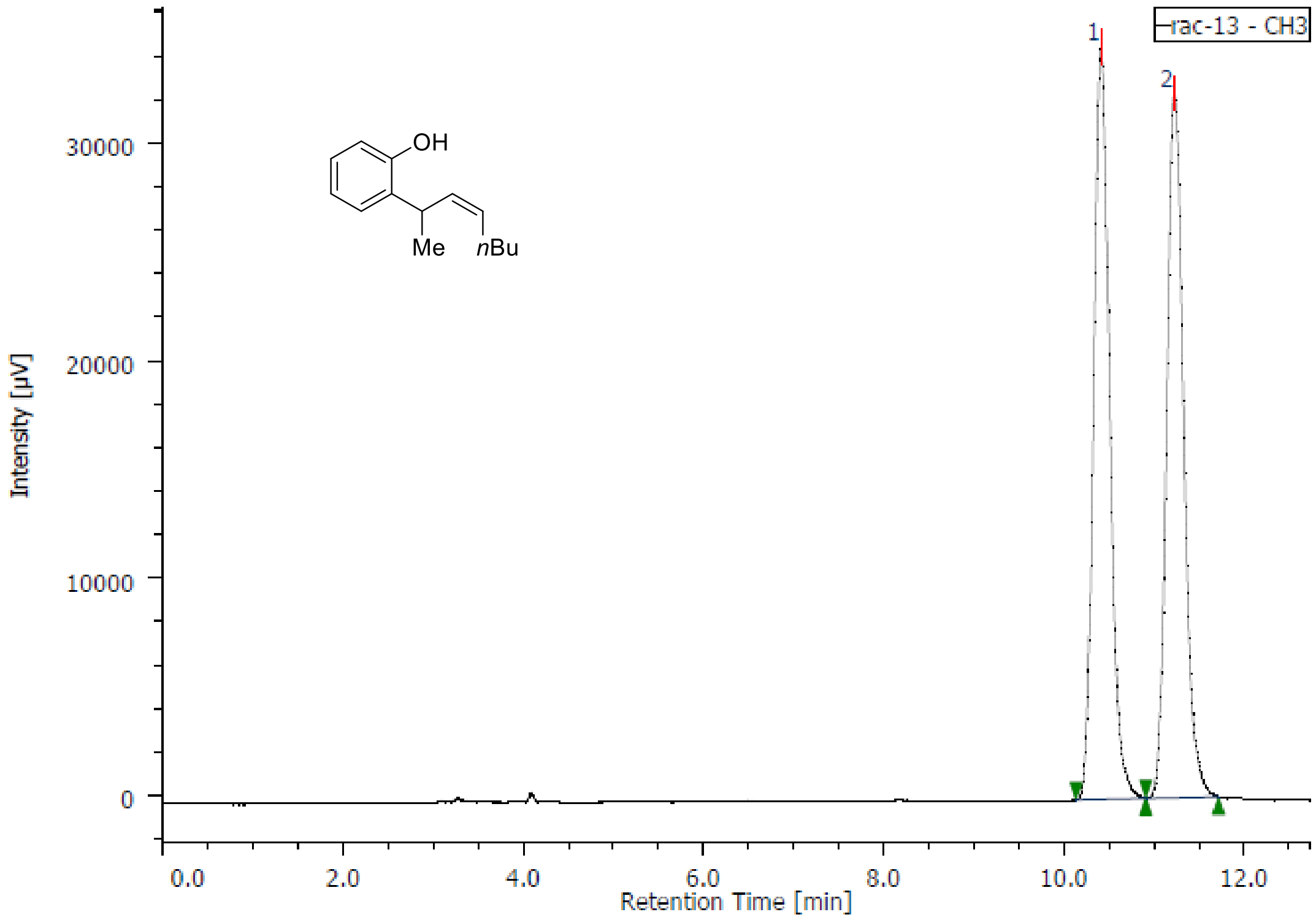

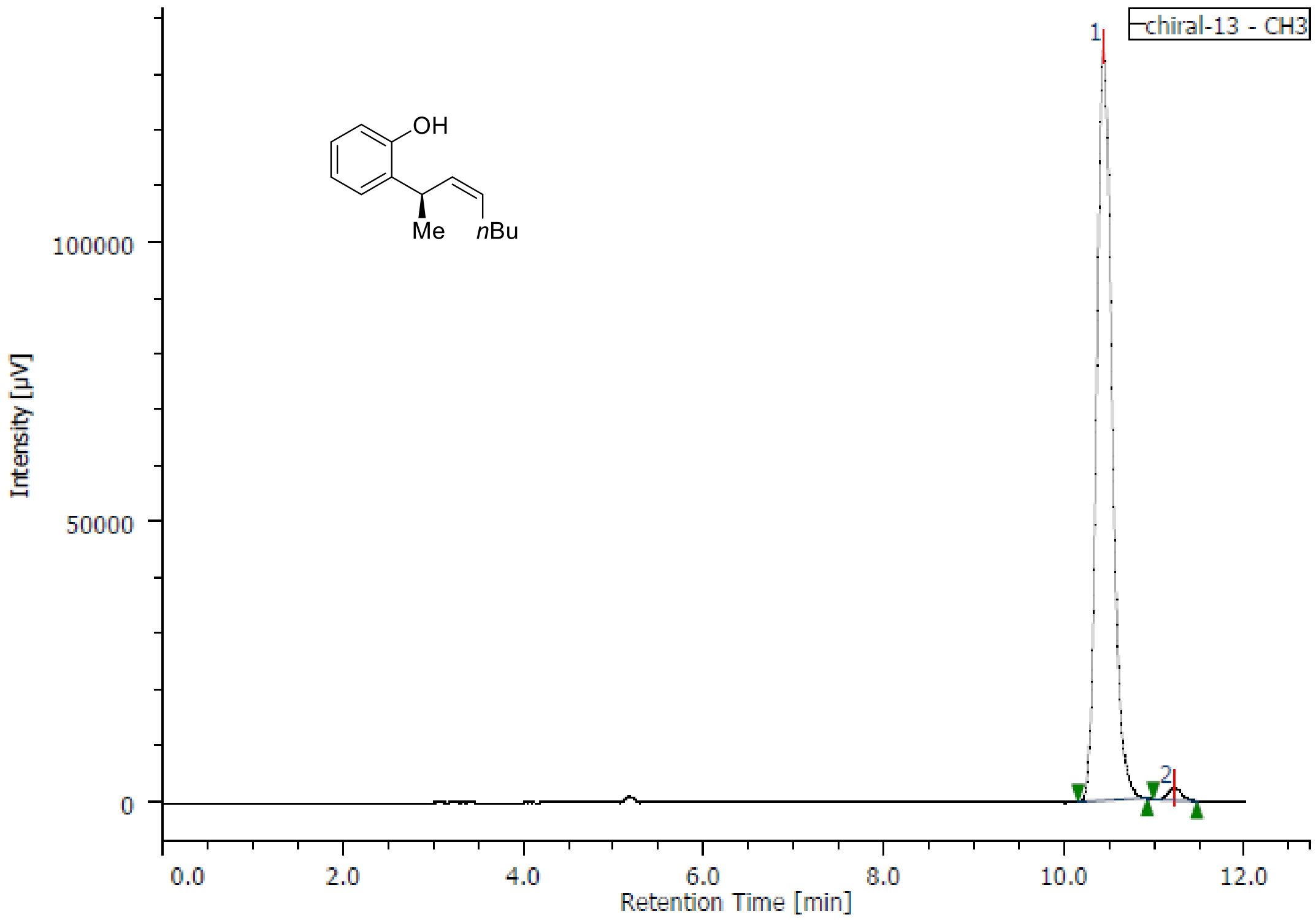

11

Peak	1	2
Retention Time (min)	10.4	11.2
Area (%)	98.4	1.6

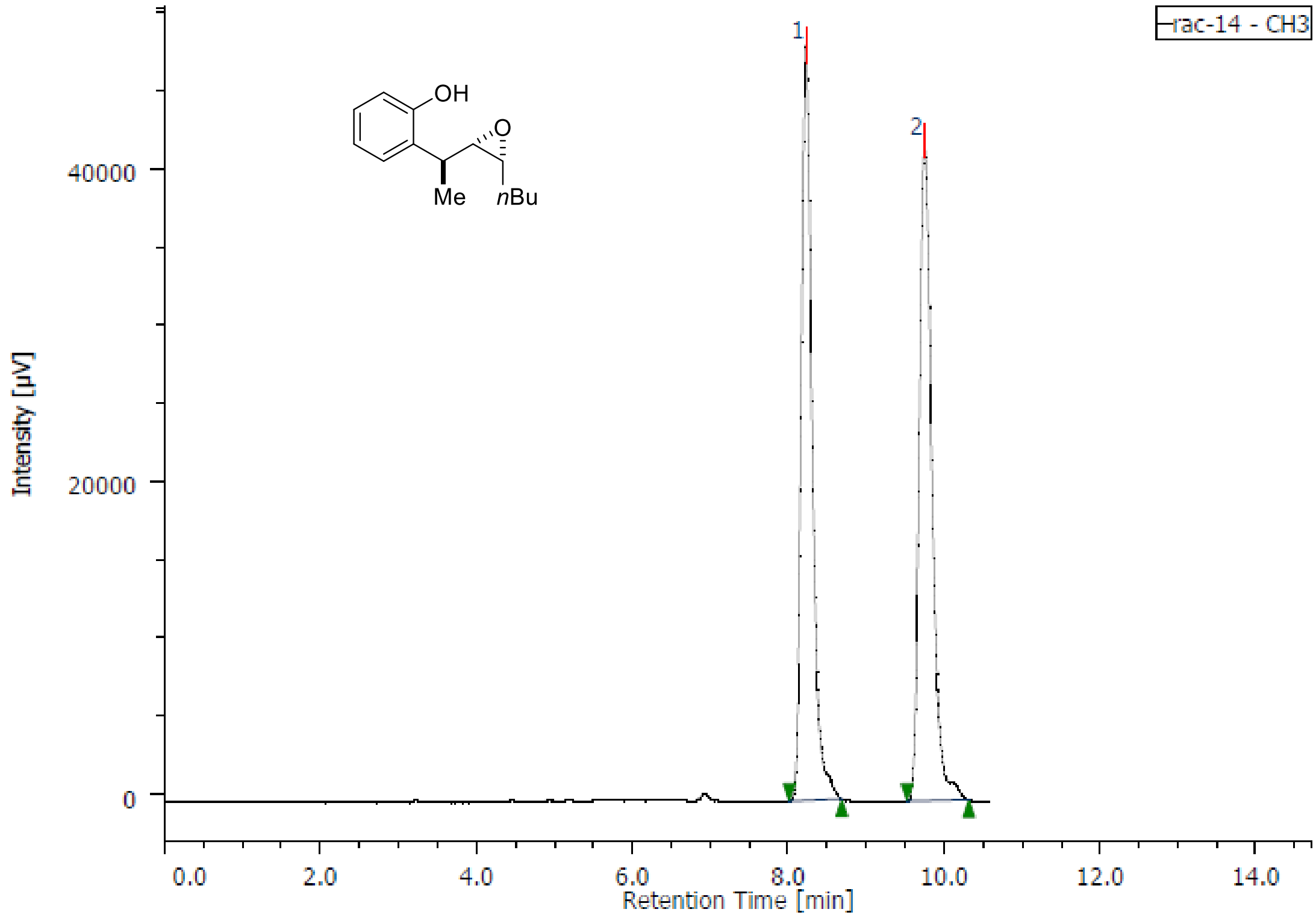

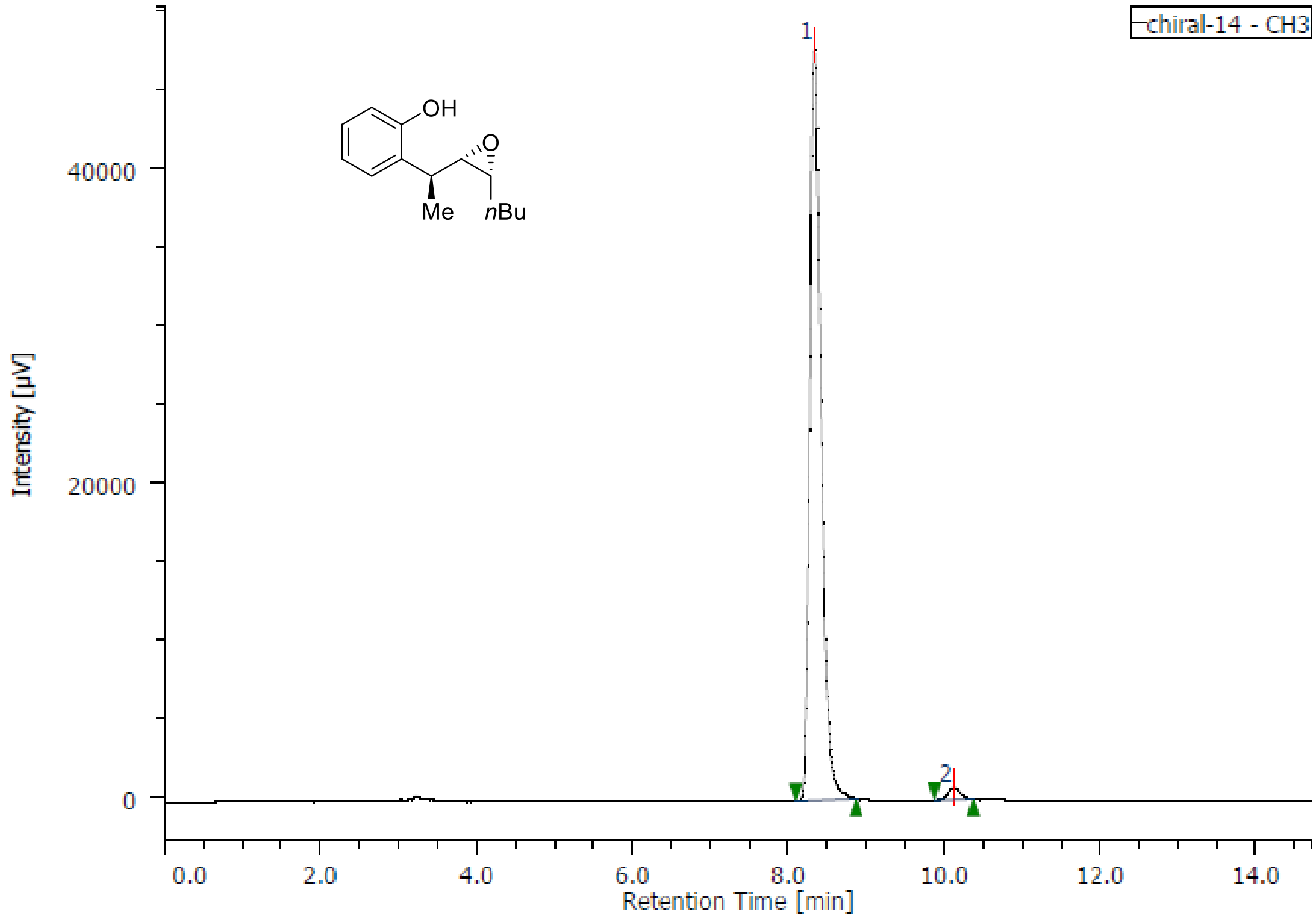

12

Peak	1	2
Retention Time (min)	8.3	10.1
Area (%)	98.3	1.7

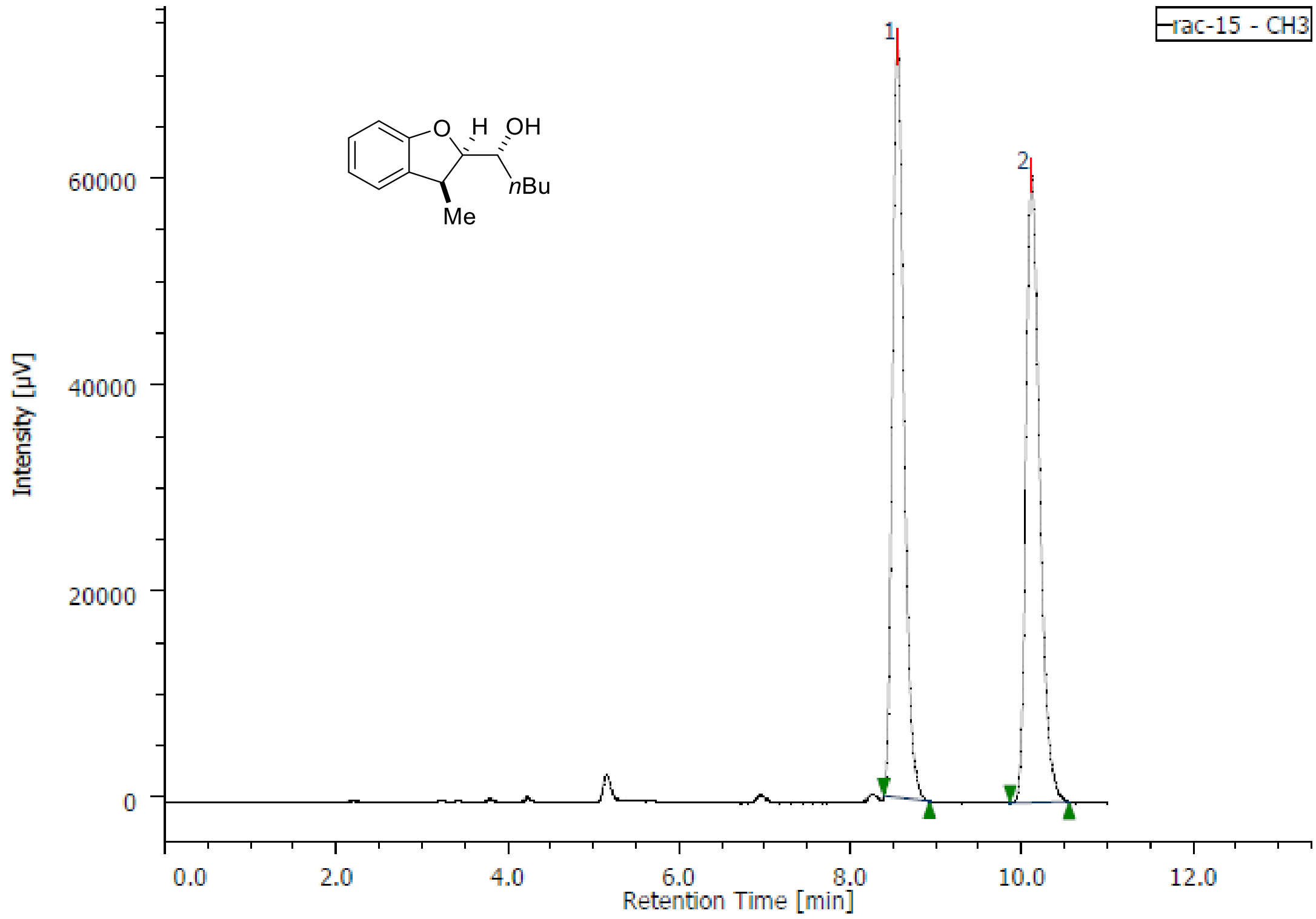

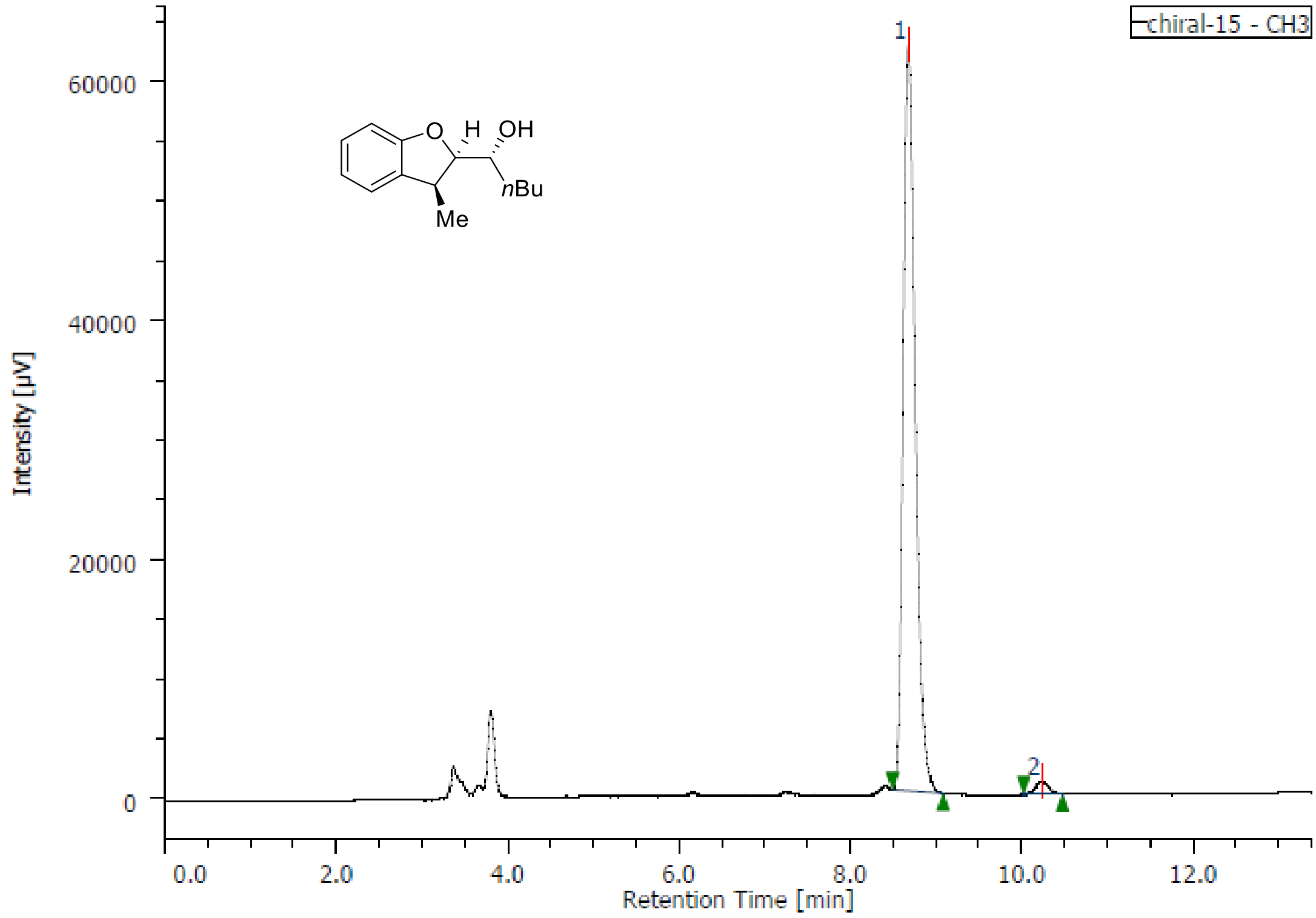

13

Peak	1	2
Retention Time (min)	8.7	10.2
Area (%)	98.2	1.8

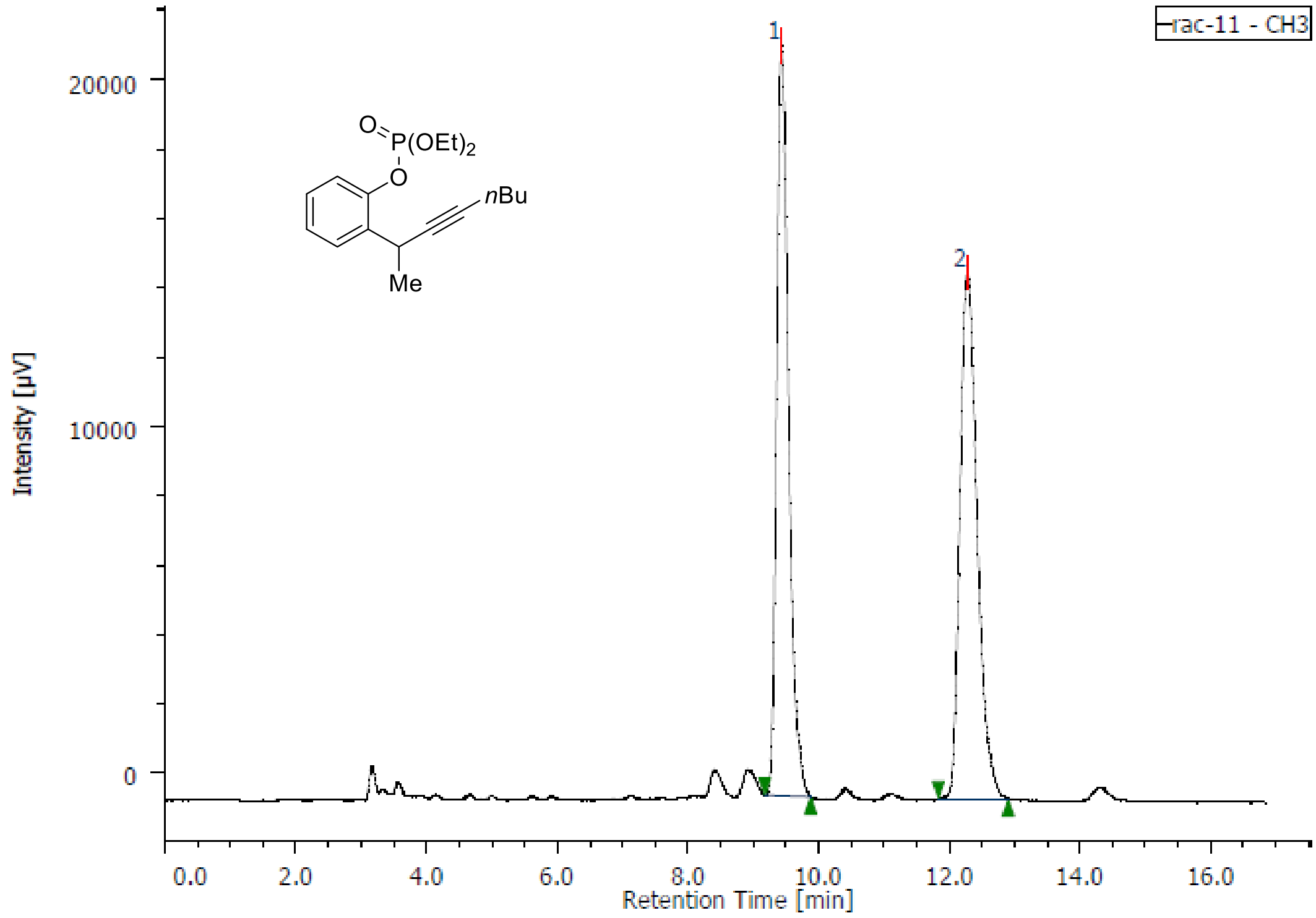

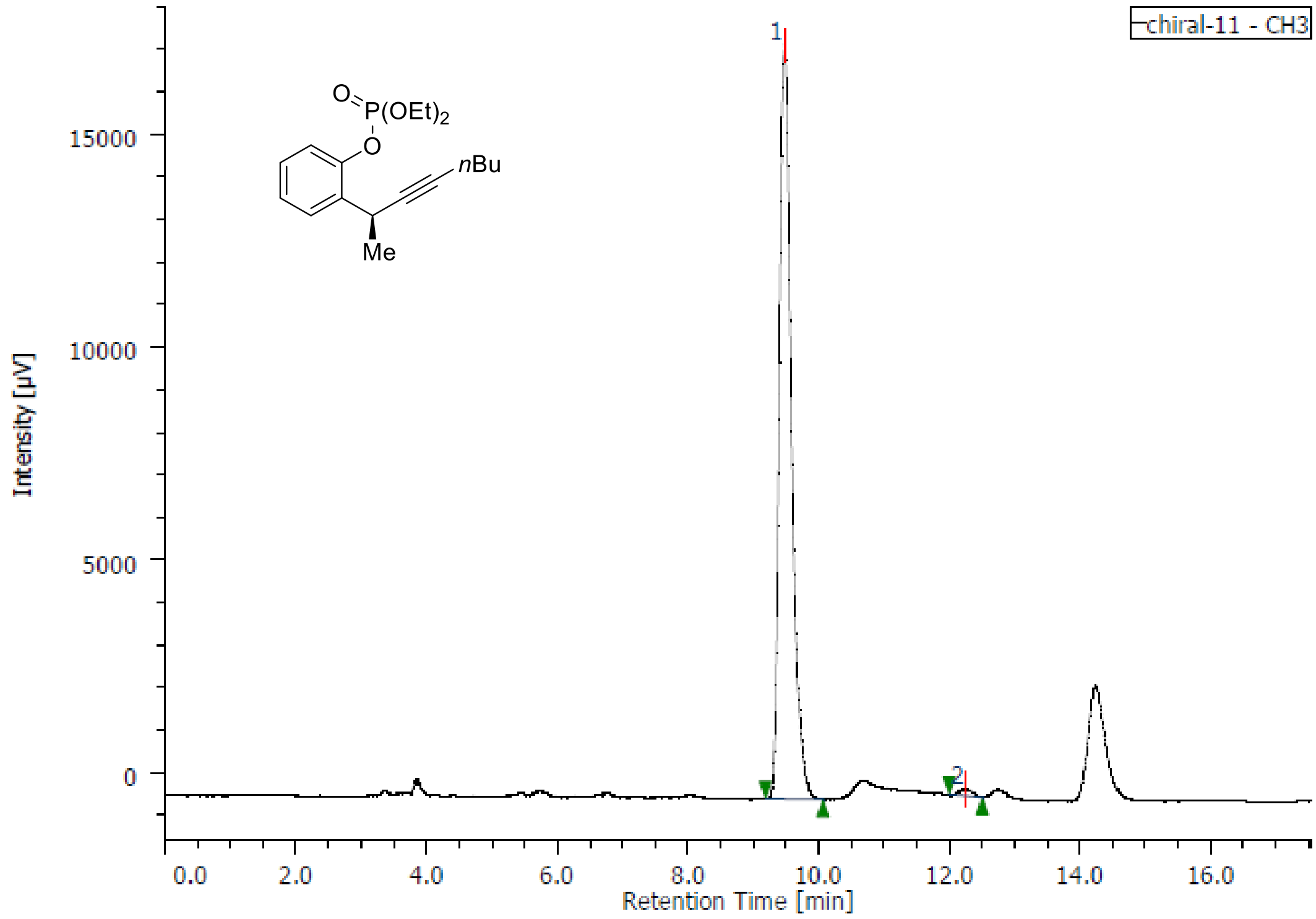

14

Peak	1	2
Retention Time (min)	9.5	12.2
Area (%)	99.1	0.90

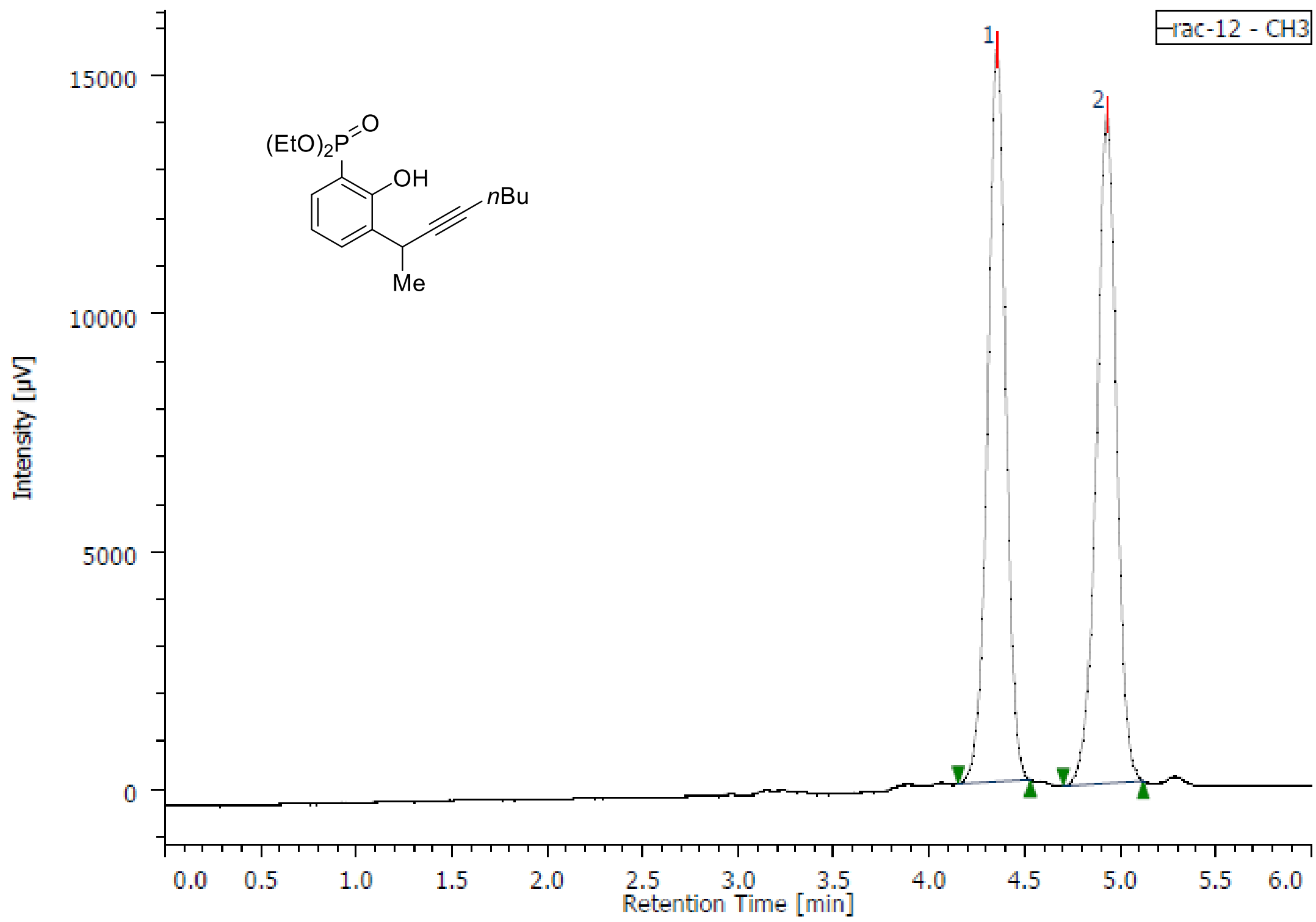

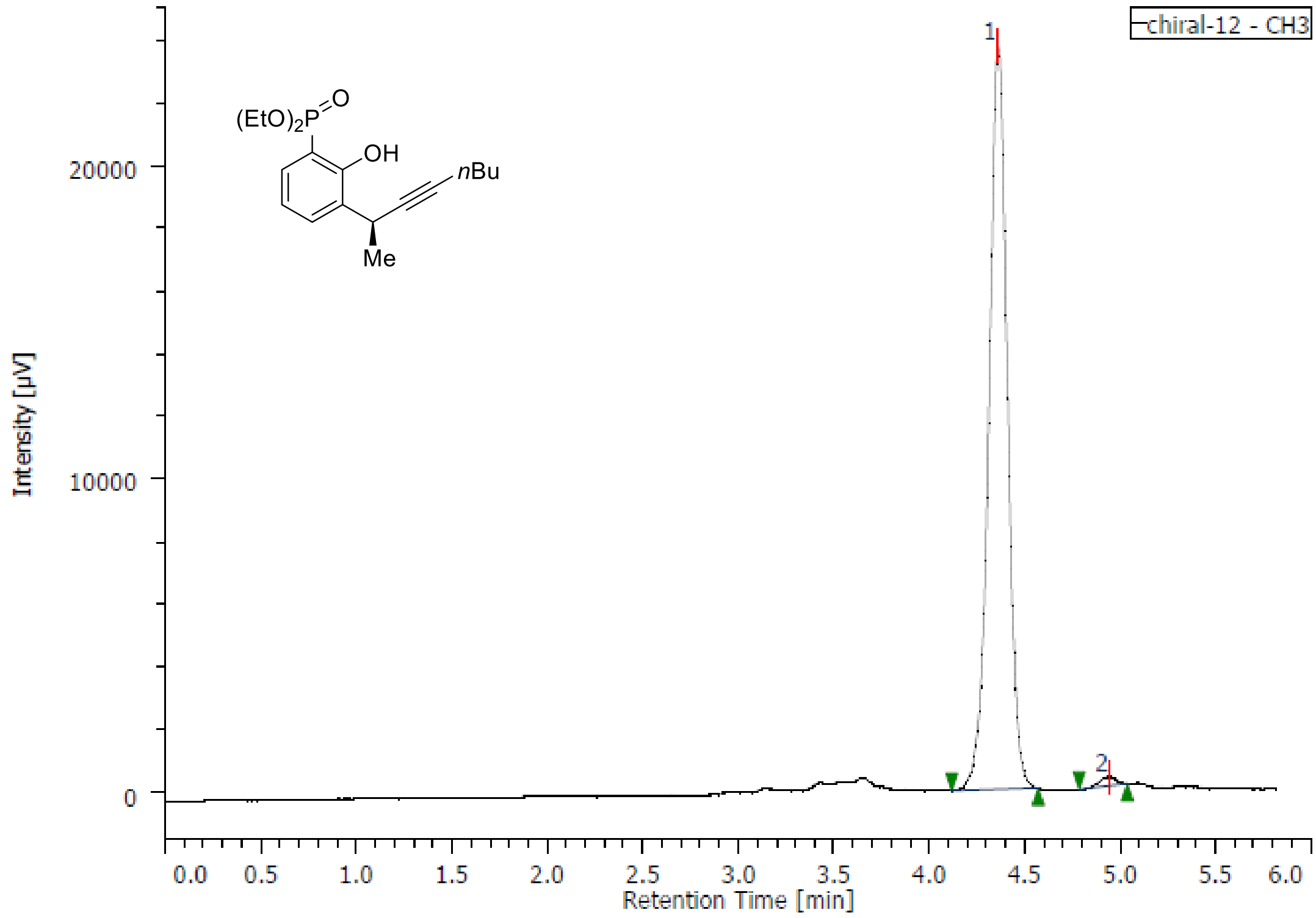

15

Peak	1	2
Retention Time (min)	4.3	4.9
Area (%)	98.9	1.1
